# Sucrose-Induced Proteomic Response and Carbohydrate Utilization of *Lactobacillus sakei* TMW 1.411 During Dextran Formation

**DOI:** 10.3389/fmicb.2018.02796

**Published:** 2018-11-23

**Authors:** Roman M. Prechtl, Dorothee Janßen, Jürgen Behr, Christina Ludwig, Bernhard Küster, Rudi F. Vogel, Frank Jakob

**Affiliations:** ^1^Lehrstuhl für Technische Mikrobiologie, Technische Universität München, Freising, Germany; ^2^Bavarian Center for Biomolecular Mass Spectrometry, Freising, Germany

**Keywords:** *Lactobacillus sakei*, proteomics, genomics, sucrose, dextran, metabolism

## Abstract

*Lactobacillus (L.) sakei* belongs to the dominating lactic acid bacteria in indigenous meat fermentations, while diverse strains of this species have also been isolated from plant fermentations. We could recently show, that *L. sakei* TMW 1.411 produces a high molecular weight dextran from sucrose, indicating its potential use as a dextran forming starter culture. However, the general physiological response of *L. sakei* to sucrose as carbohydrate source has not been investigated yet, especially upon simultaneous dextran formation. To address this lack of knowledge, we sequenced the genome of *L. sakei* TMW 1.411 and performed a label-free, quantitative proteomics approach to investigate the sucrose-induced changes in the proteomic profile of this strain in comparison to its proteomic response to glucose. In total, 21 proteins were found to be differentially expressed at the applied significance criteria (FDR ≤ 0.01). Among these, 14 were associated with the carbohydrate metabolism including several enzymes, which enable sucrose and fructose uptake, as well as, their subsequent intracellular metabolization, respectively. The plasmid-encoded, extracellular dextransucrase of *L. sakei* TMW 1.411 was expressed at high levels irrespective of the present carbohydrate and was predominantly responsible for sucrose consumption in growth experiments using sucrose as sole carbohydrate source, while the released fructose from the dextransucrase reaction was more preferably taken up and intracellularly metabolized than sucrose. Genomic comparisons revealed, that operons coding for uptake and intracellular metabolism of sucrose and fructose are chromosomally conserved among *L. sakei*, while plasmid-located dextransucrase genes are present only in few strains. In accordance with these findings, all 59 different *L. sakei* strains of our strain collection were able to grow on sucrose as sole carbohydrate source, while eight of them exhibited a mucous phenotype on agar plates indicating dextran formation from sucrose. Our study therefore highlights the intrinsic adaption of *L. sakei* to plant environments, where sucrose is abundant, and provides fundamental knowledge regarding the use of *L. sakei* as starter culture for sucrose-based food fermentation processes with *in-situ* dextran formation.

## Introduction

The species *Lactobacillus (L.) sakei* is typically isolated from fermented meat products, which was suggested as its main habitat, since it belongs to the dominating species in spontaneous meat fermentations (Hammes et al., 1990; McLeod et al., 2010). Hence, *L. sakei* spp. are frequently exploited as starter cultures for the manufacturing of fermented meat products (e.g., raw-fermented sausages) (Zagorec and Champomier-Vergès, 2017). Since the main nutrients available for growth in meat products are (purine) nucleosides, certain amino acids (e.g., arginine), glucose and ribose (Champomier-Vergès et al., 2001; Chaillou et al., 2005; Rimaux et al., 2012), the adaption of *L. sakei* spp. to these carbon sources, as well as, associated proteomic profiles and metabolic pathways have been subject of several studies, including growth experiments and -omics approaches (Hammes et al., 1990; Hüfner et al., 2007; Fadda et al., 2010; McLeod et al., 2010, 2011).

However, *L. sakei* has also been isolated from various plant fermentations such as silage and sauerkraut, while it had originally been isolated from rice wine, namely the traditional Japanese beverage *Sake* (Vogel et al., 1993; Torriani et al., 1996; Champomier-Vergès et al., 2001; Amadoro et al., 2015; Prechtl et al., 2018). Above that, some strains of *L. sakei* express glucansucrases that synthesize high molecular weight dextrans from sucrose, which were discussed to contribute to biofilm formation or could be used as fish prebiotics in aquacultures (Gänzle and Follador, 2012; Nácher-Vázquez et al., 2015, 2017a; Prechtl et al., 2018). Thus, the species *L. sakei* seems to be adapted to plant-based environments as well, where sucrose is usually the most abundant carbon source.

Despite its frequent use as a starter culture for the manufacturing of fermented meat products, the capability of some strains to produce exopolysaccharides such as dextran from sucrose has not been commercially exploited yet. In such applications, sucrose would have to be added as carbon source instead of the usually applied glucose, enabling *in-situ* dextran synthesis and thereby the manufacturing of “clean label” products with improved properties (Hilbig et al., unpublished data).

However, little is still known about the general physiological response of dextran-forming *L. sakei* to this carbohydrate and especially about metabolic pathways, which allow intracellular sucrose consumption and could hence compete with dextran formation. To address this lack of knowledge, we sequenced the genome of the dextran-producing strain *L. sakei* TMW 1.411 and performed a label-free proteomic approach for identification of upregulated metabolic pathways upon its growth on sucrose. Moreover, we determined produced and consumed metabolites by *L. sakei* TMW 1.411 during dextran formation, analyzed sucrose metabolic pathways among diverse *L. sakei* strains via comparative genomics and finally correlated the obtained results to the general outcome of the proteomic study.

## Materials and methods

### Chemicals

Chemicals used for growth media and buffers, as well as, solutions for dextran quantification and sample preparation were purchased from Carl Roth GmbH (Karlsruhe, Germany), Merck KGaA (Darmstadt, Germany), and GERBU Biotechnik GmbH (Heidelberg, Germany).

### Bacterial strain and growth conditions

*Lactobacillus sakei* TMW 1.411, originally isolated from sauerkraut, was obtained as a cryo-culture from our strain collection. To recover the cells from cryo-cultures, a modified MRS (mMRS) medium (Stolz et al., 1995) supplemented with the carbohydrates glucose 5 g/L, fructose 5 g/L, and maltose 10 g/L was used for the preparation of agar plates (1.5%) and liquid precultures, whereas incubation was carried out for 36–48 h at 30°C at micro-aerobic conditions in sealed tubes (15 ml; Sarstedt AG & Co, Germany) without shaking. Depending on the performed experiment, the sugars were replaced by other carbohydrates in the working cultures as indicated. To determine viable cell counts (CFU/mL), 50 μL of appropriate dilutions in saline (0.9% NaCl) were spread on mMRS agar with sterile glass beads (2.7 mm, Carl Roth GmbH, Karlsruhe, Germany) and incubated at 30°C for at least 48 h.

### Genome sequencing and annotation

High-molecular-weight DNA was isolated from liquid cultures (late exponential growth phase) in mMRS and purified as previously described (Kafka et al., 2017). To obtain the whole genome shotgun sequence (WGS), the Illumina MiSeq® sequencing technology was applied in combination with the SPAdes 3.9 assembly algorithm. Afterwards, the WGS was annotated with PGAP (prokaryotic genome annotation pipeline) and RAST (rapid annotations using subsystems technology), which included SEED subsystem analysis (Aziz et al., 2008; Tatusova et al., 2016). The RAST annotated genome and open reading frames (ORF) are deposited as online supplementary material, whereas the WGS project has been published (DDBJ/ENA/GenBank) under the accession QOSE00000000. The version described in this paper is version QOSE01000000.

### Proteomic analysis

#### Experimental setup

To investigate the proteomic shift in response to sucrose as sole carbon source, 4 × 15 ml precultures (four biol. replicates) of *L. sakei* TMW 1.411 were prepared in mMRS as described above (section Bacterial Strain and Growth Conditions) and used to inoculate 4 × 100 ml cultures in mMRS (20 g/L glucose) with a final OD_600_ of 0.1. The cultures were grown to the mid-exponential growth phase (pH ~5.0, determined in preliminary experiments), which had given good results in previous experiments (Schott et al., 2017), and subsequently distributed to 50 ml sealed tubes each (eight tubes in total). Afterwards, the cultures were pelletized (5,000 × g, 10 min) and washed once in fresh mMRS. Next, the suspensions were pelletized again and resuspended in an equal volume of mMRS supplemented with either glucose or sucrose (20 g/L each), followed by incubation at 30°C for 2 h. Subsequently, 2.5 mL of cooled trichloroacetic acid (100%) were added to 40 mL of glucose/sucrose treated cultures (6.25% w/v final concentration) and the suspensions were immediately transferred to pre-cooled 50 ml tubes and incubated on ice for 10 min. After centrifugation (5,000 × rpm, 10 min, 4°C), the pellets were washed twice with 10 mL cold acetone (−20°C) (2,000 rpm, 10 min, 4°C), whereas the supernatants were discarded carefully. Finally, the pellets were frozen in liquid nitrogen and stored at −80°C until protein isolation and peptide preparation (section Peptide Preparation, Separation, and Mass Spectrometry). In addition, aliquots were taken from each of the four precultures, as well as, the eight batches after 2 h incubation to determine pH values and the viable cell count in CFU/mL on agar plates.

#### Peptide preparation, separation, and mass spectrometry

Cell pellets were resuspended in lysis buffer [8 M urea, 5 mM EDTA disodium salt, 100 mM NH_4_HCO_3_, 1 mM Dithiothreitol (DTT) in water, pH = 8.0] and disrupted mechanically using glass beads (G8772, 425–600 μm, Sigma, Germany), whereas a Bradford assay (Bio-Rad Protein Assay, Bio-Rad Laboratories GmbH, Munich, Germany) was performed to determine the total protein concentration in the lysate. Afterwards, 100 μg protein extract of each sample were used for in-solution digestion: After reduction (10 mM DTT, 30°C, 30 min) and carbamidomethylation (55 mM chloroacetamide, 60 min in the dark), trypsin was added to the samples, and the solutions were incubated overnight at 37°C. Next, the digested protein samples were desalted using C18 solid phase extraction with Sep-Pak columns (Waters, WAT054960) following the manufacturer's protocol. Finally, the purified peptide samples were dried with a SpeedVac device and dissolved in an aqueous solution of acetonitrile (2%) and formic acid (0.1%) at a final concentration of 0.25 μg/μL.

Peptide analysis was performed on a Dionex Ultimate 3000 nano LC system, which was coupled to a Q-Exactive HF mass spectrometer (Thermo Scientific, Germany). At first, the peptides were loaded on a trap column (75 μm × 2 cm, self-packed, Reprosil-Pur C18 ODS-3 5 μm resin, Dr. Maisch, Ammerbuch) at a flow rate of 5 μL/min in solvent A_0_ (0.1% formic acid in water). Next, the separation was performed on an analytical column (75 μm × 40 cm, self-packed, Reprosil-Gold C18, 3 μm resin, Dr. Maisch, Ammerbuch) at a flow-rate of 300 nL/min applying a 120 min linear gradient (4–32%) of solvent B (0.1% formic acid, 5% DMSO in acetonitrile) and solvent A_1_ (0.1% formic acid, 5% DMSO in water).

The mass spectrometer was operated in the data dependent mode to automatically switch between MS and MS/MS acquisition. The MS1 spectra were obtained in a mass-to-charge (m/z) range of 360–1,300 m/z using a maximum injection time of 50 ms, whereas the AGC target value was 3e6. Up to 20 peptide ion precursors were isolated with an isolation window of 1.7 m/z (max. injection time 25 ms, AGC value 1e5), fragmented by higher-energy collisional dissociation (HCD) applying 25% normalized collision energy (NCE) and finally analyzed at a resolution of 15,000 in a scan range from 200 to 2,000 m/z. Singly-charged and unassigned precursor ions, as well as, charge states >6+ were excluded.

#### Protein identification and quantification

Both identification and quantification of peptides and proteins were performed with the software MaxQuant (v. 1.5.7.4) by searching the MS2 data against all protein sequences predicted for the reference genome of *L. sakei* TMW 1.411 by the RAST annotation pipeline (section Genome Sequencing and Annotation; GenBank QOSE0100000) using the embedded search engine Andromeda (Cox et al., 2011). While the carbamidomethylation of cysteine was a fixed modification, the oxidation of methionine, as well as, the N-terminal protein acetylation were variable modifications. Up to two missed Trypsin/P cleavage sites were allowed and precursor and fragment ion tolerances were set 10 and 20 ppm, respectively. The label-free quantification (Cox et al., 2014) and data matching were enabled within the MaxQuant software between consecutive analyses, whereas filtering of the search results was performed with a minimum peptide length of 7 amino acids, as well as, 1% peptide and protein false discovery rate (FDR) plus common contaminants and reverese identifications.

#### Data processing and statistical analysis

The Perseus software (version 1.6.0.7) was used to process the MaxQuant output file (proteinGroups.txt) and conduct statistical analyses (Tyanova et al., 2016). After filtering of the protein groups (removal of identified by site hits, reverse identifications, contaminants), the LFQ intensity data were log_2_ transformed, whereas the IBAQ intensities were log_10_ transformed. To improve the validity of statistical analysis, only proteins which had been identified (i) by at least two unique peptides and (ii) in all four replicates of at least one group (glucose/sucrose treated cells) were considered, whereas missing values after log-transformation were imputed from a normal distribution (width: 0.2; down shift: 1.8). The log_2_-transformed LFQ data were used for a stringent *t*-Test analysis, using a Benjamini-Hochberg FDR of 0.01 for truncation, whereas proteins with an absolute log_2_ fold change (FC) of ≥1 were further discussed in the present study. To estimate absolute protein abundancies at a certain condition, the transformed IBAQ intensities were averaged ranked descending for each group. The results of the *t*-Test analysis are available as only Supplementary Material.

### Monitoring of growth, metabolite, and dextran formation in mMRs medium

Precultures in mMRS were prepared from single colonies (two biol. replicates) on agar plates as described (section Bacterial Strain and Growth Conditions) and subsequently used to prepare growth series (inoculum: OD_600_ = 0.1) in mMRS medium supplemented with sucrose (50 g/L) as sole carbon source. After various incubation times, characteristic growth parameters such as viable cell count (CFU/mL; 2.2) and pH (Knick 761 Calimatic, Knick, Germany) were determined. Culture supernatants were prepared by centrifugation (5,000 × g, 10 min, 4°C) und subsequently stored at −20°C until metabolite and dextran quantification. Sugars and organic acid concentrations were determined with a HPLC system (Dionex Ultimate 3000, Thermo Fisher Scientific, United States) coupled to Shodex refractive index (RI) detector (Showa Denko Shodex, Germany), whereas 20 μL were injected from prepared samples. For sample preparation, supernatants were either filtered (0.2 μm nylon filters, Phenomenex, Germany) and diluted (analysis of sugars) or treated as follows (analysis of organic acids): 50 μL perchloric acid (70%) were added to 1 mL of supernatant, mixed thoroughly and incubated overnight (4°C). Afterwards, the samples were centrifuged (13,000 × g, 30 min, 4°C) and filtered (0.2 μm). The sugars were measured with a Rezex™ RPM Pb^2+^ column at a flow-rate of 0.6 mL/min (85°C) using filtered (0.2 μm) deionized water as eluent, whereas organic acids were measured with a Rezex™ ROA H^+^ column (both Phenomenex, Germany) at a flow-rate of 0.7 mL/min (85°C) with 2.5 mM H_2_SO_4_ (prepared with filtered, deionized water). Metabolites were identified and quantified by means of appropriate standard solutions with the Chromeleon™ software (v. 6.8; Dionex, Germany).

Dextran quantification was performed from dialyzed supernatants (3.5 kDa cut-off dialysis tubings, MEMBRA-CEL®, Serva Electrophoresis GmbH, Germany) using the phenol sulfuric acid (PSA) method as described previously (Prechtl et al., 2018). To ensure the reliable removal of sucrose, dialysis was performed over 48 h (4°C) with continuous stirring, whereas the water (5 L exchange volume, ~100 × dilution factor) was changed at least five times. As blank value, non-inoculated mMRS medium was dialyzed in the same way, subjected to PSA quantification and finally subtracted from the amount of dextran quantified in the samples.

### Data deposition

An additional file containing the protein sequences with corresponding FIG identifiers (from RAST annotation), as well as, relevant proteome tables with assigned SEED categories and the results of the *t*-Test evaluation are deposited online as supplementary data. The mass spectrometry proteomics data have been deposited to the ProteomeXchange Consortium via the PRIDE partner repository with the dataset identifier PXD011417 (http://proteomecentral.proteomexchange.org).

## Results

### General features of the genome of *L. sakei* TMW 1.411

Prior to performing the proteomic experiment, the genome of *L. sakei* TMW 1.411 was sequenced and annotated as described in the materials and methods section (Genome Sequencing and Annotation).

The obtained WGS of *L. sakei* TMW 1.411 comprises 41 contigs. A genome size of ca. 1.9 Mb with a GC content of 41.0% was predicted, which is in the usual range of different genome-sequenced strains of *L. sakei*. Additional information including the genome coverage, the number of predicted coding sequences (CDS) and RNAs is presented in Table 1. The contigs seq28, seq32, and seq36 could be circularized due to sequence overlaps, and manual BLASTn analysis of the processed sequences confirmed a high nucleotide sequence identity (90–99%) with known plasmids of *L. sakei* and *L. curvatus* species (Table 2).

**Table 1:** General features of the sequenced genome of *L. sakei* TMW 1.411.

Coverage	200x
Number of contigs	41
Genome size (bp)	1,944,239
GC content (%)	41.0
Number of CDS	1,912
Number of RNAs	63
Number of put. Plasmids	3
Origin	Sauerkraut
GenBank ac. no.	QOSE00000000

**Table 2:** WGS sequence contigs of *L. sakei* TMW 1.411 with assigned plasmid names, identified homologs, and related information.

**Contig/plasmid**	**Homolog** **plasmids**	**Size (bp)**	**Strain**	**Isolation source**	**Identity/** **coverage**	**GenBank accession number**
Contig: seq28 Plasmid: p-1.411_1 Size: 11,246 bp Ac. No. QOSE01000028	pMN1	11,126	*L. sakei* MN1	Fermented meat product	99%/100%	MF590088.1
	plasmid 4	11,068	*L. sakei* FLEC01	Human feces	99%/100%	LT960780.1
	plasmid 4	11,156	*L. sakei* MFPB19	Beef carpaccio	99%/100%	LT960787.1
	pJ112C	10,871	*L. sakei* J112	French dry-type Pork sausage	99%/100%	OBHN01000041.1
	pJ156C	10,871	*L. sakei* J156	French dry-type Pork sausage	99%/100%	OBHL01000056.1
	p-1.1928_3	11,381	*L. curvatus* TMW 1.1928	Raw-fermented sausage	99%/100%	CP031006
Contig: seq32 Plasmid: p-1.411_2 Size: 6,593 bp Ac. no. QOSE01000041	pKCA15	14,826	*L. sakei* KCA311	n.a.	91%/58%	KF559313.1
Contig: seq36 Plasmid: p-1.411_3 Size: 2,499 bp Ac. no. QOSE01000040	pLC2	2,489	*L. curvatus* LTH683	Raw-fermented sausage	99%/100%	Z14234.1
	p-1.1928_4	2,627	*L. curvatus* TMW 1.1928	Raw-fermented sausage	99%/100%	CP031007

Among these plasmids, seq28 (denoted as plasmid p-1.411_1) harbored a 5.3 kb ORF encoding a predicted glucansucrase of the glycoside hydrolase (GH) family 70 (gene locus DT321_09485). The corresponding amino-acid sequence was almost identical (>95% identity and >95% coverage) to those of known dextransucrases (Dsr) in *L. sakei* and *L. curvatus* species, such as DsrLS (ac. no. ATN28243) and GtfKg15 (ac. no. AAU08011.1), or Gtf1624 (ac. no. CCK33643) and Dsr11928 (ac. no. AXN36915.1), respectively. The main difference between the amino-acid sequences was the length of an alanine-rich amino acid repeat, which formed a putative linker segment between the GH70 domain and the C-terminal cell-wall anchor motif (LPxTG).

Ca. 14% of all SEED category assignments accounted for the metabolism of carbohydrates, including mono-, di- and oligosaccharides, as well as, amino-sugars and sugar alcohols (Table S1). Among these, the genes associated with sucrose and fructose metabolism were considered to be most relevant for a growth of *L. sakei* TMW 1.411 on sucrose as sole carbon source, since fructose is released by dextransucrases concomitantly with dextran polymerization. In both cases, the corresponding genes were arranged in an operon (Table 3).

**Table 3:** Sucrose and fructose operons of *L. sakei* TMW 1.411 with associated genes and predicted functions according to RAST annotation.

**Operon**	**Gene**	**Function**	**Contig**	**FIG identifier**	**Gene locus**
Sucrose	*scrR*	Sucrose operon repressor	seq5	fig|1664.9.peg.1584	DT321_04270
	*scrB*	Sucrose-6-phosphate hydrolase	seq5	fig|1664.9.peg.1585	DT321_04275
	*scrA*	PTS beta-glucoside transporter subunit IIBCA	seq5	fig|1664.9.peg.1586	DT321_04280
	*dexB*	Glucan 1,6-alpha-glucosidase	seq5	fig|1664.9.peg.1587	DT321_04285
	*scrK*	Fructokinase	seq5	fig|1664.9.peg.1588	DT321_04290
Fructose	*fruA*	PTS fructose transporter subunit IIABC	seq3	fig|1664.9.peg.1236	DT321_02745
	*fruK*	1-phosphofructokinase	seq3	fig|1664.9.peg.1237	DT321_02750
	*fruR*	Fructose operon repressor	seq3	fig|1664.9.peg.1238	DT321_02755

*The gene names were assigned according to the strain L. sakei ssp. sakei 23K. The gene loci refer to the deposited WGS (GenBank ac. no. QOSE00000000), whereas the FIG identifiers refer to the RAST annotation which was used for the evaluation of proteomic data (file is provided as online supplement)*.

Manual BLASTn analysis revealed, that among all currently 38 genome-sequenced *L. sakei* strains solely *L. sakei* LK-145 lacks the sucrose operon (Table S2), while each deposited *L. sakei* genome contains the fructose operon. On the contrary, only four of these strains comprised a dextransucrase gene (*L. sakei* FLEC01, MFPB19, J112, J156). In all these strains, the genes were encoded on plasmids, whose nucleotide sequences were nearly identical (Table 2). Metabolization of sucrose by *L. sakei* was further confirmed in preliminary growth experiments on agar-plates, since all of 59 tested strains of *L. sakei* of our strain collection were able to grow on sucrose as sole carbon source. Moreover, eight strains including *L. sakei* TMW 1.411 produced mucous substances (most likely EPS) from sucrose.

### Generation and evaluation of the proteomic data set

**Figure 1:**
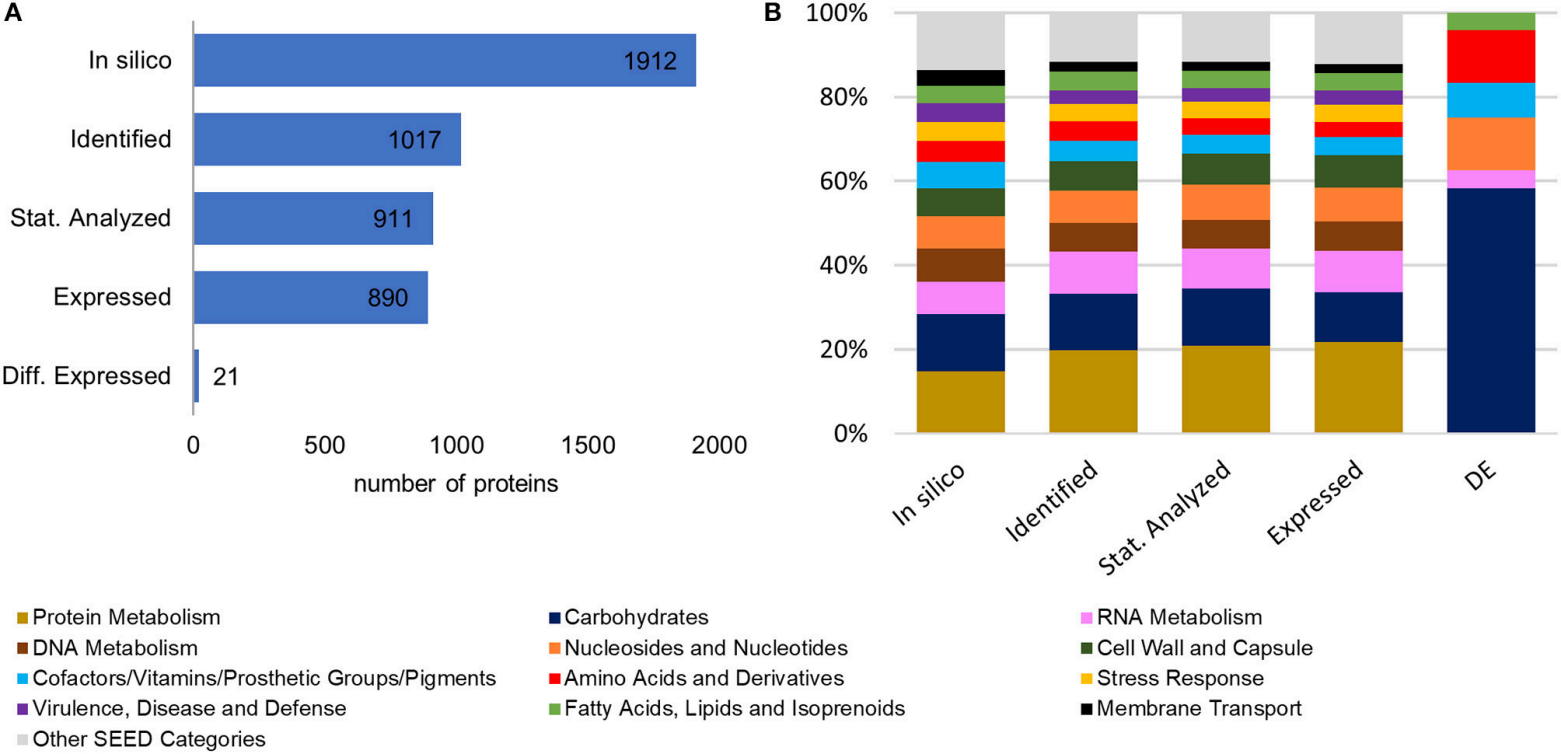
Comparison of the *in-silico* proteome with the protein sub-groups generated by data filtering steps. **(A)** Total protein numbers of *in-silico* predicted proteins, identified proteins (according to quality criteria described in 2.4.4), statistically analyzed proteins (detected in all four replicates of at least one group), expressed proteins, differentially expressed proteins **(B)** Corresponding SEED category distributions. The SEED subsystem proteome coverage was ca 50%.

A scheme of the chosen approach, which describes the performed experimental steps to investigate the proteomic changes of *L. sakei* TMW 1.411 after a switch to sucrose as sole carbon source, is presented in Figure S1. To evaluate the plausibility of the generated data set and exclude a bias for distinct protein categories during the sequential data filtering steps, the *in-silico* proteome of *L. sakei* TMW 1.411 was compared with the protein sub-groups created during data filtering with respect to protein numbers and corresponding SEED categories, which had been derived from RAST annotations (Figure 1).

Compared to the *in-silico* proteome of *L. sakei* TMW 1.411, which comprised 1912 putative proteins according to the number of coding sequences (CDS) predicted by RAST annotation, 1017 proteins could be identified based on the applied quality criteria described in 2.4.4. This resulted in a proteome coverage of ~53%, which is in the typical range of label-free quantitative proteomics approaches (Liu et al., 2014). To further increase the accuracy of statistical evaluation, only proteins detected in all four replicates of a group (glucose or sucrose) were considered for statistical analysis (section Data Processing and Statistical Analysis), which amounted to a subset of 911 proteins, whose expression levels were compared between both groups by statistical analysis. The SEED category distributions were similar for all protein sub-groups (except for the differentially expressed proteins being addressed in 3.3). Therefore, a potential bias for any protein category during the data filtering steps could be excluded (Figure 1B).

Another important measure to ensure the validity of the proteomic experiment was to confirm equal viable cell counts and pH values in both groups (glucose and sucrose) prior to protein isolation, as any variation could have been the result of non-uniform cell growth/death or acidification during the 2 h of incubation, which might have influenced the proteomic profiles as well. Thus, the average viable cell count was determined in both batch groups and the values (glucose: 1.3 ± 0.2 × 10^8^ CFU/mL; sucrose: 1.6 ± 0.5 × 10^8^ CFU/mL) were demonstrated to be statistically not different (*t*-Test, *p* = 0.05). As for the pH values, the cells incubated in sucrose containing mMRS medium (pH = 4.17 ± 0.02) showed a slightly weaker acidification compared to the reference batch in glucose containing mMRS medium (pH = 4.10 ± 0.01). Although the mean difference was only 0.07 pH units, statistical analysis (two sample *t*-test, Table S3) revealed it to be significant (*p* = 0.01).

### Comparison of the proteomic states associated with growth in glucose and sucrose

**Figure 2:**
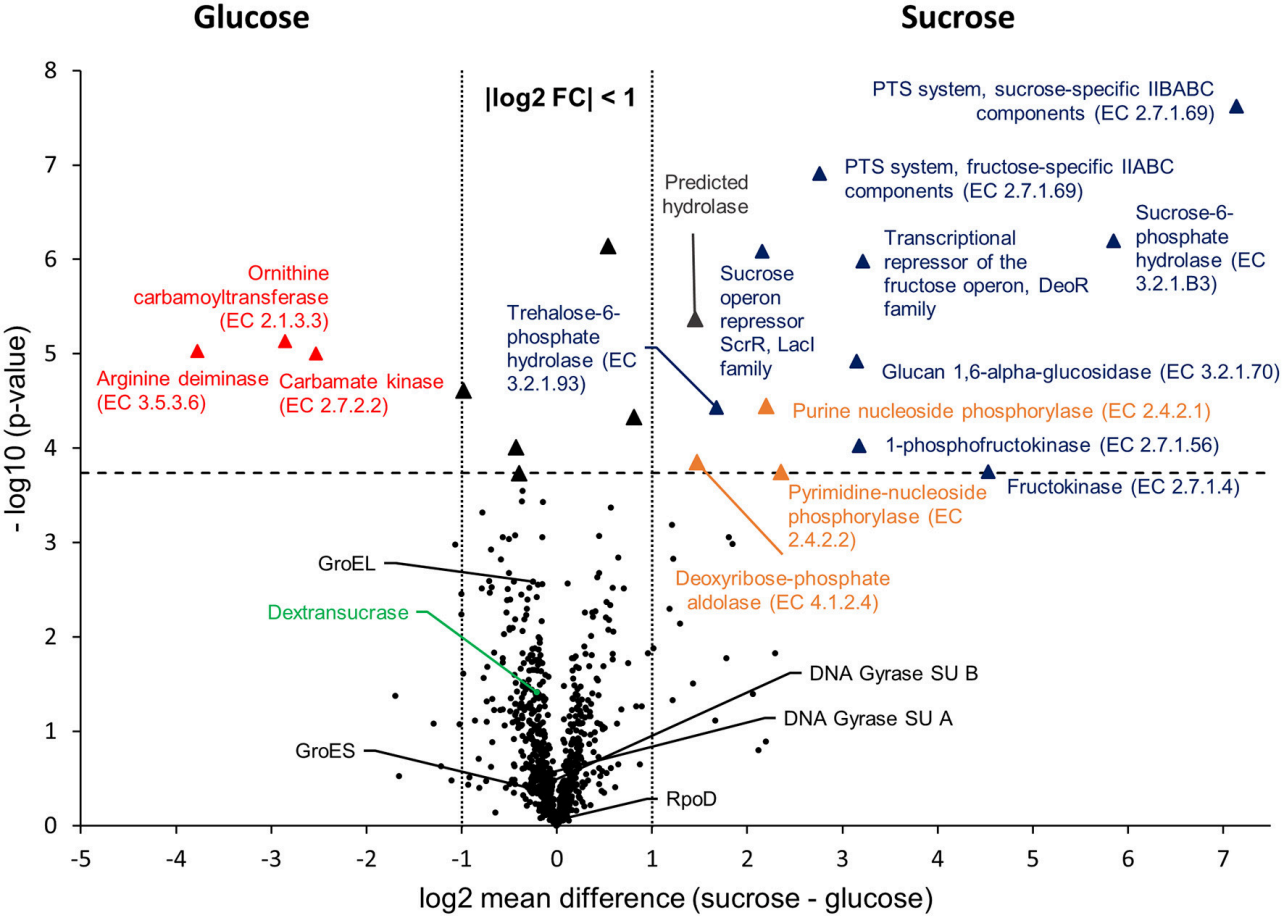
Volcano-Plot of the -Test comparison of 911 quantified proteins, which had been isolated from cultures incubated in mMRS with glucose and sucrose as sole carbon sources, respectively. The dashed horizontal line indicates the FDR threshold of 0.01, whereas dotted vertical lines enclose an area with a log2 fold change of −1 ≤ x ≤ 1. Black dots below the FDR threshold indicate non-differentially expressed proteins with a selection of common house-keeping proteins (GroEL, GroES, DNA Gyrase subunits A, B, RpoD) and the dextransucrase, being marked in black and green, respectively. Triangles indicate statistically differentially expressed proteins, whereas different colors were used to group the proteins: red = Amino acids and derivatives (SEED category); blue = Carbohydrates (SEED category); orange = Nucleosides and nucleotides (SEED category); black: absolute log2 FC < 1 (not addressed in this study); gray: predicted hydrolase (no SEED category assigned). Further information about differentially expressed proteins are provided in Table 4 and as online supplementary data (Table S3).

To compare the proteomic profiles of the cultures incubated in glucose and sucrose, respectively, the log_2_ transformed LFQ intensities of 911 proteins (Figure 1) were compared between both groups applying a stringent statistical analysis (*t*-Test with Benjamini-Hochberg FDR ≤ 0.01, 2.4.4). The results are visualized in a volcano-plot (Figure 2).

**Figure 3:**
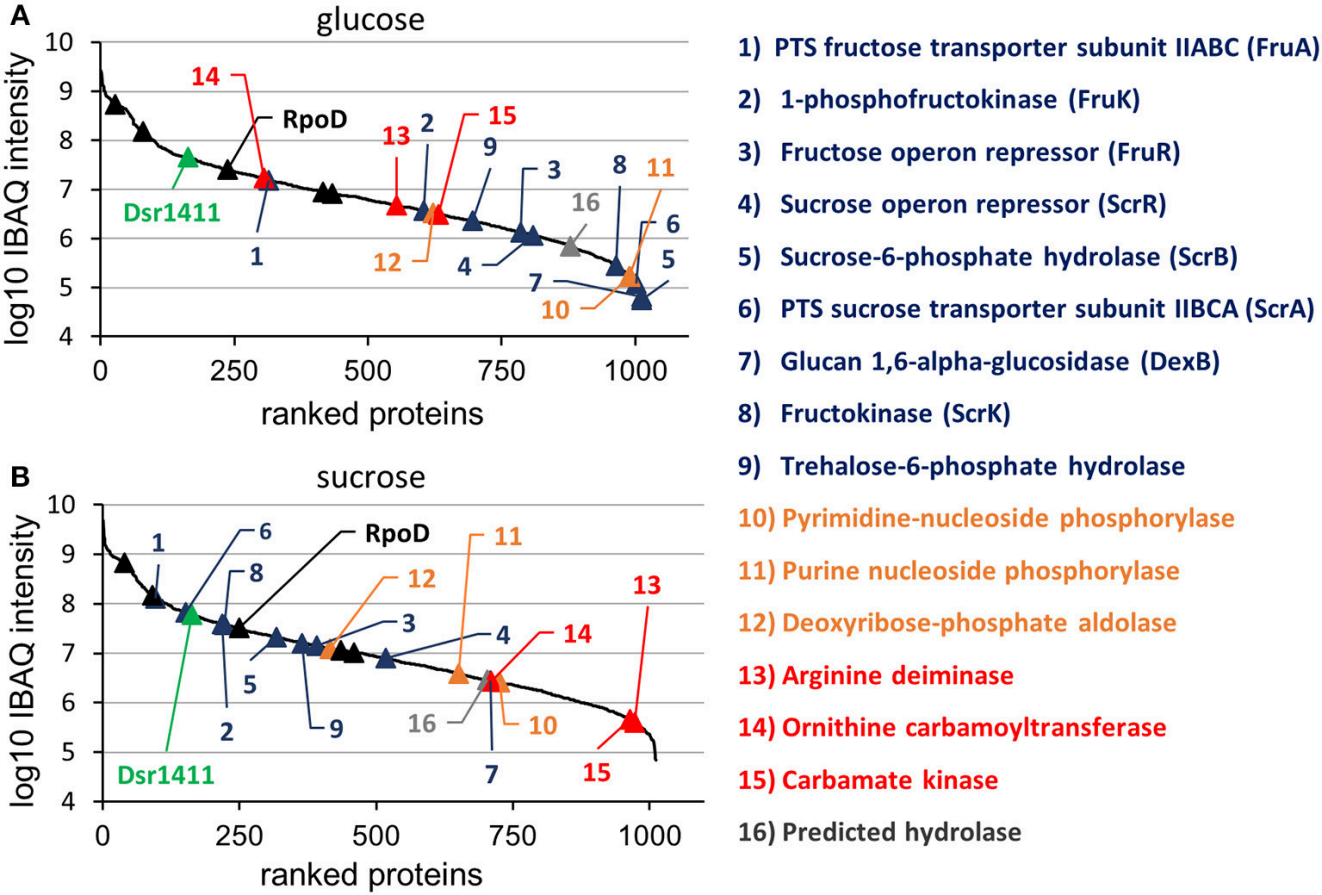
Log10 transformed IBAQ intensities of identified proteins, which had been isolated from cultures incubated in glucose **(A)** and sucrose **(B)**. The colors were used according to Figure 3, except for the housekeeping proteins, which were marked with black triangles. Further information (e.g., IBAQ values, FIG identifiers) are presented in Table S1.

At the applied statistical criteria (section Data Processing and Statistical Analysis), 21 proteins were found to be differentially expressed in cells incubated with glucose or sucrose as sole carbon source, whereas 16 displayed an absolute log_2_ FC of >1 and will be further discussed in this study. As reflected by the SEED category distribution of the differentially expressed proteins (Figure 1B), ~60% of the assigned categories were associated with the metabolism of carbohydrates. This included the genes of the sucrose and fructose operon, respectively, which were up-regulated in sucrose incubated cells, whereas the highest log_2_ FC (7.1 and 5.8) were observed for the characteristic enzymes of the sucrose metabolic pathway, namely the PTS sucrose transporter subunit and the sucrose-6-phosphate hydrolase (Figure 2 and Table 4). Interestingly, although being significantly upregulated in the sucrose treated cells, the proteins of the fructose operon showed a relatively high abundance in the glucose treated cells as well, as suggested by the IBAQ intensities (Figure 3), which can be used to estimate absolute proteome-wide protein abundances (Schwanhäusser et al., 2011; Ahrné et al., 2013).

**Table 4:** Log_2_ FCs, *p*-values (-log_10_) and related information of differentially expressed proteins (Benjamini-Hochberg FDR ≤ 0.01).

**Log_2_ FC**	**–log_10_ (p-value)**	**Function**	**SEED Subsystem**	**FIG identifier**	**Gene loci**
**–**3.8	5.03	Arginine deiminase	Arginine Deiminase Pathway	fig|1664.9.peg.1732	DT321_05025
−2.9	5.13	Ornithine carbamoyl transferase	Arginine Deiminase Pathway	fig|1664.9.peg.1733	DT321_05030
−2.5	5.01	Carbamate kinase	Arginine Deiminase Pathway	fig|1664.9.peg.1734	DT321_05035
1.5	5.37	Predicted hydrolase	n.a.	fig|1664.9.peg.175	DT321_00915
1.5	3.85	Deoxyribose-phosphate aldolase	Deoxyribose and Deoxynucleoside Catabolism	fig|1664.9.peg.1010	DT321_08545
1.7	4.43	Trehalose-6-phosphate hydrolase	Trehalose Uptake and Utilization	fig|1664.9.peg.1859	DT321_05660
2.2	6.08	Sucrose operon repressor ScrR	Sucrose utilization	fig|1664.9.peg.1584	DT321_04270
2.2	4.44	Purine nucleoside phosphorylase	Deoxyribose and Deoxynucleoside Catabolism	fig|1664.9.peg.1008	DT321_08535
2.4	3.74	Pyrimidine-nucleoside phosphorylase	Deoxyribose and Deoxynucleoside Catabolism	fig|1664.9.peg.1004	DT321_08515
2.8	6.91	PTS system, fructose-specific IIABC components	Fructose utilization	fig|1664.9.peg.1236	DT321_02745
3.2	4.92	Glucan 1,6-alpha-glucosidase	n.a.	fig|1664.9.peg.1587	DT321_04285
3.2	4.03	1-phosphofructokinase	Fructose utilization	fig|1664.9.peg.1237	DT321_04250
3.2	5.98	Transcriptional repressor of the fructose operon	Fructose utilization	fig|1664.9.peg.1238	DT321_04255
4.5	3.75	Fructokinase	Fructose/Sucrose utilization	fig|1664.9.peg.1588	DT321_04290
5.9	6.20	Sucrose-6-phosphate hydrolase	Sucrose utilization	fig|1664.9.peg.1585	DT321_04275
7.1	7.62	PTS system, sucrose-specific IIBCA components	Sucrose utilization	fig|1664.9.peg.1586	DT321_04280
**–**0.4	3.74	3-ketoacyl-CoA thiolase	Biotin biosynthesis; Butanol Biosynthesis; Fatty acid metabolism; Isoprenoid Biosynthesis	fig|1664.9.peg.116	DT321_00615
0.5	6.14	Pyruvate formate-lyase	Butanol Biosynthesis; Fermentations: Mixed acid	fig|1664.9.peg.1310	DT321_03110
−0.9	4.61	Dihydrofolate reductase	5-FCL-like protein; Folate Biosynthesis	fig|1664.9.peg.1437	DT321_03535
−0.4	4.01	dipeptidase	n.a.	fig|1664.9.peg.402	DT321_06170
0.8	4.33	Inosine-uridine preferring nucleoside hydrolase	Purine conversions; Queuosine-Archaeosine Biosynthesis	fig|1664.9.peg.662	DT321_07450

*Negative log_2_ FC values indicate higher abundance in glucose treated cells, whereas positive values indicate higher abundance in sucrose treated cells. The predicted functions and assigned SEED subsystems were derived from RAST annotation (FIG identifiers). The gene loci refer to the deposited WGS sequence (accession number QOSE00000000)*.

Three enzymes associated with the catabolism of deoxynucleosides (Figure 2, orange), as well as, the trehalose-phosphate hydrolase and a predicted hydrolase also showed an increased expression in sucrose treated cells. The enzymes of the arginine-deiminase pathway were found to be more abundant in glucose treated cells, which either suggested a sucrose-induced downregulation or a glucose mediated upregulation (Figure 2, red).

Apart from that, the expression levels of the dextransucrase Dsr1411 were compared for both carbon sources, since sucrose is the natural substrate of this enzyme and thus could have a positive impact on its expression. However, this enzyme was not differentially expressed (Figure 2, green). Moreover, an evaluation of the IBAQ intensities pointed at relatively high amounts of this enzyme within the cellular proteome—irrespective of the present carbon source (Figure 3, green).

To demonstrate the validity of the experiment, the *t*-Test results for the expression of five common housekeeping proteins (GroEL/ES, RpoD, DNA Gyrase Subunits A/B), which was expected to be independent of the present carbon source, were highlighted in the Volcano-Plot (Figure 2, black descriptors). Additional Supplementary Information about the differentially expressed proteins and a detailed summary of the *t*-Test evaluation are provided in Table 4 and as online supplementary data (Table S3).

### Monitoring of sugar consumption, as well as, lactate and dextran formation during growth on sucrose

**Figure 4:**
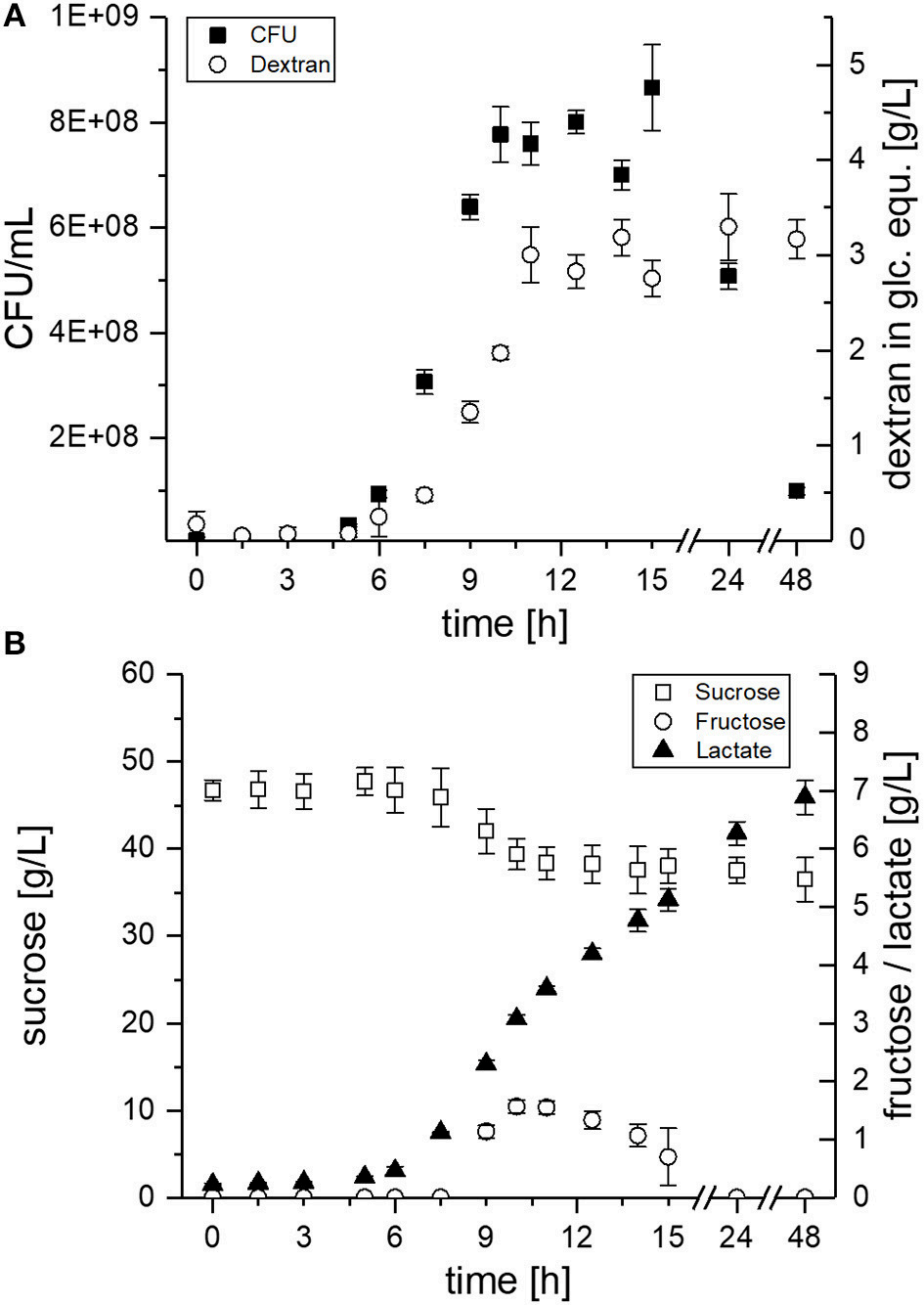
EPS and metabolite concentrations during growth of *L. sakei* TMW 1.411 in mMRS medium supplemented with 50 g/L sucrose. **(A)** Viable cell count (■) and dextran concentration (°) in glucose equivalents (glc. equ.). **(B)** Sucrose (□), fructose (°) and lactate (▴) concentrations. Error bars are standard deviations of triplicate measurements from **two** biological replicates (*n* = 6).

The proteomic experiment (3.2 + 3.3) gave insights into the basic response of *L. sakei* TMW 1.411 to sucrose at an early stage of growth (after 2 h incubation in sucrose containing mMRS). In this way, the differential expression of sucrose-metabolizing pathways could be detected. To further investigate sucrose utilization under common EPS production conditions (Prechtl et al., 2018), metabolite and dextran concentrations were monitored during growth in mMRS medium over 48 h (Figure 4).

The CFU of *L. sakei* TMW 1.411 increased after ca. 6 h, which was accompanied by dextran synthesis, sucrose consumption and lactate formation during the exponential growth phase (Figure 4A + B). Fructose was detectable for the first time after 9 h of cultivation and reached a maximum after 10 h (ca. 1.6 g/L; Figure 4B). Afterwards, the fructose concentration decreased until it was depleted after 24 h, whereas the sucrose concentration stayed more or less constant at 39 g/L between 10 and 24 h, and finally showed another slight decrease to 37 g/L after 48 h. The fructose concentrations lay always below the dextran concentrations, although the amount of fructose released during dextran synthesis and the produced amount of dextran in glucose equivalents (glc. equ.) should be stoichiometrically identical, if released fructose reflected the total dextransucrase activity. In total, ca. 10 g/L sucrose were consumed during the 48 h of fermentation, whereas about 3 g/L dextran were produced. Considering the theoretical maximum possible amount of ~5 g/L dextran (< 50% of the consumed sucrose due to one released fructose + water molecule per transferase reaction), this resulted in a dextran yield of roughly 60%. Glucose, which could possibly be released by the hydrolysis activity of dextransucrases, as well as, fermentation products such as acetate or ethanol could not be detected in the culture supernatant.

## Discussion

### Genetic adaption of *L. sakei* to plant environments

Although *L. sakei* belongs to the dominating species in indigenous meat fermentations, where glucose and ribose are the predominating carbohydrates (Champomier-Vergès et al., 2001), the operons for both sucrose and fructose utilization are strikingly conserved among *L. sakei* spp. as demonstrated in the present work (section General Features of the Genome of *L. sakei* TMW 1.411). Furthermore, a predicted glucan-1,6-α-glucosidase (DexB) is located within the sucrose operon, which was demonstrated to hydrolyze isomaltose (degradation of starch) in *L. acidophilus* (Møller et al., 2012). Since these carbohydrates are commonly available in plants, this suggests an adaption of this species to plant-based environments, including the digestive organs of plant feeding organisms. Thus, it is not surprising that several species have been isolated from fermented plant products (Vogel et al., 1993; McLeod et al., 2011), including *L. sakei* TMW 1.411, which had originally been isolated from a sauerkraut fermentation.

Above that, genomic analyses revealed several *L. sakei* strains including TMW 1.411 to harbor a highly homologous ~11 kb plasmid encoding a cell wall-bound dextransucrase (section General Features of the Genome of *L. sakei* TMW 1.411; Table 2), which uses sucrose as substrate for polymer synthesis. Interestingly, the same plasmid was found in a strain of the closely related species *L. curvatus*. As dextran formation is responsible for biofilm formation in diverse LAB species (Leathers and Cote, 2008; Walter et al., 2008; Zhu et al., 2009; Leathers and Bischoff, 2011; Nácher-Vázquez et al., 2017a; Fels et al., 2018; Xu et al., 2018), its production could protect *L. sakei* against desiccation and could enable surface adhesion, providing an advantage in the colonialization of plants (Cerning, 1990; Badel et al., 2011; Zannini et al., 2016). In a meat-based environment, however, the expression of dextransucrases is unlikely to provide any advantages, since sucrose is not available and above that, only a small fraction of *L. sakei* strains is carrying the corresponding plasmid (Table S2). Nevertheless, it was reported to be a stably inherited low-copy number plasmid, which was attributed to its *repA/B* based replication mechanism and a possible toxin-antitoxin system. The dextransucrase gene has been proposed to have integrated through a transposition process (Nácher-Vázquez et al., 2017b). This might once have led to a selective advantage in habitats, where sucrose is the predominant carbon source, such as in plants or in e.g., plant sap sucking insects.

### Sucrose independent expression of the dextransucrase Dsr1411

While in *Leuconostoc* spp. and *Weissella* spp. the expression of glucansucrases (e.g., dextransucrases) has been shown to be most often specifically induced by sucrose, many other LAB express glucansucrases independently of sucrose (Kralj et al., 2004; Arsköld et al., 2007; Bounaix et al., 2010; Gänzle and Follador, 2012; Harutoshi, 2013; Nácher-Vázquez et al., 2017b).

The switch of the carbon source from glucose to sucrose performed within the present study neither had an inducing nor any observable stimulating effect on the abundance of the dextransucrase Dsr1411 (Figure 2). This clearly suggested a sucrose-independent expression and agreed with previous experiments, where dextran synthesis by *L. sakei* TMW 1.411 in buffer solutions was possible even if the cells had been previously cultivated in glucose containing mMRS medium (Prechtl et al., 2018). Furthermore, it was demonstrated by gene expression analyses that the plasmid-encoded dextransucrase DsrLS of *L. sakei* MN1 was constitutively expressed and connected to replication and maintenance functions of the plasmid pMN1 (Nácher-Vázquez et al., 2017b). Since pMN1 and p-1.411_1 were shown to be more or less identical (Table 2), these results should be transferable to the expression of Dsr1411 in *L. sakei* TMW 1.411.

Apart from that, analysis of the IBAQ intensities of identified proteins suggested a surprisingly high abundance of the dextransucrase Dsr1411 in the cellular proteome, which was even comparable to those of common housekeeping proteins, such as the RNA polymerase sigma factor RpoD (Figure 3 and Table S1). The structural characterization of the dextran produced by Dsr1411 was published only recently by our group (Prechtl et al., 2018), and the results (e.g., molecular weight) were similar to those published for the dextrans synthesized by DsrLS (*L. sakei* MN1) and Gtf1624 (*L. curvatus* TMW 1.624) (Ruhmkorf et al., 2012; Nácher-Vázquez et al., 2015).

### Global proteomic response of *L. sakei* TMW 1.411 to sucrose

**Figure 5:**
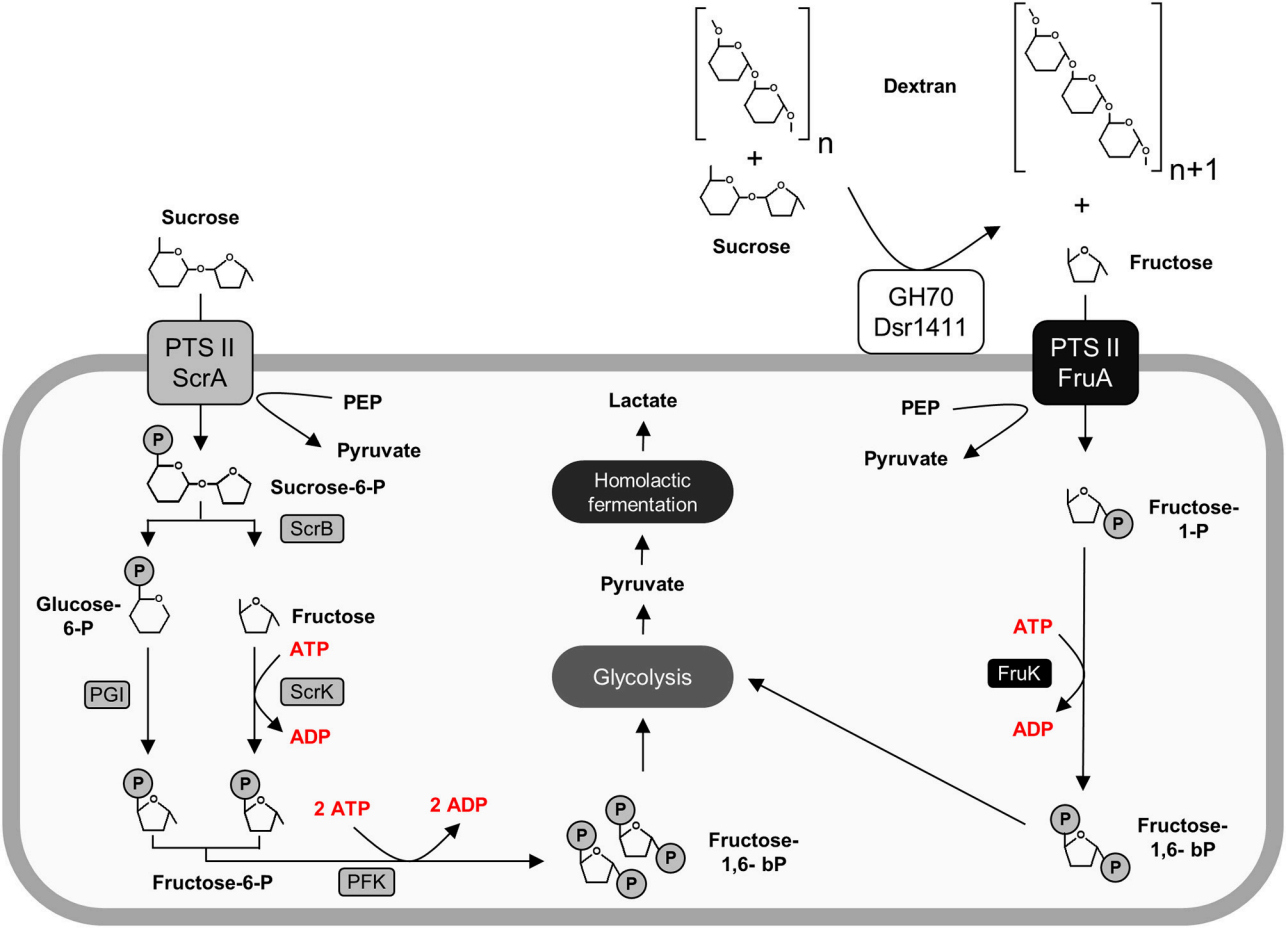
Active carbohydrate utilization pathways in *L. sakei* TMW 1.411 upon growth on sucrose as sole carbon source as suggested by the proteomic data (Figure 2) and metabolite analysis (Figure 4). Enzymes involved in the catabolism of sucrose (left) and fructose (right) are marked in gray and black, respectively. Protein names were used according to the gene names in Table 3. For clarity reasons, water molecules were omitted in the figure. Further abbreviations: Dsr1411 GH70: dextransucrase of *L. sakei* TMW 1.411, glycoside hydrolase 70 family; PEP, phosphoenolpyruvate; PTS II, phosphotransferase-system subunit II; PGI, phosphoglucose-isomerase; PFK, phosphofructokinase.

After the performed switch to sucrose as sole carbon source, the upregulation of both the sucrose and the fructose operon was detected. The upregulation of the fructose operon, which contains a fructose uptake system, indicates the active uptake of extracellular fructose at an early stage of growth. This could be explained by the simultaneous secretion of active dextransucrases (section Sucrose Independent Expression of the Dextransucrase Dsr1411), which extracellularly release fructose. The corresponding metabolic pathways are described in Figure 5.

Furthermore, the glucan-1,6-α-glucosidase DexB was upregulated in the sucrose treated cells suggesting induced dextran degradation. Although DexB of L. acidophilus NCFM (60% amino acid identity) was demonstrated to be active on dextran (Møller et al., 2012), neither of the two DexB variants contains a N-terminal signal peptide targeting its secretion into the extracellular environment according to SignalP analysis (Petersen et al., 2011). This conforms with the general assumption, that high molecular weight EPS does not primarily serve as a carbon reserve for the producer strains (Zannini et al., 2016), as active uptake of such high molecular weight polymers has not been reported to our knowledge. The upregulation of this enzyme might rather be indicative for uptake and metabolization of short-chain isomaltooligosaccharides (IMO), which could be produced by dextransucrases in addition to high molecular weight dextran. However, their possible import mechanism remains unclear, while it was reported that fructooligosaccharides (FOS) are efficiently imported by the PTS sucrose transport system in *L. plantarum* (Saulnier et al., 2007).

Apart from the enzymes accounting for the intracellular utilization of sucrose and fructose, three further enzymes were upregulated upon growth on sucrose, which are involved in the catabolism of deoxyribose-nucleosides (Figure 2, orange). This pathway includes three major steps: (i) release of 2-deoxyribose-1-phosphate from purine/pyrimidine-deoxynucleosides by the corresponding phosphorylases (EC 2.4.2.1/2.4.2.2); (ii) interconversion of 2-deoxyribose-1-phosphate and 2-deoxyribose-5-phosphate by phosphopentomutase (EC 5.4.2.7); (iii) formation of acetaldehyde and the glycolysis intermediate glyceraldehyde-3-phosphate by deoxyribose-phosphate aldolase (EC 4.1.2.4) (Tozzi et al., 2006). All enzymes of this pathway were significantly upregulated after sucrose treatment (Figure 2), except for the phosphopentomutase (gene locus DT321_08540), whose upregulation (log_2_ FC = 1.2) was only significant at less stringent *t*-Test criteria (FDR ≤ 0.05; Table S3). Interestingly, the same enzymes where shown to be upregulated in some *L. sakei* strains after a switch of the carbon source from glucose to ribose by a transcriptomic approach (McLeod et al., 2011). As the upregulation of these proteins can currently not be related to sucrose metabolism, their differential expression could be interpreted as general response to the change of the carbon source, e.g., to maintain glycolytic reactions during starvation until the organism has adapted to the new carbon source. However, further experimental analyses are necessary to confirm this hypothesis, since other factors such as glucose mediated carbon catabolite repression (CCR) might play a role as well.

Upon growth on glucose only three proteins were detected, which were more abundant in the presence of glucose (and thus downregulated after sucrose treatment). These proteins belonged to the arginine-deiminase (ADI) pathway (Figure 2, red), which involves three enzymes being encoded in the *arc* operon, namely (i) arginine deiminase (arcA, EC 3.5.3.6), (ii) ornithine carbamoyltransferase (arcB, EC 2.1.3.3) and (iii) carbamate kinase (arcC, EC 2.7.2.2). This pathway enables the synthesis of ATP from arginine upon formation of NH_3_, CO_2_ and ornithine, and was therefore supposed to provide a metabolic advantage in nutrient-poor, meat-based environments (Rimaux et al., 2011). Apart from that, several other physiological functions of the ADI pathway have been discussed, including *de novo* pyrimidine synthesis and the cytoplasmic alkalization by NH_3_ as protection against acid stress (Arena et al., 1999; Rimaux et al., 2011).

Basically, the *arc* operon has been shown to be subjected to CcpA/HPr mediated carbon catabolite repression (CCR), which is initiated at high concentrations of ATP and fructose-1,6-bisphosphate (FBP) in the presence of a preferred carbon source (Montel and Champomier, 1987; Deutscher et al., 1995; Fernández and Zúñiga, 2006; Görke and Stülke, 2008; Landmann et al., 2011). The mechanism involves regulatory *cre* sites in promotor regions, which are targeted by the CcpA/HPr complex, and both *cre* sites identified upstream of the *arcA* gene in *L. sakei* 23K (Zúñiga et al., 1998) are present in *L. sakei* TMW 1.411 as well (positions −124 and −44 from the start codon of *arcA*, gene locus DT321_05025). However, the ADI pathway should be downregulated in the glucose treated cultures, if glucose were the preferred carbon source of *L. sakei* TMW 1.411 for energy generation and concomitant lactate production.

With respect to the alkalizing function of the ADI pathway, the pH values measured prior to protein isolation indeed suggested a slightly stronger acidification by the cells incubated in glucose containing mMRS medium (section Comparison of the Proteomic States Associated With Growth in Glucose and Sucrose), which might be explained by the lack of a metabolic switch to sucrose utilization. Although the difference in the pH values of both batches was only small (0.07 pH units), it was statistically significant (*p* = 0.01) and might point to an increased lactate formation from glucose. Hence, it is possible that the ADI pathway was upregulated in the glucose treated cells to compensate for a faster lactate formation in the presence of this carbohydrate. However, this hypothesis cannot be proven by the available data and has to be examined in future experiments, as it is beyond the scope of the present work.

### Carbohydrate utilization of *L. sakei* TMW 1.411 upon simultaneous dextran formation

Evaluation of the proteomic data had revealed the upregulation of the fructose operon after 2 h of sucrose exposure, which suggested a utilization of this carbon source already at an early stage (section Global Proteomic Response of *L. sakei* TMW 1.411 to Sucrose). Monitoring of the fructose concentration during growth on sucrose containing mMRS medium confirmed this finding, since fructose was detectable in the supernatant only after 9 h, whereas dextran formation and thus release of fructose had already started after 6 h of cultivation Figure 4. If no fructose utilization had occurred, its concentration curve would have been expected to overlap with the dextran curve due to the stoichiometry of dextran synthesis. However, the concentrations of fructose were lower than the theoretically released amounts at any time during the cultivation. Furthermore, fructose seemed to be the only utilized carbohydrate after 10 h. Its depletion was observed after 24 h, suggesting its preferential use by *L. sakei* TMW 1.411 compared to sucrose, whose concentration was stagnating within this time period. Since the utilization of fructose requires less enzymes to be translated for energy generation than the utilization of sucrose (Figure 5), a preferential metabolization of fructose could indeed be energetically beneficial. As a consequence, the constitutive production and secretion of dextransucrases might provide another advantage for a life in a sucrose-dominated environment, as it not only facilitates biofilm formation, but simultaneously provides a favorable carbon source.

The induction of the sucrose operon (as suggested by the proteomic data) indicates that *L. sakei* TMW 1.411 can metabolize this carbon source intracellularly. However, solely the slight decrease of the sucrose concentration between 24 and 48 h points toward an active utilization of sucrose after fructose depletion, as no significant increases in dextran amounts could be detected. It is thus difficult to infer the amount of intracellularly metabolized sucrose in the exponential or early stationary growth phase. Although the calculated dextran yield was only 60% compared to the consumed amount of sucrose, this might have been the result of the intrinsic hydrolysis activity of dextransucrases as well (van Hijum et al., 2006; Leemhuis et al., 2013). However, glucose was never detected throughout the cultivation, which was also observed for the dextran forming strain *L. sakei* MN1 upon growth on sucrose (Nácher-Vázquez et al., 2017a). Yet, it is most likely that any released glucose was immediately taken up into the cytoplasm and subsequently metabolized. Thus, more detailed experiments will be needed to comprehensively resolve the fine-regulated sucrose utilization of *L. sakei* TMW 1.411 during dextran formation, whereas transcriptomic analyses might help to resolve complex up-/downregulation events of the operons throughout the cultivation.

## Author contributions

RP performed and planned the main experimental work presented in this manuscript and wrote the main text of the manuscript. DJ was involved in some experimental work. JB, CL, and BK were involved in conducting and evaluating the proteomic experiments. RV was involved in planning the experimental setup and writing the manuscript. FJ was involved in planning the experimental setup and in writing the manuscript.

### Conflict of interest statement

The authors declare that the research was conducted in the absence of any commercial or financial relationships that could be construed as a potential conflict of interest.

## Supplementary material

Figure S1 Overview of the experimental steps for the analysis of sucrose-induced changes in the proteomic profile of *L. sakei* TMW 1.411. This figure is partly based on Figure 1 of Schott et al. (2017).

Table S1 Proteome tables and IBAQ values.

Table S2 Operon and dextransucrase conservation.

Table S3 *t*-Test evaluation.

Table S4 RAST annotated ORFs of the *L. sakei* TMW 1.411 genome.
