# Supplementary material for: Sucrose-Induced Proteomic Response and Carbohydrate Utilization of Lactobacillus sakei TMW 1.411 During Dextran Formation
Source: Front Microbiol. 2018 Nov 23;9:2796. doi: 10.3389/fmicb.2018.02796 (PMC6265474; doi:10.3389/fmicb.2018.02796)
Supplement: Figure S1 — Overview of the experimental steps for the analysis of sucrose-induced changes in the proteomic profile of L. sakei TMW 1.411. This figure is partly based on Figure 1 of Schott et al. (2017). [file Presentation_1.PPTX]

## Slide 1
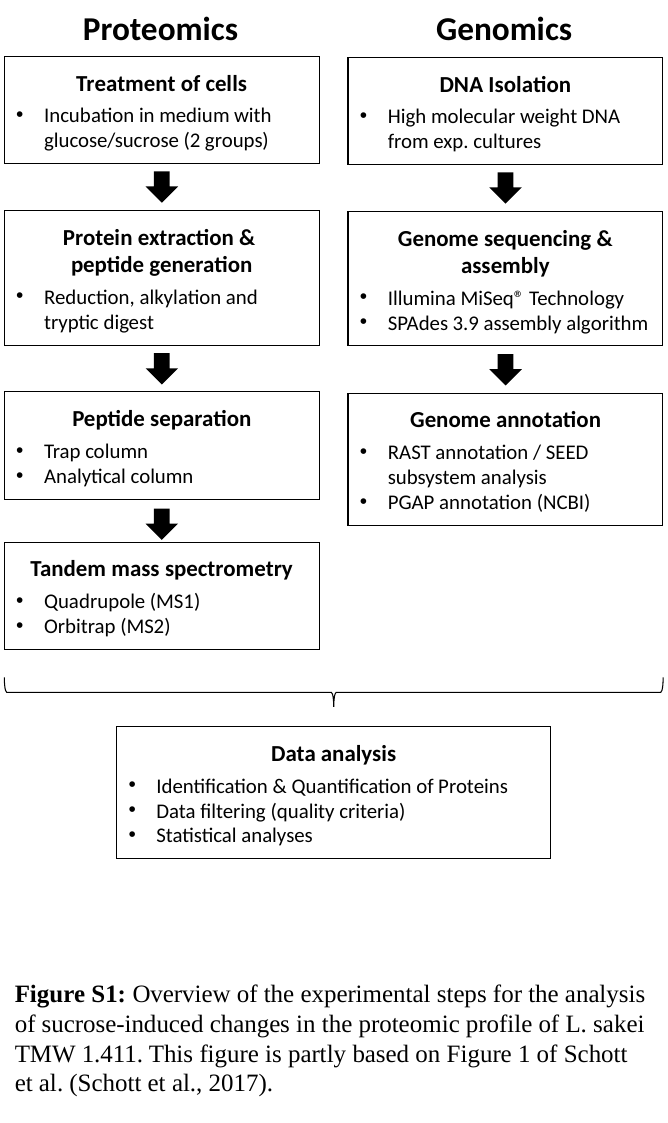

Proteomics
Genomics
Treatment of cells
Incubation in medium with glucose/sucrose (2 groups)
Protein extraction &
peptide generation
Reduction, alkylation and tryptic digest
Peptide separation
Trap column
Analytical column
Tandem mass spectrometry
Quadrupole (MS1)
Orbitrap (MS2)
DNA Isolation
High molecular weight DNA from exp. cultures
Genome sequencing & assembly
Illumina MiSeq® Technology
SPAdes 3.9 assembly algorithm
Genome annotation
RAST annotation / SEED subsystem analysis
PGAP annotation (NCBI)
Data analysis
Identification & Quantification of Proteins
Data filtering (quality criteria)
Statistical analyses
Figure S1: Overview of the experimental steps for the analysis of sucrose-induced changes in the proteomic profile of L. sakei TMW 1.411. This figure is partly based on Figure 1 of Schott et al. (Schott et al., 2017).
