# Supplementary material for: Sucrose-Induced Proteomic Response and Carbohydrate Utilization of Lactobacillus sakei TMW 1.411 During Dextran Formation
Source: Front Microbiol. 2018 Nov 23;9:2796. doi: 10.3389/fmicb.2018.02796 (PMC6265474; doi:10.3389/fmicb.2018.02796)
Supplement: Table S4 — RAST annotated ORFs of the L. sakei TMW 1.411 genome. [file Table_4.DOCX]

>fig|1664.9.peg.1

MTQLKLVALKKLLADRWLNYYEATYQNPKQEEINYHFVSREANLTTDNLKHPVTASAVTMFCLNHDHSQVLLLRQFRLPLNDYVYGNSAGLIEPGETVEQAIERELVEETGYQGVHIDQIFSPSYSTPGITDGAITLAICTIDQHPSQETALESAEELTSIWVDKKRAAEILANGKISGRTQLLLWQWVNDRLN

>fig|1664.9.peg.2

MEEKHEKTFFGQPHGLSTLFFTEMWERFSYYGMRAILLFYMYFALDKGGLGFDKATAMSVMAIYGSMVYLASVVGGWLSDRIWGSFKTVFIGGFFIMLGHIVLAVPMGATALFISIGLIVVGTGLLKPNVSEMVGGLYSENDRRRDAGFSMFVFGINLGSFVAPWIVGTIGQQVNFHLGFSLAAIGMFFGLIQYYRGRSQFSKENAKPNDPIAADELRGIIIKVIIGLVVIALGITALQLTNNLNIDNIVLIISIVAIALPIIYFYNMLSSKKITKKERSRVLAYIPLFIAAAVFWGIEESGSVVLALFAEDRTILHIGGWHLAASNFQSLNPLFIMLLTPLFVWLWSRWKKQPSAPGKFAVGLIFAGLSYMFMALPGLLHGTTGRVSPFWLIGSWLIVEIAEMLISPIGLSITTKLAPKAFRSQMMSMWFLADAAGQAVNSQLVRLYSPQTEIKYFLGIGIVSAIFGIVLIFFVKPIHKLMEGVD

>fig|1664.9.peg.3

MGVQKYRHDQLTQNIARARTVKVRPVQTKKTADDNAPQPTYWDEDTRGITVGKLAIPSVGIKLPIIKGIGQTNGHDNMLKAAVTNKQGQTMGKRNYVLSSHDIAQENVLFSPLGQTKVGAAIYLTDTKQVYTYRVISRKTVKANQVAILDDVRQKRLVTLYTCDPDGEIVNGQTYERTVVVGELVQTTKASAKTLTPF

>fig|1664.9.peg.4

MKIGFIGVGNMAQAIIKGLVKADQIATTDILVHGAHPANYEAFAAEQHITPVADNIAVVAQADIVFLAVKPYIVPLILEETSAAFKEKQPLMVSMAAGLSLAKLSQQVDADNLPILRIMPNVNVANRAGITAVVGNAQVSADQLKSVKDVLSQLGGVVEIGEKDFTTFSALAGSSPAFVYLFIDSMARAGVKHGLTKKAATEIAAQAVLGSAQNVLLSDDSPWDLIDKVSSPGGTTVAGLLAMEEAGLMSAVVKGIDATIEKDLESQK

>fig|1664.9.peg.6

MTIKVLKHAVIYTGDDVIKDGYIRFDKQILAVGPMFDYKAQAGEEIQDMKGQTIIPGFIDVHCHGGYGFDAMDGDANQIDEMATKMMQNEGVTTLFATTMTQSNENIANAMRGVKDAAEKNPLIQGVHLEGPFIAPIFKGAQPEQFIKDPDVDLLDEWNKLSGNRVRLITYAPEDPGSKAFEDYCLAHNIVPSVGHSNATREQMIESKATHVTHLYNAQREFKHREPGVTGHAMLEDNMYAELIADGFHIVPDMIKLAYEQKGAERIDLVTDSMRAKGIAEGESELGGQKVIVKDKQARLEDGTLAGSVLTYIDAFKNIQTFTGCDKFEAVMMSSVNQAEEFGLTQKGKLEVGRDADLNILDQQQNLVATYSFGQGYQH

>fig|1664.9.peg.7

MESPVYIQIHNQLKKDIEAGKWSIGDRIPSERELALNFEVSRMTLRQAVQTLVDEGILERRIGAGTYVANRKVQEKMSGVTSFTDLMTAQGKTPSSKTISFHIANPSLSEAEKLQLKDGEQVLRMERIRYADNIPICFEVATVPQSLVADFSKAEITSSFYKTLEEKGGYQMGGAQQTVSAQLASERIAEYLDIKRNGSILRLRQVSFLNDGTPFEYVRTQYVGERFEFYLER

>fig|1664.9.peg.8

MDIYQWENVFQSKALDKAFTRFMVRRHIRKLWYVGFYHFYLSLLLVLRIQKKERYFQNYWRFLDKVHITSDELDRFKNRFKVKNLILLSDSRQKAIVGQIPEFLMSLFIPREVIDSGRVKIDGLAVDQEGKWTPTSAVSKQDSTGKTYFGDLSFIFKNGKFKNKYVVFNNGIAFKTVTNMLLYWFVQMMLVVGISIFIGMASVIVGGYKYSFPMVKDVLMDSHLLMLNCLAPILMGLTLYFLLGRIWLGTGLNALIFVGLGIANHYKLMFRDDAVQFADLQLVSEASKMSQKYAIQLTWKHVVLVLLFIAVLIMLRNYFKPVMKFQFRIVGLFLVLLLTFTWGKSELFNPDTYGKAAYVKYGNIWKATSQYMTRGFPYAFAHSIESTGVSKPEGYNKAEAKALLDQYKSQNIPDKKKVNVVMIMMEAYNDFSKYPQLEIDPSVYAGLKNVEKESISGHLVTNIFAGGTIDTERKTVTGYSKLQNFNHNTDSFAWYFKNQGYQTQALHPMYGWFYNRRNVDPVLGISDFKYMENYYNKYNQFVVPDKTFFKSIHSFSEKSKKPYFNLSVTYQNHGPYANKYTGTPLLKWQDGYNQADYAIINNYLAGINKTSEAIEELTNSFKNDKPTVIMFYGDHNPWLGEQNSVYKMLNIDLNLGTAQGYQNYYQTPYVMWANKGAKKVLKNDFVGQGKDFSPMYLLPEFFKQADLKGDSYMQYLQNTENKISVIGENKYKVGNKYVKSLTGKDKKTYDNLKKVEYYRLHEKVSD

>fig|1664.9.peg.18

MTAIATVKAVLYHRVWFGLIKLVTPMSDETFFINSFFGKSFSGNPRALFEGLIEKFPNSDYIIVLNDEQARQTVKAQYPDINIQFVARHEKAYLKALARAKYWIMDINFPFRLKPHKNGVFVQTWHGTPLKHIGNDLPDDNDFKRLTAREPLNWDYFVSNAPEDNWLYERAFNLKQTKIMSYGLPRNDYLAKHKDDRELVASLKAKLQLDATRKTILYAPTFRDDEPTFKLALDLEQFEAQLGDQYDLLIRLHPNVADQMPDIAAYPHVHNVNRYASIEELYLAADVLMTDYSSVFFDYALLEKPIVFYAYDLDKYQDILRGFYFDYQAFIPGPLVTDNQALYTLLSQGLTAQTTKAFNQLHNANTDGSATQKIIQEVWKV

>fig|1664.9.peg.20

MTKVLTYGTFDLLHWGHVHLLERASQLGDELIVGLSTDEFNAEKHKEAYHSYEHRKYILEAIRYVDKVIPEKDWEQKIKDVQKYDIDVFVMGDDWKGKFDFLKDYCEVIYLPRTTGISTTQIKQDLEN

>fig|1664.9.peg.21

MKVLFVNAGNETGGGRSHIIGLMKAMIALGKDEPSLLVFEDGPVAELARANQLPVEVFEQPKTLTPHFLKQLAAHINSENIDVVHTHGPRSNLFLALIRKRITAKWTLTLHTLPKIDYLNKGFKGKILLPLSLWVLKKADHIYLIAERFRNSLVQQGIDTNKMTTIFNAIEFSPEVPTPVKQPQFTMICVARLTAQKHQQLLLEALANVDFEYQLHLIGDGELEAEFKKLATDYQITDKVHFDGFQSDVAYFYRHTDLSVLSSIHEGFPTVLLESGNYGVPAITTDVGDSKVIVDSPEYGWVMDSLDLQGYVKALNEAYAAYQNNQLVGMGLAFNKHVSQSFSTRQLAILMHDLYAQL

>fig|1664.9.peg.23

MQFPTVSVLNIPFINTTNQAFIAQLSKDLAANENRFIVTANPEIALYATKHNDYFQTISQADYITPDGIGILKGATMLGTPLTERITGFDTMTALFEIANQNSYSVYLLGAKEEVLQAACQNVAKQYPNVTIVGQHNGYFDDQEEQKIVADIERTQPNIILAALGFPKQETFIEANRHKVNAIWMGVGGSFDVLSGTVSRAPKIWQKMHLEWFYRFASHPSRLNRFPALIEYMRLVKKEKKN

>fig|1664.9.peg.24

MQKLYPDDSLLLHTDLYQLNMMLTYFKKGLHNRTAVFECYFRKLPFENGYAIFAGLEHIVNYLENLRFTESDLAYLRDEVGYPEDFLTYLAELDFDLTVRSAVEGELVFGNEPIMQVEGPLAQCQLIETALLNIVNYQTLIATKAARIKSVVGKDPVLEFGTRRAQEMDAALWGTRAAYIGGFDATSNVRASKIFGIPASGTHAHSLVQAYRNDYEAFRAYAETHRDCVFLVDTYDTLKSGVPSAIRVAREFGSRINFQGVRIDSGDMAYISKRVRQQLDEAGFTDAKIYASNDLDEKTIQNLKMQEAKIDVWGVGTKLITAYDQPALGAVYKLVSIENSDGKMVDTIKLSSNAEKVSTPGRKQVWRITKQVDGKSEGDYITLWDEDPLEQDTIYMFHPNYTYINKTVTDFNARPILRTIFENGKRVYTLPELAAIKKFCAENLDSLWDEYKRDLNPQDYPVDLSQRTWDNKMSSIRKVREDVNLMH

>fig|1664.9.peg.25

MNALQAEIIAALKTQPTIDPAKEIRRSVDFMKAYLKKNTFLKSYVLGISGGQDSTLVGALAEKAMQEMRAETGDESYQFIAVRLPYGEQADESDAMAAIEFMAADQVKRVNIKGSVDAMVQSLAETGVIVSDFNKGNIKARVRMIAQYGVAGENSGAVLGTDHSAESITGFYTKFGDGGADLVPIFRLNKRQGKAMLAELGAPKHLYEKVPTADLEEDRPALPDELALGVTYDQIDDYLEGRQVSEEAATKIENWYKKTAHKRHLPITIYDTFWQ

>fig|1664.9.peg.26

MVDKQTTFFTQSTDATLKALATTPQGLTQEQVEKRLAENGRNQLTEKKRKSLAIRFLEQFKDFMIIVLLVAAMIAGFLAHEWPDAFIILAVVILNAIFGVFQEAKAEQAINALKEMATPDAHVRRDGEIVTIKSEELVVGDIVLLEAGDIVPADLRLLESAALKIEESALTGESVPVDKTVDTLTDPESGIGDRTNMAFMNSNVTYGRGVGVVTGTGMQTEVGKIAGMLNQADETTTPLQMNLSQLGKSLTIMILIIAAIVFVVGYLRHAQSPINMLLTAISLAVAAIPEGLPAIVTIILALGTQKMVKRKAIVRKLPAVETLGSTDIIASDKTGTLTMNQMTVEKIFTNNQLQDAGATLPADNLTLKIMNLANDTKIKADGEMIGDPTETALIAYGRDKDFDINVALAAEPRVAEVPFDSERKLMTTIHQQADGQFLMTTKGAPDELLKRVTHYIDAQGVVQPMDDAERQLLLDTNHGLAKQALRVLALAYKVLPSVPATVDPTIETDLIFAGLVGMIDPERPEAKQAVTDAHSAGIRTLMITGDHRDTAEAIALRLGIITPEEDADAAVITGAELDQMDDQEFAKKVTQYAVYARVAPEHKVRIVKAWQKHGKVVAMTGDGVNDAPALKSADIGIGMGITGTEVSKGASDMVLADDNFSTIVIAVKEGRKVFANIQKSIQYLLSANLGEVLTLFMMTVLGWQILQPVHILWINLVTDTFPAIALGVEPTEPGIMNQKPRGRKSNFFSGGVFQAIIYQGLLEGGITLAVYALAITFPVHQASGLAHADALTMAYATLGMIQLFHAFNVKSVHQSIFTVGFFKNKAFNWAVLASFILLAATIMIPGLNGLFHVSHLDLYQWGIVLAASFMLVVIVEIVKFFQRRAAK

>fig|1664.9.peg.27

MNKTQQKLAKYGQAVNDLIDNTEKVQEGMSKYFVPLKAAVADGKLAEMSEQDYQKTAQIFRDGVDQYHEFLAQFKTAVAPARLMGNNQRLIGAYTDFVQGCEDLITSMHDDQTVDMQAFAASEMNQDKATERISKYIQNISKIA

>fig|1664.9.peg.28

MTDIQQLVTKQLTQYRPQQINAVLTLLDEGNTVPFVARYRKERTGSLDEVQIREIEHTFQYLTTLEKRKTDIIKNIAEQAKLTPVLQKQIEAASQLQVLEDLYLPYKQKRRTKATIAKEAGLEPFANLIAQLPNFSEQVLLEKAADYLNPDQKIIDTDAVLAGVHEIIAENISETATFRDWTRQYTRQNGQIVSQVKDADSDDKQVYQIYYDFSAPINKIAEHQYLAINRGEHEGILKVKLEVAENMIVGHIAQAVIHGQDSAAAQIIKTAIEDAYKRFIGPAIERELRKQLKEKADQQAIAVFGDNLKHLLLQPPLKGKVVLGFDPAYRTGCKLAVVDGTGKFLDKLVIYPHKPAAAPKRAAAADEFKQFLEKYNVEMIAIGNGTASRESEEFVASVLKTISREIFYVIVNEAGASVYSASDTARAEFPDLHVEERSAISIARRLQDPLAELIKIDPQAVGVGQYQHDVAQKELTTQLDVVIEDVVNSVGVNLNTASPQLLQHISGLSATTAQNIVQYRNENGLYQKRSELKKVPRLGLKAFEQAVGFLRIIGGDNPLDNTDIHPESYPVAKAILKSLGVTLEALGTPELSAAIKQLTVTDLATELAVGSETLEDILSGLQKPGRDLRDQMTAPLLRHDVLTLADLKPNMALEGTVRNVIDFGAFVDIGVKHDGLVHISQLANHFVKHPSDEVSVGDVVQVWVLSVDQERERVQLTMKAPKATD

>fig|1664.9.peg.29

MMTDQELTQLIQNISVTTFNRPFSHQANFNRRLKTTGGRYHLQDHHIDINPLMVAQGQAVLEGIIKHELCHYHLHLLHRGYRHRDHDFKRLLQQVGGARFAPPVTAKKAPQKRLAYQCTKCGQLYQRQRHINTTRYVCRRCHGHLKLVKEI

>fig|1664.9.peg.31

MTRNSSAYKISILAILTALLMIQSFVPMVGYIYILPGLPGVTTMHLTVIIGAIILGTRGGATLGAIWGTLSLIHAYTAPVDALTLLIFQNPVIAILPRLMVGLVAGLLYQHLHNTKAGQRGSMAIAASFGTIVNTTLVILLTWAFYSQQAAGIYKTDVSHLIIVMLSAVAINALMEIALAVIVTPFVAKPLLRFKRE

>fig|1664.9.peg.32

MLKILKRLLPILGLLFLLAGCEKPLDTKQQVKDDLNTILQISKTQQTALSTLEDADQGVPDSYLKIQAKYPDQNLLDVKGSKIAQTAASRAEAYKTLKIQQAELKPVVKRLIKIANQESDELPIEFIKDLNQSLKLSALDYTTLSNFYDAAATAETDFYADHTSADAHADDLAAPINRLNQYYSAIYQQAEIAKVNINTVTAQAKKVQKQLD

>fig|1664.9.peg.33

MENGTVKWFNAEKGYGFVTREDGSDVFVHFSAIQGEGYKTLEEGQSVSFDIEESDRGPQAVNVNKN

>fig|1664.9.peg.36

MAKKKKRRTTKKWYNTKKRQPTEWLVVLLGVLFALGVGLVYIQYTGAQTQERLANEAKQEELAKVKKKKEFINQLAPYAQELQQKYHVLSSVTLAQAILESDWGKSSLAADYHNYFGIKGDDPDNTKEMTTKEYLNGQWVTTTARFRVYRDYRESMLDHVLLFAHGTSWDHNHYQHVVAATNYTQAAQALKQDGYATDPNYPEKLIELVKTYRLDQFD

>fig|1664.9.peg.37

MRLLEERIKKDGQVLGEDVLKVDNFLNHQVDPELMAAMGEEFKRLFEGAPITKILTVESSGIAPAVFSGLAFHVPVVFARKHKSLTLQDNMYSATVYSYTKKVNNHISISKKFLNENDRVLVIDDFLANGQAVEGLLEIIDQAGAQLEGVGIVIEKTFQKGRELLDQRGIHVESLARIAAFEQGEVVFLDENNEN

>fig|1664.9.peg.38

MPVIYPGQTIGIIGNDQRAYKLALKAKEMGYRVAAIVTEQTQDSSLAAVLDIQIEGDPQQYETLARLGDCCQVLTYTDESLDETLLEQLVQDYNVPQGSDALSMTQDRYLEKVFLSDLNINISPYVTVVDKTDIEQGVDSIGYPCILKPIQRGFGRQYHQYLESPADLAKMDDTLELGAYLLESWVPKQQELAVMVAKDYEGQVVTLPIIENQMQDQDLQLSITPARIDQDVAEEIERLATVIGQNLDYMGIFGIEFFLTADMNIYVKRIVTAPHETGDVLNRILNFSQYEYHLKAICQLAIGPSRLNNAGVCLRTTPEQWPLIQTQMKIKPDWYFNRCQTIGAQESGYITAVGHNIQPLLDNFEAAELI

>fig|1664.9.peg.39

MLERYTRPEMGAIWTTENRYQAWLEVEILAVEAWAKLGDIPAEDAAKVRANASFDVEEINRLEAITHHDVVAFTRAVSESLGAEKKWVHYGLTSTDVVDTAQGYILKQANDLIRADIERFMAVLKQKAYEYQNTVMIGRTHGVHAEPTTFGLKLATWYAEMKRNQERFERAAAGVEAGKISGAVGTFANISPEIEAYVTENLGIRAQDISTQVLPRDLHADYINTMAVVATSIEHFATEIRHLQRTEVREVEEFFAKGQKGSSAMPHKRNPISSENVSGLARVIRGHVITGMEDVSLWHERDISHSSAERIILPDTTILLDYILNRFTRIIEKLTVFPEKMKADMNLTHGLIYSQRVLLKLVDSGLSREAAYDLVQPKTALAWDEQKDFRQLLEADPKVMAQLTPAEMDDAFDYHWHLSQVQTIFDRVFK

>fig|1664.9.peg.43

MSEQELLKGMNPEQAEAVLTTEGPLLIMAGAGSGKTRVLTHRVAYLIEEKHVLPWRILAITFTNKAAREMRERVGNLLGEGAQDVWVSTFHALCVRILRREIEQIGYNRAFTIADPSEQLTLMRHVCRDLNIDTKKYEPKAILGTISKAKNALQTPADYQGLANGPFEKMAANCYTEYQKRLHQNQALDFDDLIMQTIRLFRENKETLAHYQEKFQYIHVDEYQDTNEAQYTLVNMLAEKYQNLCVVGDADQSIYGWRGADISNIMNFEHDYPDAKSVMLEQNYRSTKTILEAANGVINRNTFRKPKELWTQNEEGQPITYYRGQSERDESLFVITKIQEEMQKNNRKYGDFAVLYRTNAQSRTIEEAFVKANMPYKMVGGHKFYDRKEIKDILAYLRLVVNAADSMSFERIVNSPKRGIGPGTLEKLGQFADDHQWSLLEAAQNAELSTIPARSRNTLIDFGNVIGDLAKMREFLNVTDLTEQLLDKTGYKKALEVENTLESQTRLENIEELLSVTKQFDEHYEPEEEDSDLFVDFLAELSLVSDLDSVEEEASEVTMMTLHAAKGLEFPVVFLMGMEEGIFPLSRALLEEDELEEERRLAYVGITRAESKLYLTNAYSRMLYGRQQSNQASRFVGEIDDKLLDYANGNAGSIPERKLPFGKSNSYARATATTYNGPKTTTSHKIADTTGGDKSGSWQAGQKVQHKKWGIGTVVKVNGTGGDQELDIAFKEQGVKRLLASFAPIEKVE

>fig|1664.9.peg.44

MGLAKPVENYTQSEAASLAQQLRDQLADYSQAYYTQDAPLVEDHVYDELYRDLEQLEAAFPVIVTNDSPTQKVGGQVLPGFTKVTHEIPMLSMGDVFSEAELAAFDQRLRKNTATEIEYNVELKIDGLAIDLIYEEGRFVRGATRGNGTIGEDITQNLKTIKAIPQKLTRPVSIEVRGECYMPKAAFADLNQKREAEGQEPFANPRNAAAGSLRQLDAKVAAARQLSAFIYTIVTFDDVQATTQSEALHVLAELGFNVNPTSVVCQNMAEVQAFIEQYQGTRNDLAYGIDGVVLKVNDLALQRQLGNTVKVPRWEIAYKFPPEEAETVVREIEWTVGRTGVVTPTAVMDPVQLAGTTVSRASLHNADMLRDKDVRLLDTVLLHKAGDIIPEISQVVLDKRPSDSQVYAVPTTCPSCGHDLVHLDEEVALRCINPMCPAQMKEQLTHFASRNAMNIDGLGPRIITQLLAKELIHDVADLYRLTADDLAQLDKFKEKSINNLLNAIDASRQNSLERLLFGLGIRHVGAKAARLLAEHFQTLAALMASDQETIITVDTIGTIIADSLVTYFADSQVQTLMAELEAVGVNLTYTGVTKAQAATSDSYFNGKTVVLTGKLEQYTRGELKQLLEDNGAKVTGSVSKKTDALIAGQDAGSKLEKAQSLAIPVINEADLDNYLAQ

>fig|1664.9.peg.45

MAKYKIWVVAAATLLLLAGCTSLPGTSSSSSSKATKKLQTTGKVTDSMYEGVIDNGQYKTSQTRGLTTQQSNTSFDVKSLESGLLKLSKEQFPTSKYVFQEGQSLKTAQAQKWLARKSKTNTEGLNPEDNGQKDPNKRNPIYLQQILEQDYLGEDGSMKGMSIGLGMNQVDYYTKEQYGATFETKISKAMMTQQGKQMANKVLSRMRQKKGLSDTTIVIGLYKQAPKDSLVGGTFFAYGVSKKGSTTIDSWHELNQENQVLPVVNDEKAINQDDATAFNSFKTQVETFFPNLSGVTAQTHYEEGVLAGMNITVNTQFYSETEIMSFTQYIATAADKYLPRNIPLDISITSAEGMQAYVGRASGSKGFTTHVFGSY

>fig|1664.9.peg.47

MIDKKQVQHVAELSKLAFSDEQLEQFTEQLADIMKMTDELNEVDTTGVPVTTHVNGLKNIVREDVAQPGTDREILMKNAPDSADGLLKVPAIMDKEEA

>fig|1664.9.peg.48

MDYLNEDLQSVHDKLVAGDLTASQLVTDTLETIKTKEQQVDAFLTIDEKGAQEAAAKIDAQPIDADNLLAGMPIGIKDNLLTDGVTTTAASKMLANFKPVFDATVVEKLKAKKAIMIGKTNLDEFAMGSSTETSAFKKTKNPWDLDKVPGGSSGGSAAAVAAGEVVAALGTDTGGSIRQPAAFNGIVGIKPTYGRVSRWGAIAFASSLDQVGVFSRTVADNATVLQAISGHDEKDSTSADYDVPDFRAALNGDIKGMKIAVAKEYMAEGVEPAVKAQIETAIETFKSLGATVEEVSLPHSQYAVQTYYIIASSEASSNLSRFDGIRYGYRSPEAKTLEDVYVKSRSEGFGDEVKRRIMLGTFALSSGFYDAYFKKAGQMRTLIIQDFEKVFEDYDLVVGPTTPTTAFKLGDKVTDPVTMYMNDILTIPANMAGLPAMSVPAGLVDGMPVGLQIIGKAFDEESVYRAGYAFEQATEFNKNVPSFKGGQA

>fig|1664.9.peg.49

MNFETTIGLEVHIELKTNSKMYSPSPVNYGAEPNTNTNVIDWGYPGTLPTLNKGAYTLGMMVATALHADIARDTHFDRKNYFYPDNPKAYQITQYEQPLGKDGYIEVEIDGHKKQIGIAELHVEEDAGKNTHGSDGYSYVDLNRQGTALIEIVSKPDMSTPEEAYAYLETLRQIVQFTGASDVKMEEGSMRVDTNISVRPIGAKKYGVKSELKNLNSFNHVRLGLAYEIKRQSQLLIAGETIRPETRRFDEKSGETYLMRVKSGADDYRYFPEPDLPPMHIGDDWLKEVQDNMPEMPAKRRTRYVDELGLPEYDAGVLTNTKEMSDFFEAAVANGAAPKQVSNWLMGEVNAYLNDKQIDLQETALTPEHLAQMINLIKDGIISSKIAKKVFQEIIQNDTDPKAWVEAKGMVQLSDPAKLTPIITGILDDNQQSIDDFKNGKDRAVGFLVGKIMKETRGQANPKVVNDLLMAELNKR

>fig|1664.9.peg.50

MQKRARLIYNPTSGHEVMKRSVADILDILEQAGYEASAYQTTPEPASAQKEATRAAKDDFDLVVAAGGDGTINEVVNGIAGLAKRPKMAIIPAGTTNDYARALKIPRDNPVDAAKVILKNQTLKMDIGQANDNYFMNIAGGGLLTELTYEVPSDFKSIFGYLAYIVKGAEMLPQIKPIQMHLEYDEGTYDGQASLFLLGLTNSVGGFEQVAPDAQLDDGNFSLIVVKTANMAELVHLMALVLKGGKHVDDPRIIYTKTSKLVARPADENDKMMINLDGEYGGDAPMTFINLQQHIEMYVNKEAIPDHAITTETPEELAAEDQFVKEVEEISKEDIDGDGKIG

>fig|1664.9.peg.51

MKIQAPVRKNEKLTVDIMDLTYEGMGVAKVDNYPLFIEGVLPDEQAEVEVTKVNKQYGFARLVNLLKKSPDRVESKANIYSQTGIAPLQHLAYPAQLTFKRNQILNVFQKAHLDIPVQPTLGMTDPTGYRNKAQVPVRFIDGALMTGFYRKHSHQVMPMEDFYIQDPKIDEAIKVVRDILRRFYIDPYNEDTHKGVLRTIMVRRGYYSHEMMIVFITRSKKLPAAREIAIEIKKALPEVVSIMQNVNAAQTNVIFGDQTKLIAGRDFIRDELMGLKFEISAQSFYQVNPVQTEVLYQKAIEAAELTGNETVIDAYSGIGTISLAVAKHAKQVYGVEVVESAVMDARHNAKINAIDNVEFTLGKAEDVMAQWQTDGLQVDALIVDPPRKGLEPSFIEAVGQLKPAKMVYVSCNPATLARDLDLLAAQGYQAEGTQPVDMFPQTLHVESVTKLVLK

>fig|1664.9.peg.52

MKVIVQSKLLLFGQWITRVQTNHPLYQIIQRSLTLLFPFVLIGSLSQLIQLTLFNRHSFIASIFHFSTWLTKHNYMQVPFNSLTSLTLGIVAAIAAFATARYTAKYYGRDEQLASITGLVAYLLLALRYTPNGELTFNANLLGMGALFFGLLVGYLVGLVFRWLGKAQQVANPNNGSIVGRSLDSLVPISLVVVTAILVGLLLNWLEISILPDQVILKLQNLKANQTSLIFSLGIGVLVTILTFFGLTSMPTLEQFGRDGFAATVNLNYAFLHHTPWHVPYPFTLGTLYGPFGAIGGTGGTLALVIAIFLFSRQRDQHLIGRWSLMPVLFNFNSAVLTGLPVLGNGLYIIPFLLVPLVNMGIAALAIALNLMPAVVYNVPLGTPGLLQAFIGTNGNWMALVISLLNVFVGVLIYKPFIQLANRLQEGVDQDV

>fig|1664.9.peg.53

MSKLQRKSLILLGLSFLFLGAMMWPSAQWTRQNVATLTKRHDSKMSPVIFIPGSSATQNRFDELVTKLNKKRGNKHSLLKLTVDTNNHISYSGEIKPRDNEPFIVVGFENNKDGYSNIKKQAKWFSIAFTALAKKYQFNNFKAVGHSNGGLIYTAFLENYFNRDDITVKKLMTIGSPYNFEETSLAHKSQMLTDFIANRKKIPSNLSMYSIAGTENFENDGIVPARSVEAGKYIYQGQVKHYTEITVTGDQAQHSDLPQNQQIINLLEQYILSKTTNRMNQPPKAGANSDDEQEH

>fig|1664.9.peg.54

MGIFLTILLIVILVLIAITLFSSFALIHTGEVGILERLGVYVKTLEPGFHLVFPFLYHITEVVNMKQIPLKVAEQEVITKDNVVVMISETLKYHITDVNSYVYKNKDSVLSMVQDTRAQLRGIIGNMDLNDVLNGTEQINHTLFEQLSAVTAGYGLNVDRVNIDSIQVAHDIQESMNKLLRASREKEANIMEAEGLKAAAIRKAEGVKEANILEAEANKQTQILEAEGKAQSQRTVAEAVKDQINLINSSLVNNGELYLQFKNIEAMEHVADGQNNTIVLPNKAIDSLGSLPAVGELLANKDKHL

>fig|1664.9.peg.55

MLDERLLGLTFQLPDAQSAGVISFFGVLYHYRVAGPAETGLLVVVTDVSPLYLTVTAQHNVLEY

>fig|1664.9.peg.56

MKRRMTPIFQDSFQKRYWRRIVLLSLLAIAVITGIWWWLQNRTDRPNEQQYPVLGAQISQEDGYQDYQVLKKEGLKFVYLKATERASYKDDNFDSNYSRAEGSGISVGVYHFFSFDSSPEDQAQQFIKSVGQDTGHLPIMVYLSYYNDYAQKPPAKAKTQRAIARFVTLINQYYHQDCIIGGAPALLKRYVPTKGTYPLWQTTQQRPTVGTKNGFWQYTTASKIPDGRDDSEYQLAVFTGTPQQWQKLK

>fig|1664.9.peg.57

MFKRKGWLLLIIIVIFFIGGSWGIKEYYYGGETYYTQITTDGTKQVDKDTEGKSYVSYQYDLPAYDQAGHQKKVSFNSAKSSRLQKQAYLELKVNDRKGVMSWQKVDKADLPKAAEKALAKQLNR

>fig|1664.9.peg.58

MRIFEFKKPRELFDQTIHQYQQKARSTQASKTATFYAGHWTFDDEKTHKRHTLEITPNLLIQIDHKPLTGKVILLSETELIFLDHFGYQLKITCENHQPVSIYDEAEDTEYPIK

>fig|1664.9.peg.59

MQKLGIIGTNWITGQFVNAAREAEAFELTAVYSRTEEKAEQFKTDYTAPEANTYTTLTGFFGSRDFETVYIASPNSLHFKQAKQAIEAGKNVIVEKPAFSNPDEMADMIALLADHPDQFLFEAARQIHEANFKIVQDAIAKLPSINGATLSYMKYSSRYDAVLAGEEPNIFSPHFSGGALQDLGVYVVYDALSWFGQPITVDYQAEMLPTKVDGKGVATLHYPDYDVTLIFGKTVDSYLTSEIYGGQSTIVLDNAGTIGQVDLYEDGGQKITSLGEQSLANPMMAEAATFATIIANHDTKRYHELLQLSQSVNAVLYQLRQSAGIQFDADQH

>fig|1664.9.peg.60

MSVSPLFQALWDEQGFKDLSPIQEAVYQPLKQDKSILGLAPTGSGKTLAFSLPLLEKIMPGEGLQVLILAPSQELVIQTRDVIQPYAKAIDCNVQAITGKANVKRQIERLKTKPEIIVATTGRLLELSEQRKIKFHTLQAIIMDEADELLTDSGLSETRHIVQESPADVQLGFFSATSTDTLTDLETWFGVPIETIDVRSIDKTGGDVLHAFIPTGNAQKVNVLRHLAHQKNFRGLVIFNQSAQLLQAAQTLKHQKVTYAALSGQGRQTERQKALKDFREGKIQLLLATDVAGRGLDITDLPYVINFDVPNAKITYIHRAGRTGRMGREGTVLTLGNQHDGRNLRKLLAPEYDLVTRYLVGDHLEETKPAVVKEAAPVQKTATTPTPQPTKTPVADDRPMGERSKAKVAKKPLPTRQPAEPLKPKRKKNRKRDQKNKGYHKKNKDLTV

>fig|1664.9.peg.61

MADEQNLETIMGLIINGGNAKSSAMEAIQAAKKGDFDQATAKLQESDAALSEAHNVQTGMLTKEASGDHIDVTLLTVHSQDHLMNAITFRDLAGEVVDVYRKMAGLPVETY

>fig|1664.9.peg.63

MEYQQLAPFPQDFLWGSASAAYQIEGAYQEAGKGQSVWDQFVRIPGKTFKATNGDVAVDHYHRYQEDVALMARAGLKAYRFSISWPRVLPTGRGTVNEEGLAFYDRLIDELIANQIEPIVTIYHWDLPQALQDEYGGWESRQIIPDFTAYAALLFERFGDRVRHWVTLNEQNIFITHGYLTAEHPPAVMDAKRTYQANHMANLANASVIKLFHEKGYQGGNGPSFAYSPTYALDADPVNQIAADDAEQLNANLWLDIYAWGRYPKLILNYWAREGLLPDITAADQALLQDPLARPDFMGLNYYQTTTVTANPLVGGVGLTKQNTSGKKGTSQASGVPGLFKTSDNPYMQQTDWDWNIDPEGLRMALRRIESRYNLPVLITENGLGAYDRLEGAGEVHDEYRIVFLKAHIQAIQEAITDGVTVIGYTTWSFTDLLSWTNGYQKRYGFVYVDRDETDEKELNRYPKDSFYWYQRVIRSNGQQLN

>fig|1664.9.peg.64

MTTQQRRDAILAAIKDSDQPISAKQLAQTYHVSRQTIVGDIALIRAQGVNIIATVSGYYYQTTTQQITGYRAQLVCQHTQAKTRAELTAIVQNGGRVIDVTVDHQVYGEITGQLGINNLTDVKGFIQKIKKYPEQKLLSTLTGGIHLHTIECASEQDFERIKAALSALDILYVNN

>fig|1664.9.peg.65

MTKEQHKLLWAAGGAWLFDAMDVGLLSFVIAALAKEWHLNSGQVGLIGSVGSIGMAIGAVFFGALADKIGRRNSLLFSLLLFSIGNGLSALSPSLIAFMALRLLVGIGLGGELPVASTLISENTPDATRGRAVVLLESFWAAGWLAASLLAYFVIPNWGWRVVLLITSLPAIYTLVLREHVKDSHEPATIQKPLPFRQRVTKLWQPAYRQKTALLWFVWFLVVFSYYGMFLWLPSVMVLKGFSLVNSFGYVLIMTLAQLPGYFSAAWLIEKWGRKWVLGSFLIGTAISAYFFGTAQSVALLISAGALLSFFNLGAWGALYAYTPEQYPAIIRGSGSGLAAAVGRIGGIVGPFLVGYLINQKVSITVIFTIFTVAILMAAGAVLLFGVETMHQKMTDVD

>fig|1664.9.peg.66

MSLVNGTEIFKAAREGHYAVGAFNTNNLEWTRAILGAAQETNTPILIQTSMGAAKYMGGYELCKNLVEQTIKSMNITVPVIMHLDHGNYEAAKECIEVGYNSVMFDGHDLPFEENLAKTKEIVALAHAKGISVEAEVGSIGGEEDGIVGAGELADVEEAKQLAAAGPDYLAVGIGNIHGVYPENWQGLSFDRLEELAAEIKIPLVLHGGSGIPKEQILKAIDMGIAKVNVNTEQQIAWSKAVKAYYAADKDEAEKGFDPRKVLAPGTKAIKDTVESRIEWFGTPAVK

>fig|1664.9.peg.68

MAWFDLLIAGLAEVWWATTMKMSSGFTKLNYTLLTIVGMVVSFGFLIRATKGLPLSIAYPVWTGIGAVGAILVGVILFNEHLSLLTWLFVILLVIGIIGIKVTSGH

>fig|1664.9.peg.71

MTTFQHNLEKYAALIAKTGINVQDGDTVVIQIAVSQAEFARDIMAACYDLGAAEVIIKWQDDAIDRINMQHKAQERLIDVPDFQKQEYDYWLANDAKRISVMSSDPDNLKGLDGDRVAAAQLANGKAMLKVRQATQANKNSWIVVAAASPAWAHKVFPDLAVQEATDKLWTEIFKTTRVDQPDPILAWEAHGKKLHAKAAELNAAQFKALHYTAPGTDLTIGLPKHHIWMGGPHNSSDGRPFIANMPTEEVFSAADANHIDGYISSTKPLSYAGNTLNNMQFTFENGRVIKATAEEGNDVLQKLLQTDEGVKSLGEVALVPDPSPISQSKITFYNTLFDENASNHLALGSAYAFSIKGGTDMTQEELKAAGLNRSQAHVDFMVGSSDMNIDGLTHDGQIVPVFRNGDWAN

>fig|1664.9.peg.73

MSKIKVMTVFGTRPEAIKMAPLVLELKKRDTEFEAVTVVTAQHRQMLDQVLEIFKIKPDYDLDVMKQRQTLSEITSNVLMNLDHVIATEKPDIVLVHGDTTTTFAASISAFYNQTAIGHVEAGLRTWDKYSPFPEEMNRQLTDVLSDLYFAPTSQSKANLLQENHNEDNIFITGNTAIDALKQTVQSDYDHDILNVVDADKRMVLVTMHRRENQGEPMKRVFKVMRQVVESHDDVEIVYPVHLNPVVQEAAQSILGNHPRIHLIDPLDVVDFHNLAARSFFIMSDSGGVQEEAPSLGKPVLVLRDTTERPEGVEAGTLKLVGTQPEMVHDAMVELLDNQEVYDAMAQAKNPYGDGFASKRILDAIAYHFEQTKERPEEF

>fig|1664.9.peg.74

MSKTISIIVPCFNEQESVPLFYAAVEKIAAQLPDHAIEYLFINDGSADQTLPAMRTLQAQDPEHVHYISFSRNFGKEAALYAGLQAATGDYVAVMDVDLQDPPALLPEMIQGIEVEGYDCVGTRRTTRAGEPPVRSFFAKKFYQIINHISQTKIVDGARDYRLMTRQMVDAILEMTEYNRFSKGIFSWVGFDTKYLEYQNQDRVAGTTSWSFWGLFKYSLDGIVTFSETPLAIASFIGFFSFIIASIALIFIIVRAIIFGDPTSGWPSLISVVLMIGGLQLLCLGIVGKYIGKIYLEVKNRPVYIVKEKK

>fig|1664.9.peg.75

MIKTIQNLIHKYWEQLMYLVFGVLTTAVNMVVFYLLDQYTGMYYLLSNTIAWFLSVLFAFFTNKTWVFQSKYTTFRDFSREIASFFFFRGISYIMDTAIMFVGISMLHGPNMVVKIIDQFVIILANYIFSKWIFNKSTEA

>fig|1664.9.peg.76

MAFIKRLLFKYRDLLTYTFFGFLASLVNIFTYWIFRHTIIWPYLLANTIAWLLANLFGFFTNKSLVFQSKYTTFKALFGEVLSFFFFRGVSFFLDNGLMIVAISFLGWQSMTAKIVDQLIVGIVNYITTSYTFKKSERQMALRGRILRHYHTILKRGDHD

>fig|1664.9.peg.78

MATAKVVYASMTGNNEEIADIVEEALENLDVSVETSEISQADPSDFEDTDICIVCSYTYGDDGDLPDEAVDFYEDLKEMDLTGKVYGVCGSGDTFYDEFCKVVDDFAGVFEQTGATKGSDVVKVDLAPEAEDIEHLEKFVAEIVAKQSAL

>fig|1664.9.peg.79

MITLKSEREIKGMAASGAVIAGVHRGLRDIIKPGISSWVIEEFANDYIEKQGAKASEKGFEGYKYATCVCVNDEVAHAVPRKNLILKEGDIVTVDMTVNLDGYESDSCWTYAVGEIAPELQKLMDDTRKALYLGIDQAVVGNRLGDIGHAIQQYTEVENNYGDVRELIGHGIQPTMHEQPNVPSYGEAGKGLRLKEGMTITIEPMVILGGTWHIKSKTVPGDDWEYYVSADGTACAQYEHTIAITKDGPKILTSQDPEMDAKYLL

>fig|1664.9.peg.80

MKSDEQNQLPVFKRDISGKQKLIAFIKLMIRRSSEANISDNAKIIAYYALLSFFPLLIVIGNILPYFQLDVLSVADYVKSAVPPTIFNRIMPILQSLLNKRNSGLLSVGILGTLWAASKGINALKISINRAYRVDKIQNFILKRLISLGTTLVLLLLLVSLIVVFTFGQQFLEFITPIFKIPEDYINVFGQLKWPITSIVLFVILIFVYFFVPNVKMQLRSVLPGAFLTTVGWLILAQAFSLYMRYFGTSWNSYGTIGAFIILLLWLNYSAMVLMFGAVVNVAIEEAGSGQVDVSQGKVHDFIERRKEQNES

>fig|1664.9.peg.81

MNSDEPELHRHHHHKHHKKTWTPWKIILLVLGILVLVGGAFAAKVYYDVDQTAKTVYKAKGKTQNKRKANQAVKLNKKTPFSILLLGTDTGELGRTEKGRTDTMILATVNPQTKQTNMLSIARDSRVTIAGYGQKGKINSAYALGGIPMTINTVQSFLNVPVDYYVMMNMKGLEQLVDAVGGVTVDNDLDFTYEGHHFEKGPVTLDGETALKFSRMRYDDPRGDFGRQLRQQAIIQAVLKKATSLNLVTQYNKFLQILENNMQTNMTLKDVLNIQHNYGGAMNFKTKQLKGTGQMIDGQSFQVMSDNEVSNMSTLLRNQLDLK

>fig|1664.9.peg.82

MEQTINIEQIFGILRKYSRLIVLSTVIFTVLAGILTFFVITPQYSASTELLVNRKQNTDANVQYNQVQTDVQMINTYKDLITKPVIMSPVAKKLNDGKPGKLSDDQIASMISISNNQNSQVFSVTAKADNAYTAADIANMTATTFQKKAPKIMSGTDNVSIISKAKPNLTPVSPKNKLNVLIGLILGLLIGIGIAFIRELMDKTVKDESFLTEELGLTSLGVVNNIAPKDLIKKAIMRTSTLSRRG

>fig|1664.9.peg.83

MGLFRKEKKLDQDSMKEGVGLITLTSPTSVIAEQFRTIRTNIQFSSIDQKLRSLVFTSSGSSQGKSTISANVAVTWADQGVNVLLVDADMRRPTIHQTFQVPNKKGLTSLLTEDEFDFNTTIQKTPVEHLFVLPCGVVPPNPSELLNTKKMDKLIVELTKHFDLVIFDAPPVISVTDAQILASKVDGTILVAPQGIADKRSVIKSKELLDVVHARILGTIMNRVKPENTGGYYGGYYGGYYGAEESKK

>fig|1664.9.peg.84

MEIQKQDTSRASIENTRQVMLFNQAVLDQRYIYRSLKRLFDIVASLAGLILLSPLFLVLAIWIKIDDSAGGVFYSQTRLGLKQKKFKMYKFRSMCSDADQKLKDLLKYNEVEGAMFKMKEDPRITSVGRFIRKYSIDELPQLWNVLVGDMTLVGPRPPLEREIKEYTDYDWQRLMVKPGCTGLWQVSGRNSIGFHEMVELDLKYIRKSSLVYDLGILFRTVKIMIMPNDAY

>fig|1664.9.peg.85

MVKNVFIIGAKGIPANYGGFESFVEQLAARKESKNIHYYVACRRDLSENKADTFEYNDATCFNVDVPDVGPAKAILYDVNAFKWTMNYIKKNNIQNAIVYVLACRMGPFIKHFKKQLQQYNGTLFVNPDGHEWLRAKWSMPVRKYWKYSERLMVKHADLLICDSQNIEKYIQNDYKQYSPKTTYIAYGSDIIKSSLTDTDQVVTDWYTKHAVKLNNYYLIVGRFVPENNYETMIREFMNSATDKDLVIISNVEKNKFYADLQNKTNFESDPRIKFVGTVYDAELLKYIRENAFAYLHGHEVGGTNPSLLEALSSTRLNLLLDVGFNKEVGQDGALYWHKNDLAKVIDYSEKLALNEVESFNQVSSERINNAFSWHKIVTDYEKTFTMEK

>fig|1664.9.peg.89

MKIIRRFRQACGLGILYCSKLAIRSKNINVYGSWFGQKFADNSAYLYLASLENKDVRNVWITKNKEIVERLKKDNFEIYYAYSLKGIWWQLRAENIFVNTGAADVEKNLIGGANLINLWHGVPLKKIMYEVNPPRLEEKVRLKATNNFIINTSKQMRPIYKAAFRVDEEHSITIGQPRTDIFFDKTNRYDFFKKVPESLETRIKNKKILLYAPTHRLEGKKIIDVENLVDLKKLDKLMADNGCIFLIKKHFYQVNDPEIPSFDNIIDITKVDIDTQFLLSVTDLLITDYSSIYIDYLLRDKPIMFFRYDLKEYLKNDREMYFDYDEVTPGIKVSTGDELLGALNSILIQGDKDYVEMNKNTKNLFFGNDFRNSSRRILDKFVAK

>fig|1664.9.peg.91

MKGIILAGGSGTRLYPITKAVSKQLIPVYDKPMIYYPMSTLMLAGIQDIMIISTPTDTPRFEELFGDGHDLGLNIEYAVQDKPNGLAEAFIIGADFIGDDSVCLILGDNIYYGGGLSEMLQRTAQKEIGATVFGYHVNDPERFGVVDFDENMHAKSIIEKPEHPASNYAVTGLYFYDNQVVEIAKNIKPSPRGELEITDINKAYLEKGQLDVELMGRGFAWLDTGTHDSLHEASSFIATVQKRQNLKVACLEEIAYRMNYIDKAKVIELAQPLKKNDYGQYLLRLVKED

>fig|1664.9.peg.92

MGKLKVTTTKLQDVKIIEPAVFGDHRGFFTESYSDRDFKEAGIDIDFIQDNHSLSTQAGVLRGLHFQRGKAAQTKLIRVVTGAVLDVIVDVRAGSPTYKQWEGYILSASNHRQLLVPKGFAHGFVTLTDNVNFLYKCDGYYDAEADGGISFKTPELNIDWPIDFDQAITSEKDANQPTFTEFEKDNPFVYGEI

>fig|1664.9.peg.93

MKNIIVTGGAGFIGSNFVHYVVNNHPEVHVTVLDKLTYAGNRANLAGLPADRVELVVGDICDAELVDKLVQNTDAVVHYAAESHNDNSLKDPSPFIQTNIIGSYTLIEACRKYNVRYHHVSTDEVYGDLPLREDLPGHGEGAGEKFTPETRYNPSSPYSSSKASSDLLVRAWVRSFGLQATISNCSNNYGPYQHIEKFIPRQITNVLSGIRPKLYGSGQNVRDWIHTNDHSSAVWAILTKGKIGETYLIGADGEQNNKDVLELLLELMGQPKDAYDQVKDRPGHDLRYAIDSSKLREELGWQPEFTDFKSGLQHTIDWYRDNEDWWQAEKAAVEANYAKNGQ

>fig|1664.9.peg.95

MKILITGANGQLGTELRHLLDENQIEYVGTDAAELDITNAEAVNTYFEANKPELVYHCAAYTAVDAAEEEPGKSINYKVNVVGTQNIANAAEAVGATLVYISTDYVFDGTNNQMYTEETPAAPKNEYGRTKLTGEEAVANTMSKYYIIRTSWVFGEYGKNFVYTMLNLAKTHDHLTVVSDQVGRPTWTRTLAEFMLYSVKNDVPYGLYQLSNDNSCTWYEFASEILKDKAVEVSPVTSDEYPQKAFRPRHSIMDLTKIKATGFEVPTWQDALKSFMTKIGE

>fig|1664.9.peg.96

MKKVITYGTFDLLHWGHIHLLERAKSLGDYLVVAISTDEFNQIKHKEAYHSYEHRKYILEAIKYVDEVIPETDWEQKKTDVAKYDIDTFVMGDDWKGQFDFLKDQCEVIYLPRTEGISTSKIKNDLNIK

>fig|1664.9.peg.99

MKVRKAVIPAAGLGTRFLPATKAMAKEMLPIVDKPTIQFIVEEAKASGIEDILIITGKGKRPIEDHFDSAPELEQNLKAKNKTKMLKMVNETTDMGVNLYFIRQSHPNGLGDAVRLAKSFVADEPFVVMLGDDLMEDEVPLSKQLINEYEETHASQLAVMKVPHSEVDKYGVINPENKVKDDLYNVKNFVEKPDVDKAPSDLAIIGRYLLTPEIFDVLENQKPGLGGEIQLTDAIDELNKTQRVFAHEFKGRRYDVGNKFGYLETSIEYGLKHPEVKDDLKKYIIKLGEELKQSEKKENK

>fig|1664.9.peg.104

MIKPIMHDTEFLSQVALPATTADTAVITDLIDTLQANTDRCVGMAANMIGVNKRIIIVQMGILPVIMVNPKIIRHSNPYQTEEGCLSLVGQRSTKRYETIEVSYQDRQFKAQQQAFSGWVAQIIQHEIDHCEGILI

>fig|1664.9.peg.106

MKKITLFLSSLLLLGLFVFGSQQVQSVQAAEKTYEIGTDVTYPPFEFANKDNKYVGIDIDIMKAIAKSEGFKVNIQPLGFNAAVQAVQSGQIDGVIAGMTITDERKAKFDFADPYYHTGVVMAVKKDSKITGLKGLKGQRVAIKTGTAAGDYAKSKAKEYGFTTVTFDDSDNMYNDVVNGNSVACFEDEPVMQYGIKTGLGLKIVTKPVQGGDYGFAVKKGTNPELVAKFNKGLKKLKASGEYAKIQAKYLNTKASSEKEADHSFFGLLKENKGAILDGLGQTLLLTVIAIFFATIFGVFFGLLGVLPNKFARGVSDTVIYIFRGLPLLVLALFIYTGIPSLTGQKIPAMVAGVITLMFNEGAYTAAFVKGGIGAVSAGQMEAARSLGLPYGKAMRRVILPQGIRIMVPSFINQFIITLKDTSILSIIGIVELTQAGKIIIARNLEGFKIWGMIALIYLIVITLLTWLSKYIERRMNN

>fig|1664.9.peg.107

MNRIEVSHLIKNYGSNEVLKDLDLTVGNNEVVVMIGPSGSGKSTFLRCLNRLEEPTSGEIIVDGYNLSDRKSDLNQIREKIGMVFQHFNLFKNLTVAENITLAPVELGKMTPEEAKETAKHLLETVGLSDKYDAKPSSLSGGQKQRVAIARALAMKPDILLFDEPTSALDPEMVGDVLEVMKRLAKEGMTMVVVTHEMGFAKEVADRVIFMADGHIVEQGTPDDVFGNPQNDRTKDFLNKVLNV

>fig|1664.9.peg.108

MVRQQPEVHVTVLDKLTYAGNRANLAGLPAERVTLVVGDICDAPLVQQLVQKVDAVVHYAAESHNDNSLQDPTPFIQTNIMGSYTLIEACRQYHVRYHHVSTDEVYGDLPLVGDAKFTPETRYNPSSPYSASKASSDLLVRAWVRSFGLRATISNCSNNYGPYQHIEKFIPRQITNILSGRRPKLYGTGQNIRDWIHTDDHSSAVWTILTRGRIGETYLIGADGQRTNQAVLEMILTLMGQPKDAYDQVQDRPGRDLRYAIDATKLREELGWQPQLTDFKVGLQQTIDWYRHHEEWWQADKAKVEARYARNK

>fig|1664.9.peg.110

MDRYAVIYNPHSGQDHGESVGKKIEQALVKNQQTVELMPTKGPKDATRLAKKAAQAQFDIVVAVGGDGTINEVVSGLATLEQPPYLGIVPAGTVNNLARVLQIPLDIDQAIENLQQGHLQPLDVGQINDDYLISTMTLGILADAALNVTQSEKQKWGPLAFLNEGIRVLAKHQHYPLTIETPHRHWQKDTQFLLVTLTNSVGGFTKFNPEAQPDDGYFHVFVAPKMSLMRSAMFLPYFLTGNFKKIPGMTYFKADKITITTDNQESVQTRIDGDPSTKSPLNMRLLQHKLQVITPH

>fig|1664.9.peg.113

MSLSNPLTIDWQQLLLAIMRNCALLILMPLGFAALLTVINRSTKTRLARWWPRSQIVIGGLGTIIHELSHALFCFIFNHQITDMQLLNRNLNDSNDHSLGHVEHRYRSGQLWPTIGNFWIGLAPIFGCSAAILGLTWLLARNTFQTWLTFAEEPIWQWQAVKPLFSQLLTTTKPSLLLIWLILCGMIAIGGFDLSKADYRGMIPGIIATLIVIIGGTFIASLFNQTTSGTSWLLTILTPLMVILTIVVLLSLISWLIVATLLKMPQRSRYHARH

>fig|1664.9.peg.116

MKEVVIMSAKRTPIGKFGGQLASLTAVDLGTIAAKAAIQAAGIEADQIDQAIFGNVLQAGSGQNVARQIALNSGLAQTSVAMTVNEVCGSGLKAIRLAQSAIVMGDADVVLVGGTESMSQAPYLNNGMRFGSKFGDQTVVDSISSEGLNDAFTNKPMGITAENVAAKYGITRLMQDTFALESHQKAAQATQAGWFDAEIVPVTVKQRRATFEVSQDEGIRPDTTLEAMGKLRPAFQADGTVTAANASGINDGASAMIVMSKEKSEALGLVYQATLVGYQEVGMDPNYMGYTPVPAIQQLLAKQDQTVADIDLFEINEAFAASSVAVQNGLALDTAKVNVAGGAVALGHPIGASGTRIMTTLLHQLKRTNQTTGVASLCIGGGLGIAYEIQLNEAWLND

>fig|1664.9.peg.117

MTSPKFYQLSKSERLARLVEASQLSKKDAQFLAQSQPLADELADSMIENYIGQYHLPLGIVQDVMVDGKVYQVPMAIEEPSVIAAANNAAKMMRLNGGVTIESSRRVMFGEVVLTDLPQLADAVKWVDNHFTVLQAVAQKAHPSIVKRGGGLQKIATTIVENRFLKLNLEIDTKAAMGANIVNTICEAIAHAVQATLGGTILLSVLTNAAFGSVVKASVTLNPATLATATLTGEAVAQRLIDATQFAQFDVARATTHNKGIMNGIDAVIMATGNDWRAIEAGAHSYAARTGHYQPLTAWSWTTDHQLCGQIAVPLPVGIVGGSIGILPTVQVAQRLMAIETVEELAGVIAAIGLGQNLAALKALVSDGIQKGHMALQAKSLAIAVGATASELTTLLPQLLKAPHLNTETATTLLSQIRAENR

>fig|1664.9.peg.118

MQIGIDKLGLFTPNTYLDLVMLANARGVDPDKFTIGIGQDQMAIAPLSQDSVTMGANAALDLLEGESRDNISLLILGTESGIDQSKAGAIYIQRLLGLSNARTFEIKEACYGATAGLMTAYDYVAAHPDQKALVIGSDIARYGLETPGEVTQGAGAVAMLVSAQPRLMVLEPETSVHSADIQDFWRPNYSKEAFARGKYSTEQYIDFFQKTWADYRVKTGRTIADFKALAFHLPYTKMGLKALRTILDEGTEEQQAQLLERYAESTLYSRQIGNIYTGALYLGFLSLLEHSTNLEAGDRIGFFSYGSGAVSEFFTGILQPGFEKQLAKQKHTDLLAKRRQLTMVEYETQFQTELPEDGSEYEIDLTMDNAPIILKGVKDHERIYMTR

>fig|1664.9.peg.120

MKIYFANALFSQADFDYNTRVVAALRQAMPTLDIYLPQENAAINDKEAYANSEMIAQADTEQLVASDLVIAVLDGPTIDVGVASEIGVAYARNIPIVGLYTDSRQQGATNSQKVAALQTIAENQFHYLNLYTVGLIKLNGTIVNSVDALIETVQKY

>fig|1664.9.peg.122

MPEQTTTNEVEVGQRFPLTIKRIGINGEGIGYYKRKIVFIPGALPEEVVIAEVTTVAPRFIEAKVHKIRQESPQRVTPRDATYGQVGGIELEHLSYPGQLAFKKDMMIQALEKFKPHGYNGYQVTETVGMENPWHYRNKAQFQVRQLGDHLGAGLYAPSSHTLIDLPEFATQTDLTMRIIRTVLKLVEDLGIPVFDEEKNAGILKTIVVRESFSTGEAQLTFITNSPKLPKKYALITAIQAELPEVVSIMQNVNKGKNVLVWGDQTMHLAGQETIMENLNGVQFELTARAFFQLNPIQTAKMYNLVKEALDLTPEDRLVDAYCGVGTIGLSLANQVAEVRGMDTIADSIEAAKHNAEINGIDNVDYAVGAAEDLLPEWLDNGFVPTALVVDPPRTGLDPKLIDAILDSRPLKFVYVSCNPSTLARDLKTLVHTYQVEKIQPLDMFPQTARVEAIVTMRLKRNL

>fig|1664.9.peg.124

MPQITKITSQKQKGRYNIFIDEQFAFGVTESVLIKFRLAKGLEIDSSLQRDIQKEDDNAKSYQLALNYLSHQLRTEKELTQYLRDHEITPEGIETSLTKLRELHYLDDQDYADSYVRTVINTSDKGPKVVRQKLIQKGVSANHIDQALTLYTDEEQVTVGLATAQKLAKKYRHSSFFEQQQKIKQGLMQKGFGGDNLSRIIDELALEEDSDSEADALAALGEKKWRRYRLLEPRERRMKMQQALYRKGFNIDAINRFIDDQEVSDDTE

>fig|1664.9.peg.125

MTPSEREAFQTAFMAWYDEHRRDLPWRQNQEPYRVWLSEIMLQQTQVQTVIPYYERFLATFPNVEDLAAAPEELLLKTWEGLGYYSRARNLQKAAKQVVDDYQGKWPQTSAELEKLAGIGPYTAGAIASICFGEVVPAIDGNAFRVFSRLLKIDADIANPKNRSIFYDAILPLIPKDRPGDFNQAVMDFGSQVCTAKNPTVGDTELAPFFRSYQDGTLLDYPVKTKKAKPKPVALFAVVIESEKGFLFQKRPSTGLLANLTTYPLVMAEDLQDDESELLTPEEQMTRIEAYFKEAYGLTLAHLKPVPVKPVTHVFTHLKWTITLLSATIAKDSDLAFFPGEWYSKAALAEIAMPTVQKKMAQRFDSL

>fig|1664.9.peg.126

MNAPKEGDYIAIQSYKHDGSLHRTWRDTMVLKTSENAVIGCNDHTLVTESDGRKWVTREPALIYFHKKYWFNVVAMIREGGVSYYCNLASPHVMDQEALKYIDYDLDVKVFPNGEKRLLDVDEYEAHSAKWHYSAETDRILKANVKVLVDWINNGKGPFSKEYIDIWYNRYQELAHHR

>fig|1664.9.peg.129

MQIKQTRDLTSPYYLDGLTIRQAVFVAEQKVPAEMEIDEFEDQALYFTGYLDQQPVATLRVLTEGAFYHVQRVATMKAYRHQGLGLELMQAAEESAKKAGHKGLILNAQVTAVPFYERLGYQATDKERFLDAGILHQEMIKII

>fig|1664.9.peg.130

MFERLRQSKLLFWSTELLILAVLIFVCTKISFLFDPVGTFISTLFGPVIGAGLLFYLFNPLINLLGKLKISRKWAIAIIFIVFFGGIIFIVALAIPQLIHQVTQVVTNLPDYVHRLQDMIDKISTNPHLKNIDVNKYVNQLDLKPSKIAENIMKSFTSGFGSMIGAVTSVTIGVFTVPIMLFYMLKDGHRLIPNIQKMLPDRYNDQVADLLRKMGATISAYIGGQALECLFVGVFTFIGYLIIGMPYAYLLGFIAGVSNIIPYLGPYIGIAPALIISLSISIPKTIMVIVVVVIVQQIDGNLIYPNVIGRSLDIHPLTIIIILLVAGNIYGILGMILAIPFYAVTKTVVVYLYDIQALRTKVDKATKL

>fig|1664.9.peg.131

MWGQLLLILILTLVNAFFAAAEIALVSLSRTKMENQAENGDKKARLLVEVLNHSNNFLATIQVAITFAGFLSSASAATTIAGRLAPLLGNAAWTKEAAVLIVTIVLSYISLVFGELYPKQVALHRSEAIAKAAVQPIRFIGVLLRPFVWLLSASTTVLMKLTPIDFSETEPKLTREEMVHMIESGKSAGVLESDEYEMLEGIITLNKKMAREVMVPRTDAFMVDICADNDQNIDAILAQPFSRIPVYRDDKDQIVGIVHIKNLLREAREKGFSETQLETVMKEPLFVPETIVIDDLLFEMKRTQLQMAILLDEYGGVVGLVTIEDLLEEIVGEIEDESDKADVLYQKIDETHYKVMGRMPINEFNDAFQTDIQIADVDTIAGFMITEIGAIPVSGHPESIDLPNGIVLTTGSVVGSRLLDLKLTLPTTVAAKDDASEA

>fig|1664.9.peg.132

MTPTELKTAVERRRTFAIISHPDAGKTTITEQLLLFGGVIRQAGTVKGKKSGQFAKSDWMDIEKQRGISVTSSVMQFDYAGKRINILDTPGHEDFSEDTYRTLMAVDAAVMVIDSAKGIEPQTKKLFKVCKMRGIPIFTFMNKLDRDGREPLDLIAELEELLDIEGCAMNWPIGMGKDLRGLYDIANKRIEMYRPEDEANPYLALDEEGRIAGDNPLKEDSVYTQALDDIELIGEAGNAYDPAKIATGDQTPIFFGSALTNFGVKTFLEAFVDMAPAPEAHQTQEETQVEPTNEDFSGFIFKIQANMNPAHRDRIAFVRVCSGEFQRGIDVTLTRTGKKMRLNNSTEFMADAREQVTSAVAGDIVGLYDTGNFQIGDTIHTGKEAISFEKLPQFTPELFMRVTAKNVMKQKSFHKGIQQLVQEGAIQLYKTYTTSDYILGAVGQLQFEVFQYRMQHEYNSEVIMEPIGSRTARWIDPEKLDQSMSSSRNLLVKDIHDQPLFLFENKFAERWFQDKYPEVKLTAKL

>fig|1664.9.peg.133

MNFINLIQSLFRRRPKRPTIQPETHFNQATSEPENANQFFETADQFEKEPQAQQTEHFVTTPVQPRKSSVTIHYLNQNRIPLRPSITLSGVVGATLDLPWLKFPGYYLAEITNLKQHFHEDPTNIWLFYKPQLAAPVMVLHQDLEGGLLIKPQFLLGALNEHYQANPLEGGVNFIYRTSSNQTGRFSAETQLVRFQYDPLNLKYSDAPDQPFIELTASVDTYKRPTEETLTTTRLPKNAIWKIYSCATATSGQQWFNLGSFWISPKHYELHATNPKATVNYQVPKFTNQYTVIETTPLNLDATLNNTTATLWAAPYEKPATYCLALKTRVHVKQVIALDNLSRWCELEGGQWLLESLLTFD

>fig|1664.9.peg.134

MSSITYSERIKIETFCELGLSNIQMGVRLNRSPSTISYELSRCQPYQAELAQIDAEYKRSRCGRKTKLSDELKQKILNHLRLSWSPGMIAHEFKLATKSIYNWLNQGRIGFSLNDLPEHGVRQRRNVDQRSKYNQSLGRSIEQRPIVVNRRNRIGDFELDTIVGPRGHSKAVLLTLIDRKSRFLWAYRLKNRTAVTVNKALNKFLATFNGPVHSFTVDRGTEFSGLVSLEAQYGIKTYYCHAYTPAERGSNERFNRNLRYFYPKGTYFEHISAQGLKTTLLEINQRPLKILDWQTPYQVMLTNLSKNSD

>fig|1664.9.peg.136

MRLINITNSYHRLVTQQLATTKADYVSVYSLGKTTVLFTRSSKTREILLKNDKRHIQQAEIDFVLKELVDIDSTDDVEILNDGSLVEITIPTPSTTAS

>fig|1664.9.peg.137

MLCQNCQKNPATIHLYTSVNGQNQAINLCQNCYQALKQQGMSGQYNDPFGFSNLDELFNAINNPNAERNAANQGPQTQGGRNGGGNNGQNGESLLSQFGVNLTNLAKGGQIDPVIGRDKEIARVIEILNRRTKNNPVLIGEAGVGKTAVVEGLAQKIADGDVPAKLKDRQVIRLNVVSLVQGTGIRGQFEERMQQLITELKQNKQIILFIDEIHEIVGAGNAEGGMDAGNMLKPALARGELQLVGATTNNEFRQIEKDAALARRLQPVAVNEPSVDETVLILKGLQDKYESYHHVHYTDEALKAAAALSSRYIQDRYLPDKAIDLLDEAGSKKNLTITLMDPKELEAKIKSAEAQKQEALKQEDYEKAATYRDQVTQLSEMKANNSVEKAQEPTITEKDMEKIVEEKTHIPVGELKAQEQAQLKNLAQDLEARVIGQDTAVDKVARSIRRSRIGFNKSGRPIGSFLFVGPTGVGKTELAKQITKELFGSTDAMIRFDMSEYMEKFSVSKLIGSPPGYVGYEEAGQLTEQVRRNPYSLILLDEIEKAHPDVMHMFLQILDDGRLTDSQGRTVSFKDTIIIMTSNAGQTDAEANVGFGAAVAGQTHSILDQLTNYFKPEFLNRFDDIIEFQPLSKDNLLKIVSLMLDQTNAMIADQGLHIAVTDDAKSKLVDLGYNPEMGARPLRRVIQEQIEDRVADYYLDHPEHKQLKAQLNDGQIQIVQG

>fig|1664.9.peg.138

MARDFLAEMDALLNHEKETIVVEHDEFLDFQKVWVNYDHRKEIVGAAQRNGQVIYRCAESGIV

>fig|1664.9.peg.139

MEKRDFHVVADTGIHARPATLLVQTASKFNSDVNLEYKGKSVNLKSIMGVMSLGVGQGADVTISAEGADEADAINAIEETMKKEGLSE

>fig|1664.9.peg.140

MTKLRGIAASDGIATAKAYMLVQPDLSFSKSTISDSEKEINRLHKALQDSTSDLETIRKIAAESLGEEEAQVFDAHMMILADPEFTGAIEGKINDDKVNAEQALKEVADLFVATFEAMTDNAYMQERAADIKDVTKRVLSHLLGVTLPNPALIDEEVIVIAHDLTPSDTAQLNGKFVKAFVTDVGGRTSHSAIMARSLEIPAIVGTETVTQDVKAGDLLIVDGINGDVVLDPTDADIAEYDVKAQAFADQKAEWEKLKNEKSVTKDGKTFTVAANIGTPKDLAGVLENGSEAIGLYRTEFLYMDSAELPSEDDQFEAYKSVLEGMDGKPVVVRTMDIGGDKKLPYLPLPEEMNPFLGYRAIRISLDRDDIFRTQLRALLRASNYGKLRIMFPMIATVAEFRQAKGILEDEKAKLIAAGQTVSDDLQVGMMVEIPASAVLANQFAKEVDFFSIGTNDLIQYTMAADRMNERVSYLYQPYNPAILRLIKNVIDASHKEGKWTGMCGEAAGDSIMAPLLVGMGLDEFSMSATSVLRVRSLMKRLDTTELTDLVETAVNVNTSNEENQKLVEDFMKDR

>fig|1664.9.peg.141

MAENTNEITTSEWEVMRIVWSLGQVNSRDLIDLLQQKRDWQDSTIKTLIGRLVKKGFLKTEKEGRRFNYTATIPEIEAMENATQSLFEHLCGMKKGQTLAALIDQTTLSQTDILRLQQLLTAKAATAPEKVACDCLPNKCDCEKEE

>fig|1664.9.peg.142

MKHDDMKMHDHEMGEMDMHDMHDMDHDHGDMMMHGGHMMHMGNLKQKFWVSLVATIPIIILSPFMGIKLPFQTTFPGSDWLVLLLVTFLFFYGGAPFLKGAKGELAEKNPAMMTLIAMGITVSYGYSLYAFIANHFLATQTHVMDFFWELATLIVIMLLGHWIEMNAVMSAGSAMEKMAALLPGQAQVLDQDGQQHVVDLKDLQEGQTVLVAAGEKVPADGLVVNGQSQVNESLVTGEAKAVAKKTGDQVIGGTVNGDGTLTVKVTGTGESGYLAQVMKLIKQAQQEKSKVEGLADKVAKYLFYAALVAGIIAFISWFFLADLNTAFERMVTVFIIACPHALGLAIPLVVARSTAIAATNGLLIRNRQAIEVADQITMVLMDKTGTLTQGAFKVNAVQPTGEMSKTQLMAYMGALERHSSHPLATGILAYNDAEKITMPQAENVQTIQGIGLTGAIEGQTYQIVTADYLQEKQIAFDTAAFETLAAKGNSISYLIQGQTVLGLVAQGDQLKPEALTFIKALKKRHIQPVMLTGDNQQVAEKVAQQLGGMTVQANLKPEDKEALVQDYQAKGEVVLMVGDGVNDAPSLTRADIGIAIGAGTDVAIDSADVILVKSNPNDIMHFLNLAHATSRKMTQNLWWGAGYNILAIPLAGGLFAAWGLILSPAIGAILMSLSTIVVAINAMTLKLKN

>fig|1664.9.peg.143

MLEIREEQDPKMDLEIKVVKRDGRILPFSADKIEQAVIKAAKNVKDELEPLDYQILNSVATDVVSEVGARFDKDVKIYEIQNIVEHKLLEHHQYDIAQEYINYRTQRDFARNKATDINFTIQKLLAKDQTVVNENANKDSQVFNTQRDLTAGTVAKAMGLKMLPPKVANAHLKGEIHWHDLDYQPYSPMTNCCLIDFKEMLNHGFKIGNAEVEAPHSIQTATAQMSQIIANVASSQYGGCSADRVDELLAPFAQKNYDKHLTDAKNWIDGEAKQTAYAQTKTEKDIYDAMQALEYEINTLYSSQGQTPFTTLGFGLGTSWMERAIQKAILEIRIEGLGKEKRTAIFPKLVFAVKRGLNLAPTDPNYDIKQLAVECATKRMYPDVLMYDKLVELTGSFKAPMGCRSFLQGWKNEQGQEVNSGRMNLGVVTVNLPRIALEAAGDQAKFWEILEQRLKVCKEALVFKVERAKEAKPENAPILYRYGAFGKRLKKTDSVDEVFKNSRATVSLGYIGLYEVGAAFFGPDWEKDATAKQFTLDVVKDLYNHCVDWEKEYGYHFSVYSTPAESLTNTFCQKDTQKFGIVENITDKEYYTNSFHYDVRKAPTPFEKLDFEKDYPKYCAGGFIHYCEYPNLRQNPKALEAVWDYAYDRVGYLGTNTPIDQCFKCGFKGDFKPTERGFQCPQCGNTDPATCDVVKRTCGYLGNPQQRPMVHGRHKEISSRVKHMHITIGNDNNNVARQ

>fig|1664.9.peg.144

MGNTKRGPKNPAPQEWLSEELSQDYIADYKPFNFVDGEGVRCSLYVSGCLFACPGCYNRIAQNFKYGRPYTKELEDQIIDDLGQPYVQGLTLLGGEPFLNTKTCLSLVDRIHETYGQTKDVWSWTGYTWEELMLESPDKLELLSQIDILVDGRFMQDKMDLTLQFRGSSNQRIIDVPKSLVAGKPVIWDKLAR

>fig|1664.9.peg.145

MDSVISFMEKRFLPVAAKVGNQRHLIAIRDAFVTTMPLMILGAFATLLNNLPIPAYTNFMNSVFSKTVGTGADKAYIWTKFGGNAWSGTFAILSVFIVFLVAYNLGKSYDVNPLASGVTALGSFFAVGGLAGMDSMGLFVALIVAMISTEILRRLLGNKHLVIKMPDGVPPAVAKSFAALLPSMITISIFALFATILLGFGIENIVTSFYKAVQEPFQGLANSYPSALLLAFITPFLWFFGLHGANMIEPFMQTINAPAITANITAIKAGKVAPYIVNKPFFDSFVNLGGTGATLGLIFAIWLVGRKNKQYMIVGNLSAAPGVFNINEPLTFGLPIVLNPILFIPYIVTPMVLVTIAYFATSTGLVPAATIMPPWVTPPIIGGIMASVSWQGGGLAAVNLLVSTIIYLPFTKIATSMALKEEAAREQQEAA

>fig|1664.9.peg.147

MTFKKFICDHLFHIVFFFGGMFVLDIVLWLDPHMRLAKETLMYLDFLLTIFFCAFLIGLYLYHRKWFRTIQIRLNAKEDALNWPLTGATSAEKQYFQKYVNSLLDYHQQSIERLMHAQQDQKDFIDGWVHESKVPLAATQLLVESIEDQIPEEKFNQLTDELVQIEHYVEQVLYYSRLDSFSKDYLVQEYALKPLINQTIRQNRNYFIQNRIQFKLTGEEQTVLTDAKWLVFILNQIVSNALKYTPQNGQITIDLAHDEQGVWLSVSDSGIGIPAEDLPRVFDKGFTGQNGRQSNQRSTGLGLYLAKSLSNKLGHELYASSTPGNGATFKLLFPFLSYYNDPDGERLMGKENHFR

>fig|1664.9.peg.148

MFEIMIVEDDPTITNLIAENLEKWQLKAIIPDDFDTIFDRFLTDKPHLVLLDINLPVYDGFYWCRKIREVSKVPIIFISSRSTNMDMVMSMNMGGDDFVNKPFSMEVLIAKINALLRRTYNYVDQNTDVIEHNGLLINLQSGGAQVGDTVVDLSKNEYKLLQFLMRQHGQIVSREKLLRALWDDERFVDDNTLTVNINRLRKKIEQAGLEDYIQTKIGQGYIIP

>fig|1664.9.peg.149

MTVVAVQQVSKVYGHRFNLVNALNNISFEIEEGEFVGIMGPSGAGKTTLLNMIATIDRPTHGQVMIKNQVVTKMREAKLANFRRHDLGFIFQDFNLLDSLTVRENILLPLALDKMSVALIEKRLAQVTQVLGIGGLLDRYPDEISIGQRQRVACGRAMITNPTLLLADEPTGSLDSKSATELLRYLTEINHQEKTTILLVTHDAFTASYCRRILFIKDGQLFSEIVRSGDRQQFFNQIIDMQATIGGGGRYNAVTTPR

>fig|1664.9.peg.150

MLLRLLVRSTINRARDYAAYLMACIMAIVVYFCFTAIKTDPVLSKLKSAGERLIFGGTIQIASIIIILFAAFFLAYANLFFMKKRRQEIGMLSVIGVTRLQISVVFFAESLVIGTIALLIGLFFGIFLSKLFGMLLLRMMGISVAIPFLISWGAIKQTTITFGLLFLLTGLLNSSLIFRYQLVDLLHPTEFSRKIRRPNWLTTIWGIIGIVLIISGYYLAERILVLMPELERRYGYGIDAGYLGAILWFEVIGTLAFFQAYTQIWLRIERHWKRLYYRGTHLLSVTNLSYRFKKNAKTLWMITILSAVTITALGSAAMIYTFSQKTLRENIPADIIYSKYQKKGVEQILRAHQVTPKKITETTYKIVDAQFELATPLLSDSMTLAGATGVLPLSRYNALMARQFKLPATPLRPNEAIAMIDLPLTMIKMPHHKRLAHANQQGIPLQIKQPELPQLRIIHLRRLFPNGVSVNFYGSLNMVAVPDQYYDQIKPATIDHVYALDLTPKQRRDKKLMKSFFKLSQDTKKRSYLTAVRNENGFKRYRVTTKEERSGFVRADLLLRKPNQDRANANFGFYMYIALFIALTFMLATGSIIMLKQLSEAQEEILQYKTLKRIGMTYSEIKRTIYFQILIIFLLPIILGTLHAIFAIRLLSLFLDNPGLQLVYIVCGIFIMIYFVFYLVTAEIYNRIVNIPLNDDALY

>fig|1664.9.peg.152

MELTVLGYYGGYPYAGVGTSSYLLTSGDYHLLLDCGSGALLALEKELDPLQLDAVLLTHYHHDHIADVGVLQYYWQLQAGQKKEPILPIYGHLQDPLHFGSLTLPNVTQGFGYQEDTTLQLGPLKVTFKRTEHPVPAFAVRIEEETTGKVLVFTADTAYFPGLAEFCQGADLLMTDTNFLADRTGKMAHMTTTQSANLAQISGVKQLLLTHLPQAVTPETLLAQAKTVITTIPVTIAEQGLTIKID

>fig|1664.9.peg.154

MEKIDENTIRVLLENDDLEERGITGLDLLSNHKKIERFFYSILEEVDQDHVFANNDAVTFQVLPNEQGLELLISKNLSNLDLNKAQSEMVQGKDGITEYIKDQLLKRDTASSEDDEEDDEIESYLNQADNPTKQVVLGFDTFEDWIALAQQLHIESGVSNLFVYKHQYYAQLVFFLENTTETFVQDDLAVANEFGHKTNYTADFLSEYGQRVMENSALELTRYYFKD

>fig|1664.9.peg.156

MLEVFLYINPIGQRCLQSEKALLKIIDEADMNIHFKFIPVLNLQNVERYMQFQNLNRRDLDLRNHVFSTMYEAVLAYKAATFQGSKKSQAFLMQLQQVFQQPDTTLDTKLVLEIAEKAHLDTEMLLEDWHSDLTKQVFDSDQQLACEMNIKMTPSAVAFDYSKDDSEAGLLIENCDSYDLLKEVCSQGVSPEDTYQKLQKHKANVTKIAFRVLS

>fig|1664.9.peg.157

MTQTIEREFKVSLTEQQFKQLQAAYPWPTPFTQTNYYYELPQNELQRRHMGLRTRLYQDHAEQTLKVPQAGATRTLLEITDTMPLATAQQLIAQQTIQPNQQIAKYLETIGIDWSPLFIWGQATTKRRVLTLPSRRLTLDQTTYPDQTADYELELEFDDMILAQPFFQDIVARFKLTQTAPLNKIQRAKNHQS

>fig|1664.9.peg.158

MVENWKKFLMPYEQAVSELKIKLRGMRKQYQQNNEHAPIEFVTGRVKPIDSIKEKMQRRYITEDLLEQDMQDIAGLRIMCQFVEDIHEIVGLLHQRTDMKIIEERDYVSNVKPSGYRSYHIVVEYPVQLISGEKLILAEIQIRTMSMNFWATIEHSLNYKYQGEFPETLKARLKRAAEASFMLDKEMSEIREEIQEAQQLFSYGKWQSALEKATENDSEGDAPD

>fig|1664.9.peg.159

MADKLTNKLINNGFTMDAQTPEVVISVGGDGTLLSAFHRYADALDQIRFIGVHTGHLGFYTDWRDFEVDDLVVALQEDLGQSISYPLLEVKITYADTNEVQHFLALNEVTLRRYAATLRTDVYIKENFFESFRGDGLCVSTPTGSTAYGKSIGGAVLHPRLEAMQLTEIASINNRVYRTLAAPIVLPSDEWLLLRPSRTSDYVVTIDQFTFKDRPIESMQFKIAKERIQFARYRHTHFWDRVEDAFIGSKHEI

>fig|1664.9.peg.160

MNGQEAHTNIRLVQNDVVTVYLAPEVAVEHVIPVKGDLKIAYEDDHFLIVDKPAFVASLPAPNHPNNTMANFVKQYLIDEQAESLAVHVVTRLDRDTSGLMMFTKHGFAHSLLDRQLQSKELQKTYIAIASGDMREDLPQHAWIDRPIKRSEEFYMRRVVGEGGKRSLTEYWQAAHGDGATLVKLKLLTGRTHQIRVHFASLGHSLIGDNLYGESDDLLPRQALHCRQLTFYHPFEERTITVKSPVPADFSAVLAQRQFDFDETTL

>fig|1664.9.peg.161

MLTLNVIKAIILGIVEGFTEWLPISSTGHLVLVGSVLKMGESKAFMDMFNYVIQFGAILAVVVLYFHKLNPFSPQKNQLEQKQTWTLWFKVILAVIPSVIIGFPLNDWMDEHLMQNWVVASMLILYGILFIVIENRNKQRTPKFADLNTLPWLTAFWIGCFQALSIIPGTSRSGATILGAILIGTSRFVGAEFSFFMAIPTMIGVSILKIGKFFYQGNTFTGDQSIILLVGMVVSLIISIISIKFLMGYIKKNDFKVFGWYRIILGVLVLGAMFL

>fig|1664.9.peg.162

MLIKEVCVENFTEIPAAIRNGAKRIELNDNLAVGGTTVSRGVMAESVRYAHEHDVPVMVMIRPRGGNFVYNDQELKIMEADIFSAQELGADGIVVGCLTPDNKIDDEAMEMLIGAANGMQVVFHMAFDELAVADQAEAIDWLVEHDVKRILTHGGPLTAPLNEERLQELVRLADGRLGILPGGGITAENVAGITERLNVTEAHGTKIVG

>fig|1664.9.peg.165

MLKETRKQRIATQKIILNRQKKELRNKKVRHMTTFAGASFLMGSAVAPSLTSGKVKAAVETVSANETNLNTATSSTSSVANSTTSTSSTDQATSSSQSQSSQTTSSSQSQSQPVVKPQVTENDANKAATAQPKPTQAQPVVGTQNTGANIATAQAIMAYSSTSASTFINSIASSARQLASENDLYASVMIAQASLESGFGNSALGKAPNYNLFGVKGSYNGSSVYMLTNEDDGHGNLYQINAAFRKYPSYYQSLQDYVHVLKNTSFGSTPYYQGAFKSHTTSYQNATQYLQGRYATATNYAASLNRLIQQYNLTQYDTPATSGNNGGTGTTPTNPTTPTNGEKYTVKAGDSVYSIAKRYGISMSTLISWNNIKNNMIHPGDVLIVSANKPTTPTNPTTPTNPTNSTKPTNGEKYTVKAGDSVYGIAKRYGISMNTLISWNNIKNNMIHPGDVLIVSAKQTTNPTTPTNPTKPTNPTNPTTPANNQTYTVKSGDSVWAISNRYGVSMANLVKWNNIRNNFIYPGQKLIVGQKSTGQTTPPTNNNNNSSSNNNNGSTTTTAKTYKVKAGDSVWRISQQSGISMAELVRLNNIKNNFIYPGQVLKVSAGKTTTNNNNQTTPNKTKSYTVKSGDSLWSIAQKNGTTISQLKTANNLRSDLILVGQVLKLK

>fig|1664.9.peg.166

MTYDHRAVEQKWQAYWQSHKSFKTTEDKDKKNFYALDMFPYPSGQGLHVGHPEGYTATDILARMKRMQGFNVLHPMGWDAFGLPAEQYALDTGNNPADFTQKNINTFKRQINSLGFSYDWDREVNTTDPDFYKWTQWIFEKMYEKGLAYEAEVAVNWSPDLGTVVANEEVIDGKTERGGYPVYRKPMRQWMLKITAYADRLIDDLDLVDWPESVKDMQRNWIGRSKGAEVSFAVENHDANIDVFTTRADTMFGVSYIVMAPEHKLVADITTPEQKAAVDAYLKEIEHKSDLERTDLAKDKTGAFTGAYATNPVNGERLPIWISDYVLASYGTGAVMAVPAHDPRDWEFAKKFGLPLKPVVSGGNPEEAVHTEPGVMINSDFLDGLDKQAAIDKMIPWLVEHKVGHEQISYKLRDWLFSRQRYWGEPIPIIHWEDGTTSVVPEDQLPLELPLTSDIKPSGTGESPLANLTDWLNVTDENGRKGRRETNTMPQWAGSSWYYLRYIDPKNPDKLADFDKLKDWLPVDMYIGGAEHAVLHLLYVRFWHKFLYDIGVVPTKEPFQHLYNQGMILGDNHEKMSKSKGNVVNPDDVVDRFGADTLRLYEMFMGPLDASISWSEKGLAGARKFLDRVWRLYTEEDTDENDQLSSKIVADNNDQLKKVYNETVKKVTEDFESMHFNVAISQLMVFINDAYKADTFPREYAEGFVKLLAPIAPHMMEELWAMLGHDDSISYVDWPTFDPAALIANEVEVIFQVNGKLKAKVTVAKDTPKEELEAMAKANEKVAEFIADKTVRKVIAIPNKLVNIVAN

>fig|1664.9.peg.167

MKRNLNARQMQMIALGGTIGVGLFMGANTTIKWTGPSVLIAYGIAGLLLYLIMRALGEMLYVDPDTGSFAKFATEYMHPIFGYLTAWSNVFQFVVVGMSEMIAIGGYLEFWWPNLPGWVPGLVAITFLCAANLISVSMFGELEFWFALIKVVTIVLMIVAGLGLIIFGIGNHMHPVGISNLWTHGFFTGGFKGFVFALAIVLASYQGIELIGITAGEAENPQETLVKAIQSTVARILIFYIGAIFVILSIYPWNELNQVGSPFVQTFAKIGITAAASIINFVVITAALSGANSGIYSASRMTFTLANNGQLPKRFLKLNRHGVPFYAVGAISLGILIGVILNAILPLVYPKAGNIFVLVYSSSVLPGMVPWFVILISQIAFRKQHAEKMATHPFKMPWSPYSNYITLAMLCLTLVFMFINPETRISLIVGLIFLVLMIIIYFVKFGRQKE

>fig|1664.9.peg.168

MNEKTYNIGVEATMEIIGGKWKPVILCHLRNQPLRSSELLQNIPKISQKVLTQQLRELEADGITNRKIYQEIPPRVEYSLSEYGQSLNKILNLLCLWGEDNIDRRQSAGESINVLHRDQINPIY

>fig|1664.9.peg.169

MSKKQRANLTLLALAISAFAIGSTEFISVGLMPLMIKDFGITMSQAGLTVSMYALGITIGAPILAILTSRWPRKNLLLGIMIVFIIGNLTVAFAPVFSVVLIGRIISALSHGIFMSIASVIAADVVEPERRASAIAVMFTGLTVATVTGVPLGTFIGQISTWHMSFIFIAGIGVLGLIANWFLVPNDLPLGQPSQFSGIIRVFKNKAIVIALLITALGYGGTFTVYTFVTPILQTKMGWSPSAVVIILVVYGLMVAVGNTLGGKWSNHKPLLALLRMFVGLLVTLMLFMLTLNSHWFGLINVLLMGFFAFMDVPGLQLYIVQLAEKFTPNDIPLASALNISAFNIGITIGSLVGGQASAAWGLGSTPIFGSIMVFISILLTWYLIKQTTKTK

>fig|1664.9.peg.170

MTNSSDTKATDKSSQSKMLRGSAWMTAGSIFSRILGAIYVIPWPIWFGVNFLAANNLFGRGYQIYSVLIVVSTAGIPGALSKQIAHYNEMNEYGVGQKLFQKSLILMLIMGILSAGALYLLAPVLSQGDARMIPVFHSLCWPLLVIPLLSIMRGFFQGYAEMAPSAISQFIEQVARVIYMTVTVFVITQVLRGSYIDAVTQATFAAFIGAVAGLLLLIWHYLKQRPRIEKLIAGSNNAITVSTNDLLKGIIKQSIPFIVIDASINLVYIIDQYSFNRMMSDFVIASDHQLWNLYTLFAVNANKLISITVSLAAAMAVTAVPLMSAAHVRGNQKELRQQIGSTIQLFNLVMIPAAFGMAAVAGPLYTLFYSHDALGISVLQFSSYISILLGLFTVLGAILQGLYQNRLALFYLLIGFIVKVVAQYPAIYFFRVYGPLVSTGLAFATSSILMLAALHRYYHFDVWQTIRRLIGIVGFSVIMYLITSLTVKGLLLVFNPQSRLQSVLVLMISVAVGGLVYAVLALKSRLADYVLGSNLDRFRRKLKLK

>fig|1664.9.peg.171

MRIDRFLSHMNVGTRKTLKPLLKAGRVRVADKVIKEAKYQVTPETVVYVDDEPIRYQTNFYWLLNKPAGVISATTDPQKTVMDLFAPADYREDLFPVGRLDKDTTGLLLITNDGDLAHDLLSPKKHVAKTYRAKIAGVVTAADQAQFAAGLKLSDFDAQPAQLTILRTDTKRQESLIEVEIHEGKFHQVKRMFHAVDKEVLTLERLQMGPLRLPADLKRGQYRALTTTEIDALQTATQK

>fig|1664.9.peg.172

MQIITDELARQVIVARPAISHKGDFGRILIIGGSQQYGGAAIMSASAAVYTGAGLVSVASDLSVLSSLHTRLPEAMFIDYRQPFVQWVPAVQAADVIVIGPGLGLSAFASLLLKQVLQAVRPEQTLLIDGSALTLIAREKLALPDAQLILTPHQMEWARLSGLSIAQQTVANNQVALADYPKATLVLKKHHTEIYHQTQVSQLMIGGPAMATGGSGDTLTGIIAAFCGQFGTNYQTISAAVYTHSAISDQLAQKQYVTLPTQMIQALPQFMAQMAHQ

>fig|1664.9.peg.174

MTTKTFNIDGMVCASCAQTIEGATQKLPGMQKAAVNLATEKMQVEYDDSELSVTAIEETVSAAGYGATEQIDPEKEAQAIQSKRTAHLQALWRRFWLSALVTLPLLYLSMGHMVGLPLPAFWQPMQAPVNFAVTQLVLTLPVLYWGRQFFIVGFKALFNGHPNMDSLVALGTSAAWGYSLIATLAIISGHVDLVTSLYYESASVILTLITLGKYFEARSKGQTSSAIEKLLNLAPKQATVLRDGVETTIDVAEVVVGDVLVVRPGEKVPVDGELIAGHSAVDESLITGESLPVEKQVGDVVIGASLNKNGHFQFKATKIGKDTALAQIVQLVEQAQGTKAPIARLADKIAGVFVPIVIGLALLSGLAWFFLGQESWLFALTITISVLVIACPCALGLATPTAIMVGTGKGAENGVLIKSGDALETAQHIQTVVLDKTGTITAGQPIVTDMVVYQGTEDDLLQLAASVEAGSEHPLASAISQAASERQLTLSAMTDFTAIPGYGLSAQVANQTILFGNQQLMAENGVAIEIGLKTAQQLAEQGKTPMYLAQDGQLQGVIAVADPVKATSQAAISRLQKMGIQVVMLTGDNQKTAQAIAHQVGIEQVISDVLPEQKAAQIKTLQATGQLVAMVGDGINDAPALAQADVGLAIGSGTDVAIESADIVLMHDDLMAVPTAFKLSRKTMQNIKENLFWAFAYNILGIPVAMGVLHLFGGPLLNPMIAGAAMSFSSVSVLLNALRLKRLKLS

>fig|1664.9.peg.176

MKKFIVQSTDRQHQLHVVAWLPTQKPIAVVQLLTGMAEYIERYDALAQFLAARGIVVIGHDHVGQGHSVQTTDELGYFGPHGLQTLLNDCTLIGRIAHEEYPECPLFVLGHSMGSMLATQYVKQTTVPLAGAIFMGVIDVPVVLKPVLPLVNLIGALAPKQPGKLLNNLAFGVYPKRFDAKRPFSWLSYNQDNIAAYENHPLCGYVFSNSGFAMLLTLTELTRHADWQLALKERPVLVLSGQDDPAGGYGRRARHLATQFKKHTVTNATVRILPHTAHEILQETNAIEQQQAIYDWLLTAISQSPATRHNSR

>fig|1664.9.peg.177

MLEKNKMSLFSLVMLGLSSIIGSGWLFGAWEATTVAGPAAIISWIVGAIVIGAIAFNYVELGTMFPESGGMSHYAQYSHGSLLGFIASWSNWVSLVTIIPIEAVAAVQYMSSWPWSWANWTNRFMHHNEISTRGLLVVFAFIIVFTLLNFWSVKFLTRFTSFIAIFKLAIPLLTIIMLVSTGFHSENFGHSVATFMPYGSAKIFAATSISGIIFSYNAFQTVINVGSEIRDSKRNIGRGIAISLGISIVIYLLLQITFIGAVSPALIAKTGWHGLNFQSPFADLAILLGIHWLAVLLYLDAFISPFGTGVSFVASCARTLAALKQNKHMPPIVGKMNHQYNIPRVAMAINAVVSMLLVSVFRSWGTLASVISTATLIAYLTGPVTVMSLRKMAPDMNRPVHSPWMKVMAPLSFVLASLATYWAMWPTTIKVIGVIMLGLPFYFYYEYRIHWENTWAQFKGSLWLIIYLALLSLISFLGSREFNGINVIPYPLDFVLISALALIFYYWGIISRFYSKYFSRAKRINQDVQL

>fig|1664.9.peg.179

MDFKQQVVTALTGVLGDSLPAEKIAQLIETPKTSDLGDYAFPTFILAKTLRKAPQQIAQDLVDQMDVAGFEKVIANGPYINFFLDKAAFSDQILKTVLTEAAKYGESDLGHGGNVPIDMSSPNIAKPISMGHLRSTVIGNSIAKILTKVGFNPIKINHLGDWGTQFGKLIVAYKKWGSEEEVKKDPITNLLKYYVKFHQEDVEHPELDDEARAWFRKLEAGDEEATQLWSWFRSESLKEFQKIYDMLGVEFDSYNGEAFYNDKMDAVVEAIESKGLLQESRGAEIVDLTAYNLNPALIKKSDGATLYMTRDLAAAMYRHDTYNFVQSLYVVGGEQREHFNQLKAVLKEMGNDWSDEIHHIPFGLITQGGKKLSTRSGRVILLEEVLNDAVKLAGAQIEAKNPDLPNREEVAKQVGIGAVIFHDLKNDRLDNFDFDLEEVVRFEGETGPYVQYTNARAQSILRKANQEVVVDDQLVVADDNAWDVLKMLSNFPAVIARASKEYEPSIVAKYALRLAKAFNKYYANSKILADDDQRNARLSLVKSVSIVLETALDLLGVQAPKEM

>fig|1664.9.peg.180

MVLVLKRQIKGIPVLEVVAENARHEPLPLVVYYHGWRSAKELVLTQARKLAQAGMRVVLPDALNHGERLQPVSEIPSWTFWQSIQTNLAEFSLIIDYWQQLHLIKDDLIGVGGVSMGGMTTAALLTKHPEIKVAACIMGSPAPLTYARLVRDNVRQHGLQQPADLGLLTSWLTAYDLTQQPNALANRPVLFWHGTEDERIPYNQMADFERQVKGQSYAQNVTFMTGQGERHLVQPALMTTITDFFATHLKIKSE

>fig|1664.9.peg.181

MMIDKTSAKPYYEQIILLIKERVLQGILKPGEQIPSVREMARQLMMNPNTVSKAYKLLEVQGIIVTVKGRGTYIAEHQATDRDNGKIAQLKAQLQELVLEAVYLNVSEAEMSEWLTAQFERGPKDADN

>fig|1664.9.peg.184

MQSKSKQLRWYNVALIAFVAVWGLGNVVNNYAQQGLSVITSWILIMLLYFVPYALIVGQLGSTFKDANGGVSSWIKATSTKRLAYYAAWTYWVVHIPYLAQKPQGILIALSWLFKGNGNFVNTISSMAVSVICLVLFLLFLWLSSRGIATLNRIGSIAGTAMFVMSILFIILAVSAPFMTKGAHIATPDMGNIKTYIPKFDVNYFTTISMLVFAVGGAEKISPYVNNTKNAAKEFPKGMLVLAGMVAFCAILGSFGMGMLFDSNHIPTDLMANGAYEAFHRLGVFYHVGPLFVILYALANTLAQVSALAFSIDAPLKILLGDADSNFIPKGLSKLNKKGTPTKGYMLTGVLVGLLIIVPALGIGNMNELYNWLLNLNSVVMPLRYLWVFLAYILLNKQLKKFTSEYHFTKNPKIGLIVGSWCFFFTAFACLMGMIPKLSYAANPSTWWFQLSLNIITPIIFIALGMILPAIARRQKA

>fig|1664.9.peg.185

MENGRIRRASRFNKTRFVFILKGLLVGGVTGLVVSLFRLAIEKGLALTIKLYQSLHSQPLNWLWIIGANLLIGLVVAQLLKKEPNISGSGIPQVEGQLEGEFELAWWSILWRKFVGGILSIAPGLFLGREGPSIQLGAAVGQGVADRLDDHGADRRILIAGGAAAGLSAAFNAPIAGTFFVLEEIYHNFSPLVWLTALTSAIGANFISLNFFGLTPTLHITYTHNLPINQYWHLILLGIVLGLLGYVYQKTLLWLPQFYKRLPVPKYYWGLLPLVLVLPIGYFWPTVLGGGNGLITQLGQQVPTLTTVALLLVLRFVFSMISYGSGLPGGIFLPILSLGALIGAVYGLVMVQLGWLAPVYVPNLIIFAMGGYFAGIGKAPFTAILLVTEMVGTLTHLMPLAILSLVAYVVVDLMGGAPIYASLLQRLIGQPKTDMAQFKDRLEVPIFAGAPLEDHQVRDVAWPETCLLIAVRRGETELIPHGDTLIRAGDTLVILTDHHLRATVRRQIEAAAQTLKKADENLVK

>fig|1664.9.peg.186

MENLILNLLLIDSILIVIAVMMQPSKQQDALSALSGGSGDLFGKQKARGFEAFMQKVTVVLGTLFFVFAIALVYLSSH

>fig|1664.9.peg.187

MQYRLPQPYYYDSGPVGVVLLHAYTGSANDVHMMGRFLEKQQISVLAPHFTGHATFEPLDILEEGSVQAWWADTTAAIQKLQVAAKKPLFVFGLSLGGLFAMRAIETMPAVIGGGIFSAPVLEGPTDKLEPLFLGYAQRLYQMAEKTAADQAVQLAKIKQQLPQQLQAIADFSALVTADLDQIGDKPVFIGQGGQDQVIDPNGASVLKQRLEAQQTPVDYHWYDNAGHVITVDRAHHQLETDVEAFIKRYEK

>fig|1664.9.peg.188

MTQVDQMKAAILAIFAADETQQLNVSEISERIGVAGSQGFKDLVKVLAELEGDKHLLMDDAGKFRLPASQRLVEGVFHANDRGFGFVSMVDKEPSDPDIFIAPPNTNFAMNGDTVEVAIIKEAEENSRRGPEGKVAKVITRGIEEIVGEFMPYSDIQKEKTGLIGYIQSHAKKLSSYLIYLTDNGLHPQKGDMVQVDITSYPSIDTPGQMRGIAKQVLGNKNDPGVDVLSIVYQHDIKTDFPEEVRLQSEAIPDTVLETDKFGRKDLTDQPVVTIDGDDSKDFDDAVNVRQLDNGHFYLGVHIADVSYYVTEGSPLDAEALERGTSTYLTDRVIPMLPFRLSNGICSLNPDVERLALTCEMEIDEHGNVVDHNIFPSVIKSTARMTYNNVNKILTDQDADLRDQYARLVPMFETMAELHEILLKKRHNRGAIDFEEDEAKIIVDENGKAIDIELRQRGLSERMIESFMLAANETVAEHYSTANAPFLYRIHETPDTDKMKNFFEFITAYGIQVQGSSKKVTPMMLQEVLTQIEGQPEQPIITTMLLRSMQQAHYSDESLGHFGLAAEFYTHFTSPIRRYPDLMVHRLIHNYATNGMTIEEKEKWAPKLQPIAEQTSLEERRSIDTEREVVDLKKAEYMLDKVGNEYDAVISSVTSFGMFIALPSTVEGLVHISQMKDDYYSFVESQLALVGERTHKMFRIGQPVRVKVANADLDAHSVDFELLASEEVPLASAEILSKIEARPKRPTRPRDHEKGGQRRGDQKKNSHPRRPVAKKDSQKRTFKK

>fig|1664.9.peg.189

MAKKHPQKPANLIAQNKKAGHDYNILETFEAGLVLTGTEIKSVRKGKISLRDGFARVRRGEAYLENVHISPYEQGNQFNHDPLRNRKLLLHKKEIAKIGALTKDKGITIVPLRVYLKHGFAKVLIGVGEGKREYDKRETLKRKEQDREMARALRKR

>fig|1664.9.peg.190

MQFKKIGLMILTGLMIIVTTAGPVSAASLSELKQQEDQTQKSIDTINGKIQSTLTKVNTKYQEVDQLKTKITENEEHINKAETILAAQKKELAERKDYAKAHLQALQKSEDNRDVLNTLLSADSLSDLFSRVYTLTILQDADNQNMTDLATSYQKMVTIQKHLEESKAALDKQKKDLDQQTADLQKQVDQMKKDLSQSQKQLADISKKKIEEQKREAEEQAKAAAKAKNEAAAKAASQKLSAVNNQQQAALAAAAKGKPGQTIEVQATAYSTAEPGLSRYGHWFGENPNCIAVDPKVIPLNSLVLVPGYGYAIAGDTGGAIKGHIIDVHFPSVAQCVSWGRRHIAITIIK

>fig|1664.9.peg.193

MGSQKSLLLIDGNSVAFRAFYAMHQVLDSFTNQDGLHTNAIYAFKNMLDIILKRFEPTHVLVAFDAGKTTFRTAQFADYKGGRSKTPPELTEQFPYIKELLHAYGIQTAELANYEADDIIGTLACQAEQDGFVVNIVTGDRDLTQLTTDKTTVNITVKGVNDTEAYTPAHVKEKLGLAPSQIVDLKGLMGDTSDNYPGVTKVGEKTALKLLTEFGSIEGVYENVDAMKKSKMKEHLIEDRDQAFMSKKLATILRDAPIETTLADITYEGPNIDTLIAFYQEMNFQNFLKQLNVTPEAVEPAENYQYTVLTLDNLETVQAIKTPVAFGVEMLTDNYHTADLIGFYIGAPDHWYVSDDVALLAEPVLKDWLEDASHPKAVFDVKRTQVAANRLGVHLAGVNFDLLLASYLLNTTNNSNDLGTVANLHEYQNVQTDEAVYGKGAKRAVPTEPALLYQHLVQKAAAIYHLQPQLEEQLKEHQQFDLFTDMELPLALVLAQMEITGIRVDASRLQQMGSEFSQTLKILEEKVYAEAGETFNINSPKQLGVILFEKMGLPVIKKTKTGYSTAVDVLEQLRKDAPIVQTILDYRQIAKIQSTYVEGLLKSIHQDNKVHTRYLQTLTQTGRLSSVDPNLQNIPVRLAEGRKIRQAFVPSHPDWQMFASDYSQIELRVLAHISGDANMQEAFKEDRDIHANTAMKIFGLNSADEVTPDMRRQAKATNFGIVYGISDYGLSQNIGISRQEAKRFIDAYFEQYPGVQQFMVDIVKKAREEGYVETLFHRRRYLPDIHAKNFNLRSFAERTAMNTPIQGSAADIIKVAMILMQQELKKANLQARMLLQVHDELIFEAPTEEIPILQKLVPSVMDSAVKLAVPLKVESGFGPTWYDIKK

>fig|1664.9.peg.194

MPELPEVENVRRGLETLAVGKTVSAIDIRWSKIIVNPDEVFTAGLVGQQITAVDRRGKYLLIRFGEQLTVVSHLRMEGKYEVVAKEAPISKHTHVIFEFTDGQQMRYLDTRKFGRMQLIETGQENTVAGLKDLGPEPTPTTFLKADFYQRLQKHHKAIKPLLLDQKVVTGLGNIYVDETLWLSHIHPETPANDLTRAETDRLHDEIIAELELAINHGGTTVNTFLNATGHAGAFQEMLRVYGKKGVPCERCGTPIEKIKVAQRGTHFCPKCQIKRNAK

>fig|1664.9.peg.195

MTYFLGLTGGIATGKTTVSQMLAQQGIPIIDGDQVAHQVLANNQSVQAQIQATFGKQLVQDGQVDRAALGKLVFGNQAALAQLNAITAPVIRETIMTEMAQAKAHQVPLVVLDLPLLYEQHYETVCDGVLVVYLPVEKQLARLMARNQLSREDALKRINSQASLAEKRDRADFVIDNQGSLDQLKAQLKTVLEGVCHKSGMS

>fig|1664.9.peg.196

MLCPHCHQNSSRVIDSRPTDEGRVIRRRRECENCQFRFTTFERVEQTPLLVIKKNGTREEFNRDKLLRGLIRAAEKRPVTMEQMTEIVDEVENKIRALGENEVSSQAVGEYVMAVLPGVDEIAYIRFASVYRQFKDMNVFMAELQEMMKKEKAKDQD

>fig|1664.9.peg.197

MSDAFDYLSPKDGFMVTKANYLSDFDQTVVTYLYQPLMGAVAYSLYLLLWSQSTQAKSQTTFQTHATLLNLLGVDLPAFYDARNKLEALGLMRTYQKNNNTGRNLVYELYAPMQPHIFFNDDLLSVILYESVGEKRFIELREHFKLQPVRSKEYEEVTKNFLTVFHVQNDHLTTAPTAVAEAQSTYQQKKAQKPSFTSVELRTFDWALLQEVVAQNQIDTDEILRNEQRLFNLHYFYGLDEMELGRLIGLTMDVADSRVNMDALEQNVLNNYTRRRSVQNNSEQPAKKPLETLQKEWQKAGFNEKEMQWLTESQQYLPVDYLEYLKQKNHGFVAKNEIRALRDLQNRYIFNNDVLNILVVYIISQYEGLTQALLDRIANQWAQQGVKTPADAILQIRDFQTKQETKQTKRQPRYQAKTKQETVPKWAKSDYQAPQTTSSKADQQEIDQLLKKIREGRES

>fig|1664.9.peg.198

MENMGKDLTDYMNRQRLNQRFNKLVEEALQDPDVQAFIQANQAALSQETVARSAAKIYEYVNEKKKILNGETTLAPGYAPKLVLSNHFIDVSYEPTADLVQKRAQAELKARVKSINMPKDIVEASLAQYDASERQVPLIEALKFVEAYRQAPTDFHQGLYLSGQFGVGKTYLLGATANELALQGFETTLIHFPTFAVEMKNAIGQNNVLEKVNQIKKAPILMIDDIGADALSAWVRDEVLGIILQYRMQEQLATFFSSNFTMNELEKHLTTSQRGDEEPVKAGRIMQRVRYLAREVEVSGQNRRLQSN

>fig|1664.9.peg.199

MAEIQLTFPDGAQKTFEQATSLLDVAKSISTSLAKKAIAGKFNGEVVDLQQPLLEDGAIEIITKDDQAVNLTATWHTAAFVLAATLSQHYPEMQFGEVTATEDGFYYDTDNEAGQVAVTDLPEIKTEMAKLIQSGEAISRVSVSKDDAKKLFAGQTYKLALLDEVEADQVLVYQLGDYSDFSLAPMLGKVSDVKFFELLSVAGAYWQGKSSNQMLQRIYGTAYPKQEELDADLKRRQEAKERDHRVIGNQLDLFFVDPKVGAGLPYWLPNGATIRRSIERYIIDKEVANGYEHVYTPILANLDLYKQSGHWDHYREDMFPPMDMGDGEMLELRPMNCPSHIQVYKHHPRSYRELPIRIAELGMMHRYEKSGALSGLQRVREMTLNDGHTFVRPDQIQDEFKSILGLMIDVYADFNINDYTFRLSYRDPANTEKYFDDDEMWNKAQAMLKGAMDDLGLDYVEAEGEAAFYGPKLDVQTKTAMGNEETLSTIQLDFMLPERFDLHYVGEDGEMHRPVMIHRGLVSTMERFTAYLTEIYKGAFPTWLAPTQAVIIPVKNDLHYDYAKNIKDEMIKRGLRVRIDDRNEKMGYKIREAQTSKIPYTLVVGDQELAQATVSVRKYGEENAVEEASDMFINAIVAEVGNYSRDGKQHTKKINL

>fig|1664.9.peg.200

MATEKYMAGSAEKALDIINLYELEEAAGKVVPKGGFGYIYSGAGDLYTINENITAFNHKYIAPRVLQDIENPDTSTEIFGDHLTAPIIMAPVAAHKLVNTQGEVATAKGVAEYGSLLTMSSFASASIDDMATAADGAPQWFQLYMSKDNDINRKILDEAMSHNVKAIVLTADATVGGNRETDKRNHFTFPVGLPIVEAYQTGVGQTMDAVYKSAKQRLNPKDVAFISEYTHLPVFVKGIQTAEDVEIALQAGAKGIWVSNHGGRQLDGGPAAFDSLHVVAKAVNKRVPIVFDSGVRRGQHVFKALAEGADIVAIGRPVIYGLALGGSIGVKNVFEYLQKELELVMQLAGTHNIDEVKATQLIDNHYGN

>fig|1664.9.peg.201

MVNDGIRARELRLIAQDGEQLGIKSTQDAIALAEQANMDVVLVSPNAKPPVARIMDYGKYRFELQKKQRDARKKQKTISLKEVRLSPTIEQNDFNTKLNNARKFLGKGDKVKASIRFKGRAITHKEIGQRVLERLAKETEDIAKVTARPKMDGRSMFLMLEPLKDQK

>fig|1664.9.peg.202

MPKFKTHRASAKRFKRTGNGGLKRSHAYTSHRFHGKTKKQRRQLRKSGMVSNSDMRRIRQMLSGLK

>fig|1664.9.peg.203

MPRVKGGTVTRKRRKKILKLAKGYRGAKHLLFKSANTQVMVSYRYAFRDRRQRKRDFRKLWIARINAAARMNDMSYSQLMHGLRVAEIDMNRKMLADLAVNDAAAFTSVVEAAKAALNK

>fig|1664.9.peg.204

MEKFGFNHFGGPDVFETLNQSQPVPKANQVLIKVLAFGLNPYDVALRTGSQQTVRPLPMPIVPGTDVVGEIVMLGADVTDYRIGELVINHRPLGGYSEFVTASTNKIIRKPVSLSIAEAAGLASAGITAYNTLMHFTNFKHGDTIAIMGASGAVGSLLTQLSLAAHLNVIAIAHSSNLDYLRSFGVQETIAYDQSGDLSHFKQTAKIAVNLSRDPKSTELLNELVQPDGFFASTTIMPSEAEQMAGVTYQSVYPRRDYPDLTALQDLSELIDQGLLHVTVLEELPFTLEGVIKGHQLLESHHAPGKIIVSR

>fig|1664.9.peg.205

MLKNFKPTWMVNAIYDLTPEQLKAQGIKAVLTDLDNTLIAWNNPNGTPQLKEWLAQMQAAGITVMVVSNNNQQRVQHALAELQLPFIARANKPLATGIVKAKKQLGLSRHEVVMVGDQLITDMHAGNIAGVRTILVKPIIDSDAWNTSINRFFEKYIMQQLLKKHADLTWREDLND

>fig|1664.9.peg.206

MTEEQQQTEEALFCIGCGAQIQTTDKEALGYTPNSALKKGLETGQLYCQRCFRLRHYNEISDIKLNDDDFLALLNQIGDSDALIVNVVDIFDFNGSLIPGLHRFIGDNPVILVGNKVDILPTALNPNRVINWIQKEAKEHGLHPIETMLVSAKKGNSVDDLLAVIERERKGRDVYVVGVTNVGKSTLINRIIKQTGILDSDVITMSQFPGTTLDRIDIPLYEDDHALIDTPGIIHAQQMAHYLHDKDLRLIAPQKQIKPKVYQLNEQQTLFLGGLARFDYLQGPRHGFVGYFDNELYIHRTKLENADNFYVRQLGDLLQPPHPEDLADFPDLQRYEFSTKEKSDLVFAGLGWITLPENVVVAGYAPKGVSVMIRKALI

>fig|1664.9.peg.207

MLTGKQKHYLRGLAQTTKPLFQLGKNGLSEAYFEQIEEAIEKRELIKINLLQTTEVTVDELANFLKQHDNRIEIAQKIGRTLVIYKASRKEKYQKISTIVTKLGR

>fig|1664.9.peg.208

MQNATLKKVHGVSESKPSVLVVEDKQRQQVGIMGGTFNPPHLGHLIMAEQVGTQLGLDKVLFMPDATPPHVDTKKTLPAKHRVEMVKRAIADNPLFELSMAEIERGGVSYTYDTIVALKKQYPNTDFYFIIGGDMVDYLPTWHRIDDLVQLVQFVGIQRTGYSRDTPYPVLWVDAPLVDISSTQIRNKVQQSCSIRYLVPTKVADYIEEEGLYRE

>fig|1664.9.peg.209

MSDFTYQENIVPYSRAELAQKVAANLSESRFQHCLRVEETAIKLAELNHADVERAAIAGLVHDYAKQLPDEVFIEAIHEAGFDPELLQYGNAIWHGVVGAYFIERDLGIHDETILNAVRRHTIGAPEMTVIDQILFVADFIEPGRDFPGVVQARETAFADLAAGVRFEILNTLTFLIEGQKKIYPKTIDTYNAWVPTQAH

>fig|1664.9.peg.210

MKSLDLLKIAVKAADGKRAEDIVALNMEGISLLADYFVMMTGSSERQLDAIVDAIEDAEEEAGLMVKKIEGKKGSRWILMDFGDLVINVFMPEERALYNLEKLWDEAPMVDVSEWLTEE

>fig|1664.9.peg.211

MIYQSFATVYDRLMDHELYGQWRDYTLQTLQNQHRVLELACGAGDLAILLKQAGLDVVGLDLSTEMLTIASEKATEAGVSFPLLQGDMLDLSEVGTFDAITCFDDSICYMQDLDQVQQVFKQVYQQLAEGGQFLFDAHSLYQMDVLFPGFMYNDRTEETAFMWTSYEGEVPHSVEHDLTFFVWDEQLGGYQELNELHHERTYPMADFVAALETVGFKQIKVSADFGQEAVQDDSVRWFFSAVK

>fig|1664.9.peg.212

MRITGIIAEYNPLHNGHIYQLQQARQQSQADLVIVCMSGNYVQRGQAALLDKWTRAELALQNGADMVVELPFVSAVQPADRFATGAVQLLAALGCQTIAFGSEQPTFDYQQAGQQIIDLPEQSAAFVDYQKTYATQLNEFYQEQLSLQIDQPNHLLGISYAVANARLAQPLNLLAIKREQAQHGDQTVSTAPYASASAIRQVVTQGTALAALQHVVPAGTLAALADSTLLTEDQLWPLLKYRIESLSLAQLRQVYQMSEGLEYRFKKVIHKANSYTELLQALKTKRYTYARLQRVCLYVLLNIQPADIQQGLQRTYIRLLGFNEIGQQHLHQLKKTTDLPIISKVSAKMGAETGLLGLEVRVDRLCEQFGWQSQNFARQPIFYHGKDEAHC

>fig|1664.9.peg.213

MLKWSVQALQKHREHPLAFEETVDLEKQLHERENDVLAATPFVISGQLTVDRRAIIADLHVVGQLTVPSTRSLNPVALPLDFTFTEIYVDPTDEELSRFPDEDMLFPLEGEMLDMQVAVEDHILLHIPSHILTPEEENEDVMPTGQAWSVISEEEYAKQQAEEAEAPNPELAKLKALLEAQENDAD

>fig|1664.9.peg.214

MSQPQIGVIGMAVMGKNLALNIESRGYTVAIYNRTGAKTEAVIQDHSEKKLVASYTIEDFVKSLEKPRRIILMVKAGAGTDAVINELLPLLDKGDILIDGGNTFFGDTIRRNAELDKSGINFIGMGVSGGELGALEGPSMMPGGQKEAYDLVAPILEQMSAKAEDGQPCVTYIGPNGAGHYVKMVHNGIEYGDMELISESYNLLRNVVGLSVEEIADVFKEWNHGELDSYLMEITADILTRKDDLGTDKPIVDVILDAAGNKGTGKWSSQSALELGVPQSLITESVYARYISAMKDERVAASKILPAPTVAKLTEDKKELIEKIRQALYFSKIMSYAQGFEQMRVASDQYDWSLNYGEIAKIWRAGCIIRARFLQKITDAFDKKADLNNLLLDDYFLDITKKYQGAVRDIAALAIQQGVPAPGLTAAITYFDSYRSEVLPANLIQAQRDYFGAHTYERTDREGIFHYTWYKEQ

>fig|1664.9.peg.215

MSRILIIEDEKNLARFVELELKHEGYGTEVHFNGRTGLEAALNEDWDSILLDLMLPELNGLEVCRRVRQVKNTPIIMMTARDSVIDRVSGLDHGADDYIVKPFAIEELLARLRALLRRIDIEGEHNAAKQTTVTYRDLTVEKENRIVRRGDDIIELTKREYELLLTLMENVNVVLARDVLLSKVWGYDSDVETNVVDVYIRYLRNKIDRTGEQSYIQTVRGTGYVMRS

>fig|1664.9.peg.216

MKWAAAVGLAIFITFAIFSIVIFSSISNILLHKEVDNVHDTVTVIQQRLGRPEKTLTSEFVAEQLDPAPRDSKAIYQDSVLMRLAQQDTTVKVYDQNRQGLFASRGRATSFPKQDTDVLKREHIFGKIHLMGIRPIKTANTNRTIGYVEVTNNLRDYNHAMLELRIVLVVVVLFAMLFSAVIGYLLANRFLQPIEAMKQTIEAINVEPNSNVRIPETRRSQDELSDLVIAFNEMLNRIQHYFEQQDQFVSDVSHELRTPVAIIEGHLQLLNRWGKDDPEVLSESLAASLQEILRMKNLIQEMLDLMRADQAVTQGTTEVTDVGKVVLQNYHNFEMLYPEFTFILDDDLHGQALVNINRNHLEQIMIILLDNAIKYSTTRKEIHIAVANDERYAQVAVQDFGEGISEEDKKKVFHRFYRVDKARSREKGGNGLGLAIAQQLVEGYDGNVSLESSVGHGSVFRIELPLVRETKAAPEEDAD

>fig|1664.9.peg.217

MKNSKRIATLAMLAMTVTLFLSGCMPQKMSSHPKVPTGFIYGSSYKYIAVPLQHIMERIADFFGGINGYGWAIIIITFVVRMILLPLMLNQSNKMTAQQEKTRRLKPQLDIVQAQQKVATTPEEKAELSQLMMKVYKENDSSMMPSLGCLTLLIQLPIFSGLYQAIQYSPEISSSHFFGIGLGQPNIIITIIATLFYVGQSALSLVGMPAEQKKQMQTTVLMSPAITFFISLFAPAGLALYFLAGGMIMIIQQMITTFIIMPRVKKRIDQEIKEKPIVTVVTKDMFTKKAAAKATETPVTPETTHTNKATMPSETGKRNSGKQRHKPQA

>fig|1664.9.peg.218

MQKAVQLDVFGRVQGVGFRWTTKLVADRLGITGTVSNQPDGSVKIIAMGPDAILEQFIGAVKASPTPSGRVDRVVQTPLQDVSACHKFSVVG

>fig|1664.9.peg.219

MEYIQSNQNTKIKAAKKLTVKKNQRKENTYLLEGWHLVQEAIQNKAVIRQVFATEKYVDERALRGLYDDTFEIAPEVAQHLSETKAPQGIFAVVEMSETPMPEKLTGRYLLLDAVQDPGNIGTMIRTADAAGFAGVVLGNGSVDLYNPKLLRSMQGSHFHLPIYQGNLIDWMKKFNSQDVPVYGTELNPEAISYQDVPKTADMALILGNEGNGVAKDVLAATTQNLYIPIPGNAESLNVAVAAGILMFKLIEG

>fig|1664.9.peg.220

MNATAKWYQDADYMALIADLLEKEEVQKLANYTQHHHSDRLEHSLSVSYRSYELAKKWHLNVRSVARAGLLHDLFYYDWRDTKFDLGTHAYVHPRIALRNAEKLTELSPMEKDIIVKHMWGATVSHPKYRESYIVSLVDDYCAITEFCSPLWSKFKQKVRHQSAATQTNK

>fig|1664.9.peg.221

MMSIVKENANSDCQETFQLCPKFEKTFSILGKKWNGLIIDVLLEGGAQRFKHLVHSVPKCSDRVLVERLKELEQAGIVARITSADSSLIEYQLTEKGESLAPVMDAVHNWSDQWCDTND

>fig|1664.9.peg.222

MSLKEQLEHLQQKSLQDIQKVVDLDALNQIRVEVLGKKGPITEVLRGMRDLSNEERPKVGAFANEIKSDLAQAIEARKAELEAKKEAAQLAHETIDVTLPGQPVKKGTPHVLTQVIDDLEDLFIGMGYQVVAGFEVEDEKHNFEMLNMPADHPARDMQDTFYITKDTLMRTHMSPNEARDLESHDFANGPIKMISPGRVYRRDTDDATHSHQFYQMEGQVIDKNITMADLKGTLEYTIHHIFGEDRDLRFRPSYFPFTEPSVEVDISCFRCNGEGCNVCKQTGWIEVLGAGMTHPNVLKAGGVDPEVYGGFAFGLGIDRFAMLKYGVDDIRNFYLNDVRFLNQFTQEV

>fig|1664.9.peg.223

MKVSYNWLKDYLDLTTAPEALAEKITRTGIEVADVAQMSAGLKKIVVGHVLSCEPHPDSDHLHVCEVDVGEEEPFQIVCGAPNVAAGQYVIVALPNSRIADNVKIKKGKMRGVVSMGMICGLQEIGFADSVVPKEYVDGIFVFPEAIAPGTEVYEALGMTDYIIDLDLTANRADALGIHGVAHEVAAIESLTPHFEDVAVSESDVQTKDQVSAQVVDDQLAPSYHLRMLQNVTVQPSPLWLQTRLWNAGIRPINNLVDVTNYMMLTYGQPLHAFDADTLTSDHKQIEVRLAKTGEKLTTLDEAEHDLTNEDIVITDGNQPIALAGVMGGFNSEITANTKNVIIEAAIFAPTAVRKTAQRHNLRSDASSRFEKGVNVADVQVALDDAAAMMAELGTGQVTAGVVSPTNLAPQPKVIQIDSARVNRVLGTDMSVQTMIDLLERFGFEVANNADQLTVTIPARRWDIEIQADVIEEIARLYGYDNLPSTLPTGDMTTGALTTEQKALRRTRHTLEGAGLTQAISYALTTEEKAGQFTLAAKQTATVLDWPMTQDHAYLRMNLVTGLLDDAAYNVARKQTDLALYEQGRVFLKHADQARPNEVEYVAGLMSGNRQVKSWQEAAAPVDFYTIKGVVDTLMASYNLQAAVAYQATDAYPEMHPGRTAAIYVGETFVGIVGQIHPKIAKATHLKETYIFELDLAKILELQRQTIIAKPAPKFPEVTRDIALQVPEAITNADLVNAIKEKGGRYLVSVSLFDVYAGSHIEAGEKSMAYTLTYLNEDATLTEEEVNAAFEKVVAHLVATFQAKVR

>fig|1664.9.peg.224

MKNADQKEPVNQKTAEERQYAERRAEKKAANKIVYWIISILIALVIIIGFMGYRFVQSSLKPYNTNAKQEITVQIPNGSTNKEIAAILQDKRLIRNASVFNYYVKTHNFTDFQAGYHVLKQSMSLDKIIANLQKEGTPTRPRDAKGKVLVKEGVTLEQIATAVGKETKFSKAAFMKQVQDKKFLASLEKKYPQLLSSTMAKKDVRYHLEGYLFPATYEVYKDSTLKELITEMVKTTDQNLQPYYATMKNKKLSVQQTLTLASLVEREGVTASDRQKIAGVFFNRLDIDMPIQSDISVMYALNTHKTHLYNKDTKVDSPYNLYVHSGYGPGPFNSPSLQSITAVLNPADRDQGYLYFVANLKTGKVYYSKTFEQHTDTTSSIED

>fig|1664.9.peg.225

MTSQKKAPIIIGVTGGSGSGKTTVSQAIAQKFANHSVMLLPQDAYYKHQDGSFEERQETNYDHPDAFDTDLLIEQATMLKNHQSIEQPVYDYKIHNRTDEVVHVEPQDVIILEGILVLADARLRDLMDIKVYVDTDDDIRLLRRMSRDMESRGRSFDDIVMQYLKTVKPMFHEFIEPTKRYADLIVPEGGNNRVAIDLLVTKIQSILNMND

>fig|1664.9.peg.226

MAQKKFPMTAEGKENLAKELENLKMVKRPEVIDRIKIARSFGDLSENSEYESAKDEQSSLETRIVEVENMLRFAEVIDVDNIAVDEIAVGKTVSFTDDEDGEEETYQIVGAAESDPFNGKISNDSPIAQALVGKHVGDKVVVQTPGGDMHVTITEVK

>fig|1664.9.peg.228

MPFFWRIFLIVEAIIFGTFIWQIITYPPYLVYIIIGILGLVWANKRRMANKRGFWTTFLTMMSSVAVAVPLLINFSLWLMLIVAIVFLAINHSHASHFNNRNLNNLFENAPWRKKNFMPINTTAPKPKQATVTRHNWFGHQTIGTNIFEWDDINFAVLAGDTIIDLGSTILPKDDNHIVIRKGFGKTRILVPVGTGIYLDHSAMLGTVTIDEQQYHLKNESLQLYSTNYDEESRHLKIITNVVIGDLEVILV

>fig|1664.9.peg.229

MSKLARSFVFISTGLLTLLFSVALFFAYFYSIQQDQWLLSLATVRFFYVPLIAYIIVAALIVATVVTSIFYLVLRQQVSHTESQLQYLVSGQYESPVFKQINADNDHFAMTIEQTVEQLRQKMIRLQRDIQSYSDRPPVIAGETKEEILAQERHRLARELHDSVSQQLFAATMMLSALSEVAQKQADNPQLLQSLTTVERVLNDAQSEMRALLLHLRPVTLEGKSLRDGIIQLLNELKTKIAVEITWDIDNVTLPSGIEDNLFRIVQELLSNTLRHAKAQEIEVYLKKIGPSVLLKVMDDGQGFDQKVAQKAGSYGLNNITERAQSVGGTAKIISLPNQGTSVEIRVPLLKERN

>fig|1664.9.peg.230

MIKVLIVDDHEMVRLGISTYLGVQDDLEVVDQAVNGQEGVEKALALRPDAILMDLVMPEMDGIEATKTILKAWPQAKIIILTSFIDDEKVYPAIEAGAASYILKTSTAEEIANTIRKTTAGQSVLEPEVTNKMMNRMTHQAETKLYEDLTNREREVLGLVAKGRSNQEIADELFITLKTVKTHVSNILAKLQVDDRTQAAIYAIKHQLAD

>fig|1664.9.peg.231

MQITVTDAASHWFQEELALKPGEAVRFFGKVYGQTAVHDGFSLGMERAEVSQPIGQVEKDGITYFASDSDAWFFADYDLQIDYDDQLDEPKYTFPKH

>fig|1664.9.peg.235

MVAYQALVLTHDNETVTAQYATKLTPDLPKEGVLIKVAYSDINYKDRLTMNAKSGVLKNYPATPGVDFVGTVVTSDNPFFEAGECVLCTGYGTGISIDGGYAQYVKVPSEWLLKCPIELTPQETMQLGTAGFTAAIALTKILKQPFTREHDTPLLITGATGGVGSYALTMLAQLGFTNITATTRDLSQADYLTQLGANQVIATDDLLNAPLKPLAHQSYTGVIDTLGGDVLAHILPYMAQGGLVAACGNAAGFKLATTVFPFILRGVTLVGIDSVNYPRKDRKEIWQLLATNFKPQIWPQIRTIAFSELATELDTPIKQTGRVVVKIDTEH

>fig|1664.9.peg.236

MKMQQPMVEFKNVIKKYGDNQILKGIDMALEKGKFYTLLGPSGCGKTTILRIIAGFTNASSGDVLFEGKRMNDLPANKRQVNTVFQDYALFPHLNVFDNVAFGLNLRKVKKSDVEKKVIEALKLVRLGGYEHREISELSGGQQQRVAIARALANEPKVLLLDEPLSALDMKLRKAMQYELRDLQQRLGITFLFVTHDQEEALAMSDEIFVMNEGNVLQSGTPVDIYDEPINHFVADFIGESNILPGKMVADYQVEIVGKVFECADAGMKPNEAVEIVLRPEDLDIVAVNQGQLVVTVDTQLFRGDYYEITAYDDDRNRWLIHSTNPAKDGQQVGLFFEPEDVHVMRFGESEEDFDARLESYEEED

>fig|1664.9.peg.237

MRKKQLIYYLPYALWLLLFVLAPVALIVYQSFFDINNHVTLQNYVTYFESGTYLKMTLNSLWYAFLITLITLLISYPTAYWLHQTKHKQLWLLLIILPTWINLLLKAYAFIGIFSQDGAINHFLGWFGIGPQQILFTNFSFLVVATYIEIPFMILPIFNSLEELNPSLVNASRDLGASNWQTFSKVVLPLTMAGVKAGVQAIFIPSLSLFMLTRLIGGNRVITLGTAIEEHFLTTMNWGMGSTIGVVLIVAMGLIMMLTGEKKTKKKVTMK

>fig|1664.9.peg.238

MKQAKIKWQHVWLIVVFILLYAPIFYLIFYSFSSGDNMNGFKSFTWQNYQDLFADTRMIEIVLETLLIAILSSLCATIIGTFGALGIYSTKQRRLRNIILSLNNILMVSPDVIIGASFLIFFTFLGLGLGFGSVLLSHIAFSIPIVVLMVLPKLNEMRPALVDAARDLGATDWQVLSKVILPYISSGIFAGFFMAFTYSLDDFAVTFFVTGNGFETLSVEIYSRARQGISLEINALSALMFVFSLLLVGGYYLLNQRNQAHKAKRKQKMGAPVK

>fig|1664.9.peg.239

MNKLGRLTLAILVVCAALWFGAVQLQKSQGYTKNNTLTLYNWGDYIDPSLIKKFEKQTGYHVSVETFDSNEAMYTKIKQGGTAYDLAVPSEYMIEKMRKANLLKPIDHSRLTGFQNIGKPYLNQPFDPGNKYSLPYFWGTLGIIYNDQLVDQPLTHWSDLWQAKYRGKIMVVDSARDMMAVGLIENGQAVNSTNPVALKAAKACLDQMAPNVKAIVADEIKMYMVQNEASVAVTWSGEAAEMLANNKHLHYVIPEEGSNVWFDNLVIPKTAKHTKAAYAFLNFMLEPKNAAQNAEYIGYSTPNMPAKALLPAATRNNPAYYPSQKVLNKLQVYSDLSPEMVGLYNDLFLEFKMQH

>fig|1664.9.peg.240

MSVDWAQLLTWRMLANILDIIVVWYFIYKLIMLIRGTKAVQLFKGIMVIAIIKIVSWYLGLRTVSYIMDQFISWIVIALIVIFQPEIRRGLEHIGRGSLLMRGNNPKLEKENNLIKALDKAIQYMSKRRIGALITIQMNTGLEDYIETGIDLDADVTGELLINIFIPNTPLHDGAVIIREDKIAVAAAYLPLSESNLIPKELGTRHRAAVGISEVTDALTIVVSEETGGVTLTKNNELMRDLTREGYLKYLNSHLITQEEDKVSLLQEFVDGFKKGGHK

>fig|1664.9.peg.241

MKNFIEGPWLYRILALFLSLLLFSYVNVDKINTTRQINNNNTQMLATRSETVKVPLQINADTDKYFITGYPQKVKVTLSGPNALITSAVNTLNFRVIGDLTKLGAGTHTVKLTQTGINKDIKVKIEPATIKVKIEHKLTKEFPVQVNVKEENLETGYSIGNPKISQDVVQVSGAKAAVNSVARVVATIDPDKGTKNDINEETLLQAVDRNGKTVNVLISPQTVNIKIAVQMNKKTVPLSLQKTGERSGYEYTLSSETEQVTVYGKKQTLATLTDLPVTIDMADITKDTTRTVTLTLPSGVYDSHPKQVKVNIKVTAPTGMTTSRTETTTQSSSESTTSMSQSQVQSSSSSTISESQTTDNNQSSESSSSSDSSSSTVNTNES

>fig|1664.9.peg.242

MTKYFGTDGVRGVANLELSPEMAFKLGRAGGYVLTKHADKNQRPKVLVARDTRISGQMLEQSLISGLLSVGIEVLSLGVITTPAVAYLIKVQEASAGIMISASHNPAIYNGIKFFGADGYKLPDATEEEIEAILDAPEDTLPRPEGEGLGTVEEYPEGALKYTQFLEHTIPDDLEGLKVAVDGANGSTSGLISRVFADLETDFTTTATHPNGLNINDGVGSTHPENLVKKVLESGADMGLAFDGDGDRCIAVDELGNIIDGDKIMFILGHYLSEKGRLKQDTIVTTVMSNIGLYKAIEANGMKSAQTDVGDRHVVEEMREKGFNLGGEQSGHIIIFDYHNTGDGMLTGIQLMNVMKQTGKKLSELAAPVQTYPQKLVNLTVKDKDAWLTNDKIQAAIDTVEKEMNGDGRVLVRPSGTEPLLRVMCEAATEEKVNQYADQIAAVVQSEIGE

>fig|1664.9.peg.244

MCGIVGVIGKEDTTEILLNGLEKLAYRGYDSAGIYVNSQSGRDYLVKEPGKISVLKAAVGPEVQGTIGVGHTRWATHGVASQANAHPHVSEDGRFYLVHNGVLTNYAELRATYLSQVNFASETDTEVAVQLIAYLAKTEQLSAKEAFRKALTLIEGSYAFVMIDRNDPETLYVAKNKSPLLIGVGADFNVVCSDALAMLNETKDFIEIHDREMVTLKKDKVEIEDLDGTFIHRDSYHVDMDASAAGKGTYPHYMLKEVDEQPAVMRRLLTEYVDDAGQMKIDPAILKAIDDADRIYIVAAGTSYHAGLIGQSLFEKLAEKPVEVHVASEFGYHMPILSKKPFFIFLTQSGETADSRQVLVQTNRLGYPSLTITNSQGSTLSREANYTLLLHAGPEIAVASTKAYTAQIAVELFLAVALGRHTAIKAAQDFDVNHELALVATGMETLLSEKQAIEDFTTMYLKTTRNAFYIGRGEDYALSLEAALKLKEISYIQAEGFAAGELKHGTIALIEDGTPVMAFLTDAKTIDHTRGNLQEVRARGAKTLTIVSEDHAQAGDQVVLPNVNPLLAPLLGIIPGQLIAYYASLQRGYDVDQPRNLAKSVTVE

>fig|1664.9.peg.245

MSINLNQIKQDLTQITNDVLTAANLRRGDIFVLGCSTSEVVGGHIGKASSREVGAMIIETLLEILKPLDIQLAVQGCEHINRSLVMERSVAEAHDFEIVSVVPAMHAGGACSVAAFEQYSDPVEVEHTVAHAGLDIGDTAIGMHVKFVQVPVRPSLDTLGAAHVTALRSRPKYVGGPRATYDI

>fig|1664.9.peg.246

MNFKQRQIPVIAILTALTVSLSMLFIIPVPATKGFVTLCEVGIYTTAIILKNPGGLAVGALSGLLIDLLSGYPEWCLFSLLIHGTQGFVVAYLTRNNEQPRRLLMPLLCGSVIMVVGYCLATSWLFGWTAGIASIFSNIVQNGFGILVTVPLVKQLYRVRPQLFSE

>fig|1664.9.peg.247

MSNILTIAGSDTLGGGGLQADLKTFEAFGHFGVSAVTCLATVMPDQTFQIQNIETETVLAQIEAILHYVPLTTIKIGLINDATTLEKIGHLLQKQPHLQIITDPVLAFKETTDKLQQHYLDSLKEFLLPQSLVVTPNLVEAQILSGLPQIQTRAELEQAATIIQSLGPQHVVIKGGQRFPDDDAIDLLHSGTTNHYLSAPRLTSATRNGAGCTLSAAITAEIASGHAVYPAVKAAKQFVYHGIQNGIQLRHGFGNVRQGGSYEF

>fig|1664.9.peg.248

MIEWARYLPTQKPKYLAIKKMVINLLEQGLLSPGEQLPAERWLATQLTVNRSTVTRAFDELTTDGILVRRVGSGTFVSQQAGEQQAQRRVNWHTYLTSQPFQVGQVKRQALQQLIAQQPADLIDVYSSDLPADLMPQFQFPPLTWQDFNQAQEAETRLGYAPLLTAINRLNQENQKLVLPEGQLLLTAGAQQSLFLVLQGLLVPGDAVAVESPSFFHEATLFEATGIRSFGAPLDAEGIQLAALEQLIVKHRIKLVILNPNYQNPTGQVMSLERRQAVIRLCQTYQVPIVEDDVFGWLRFETTPALPTLKQLDPENVIYMSSLSKIMGASTRIGWVAATPQVIDQLVRVQREMDLVPSIMSQVMAALAIQEPHFQTQLVTLRQQLQTRARQTQQLLAQALPDWQFSVAQGGFYIWGTRAVKPYALAPFVAAHVALAPGTLFGAHQAAFRINFARLNTSQLTALAQRLQQLLNEED

>fig|1664.9.peg.249

MTEKFIPEIYTPLKTSVVGPPAIARQVGGIKILGQKIKTLIFTTDVAIVNNTNADAVLAVYPFTPHPAVMNAISNVATIPVLNGIGGGLTAGSRSAYMGLLAEAAGSMGVVVNAPTSVETIKMINKVVDVPIVGTVVSERQNYAAQIEAGVSILNVSGAAKTPAIIKAIRQKYPQIPIIATGGHDEETILSTIEAGANALTYTPPTNGELFAKKMADYRADLD

>fig|1664.9.peg.252

MPTGVIINSLSVLFGGIIGGLLGDKLSARFKTEITLIFGVCSMGMGINAIVVMRNMPAVILAIVLGTGIGLAVHFGHWIDRGAILMEKPISKLFPVRKTTLTDAEFISALVTVIVLFCAGGTGIYGTLDAGMTGNSTILISKSVLDFFTAAIFACNLGYVVSAIAIPQFIIFYIIFLLAKLIFPLTTPTMIADFKACGGFLMLATGFRMVKIKEFPIADMIPAMILVMPLSWLWTTVIMPLLS

>fig|1664.9.peg.253

MPDLYIKHQASALKGVSTIMNDHQRPLFLLVGSRGLRQDGFSLYRLSGELLGEIKQKSLGFSPSFELFQNGQSIGSLQKIWGVWHEFVYVKNLNWLIMGDLIQNQYRIIYHTQTIMQTNLILTSAGPTFKLTIANEADQVSCILIAAVLNHWVYTQPKAFIRKLAPNTLRFS

>fig|1664.9.peg.254

MISLIASDMDGTLLNDKMQVSDGNINAIKAAQDAGIEFIVATGRSRNEALPLMAEGGIKPALITMNGAQVFDQLGNEVINIPIEPATVVAVTDELRASGYYFELMTTAGNYSDSQVNRIQNVADLLVDLNPDTSYKIAVALAAARLELMNINYVDDHYAGIIADPTIKIVKIIAFSTKGLADLEPVKVFCEERGDLIVTSSSSNNIEINHINAQKGIALAAYAKEKGIPMSETMAIGDNLNDYSMIEAAGIGVAMANAVPTITDLANYHTAKNTQDGVAQAIYHAIDLNQAAEKK

>fig|1664.9.peg.255

MKTIIHNDWQTVLEPEFAKPYYGQLHQFLKQEYATTTVYPEMHHIFQAFEWTPFEQVKVVILGQDPYHGPHQAHGCSFSVLPGVKVPPSLKNIYKELQTDVGFNPVNHGYLKAWADQGVFLLNTVLTVRAGEANSHRQQGWEQLTDAAIAALSQRGHVVFILWGNAAKAKRALIDESQNAVLTAVHPSPLAAYHGFFGSKPFSQTNQQLKQWAMTPINWQLPQDVSEQK

>fig|1664.9.peg.256

MDLFESLKAKITSHDFKIVFPEGTEPRIIGAAARLNADRILKPILIGDAADIQSIASAKGFNLTGITIIDPQAYPQADFDTMVAAFVERRKGKATAEQAQTILQDPNYFGTMMVYLDLADGMVSGAVHSTGDTVRPALQIIKTKPGIQRTSGAFIMQRGRDNERYLFSDCAININPNAQELAEIAVASARTAELFDIDPKVALLSFSTMGSARGEEVTKVQEATKIAQELAPDLDIDGELQFDAAYVPVVANQKAPDSKVAGQANVFVFPELQSGNIGYKIAQRFGNFEAIGPILQGLNKPISDLSRGSNEEDVYKLAIITAAQALQN

>fig|1664.9.peg.257

MLELQTTQPEETITIAKKLGRQLQAGDVLLLDGDLGAGKTTFTKGLAEGLDIKRYIKSPTFTLIREYPDGRIPLYHMDVYRLEETGASDLGLEEYFDGDGVSVIEWSQFIADELPSDYLTIHFNKNDDDDQMRTLVFEPNGARYTALVEAIFDN

>fig|1664.9.peg.258

MQVTDARAYLCFLNQVVQETPFLPIAAENLAMSEAFCELTLRAVLDSSTDALWVTVDGAKIVGAARLVSEPEPGLQHIGEISVAVLKAYWRQKIGTQLLDALLQAISASGQPRRIVLTVQARNQGAIQLYQQFGFQIEAKLEAGYYDPAVGMLPVLQMVRLINIE

>fig|1664.9.peg.259

MIQQRRKGIYLAILGSIFWGIQGPVSQWLFTDTPIAPEWLMGVKMGLSGLFLLGYAFSKQPKTVTAVWRQPRDAGRLLAFAILGLSAVQYAYFLTIQASNAPTTTILQQLGTVMIIIISLVIYHKTPSRTELIAVIVALLGTWLLVTKGQLTQLSISGAALGLGLCLGFSGALNTLIPGKLFQKYDTLVIVAWAMLIGGVLFNVIHPFWVDAPPMNMPTIISVLFIAIFGTALANLCFLGSLNYITPTTAGLLNTFEPLAATIGTVLFLKTSFNHWEVIGGLLVISTVFILSFGNRRGISKK

>fig|1664.9.peg.260

MNFVAMDFETANGQRDSACSIALVVVRNDQIVDQFYTLIKPETYFSSRNSQIHGIYEEDVAQAPKFDQIWPHIAPFFTPQKLVVAHNATFDIGVLRSTLQHYDIPEPHYLALDTLTTSRRLYPQFENHKLNTLCANLEIPLENHHNALADSIACAKILIKEAQEFGTDPLKQVTKIIG

>fig|1664.9.peg.261

MKFISWNVNGLRAVLKKDFMTTFEQLDADFFCLQETKMQAGQVELDLPGYHQYFNYAEKKGYSGTAIFTKHEPLNATYGIQQPEHDHEGRVITLEYPQFFLITCYTPNSQNQLKRLDYRMTWENAFRTYVQQLGTQKPVIFCGDLNVAHEAIDLKNDRTNHKSAGFSDEERAQMTTLLQSGFTDTFRYFYPDQTDIYSWWSYRFHARDNNAGWRIDYFITSDELQPKLMDAKIHTEIYGSDHCPVELDTQDLFA

>fig|1664.9.peg.262

MLSQINAFLEANQTINVKTNEPLSKYTFTKTGGPADLLALPTSVPEVRQLLVAAKQNQLPITVIGNASNLIVRDDGISGLVIILTAMDQIDVQGTTVVAQAGAGIIQTSEAAYSGSLTGLEFAAGIPGSVGGAVFMNAGAYGGEISDVLTSAEILTQDNEVETLTNAELNFSYRHSLIQENGSIVLSARFEMAKGVAPTIREKMDELNALRAAKQPLEYPSCGSVFKRPVGHFVGPLIQKAGLQGHQIGGAQVSEKHAGFIVNRGGATATDYLTLIAYIQETIWHKFEVRLEPEVRIIGKESE

>fig|1664.9.peg.263

MAIIEAVILLVMLLIVANIIGHYVPRLPVSLIEIGLGLLVALLFSVEIPLRTDWFMLVFVAPLLFNDGRRFPKRELWALRGPIIGNAIFLVFVTTLIGGLAIHLLVPKLPLAASFALAAILSPTDPMAVQSISEQADLPPRVLHLVSGESLINDASGLISFKYGIAAAVTGYFSIQQAAGDFLYISIVGAIAGMLLMYAIHFIRLFMLQQGIEDVVFHTVLQLITPFVIYLVVEDLLHASGVIAVVVAGIISHASRNIYLDYLPELKLVTEQTWSIGVYLLNGLVFLILGIELPVAMRGTVHSPLINTGEEILLVLAVWGILILIRTCWTLSYLLYTNKSNPKQLKDNLKISVLSGLSGVRGAITMAGVLSVPTVLADGSPFPERGLMLFIAAGVIIVSLVMAIIFIPILTKVRTPIELRGTAHEDTNDETGLVPVDDQLLSEFDAKIYMYRVAVGTVESLRHIENNRAALDLIAEYQLMMRRFENQQNEQTEGDELPPLIAEELILRQVAFEGERQKLETLRSDGEITAKTYRRGKRKLRQKQIALSVIGSNFSWRHVQLMFYRGLTMLKRLIARLKTSQKSTAALTEWRTTEKEMAKAGIKQLSYFLKQPENRQHHYNRQVIYQIIIRYRNQIENMKNIGHTKTDIYEQQLQKLRIKALTSERLAIQELLEQHKISWELADELRKDINYAENALILADTDTE

>fig|1664.9.peg.264

MTRLYTFLRRHYALGLSFLLPFAIIFIYGLSRHVFPFGGQTIMTVDLGQQYIDFFAYFRTTLLQHPDTFFYSFAKGLGGDMLGVWAYYLMSPFNLLVLLTPGKWLSFGIWLMVLLKYGFSGLSFAYYLKKSRLLSGWWLPTLSLAYALSGFAIANQFNVMWLDAMIWLPLVILGIEQLFERHRFWLYPLSLAALLIINYYMGYMVCLFVIAYFFWASVHHFKTWRQTYLAYLKFAGGSILAGLLAAWLLLPTFFQLTQSKGQYTIQKIHWKIDYNPLKILSKLVVGNFNFDQMPKGEPNIFVGSLVLIGFIAYFLTRSIPIKERLAALLVTGFLGLSLCFEPLDLLWHGMQFPVWYPYRFSYVISFWLIVLAVQRLHYQPQFKWYSLLAPLLLLAASLAYTFKHVKTFSALSERQVLLSVGFLIAVSLILMLPARFKRYTTVFLLLVTGGEMTANAVLSLNQISYVSQKDYADYTEQLQSAVAAVPNKVPFQRIGKTFMRTKNDPMQANYFGGSHFNSMLEPSYPKFMGSIGEPAGDGSVADTNQTMFTDALLSYRYYLNSTSVQATVPRSTNKPDLSDYRQIKQNGQIAIYQNDNALPLGFAASDKVLKHKVTADYPILEQGNIATKLTDNPTLGSQLFTLQYFNTVSYQNTKQKPKLNETFLRINSKKDAHVTYTFTPTTNDPYYLTLGSSLEKNAVRLTLNGQPITQYDTFRDTVALNLTNKALGKPQTLDIAFSKDEIWLQNINLYSLNLETLSKITTQLKQSPLHIKHFSNTHISGDITIKHKNQVLMTTIPYAPGWQVTVDNHPVKAVKGLKNFIAVPLKAGHHQIKLTYRPPYFKLGLLISVVSGLMAILIAIITRYFRRRRLF

>fig|1664.9.peg.265

MKIMKKKTKRNRERSHIPFRLNFLFFIVFALFAALIIQLGRLQVLNGASYQAMVNSTDKKIVTGNVPRGMVLDAKGRVLVGNSAKSAITYTKNMNVLADKMYAEANQLTKYLTIDKLDTLTERDQIDYYLSNPKNLKKWNARLPKSRKVAADGSKIPDKTVYQSTIDLVEKSFTGLSDKQKQAAAIFKIMSGAYQLSTVYIKNDNVTDKEIAEISEHLTSLPGINVGTDWERSYPNGDSMRSIIGHVSTEEQGLPEDGINSLLAQGYSRNDRVGTSYLEKQYESILHGSKSQTQVEVGNNNQILSSKVVFKGQKGSNLNLTIDQAYQTKVEAALKKIYAKAVAAGATTYSDGAYAVAMNPKTGAILAMAGEQNDRTTKQASDDALGVINRTFVMGSAVKGATVMGGMMDGVISPTNNTLPDTPIYLPSTPVKKSVYPVGTFGSLSAQSALEVSSNIYMMQLALREGKAEYIPNKKINISPDIFPKMRGYFNQFGLGQKTGIDLPGEAKGIEGPTLTDAGLPAVGSALDLSYGNYDAYTLIQLAQYISTIANGGNRMKPYLVDSIQQTGTDGNLGKLESKTSPTVLNTINAPKSYFNVVKEGMYNVVNGTNAWGTAHTLKDIKPTFGAKTGTAQSFAREDPNDSTSKQVETVTSSLVGFAPYNDPQVAIAIVFPNLTSDEGHYNTLLAREMITDYYKLNNISK

>fig|1664.9.peg.267

MQTKQQIRQQGLAALQRLAQQPANKQQQEAILLQKLIDSAAFQSATVIGMTLNQPIEVATQPIIEAAQTAGKRIVVPRTLPKRQMAFYELTPTTVLERSHFGVLEPQITGEPIVPDLMIVPGVVFSLDGWRVGFGGGYYDRYLAAHQMDTVALALKPQQVKVPDWSVDQFDVNLKHIITA

>fig|1664.9.peg.268

MKVNHTTKDWREIPFVAYALIIMNVLYFGLMFVAPMIWAQGITSVQGLLAGGVVPFLMASINHISFLSLLMDCVILYFVGSQLESLIGHWRLLAIYILSSLASLITQGLFLGTGSQTITAGLAVLGIFGGFLMLGDAFKDNPVFGQIARQYWLFLFLTAGLQFVVNRNAMYGMVGGILGGFLSSMALGAPRIGKINPLNRVVSGLIYVITLILFVYLGMNR

>fig|1664.9.peg.269

MRTLYDVQQLLKQFGVYVHVGKRLWDIELTSIELRHVYDAGLIDGKVYRNAKMVLVHEYRLEEKQTFNHELLEEKFK

>fig|1664.9.peg.270

MTDKKLIGVDLGGTTAKFAIMTVDGDIQQRWSIDTDILDEGSHIVPDIVESINHHLDLYNMTPEDFIGIGMGTPGSVNSEEGTVIGAFNLNWKVTQYLRRDIEAGTHMKLAVDNDANVAALGERWKGAGENQPDVAFVTLGTGVGGGIVADGHLLHGVAGSAGELGHVTVEPRGYECTCGKKGCLETYASATGVVRVARDMAEEYAGNSKLKTMLDDGEQISSKLVFDLAKESDPLAVKVVDRVSYYLGLALANVGNMLNPAYIVIGGGVSAAGEFLRKQVEAYFEEFTFPNVKQTTKVRLANLGNVAGVIGAASLALQFDQQ

>fig|1664.9.peg.271

MFVIGTINPMFVVNIILIAILLWIIGVWVYYKIQRKRLGGELTEKAFEENMRKAQIVDLRERKDFDAGHILGARSIPYPMLKQNMGELRMDLPVYLYDTGITLSVRAALKLRKVGFTNVKWLQKGFNNWTGKTKRKK

>fig|1664.9.peg.272

MKHKFFKGFVIGTVSTVGAIAGSLFAFKKTVLDPIEEEENKIEENRRRANRKSHSAHQG

>fig|1664.9.peg.273

MKKEKIIVIVGPTGVGKTALSLQVAQQLKGEIISGDAMQFYRHLDIGTAKVTPAEQAIAPHHLIDVADIDERFTAFDFQQQGQQLITKITDRQHLPLIVGGTGLYLQALLYDMTLGSATDAEQDFSIRNKWQAYLEQHSETDLWQALAKIDPDAAAKIPAANTRRVIRALEVYETTGVLFSQQKPKELRYDTFIIGLNCERPVLYERINQRVDQMVDAGLIEEARWAYERRATSPQAVRGIGYKEFFPYFDGECSLEAAIEQVKQNSRHYAKRQLTWFRNQIPVNWYNLVEHQEADLARIQADIQTWLQK

>fig|1664.9.peg.274

MNWQDSLAPELRAKIEAVNAQIAPRLQALDEQIINNQAKVLQAFQEENISESHLNGTTGYGNDDQGRDKLEAVYSRFFKTEDALVRPQLVSGTHAIGTALLGMLRPGDDLLYLTGEPYDTLQEVVGMAGNGIGSMKEYQIGFDYVPLLADGSVDFETAKTKITAKTKVVAIQRSRGYADRDSFTVAKIEEMVRFIKGINPELIVFVDNAYGEFSETIEPTEVGADVMAGSLIKNAGGGIAQTGGYIVGPERLIEMIGYRLTVPGVGASEGATQGNLQLMFEGFFLAPSVTGNAIKGAVFEAALLEQMGLNVSPKWDAPRTDLIQTVNFGNPDDMVKFAGCVQAQSPIDSFVTPIPSDFAGYEDQIVMAAGTFIQGASIEFSADGPLRAPYTLYLQGGLTYAHVKLAISRAVQTTFFEK

>fig|1664.9.peg.278

MARKMLTAEAIKQLVDQENVKFLRLMFTDINGIIKNVEVPISQLDKVLSNKMMFDGSSIDGFVRIEESDMYLRPDLSTWLIFPWEAEHGKVARLICSVYTADGEPFLGDPRNNLKKMVREMQDKGFKDFNIGPEPEFFLFKLDEIGKPTLKLNDQGGYFDFAPVDLGENCRRDIVLELEKMGFEVEASHHEVAPGQHEIDFKYADAVDAADNIQTFKLVVKTIARKHGLHATFMPKPLHGVNGSGMHINMSLFNQDGTNAFFDENGKEQLSETAYHFLAGLLRHARAITAINNPTVNSYKRLVPGFEAPVYVAWSGHNRSPLIRVPQSRGLSTRLELRSVDPSANPYLAISSILAAGLSGLEQGLSPEAGVDRNIYSMDETERKENHITDLPSTLHNALKELAKDDIIKDSMGTYLYQSFMDSKSLEWAAYRQQVSEWEREQYLELY

>fig|1664.9.peg.279

MKTLVIVSHPEMANSLVQNFLKASAMPFQDEVTWHDLATIQQLDVAAEQALLRDHERIIFQFPLYWYAAPASLKTWLDTVLTGEFAFSGRMPLQGKSLGVVVSTGIAGKHFQAGGSEQATLSEILRPYELVARKLGMTYLPPFAIHQFSYLTETARQLLLVDYQRYLTQTTFNFEQKVAWFEQQLRQRQSAATDSEQQQQLALLADQLTEQGELLTDLGWQVQLIRQEEGEG

>fig|1664.9.peg.282

MTKNVAVLIGSLRKDSFSKAIAKQLMPLFPEDMHLEVIEIGDLPLYNQDYDDLDQVPEAYTTFRNKMQQMDAVLFVTPEYNRSVPAVLKNALDVGSRPYGASVWDNKPAEIVSVSPGAISGFGANHHLRQSLVFLNMPTVQQPEAYIGNVMNLLDDNGQVANPDTIAFFQVIVDAFVTLINRYID

>fig|1664.9.peg.283

MEIKWQVATQQSQTMKQFLLKNGISQRLYAKIKAQGLPVRLNNRLSSPADQVQNGDQVSLTLPAEPADETVAVSGVPIEVLYEDDYWLVVNKPAGLATIPGPTNTTDTLLNRIKGYWQQNGAIDLVPHIITRLDFDTSGIVLAAKHQVAQSLLQPQIEQHQLQKFYLAVVAGTGLPEFGTIKAPIGRVGVEPRRRVIADGQSAWTDYWRLAQDERLTVLKVQIHTGRTHQIRVHLSDLNHPLVGDQLYEGPLGWGIDRQALHAYQLTFNDPFSQQKRQFTAPIPADIQMILPTKINDKL

>fig|1664.9.peg.284

MRKLRIVLICVFCLVLGGVIGTLGTTRVAVQGLVSPQKADRQPIVFVPGSGGTNDRFDQLFKTVNQHYRQHSILKVEVMADGQVKTKGQIVWRDKVPLIVVSFENNNDGDENVFKQTEWFEKAMQQLTKQYHFKKFNGVGYSNGGLVLTRYAERYPKEAQLSRLLTIGTPYNGLNASIKDESPMLTDLKSQKEHLSKKMVVYSVGGTTTGGDDGIVPASSVAAGREVFQNQIAHYMSMFVSGNDAKHTSLPENETIVYLIEELIVQQPVDPQKPLSAKLK

>fig|1664.9.peg.285

MTNSPQIKKELTGYIIRDVFSSLGLSIYILADTYFIAYAVGPIGLAALNIDLPLFNLFNGLGLMLGIGGATIFSINKVNHPEKSQAIFTEVLSFGILLGLIIMSLGLIFIHPLLHLLGANHETLGPSLAYLRVILIGSPLFILNNLVLSFVRNDSNPHLTMIATLSQSLFVIIFDYLLMFPLHMGMMGAALATICSPLVSLLILTRHRHHPDRLLTLKKLTLNFKPTFKAVQLGFPSFLTEMSTGVSIFVFNIVILHLADNYAVAAYGIIANILLVGLSLFNGVAVGVQPIVSREFGKRNWHNVKTSLRIGLLSALGIATGLYLVLLGFKEPIIAIFNHDHNQLLVHYAAAGIPLIFISFFFSSMNIVNNLFMTAIAQPRLSFFVAIMRGYVLLIGSVLLLSALFGLTGVWLSVPLTELVVLIGGFFITHSLLNTLGE

>fig|1664.9.peg.286

MFKTLEIFKTVYETKNFTTAAQLLFLSQPTVSVQIKQLEESLDVVLFERNGRQQIVPTKQADLFYEQCQQLLDLWHQDLHQLQHQERQAKIPCRIAASHTTAAYFLPALLSRHRDALDALEIEICTLNSSQIIEQLSQHKLDFGFIEKPLTEKDIVRTPLQTDQLVLAGQPTEPWLLRENGSGVYHYTMNYLKENNLTLDNVMTVKSNAIIVQLLRQGLGQSLISSAALLDYPEIPYRPLSKQYQRQFYLVNRKGLPQPIQDFVATLLATPLPIE

>fig|1664.9.peg.287

MQSLIKKLPGFIVSLVIAIGSYYIAQLWLPMLGGATIALFVGIILGNTWLKRPGLAAGTKFSEKRLLEYSVMLLGATITFQSIQKIGWQGVVLTVLQMSLTIGFAIWLGRRLRFSEGTYLLMAGGNAVCGSSAIGAIAPVIDADNEDTGIAITMVNLMGTILMLALPVLGTLLWGHDNLARGILIGGTVQSVGQVVASATMINQGTVVTATLFKILRIICLVFVVTGFGYLHQKKQSQATDTKLSQQLLAKKSSLVPWYVLGFLIFCVINSLHLFPGQFGTICHFLSTWCEMTALAAIGLRLNLKTLFNQGKQLLFYAGGLVVFQVAAAILLITILF

>fig|1664.9.peg.292

MFKKKSDWFLVLALVMMAILFYSSSMTYHEQTSVPLLERVLKNEPLKQWLSQFSFHYAGSEQSVKASGYFKFVEFFVRKGAHFGTYFLLALFSYLGLRPRIEGAFLAGIFSWLAATGYAATDEFHQMLTGDRTPLFQDVMLDSVGALSGIVIMSVILYFTGRKKRR

>fig|1664.9.peg.293

MSGHSKWHNIQGRKNAQDAKRGKIFQKLSRELFMAARSGGPDPSSNAALRLIMDKARSANMPKDNIKRAIDKADGSDGANYDEITYEGYAPGGVAILVHALTDNKNRTSSDVRVAFTRNGGTMGAAGSVAYMFDRRGYIAIAREGLDVDEDQMFEDILEAGADDLQTEEDVFEIYTDPKELANVRDILEKKYTLANAELTMIPQNTTPVDPEKVEQFQRLVDALEDNDDVQDVYTAGELPEEE

>fig|1664.9.peg.294

MSTEEKLTTLLKHCAKQRVQDLYFTPTIGGWRLTERRQSDLVTNQELDKPTGTRYLNRLKYMAGMDISETRRAQTGRSELQLADQTIYLRLATVGDFLNRESLVIRFIYPIGAIYHCDDQQILAQLTQMSRQAGLILFAGPTGSGKTTSLYHLAQQSMSAKMVVAIEDPIEIVAPEFLQLQVNDNAGLSYAELLKISLRLRPDTLIIGEIRDLETAQYAVSAALSGHLVLSTIHARSTQGVVARLLDLGISELQLRACLTGIAYQSLVTKDDVVQAHYELLTGPDYFSKKEGGCDV

>fig|1664.9.peg.297

MIEMCVVLAIVSLLSWLPIYQIKQYRAQQAEQLFLHQFETSWDAARQYVAIEPRAVRVMWDAPTHAITFKGAGEAFRNHQLVLPETLTVSNPAEWHLINITHNKGIKPRTLKLKSTLNHREYQYKVQMMWGVLHVQK

>fig|1664.9.peg.299

MVISMAILILTSQLLVATVGVLQKAIKVTQQQQNTINWHISTIQLDQFLADAYFTHHQDQDGSKITFRKPDTNQSGEEAETVYYLKRMKNEMVVVSQLDGYMPLLGNLKQLQLDYQRPFVQIDATFTDQSRYQHEVFLEEQDEQAKKKTIKTD

>fig|1664.9.peg.301

MPKSAVETLFDLIDQTTTLIEQNLETTYLDALTESIANIADGGRVKVEDNLPDAQTVAQLETIYATVKLNQFDAETIRQALQLATLKGLRQVKVEPNKQMTPDAIGYLVAYLAEVFGGADQIKTVLDPVIGTGNLLATVMNHIQNLTGNKLQGFGVDNDDSLLELAGISSELQGLDTTLFHQDAIEPLMVKPVDIAVADLPIGFYPIDERAADFETHAASGHSYAHHLLIEQTMHYVKDGGFGFFLVPNVILETDEAKQLVKWITKHVYLQGLLSLPVNLFKTKEGQKAILVLQKQGAGAQQAKQILLGEFPNSNDQKAFAAYLQQIRTWHKENIN

>fig|1664.9.peg.302

MSKILAINAGSSTLKWKLFVMPEEKVIAKGMVDRLGLSDSVFTVKYGDGQKYEITQDIETQDVAVDLLLKKLIEFKIIAEYSEISGIGHRVVAGGEDFKTSALITEASLKRIEELAEYAPLHNPAEAGVIRAFRNILPNVPQVAVFDTSFHTTMPEENYLYSLPLDYYRKYGARKYGAHGTSHRYVSHRAAEMLGKPLEDLKMITMHLGSGVSMTAIKDGKSLDTSMGFTPLAGVTMSTRSGDIDASLVAFLMKKLDIKDPEEMIDILNKKSGIYGISGLSPDMRDLEKTREERPESQLAIDIFVNRLIKYVGSYVAIMGGLDVLVITAGMGEGDILMRQRIGDRLSYFGVEVDPERNNVMAQERIISTDDSKVKVLLVPTNEEVMIARDVMSVGQIK

>fig|1664.9.peg.305

MQTKSHQILNNFDIKILGILLMFVDHIHQMFAGAGVPDWVDWFGRPVATIFFFMAVEGFTHTRNQKRYLTQLLIGFWVMNFGDRLIQQFFTVGDIALSNNIFTDLFIAVLAMYGIQEITAGRRAHNTKQMIIGFLAIIVPIMMSVLVILLLVNPKTAMLAANIGMVIPTITLAENSIFLYIGVFFYLFRNNRLLQCLTIIVFAVINAGANSGFTFTGLLTTNTQWMMIFAIIPILLYNGQKGRSMKSFFYFFYPIHIWLLYILASFII

>fig|1664.9.peg.306

MSKLKATTTLIIGLLLASSPLITPKPIQAASGTADYQQAQKINQKNKKTGKKISKLQRQINKLNKQLAKLATETNTPIANTTNHAASELATLDYQGDNEIIVNNNQPTFTTADLATTNGPWQTFSNLDQLNRAGTANALLNKSMMPTAKREGLTWNPTGWRNKRVKSGWLYNRSHLIGYQLTGENNNPKNLITGTRQLNAPEMLAHESDIAAYLKKHPSSDVRYRVTPIFRGNELLARGVQMEGQSIGDNSIQFNTYIFNVADGVTLNYADGSSKTAE

>fig|1664.9.peg.310

MDYAEMLDRQKHIRNFSIIAHIDHGKSTLADRILEMTDTIAKRDMQAQVLDDMELERERGITIKLNAVELHYHAKDGETYIFHLIDTPGHVDFSYEVSRSLAACEGALLVVDAAQGVEAQTLANVYLAVDDDLEIVPVINKIDLPSAQPDVVKAEIEEMIGLDASEAVLASAKSGIGIEEILEKLVTDVPAPTGDLEAPLKALIFDSNYDSYRGVVLNIRVVDGTVKVGDKIRLMNSGKEFEVTEVGVMSPKAVKRDFLMVGDVGYITASIKTIQDTRVGDTVTLADNPADAPLDGYRHIQPMVYSGMYPVDNAKFNDLREALEKLQLNDAALEFEPESSQALGFGFRCGFLGLLHMDVVQERLEREFNLDLIMTAPSVDYHVALTDGTEEVIDNPSEMPETSNISEVKEPYVKASIMVPNDYVGAVMELAQRKRGEFVTMDYLDTYRVNVIYNMPLSEIIFDFFDDLKSNTKGYASFDYEVTGYRASDLVKIDILLNGEAVDALSFIVHRDFAFERSRVIVGKLKETIPRQQFEVPIQAAIGNKIIARSTVKAFRKNVLAKCYGGDITRKRKLLEKQKAGKKRMKSVGSVEVPQEAFMSILKMNDEDTKGK

>fig|1664.9.peg.312

MEAMQLSIETVARAQERLLEALEQMTLEEANTMPNPLIKSVTWLIWHTARELDYQISDLKGQTPLWLSANWTGRFGLALPDDTQDWQHSPEEAAKVVVSDRQLLIDYLSEAVKLTQTYLQDVSLESLDEVIDDNWTPAVTRGARLVSVIDDAVMHSGQAVYTRRLVIAK

>fig|1664.9.peg.317

MTNQILITTTESIPGKHYEVLGEVFGLTTQSKNVFKNIGASLKNVVGGEIRAYTEMMTESRDVAIDRLRQNAIEMGADAVVMMRFDSGSIGTDMQSVAAYGTAVKYID

>fig|1664.9.peg.319

MSILVLGGAGYIGSHTVDQLIQRGYDVAVVDSLVTGHQGAINQKARFYQGDIRDKDFMRTVFQQEDVTGVIHFAAFSIVPESMQAPLKYFDNNTYGMTALLEVMNEFDVKRIVFSSTAATYGEPKSIPIKESDPQLPTNPYGESKLMMETMMKWADKAYGIKFVALRYFNVVGAKPDGSIGEDHHPETHLLPIVLQVAAGKRDQLSIFGDDYDTPDGTNVRDYVHVLDLADAHILAFEYLADGHDSNAFNLGSSTGFSNMEIVEAARKVTGKAIPLEMAPRRAGDPSTLIAASDKARETLGWAPKYDNMEAIIETAWNWHLNHPNGYADR

>fig|1664.9.peg.320

MEAQYEWQLKPVPTEDELQTITKDNPLPKAAAQLLWQRGLRDAEQINGFMNPNVGQLHDPYALHDMQKAIDRIQEAIVNGEKITIYGDYDADGITSTTLMKETLDDLGADVEVYVPNRFKDGYGPNLEAYKRLIENGTQLIVTVDNGVSGLEPIAYAQEHGVDVVVTDHHELPSELPNAVAIVHPRHPEGQYPFGELCGVGVAFKVATALLEEIPYDKLDLAAIGTVCDIVPLVDENRTLVSLGLQQLQNTDRPGLVALCQSAGLEQATLDATNIGFGIGPRLNAIGRLGDATLGVQLLTTLDDEEAVEQAQFIEQQNKKRQGLVQEITATAMTIAETTENQASATLVIAHEGWHEGVLGIVASHVVEQTGKPTLVLTIDPETGLAKGSGRSVTAFHLFKALDAHRELLVHFGGHHMAVGLTAKVDQLALIQAAMNEYAQVNQLDLTGRQPLPVDLALTLADITPDLYQALQQLAPFGSDNPEPLIELSAPQLADVKQIGADQKHLKMMAVDQQQQLAVLAFNKGALMPDLTAAEDVKLVGSLSENTWRGQTTLQLMVKDLATSGQVILDQRSNRLTKQLFQSTGDYVFFNDKMREQLQSYLPAGSQALLATDEQVATSQELIVVDEPADLTAFEQFYQQQQAAKLSLIFYAKHSAYLEGMPTKQQFATFLVYLRKHPNIEKARLAELAVYLKLQLPLMIFMLQVFFDLGFVRIERGLITAELQPEKHALESAPSYQKRLAKIEMEKQLVYSTFTEVKDWLATLPAVANR

>fig|1664.9.peg.321

MSINFKDYVASVPDFPEAGVTFRDISPLMSDGEAYAAATDKIVEYAKNKGVEMIVGPEARGFIVGCPVAYKLGVGFAPARKKGKLPRETVSASYGLEYGKSTLYMHKDAVKPGQKVLVTDDLLATGGTIAATIKMVEELGGIVVGTAFFIELKDLNGREKIKDYDIFKLMEY

>fig|1664.9.peg.322

MTKTESKQLEVLRFIHERVQDKGYPPTVREICEAVNLSSTSTVHGHLARLEKKGLLQKDPTKPRAIELTPAGLTAIGATPQKIPVLGVVTAGVPILAVEEATDYFPLPPSLQTEQDLFMLTIRGESMINAGILDGDEVIIRKQSTADNGDIVIAMTAEDEATCKRFFKEADHYRLQPENDTFEPIILNEVSILGKVVGLYRDRM

>fig|1664.9.peg.323

MAMDQKRLDRINELAHKAKAEGLTPEETTERQELRDAYLKDFRSSFRSQVEMLQVYDKEGKEVTPEKVKDIQREKGLRDD

>fig|1664.9.peg.324

MNIGIGVLIFVIGALLGAVAGFFGARAYMKKYFEENPPVNEDMLKAMMMQMGQKPSEKKLNQMMSSMKAQQKRSKK

>fig|1664.9.peg.325

MGIFKKLGWYFKQEYRRYILGVIFLVLVAIVQIVPPKVIGTLIDLMAARKLKASVLLMWIGVLLAAAILQYLFRYGWRTRIWGGAAKLERTLRSRLFWHFMKMDTTFFQKHRTGDLMAHATNDLTAIQQVAGAGILTFTDSIITGGTTIVAMIIFVDWRLTLMALIPMPLLAVASGRLGTHLHTAFRESQAAFSKLNDKTQESVSGIKVLKTFGQEEADINDFDQIVTKTIAINKRVNFIDGLFDPAISLIIGLTYLVTIIYGGTLVMNHSITIGQLIAFISYIAALVWPMFAIGRLFNVLERGNASYDRVESLLKETSTIIEKPDAIQTLATGDIDYAIQQFAYPEEQTPTLTNVHFQLPQGKTLGIVGKVGSGKSTLIKLLLREYDQYQGQININQNDIRDYTLDALLDSIGYVPQDNFLFSTDVRDNIRFADFEKEQEAVEAAASSGAVHEDILTFAQGYDTVVGERGVSLSGGQKQRIAISRAIMTDPEILILDDLLSAVDAKTEEAILMNLKTLRANQTTIITANRLSSVMHADEIIVMDDGTIAERGTHDSLLAQQGWYAEMWVKQQLNQAMGGQ

>fig|1664.9.peg.326

MAQKYQSEWSQKISTKEQFQIMKRLFGYTKRYKKQFIGAIIAAAGLATINILLPRLLQVFMDRYLTKQTATTQILLGVAGLYALGVILKAITQFAQSFLFGMGSERAMEDVRRDLFRQLHRLGMRYFDQTPAGSIVSRVTNDTMTLIDYWNVFLSLLVGSFSIISAFIAMWLLSPKIALATAGFLPILLVIIWYYSSYSAKVYRHMREKLSQLNTKLNESIEGITIIQQFRQEKRLTAEFESVNEDYLKTREAMIRTNSLLLAPVVNLLYTLALVVVLSLFGLTALHSFVAAGLVYAFTTYVSNFFNPMTNMMDSLSSLQDGMVAGRRIFRILDQTEFAPAQDADAASQITDGRVEFKHVSFSYDGKHEILHDISFVVEPGQTVALVGHTGSGKSSIINVMMRFYEFYEGEILIDGQDIRSFQTAELRRKMGLVLQDAFMFYGDIASNIRLFNDEITDEQIQAAAEFVQADEFIKELPGQYHARVIEGGAQFSSGQRQLLSFARTLVTNPKILVLDEATANIDTETESVIQTGLARLRQGRTSIAIAHRLSTIRDAELILVLDQGQIVERGNHDSLVAQKGRYYEMYQLQNGQSGL

>fig|1664.9.peg.327

MKKKRWLKWSLISVVILIVIGVFGATLYMYQYAFVPGQKSFLNNGKPSKFYRDNQKWLAKADKVKWYQKSATDDLKLDAIYVPAAKKTTKTIVVAHGYMGYKEDMARYIHLYHDLGYNVLAPDDRGSGESEGDYIGYGWPDRLDYVKWIKQVIAKNGQDSQIALFGVSMGGATVMYTAGEKLPKQVKAVIEDCGYSSISGELAYQLNDLFGLPKFPLFYTTNLMARVRAGYNFSEGDATKSLAKSKLPIMLIHGAKDKFVPTKMVYENYKAANAPKQLWVVPDAGHGEALAKQPTAYRNKVAKFLDQYWEK

>fig|1664.9.peg.328

MFFSFARFIVRILAWLLNGRINVENKSALPEGTYVLVGPHRTWWDPIFFALAASPRKFTFMAKEELFKNPILRFILKHANAFPVNRQNPGPSAIKTPVKALRSKDLSLIMFPTGSRYSSELKGGAVVIAKMARVPLVPAVYQGPLKFSAVLKRKKITIRFGEPIPVDPKLKLNDENLTMIGDQMQAAFDQIDHDINPDFKYEIDDHK

>fig|1664.9.peg.329

MLKADERIDQLFRLDIQIIQSPSVFSYSMDAVLLADFAQLPKRGLIVDLCAGNGAVGLFMSPKTNGQIVGVELQPRLADMAQRSIELNALTEQLRVLNMDLKDVTTVIKKDSVDIVTCNPPYFKVAPNSQKNPNEHLALARHEIATDLRTVVQTMSGLLKMNGKGYLVHRPERFLEICDELRAARMEPKRVRFVYPKAGKEASMVLIEAVKDGKKGGLRMLPELTIYDENSDYTPEMRTKLFMAEDD

>fig|1664.9.peg.330

MRLLYYETFADKSSALKAEYAFKHQSRLKKERYLIAHGIESTQF

>fig|1664.9.peg.331

MAVLSMKQLLEAGVHFGHQTRRWNPKMKKFIFTERNGIYIIDLQKTVRMVDDAYDFVKEEAANEGVFLFVGTKKQAQDAIAEESVRAGQYFVNHRWLGGTLTNWDTIQKRIKRLKEIKAMDEDGTFERLPKKEVALLKKQQEKLEKFLGGIEDMPRIPDVMFVVDPRKERIAIKEAQKLNIPVVAMVDTNSDPDDIDVIIPSNDDAIRAVRLITAKMADAIIEGRQGEDDVEEATFAAENKSADSMEEIVEAVEGNNDTNTDAK

>fig|1664.9.peg.332

MAEIKAAQVMELRKKTGVGIMDAKKALVASDGNVEEAIDALREKGMAKAAKKNDRIAAEGLAGVAIDDNTAAIVEVNSETDFVASNDQFKALLKDIAETIAKNKPADMAAAEELPMGEGTIASSVINLTAVIGEKISFRRFELVEKNDGDHFGSYLHNGGQIASLVTLEGADDEAAHDVAMHVSAVNPQYVSREDVPAETLDHEREVLTEETKNEGKPENIIPKIVEGRVNKFLSEISLNDQAFIKDSDQTVAQFVASKNGSVKGFVRFTVGEGIEKKENNFVDEVMGQIK

>fig|1664.9.peg.333

MTNTKYKRIVLKVSGEALAGEQGFGINPEIVKVVANEIKSVHDLGVQIAIVCGGGNIWRGVTGEQMGMERAQADYMGMMATVMNALALQDNLESIGVPTRVQTSIEMRQIAEPYIRRKAVRHLEKGRVVIFAGGTGNPYFSTDTTAALRAAEIDADVILMAKNNVDGVYSADPNTDPSAVKFDTLTHLDIINKGLQVMDSTASTLSMDNDIPLVVFNLNEADNIKKVVEGANIGTTIEGEE

>fig|1664.9.peg.334

MVNPILEQAKENMEKAEASLRRTLGQIRAGRANASLVNRVNVEYYGAMTPLNQIAAITIPEARVLLITPYDKGALEDIEKALYTADIGISPANDGSVIRLVIPQLTGERRKEIAKEVGKEAELAKVVVRNARRDAMDNLKKAEKASEISEDEMHDLEEQVQNLTNEATKKIDAISKDKEKEITEG

>fig|1664.9.peg.335

MKKTFELFRLDWRRILKSPIAFLLILALIIIPSLYAWFNIWALWDPYSKTQDLKVAVYSADQSVTVAKKKVAIGDELIDQLKKNDKLGWQFTSSKKQLDEGVKTGKYYAGIYVPKNFSKDLISFVDGKIQKPTIIYSSNDKINAIAPKITSAGATTLQSTISEELVQTVAKTLMSSLNKAGFKLDENLPVINRFSSMILKTDQQIPEVNQYTDQVIALQKKIPEMQAKLTQANDFVNFLPEANKMAKKVVGVNQYLPEVESAGALAVKVQGKIPEIQSAGKQVATLDSDFDGIANTLTNGISEAKTGLTVLNKTQTVLPEIITFGKSAQQVSSQVKNDLIPKLKTTLPVIQSAVDQGLTITDQLAKNIDNDLNAVNKLLEQAKNDPDNQELKAALKTNLEQLSAHTATLQANNTQLANTLQSLQDAYNESAVAAGKPESHLLDDPIQKLNAAATHLGQLKTKIDNVLAHYDDLSLTDIQNQITPIIAFAKQVQNTVSQVQNAGIGANVKSVLAKFETMITQGDAVLNQINTAVLPKLPSLLTNTQKTLKTAIDYLEKYQKQIPALKQEIHDANTLLNGNTGTIVSGLNTASDLYQNDYPTLKNKLQLATDFINNDLPGIENDLTTTLAMANAKFPLLEKALNDATDLIHNDWPFLRSGIQKGAEAIRKQQKTVDLKDLIKLLRRDATKESNFLAEPVKLQEKHVYPIKTYGSASAPFYTALCLWVGALLLSNIVLTNFSLDDKQKERYSKKQQFIARWLTYIVIGVAQAVFVALGNIFLIKAYIVSPVALLLLAVFLSMVFMTIVYVLAAMFGNVGKGLAMIILVLSISGGGGNFPVVLSDKFFQFINPLLPFTYGVNLLREPTGGIYAPNLWHNFIILAIYAVVFFFIGLFLKDRINPFFEKLHKEAAKSKIIH

>fig|1664.9.peg.336

MFTNKKETAKIQLDPENIPNHIAIIMDGNGRWANKRLMPRVAGHKAGMENVKTITKAASRMGVKVLTLYAFSTENWKRPSDEVNFLMQLPVDFFGTFMPELIEENVRVKVMGNIHDLPEKTQKAAQDAMADTAQNTGMILNFALNYGGRDEIQQAVQQIAKDAVSGTIEADSIDDDLISQYLMTGFLGEYADPELLIRTSGEERISNFLLWQIAYSELVFVDEFWPDFTPQVFEKSIFTYQQRHRRFGGLK

>fig|1664.9.peg.337

MKQRVITAVVALIIFIPILISGGIFIDIAAAVLALVALSEVFIMRKRIIVSPDAMVAGLAVLSLALPAGAFDWLPDGVSQFDLFYLFVAILLAITVFTKNRVNFDDMGVTTLASLYIGLGFHYLAGARNLGGFDTVMYILLVIWMTDIGAYMFGRAFGKNKLWPAISPNKTWEGSIGGTLLAVAVAAVYLYFFPQIYSLPIMLGLTLIFSICGQLGDLVESAYKRYYGVKDSGKILPGHGGILDRFDSMLFVLPLLHLFGII

>fig|1664.9.peg.338

MAAIIAFIIIFGILVVVHEFGHFYMAKRSGILVREFSVGMGPKLFATRKNGTTYTIRWLPLGGYVRMAGMADDESEIEAGTQATLILDEQGRVQQINTSDKVTTLNGVPFQIAKTDLQKELWVEGYEGGDESEMKRYPVLHDATIIEADGTEVQIAPVDVQFQSATLINRMLTNFAGPFNNFILAILAFILFAFLSGGVPQQSNQIGTVQENSAAQKAGLKANDRLLKVDNKKVASFTDFSAIISEHPNETVAVRVQRGATEKTIKVTPKAVKVANQKEKVGQVGVTQKVKMDHSLKAKISYGFTQAWSIASQIFKILGSFLTGGFSLDKLSGPVGMYSMTTQFTQQGFNALVYFLAFLSLNLGIMNLIPIPALDGGKLVLNIIEAIRRKPISPEKEGIVTLIGVGIMVLLMVLVTWNDIQRFFF

>fig|1664.9.peg.339

MKQSKLFIPTLKEVPNSAEAKSHRMMLRAGYIHQVSAGVYSYLPLAYRVLENIQAIIKDEMSKIDAVQMQMPGILPAELWEESGRYATYGPNLFKFKDRHSRDFILGPTHEETFADLVRNNIKSYKKLPLTLYQIQTKYRDEDRPRYGLLRGREFIMQDAYSFSANEADLDTTFQQMRQAYTNIFERCGLDFRAIVGDAGAMGGKDSMEFSAIAEIGEDTIVYSDQSDYAANLEMATGVRPGQSSTDVQLEMEKVATGDAHSIEEVAAHLEVPAQKIIKSVLFIADEKPVLVLVRGDYEVNDVKLKNFLDADFLDLATAEQVQATMNAPMGSIGPVNAPEDVQIVADYSVEALVNAVVGANEADHHFLNVNSKRDFNVADYADLRFVQEGETAPDGEGKLQFTKGIEIGHIFKLGTRYTEQFGATFLDENGRAKPIIMGSYGIGVSRLLSAITEQQADENGLVWPSAIAPYDLHVVPVNVKDDAQVELAEQIEGLLEEAGYSVLVDDRKERAGVKFADSDLIGLPIRITVGKKAAEEIVEVKLRKTGETLEVKKDELINSLSILLASEK

>fig|1664.9.peg.340

MALDQTALFQKLLEQIALPADVAHYPGFKTGQVEQVIVHETSKRWTFKLHFDNVLPFAVYTAFEEHLEAAFQAIAAVSIEISTDAKELDGGILAAYWEYVVNHSGIQSSLLHELCAKETPYIEGRKVLLVVENDIVKTFFINQAQTTIQAGYKRLGFPNFAIQPLIDESASQKSIEDFQAKRAVQDAERAQAAAEAIKKNEALKEKRKTEGKPIDGPIVMGRGINPAEPVRQMISITEEERSIVIEGFIFDKEVRVLRSGRQLLILKMTDYSSSFTVKKFSRDASDESLFAAIDKGMWFKVRGSVQEDNFMRDLTVNANDLVEVSHPKREDTATEGKRIEIHLHTNMSQMDATNPIGDYVKQAAKWGQPAIAVTDHYNLQAFPDAYAAGKKNGVKILYGVEVNLVNDGTPVVYNLRDQVLESAEYVIFDVETTGLSAVYDSIIELAAVKMRDGEVVASFDEFIDPERPLSAFTTQLTSITNEMVHGAKKEAEVLAMFKEFTGDAVLAGHNVSFDMGFLNAGYERNDIDLIDNPVIDTLELSRMLHPEYKNHKLDSLTKRYKINLEHHHRANADAESTGYLLYKLEKEAAENYDMVNVNQLNDRVGVGEFYKQARPAHAVLMAQTQAGLKNLFKLVSASMTEYYYRTPRLPKSKLDALREGILVGSACSNGEVFEAMMQKGYNEALDRAKYYDYIEVMPKAVYAPLLERELVRDNQALEEIIRNLVKVGEKLNKPVVATGDAHYLNPEDAIYRKILIHSMGGANPLNRSKLPDVHFRSTDEMLTAFDFLGPELAQELVVANPQKIADQIDEIVPVKDKLYTPKMAGAEDEIQTLTMNRAHELYGAELPEIVEARLKKELKSIIGNGFSVIYLISQKLVYKSGKDGYLVGSRGSVGSSLVATMTGITEVNPLPPHYRCSNCHYSEFFTKGEVGSGYDLADKDCPECGTPLDKDGHDIPFETFLGFHGDKVPDIDLNFSGDYQPVAHNYTKVLFGENNVFRAGTIGTVADKTAYGYVKAYERDTEQTFRGAEVDRLAKGSTGVKRTTGQHPAGIIVVPDYMDIYDFTPIQFPADDQDAAWKTTHFDFHSIHDNILKLDILGHDDPTMIRMLQDLSGIDPKSIPTDDPGVMALFSGTDSLGVTPEQINSKMGTLGVPEFGTRFVRGMLEETHPTTFSELLQISGLSHGTDVWLGNAEELINKGVVTLKDVIGCRDNIMMDLIHWGMDDSMSFNIMERVRKGKGIPDDWQQAMRDNENVPDWYIDSCLKIKYMFPKAHATAYILMALRIAYFKVYFPIIYYCAYFSVRASDFDLVAMAQGKEGVKARMKEITDKGMEASTKEKNLLTVLEIANECLERGITIKMVDIEKSDSSDFLIQDDHTLLAPFRAVPSLGDNVAKQIVSAREEKPFLSKEDLSNRGKVSKTLIEYLTENNVLNDLPDENQLSLFDMM

>fig|1664.9.peg.341

MTETSMEKINQFRNARNWRPAHNEKDLALSISLEASELLELFQWKTAEEGVQDIERVKEELADVLIYSYTMADNLNLDIDDIIAKKLVKNAEKYPAPKE

>fig|1664.9.peg.343

MNELSFETKLIEYITSGTVTVSEDDFQKGNIYETAKDYVVKTKLWQYRNDIRTTEQLWANFKQILEQHNQNTLDNPLSIMEFNQVKKIISDLKTPYDAGQFLYGLNGVSQIEIDLDDGRHVFLTVFDQKQIGAGNTVYQVVNQIERPALLTGKKDRRFDTTLLINGLPIIQIEEKRDSHDVNEALNQMHQYILENQYTDIFSTLQILVAITPNNVKYMANTTADKFNKDFAFNWQRKSDNKIVRNWREFADSMLSIPMAHQMATNFMILDGTKNKQMIKVMRPYQVYATQKVIQKLKKVDFDMGLNKIGYIWHTTGSGKTITSFKTAWLASRMPKIDKVVFVVDRIALTKQTNENYKAYDPDGAVDDDSRSGSVQGTQSTNDLSRKLKSKDNNIIVTSVQKLETLVKRKSFKSPDKHIVFIVDEAHRSTGGDAFELLQKSFKKSAWVGYTGTPMFDETTNGLRTEDIFGELLHAYTIKEAIADRNVLGFKVDFETTIDTESMKTQYLPDFYKKQHPDWKQQKVQDKIDNLSQEDMDDEIAPSFYDENKDHVHLVVQDIFKNWRNRSNEGKYNALFTTHVGGGKASTPMAMMYFKEFQRVNEEHTKNGGQLLKVAITFSQNTTNKDGMVETNQNLHDAMKIYNHQFGTSFGLDDVSGYTQDVTSRLNRTATDKNYLDLVIVVDQLLTGFDAPEMNTLYVDRTLKGAGLIQAYSRTNRIADMKEKPWGRVVNYRWPSQNEKLMNKALAIYSNKDSATLSEKEQKKKNVDSGVLAKDFKNMVQEVRGIVGQLDDLTDGFNQLPPSERQKDEMFELLRQYNVGMSKLKQYTTQDGEEDSGFNYDTPDDLIRALGMTPAQEKELTTVLTNEIKQYISVRDVVPIQQIELKMTHIKDVQVNYDYLTELVEKLLNEMHEEKVEAAQKTKKDIDQFANGLEDQNYANKIKNATQAILDGYYPPKGSSFTYPVKLDDSEQVIQEANQISVDRILLQFRNQWGITDVITSAGLRKLFSTHQYGQQDLDDAGRLRDIIAEASLSYKNLALDDDVQALSKIRYRNGLREAIYLAADKHVIH

>fig|1664.9.peg.344

MSETTTLQDITKKLWAMANELRGNMDAGEFKNYILAFMFYRYLSEHQEQYLVSNNVIDVAAGQTINAAYVEQATTEELTDYLEDISSSLGYAIAPADTWQSLNERIENSTVIPSDYQTIFDNFNKNAVLNQEAAQDFNGVFNDINLGDSRLGNSTTARAKSLNNIVKLVDDIEYKSDEDKDILGEIYEYLIGQFAASAGKKGGEFYTPHQVSKILAKLVTMDVAPSNTAFSVYDPTMGSGSLLLTVRNQLPETTQAGAIKFYGQELNTTTYNLARMNLMMHDVSFNNMTLNNADTLESDWPDGPDEAGVDQPRSFDAVVANPPYSAKWDNNETKLKDPRFSEYGKLAPASKADFAFVLHSLYHLNNDGTMAIVLPHGVLFRGAAEGKIRQTLIEKNYLDAVIGLPANLFYGTSIPTTILVFKKNRQAKDVLFIDASSDFEKAKNQNNLRDSDVDKIVAAYKSREDVDKYAHVATLDEIKENDFNLNIPRYVDTFEEEEPIDIDEVKKEIAAIDLEIAKLEKEFAEMEAQLVSTKKESD

>fig|1664.9.peg.345

MKAQKKQPELRFKGFTDDWEQSKLGNLVEIKDSARISNIYWQEDGIPYLRSSDLVDYTASGALFISKQTYNTFSSKTGSPKKGDVLFTSGGTIGIPTLKKDDSLIYVQGGSILYAKTSNSVKLDGRFLVNYFYNSRIKIYIKNSSVGGTIKHFTLMPAKKMPIIFPKKKEQQKIGEFFRKLDRTITLQQRKLELFKKLKQGYLQQMFPSKDQYIPRLRFKEFNGDWEQVKLGNIFEKKNELNRNQFTENRTISIATMTFKPSGNGADENSIPKYKVLRLGDIAFEGHKNKDFAFGRFLMNDIGDGIISPRFTFLHAKEKLDIDFWKYYIHHESIMRTILVKSTKQGTMMNELVVTDFLKQGIKVPEKSEQKIIGIFLTRLDHTITLQQNEIEQLQQLKQAYLQKLFP

>fig|1664.9.peg.346

MRRKYLFHEYFEEWMVLYKRGAIKQVTYDKYELTQRQIKTLAPTLLLSDLDRRAYQQLLNDYAATHEHQTTMDFHHQLKSAIYDALDEGYLKKDPTRKAIIKGVVQRRHKMKYLNQGELRRLFGVLNLEDEVNWDYFILLLAKTGLRFGEALGLTPSDFDFRTQQLTIDKTWNYKHYRGGFQSTKNQSSVRKISLDWQIATQFAHLIKDLPEKEPIFIDRTRRTHNDTANHYLERKCKQAQIPVISVHGLRHTHASILLYAGVSLASVAKRLGHANMTTTQKTYLHIIKELDSKDNDKIMQCLMTLN

>fig|1664.9.peg.347

MAESLNYGLNVESREYDGRNKYIRITDINDVSRKFTPDPITSPNVDISELNDDYKLQLGDVLFARTGASVGKTYHYESSDGNLYYAGFLIRARIKTAFSSMFLHQFTWGQKYKRFVNITSQRSGQPGINAKEYGNLTLLLPNYKEQQKIGEFFRKLDRTITLQQRKRELLKKLKRGYLQQMFLAKDSVNPQLRFNEFNSNWEPYKLGEIAEIVGGGTPSTGVPEYWDGDIDWYSPTEIGESIYVAESLKKITESGLNKSSAKMLPTGTILFTSRAGIGNTAILAKTGSTNQGFQSILPKRNILDSYFIFSRTTELKKYGEITGAGSTFIEVSGKQMAQMPICIPCFEEQKKIGIFFQKLDHGITLQQSKVDKLKNLKQAYLQKLFP

>fig|1664.9.peg.829

MAGRPRKPTGNNKKHLTNDEKDVRNESELMASDFPALSTTPPKWLDDDAKREYKRVVVDLKRLHITKLDQTQLSLYCNAYSKYIMASQDVDERGLLIDDKKNPSVNIMTDMSKEIRATAGSLGMTLDSRMKLVVPQIDKQPDDPFAKFGDLSD

>fig|1664.9.peg.830

MIDYTTEYAQAIVDGKIIAGKKTIQACARHLKNLTDSKDSDYPYFFDVKKANRVIEFIEMLPNPDDGKPLKLVNFQKFIVGSLFGWKNKETGFRRFKKAVISMGRKQGKSLVVSGIALYMLLYEEIPKYDRQIYCAANTRQQAKVVYNMIVNFLKQLRSKSKIIKKATSVLKSEIRQDGSGSYIMPLSSDYNSLDGLNVLLGIIDEQSRSTDYGLVDVLETSQGQQSQALLMIISTVSEKVNAWFCTQEYPYVTDILSGKITNESYFCVWYEQDNEAEIADEANWIKSNPILFDEKVKEKLLPNIRAKWQEATDKDNQPPALIKYFNMWQQESSESYIKIKDWKDTEIEIEPDLMNKDVYIGMDLARVGDLSAVSWIVPLEDENKFFIDSHAFVGTRGGLQNKIDRDKIPYDRLAKKGLVTLSETETGNIDDQQIIDFIYRLVDKYQFNVKNICFDRYSANNIINNLVEDFEMVDVAQGYATLSEPTKQFRKYVQDRNIIHTSNQLLEIAVNNAVLKQTNDAVMIDKAMYRNKIDPLAAGMDAWTRAVLHDFKHSDIANNDFYINEFTF

>fig|1664.9.peg.831

MGFFKPVNNENYNTMQSIIAGKQSPNYVSISYLRNSDVFTAVKVISQDVATNPIKLISNEDNVISDDLNYLLNVKPNETMTAWTFRFALAANLLLSGNAYARIYRDKQGNPVELRLIKPSWVTIYRDENDVLIYKINDDDARDYDLVADDILHYKYFSTNGIVGISPLHSLRNEVEVQDSGNRMLMNFFNSGLHSRGTLKIDKSELAPEAAKAIKNKFIASNNDDTGVTVLDNTMDYAQIEVDTSVLKLINSNQYSTKQIAKAFSIPTSKLGIESAHTSVVQENLDYIQNSLDHYFSVFNSENNVKLLDFKDRLKLHFEFDVSRLLKLDTKTNIEESIKMWQNGGINHDEYRKRLGYQPDSDQHQYYVMSNYIPLDEAYLTLKGGDMNAKNGNKSD

>fig|1664.9.peg.833

MLNEKIEALEVEIRDLETKFNTDLESANKLVKDGKLEEVRSLKKTLDDGKKALAEKRDTLADLKSIAEQRKIETGAKKEVKTDEPVEQRDLLRHYIASKGEIRAGLTTVDNEAILPKDIVYSAEKELKTVVDLRQFVDVIPVTTMSGTYPVLENVSEVFPTVEELEKNPELAKPKFNKVDYKIQTRRGALAISQEDIDDAVNVDGIVADQMAQRDINTSNAAILEKAKTMTAKKVSSFDDIKKMLNVDLDPAYSKVIVASQTFFNWLDTLKDNNGRYLLQDSITSASGKVLSGNVPVAVVPDTQLGKQGDSVAFVGDLKRAVKFFDRKQLSLRWMDNDIYGQYLAGVMRFDTRVADTKAGFFVTQENVVTPPASK

>fig|1664.9.peg.838

MSKAKIGLSQFQYATVENEKTNSEIFKIPGMRSAKLDITNELETIYADDGPYLVIPAGITELKLELGLVDLPTIDKQQMLGVTVESGIERYTKTIKVPDVAVMFRALMDDNKYCYVGLAKGKFNLPGMDLKTKEDKIEVAEDSITGNFVARGEEEDMLFIGREDNKDFKLDAFTKMVFNGEPSPKV

>fig|1664.9.peg.841

MAGSLGHISGTVSLDINPFKQSTRVLQQQIKATGNAVRAQETAIKSSGKSINDLKTNYATMGKQLKQYNALLAQQKGQFESYRSSIKDVNGATDEQKNKLSRLENEYNKTAASTSRLEAKMQATARTIAIQDSGWTKAGEKLTKFGNATTTAGEKLTSLGRKATIGITTPIVGGFTAAAKSAIDFNSQISAIGPLLTNGGKITASVRRELDQMSSSSKKWAMQFGVSTDKINDGMTEMVKRGYTAQQTMGAMPAVLNAAKASGDDFNDVMHVSTSVLEQFGLKTESTTGMLKNTSRVTDSLTYVANATAAGFQDMGEAMTYVGPSAHAAGISLEETAAAIGIMSNKGIEGSVAGTALRGALTRLMKPSKQNVAGFKAMGVSVEDFKKGTLTLPEIIDKIKNNTEGWTDQQRASSIAMAFGTEAQAGMNALISEGGDELRKYTKGAEDSAGTTKKIADQLNDTQAAKVARFKESIHVLGIEFGEKLLPTLVPVIEKLTSMIRGFSNMDSATQRSIIKWALFAAAIGPVSGVLGNVLKVTGSVSSGLGELFTMLGRVSGTSKAAKLGIEGAGTALNSAGKGAGLFSSGLSLLNPYVLGTIAVVGAGVAVWELWGKKAVESANRTSRWGTDVSDSADKSLTKMQGFSTQASAALEGFNGKTKTTTKQVASNFSDMYEQMVTDSNNSIKKMEDDINDLPGFAKKDAQANVLQRKKANAEILADARKQNELIQAVLKKHNGDVSKLTDDERTIVLNGRTRMNADEVKLLQIGGKAKKSVIAALNGDIDTMNNQQRGKAIDDLTSQFRSEEKAYQKQAATIKKAYSDNTISAKQYGAYMKQLKAEHNASTESMVASVFKLAKANGESKDQITQDLLNFGYTYEQAAKIVKTQSQNMSDSTGIVAADTANMSKATAKANEQWNKLIFDPKTGKVKTNAQEEVNKAAQSKKGWNDLHYDLKHANLSSNAKLMIGEAALANGKWDDLTWKEQQAIVAVKGNKEMADIIQQFGIWDQFTPKQKEAILHGDASPIANLLLKGGQWNMLTLKEQQALVKDKATVPLVNILDKYGVWQGLSDEEKNAILNTKGAPDLADMVIKYGAWNDMPQKQKDLMINNADARQKLTDAGILLDNYKTNNPASKPLKAHDGGLAGAVIAGNDQINSIKRNNPPSKPLKGHDAGLGGSVSRGNNQLNGFKGNNPSSKGLKAHDSGLGGAVTTANDNVNNFKRNNPKIKNLKANDNASGPASSASNAVDIFSRKKDHTVTLTSIFKSITKKITGHARGTSFHSGGDALVNDQKGSNYRELIITRKSAFVPEGRNVFLPNLERGARVIPANKTKRILKSIPHFKNGTANNTRALGTLLKAGKISDSKSSIINQTVQVSNFSGLEEKLDKLDKLDEVLGYLSKILVKNPNLIMDGKRVSKIVEPGVSKNQMQNNVLTERGVFSG

>fig|1664.9.peg.847

MKLNQFARLTTTYSEQIKALQRIKLLDEGYEALSVQALAQQIFARFFPEAHSKTAQNEQMQKIQATASLNLADYLAGISTSFDQRTFYNIALQLLGFKVTTDFQFNHPRRFMAKVGIPYVDQPVLTQELFLEAVYLLLTTRSQNGLLYLDCLANRGFFAHWQKATAPEFLIFNGKTQPVFDTQNFIREVVYVESSLDTDLDGHLDLLETTIFRPKETEKGLRVPALYTASPYYKGTNDVDADLHNVDVPIQAKAAIQPNLADLKTGTNQSVPAAREPLGETTEPELEAADDSNYLLNDYFLARGFATVYAGGIGTRGSDGMRTCGSPEETASTTAIIEWLAGNRRAYTNKTDRIEIKAWWCNQKVAMTGKSYLGTLATAAATTGVEGLKTVIAEAAISSWYDYYRENGLVVAPVDCQGEDADVLAKLCQTRQMDAADHTKSGALFEEQLAALREGQDRITGNYNAFWAERNYRDNVQKINCDVVLVHGLNDWNVKLQNAGALWDDLRQLPIEKKLFLHQGQHIYMNNIQSIDFTDMMNLWLSYQLLDIDNHASEILPTVTIQDNTQEATWHTQDDWLNPKNPRQTYFLNDPEHLGLDQTPTTPVADFSDDGVAMFKKQHLSEAAWQDQLLAPQSDFTQNRLLLLSQAQSKQLVIDGRVQLKTKVAVNTDRGLLSVMLVDYGLFSRLGTTPAILAAKGQQLGYHWRYDDLKEFKLGALSPYQLITKGHLNLQNRHNSYQTETVDAGTFYEVQLDLQPTHYHLAAGHQLGLVIYATDMGMTLREEQQNQYQVDLGASRLVIPTLD

>fig|1664.9.peg.848

MAETITCRQTLSISNHRVFASDLNEHETVYGGRLLELLDGAASIAASRLARVETVTASIDQLNFIAPFKLQDSLCIESYVVGVGHRSIEVFTKIIGEHLQTGERFLGLTAFLTFVTTEKTPNLPEIAPTTAEEEFICAGYGQRKVQRLANFKNQQAFNQQINLDYPWDY

>fig|1664.9.peg.850

MGSIKKARIRWFSLIRIFGLGMVLYYHYFKTRYAGGFVGVDVFFTFSGYLITALFIDEFIRRDKVKLWAFYRRRFNRIFPPLLLMVLIAVPLSSIANPDLRTNLTKQITATLSFTTNFYEISTGGSYETNFIPHLFVHTWSLAVEMHFYLIWGLIVYLVSRTVKSAKYFRTAIFSVSFVLFAVSFLSMFTQIKGLSEYSPIYFSSLTHGYPFFVGAMIGSFCGVGQSAQGFNQLTSRLTWKTPLIGFVVSALLLFGLGTRMKFDQASTYAYGILLGSLITGCLIIFARLLHDKAPNVAEPRFITYLADISYAMYLFHWPLYIIFLSYFNNNIKAVVAALIGSVIFSTLSYYYIEPLLMGKPVAYQKAVGGPLLFAMVILGVVTANYAVDAPHMSSLEQHLWVGNLYQDADQIQAVHAKVSQPKQTVPVSQTNYNVPQGISIIGDSVTLGVRSYLLEHVKNSDINAEGNRRMDQAYDVLMNQQNNNQLREYVVICIGTNAFPDFKEQIDKIIHDLKKGHRLIFMTPYDGHADATYDSTKIAVYERTLPAKYPFITVTDWNKIAASHPEVFEGTDGTHFGGIEKGNILYTKGINDAIKTAGKKTVKA

>fig|1664.9.peg.851

MSMVAVSALNTAAANAATVDETQEAITNNKSETAKLLKDIATANADNIKLQQQITDNTTKIETTKTNIDQSTQKIATLKTQITSAKAEVQKRTTVLKAQLVALQKQTGDTVSGNVYFDFVLNSKDFSDLVARSFTVNKLNQASKSALEDVKAAKAEYSNLMTEQETTKSNLQSEKTSFEAQQTDLKAMKAKSDQQQDALNQKVNDNKATLEALQTQYDEAASALKVANDTKAKETKVVKTAVSTKDTSDKAEATSTSNDSKASTSSKIENKTSDNSESKKSNDDSDSQGDGSTHGNVAGNSYAWGQCTWYVKQVAPWAGNNWGNGGQWGASASAAGFRVDHTPSAGAIIVFVPGQSVGGQWTADPTYGHVGYVQSVSGSSVTITQGGMGFANAAGPNTATLSDGGSYMYIHR

>fig|1664.9.peg.853

MTEKYILAIDEGTTSTRSIIFNHDGQKVADAQREFPQYFPEPGWVEHNANEIWNAVLSTIANAFIKSGIQPNQIAGIGITNQRETTVIWDKATGLPIYNAVVWQSRQTNDIAEKLKTDGYEDLIHKKTGLIVDAYFSATKIRWILDQVPGAQERAEKGELLFGTIDTWITWKLTGGATHVTDYTNASRTMIFNIHDLDWDDDILKLLNIPRAMLPDVRPNSEVYGETAPYHFYGSSVPISGMAGDQQAALFGQLALQPGMVKNTYGTGSFIVMNTGEKPTMSENNLLTTIGYSINGKVNYALEGSVFVAGSAIQWLRDSMQLVEKSSDSEAAAKASTSLNEVYVVPAFTGLGAPYWDAEARGSILGITRGTNRQDIIKATLQSLAYQTRDVVQTMQKDTGIDIKMLRVDGGAANNNYLMQFQADILNSAIEKAANLETTAMGAAFLAGLAVGFWQDTDELAQIFSVGQRFEPNMPEEEREDLYAGWQQAIEATKIFKHRPYKMNE

>fig|1664.9.peg.854

MTFSLESRQQAIQNLKNEQLDLLIIGGGITGAGVAIQSAASGIKTGLIEMQDFAEGTSSRSTKLVHGGIRYLKTFDVGVVADTVSERAVVQGIAPHIPRPTPMLLPIYDEPEATYDMFSVKIAMDLYDKLAGVTGTQYANYTIDRKEVLQREPGLKSDRLMGAGVYLDFVNNDARLVIENIKEADELGATVASHVKAVGMTYDANGRVNGLKAHDELTDETFDIHAKLVINTAGPWVDKVNALNTEVKAQETLRPTKGIHLVVDSSRLSVPQPTYFDSGLHDGRMIFVVPREGKTYFGTTDTDYTGDYKHPRVEQTDVDYLLKAINNRYPSVDIALNDIEASWAGLRPLISNNGSSDYNGGGANSGKVSEESFNNLIKTVDDYENKTAARADVEKAISQLRTAHAEETVNPSQVSRGSSLKVAENGLMTLSGGKITDYRKMAAGALEMIRDILKKDFNETVREIDSKKLQVSGGHFDPNNVEETLKFYTKVAMSKGISEDDATDLANRFGSNVSRVVSYADQGTAEGLNLKETISLRYSVNEEMTLTPVDYLLRRTNFVLFHNDQLAAIKQPVINEMARLLNWDAAEKERQTAQLDAAIAEAQLDYLK

>fig|1664.9.peg.855

MDNLIMVQALGELVGTFILVLLGDGVVAGVSLAKTKANGAGWIAITLGWGFAVTLGVYASAFMGPAHLNPAVTLAFAIIGKVSWSVVLPFIIAQMIGAFLGAVVVWIQYYPHWQATDDQSAILGTFATGPAIRSPFANLMSEIIGTFVLVFALLALTRGQFTEGLNPLVVGVIITAIGLSLGGTTGYAINPARDLGPRIAHAVLPIANKGDSDWQYSWIPVIGPFVGGILGALLFVLLP

>fig|1664.9.peg.856

MTDVAIVMGSISDWETMQETAQILTDLNVSYTKKVISAHRMPIEMIAFAQSAAEEGYQVIIAGAGGAAHLPGMLAANTVLPVIGVPIQSKALNGMDSLLSIVQMPSGVPVATVAIGKAGAINAGLLATQICGLKTPTYQKALVQYKQALHQKAVASNDQLN

>fig|1664.9.peg.857

MTNLTKQILPGQVIGIIGGGQLGQMMTLAAKSMGFKVIVLDPQVDCPAGQVADDQIVAAYDDENQIIELAKRSDVVTYEFENVDATTIEKAQQLTQIPQGTKALRIAQDRVLEKQFLATEKLPHAAFKVVTSISELTKAVAEIGLPCLLKTARGGYDGKGQLVLRRASDIEAARALLDFGVCVLEEMVTFDQEISVMISQNWAGQQALFPVIENQHRHNILHISICPARVPAAVAQEAKEIAQKIATEIELVGTLGVEFFVTPTGQLFVNEIAPRPHNSGHLTIEACDLSQFMAHIRGGCNWPLQTPRLWQSAVMVNVLGQDVPGALAKSRQSTDWSYHDYGKAERKENRKMGHITLLTDDLNKTLSEIEATQIWALPKE

>fig|1664.9.peg.858

MIERYTNPEMGAIWTDHNKYEAWLAVEILADEAWSKLGHIPAEDVAKIKANAQFSIPRILEIEQETRHDVVAFTRAVSETLGAERKLVHYGLTSTDVVDTAYGYLLKQANAILRADIDALIEVIGKQAKRYQDTVMMGRTHGVHAEPTTFGLKLALWYSELKRHKERFEATAEGVEAGKISGAVGTFANIPPFVESYVCDQLGIRAQETSTQVLPRDLHAAYVAEMALLATSIEKFATEIRGLQKSETREVEEYFAKGQKGSSAMPHKCNPIGSENITGLTRVIRGHMVTAYEDVALWHERDISHSSAERIILPDTTILLDYILKRFTKIMTNLTVFPENMKRNMGRTFGLIYSQRVLLKLIDHGLSREAAYDLIQPKTARSWDEQIDFRPLLEADEQVMSILTPADLDDAFDYHYHIRHVAEIFERVGLG

>fig|1664.9.peg.859

MMSETLLYSGKAKDLFSTDDPEVLKMIYKDQATALNGKRKEQITGKGEVNYEISKLIFAYLSQQGIETHLIKNVSETEQLVKKVTIIPLEVVLRNVVAGSFARKFGQPLGQRLEQPIIEYYYKNDALDDPAINISQATALKLVTPTEVATIEAMTKQINTLLIKLFNEIGIDLIDFKLEFGRYQGRLILADEFSPDNCRLWDQKTHSSLDKDVFRQNKGDLVTVYETVLQRLTKMIGGFANV

>fig|1664.9.peg.860

MYNVQVYVTYKDSVLDPQGQAVQGAIQRLGFEGISQMRIGKFFEMQVTGTDQATVIEKVEAICDRLLANPNMEQYRYDVQLVEDAK

>fig|1664.9.peg.862

MVNNPTATEIQQTKLYRQWGLTDSEYELICTKILKRLPNYTETGLFSVMWSEHCSYKNSKPILKKFPTNGPHVLQGPGEGAGILDIGDGQAVVFKAESHNHPSAVEPYEGAATGVGGIIRDIFSMGATPIAILDSLRFGELNDNQTKYLVQEVVAGIGGYGNCIGIPTVGGEISFDPCYQANPLVNAMCVGLIEQKDIQQGKARGAGNSVLYVGAKTGRDGIHGATFASDEFAEGKATQRSAVQVGDPFMEKLLMDACLELILQHSDWLVGIQDMGAAGLVSSTAEMAAKAGTGMILDLDQVPQRETDMSVYEIMLSESQERMALCVRAGYEDQVIALFKGYDLDAVRIGEVTTKEQYQLWHQGQLVADLPVAALTDAAPVYHKDQAKPERLATFAAQAPYVPSVTDTQATWLALLKQPTIADKSSFYRHYDAQVKTNTVVLPGSDAAVVRIRGTKKALAMTTDCNGRYLYLDPHVGGQIAVAEAARNIVAAGGQPLGITDCLNYGNPEKPAVFWEFDQSAQGIAAACETFGTPVISGNVSLYNEFNGEAIYPTPMIGMVGLIRDIQDITTQDFKQVDDLIYLIGETDDNYAGSELQKMQQGTISGQLGHFSLATEKANQDLVLKAIQQGLITSAHDLSEGGLAVALSEATFKQGLGYHVQVDLASRQLFAETQSRFIVTVKAADQAAFEQISDQSAQLIGWVTAEPIMHIQTKDEMIDLTVEAAKDAWEAALPCLMKSEA

>fig|1664.9.peg.863

MLNEIRSLNEECGVFGVWGHPDANQLAYFGLHSLQHRGQEGAGIVTNDNGQLLGYRDRGLLADVFRNPENLAQLTGEAAIGHVRYATAGSHGLENIQPFLFDFADGQFALAHNGNLTNAVTLRRQLEDEGAILKSSSDSEILMHLIRRAAGPDLHTKVKTALNQIHGGFAYVLLTQDSLIAMLDPNGFRPLSIGQMANGAYVVASETCALNAVEATFIRDVQPGEVVTIDRDGLHSDFFTTETTHSICSMEYIYFARPDSDICGINVHAARKRMGKRLAAETGVDADMVIGVPNSSLSAASGYAEASGLPYEMRLVKNQYVGRTFIQPTQALREQGVNRKLAVVKSVVAGKRIVLVDDSIVRGTTSKHIVKRLKEAGALEVHLRVASPPLKYPCFYGIDIQTREELIASQQSVAQMCETIGADSLAFLSQEGLIDAIGLHQDAPYSGLCMAYFNGDYPTPLYDYEADYQASLKQDKE

>fig|1664.9.peg.864

MTDAYQKAGVDVTAGYEVVTRIQKKVGADNHNIGSFGGQYALEMAQYQKPVLVSSTDGVGTKLMVAFAADQHATIGIDCVAMCVNDIVAQGAQPLYFLDCLATGKTDPDKIEDIIAGVLEGCKQANMALIGGETAEMPGMYADKHYDVAGFAVGIAEQDALVTGETIQAGDVLIGLASSGIHSNGYSLVRKIFFEQNNLTVASQLPELPGKPLGDLLLTPTKIYVEDLQPLLQQRVIKGAAHITGGGFIENVPRMLPADLAAHLTLGSWPILPIFKALQQYGQLAEMKMYNIFNMGIGMVLAVAPEAAPAVLAQLNAKQEQLIRLGPFKNVNKRRLNW

>fig|1664.9.peg.865

MRVAIFASGTGSNFEAIADNQRLQQAGLEIVQLVCDRPQAAVIEKAHRREVPVTVLVPRQFENRQAYEQAIVTQLAPLAIDYIILAGYMQIITPVLLGTYPQRIINIHPALLPDFPGIHGIEDAYRAKVSETGVTVHYIDEGVDTGPIIAQATVPVKSNDTLATLEARVHAVEHQLYPAVIYDLIQKNN

>fig|1664.9.peg.866

MKRALISVSDKTGIVAFAQGLVAADFEIISTGGTFSALEKAGVPVTPINEVTHFPEMLDGRVKTLHPDIHGGLLAKRDEPTHMAALAEHEITPIDLVCVNLYPFKETIQKPEITQAQAIEQIDIGGPSMLRSAAKNFKSVYVVVDQADYDETLAQLQQDDVTYRRHLAAKAFRHTAAYDSLIARYLSDADAVEFPEKRSLTYDFKQALRYGENAHQKAAFYQSALPSDFSIAAAHQLHGKELSYNNIKDADAAIKVAAEFSEPCVVAMKHMNPCGVGLGTTIYDAWQKAFPAGSVSIFGGIIALNREVDVQTANALHELFLEIVIAPSFAPEALAILEKKKNIRLMTLDFEQAAKADRFKTVSVLGGLLVQERDRLTDQASELKTVTTKAPTEDEKEALLFAQRVVKHVKSNAIVVATKDQTLGVGAGQMNRVGSVKNALEQALANGMQAPFVLASDAFFPMDDSVAYAAEHGITAIIQPGGSIKDQDSIDMANQKGVSMVFTNRRHFKH

>fig|1664.9.peg.867

MDILVIGSGAREHTLARELLKSEQVEQVYCLPGNPGMQLDQIKTAAIDVNDFEQLIAFAKKQAVAYTVVGPEQPLVAGVVDAFQAAGLAIFGPTKQAAALEGSKTFAKEMMARAQVPTAQYQTVTNESEALTALQGRSLPIVIKADGLAAGKGVVIAETLADGLAAVRRFFKQGRRQILIEDYLVGEEFSLMALVADDQVHPLPIAQDHKRAFEGDRGPNTGGMGAYCPVPQISDQMVQTAVETIIKPVVATMAANGTPFTGVLYAGLIATATGIKVIEFNVRFGDPETQVVLPRLQGDLAQVFQQLLTHQQPTLDWQADGVTLGVVVAAKGYPNQTPAIFQLAPTARFKGANSHFSFAGVVATTGGLMSAGGRLYTAVYTAPTMAQARVQIEAGLQSVADPQSFFRRDIGFKSQM

>fig|1664.9.peg.868

MTYPTSDAEHPVDVLNLVSLEGRVKERMEAGAFGYIRGGSEDEWTMTENTSAFNQKKIMPRVLQGVEHADLHTKLWDIDLKTPIIQAPSAAQGLAHEKGEVDTAKGMAAAGSIFSISTYANTLVEDAAAAAPDAPQFFQLYMSKDDQFNEFLLQKAVNAGVKGIVLTVDSTLGGYRETDIETKFQFPLPMPNLAAYSNSDGAGKGISETYATAKQGIVPEDIQKIKEITHLPVIIKGIQSPVDAELTVQAGADGIWVSNHGGRQLDGGSASFEVLPLVAQQVAKRVPIIFDSGVRRGEHVFKALASGADLVAIGRPIIYGLNLGGAQGVTSVIDHMNHELSITMQLAGTKTIDAVKETTLLD

>fig|1664.9.peg.871

MVREINPETTDPRVQRTKQQLRRALVELLKTATTKEISIQAITKQAQITRGTFYLHYEDKTDFIQKAVQDLTQDLYQTITIQNDKLAQPYQQINLLALFEYVERRQDAFKVFHKNDTSLAFKQQLTTEMRRLIEAYLNNLFQEATQSDQIELSIIADYLAGASLVLVFKWIEDGLVYSPRYMAKMLYQLVQMPNMAFNVAGFFVEA

>fig|1664.9.peg.872

MYAIIKTGGKQYKVEAGQAIYVEKLTAEAGEKVTFDQVILVGGETTKVGTPNIDGVTVDGTVEKQGREKKVVTFKYKPKKHSHQKKGHRQPYTKVMIDAINA

>fig|1664.9.peg.873

MIKADFIVNADSQIISFQITGHADSGPYGSDIVCAAVSAVSIGTINSLQKLAGFEPEVEADNLNGGLLTCQVPATVTGQALATAQILLQNLQLTLEDITQQYGQYIQVQTKRK

>fig|1664.9.peg.874

MLQMNLQFFSHHKGGGSTTNGRNSAGRRLGSKRADGQTVTAGSILYRQRGTKIHPGENVGRGGDDTLFALVEGVVRFERFGKNKKKVSVYTAEAK

>fig|1664.9.peg.875

MLSVNDFKNGLTIEFDNNLWRVVEFQHVKPGKGGAFVRSKLKNLRTGANQEKTFRSTEKVERAIIDTKSMQYLYADGDNHVFMDTDTYEQLELPAAQIQDELKFLKENMQVSIMMFGSETLGVELPKTVDLAVAATEPGVRGDTSSGGTKPATLETGLVVQVPFFINAGDELTINTSDSTYISRA

>fig|1664.9.peg.876

MAEETNIVLDTQENSLGKIQMAPEVVEVILGIAASQIEGVHQMRGTISSSINELFGRSNHGKGVTLKVIDGKVNADVYVYLNYGVSVPKVALAMQEALKEQLLFMTDLELAEVNVHVVGVVTEKTQNIIDPDDLFNDETPDLNGDQS

>fig|1664.9.peg.877

MPNFNRHQIRQAAFQVLFTLNANQSLELEDAYQAVLTMDQFDAEEVTPVEVPAYLAFLVSGVTENQAALDAALTPYLKKGWQLSRLAKPDLIILRLGLFEMQNSTEAPAKVALNEALELAKQFTDDQAKGFINGVLSKFVEA

>fig|1664.9.peg.878

MELLDGRALANELTHAQQTAVETLKEQGVTPKLVVIMVGDDPASAIYTQSKQKRATKIGMASELKRLSAETTEAELLALVKTLNDDRTVDGILVQLPLPKQINEDHVIQAIDAKKDVDGFSPVNIGQLWLNQQGLVACTPNGIMRLLAAHKIDVAGKNVVIVGRSNIVGRPLAALMLNANATVTIAHSRTANLKALTKTADILVAAIGKPHFFGIDAVKEGAVVIDVGINRLEDGSVTGDVDFEALQTHVSAMTPVPRGVGPMTITMLMEQTIEIAKERVKRG

>fig|1664.9.peg.879

MVKPTDYLTVTALTQYLKAKFERDPYLDRVYLTGEISNFRLRPNAHQYFSLKDNKAKISAIMFKSAFEKVKFTPEEGMKVLIVGRISLYEASGNYQIYVERMEPDGLGALYQAYEQLKAKLATEGLFDAPKQPLVRFPKRIAVITSPSGAVIRDIITTTQRRYPIAQLVLFPAVVQGDGAADVLVERLKQVNEHGDFDTIIIGRGGGSIEDLWPFNEERVARAIVASQIPVISSVGHETDTTIADLVADVRAATPTAAAELATPVLTDEILKLQQQRLRLYQAFSKTVALNKKQLDHLQNSYVLKQPKRLYDGYLQNVDQLQRRLLTAQQQLLKDRRQQVQLLQQRLSAQSPLMAVRQAQKDVTEQQRRLNQAMNRLMADRRQKAAQVIAALDLVSPLKILGRGFAYVTDDQQQMLKKVADFKAQQPIELHVQDGLVTAEVVATHKGEE

>fig|1664.9.peg.880

MAEKKLTFEENLAQLEVIVNELETGDVPLEKAMTAFQEGVKLSQTLEETLSQAEKTMAKVMADNGEEVPLDAEEQQ

>fig|1664.9.peg.881

MADFKQLAARELPQFDAYLKNELAKIQEPTLQKAMTYSVMTGGKRLRPLLLFAVLESAGQSIDETAFRAAGALELIHSYSLIHDDLPAMDNDDLRRGQPTNHKVFGEAEAILAGDGLLTLAFEWLATLNVTAEQRIELVRLLAQAAGANGMVAGQVEDIEGEHKALTLAQLQQVHHLKTGCLIEVAVAMGAVLAQLGEVETTALRGFAQQFGLAFQIQDDILDVTSTTVEMGKAVHKDAAEQKNTFPILLGLSGAQEALHTTCQQAQQQLAQVTTFDRTLLNSFLDYFNE

>fig|1664.9.peg.882

MKKERVDVLLVQQGLFESREQGKRAIMAGEVYGKNDERIDKPGEKIDAETVLRVKGHKIPYVGRGALKLAKAIDVFDIDLKDKLTLDIGSSTGGFTDVALRNGARRCYALDVGYNQLAWKLREDSRVVVMERVNFRYSQPEDFTDGLPDFAMTDVSFISLHKILVPLKPILKPNCDAVALIKPQFEAGPANVGKNGIVRDPKVHKNVVTDIVNFSLATGYDVLGLDFSPIKGGEGNIEFLIHLRNTEAETGTLAENVNIDAVLERAYAELKRPSGK

>fig|1664.9.peg.883

MRKVDRQRLLRNLIQTHEIERQEDFVKLLQAEQIEVTQATISRDIKEMQLVKVPNTDEGYHYSLPLEKKLDVKEKLRRTLRDAYVTHNQQDYFVVIKAHPGNAPAIASLLDQLQSEDIFGTIGGDDTVLIICKSEEQAKQFRLFIEDLLK

>fig|1664.9.peg.884

MLQELVIHDFAIIDQLAISFDEGMTVLSGETGAGKSIIIDAVGLLAGGRGSQDFIRTGAKKATLEGLFTSPENATTAALLAEYGIEHDDQTILLQRDLALNGHNVCRVNGHLVNTATLKKIGETLVDIHGQNEHQELMYPDRHLGLLDEFAHTALASAKAAYQETYQAYRQTEKALKKKQQNEQEWAQRLDMLKFQTNEIDAAQLQPDEESKLIEERAKLSNFQRINQALQASRAILSGEEGNALDQIAIAMNQMQEIAELDPEFKRIADNLQTGYYTLQDSGSDISREVDNLEWDEARLDEIEQRLETINNLEHKYGDSIPEILAYGAQITKELQAMQATEANASELEQQFEQQQAQLLEEAAVLSEKRQAAAQQLEKAVHQQLKALYMAKTVFKVEFAMVDNQQFMRSGRDKVEFYIQPNPGEKLRPLAKTASGGELSRLMLALKTIFSESQGVTSIIFDEVDTGVSGRVAQAIANKISEIAQHSQVLCITHLPQVAAMSDHQYQIQKQIKQNRTETSIQKVTGKKRVAELARMLAGTEVTELTLEHAQELVDLAETAKAQIRQA

>fig|1664.9.peg.885

MEIKQSAVVGFSHELVKRYQRFFELDDAQELIKQPQMQPSQIQLYMMQAFDRHYRLAIQFNVAEHIEQVNGRLIKQLDEQTYLLKENHSNLYKIVTLNQIRYISKNN

>fig|1664.9.peg.886

MSDINYKIMAEIGVISEDKAGWKKESNLIAWNGRAPKFDLRDWAPDHAKMGKGITLTNEEVAQLQTILNEMNLK

>fig|1664.9.peg.887

MSNRGMLIVLSGPSGVGKGTVRQAMLEDEFRDFHYSVSMTTRKPRPGEQDGVDYYFVSKEEFEQEIANDGMLEYAQYVDNYYGTPMKYVNQTLESGRDVLLEIEVQGAMQVREKCPDGVFIFLTPPDLLELRNRIQKRGTDDQATIDKRMQKAADEIRMMENYDYAVVNDEIPNAVQRIEKIIESEHLRVPRVIDQYKKMIGE

>fig|1664.9.peg.888

MIVYPSIDKLLENVNSRYSLAVLASKRAHQIEAGDLKMLSEYKSPKTVGMAMEEIAAGNVTIDPDSLMLEKDAEKMDKLSQKDGE

>fig|1664.9.peg.889

MVSLENKHVTVYVTGGIAVYKAASLVRQLIKNGAIVRVAMTQAATEFVTPLTFATLTRQEVLTDLMTMNHPEQVAHIHLADWTELAIVAPATANIVGKLANGIADDFATTALLATTAPKLLVPAMNEHMWLNPATQRNLTQLKADGIAIVQPATGFLAEGYEGQGRFPEEDVIMSNVYRQLTPNRLQDKVVLVTAGGTQEPLDPVRYIGNRSSGKMGYAIAGMAAQMGAKVTLISTRPELAVPANLAQVIYVRTAAELQGAVASLYNEADVVIMAAAVADFKPAQYVSQKIKKQVGQTDMTLELNRTPDILATLGQEKKKQYLVGFAAETNDLMAHAQVKLTAKQVDLLVANDVSQTDRGFGADQNAVTLLRPNQAPEALPLASKKVIATQILERISTALSSKEE

>fig|1664.9.peg.890

MPQIAKVIVDVPTMQTNRPYDYQVPVTLVDVIQPGMRVIIPFGNGDRKIQGFVLQVLDQPSFTGQLKAIEAVVDLTPAVNPELLSLSDWLAQQTYAFQITCLQTMLPSVMRAKYTKTATLTDEIPTDLQASLFNGNDELTFDQNLVPDKLKQLMQLQRQGKISVTYHVDNRAQVKQLTAIEPALSFEQLEEARMGLRKSAQKQSQLISYLQTLIDQEAPTLLKTILEKTTFTRENIRIGAEKGWLRQVKVEAYRNPYANAVQPTHKMTLTSEQETAVDAVVQSASTDASEVFLIEGVTGSGKTEVYLQTIDAVLQQGKTALMLVPEIALTPQMVQRVKGRFGDLVAVLHSGLSEGEKYDEWRRIERKEARVVVGARSAVFAPLEDIGVIIMDEEHEASYKQDESPRYHARDVAIWRSQYHNCPVILGSATPSLESRARAQKDVYHLIRLTQRVNQQALPAVELVDMREAMRVQKASNFSPALLAGIKERMQKGEQSVLLLNRRGFSSFIMCRDCGFVLQCPNCDISLTLHMDTHSMKCHYCGHEEAIPKICPSCQSRQIRYFGTGTQKVEAELQELIPEARILRMDVDTTRKKGAHAKLLKKFGDHEADILLGTQMIAKGLDFPDVTLVGVINADTALGLPDFRASERTFQLLTQVSGRAGRADKVGQVIVQSYNPENYAIQFAKQHDYEGFYQYEMAVRHRGGYPPYYFTVKVTVSHPEEALAAKKSFQIAKQMRPILSDQALILGPTPKSIARINNRYFYQIVIKYKQEPQLQAKLAEILETTQIDTRHGFKIGLDNEPLQFT

>fig|1664.9.peg.891

MGTPQFSVPILEALVANDYQILAVVTQPDRKVGRKQVLQQTPVKEAAVRLELPVFQPEKLSGSPELADVIALQPDLIVTAAYGQFLPTKLLEAAKIAAINVHGSLLPKYRGGAPIQYAVLNGDSEIGITIMHMAKKMDAGDMIEQASIPIEATDDTGSLFDKLSYVGRDLLLKTLPGIIAQTAPRTPQDEVQVTFAYNITKEQEQLDINQPAEQLLNQIRALRPQPGAWLAVNGQRTKIWQATVAETTTDQAAGVVVALNKKDFELAAGNGTVLKITEIQPAGKAKMPVQSYLNGVGKQLAVGQQVVVQDAE

>fig|1664.9.peg.892

MPNKTPRYLAVEILTAIAKQNSYSNLALDQVINKNRLNPQDAGFLTQLVYGVIQHEYTLDYYLAPFIQKPKKMDNWVRQVLRTAVYQQVYLDRVPEHAIFYEATEIAKKMGHAGVAKLVTAILRQLQRSGLPDLNAIADPIERLSVQYSVPQWLVAKLQADLGDDKLESLLATINQPANASLRVNTRVNTVDAILAELQPQFNDLQPSQIAKVGLVAQGGHLASGALFEAGDYTIQDESSMLVAPSLDIQPGDQVLDACAAPGGKTTHMATYLDAQRGGQITALDIHDHKVRLIEQNAKRLHVEDVVSAQALDARKVGEQFGEAHFDKILVDAPCSGLGLMRRKPEIKYSRQPADLMNLQRIQLAILDAVAPTLKVGGRLTYSTCTIVPEENQQVVAAFVANHPEFEIEPVPLELPLAQNQASPFVQIYPDDYQTDGFFIACLKRRA

>fig|1664.9.peg.893

MEVAYQTDVGQQRQNNQDYVGFYTNQRGVQFAIVADGMGGHLGGDVASEMAVSHIGHEFEKTDDTDIEAMVKWLIFELQRENQHILAKANQYDDLSGMGTTLVAVLISGTHYLVANIGDSRVYRLRRNTLRQLTEDHSLVNELVKQGELTAEAAKHHPQKNIITRTLGVSQEVDADVTIYEFEPDDYLLLCTDGLTNMVDDDQLQQTLMAEMTLEEKCAALIQKANEAGGMDNITALILHHEGEVPTP

>fig|1664.9.peg.894

MMETGYAVAGRYKIIRPIGEGGMANVYLAQDLILDRQVAVKVLRLDLRNDPSTVRRFTREALATTELNHPNIVSIYDVGEENSMQYIIMEYIKGTDLKKYIVEHFPIPYQRVIDIMTQILSAVQNAHAHNIIHRDLKPQNILVDEDGNIKISDFGIAIALSETAMTQTNTLLGSVHYLSPEQARGSMATKRSDIYSLGIVLYELLTGMVPFEGESAVSIAIKHFQDEVPPVRNYDPRIPQALENVVLKATAKDPDERYSGVDAMMADLATSLSASRAHEPKFVPSKADDLSETKVIPALDPEGITQTNNDEEQVKEAESTQKKVKKPAKSKRKWWLLGGGVVLLLLLATVIAFAVQGSEVTVPDLTDMTQQNAESALTDKKLKVGTVQKTTSQKYSKGHVIRTTPKAGLSVKSKSTVNLIVSSGRQKYKIKDYTNQAYSDVRDALKDKGLKVKRKYQSSSEVSPGLIIDQSIAAGKRVVPSKTTITLTVSSGKPGFALHNLSNYTKQGIQDYAAQNGLTVVFSEEYSSSISEGMVISQSPASGTMVQEGDSVSVILSKGEQPTEHHSIVTSSSNSVIESSAPESSQSSESSASESESESESESESESASESISSSASQSSQSSVQSK

>fig|1664.9.peg.895

MQTGQIIRALSGFYDVQSEHKIYRTRARGNFRKRKITPLVGDFVEFESESQTESGYILEILDRKNEMIRPPVANIDQAVVIVSAVEPDFSLNLLDRFLIYLESLNIQGLVYLTKTDMISDEKYQEIKQYLDYYEKVGYPTFAPRTAFTPEIIQAIEDTFPDKTTVFTGQTGAGKSTLLNHIDPKLNLATAEISQSLNRGKHTTRHIELIPLNDGLVGDTPGFSSLGILNVTSETLVSRYPEFREIGQDCKFRTCQHVMEPKCAVKAAVDAGEIMQSRYTNYLQFRAELKDIRPVYKKSK

>fig|1664.9.peg.896

MTQIAPSILSADFMNLQRDVQNLEAAGADLLHIDIMDGMFVPNMSFGAQVVSGIRPLTQLGLDVHLMVEQPERYIEQFITAGSDLIMIHAEATEHLYRGLQMIKDAGVKAGVVINPGTPVSYIESVLPLVDQVLVMTVNPGFGGQKFIPAMLTKVAELAAYREAHADADFTIEVDGGVNDQTIAACAKAGADVFVAGSYTFAGDLATRIETLKKAANEAR

>fig|1664.9.peg.897

MKRVNLLAGGPVDQWASELATITTVPGQWITADRGTLRLLQQGVTPTIAVGDFDSITPAEKEQVLAAVSDIRSVQAEKDETDTQLALSIAFEELQADRVVVYGATGGRIDHFLANLFMAVEERFRTVLNRIEMRDRQNTIQFYAPGTYELTKEADKKYLAFIPLVPTTDLNLVDEKYPLTHFDTTVPISWASNEFIGEKAHFNFKTGIVAVIQSKD

>fig|1664.9.peg.898

MAKDVITGRKTSFGNKRSHALNASRRSWKPNLQKVRILVDGKPKRVWVSARALKSGKVTRV

>fig|1664.9.peg.899

MAVKIKTQYGDIDITNNVIATVVGGAATDIYGIVGMASKNQIRDNLNEILNRENYNRGVVVRQEENGVAIDVYIICGYGIKISEVCRNVQSKVKYNLETMLGVSANSVNVYVQGIRVLED

>fig|1664.9.peg.900

MKVTEITATEFQAMMRVAAHRLTKNAEFVNSLNVFPVPDGDTGTNMSLTFQSGAKAVNENNAQAVGELSKSLAKGLLMGARGNSGVISSQIFRGFSKSMEEKTSLTAQDLADAFTSGVQTAYKAVMKPVEGTILTVAREGAKAGAKVAAQTDDIGAVVEAVVEGSKKALLKTPELLPVLKEVGVVDSGGQGLVFIYEGFLEGLTGQAPAKDLYTPDEAEMDEMVNANHHQTAQMGTEEIENGYCTEIMVALGDGTTVDREFDYDEFRNHLNGIGDSLLVVADDEVVKVHVHTEHPGTVMAYGQHFGSLMKIKVDNMRLQHDTIVENDQQAAPVQAEPVDTGIIAISAGAGVAELFRSLGATYILSGGQTMNPSTQDILDAIQAANAKKVILLPNNKNIFLAAEQAAEVAEIPVEIVQSRTIAQGMTALLSFDPSAELQANQVAMTEALDTVISGQVTQAIRDTTLDGLEIKKDDYMGIIDGKIKVSTADRQTAAIEMVQTMLDEDSEIVTIIVGEDGDMTEAEAISDAIMAQDDELEVEIHEGDQPVYPYLISVE

>fig|1664.9.peg.901

MLSLTDSVTVLTGVGPKRLTALNQLGIATISDLLYYFPFRYEDLKVKDLSEAVDQEKVTLKGTVVADPVVSRWGPGKTRLNVRLLINHDVIMVTFFNQPYLKDKFEAGVDIAVYGKWDARRNSLTGMKVLAVQSADNPSFAAIYSTNKNVRQGTLVKLIREAFDNYQSVIPDLIPADIRERYKLVSEVELIAGMHFPESYPEAKQARRTAIFHEFFLYQLQLQAIKQADRHVENGLALPYQNAALKEFIKTLPFDLTDAQKRVVNEICLDLKAPAHMNRLLQGDVGSGKTIVAAIAMFAAVTAGFQAALMVPTEILAEQHYQSLQKLYAPMNVTVGLLTGSTTAKERRTLLADIESGRINIIVGTHALIQDAVVYHKLGFAVIDEQHRFGVNQRRMLREKGLQPDMLAMTATPIPRTLAITAYGEMDVSTIDELPKGRIPIETSWVRSNQVEQALSFVQKQLADNSQVFAITPLIAESEQMDLKNAEEIYATLAERFEPQYHVALLHGKLKDDEKNRIMTAFSNNEIQLLVSTTVVEVGVDVPNATVMMIFDADRFGLAQLHQLRGRVGRGKKKAYCLLIADPKNQQGVDRMTIMTETTNGFVVAQKDLELRGPGEVFGDRQSGLPVFKVGDPVADFVSLQVAQQEVQKIFTVDPTFSQPAYAPLKAYLAQQKQNYQTLD

>fig|1664.9.peg.902

MKVAVDAMGGDFAPQSVIEGVLQARSEFTDLDFILYGDQAQIEPLIDDMTRLTIVHTTEKIASDDEPVRAIRRKKQASMVLAAQAVKDGQADALFSLGNTGALLAAGLFIIGRVKGIDRPGLMPTLPSINSDQGFNMLDVGANAEAKPEHLHQYGLMGNFYAKDVRGIENPRIALLNNGTEATKGDELHKATYQLLVDDPDLNFVGNVEANDLLKGVADVVVTDGFTGNATLKAIEGTATIVMSQVKHAIMDAGVKEKIGGLLLKGSVGGIRERFDTSIYGGAVLLGLKAPVIKAHGAADARTVYYTVKQIHVMLANQTVQKVIDYFSDQAAQKNSN

>fig|1664.9.peg.903

MSEQEIFDKIAAIIADRFELTQDQVTNELNFKADLDADSIDIVEFVLELEDTFGAEIPDEEAEKIATVADAVAYIKAKTK

>fig|1664.9.peg.904

MRREKGLVLGVTTVAITLLLAACGSSSSKNAGSADTVSFSTTDVISTMDPALNTDVIGAQALTDTMEGLYRYEGKTIKPAIATKVVKPTNNGLTYTFPLRKDAKWSNGKAVTAEDFVYAWRRVVDPKVGSQYAYIYEGIENAAAITSGKQPVSSLGVVAKDKHTLQVTLEKPIPYFSQLMTSSTFFPVYPDAVKKAGKSYGTNAKTLVFNGPYKLSGWNGPDSAWKEVKNTNYWNAKAVKVKTLKYQVVKDPSTALNLFQSNKLDRTNISGDTAKQMKNDSHYSTQQQAATFYMQVNQAKNPIFKNAKIRQAISMTINRKELVNQVLGDGSAPVASLTPKGMSFDPTTKKDFTSELNAEGKKNTTYNPKEATKLWKEGLAETGQTGKTFNYTLLGDDTDTAKKQAEYLQDTLEKNLPGLKVTLANVPFKTRLTRSDNGDFDMVVSAWNADFPDPITFLDLFVTGGDNNKGKWNNPEFDAQIKASKTENANNDKARWQNLIKAQDIMNKESGVIPLYQSGRAYLTNPSVKGLDYGPSGSYNNVSLYLKK

>fig|1664.9.peg.905

MSKYLIKRIFYLVLTLFIVASVTFFMMKLMPGTPYTNQQKMSADQLRIMNEQYGLNKPLFQQYIIYLGGLLHGDFGTSFQFNNQPVANLIMTRLGPSLQIGAQAMLLGSVLGVLLGAVAAIRKNTWVDGVATFFSILGISIPNFVLAVLLQFFLAFKLQMFPIALWDGWSSSILPSLALAVAPLANTARFMRTEMVDVLSSDYIELAKAKGNSKWQTVAKHALRNSMIPVVTIIGPMAVNLMTGSLVVENIFSIPGIGEQFVKSIMTNDYPTIMGLTIFYSFLLTVIILVVDILYGIIDPRIRLSDGQGAR

>fig|1664.9.peg.906

MADAKLTAGSFKPLNKDTHEEQEKISAPSLTFMQDAIRRLKQNKVAVVSLFVLLIIIIAAALAPVIAPHNPNTQNVAYANLPPKIGDGQLPGFKGETDVSGVKVDKYQQAGVPKGKYFILGTDYLGRDLFSRILYGTRLSLLIGVLATLVDLLIGIPYGIISGWRGGRIDTFMQRFIEIISSIPNLIVVVLMMLILRPGLLSIVIAIGFTGWITMARLIRAQTFQLKEQEFVLAARTLGETPFKIAMKHLIPNLSSTIIIQTMFTIPSAIFFEAFLSFIGIGIPAPNASLGTLLSDGQKAFRFLPYQMWYPAAVLCVIMIAVNLLADGLRDAFDPKS

>fig|1664.9.peg.907

MANILEVNHLSIDFKTYAGDVHAIRDVSFNLKKGETLAIVGESGSGKSVTVRSVMGLLASNAHVKAGTVIYHGDDLLKKPEREMDAIRGSDISMIFQDPMTSLDPTMTIGKQVAEPLLVHKTMNKEDAMKRAQTVLELVGIPNAKERMKDYPHQFSGGQRQRIVIAIAIVDYPQILIADEPTTALDVTVQAQIIDLMKELQRKIETSIIFITHDLGVVAGIADRVAVMYAGKIIEYGLVDEVFYEPKHPYTWGLLNSMPTLETAGKLEAIPGTPPDLLDPPKGDAFAPRNPYALQIDVEEEPPFFEVSKTHFAATWLLHPDAPAVEPPAAIQALMKKHNVL

>fig|1664.9.peg.908

MDKQQRKVLVEVKHLKQYFNPGKKSEVKAIDDISFEIYEGETFGLVGESGSGKSTTGRSIIRLYSPTSGEINFDGQDISKIKEHSRQMKEFRKEIQMIFQDPYASLNPRMKVKDIIAEGLHIHHLVKNEAESDARVNELLDLVGLNKDHATRYPHEFSGGQRQRIGIARALAVKPKFIIADEPISALDVSIQAQVVNLMKEIQEQQNLTYLFIAHDLSMVKYISDRIAVMHNGKIMELASADDIYDRPLHPYTQSLLSAVPLPDPKIERQRRRMPYDSSIESDDGDRQLREVAPEHYVYATEAEVASYQSALATLVQQAN

>fig|1664.9.peg.909

MGYLILDAALVSELRARYGIEFNNLALLDEAFTHSSYVNEHRELGLRDNERLEFLGDAVMEITVSEYLYKKYPDWPEGKLTRLRAAIVCTKSFSSFSKEAHFDRYIRLGKGEEKNGARARATLLEDLFEAFNGALFLDQGRGAVVDFVSQVIFPKIEAGEFSDQTDYKTNLQEFLQQDGEIEIDYQLLAEVGPSHDRQFEVDVLVGDRVLGSGVGRNKKAAEQAAAKKALEQLKA

>fig|1664.9.peg.910

MQLKSLVLSGFKSFADKTEINFSDGLTGIVGPNGSGKSNITEAIRWAMGEQSAKSLRGEKMPDIIFAGTDLRPQMNRAEVTLNFDNSDHYLNQELDNVTLTRRLFRNGDSEFYLNQKSCRLKDIVNLFMDSGLGRESFSIISQGRVEAIFNSKPEDRRNIIEEAAGVLKYKQQKKKAQSELDQTDENLSRIADIVYELKGQVEPLKEQSSIAQDYLEQKAQFNALHQQLLVVEIDQLAADQTQYQAQAKTLAKALGEIESEIQETNTALADNQQEVATLDQEIETANETLLVKSRLAENLQGQENVSKERASYTDANRQNLVDRIAQLETQSTTAKQQQIALKATYTEKVATLTNLKNELVTLKRQASGSEAELKAKIEQIRQDYIDQMQAQTTNRNEQQYLEKALLQTKTQLTRQDSSINETSAQLTQMQADKAAKEAQVAQLATDYAALETQLSQLQQQISATQKQYQTEQNNWFQASGILQKAKAKQASLAELNDDYAGFYQGVKAVLKQKQQLPGLLGAVAELMTVPNDYQQAIELALGAQLQQIVTTDEKTAQQAIDYLKRNRLGRATFLPNNVVKPRTLPSSLVNQLQTEPGFIGIASDLIQFDDAVSPVMMHLMGNLIIATELTEAIKIGRLTGHRYRIVTLAGDILSPGGSMTGGHNNRQNNGGLLARKQTLTDLEQQISKMQLALDQKQTKVQTLHQDLAEQQAQLEQQQGAFETAKTHYQTQKNELTLLNERLTQFERQQQATDYQVQQQQQSYDVDLKRQGELQAAATEIEAQLTQLKADLGAANEQLQHFDQSQEQIRQQQTALETKLAVAQSEQKNVQEKLTDATQLANDLKQQLETSQQALAALQQADSEDAMTQKERRTQLKATKALIQKLTAELATKRTARETLKATQQTLQANATRVYQLQRNSLAEQEQNAVALNRVKINIDQRLTTLREDYQLSYEAAKSDLQASDLTNDQLKSKLKLLKLGLADLGTVNLAAIEDYQRVKERYDFLMQQDADLLDAKSQLLASMAEMDAEVEKRFKQTFDQTAAAFEEIFPMMFGGGHASLTLTDPSALLTSGIDIIAQPPGKKLQRLSLLSGGERALTAITLLFAILKVRPVPFCILDEVEASLDEANVDRFGRFMKRYESETQFIVITHRKGTMTQANRLYGVTMAESGISKIVSVSLEEHETA

>fig|1664.9.peg.911

MGLFDRIKKAFTLEPEKEPEKVDEQATTAEKEADDPAEKSTEKLDDSTGETDSGETPETPETPETPETPETPETPETPETPETPETPETPETPETPEVPVEEEPTEPVVAETPTPVETESEPEVVEPEVTAAPEEAVVATDEPTATEETTVADDVEAEPVQAAQTEKYDQGLEKSRKTFGQRLNALFANFRSVDESFFDDVEEMLIEADVGYETAMKIADELREEVKLRNVKKPEAVSQAIVEKLVDLYGQEGQQEDNELHFAPEGELTVFLFVGVNGAGKTTSIGKFAHQLEKAGKKVLLAAGDTFRAGAIEQLQEWGRRVSVPVVANKAGSDPAAVAFDAVKRAKEENFDVLLVDTAGRLQNNVNLMKELEKVKRVITREIPSAPQEVLLVLDATTGQNALVQAKQFKSTTDVTGIVLTKLDGSAKGGIVLAVRTELHLPVKMVGLGEQMNDLQLFDPNRFVYGLFKDIIVGDGPQSEIDTDVSQAIK

>fig|1664.9.peg.912

MEMDKVLAQKVVARLHREIPYNINVMNQQGTIIASGEPSRVGQSHPGAIEAIQGQKTNIIEQDQPDGTLRGFNMPIVVQNEVIGVVGITGAPEKLTYIAPLVKVTTELLTEQALADQVLSRAKRRLERFLFDWLAIQDPAALTPDFVQEANALGVNIDEQYQAMVIKTPHLLPETVEVGSYKLRITPDELLILTRQAEVLAHWHQNAKEVQAAVGISQPQKLVSEALKQAREVIKYNQGTNQKLLYYTAEVNYAYRLLSADIVVDEYVEKFRVLAQTDSGQVLITTFQKYFRNNGNIVETAHQLHIHRNTLNYRLGQIKDKLGLDPKRYDELVILYTAFLNYELQ

>fig|1664.9.peg.913

MTIIWWAALIGLAVAIILILSRLNPVYALLMGTIVGALVGGASLAQTVDIIVTGAQSVMGTVIRVLAAGVLAGVMMESGAADAIARAIVEKMGDRFAILALTLATMIITGVGVFIPVAVLIVAPIALEVGQRMHISKLALLVALSGGGKAGNLISPNPNTIAAAKGFNLELSQVMVADFIPALFGLAMAVLLATLLRHKGTQVTEADIAQQQTTTKDIPSLKTAIVTPLLAIILLLINPMGNILHLKALQSFQVDAMYILPIAGIVGTLAMGKASHLLDYMKAGMNRMTDVVLILIGAGSIAGLIGASNLPSQVVSLVKASGISGTFLAPISGILMSAATASTSTGVILATGSFSKPILAFGIPALGAAVMVHAGAMVIDQLPHGNYFHVTANAMQMSFKERMQAVIWEACVGLTMTIVAVVMYGIL

>fig|1664.9.peg.914

MKIVLAPDSFKNSVTAIEASHAMRAGFEKVFPKATYVEVPMADGGEGTVQSMVDATGGRFLTAEVVNPLGQKVTAQYGILGDQETAVIEMAAASGIQFINEQTRNPLVTTTFGTGQLIEAAVKQGVKTIIIGLGGSATNDGGAGMAQALGVQLLNADQQSLGFGGGALADLASIDATGMLPALADVKIVIASDVTNPLIGEKGASAVFGPQKGATSEMVAQLDRNLAHYAAIIERDLGISVAQTPGAGAAGGLGAGLLAFTKAQLQPGVEIVIEKTQLKTAVADADIVVTGEGGIDFQTQYGKTPIGVAQAVKAVNPQATVIAIAGNIGEGTDVLYDLGIDSIFCSTPGVMSLEKALANTKANLTQTSMNIARLIQKTKTH

>fig|1664.9.peg.915

MELAQNARMNSLFEFYGALLTAKQHSYLSLYYGDDFSLGEIAEEYQVSRQAVYDNIRRTEKILEGYEAKLHLFQNYEQQNASADALQKYIQAQYPNDQQLAKLLADLLNLTEQ

>fig|1664.9.peg.916

MAFEGLTERLQGALSKLRRKGKVTEADVNQAMREIRLALLEADVNFKVVKDFIKVVKEKAIGTEVLESLSPAQQIVKIVDEELTKMMGETAVPLNKSPKIPTIIMMVGLQGAGKTTTAGKLATYLIKNEKARPLMIAADIYRPAAIDQLKTVGEQVGAPVFEMGTDVNPVEIVRQGLAQAALQKNDYVLIDTAGRLQIDEALMQELADINDLAHPNEILLTVDAMTGQAAVDVAEGFNSRLDITGVVLTKLDGDTRGGAALSIRAVTGKPIKFTGQGEKLNQLDVFYPDRMSSRILGMGDMLSLIEKAQEDYDEQKAVDMAEKIKENSFDFNDFLDQMDQLQNMGPLEDIMKMIPGMANNPQLKNIKMDPKDMTHMKAIVQSMTPQERENPDLLNPSRRRRLAAGAGRPIVEVNRMIKQFNQSKKMMNQMSKGNFNGMEGLMGNGIKGKMGKMAMNSMMKKQKKNKKKRLKNARRFKS

>fig|1664.9.peg.917

MSVKIRMKRMGSKKRPFYRIVVADSRSPRDGRFIQQVGYYNPLTEPVDLKLEEEVIMDWLQKGAQPSDTVRNLLSKQGIMQKYHEARFAKK

>fig|1664.9.peg.918

MTDVKELIIAIVQPLVEHPDDVKLTEHETERFMEFDLQVNPSDIGRIIGKQGRVAQSIRTIVYSVKTPYQKRVRLNIVDA

>fig|1664.9.peg.919

MPEKYYQVGKIVNTHGIRGEVRVIATTDFTEERFKKGTHLAVEMPNGLTPVTVSAMRQHKQFYLLQFEGLGNINDVELFKGHNLKIAASERDDQLEDDEYYYGDIIGLEVIEESEGTSYGKVSEIIDPGPNDVWVIKRRGRSDLLLPFLKSVIKKIDIEAGKAYVEVPEGLIDNED

>fig|1664.9.peg.920

MKIDVLSLFPDMVQNGLSQSIIGKAIDRDLIDLEVTDFRDFSVNKHNSVDDAPYGGGAGMLLRPQPIFEAMDQVNAKNPGHKRVILLDPAGVTFNQKVAEEFAQEDHLVFICGHYEGYDERIRTLVTDEVSLGDFVVTGGELGAMVMIDAISRLVPGVLGNEQSAVTDSFSTGLLEHPQYTRPPEYRGLKVPEVLMNGNHKLINQWRDKMSLKRTYERRPDLLENFDLTADQQKWLREIKQEAAEK

>fig|1664.9.peg.921

MNLLIQEITKSQLRSDMPNFRPGDSVRVHVRVVEGTRERIQLFEGVVIKRHGVGVSETYTVRKISSGVGVERTFPLHSPRVAKLEVTRHGRVRRAKLYYLRALRGKAARIKEARR

>fig|1664.9.peg.922

MNHWIYIVMYQANPLYYEKSKMIRAFSSEQRAKEYVSLLNETPYANQSLKEGHYTYQKLSLN

>fig|1664.9.peg.930

MGKKKKQGQLAKTLVEKILDKQKVAYTQFEFPTHEEHHVAQMQVDHLDIDEHLIYKTLVLSGKQTGPIVGVVPIDCHLDEKKLSKISGNKKIAMVPLKDLVATTGYEHGANTPVGIWEKAHFPIYIDQQAEKEPLIFVSSGKIGRSIQINPQDLSRVVSGTFADISED

>fig|1664.9.peg.934

MNKLKDTVTIMVEKNMLEFFEAELDDLQFSGDININWDKQSRAIELEFTLNVTKSQELAVEDQNGELNESDEISYEDAILFFDESKIDGFEYADNYLAVIPFSGRQGLNAQVGRGVFNYLQDLLDDGEDRLAAFVNDESDDETFILDWDDTVFQATVAQASDDLAKKYYTYPKY

>fig|1664.9.peg.935

MEWTKVTVATVNEAVEAIANILTEAGAEGIQIEDASINDTLVGDVAAVSAFFPETVFVPEKLPLIRQRVLQLTEFGLSIGEGSVNLQQVADDDWATAWKKYYQPTRLTRFLTVVPSWTDYQPTDEREALIRMDPGMAFGTGTHPTTHLSVQMLEMVLRGGEQLIDVGTGSGVLSIAAAHLGVQDIRAYDVDQVAVDAAVANFDLNPVTQGIIAKPNDLLHGITGAADVIVANMLPVVLVPLIPQVPALLKEGGHLLLAGIITEKEAVIRETLSANGLMVEESLHMGDWVGLITRLKTDED

>fig|1664.9.peg.936

MQRYFIEETVTVGQSLQLTGETAHHMLKVMRMQVGDQIEIVTPEELAFITQLQSADATQKQATVTVESAVETDVELPIETTIVCGLSKGDKTDWIVQKGTQLGAHRFIFCNSQFSVARWDAKKQVKKLARLQKIAQEAAEQAHRTHVPVIEWRASLTAIAEESADIKLVAYEESAKAGEHGQLVQSLQSAQPGQSLICVFGPEGGIAPKEIEALQAAGFLLAGLGPRILRAETAPLYLLSAISYVTELSTL

>fig|1664.9.peg.937

MDKIKNNRFLKQVWVRYFLVALLLAVVLPLVFGWLSISKTWRIGLLFMAINGCAAFFIGYRIQKTHAPWYNIFYLPVLFALMVVVRFADYNYWFVPIYFLLSYLGINTAYERRK

>fig|1664.9.peg.938

MSEEKVISQQEVIQLCQKYMNAEHLAFVQKAYDFAAYVHKEQVRQSGEPYIIHPIQVAGILAELKMDPATVASGYLHDVVEDTNITLGDVQEMFGQDVAVIVDGVTKLGKIKYKSHQEQLAENHRKMLLAMAKDLRVIMVKLADRLHNMRTLKHLKPEKQRRIANETLEIYAPLADRLGISKIKWELEDISLRYLNPQQYYRIVHLMNSKRTQREAYIQEAIEDIKDSVADLDMNYEIYGRPKHLYSIYRKMRDQHKQFEELYDLLAIRVVVDSIKDCYAVLGAIHTKWKPMPGRFKDYIAMPKANMYQSIHTTVIGPKGKPLEVQIRTQEMHNVAEYGVAAHWAYKEGVKEQIKIDESGRKLDLFREILEIQDASDDASEFMESVKGDIFSDRVYVFTPRGDVYELPKGSVPIDFAYLVHTEVGNHAVGAKINGKIVPLNYQLRNGDIVEMLTSTSATPSRDWIKLAFTARARNKVKRYFKKEDRDENVEKGRTAIEHLLLEQGHTVKAHLNKDSLAIILPHFNFNSEEELFAAVGFGEISAQAVLNRLTLKERREKEENEQKALEAEILSAKDSQKPGQKAAGSHITIKHEDGVIIEGVDNVLMRLSKCCNPVPGDDIVGYVTKGRGVSVHRRDCPNIVDRADMNSRLTEVSWENTSAKHDQLYDANLEVYGYNRGGLLNDVLQVLNAHSKQLNNVMGRIDHDKMADIHVTVGIRNIDHLERIMEAVKNIPDVYEVKRVQG

>fig|1664.9.peg.939

MKVVLQRVSQASVTIKEQVVGQINKGFLLLVGICDDDTEADLDYLVKKISQLRVFEDEAGKMNLALGQVNGAILSVSQFTLYASTKKGNRPSFTGAGQPDYAQKMYHLFNQKLAATGITVETGEFGADMQVALVNDGPVTILFDTRDN

>fig|1664.9.peg.943

MFMGEFHHTIDTKNRLIVPAKFREALGTEFVLTRGMDNCIFGYPLSEWEQLEEKLKQLPLAKKDARAFVRFFYSAAVQCTPDKQGRIMIPQALSTHASLEKECVLIGVSNRIEIWSQEKWASFSEEAEENFDDIAENMLDFDF

>fig|1664.9.peg.944

MTETFKHQSVLLKETVDALNVQPDGIYVDATLGGGGHSEYLLSQLTTGHLYSFDQDDHALESSRQRLAKYADAGQVTFIKSNFRFLKTALAEQGITKIDGILYDLGVSSPQFDDAQRGFSYKKEAPLDMRMDQGAELTAYTVVNEWSYQQLIKIFFRYGEEKFSKQVARKIEQQREIAPIETTIQLADLIKEAIPAPARRTGGHPAKRIFQAIRIAVNDELGAIEDSVEQAIPLVKVGGRISIITFQSLEDRLVKTMFKEQATLPDIPKGLPIMPGTEKTVLKLVNRKPILPTEDELTVNHRAHSAKLRVAEKQKEID

>fig|1664.9.peg.945

MMATNAARQLTTPESIPSQVQEPARQSAQQTTQKVAYSTLEKLAVTVIGIAFFAMLVSLLSTKIAVVNAQRTLENTTQDMSQTRAKNNDLKQEIGELTSSDRLDAFAKKNNLKLNENNIRNVAK

>fig|1664.9.peg.946

MLIIAAIFLIFMGRFAYIAATGHIAGVDLTKRSNKKYEGDSVIRAQRGTIYDSSGNVLAQDTNAYAVYAVLSHDYVTADKKPLYVADKKKTAKALSKYLPMSEANIYKQLTPKNKAVYQVEFGSAGQNLSLSIKRKIEKQHLTGIYFKESPTRLYPNGTFASHVVGIAQTPKGSEDSGSQDLVGVMGVEKYFNEQLTGQDGYKNLTKDSYGYSINQNKNKVKAAKNGQDVYLTLDSRMQSYLESLLSKVNKKYQPASMNAVLMNAKTGEILAASQRPTFNASTKKGIGDVWRDTLVEDGYEPGSVLKIATLSAAIDSGHYNPDQYYKSGSVLVSGKKISDWQTAGWGMIPLNQAFARSSNVGMVKLEQEMGAKTWKSYIEKFGFLKKTGIELPGETAGTLQYERPLDQATTSFGQGINVNVMQMLQMTSAIANNGTMIKPQILAGVGQEQQITPIKAGKPIKASTAKKVMQNMEDVVYKDYGTGQVYQIPGYKIAAKTGTAQITGPNGGYLTGASNYIFSVSGIAPADDPKYILYITMKQPGNMTDSAETILSQVFNPMMKRALEYTKEAEKDKQQQVKVASVLSQSTKDAQKKLEAQNLKTTLVGSGNLVVQQLPKAGSVILPNQRVLLMTNGAMTMPDVNGWSKNDLLKLAQLTGIDVDIKGSGYAYHQSLAVNSLLDGVKQIKVQLK

>fig|1664.9.peg.947

MLTGQFLIPLMSGFVITVIFMPLFIGYLRFKKEGQTIRDEGPKWHAKKNGTPTMGGLVFIVAAVISSIWVAIWLQQLTNSLWIALFILVLYGLLGFSDDFIKVFKKQNLGLRAWQKLAGQILGGAVFLAVYFHEGFSHALNIPLIGTISSSWFFSLFVIVWLVGFSNAVNLADGIDGLVAGLAIVSFATYTIIAFRQNQIDVAIFGLTIIGGLIGFLIFNHKPAQIFMGDVGSLALGGALAAMSILLHREFSLLLIGLVYVIETASVMLQVASFKLFHKRIFKMSPIHHHFEMSGWSEWRIDISFWVFSIICSAIYLLIF

>fig|1664.9.peg.948

MRTIATYQNQSVLVLGLGKSGVNATKLLLQLGAKVTVNDGQDQEDTPAVAELRALGATVITGSHPVALFEEGFHYLFKNPGIRYDNPMVAETIKREIPVLTEPELAYEVSEADWVSVTGSNGKTTTTTLIALMLNYQRAQGHAYAAGNIGIPLSEVAQKATAKDTMVTELSSFQLMGTTTVKPKVAVLTNIYEAHLDYHGTRENYVQAKMRIVQNQTASDYFVVNWDLPELRTLSQQTKAQVVPFSRLGTSEEGAYVKDGQLCFKGEVIMPVTDINVPGDHNVENALAALAAAKLMGQSNEAIIEVLTTFTGVKHRMQFVKEFAGRRFYNDSKATNMEATEVALKSFKQPIVLIAGGLDRGFTFEPLTDLLKAHVKAIILYGETKQLLAQTAKEAGIETIEIVDQLTEAVPAAYAASQEGDVILLSPACASWDQFKTFEERGDVYIDAVEQITE

>fig|1664.9.peg.949

MISGGGTGGHIYPALALIERLKQRGLLDAVLYVGTERGLESKIVPDQGIDFKTLEIQGFKRSMNLNGIKTNLKTIELFMSSIKSAKKMIKEFKPDVVIGTGGYVSGSLLYAASRLKVPTIIHEQNSAAGVTNKFLARFVDKVAISFESVSDQFPMHKVVLTGNPRAQQVAGMVPNERLSEFGLKTDSPTVMIFGGSRGAPSINKAFIDAVPLLNERDYQVLFVSGQVHYENVQAALANTTLNSNLAFVPYISNMPEVLPDLKAIVGRAGATSLAEITALGIPSILIPSPYVTNDHQTKNAQSLVKEDAAILIPEPELTGASLVKALDTLFETPEKQHAMAKAAKKSGIPDASDRIIEVIETII

>fig|1664.9.peg.950

MKNNKKQLGSHDPLQLLKVWRAYQIKRWKRQRRQRLKPGRPTITNQLPQLKKQRGKKIRWQLVVILGIFTLGSLTATYFISTKSDIQQLAVNGTKSVPDQQVINASGIQLGDNVLWQLMHHTKAKHNIQTKLPKIKQVSLQVSQMNHVAINVSEYKTVGYMFKHKQYYPILENGTILKTKMTQSLGNSPVYSHFKNDRYLKLGLKLYNDIPDSIQSAVSEIRLTAQNDNPYQVHLYMNDGNEVIGDLRTLAKKIKYYPVLVKQMDGKGKIDLEVGAYSKLFKQSKS

>fig|1664.9.peg.951

MENSKIYVGLDVGTTSIKVIVAESMNKQLNVIGVGSARSNGLSRGTIVDIDQAVSAIQQAVQQAEQKANIEITQVVAGIPANLLQIEECQGMIAVSDQSKEITSQDVQDVASAALVRNLPPEREILTVLPMQFTVDGFDGIKDPRGMMGVRLEMSGILFTAPKTMIHNLKKCIEKAGLELSQLVVNPLALGKLALSDGEQDFGAIIVDLGGGQSTAAVIHDHQLKFTAIDQEGGDHITKDISVVLNTSIENAEKAKRDYGNADSLVASEEEQIPIEVVGQTAPIQITEKKLAEIIEARMMQIFDRLKTALDKVNAFDMPGGIVMTGGVTALPGIVDLAQEVFNCPVKLYIPDQMGLRHPSFAQGLSLINYIANLTEVDWLVQSALGNVEQFEARQTATKSTAKTQATPKEAPQKKHKTESKHTFEGLKSFFSNFFE

>fig|1664.9.peg.952

MEYSLDAEQENNGAVIKVIGVGGAGGNAVNRMIDEGVKGVHFIAANTDVQALEDSKAETKIQLGPKLTRGLGAGSTPDIGQKAAEESEEVLAEALKGADLIFVTGGMGGGTGTGAAPVVAKVAKDLGALTVGVVTRPFTFEGPKRGKNAASGIAELKQHVDTLVIIANNRLLEIVDKKTPMLEAFHEADNVLRQGVQGISDLITSPGYVNLDFADVKTVMANQGSALMGIGSATGENRTAEATKKAISSPLLEVSIDGAEQVLLNITGGPDLSLFEAQDAAGIVQQAATSEVNLIFGTSINENLGDEVVVTVIATGIDNDGKTPKRPEAAQTTSQPVQPSTPKQTTNDDPFGNWDMRREPNPREQAKATEKFDEVEKKDFDIFKRTAQVDSDAVDTKQDNNDVPPFFKRRRK

>fig|1664.9.peg.953

MAGKFSFSNFFGMTEDEDYSTDLQGTKTTDEVSPTSRPDNIISMTAAGNAKMNKIVLCEPRIYSDAKKVGKHLLENKAVIVNFTRIEGAQASRIIDFLTGTVFAINGEIQRVGEQIFLCTPPNYEIDGNLSDIIDQNDFDSEVN

>fig|1664.9.peg.954

MQFVIYLLMFLMQATRIYVGIIFIYCLLTWIPGAMDSKLGQLLARLVEPFLGVFDRIIPAIGGIGFSAIIAGFVLYLVERGLGTIIQILAMRYY

>fig|1664.9.peg.955

MADNEAVYQHFRKSEAPFVAQVLDWIEQATTEYRAILTDFLDPRQAYILQTLIGEKGELKYHFSGGYNAAERQRALIAPTYFEPEEADFEIQLFEIRYPIKFAQLSHGKILGTLVNAGIDRNVFGDIMTDGQRWQFFVTTKMADYVVNQITKIGKVTVHLEEQAYTELIIPRDAWQFEQLLVSSFRLDTIIAAVYNMSRQRAKELVQGNKVKLNWQAFDKPDFELDLLDIISVRGYGRIQLKSIDGQTRKEKYRVTLGILRK

>fig|1664.9.peg.956

MVLTPLDIHNKEFGNKMRGYNPDQVNDFLDQVIKDYESVLNENDALKAELKSSDEKVTYFNELKDALNQSIIVAQEAADKVKENAKREAEIIQTETEKNSKSMLNDATEKANHIIEDATERAKQIAIETDDLKKQTRVFRQRLQVMLESQLEVVKSPEWTDLLSKDDLAGHEALSAEIDLTDLDSKKHNLVDSETVVKPWDDITTDDFSDQTTHTIVFPDDKLPEVADEEAPEEPVIKADTLDDEIQSEETHNDYGYNPIEEDDSEPLTDNNKN

>fig|1664.9.peg.957

MRIKETLNLGKTAFPMRAGLPNREIDWQKGWADNNLYQQRQKLNEGKPSFVLHDGPPFANGNIHMGHALNKTSKDIIVRYKSMNGFRAPFVPGWDTHGLPIEQALAKKGIKRKEMSLVDYRKLCYDYAMEQVNTQRQDFKRLGISADWDNPYITLTADFEAEEIRVFGEMAKKGYIYKGKKPVYWSPSSESTLAEAEIEYKDIKSPSMYVAFNVVDGKDLLDADTKFIIWTTTPWTIPANLGIAVNPAFDYVQVLADGQKYVVAAERLNKMTDLLGWESVEILKTFKGADMELMTARHPLYDRESLVILGNHVTLETGTGLVHTAPGHGEDDYNAGTKYKLPVLSVVDSKGIMTEDAPGFEGVYYDKANPMVTEALEKNGSLLKLDFFTHSYPHDWRTKKPVIFRATAQWFASIDAFRDQILAQIEKVEFMPEWGKTRLYNMIRDRGDWVISRQRAWGVPLPIFYAEDGTEIITPETIERVAQLFAEHGSNVWFEWDAKDLLPAGFTHPGSPNGEFTKEKDIMDVWFDSGSSHQAVLAARDELTYPADLILEGSDQYRGWFNSSLITSVAVGEVSPYKAVISQGFVLDGNGRKMSKSLGNTILPEKIIKQMGADIVRLWVASVDASSDVKVTMENFQQVSEAYRKIRNTMRFMIANTTDFDPAKDTVDYAELGSVDKFMLVRLNAIIESCKAAYDAYDFATVYKTINMFLTNELSAFYLDFAKDVVYIDGQNDAPRRNMQTVFYAVAVALTKLLTPILPHTAEEIWSYLHEPEEFVQLAEMPEVAHFAGEEDLVATWNAFMGIRDDVLKALETARMDKVIGKSLEAAVTLYPNEANAALLASLDADVKQLLIVSQLTIADQAVEAPAEATQFDGVAVSVAHAEGDVCDRCRMIKTDVGSDDKFPMLCARCAAIVTANYPEAVAEGLEK

>fig|1664.9.peg.962

MRKKLVITEKPSVAKELAKVLGTNQKTKTYFEGDQYVITWAFGHLLSLQLPEDIKKEWQQWRLEDLPIIPKHIGLKPLPKTGGQLKAISHLAHRQDISGAVIATDSGREGEAVARYILEWIKFDKPVERLWISSQTTKAIKDGFANLKPAKQFDNLYASALARGHADWLIGLNVTRALTTKYEDNLSAGRVQTPTLAFVNETAQQISRFKPEKYYTIQLKVGQEQATLQMAKPQGLKDRAQAETLVAKLKQAQGKVGSVKTSQKSQAAPLPYDLTELQQVANAMYGLSAKKTLSLIQSLYEIHKVVSYPRTDSKYLSTDIEGTLKERLSALVKFDPRAKQYLGQGAKVKQPAVFNDRKVTDHYALIPTEEPVRAEKLSTDELRMYRLIVERFMGLFADQYVTAVTKATVQFGDHTFTFKQTKVVQQGWHADSKETPTTSVDWQEGAVVPADFSIKEQLTSAPKPLTEGTLLGKMEHYGLGTPATRAEIIEKLVRSELLERRGHSLAVTPKGQQLLKLVNKTLVSPELTGKWENSLEAIARGQENQKAFITEIEAATKTLVSEIKKSETEYHDFAITNKKCPECDHPLKERNTRDGKVYVCTNCHYRRRKDPKVSNHRCPQCHKKMVIIEGQAGAYFRCLNDGTTEKMLDKKDCKKKISKHEERRLIQKVNQEEEPAESPLAAALRKAMEK

>fig|1664.9.peg.963

MKTKKMRRLLLTVAILAIGYLGGRNNILQDITQKIPAITQSTIQQAQVSPDYQAIANRDFENGSAAYLAINNDKSQLTSADWTSEKIDYGQLDQLNRTTSATAYLSSRNLGKSEGRSRQVWNPTGWHNQPLVVNGRRITPQNRGHLIAYTISFNFDQNGHFQSGQPGSLDNPLNLATQTEYSNQKTMQIFEEKVRNALAANKKVIYKVTTVFRGNELMPRGYWVQAISTDGTLNFNDYVWNIEPGVAFDYATGRSHLDANIQVKDNHPIVKGTENTVNNIKNRF

>fig|1664.9.peg.964

MATIKDIAQLAKVSNATVSRVLNYDQSLSVSDETRQRIFEAAESLSYTKNTKKNNNHRGRIAIVEWYTATEELEDLYYLSIRLGVEKKLQELGFEVTRFFQNDPFDTLFDAAGVIVIGKYSLGQITRLKKLNRNLVFIDMNTLDYEISCVMTDFKTPVKAVVDHFMASGLTKIGMLTGQESTSDNVKLGTDPRSTHFKHYLKKQNNFHPEWLLTGDFTPESGYQLITQLLHEQADNLPEALFIANDAMAVGALRALHEAAIKVPEQLSLISFNDTSICQYTFPTLSSVKVYTERMGQSAAELLIKQLDAEQVIPQVITIGTSLTLRNSSK

>fig|1664.9.peg.965

MNMTELNTAFKAAYNKEPEASYFAPGRINLIGEHTDYNGGHVFPTAITLGTYAVVAKREDQTINLLSGNFEDAGVISFDLSDLSYQKAHNWANYPKGMIVYLQEQGYTIDHGLDIYLKGNIPNGAGLSSSASIELLMGVILEDQFNLDIDRVDLVKTGMMVENKFIGVNSGIMDQFAVGMSKANHAILLDTNTLDYDLVPIDLQDNVIIIMNTNKRRELADSKYNERRSECEKALAILQTKNDINSLGDLDNETFDLQTYMLQDENLLKRARHAVSENQRTMKAREALKNNDLERFGKLVNASHVSLQFDYEVTGIELDTLVQSAWQQPGVLGARMTGAGFGGCAIAIVAKDQVDNFEENVAKAYTDKIGYAPSFYVAEIADGAKKLG

>fig|1664.9.peg.966

MSILVLGGAGYIGSHTVDQLIERGYDVAVVDNLVTGHKAAINAKARFYKGDVRDKEFMRNVFQQEDVSGVIHFAAFSIVPESMQSPLKYFDNNTYGMTALLEVMNEFGVKRIVFSSTAATYGEPKAIPIKESDPQVPTNPYGESKLMMETMMKWADKAYGIKFVALRYFNVVGAKPDGSIGEDHHPETHLLPIVLQVAAGKRDQLSIFGDDYDTPDGTNVRDYVHVLDLADAHILAFEYLKDGHDSNAFNLGSSTGFSNMEIVEAARKVTGKAIPVTMAPRRAGDPSTLIAASDKARETLGWVPKYDNMEAIIETAWNWHLNHPNGYADR

>fig|1664.9.peg.967

MQARELIQKFITKVSTFPETTYTAEDEQYLFNVVLDLVGEGDETPVVAQETTTLIDLKEALVQLAVDHQRIQDLAAERDILGAKLMDLVTPLPSAVNKRFWTLYEKDPEKALAYFYELSQANDYIKVAAIAKNIAYDVPTKYGDLEITINLSKPEKDPKQIALAKTMKQTGYPKCQLCLENEGYAGRLDFPARRNHRIVRFDLEQQTWGFQYSPYAYFNEHSIFLDGKHEPMVIDQTTFKNLLQIVKQFPGYFAGSNADLPIVGGSILTHEHYQGGRHDFPMAKAPISTPLQFAGYDDIQAGIVDWPMSVIRLTGSDAKRIETLAADILTKWQHYSDPEVQVLAQEADGTPHHTITPIARMRGDQYEIDLVLRDNQTSPEHPDGIYHPHADVQHIKKENIGLIEVMGLAILPPRLKSEMAEVEKYLCDQPNEMAAYHKTWADQIKADHSAVTPANVTALVQNEVGKVFMRVLEDAGVFKRDAKGQAAFIKFTERVNNA

>fig|1664.9.peg.968

MKYWRQEFGTINGQTVWQHWLENSQGYQLAVIDYGATITNLVMPDKAGQFKNVVIGYDNLADYLKQEAYFGATVGRVAGRIGKGAFTLDGHDYQLPLNNGENTNHGGPNSFESQVWQASVEEQADAISVTFKLTSPDGANGFPGNLSVATTYTLNEKNEWLVDYQATSDQTTLFNPTCHVYLNLTGDFDQLVDGHQLQIDSDRFGEIQPDGLPTGQLLPVDQTVFDLRQPRAIADAFASQETQNQLVNGYDHPFLLNKNADRNDAVLIEPTSGRSLTINTEGNAIVIYTANGFGSEPNLTDKAIQPHEAVAIEAQMMPDAIHHADFGNVILKANEQYHRQTCYKLN

>fig|1664.9.peg.969

MEKGTVKWFNNEKGFGFISVEGQDDIFVHFSAIQDEGFKSLEEGQAVEFDIVEGTRGAQAANVKKL

>fig|1664.9.peg.970

MQKNIIDELTWRDAINQQTDADGLRELTEKKSISLYCGVDPTGDSMHIGHLIPFMMMKRFQLAGHHPYIVIGGGTGSIGDPSGRKSERQLQTMEMVQHNVEALSGQMKRLFGEDANVSMVNNYDWLSKISLLDFLRDYGKLFNINTMLAKDVVASRLDVGISFTEFTYQILQSVDFMHLYKAHDVQLQIGGADQWGNITSGIDMIHKIEGSETEVYGLTIPLMLKADGTKFGKTAGGAIWLDPEKTTPYEFYQFWLNQDDRDVVKYLKYFTFLDEAEINELAKTVETAPEKREAQRRLAEEVTRFVHGQAELEGAQKITEVLFSGDIKALDVKEAEQAFQKAPTVEITAEKKNIVDFLVEAGIESSKRQAREDVQNGAITINGDRLRETTLEIDPSENFEGKFVIVRRGKKKYFLARVK

>fig|1664.9.peg.971

MALSYADNPQVQKNRWRILTAVGLFTFMATLDGSIVNIALPVMSRDLKIPMNQSEWIVSIYLIMICALLLLFGKVGDAFGKVKVFKIGTFLFVLGSLLCGLLLGARVVQALGASMTMSNNNGIITEVFPLKERGRALGLTGSFVALGSIAGPGIGGLILAHFSWGYIFWINVPIGILTIIFGHFILPKDIIKTHEKIDYAGFVSFAALIVSLFLGIFIGQQIGFTSPIILTAFVVALISAGIFVWIEQHVEQPLLSFRLFKNIDFSLSLLCAFLIFIVNFFFNVISPFYLENALGLAPNLAGYSLMIFPIVQVVVAPIAGSISDKIGPELLTFVGLILISISQVGYMLMNLQTPIWLFMFFVGLVGLGNGIFQAPNNTIVMSSVGVQDLGVAGGINALARNLGMVFGISLSTTVLYAAMSRNYGQKVTGYIPGHADVFIAGMYDAFMVALVICLIATALTGWRMLKHRRMVVKN

>fig|1664.9.peg.1193

MADTPHFYIILNQVAGTGQAQKIWPQIETALKQRGISYELQISSYPGHTTRIAYQFARFKRTNQVLLIVGGDGTLNQAINGVQSAGKHDIPIAYLPCGSGNDFARGIGLNSDPLQALEQILAATAPVNIDLGVYHDALKNETGYFVNNVGIGFDASVVSITNNSKSKRFLNKCHLGSLSYVFSLIQAFTSQDAFQVTVKANGSVKNFSRGFLVTTTNHPYFGSGVPIMPSARVDDQKLDLVVIEKLNVFFFFCYLFIMMLTGKHTRYKTVHHFRTDDLVIRTQTLEFGQVDGEEMGSRAFDMHFKVTSQPFWLTTTKTEQFK

>fig|1664.9.peg.1194

MKLNIKNNETAVFAVGGLGEIGKNMYGVQFQDEIIVIDAGIKFPEDELLGIDYVISDYQYLQKNQQKIKALVITHGHEDHIGGLPFFLQQVPNIPVYAGPLALALIRGKLEEHGLLRSTELHEINEDTVLKFRKTSVDFFRTTHSIPDTLGVAIHTPQGVVVETGDFKFDLTPVGNQPSPNLQKMARLGEEGVLCLMSDSTNAEVPNFTKSERFVGDSIRHIVERIEGRIIFATFASNISRVSQATDAAIANGRKIAVFGRSMEAAVVNGRELGYLNIPDEALVDASQLNSLPADKVMIICTGSQGEPMAALSRIANGTHRQISIQPGDTVVFSSNPIPGNTTSVNHVINKLSEAGAEVIHGKVNNIHTSGHGGQEEQKLMLRLMKPKFFMPCHGEYRMQKIHTELAQQCGVEPDNCFILENGDVLALTADSAHVAGHFSADDVYVDGNGIGDIGNVVLRDRKMLSEEGLVVVVATINFGEKNILAGPDILSRGFVYMRESGELISEAQRRVFRMIVNTFNANDKVTENQVREAIIDGLQDFLFEKTERHPMILPMIIQSNE

>fig|1664.9.peg.1195

MIYKVLFQADKTQSPLREATKSLYLEANSAVEARQLVEDNTPYNIEFVQELTGEHLAYEQESEDFKLTEF

>fig|1664.9.peg.1196

MLIWLLLLVLIGLVGGLAWFIHRIRRQRAMRILQSSSQETVDEIVNIAYQRLLSVQPQFYTKQATLTANLPTEVWGGNVMLFEYRVNVLQTDVSLKPITETLAQTLNEVADERRIASVNPKYPALVITDAWLLDQEYHFDVAFIVNRETIEYVKDMARVMD

>fig|1664.9.peg.1197

MKLMWRYTIRYKKLLFWNFICVFGFILIELGLPTLLGQIIDKGINRHTVGPVWQYGTLMVVISLIGLVGLIGLAYTGSRLTTNLTRDIRNDIFKKTMQFSHHEYGEFGVSSLITRTTNDAFQVTQFMVQVLRTGFMTPMMIVASAFMIVRTSLSLSWIVFAAIPILLVGVVIIGWLSEPMSAEQQKNLDAINLNLRENLTGIRVIRAFVREKFQSMRFRKVNEAYSQSSIKLFTLVALAQPGFSFIFNIVFVLIIWQGAVQINTGHLAVGTLIAFIEYIFHVLFSFMLFASVFMMYPRAAVSAERIEKVLNSPVDITDDDQGIQTTTTQGELAFENVSFAYPGPTESPVIRDVSFKAHPGETVAFIGSTGSGKSTLIQLIPRFFDVTRGQVMIDGYDVRDYQLKALRQKIGFIPQKAVLFTGTIADNLRYGNPEATDAELWDALTIAQSKTFVAEKPEGLQTYLAEGGNNLSGGQKQRLAIARAIVRRPDIYVFDDSFSALDYQTDAALRGALKSITKDATVLIVAQRVGTIMNADRIVVLNEGQVVGIGTHKELLKTNEVYRAIAASQLSKEELNEEY

>fig|1664.9.peg.1198

MKNIKVLKRLMRYIGPYKITFWSAITATVISVLANALWPFIMGLAVTEISQNLAQHVAVNFSYITKVIAMILSFALLYQTMMFLGSFLMTRAVQHAMRDLRRDINLQMNALPVRFFDSQQQGNILSRVTNDVDAITNALQQSLIQVVTSILGIIMAVVMMLTINLWMALLSLIMIPTSLLISKAIIKKSQVYFQGQQNTLGEMNGYVQENYGGFSVLKLYGQEERAVDEFAAITDRLTEFGFKAAFISGIMMPLVGLTTNLTYAGMATLGGYYVLHGVITVGNLQAFIQYIWQINQPISQITQLSGVMQAAAAATQRVFEILDEPIEPTDQETAELSADVQGKVEFKDVSFSYDPAKPLIRDLSFTANPGDKIAIVGPTGAGKTTMINLLMRFYDVSSGQILIDGVDTRQLSKQTVRSLFGMVLQDAWLYHTTITENIRFGKLDATEYEVVDAAKTANVDHFIHTLPNGYQMTIDEEADNVSLGQKQLLTIARAVIANPKILILDEATSSVDTRLESLLQKAMEKVMQGRTSFVIAHRLSTIRDANLILVMDHGQIVEKGTHDDLLAANGVYAKLYNSQFAEAKLNA

>fig|1664.9.peg.1199

MILMKDIIREGNPTLREIAQPVSFPLSDEDRQLAADMMTFLENSQDPEIAAKYQLRAGVGLAAPQVDVSKQMSAVLVPGPEGEAPILKDVIINPKIISHSVQDAALAEGEGCLSVDREVPGYVPRHDRITLRYQDVEGVSHKIRLKNYPAIVCQHEIDHLNGILFFDHINKENPFAAPDDMIILE

>fig|1664.9.peg.1201

MANNKAAVNFDKLLGDESQDFKTVQILDETGQVVNPDIMPDLSDEQLVELMKQMVWSRVLDQRATALNRQGRLGFYAPTAGQEASQLASNFAMNKDDFLFPGYRDVPQLVQHGLPLSQAFLWSRGHIEGNKYPESLKAMPPQIIIGAQYIQAMGVAVGMKKRQSKNAVYVYTGDGGTSQGDFYEGINFAGAFKAPAIFVVQNNGFAISVPREKQTAAVTLAQKGVAAGIPAIQVDGMDPLAVYEVMKEARDFTTAGNGPVLIETLTYRYGPHTLSGDDPTRYRTKETDDIWLKRDPLVRMRRFLTNKGLWSEDQENELIDQVKAAIKEAINEADAIPKQKVTDFLKVTFEEQPQNIQEQITEYSAKESN

>fig|1664.9.peg.1202

MAQKTMIQAITNALDLELASDENVLIFGEDVGKNGGVFRATEGLQAKHGEERVFDTPLAESGIGGLSIGLALEGFRPVPEIQFFGFVFETLDSIAGQMSRTRYRMGGTRNMPITIRAPFGGGVHTPEMHSDNFEGMITQIPGIRVVVPSNPYDAKGLLIAAIRSNDPVLYLEHMKLYRSFREEVPDESYTLPLDKAAVTLEGSDVSIITYGAMVREAKKAAENLAKDNISVEIIDLRTIAPLDIKTIIASVEKTGRVVIVQEAQKQAGVGAQVASEISERAVLSLEAPIGRVSASDTPFPFGQAESTWLPNATDIENKVKEVINF

>fig|1664.9.peg.1203

MAYQFKLPDIGEGIAEGEIQKWAVAEGDTIAEDDTLLEVQNDKSVEEIPSPVSGKIVKILVGEGEVATVGQVLVEIDAPGVEGNDAPTAETTTPAAEQPAAPAASEGVYQFKLPDIGEGIAEGEIQKWAVAEGDTIAEDDTLLEVQNDKSVEEIPSPVSGKIVKILVGEGEVATVGQVLVEIDAPGHNTATASAPVATTPAPQAAETPVATNNSSDTSVVAISDPNRKVLAMPSVRQFARENNVDISQVPATGKHGRTTKEDIQNFMQNGTALVASTTTTDTKSAPVTTATPEPAVAVKPYESATPELETREKMSPTRKAIAKAMVTSKHIAPHVTLFDEVEVSKLMTHRKKFKDVAAKKDIKLTFLPYIVKALVTVLRDFPTLNASIDDTTSEIVYKHYINVGIATDTDHGLYVPNIKDADSKSIFAIAKEIGENTQKALDNKLKPAEMSGGSMTISNIGSIGGGWFTPVVNYPEVAILGVGRIGTEPIVNEDGELAVGKVLKLSLSFDHRLIDGGTAQRAMNELKELLADPELLLMEG

>fig|1664.9.peg.1204

MVVGDFAIELETVVIGSGPGGYVAAIRAAEMGQKVTVIERENIGGICLNVGCIPSKALISAGHRLQEAKNSEVFGVSTTGATLDFAKTQDWKQHQVVEKLTGGISMLFKKHKIDVLDGSAFLVDEHSLRVIKEESAQTYSFKNLIIATGSRPIEIKGFKFNKRVIDSTGGLNLTEVPKELVVIGGGYIGSELAGAYANLGAHVTILEGTDSILPNFEKDMVQLVTKNFKTKGVDVITGAMAKESIETDQNVTVKYEVGGKEEQLVADYVMVTVGRRPNTDDMGLEQAGIKMADRGLIEVDQQGQTNIKGIYAIGDIVSGAALAHKASYEAKIAAEAIAGKKVIVDYKAIPAVCYTDPELATTGLTVAEAKEKGYNAKGFKFPFAANGRALSLAETDGFVRLVTNTDDNTLLGAQVAGVGASDLISELTLAIESGMNAEDLALTIHPHPTLSEAIMDDAELALGLPIHI

>fig|1664.9.peg.1206

MEQEALYQLSRSLQKWVLEAAAETRKKMTQPLEIMTKSSRNDLVTNVDCANQAFFAEQIKAEYPMAQLVGEEGANVGAGDWHNGLTFFVDPIDGTMNFVKQQENFAIMIGVYEDGQALFGIIYDVMADQLFWGGPQLGIFRNEQALEQAADQPLSEGLICVNTRMLVTNERQLQQVFVQSSGVRIYGSAGITFAQLLRGNAQAYISKLAPWDIAAGRVLAETLGFQILNFDGQPISMVAKEGMIIAPKQAMQEIIAIMAHSKTAE

>fig|1664.9.peg.1207

MKKREDIRNIAIIAHVDHGKTTLVNEMLKQSDTLDGHAEISDRAMDTNDIEKERGITILSKNTAVKYNGKQINILDTPGHADFGGEVERIMKMVDGVLLVVDAFEGTMPQTRFVLKKALDQHLTPIVVINKIDRPGSRPEEVVDEVLELFIELGADEDQLEFPVVYASAINGTSSLESDLDTQAHTMVPIFDTILDTIPAPVDNSDEPLQFQVAMLDYNDYVGRIGIGRVSRGTIKIGDQVSVLKLDGSAKNFRVTKIFGFIGLDRVEIQEAKAGDLIAVSGMEDIYVGETVTPVDHQEALPILRIDEPTLQMTFAANDSPFVGREGDFVTGRKIEERLRHQLHTDVSLRVEDTDKAGAWIVSGRGELHLSILVEEMRREGFELQLSRPEVIYRDIDGVTCEPFEMVQIDTPDQYTGSVIDSMAQRKGEMQNMESDANNQTRMTFLAPSRGLIGYSTEFLTMTGGYGIMNHTFEKYMPVIKNWEPGRRNGALVSINAGSSTTYSLQSVEDRGQLFIGAGVDVYEGMIVGQNSRDTDIAVNVTKGKNLTNTRASGKDHSAAIKTPKEMSLEESIEFLNDDEYCEVTPTSVRLRKKILDTGMRTKADKKRKRG

>fig|1664.9.peg.1209

MWHKLRDKAQYLDYWLLIPYVILCAIGALMVYSASSDLMSIHGAKPDAYFTKQLIYIGLGFGLLLFSFFLKLNLLRKPKFLIGVTVVILLALFYLLVLSRIRPDAAINGATAWINLGPFSIQPAEFAKLLIVLYLANMLSKREKNLSEHWRDNVKLFSAPVILVAVIIAFVLVQPDTGGAAILGIILLVLLFASGISFWWGISIISATLAVITAAIAGLSRLNLTTGVNYRFNRILAFLEPFKLENMGGSQLVNSYYAINNGGWFGMGLGNSIQKRGYLPEPYTDFILSITTEELGVIGALFILGLLFLLIFRIFTIGIRARQTYNALICYGIGTIIFVQSLFNIGGLLGLLPITGVTLPFISYGGSSMLVLATGLGLVLNVSAQEKKALKMKQS

>fig|1664.9.peg.1211

MALEVTQRQGIVVWLYSLRQVKQLRRYGLVYYTSKRMKYVYLYVDADQAPAVIERLKKLHYVKRVTRSQRPMLDMEFGAVAELANQETATKAALKE

>fig|1664.9.peg.1212

MRIIAGEFGGRRLKAVPGMKTRPTTDKVKEAMFSMLGQFFDGGQALDLYAGSGGLAIEAVSRGMDHAYLIDRQYAAIQTIQDNVAVTKMPECFTIWKMPAEKALLKLADAKEQFDLILLDPPYKQQQMLVELQQFVKMDLLKPGAIILCETDTSLTYPEEIPHYTLKRQQNYGITEVAIFEFIG

>fig|1664.9.peg.1213

MTERIALFPGSFDPFTKGHLDTVERASRLFDRVIIAVMTNAAKKPLFDGPTKVALIETVIADLDNVSVVAQPKTLTANFAQAVGARYLIRGIRNANDFEYERDIAALNQTQDAQLETVLLLAKQEFSFISSSMVKEIAAFGGQVDQLVPPAVAVALQEKLKHGRD

>fig|1664.9.peg.1214

MPKNKKLTKRLIVIVVLIIVAVVCLMPTGYYIEGAGTAENTSQYVKVDGKHDHQKGHFMLTTVGVRGPVTPLQLLLSKTQPFTEIESRDELMGNEDTEAYEQIQKYYMDSSINAAVEAAYKKADKAYHTKYLGVYVMSMLGNSTFKDDLAVGDTITAIDHHQFKNANAFVKYVQGLKVGQKVTVSYDHGQKHQTATHRLIKLPRTHHPGLGITLTDHSKIETDIPVKIDAGDIGGPSAGLMFTLQVYNQLTKQNLQKGRTIAGTGTMAADGTVGAIGGIDKKVLVASQEGATVFFAPNDPVTKAILKVDPDYQNNYAIAKNAAKKIHTKMKIVPVKTLDDALTYLKNH

>fig|1664.9.peg.1215

MMWQDYWQRYRRFWWVPCLVLLGIIAGGFYWHQSQQKQKVTQNEQLLATSASKTKPATKKTHQAATKKKGGFVDIKGAVVYPGIYPITVSETRLFEVLKNAGGPTDAADTKRLNLAQKLTDQLIIYIPKKGEVVTDQELIQAPQQIATTSERTQEQASTGTSANASSTSDTAKIDLNQADSTQLQTLNGIGPKKAEQIIAYRDEQGKFKQIEDLQKVGGIGPKTFEQLQSQICVN

>fig|1664.9.peg.1216

MANKRMPWNQYFMLQSVLLSMRSTCPRLFVGALVIRNRRIIAGGYNGSVAHEDHCIDVGCLMRDGHCLRTIHAEMNALTQCAKFGVQTEGAEIYVTHFPCLQCTKLIIQAGISKIHYQKDYHNDPYALKLIEKSGIELDQVNLSVDDAHTLVNFLENEQATE

>fig|1664.9.peg.1217

MKNNWIYPVICLISLNSLMLGGQSWLTWSLLLLAFVKLISLRQTQITIMTIGLCLVYGGRFWISYHSVTQPQQPVAQYFKVQPDAIQVAGDSVQLTAIGQQDGQTIKAYYRCQTFAEKRRWQQVRHPVLFYGEKDFKRIQGATNQNEFDYARFLAQQKRCFYQLTLAKKTTFALRRPSSGLDWLHYWRQTCALYLRKLPTALRFHAQTLLLGLREAQVDHYQVVLGHLGIIHLLSLSGLHVFYLVQVIRWCATYCRIPREWLNCMLFGLLPVYALLVGGTTSISRAIALILSRLLCKQLGIRQSRLDSWSLVLLVNLFWQPYLLHSMGGILSYLMAFALIYIGEGSTFKVAYWLSLLSLPVCLRFNYRWHVLTIMMNALVTPIYLPIVLGLVIVASVMLPVSNLVVNGCEWLLQLIYKGLGLVVQIPHAMITFGKIPLLPLLLIVTVSLLLIDGQAISNQWRKRLKRTLVSLYLASFLAIHFNPTGRVVMFDIGQGDSLLIQTPFNRHQLLIDTGGRLALPQAAWQRRAQVSRAEKVTVNYLYSQGVDHIDAVALSHQDADHIGDLNQILKQIRVDRIICAAGLPQNRQFQRQIRPFLATVQIEPYLAGAAFKVGSQKFNVLAPTKPGKGENSDSLVLQAQIGGASWLFTGDLEQEGERAIIERYPQLTVDYLKVGHHGSKTASDPTVIKKLHLKGALISTGRENRYGHPHQETLTTLQAAKVPYWLTAQQGMLTWAYGPGQSEKLQTTIKDSEK

>fig|1664.9.peg.1218

MTIEQLQKELQQNQLAPIYLVLGQESALIEKARQLFKHYLPEEEQTMNFASYDLATTDVANALDDAESAPFFGERRIVFMQNPYFLTGEKVKNKLDQDLVRFQAYLEKPQPTTSVVLFAPYPKLDERKKIVKQLKKVASLVSVEKVNERQVQQLVTAELKQQGMQIESEALALLISKTNSDYSQIMAELPKLMLYAQQTKQITTAAVAELVSSSIEDNVFSLVPLVLQKKVGPVLTMYHELLLQKEEPLKINAILIGQFRLLLQVKILFEKGYSQGNLASVLKIHPYRIKLAMQQIKHFSKQTLAQAYLGLEEIEVKIKTGQGQPELLFELFMLQFAAKKVA

>fig|1664.9.peg.1219

MPQIKSAMKRVKTIEKANNRNASQLSTMRSAIKKFKAAQAAGNEEAADLLKAATRAIDMASTKGLIHANKAGRDKSRLNKMMAK

>fig|1664.9.peg.1220

MAISQARKNEIINEYARHEGDTGSAEVQIAVLTAEINSLNEHLSVHKKDHHSYVGQMKKIGHRRNLLRYLRDNDIQRYRELIKSLGLRR

>fig|1664.9.peg.1221

MSNIKIMPLGGVRENGKNLNVVEVNDEIYILDCGLKYPETELLGIDIVIPDMTYLEENAERVAGIFLTHGHADAIGALPYFLKGHDVPVFGSELTIALAKITVSSEPKLRHFNNFHVINANTAIDFEQATVSFFKTTHTIPGSLGIVVKTDEGQIVYTGDFKFDQSALPMYQTDYARLTEIGQQGVLALLSDSANAESPFETANERDIAESILETFKYQNGRIIVASVASNILRIQQVIDAAYKTNRKVVLTGRDLEKIVRTAMRLEMLTLPDEDILVQPKALSTLAPEETVILETGRMGEPIKALQKMATSHHRLIHIEQGDLVYLTTSPSHAMETILAKTKDMIYRAGGEVKAVGDDLHASGHANKNDLQLMLNLLHPQFLMPVQGEYRQMAAHADIAHETGMDYKNIFIVGKGDIVAYDNDKMHVGGQVDATDVMIDGSGIGDIGNIVLRDRKLLSEDGIFIAVVTIDRKNKKIIAEPKVTARGFVYVKTSKDLMAESSEIIKQAVQNNLDNKEFDWSHLKQDVRENLSHYLYEQTKRRPVILPVIMEINQNHRRQKKDTKPAVEKKQD

>fig|1664.9.peg.1222

MGELTALPNQFENLVRLGKVALNQRQWPEAIAHLEAAYDLQQTTALNLLLVSVLVAAGDNQRALQLAFEKKDFYTNETDLRLLYNGLLIKNQHFIAANELWSAVEATDAFEQRTKQKGQALLKTAEKAYQATEQQAIKKRQEQLYNITTGDFQDQQRRLDAAELLPLADYLQGVSPILVNPYCHPFVKTTILETLVRLQYSESVTINWFDQVRQVTPSELELMTNRPVLKLIQKTIQNILGQQDPIILEAALAECQMTFAYLYPFEETVIDNPEEWGQALIQQLGVFIFEPTVPISEKTIQWLKKLQELTEELVF

>fig|1664.9.peg.1223

MAEKAHYERTKPHVNIGTIGHVDHGKTTLTAAITKMLADKGLAEAQDYANIDAAPEERERGITINTAHVEYETENRHYAHIDAPGHADYVKNMITGAAQMDGAILVVAATDGPMPQTREHILLAHQVGVDYIIVFLNKTDLVDDDELTDLVEMEVRELLSEYDFPGDDIPVIRGSALGALNGNPDDVKAVEELMATVDEYVPTPERDTDKPFLMPVEDVFTITGRGTVASGRIDRGQVTVGDEVEIIGLKEEITKTTVTGLEMFRKTLDQGQAGDNIGALLRGIDRESIERGQVLAKPGSIQTHKNFKGEVYILSKDEGGRHTPFFSNYRPQFFFHTTDVTGVIELPEGVEMVMPGDNVTFTVELISPVAIEKGLKFTVREGGRTVGAGVVSEIID

>fig|1664.9.peg.1224

MSAKWEKKGANDGELTFEIDLDKISEGLDVAFKRVRKNINTPGFRKGKMPRQIFNKMYGEEALYEDALNAVLPEAYEAAVAEAGIDPVDQPQINVEKMEKGEAWVLTAQVTVKPEVKLGDYKGLEVPKQSRRITIKDVEQELETRRERQAELVLKEDAAAENGDTVVIDYAGSVDGVPFDGGQADNYSLELGSNSFIPGFEDQLVGHKAEEDVDVTVTFPKDYQAEELAGKEAIFKVKIHEVKGKELPELDDEFAKDIDEDVDSLDELKEKIREELKQQKNDAADAAIQDTAVAKATENATIPELPQAMIDEEINSQLQQYLGNMQQQGINPEMYYQITGTTEDDLKKQFAGDADKRVKTSLVLEAVVEAEKIEATDEEVAAELKSLAEQYNMEESAVRSVLSDDMLKHDIGVKKAIEVIADSAVEVKDAK

>fig|1664.9.peg.1225

MLDDTETTNGPVTCSFCGKSQDQVKKIVAGPGVYICNECIDLCKEIIDEEFKDEAVNDLLEVPKPREILAILNDYVIGQTEAKKALSVAVYNHYKRVNQMTVAEKGDTELQKSNIALIGPTGSGKTFLAQTLARILNVPFAIADATTLTEAGYVGEDVENILLKLLQNADFDVDRAERGIIYIDEIDKIAKKSENVSITRDVSGEGVQQALLKILEGTIASVPPQGGRKHPQQELIQIDTTNILFIIGGAFDGIETIVKNRLGEKTIGFGSKAGQAVDENQSLMQQIVTEDLMQFGIIPEFIGRIPITAALEKLTEDDLVRILTEPKNALVKQYSKLMALDDVELEFTPEALHAIAHLAIERNTGARGLRSIIEEVMMDMMFDVPSREDVVKVLVTKEAVDGTAKPQLFLADGQEAS

>fig|1664.9.peg.1226

MEVHEVEMVMSAVAASQYPTDQLPEIALSGRSNVGKSSLINKLINRKSYARTSSKPGKTQTLNFYRVENELYFVDVPGYGYAKVSKKQREKFGQIIEAYLTNRDNLRGLISLIDGRHEPTDDDIAMYVFAKYYDIPVLVVATKMDKISTGKWNRQEKIIKEALDFNPVDKFVCFSALTGAGKDTVWKWIEQQCDIGGRR

>fig|1664.9.peg.1227

MEFNEYQKLANRTLYGNEQVLTNLALGLASESGEVVDIVKKYTFQGHQLDEQVLTKKIGDVLWYLSQIAEWNNVDFEKVAQENIEKLKERYPERHAE

>fig|1664.9.peg.1229

MTIKKSLAFILLPLALLVALLVGNNQAVSAAGQTVSRVQAKKTLVLGTSPDYAPYEFLVNKNGKNKTVGMDIDVAQKVADDLHVKLVIKSMDFDALLVGLETGKVDMVISAMSPTDERTKSVDFSDVYYLGGQSILVNKADAKIYKSKNSFIGKKVGVQTGSLQQTLAKKQIKNAQITGLTKVPDLVLALKSHKIDGIVAEEPVATAYASNDPELVQIDGKFNLGADMQGSAIAFPKNSPTLVAAANKSIAQIKQQKLIPQYLKSAGSLMKTNTHNTSMWHYWSYFAKGIGYTLFITIISIIIGIFIGVVLAFARLSKSKLFHSLAVMYIEFVRGTPLMIQIMFVYFGIGLWVNIPALFAGVIAVSLNSAAYVAEIIRSGINAIDAGQTEASRSLGLSQHDTMRYIILPQALKNIWPALGNEFISLIKESSIVSIIGVTDLIYQLKVVQSATYKGVAPIFVAMVIYFILTFSLSKLLNHIERKMQHA

>fig|1664.9.peg.1231

MASEHIEHKLALLPALPGCYLMKDVNAQIIYVGKAKNLKNRVRSYFKSSHEGKTAKLVSEIVDFETIITSTNKEAFLLEITLIQKHQPYFNIKLKRGTGYPYIKITNERDPRLLITGDVKKDGGYYFGPYPDVYAAQETLRFLQRVYPLRRCQGHQGRPCLYYHMGQCLGACFQEVPEAAYTEQIKKIKRFLNGQVDNVKKDLTEKMATAAQEMQFERAAELRDQLRYIEATVEKQKIISNDHTPRDLFAFYMDKGWLSIQIFFIRQARLIRREKRLFPCVNTPEEELATFILQWYNRKNNVLPREILVPDGIEKQVLSDILQVPIRTPQRGEKKALMEMAQKNSRLVLEEKFRLLELDNRKTVGAQDEIMDALGLPHGHVIEAFDHSHIQGTDPVSAMVTFVDGRAEKKLYRKYKLTQTAERAGANEDANTREVIYRRYSRLLKEGKALPDLILMDGGVVELNAAKDVLENELGLTIPVAGMVKDNHHKTASLLFGEADQTIQLDPKSQGFYLLTRIQDEVHRFAISFHRQLRGKNSLSSKLDDIKGVGPKTRTKLLKNFGSMKHIKDASVEELEALGISKTVAQTIKLSL

>fig|1664.9.peg.1234

MQKKVILKNGLGIIIGAFIYAIAINDFLIPHQIGEGGVTGLTAIGYYALAIPPAFTNLVLNGLLVVIGFRYLERQTIWYSVWAVIWISLFLKLPHFIGYHTTQTLIPTVIGGVLMGIAMAIIIHCQGTIAGSTILAKIVNRYLGIPNGSAMLFFDLIVAIPSGLIIGFQNMLLTIIELYLSAVVLNKLLAKFGAKRAVTIISDQYETIAETLSTQFQQGITIIKAQGYYSQNERPMLYVICTAKQLATLVPVVSQIDVKALVVVEEARSVQAEQLKQLL

>fig|1664.9.peg.1236

MNIQDLLLKDAMIMDLQATTKEAAVDEMVARYKDIGVITDDTLYRKDILAREAQSSTGIGEGIAMPHAKDSAVQRATVLFAKSQNGVEYDALDGQPVYLFFMIAAPEGANDTHLQALAGLSSLLINPQLVADLKLAQTPEDVQELFAKAQAAKDAEDAKEEAEEAAKAQAAAATPADEQKPFIVAVTACPTGIAHTYMAEAALKETAAKMGIDIKVETNGSEGVKHQLTADDISRATGVIIAADKKVDMPRFNGKHLLNRPVIDGIKKPAELIQKTLDEEGSIFHATESQASEEASADKKTLWSRIYQDLMNGVSNMLPFVVGGGILMAISFLLEQTVGAHSTTFIFLNSLGNNAFNFLIPVLAAYIAISIGDRPALMPGFVGGYMASQATASVISSDSPAGFLGGLAAGFIAGWVIVGLKKVFKNLPQTLDGLKPILIFPVLGLLIVGFIMFFMVNPVFAQINLWLSTFLTHLGAGNALLLGAILGGMMSVDMGGPFNKAAYTFAIGVFTTTKDGSLMAAVMAGGMVPPLAIAVAATIFKNKFTGSERKQAFANYVLGISFITEGAIPFAATDPLHVIVSSVIGSAIGGGLTQLWQITVPAPHGGIFVAPLANHGLLFVLAVLIGTFISALILGLWRPVAKES

>fig|1664.9.peg.1238

MLTEERHQAILDLLQQQDVIKMKALCRLLDASESTIRRDLQLLEEQGLVTRIHGGAKRLNKLQVELGQIEKTSKNVHEKNEIAKFTASKIQLGCVIYLDAGTTTQAIIPYLTPEMNLTVVTNGVDTASLLADHHIQTYLLGGRVKNKTKAMVGAGALETLLQFQFNQAILGTNGVDQYSGLTTPDPEEATLKRFAIQQANQTMVLADHTKFDAVSFSKFAELTQVQLITDQLSPALRQTYQTHTDLQEVL

>fig|1664.9.peg.1239

MGKLGVSIYPERSTFEKDAAYLDLAHQYGFKRVFTSLLEVSGDQDAVLTNFKRVVDYANQLGMQVMIDINPGLFEQLGISYNDLSFFHELGAWGIRLDNGFTGQEEAKMTRNPYDLKIEINMSQGTSYVDNIMSFSPKRANLLGCHNFYPHRYSGLGEDFFNQCTAQFAQYNLNTAAFVNSHEATFGPWPTQDGLPTMEVDRDLAMATQMKHYMLMDNIDDIIVGNAYASEAELKTMQTVFNAEFPAIMIDVAEDITADERKVLFESLHSYRGDRSEYILRSTMTRVIYKDLPFAAHNTVDIKRGDILIDNVGYGQYKGETQIALQAMPNDGRVNVVGHIAPDELFLLKYLKPWSSFKLIPNAQ

>fig|1664.9.peg.1240

MFVDQVKIDVKAGKGGDGAVAFRREKFVPLGGPAGGDGGRGGSVILVVDEGLRTLMDFRYRHHFKANSGGNGQNKQMYGRGAEDTFVQVPPGTTVRDADTNELLGDLTEDGQELVIAKGGRGGRGNMHFATAKNSAPEIAENGEPGQERSIQLELKVLADVGLVGFPSVGKSTLLSVVTSAKPKIASYQFTTLVPNLGMVQLDDGRDFVLADLPGLIEGASDGVGLGIQFLRHVERTRVVLHLIEMDDQTGRDPYEDYQQINHELESYDPKILERPQVIVATKMDLPGSAELLAEFKQKLAAAGDTHEIFEISSITHQGVQPLMNKTADLLAETAEFPMGDVEEAQTQKLYQYQPEGPAFNVEQDEDGTFYITGEKVERLFKMSNLDHQDGVMRFARQLRSMGLDEALREKGAHDGDLVAIDNFTFEFVE

>fig|1664.9.peg.1241

MAGKKRLNNSRYITGFDGLRAIGVIGVILYHLMPYTFTGGYLGVPIFMVVSGYLITDLLVQEWEQNHWINLKSFYLRRVKRLYPGLLTMLFATGAYITLFERQMLHNLNQIIITNILYVYNWWQIGHGQSYFDRFANNESPFTHLWTLSIEGQFYLLWPFVVVALIIGFKNKGRIARVLLGAGLVSAIWMAILYHPNADPSRLYYGTDTRMFSILVGAALAFVWPSTALKQKIIPRQRWTLDVIGLLSTITMLILMVKMNAESAFVYRGGLFIFSLLICVLVAVVAHPGSDWNRLLTNPVFNWLGKRSYGIYLYQFPVMIFFEGVFKNVAAHPALYPVIEVAIIFGLTELSYRFIEQPLAHYPYQELGQRMRQFVKHPTLNRQSGLVFGMAIVFLVGLVGVIQAPGQPAPDAHKSALAVNIQKNKKENDARNKALVEKAKAQNKQQKKAKSKAQADKEAEAAAQKHPVNQEYEKYGLTQVELQKAQSMPITAIGDSVLLDAGKTLQTLFPNMLVDASVGRQLNKSTPVVDSYAQKGALSDTVLISLGTNGSFTQAQLDEFMAAIGSQRKVFWLNAYVPTRPWQNDVNQMLKGAAKKYPNLTIINWYDQARKHGDWFYDDQVHPNPVGVKEYAAIVTKALVKQ

>fig|1664.9.peg.1242

MELEFLGTGAGVPARQRNVTSIALKLLDERNEVWLFDVGEATQHQILKTTLKPRKVKKIFLTHLHGDHLFGLPGFLSSRSFQGGDEPLTIYGPKGTEEYVRTSLKLSESHLTYALKFVVLPENGVIIDDKTFRVECAKLDHRIASYGFRIVEKDHPGELQVEKLQADGVPSGPVYARIKNGETVTLSDGREIDGRNYIGNAQKGRIVTIIGDTRNCEAITRLAENADVLVHESTFGKQEQKIARQYYHSTNINAAKVAKTAHVKRLLLTHISARYLGQAVRELQNDARDIFKNTRVVSDLDLYDIPFHGRKE

>fig|1664.9.peg.1243

MQNLAALQDLKQKIVVVTGGSSGLGRAIAYEAASKGAIVVVLARRQAALEEVRDECQRLSGQAAYAYQLDVSAPEAIEAVVAQLTTEVGIPDVLVNAAGFGHFEEALATPMPLVEQMFRVNVLGLMYLTRAIGSQMIEKQRGHIINIASMAGKMATPKSAIYSATKFAIVGYSNGLRLELKPFGVQVTTVNPGPIDTAFFDIADQTGNYLKSVNWIVLDPDQLAHKIVGAIGHPKREINAPWLMSLGAQFYQLAPHIGDYVAGNLFNKK

>fig|1664.9.peg.1244

MMKKQGRLITILILALIVVVFAVLNVNPVAINFGFTTVDWPLIIVIVVSLLIGAIITFLAATGSSLSQRKAQKQWTKEKADLEQGQEAKIKAAVEAEQVKQKATYDKLLADKEAELHEMNAKMKQLMQSKEV

>fig|1664.9.peg.1245

MNPEQMTQAVQDAIAEAQNIAKTRHQTEIDVPHLFKYLIQPQQLGRQIYQEAGIDLTALESEIDREIDTIPTVEGQTNYGQNISQNLYQVLNAAQTYMQEFQDEFLSTETVILGLMKSKYNPLVQFLQKNNVTTKQLKDIITKLRGGERVTSKNQEDNYQALEKYGTDLVKAVREGKMDPVIGRDEEIRNVIRILSRKTKNNPVLIGEPGVGKTAIVEGLAQRIVRKDVPDNLKDKTIVSLDMGSLIAGAKYRGEFEERLKAVLKEVKKSEGRILVFIDEIHTIVGAGKTEGSMDAGNLLKPMLARGELHMIGATTLDEYRQNIEKDKALERRFQKVLIQEPTVEDTISILRGLKERFEIFHGVRIHDNALVAAATLSNRYITDRFLPDKAIDLVDEASANINVEMNSQPTELDQATRQLMQLEIEEAALKNETDAASQKRLAETQKQLADLRETTNSLKMRWETEKADLNKINDKKEELDQAKRQLEDAQSNYDLETAAKLQHATIPQLEQDLADLEKAEKPEDWMVQESVTENEIATVVSSQTGIPVAKLVAGDRQKLLQLADHLHERVIGQDQAVDAVTDAVLRARAGLQDPNRPLGSFLFLGPTGVGKTELAKALAEDLFDSERHMVRIDMSEYMEKYSVSRLVGAAPGYVGYEEGGQLTEAVRRNPYTIVLLDEIEKAHPDVFNILLQVLDDGRLTDSQGRTIDFKNTIIIMTSNLGSEILLDGVDDQGQISKEAANSVQALIQTKFKPEFLNRIDDTIMFTPLSLQDVEQIVVKMLADLSHRLADQEITLTISEDAKLWVAKEGYDPAFGARPLRRYITNRIETPLAKEIIAGNVLPNTTVEIGLKDDQLVFENKATD

>fig|1664.9.peg.1246

MAVPARRTSKTKKRMRRGHIKLNTPNVQFDATNGEYRVSHRVSPKGFYKGEQVVTPKAND

>fig|1664.9.peg.1247

MNNELLKRDLNQLQLLQLPRWTDLPGLNLYMDQVVSYINQYLEVLDLDEITAAMINNYVKKGLVVAPDKKRYAKQQISQVLIIALLKTNFSIEEIGRLLSVETATDRNEKQLYDYFILTFLDAIHQLDEEYSPFEQGTKPANGLVTETQKTLLKLACQSVVYKIAIRITVKKDQTTTLKSADKK

>fig|1664.9.peg.1248

MAEKKKTSKLTLGALILMIFTSVFGFTNIPRAFFLMGYASIFWYVLAAVTFFIPYAFMLGEMGAAFGEAKGGIYSWMEKSVNAKFAFIGTFMWFASYIIWMVNVGNGIWIPLSNAIFGVDKTQSWTFFGLTNGVQTLGILAIIWIIVITFVTSKGLDKVKKVTSIGGSAITLINVILFLGGLLILILKKGVTAQPITAKAFFQSPNESYTSMIQVFSFVVFAIFAYGGLEVVGGLVDETENAKVNFPKGLAISAIVIAIGYAIGIFMMGTFTNWDFVFKTGGGFAKATGSNAEVTLGNVAYIAMNNMGYQLGLALHMSHHAAIQVGIWLSRYMGLSMFLALTGAFFTLIFSPLKTIIEGTPAELWPKSFAKKNKHNMPVNAMIVQAAIVIVIIAFVAFGGKSAAKFFEILTAMTNVSMTLPYLFVAFAFFGFKKDNSIEKPFTIYKGKTFYKFCVWMVMFTVGFANVFTIIQPAIDGNLGQTFWSIAGPVFFTVVALIMFNRYEKKTGLK

>fig|1664.9.peg.1249

MKKTVVGLVVSLGLVSLLAGCGQAKLTTSKTNYHQNGMVAVVKGTTNKKAHLSYQIDQGAMHKTSVREGTYVVQVPTTTKQQTVTLKAKTGSKAIEKKATVAAGTAVGQYQAIADKYNQAVIGMALSKADQAKAQSLQKQAAAAKAGQLAPAQLMALQQTQKDVQAAMQQAKTAKKAQLLPAKSADGVHQLVKTDNVVIRGAIADQDVMGLTLIAPIKALKDKAALKEFGTSFALLAQSTGADSKKVMSEFSKFTKKNKKSNQTTMKTITSNGVKFNTGFSASHLYIYITK

>fig|1664.9.peg.1250

MKYVMTLFWTLIFGFVVGFIGSKLVKLDFNVQATLTVSVIFGILLNLVPLVLPKDPAA

>fig|1664.9.peg.1251

MAFVQLQVKSTYTLLESTTKITDLVTAAKARGYQSLALTDKNVVYGLVDFYKAAKAADIKPLLGITIEVGGLIQTDERFPLILLAKNLTGYQNILKISTKIMTQSELVPFEAVQSLLQQLVVITPSQDGELVRLLLQNDATAAQTYIERLKAVVDADSLYLGISAKQAASRQRVPLGQLSQQTAVPLVALGDVRYLEPEDAFAVQVLQHIKAGTQVNLQTPQAPGGYFLEDVQQATAAFEALQLAEAVANTQKIAAQCQVDLSFKRAQLPQFETPAGQSAATYLKQQSQAGLQARFKTQPIPETYQTRLDYELQVINEMGFADYFLIVADVMQYAHQQNIMTGPGRGSAAGALVAYALRITDVDPIRYQLLFERFLNPNRANMPDIDLDIPDNRRDEVLDYVYQKYGQNHMAQIITFGTLAAKMALKDVGRVFGLSQFEMSAWSKAIPNVLKITLQEAYDQSQALKNLVADSQQNRLLFETAQRIEGLPRHYSTHAAGIVLSQAPLTETVALQSGGDEIELTQLPMGNVEELGLLKIDFLGLRNLGILANIVKLVSQQTGQPFNPQNIPLDDPATLALFQQGDTNGIFQFESTGIKNVLRRLKPTSFEDIVATDALYRPGPMENIDTFIDRKSGKVPVTYPDDSLAEILQPTYGILVYQEQVMQAASKMGGFTLGEADILRRAMSKKKKAVIDASRTKFIEGALALGHTEQAATTVYDYIEQFANYGFNRSHAVAYSKIAFWLAYLKVHQPEAFFAAVLNAVLNQGTKTKTYLAEAKKRQLSVLPPDINLSQRYFKVTDAGIIFGLYSIKGLRRDFVAAILDERQTGGPFKSVLQFLQRLDAKYLKKEALVALIYSGAFDQFNPNRNALLMNLDDLLDSVKLAGDNMSLFEILAPKEKTVSNLTLTERLDKEAEYLGAYLSGHPVEKYERVRRYYHVPEVSDLAPNQNVRLVLFVRRIKVIRTKKGEQMAFVTGQDATGEISVTLFASVYRQVADWLTKEQVILVIGKTEQRQELQLIGNQIMLAEQAQKGLPKATLYLRLSADLTREQQQKMYQLLEKSRGPIPVILYNSATKKSILLNERYWIANDEKLTTALTTLLGPTNVILKGFS

>fig|1664.9.peg.1252

MTLKDQIGFLSKIFAWGEKMKRIGILTSGGDAPGMNAAVRAVARKAMHEGLEVYGINYGYAGLVAGDIFKMDPITVGDKIQRGGTFLYSARYPEFAQVEGQLKGIEQLNKFGIEALVVIGGDGSYHGALRLTEHGYNAIGLPGTIDNDIPFTDQTIGFDTAVNTVLESIDRIRDTAASHERTFVVEVMGRGAGDIALWAGVAGGAQDIIIPERDFDVKAVAEKLRASREHGQKHAIIVLAEGVMHADEFSEKLSEYGDFHLRSTVLGHVVRGGSPTASDRVLASRLGSKAVELLLEGKGGVALGIEKNEIIAHDMLDLFNHKHHAMLDLYDLNEDLAY

>fig|1664.9.peg.1253

MKKTKIVSTLGPASNSVEIITKLIEAGANVFRFNFSHGDHEEHLSRMNMVREAEKATGKTVGIMLDTKGAEIRTTVQQGKKFELHTGEVVRISMDDTLEGTPEKIAVTYPGLYDDTHVGGHVLIDDGLVDLKIIEKDEANKELVTEVQNDGMVGSRKGVNAPGVSINLPGITEKDASDIRFGLDNDINFIAASFVRKPQDVLDIRELLEEKHMEHVQIFPKIESQEGIDNIDDIMKVSDGLMIARGDMGVEIPFENVPFVQKNLIKKCNAIGKPVITATQMLDSMQENPRPTRAEVNDVANAVIDGTDATMLSGESANGDYPVEAVATMARIDERTEKSLTDRDAYALKAYSKTNLTESIGQAVAHTARNLGIKTIVAATESGSTARMISKYRPQADILAVTFDERTRRGLTVNYGVYPVMAKKPANTDDMFNLATQTAQEHGFAKEGDLILITAGVPVGERGTTNVMKIQLIGSKLTQGQGVGDETIVGKAVVASNAKEAAEKAVDGGILVVKATDKDYLPAIEKSSAIVVENGGLTSHAAVVGIAMGIPVVVGAKDATEAIKDGEVVTVDSRRGIVYRGATNAL

>fig|1664.9.peg.1254

MESWLFLAAILIVALLAKNQSLIIATAVVLVLKALPISEKVLPVIQAKGINWGVTVISVAILVPIATGQIGFKELISAFKTPAGFIAVGCGVLVAVLSAKGVGLLGASPEMTVALVFGTIMGVVFLKGIAAGPVIAAGITYTILTIFNLVPIH

>fig|1664.9.peg.1255

MNELIGQVITALVTDENEEAYFAQKDGVTFELKKDHLEEGLAIGATVTGFAYENSSRELVLSTVIPKSRVGHFAFGEVVETRRDLGVFVNIGLPDKDIVVSLDVLPTIMKLWPKVGDRLMIAIEVDQKGRMWGQLADENIFRAISKGAKQEMKNQDIEGTVYRLKMAGTFLLTDDFYIGFIHPSEREAEPRLGQRVKGRVIGVRPEGSLNVSLKPRAYEEIGDDAEMIMAVLKRQPGYAMPYTDRSNPELIKSYFGISKGSFKRALGRLMKNGFIVQEEGETILTAKGQKDIEPIVDNDQDQD

>fig|1664.9.peg.1256

MSKAEQLEQIRKQLQEAGYKLTAQREATVAVILECSQAHLSAEQIFIKTKAEVPEIGLATVYRTLEMLTELHVLNKISFVEDGVTHYDLCEENASQHFHHHLLCLQCGAIEEIHEDLLVSVEQTVAERFSFAVLDHRLTLQGICANCQAANSANGLPANQLDPSKQVDISQL

>fig|1664.9.peg.1257

MDDLIQDYLHYLKIERGLSENTRQSYRQDLKQYQQFLVSQKLTSFTEDRFIVLGFLQAQTTAQKAQSSITRSISTLRKFYQYLAREGRIQKDPMLQIDSPKQGRHLPAVLSSEEIERLLKTPDTSKPLGLRDRAILEVLYATGLRVSELVHLKLTDLHLSLGLIQTLGKGDKERIIPIGDVAVDWINEYLERSRNRLTKGKDSPYLFVNFHGNGLTRQGIWKNLKAIVQAAGIDKGVTPHTLRHSFATVLLENGADLRIVQELLGHSDISTTQIYTHISKKHLTEVYQRSHPRD

>fig|1664.9.peg.1258

MLFKYKNDYEKIAMGFLSFIPDLKEVSHVQSELAFYNQDDQHVLYLWQNDEGDFAGVVGVEIGTDFILVRHMSLSPAERTDTNLFRILNELAALYPDNRVMGSLDTAPLIAKWEQQQTKEMD

>fig|1664.9.peg.1259

MTEQLTLILTDFEGPLDLLLHLINQTKIDIYDIPIAEITTQYLDYLHQMQELKLDIVGDYLVMAATLMKIKSKLLLPAPPAMAETLEEDYVDPRQELVDQLLAYQAFQKIATVLQERESERQLLFAKPASANPAEKVVPLARGVIKPRDLAAAVSKMLVEATVQQTDFKTVQNDEITIAEKMDWIIDCLKTRATTTFEQLVINRFDREEIVTTFLAVLELMKERLINCDQAGYDEQILVTLKEGIADEPSR

>fig|1664.9.peg.1260

MNHLAEIESLLYVAGDEGITLQHIARLIMLDEAAVRQLLTKLAKRYQEDEQSGLNLIQAADCYKLVTKKAYAGLLKAYFDGPVSTSISQAALEVLAIIVYRQPLTRIEIDEVRGVQSSGAIQTLLARQLIVEKGRKDAPGRPILYGTSDYFLDYFGLGSLKELPELEQMTLTDDTAESDNDSADLYYRQFEQTLNETGPETAPKGEQ

>fig|1664.9.peg.1261

MERLQKVIANAGVASRRKAETLIATGHVTLNGKVVKEMGIKVDSTSAHIEVDGVPLTREKKVYYALYKPRQVISAVTDDKKRRVVTEYLEDIQERVYPIGRLDYDTSGLLLLTNDGDFANMMMHPKYKVDKTYVAKVSGLPTAEMLRQLRKGIMLEKRKTAPAKVKIISTDRRKNTAILSITIHEGMNHQVKNMLAAVGLPVSKLKRETYGFLTLDGLVSGEYRALKPIEVAEFKKIAELNEKPKHKNRPKLSRHD

>fig|1664.9.peg.1262

MRVSKVRTMVGVAMLGAISFIVMFFEFPIILAFPFLKIDFSDVIVLLGTFIYGPIGGIGVAVIRSLLHFIMTGASLPSLVGDFAGVMASIFYLLPFYYLFNRQKSVLWGQVIAGTVSSISMTIVMCLANWLFILPMYIKLLNFNLGMSMTKYVFIGLVPFNLIKGALVTVVFAVLYLRILPWLKQHMKTAI

>fig|1664.9.peg.1263

MLYSKVIPERCIACGLCQLKAPELFDYDHEGVAFFKPDNNAGQTPIENPHDLIAFKAAYTACPTRAIVRQEEPFSS

>fig|1664.9.peg.1264

MLTDYLLCLFATAPRRYQAIYYTLVGKRTTSNLYAGLAYGLLKWLQIYPNLELAQFQAELTQLQEKGWLMVEDRQAWLTPTGAAQKATLMTTLEWPTHYYGPEMGHLQQFQSRLFLAIQIMSEYSHHNTHYFPIETSMRDRVQVVSWFKQHKQDSEMVTQFKQELQQILTQLTPVQADLLANRLVGHQIIGQSQQQLAQKLTLEPLTVTIRLLDAQAHFFAILSQHTTDYPLLACLLSPLDSTLSLSTAQTWTLLQQGQSIEMISQKRRVKLGTIREHILETAILQPQFPFERFISSDLETALAALMTEMPDLTFNVAHEQLPTLDFFSYRLYQIKTKVVAA

>fig|1664.9.peg.1265

MTSVQLIYDQLAQHFGYSTFKPGQLAVIKSVLAGQETLAVLPTGTGKSLCYQLPAYILGHTTVIVSPLIALMQDQVAQLNYQGEKRAVAINSNLVPQQKYQLLTHLADYRFIFMAPETISQREVIQALQRCQIDLFVVDEAHCISQWGIDFRPDYLVLAAIKAQLQPTATLALTATANDRVRQDILNKLALKNAAQIMTSVDRPNIYLGVSQQPDEQAKNQFLANLVKKSQGPGIIYFSSRQKTMAIATFLQQETDLKIAYYHAALSTQERYLIQQQFMQGQLDLICTTNAFGMGINKADIRFVIHYHLPQNIESYLQEIGRAGRDGQAAYALVLTTPQDLYLQQSLVSFGIPEPGLIRRYYQEPQLFKQSQGSEFELLRRYQQLGMTEEEVTALLDNRQTEKQFSLQTMLDYLTLKGCRRQFIVNYFDQAPKTIEHSETCCGEQDSTDFLAQLDLEQAINYKTAETVVLTGWTGILDQLF

>fig|1664.9.peg.1266

MLKDDKKKQDNPTEEKPWESQFDDDRDDKGNLSRVAAKSKEKGNTYFAIILAVLLVLMIAFPIIAYTVHSSRANRPGLEDSKIVVKTADSEKSKSKSTAKKAKEASESQEAAESKAASESKAKADSEAASAKSASESAKAQEASEQAASQQQAAADSASQQAASESAASSSQAEASSSSSSSETAGGTYTLKQGEGLYRAAANNGMSLSELLALNPGLTANSSVAPGTALKVK

>fig|1664.9.peg.1267

MQIAIDGPASSGKSTVAKIVATKLGFVYVDTGAMYRSATVLAQQLGLDYGDEAGLMAELTKADIQFQPDEAGQKVFLNGLDITLAIRTPEITNNVSQVSALPAVRTEMVDRQRQIAEQGQVVMDGRDIGTTVLPDAKVKIFMVASAKERAKRRYAENTAKGIMTPLATLQAEIELRDQKDSTRAVSPLRKADDAIEIDTTKLLIDEVVAEILTIAKQNM

>fig|1664.9.peg.1268

MNDANLNDTQSSETMAAALDSVHEVKIGDVVKGEVLVIEDRQVIVGIEGTGVEGVVPQKELSSQPIETLTDAVKVGDQFDLVVIATIGNDKENGSYLLSKRRLEARKVWAEIEQKFENKETIKATVLNVVKGGLVVDAGVRGFVPASMLSDRFVRDLNSFKGQELELQIIEIEPSENRLILSRKALVEQERQAQLKAVFEKLLPGDIVEGEVARLTNFGAFVDLGGVDGLVHVSEISYDHINQPSDVLEVGQNVKVKILSLDQEKGRISLSIKDTQPGPWDDIAEKAPVGSTLEGTVKRLTTFGAFVEVFPGVEGLVHISQISHQHIATPNDVLEEGQTVQVKVLSVDPEQQRLGLSIKALQEKPAGAKDSEQEDVAPSDYEMPEEETGFSIGDMLGKALDEK

>fig|1664.9.peg.1269

MSKPVIAIVGRPNVGKSTIFNRIAGERISIVEDTPGVTRDRIYTASEWLGHEFSLIDTGGIEISDAPFMEQIKQQAEIAIDEADVIIFLVSAREGVTDADERVAQILYRADKPILLGVNKADNPEQRQDIFDFYSLGFGDPIPVSGAHGQGVGDLLDAAVAKFPTDLEEEDDDSIKFSLIGRPNVGKSSLVNAMLKEDRVIVSQIEGTTRDAIDTKFMAENNQEFTMIDTAGIRKRGKVYENTEKYAVMRALRAIDRSDVVLVVLNAEEGIREQDKKVAGYAHEAGRGIIIVVNKWDTLEKDNHTMKEFEDHIRNQFQYLDYAPIIFVSAKTGVRLQNLPAMIELVSENQNRRIQSALLNDVLMEATTVTPTPAINGKRLRIYYMTQVAVQPPTFVVFVNDINLLHFSYQRFLKNQLRKTFDFTGTPIHLIPRQRK

>fig|1664.9.peg.1270

MANKAQLIENVASKTGLTKKDATAAVDAVFGSIQDTLKQGDKVQLIGFGTFEVRERAARKGRNPQTGAEIKIPASKVPAFKPGKALKDSVK

>fig|1664.9.peg.1271

MTNSEQMLSALEDNDLKQADELFQKALKEDDPETLYSLAEELYAMGFLEQASTIYQQLLAQFPDEDQIRTTLADIAVSNGQYDDALNLLTDITPESNAYAESLLTAADVYQTMGLPEVSEQKLLTALRLYPDEPVMQFALAELYFEMGEYGKAANYYEALIDQDVLELGQVNIQKRLATSYASSGAFEEALQIYEIITVEFLTEDDQFQMGFLYLQIKDYQKAIEILTKLQLADPQYTSLYVYLATAQEAQNELQEALTTVQTGIGYDMYSEVLYQKGSDLALQLGDDQVAEQMLNKALEINPDNMGEISALSQVYRRQNRHAENVAFVQEAIANHKPTAELYWSLAQSQFALEAFDDARENYLLAYPELKDNPEFLHDLILFFQEMAQVPELLAALKQYLQLVPNDLDMQTLYDDYQTMQ

>fig|1664.9.peg.1275

MLMENQNKGLRSAYALAFTCMVAFMGIGLVDPILKTIAVKLNATPAQTTLLFTSYMLVTGVIMLFTGFISTRIGAKRTISIGLIIIVLFAALAGRSQTISQLISLRAGWGFGNALFLSTALTAIVRVMPEKTEQAIMLYEGSMGIGMAVGPLVGGRWGVFLGVFRFMA

>fig|1664.9.peg.1277

MMKKQLLKWTIVGFCSVLGVTALGAGQVSAHGYITSPGSRAYLGSNAFTNDTGQKPLNTNVGQVQYEPQSIEAPKNTFIDGKLASANISSFLNLDEQTSTRWHQNQVKSGALTIKWHLTAQHKTSTWDYYLTRPGWNPNQPLSILNFEKITSIDDQGKLPAKDVAQTINIPTDRSGYNVLLGVWNISDTANAFYQAVDLNITK

>fig|1664.9.peg.1279

MMNMNKAVGFYKHLPISNPDSFEDIMCQLPTIGDHDLLVKVEGVSVNPVDTFVRCSGRSTKMAHPKVIGWDAVGTVVKVGSECTLFKPNDQVWYAGDFKRSGSDSQYQSVDERIVGPTPQNLSLQEAAAIPLVGLTAYESLFEKLALSWDILKNKGKTILIINGAGGVGSMAIQLAKLAGLRVIATASNPDAIAWVNQLKADAVIDHHQDLVKQMHHLKIRDVDYILNLNNLDAHWNEIAEIIKSDGRVTATTENHKLIDLQKLTKKRVTFSWEWMYSKSYYQTADMATQNQILTELCQLFEMEKLMPITTKTYTPINAEHLKRAHRDVESGHMIGKVTLTGW

>fig|1664.9.peg.1280

MTQKTNLTTTLRDLIAIAIGCAIYAFGLITINIQNYLAEGGLTGITLIVRYWLHIDPAYTTLILNIPLVILGYKFLGKRALAYTIYGTVMMSVFLWIWQRVPVTINLNHDIFIAALLAGLCGGFGSGIIYRYGGTTGGSDVIARIIEKRRGIQMGRSLLIFDIFVLTASLSYLDIEHMMYTLIASYVFSRVVNFTLEGTYAAKGLLVVSDHYQKIADQIMTQTGRGVTFLHAEGGYSHQSRQMIYCVVSASEVAHLKRIIEHQDPKAFISIIDVHEALGEGFTYETPTKD

>fig|1664.9.peg.1281

MKLTTFAPEFAEALPILEQIEAAGFEAYFVGGSVRDNLLGLPIHDVDIATSAYPAEIKQIFKRTVDTGIQHGTVMILDHGNGYEVTTFRTETGYQDFRRPDSVTFVRSLEEDLKRRDFTINALAMRADGEIIDLFDGIADLKAHKIRAVGVADERFHEDALRMMRAVRFESQLGFSVAETTQAAIEKHAALLEKIAIERIHVEFMKLMQGIERQNGLRTFIDTGLYRYCPDLADQLLALERLTALPTEQLHDESAVWLVVTYLLGQTPAQAGRFLKHWKSANDVIDAVKAGLVLLPKLLTATADQWDLYQAGQAVLVISLQIAQLVTTATIPTADWLERYDRLQIKQKADLAINGQILMQNGFQPGPILGKTLAILERKVVLDELLNTTEALLKAAK

>fig|1664.9.peg.1282

MAQHVFKELKLYFRNGDAWTINHKELNDIWISRVTTSYGRINGGPMQEIHPCKSFKAEILPEADHVKSEDINTGSLEMGMFGRATKYQDIEKLDLIFEDEQDKKPLQIYFPFKNKDTSGLDNIYQTSQISQKNGHLYIVIDATSSIADEYPKI

>fig|1664.9.peg.1283

MQTLTATNLTKTYGEKTLFKDISFLIREGDRIGLVGINGTGKTTLLNALTGTDSYDSGQIETPNQYAMSYLAQKPVFDTNLSIMAAIFAGQNPVFKTIQNYERVLAAYTADPTNPKLQTQYEQADAQMTQQDAWTVDTDIKTILTQLHLTDLDQPINTLSGGQQKRVGLAQALIESPDLLILDEPTNHLDFDSIEWLEKRLAQYKGALLIVTHDRYFLDRVANQIFELDAGQLYTYTGNYEQFLTQKADRIEREQQAAHKQQQLYKQELTWMRAGAKARTTKQQARINRFNDLKGNLTTGPDTSEVDIQMGQSRLGKKVLELKDANLTLDAHPILKDFSLLVQANDRIGITGVNGAGKSSLLNVLAERIPLDSGELVVGETVNLGYYTQQNEDLDLDKRVIAYLQEAGEEVVGKNGDRISVTQLLEQFLFDRSTHGTLIEKLSGGEKRRLYLLKLLMQQPNVLLLDEPTNDLDIGTLTILENYLENFNGTVITVSHDRYFLDQVADKLLVLDGHGNITTEVGLFSDYLAKQKTATTKVTAPKPTPEVTVEAVKEPAAKHKLTYNEQKEYAHIETDLEKLDEQIETIKTEMNQNGADYGKLAEWQAELDRLNQELDEKMARWDYLSEYAD

>fig|1664.9.peg.1284

MSEQVYLDLARTILEEGHYKGDRTNTGTYSLFGYQMRFNLQEGFPLLTTKKVPFGLIKSELLWFLKGDSNIRYLLQHNNHIWDEWAFERFVKSTDYTGPDMTDFSHRAQDDADFKVVYQEQMRLFNDRILADEGFAKQYGELGDIYGKQWRAWQTRSGETIDQIKNVIEMIKTNPDSRRLIVSAWNPEDVPSMALPPCHTMFQFYVNDGKLSCQLYQRSGDVFLGVPFNIASYALLTHLIAHETGLEVGEFIHTLGDAHIYSNHVTQVKTQLARSMHAAPKLWLNPDKKSIFDFDVADIKVENYESEPAIKAPVAV

>fig|1664.9.peg.1285

MIAFLWAEDQNGLIGQDGHLPWRLPNDLANFKRETINEAVIMGRKTYDSLPKKPLPGRQNIVITRQSGLTVAKDVLVFNERTQLLDYQKEHPMQKLFIIGGADIFKLFAANVDYLYVTKIADKFQGDVYMPTIDYTQFKLISQQAGTVDERNQYPHTFEIYQRITK

>fig|1664.9.peg.1286

MAEVVVANIKIVTDSSIQLTPEEIKQHHITVIPLTIMIDNTVYIDGETITRDQFMTEMASASALPKTSQPAIGNFIETYEQLAADGSQILSIHMLRAISGTVDTARQAGEMAKADVTVIDSDFTDRAMAFQVLKAAEVIEAGGSLEDALAAIQAVHDNTKLYMGVTDLTNLVKGGRLSHAAGVISSLLNIKVILEVADSELKVLRKGRGMKTITKFIDEMDDDLRKLKNVKAIGISHADGLELSEKIKAQLQAAFPAIEILVRTTDPVIATHAGAGAFAVMYYTEN

>fig|1664.9.peg.1287

MKKIRQLLIDLGIFAGVLLVVFIVWQLLTPRPMKVRSHQESVQKTKVVQPKKVKKTLHLVAVGDSLTHGVGDEQNKEGYVSRIAAKIKTETGHPVATENYGVTGDTSVQIEKRVRTQPTLQANLKKADIITLTVGGNDLMAVLQNNFLDLDQKQITAGQKAYQTHLMTLFRQIRQQNPTAPIFVMGVYNPFYVYFPEITGMSKAVTAWNKTVKEVANDFESAYYINSDRLLTHGDGHYVKQTKSLAKMDSSKLQKTLAENEHLNPYISDDDHFHPNQKGYQLITNAFWQQMNAHQKDWTK

>fig|1664.9.peg.1288

MQTRQDLKKEQVSPKRTINYWKYSFIVLLALVIGTLGFIAYNVSAPQTKQVQTEKMIKADSTFDIQMHKKQINSVVAFYLENYLENSKVKYNFTLDQQAILSGQFKFLGFPVEFNLFFKPYVLENGDIQLRAKQLAVGQLKVPMSFVFNYIQRQYKSPKWVVLNAHKNRITLRLNEFKLANGMQVKARHIDLKNDKIDLSVYVPLKNKK

>fig|1664.9.peg.1289

MKQSFYRYLMTQRDPNNYEPVAQFANNAFFDQSFPKHEGDYEPLSQYLELNGSYLPSMLIFDEAYQLYQESEQA

>fig|1664.9.peg.1290

MRRKLAIAGNWFLAILVAGLVAFVIRSFFLVPATVAGNSMQPTLKSGDQLLLKKFGQVHRFEIVVFRLANGTTYVKRVIGLPGEHIAYQEGQLYVNDRPVVEPFLKQSQQKTVLTSDFDLKTLTDHDRIPANQYFVLGDNRRISKDSRTFGTIERETIIGRAVGVYWPFEDITYFK

>fig|1664.9.peg.1291

MTIQWYPGHMAKAQKQIKERLKAVDLILEIVDARVPESSINPMIQELGQNKPILLIMNKADMADPRRTQQWIETFKKRGITAIALDAQHKAKLPMIEKVAQEILAEKLAKKRAKGIRNPVIRAMCVGVPNVGKSTILNRLVQKNIAVTGNRPGVTKNQQWLKAGQTLELLDTPGVLWPKFEDPTIGSKLALTGAISDAIYHPDDVVIFALEHFKEYYPSQLQKVYKVTDAELEEPAAELIMILTKRLGFKEDYDRFCVKFINDARQGKLGRFTLDVVPVEAS

>fig|1664.9.peg.1292

MTKAQSIAEIKQLLQAPVTEEQLATFAADSRAGVQKLVVQYHKRAAKLAAQKAAFEERRQMEKALWPDYPLVAGIDEVGRGPLAGPVVTAAVILPHDFDLWQVNDSKQLSFKLKQELYREIMAQAVSVSIGIASPERIDTENIYHATELAMGEAVAGLDKQPDYLLVDAMTVPTDIPQEKLIKGDAHSISIASASIVAKVIRDQLMINYDQQYPGYDLANNMGYGTAKHLAGLAELGVTPIHRRSFSPVQNVL

>fig|1664.9.peg.1293

MLTKNQLLLKVHLAAGFGPISELRLAAWLATSHNWALSALSALEIAQITRLPERYWPTFQASFQSTTLQRQCIDHEVRTTYLTILDKDYPQRLLETYLPPVLLFYRGDLRLLKQPCLAVVGARQATHYSKQSLEQLLQGLTATTIISGLAQGADAMAHEVALQRGLAPIGVIGTGINCSYPPQNEHLQQVVAEKGLLLSEYALGTPARRYQFPARNKIIAGLCHSLIVTEARHKSGSLITANLALQANRNVYAIPGRIDQSLSQGCNQLIAAGATPLLDKNILIEELRYFD

>fig|1664.9.peg.1295

MKYSHKLSDAIHILAYVEIYQDGDLSSQSIAGSIESNASLVRRLMSLLVKADLLESRPGTIAPKLARPAEQISVLDVYQALDTDQQLLHIDEKTNPQCLVGANIQSALNDAYDQVQKAAEDQMAQIKLAGIIADILANSAQVNA

>fig|1664.9.peg.1296

MAGKNLVIVESPAKAKTIEKYLGRNYKVVASKGHIRDLPKSQMGVDFENNYEPKYISIRGKGETIKELKKHAKKADHIYLAADPDREGEAIAWHVSHILKLDPTDKNRVVFNEITKDTVKSAFKNPRSIDMKLVDAQQARRVLDRIVGYSISPLLWQKVKKGLSAGRVQSVALKLVIDREQEIKAFKPEEYWSLDAEFKKGRSKFKASFYGLNGKKTALGNNDAVQDVLKQIDKTQPFTIEDVKKRERKRFAAAPFTTSSLQQEANRKLNFRTRKTMMQAQQLYEGINLGGKEGTVGLITYMRTDSTRISTGAKHEASQFIHDNYGEEYAAVKAHKTKNPEGAQDAHEAIRPTSVMRTPKSLKDVLTNDQYKIYNLIWSRFVASQMTPAVFDTVAVKINQNNVLFKANGSQMRFPGFTKLYVSSRDTEDSAKDNLLPELAVGDEVKLAKTDPAQHFTQPPARYSEANLVKALEENGVGRPSTYSPTIETIQRRYYVKLNAKRFEPTELGEIVNNLIVEFFPDIVSIDFTADLEHRLDEVETGQENWIKIIDNFYQPFEKEVEKASVQIEKIQIKDEPAGFDCDICGAPMVVKMGRYGKFFACSRFPDCRNTKAIVKEIGVTCPKCGKGQVIERKSKKNRLFYGCDRYPDCDFVTWDKPVGRNCPKCEHYLVEKKIKGGKQVICPNGDYEEAVQK

>fig|1664.9.peg.1297

MTTKPFVNVIGAGLAGSEAAWQIAKRGVDVHLYEMRPVKMTPAHHTEHFAELVCTNSLRANQVTNAAGLIKEEMRRLDSIIIAAADENAVPAGGALAVDRTPFSQTVTDKLANHERVTVHHEELTAFPEGPTVVATGPLTSPGLAEEISALNGSEGLYFYDAAAPILDKNSIDFDKVYLKSRYDKGEAAYLNCPMTEAEFEAFYKALISAEVAEMHDFEDEKFFDGCMPIEVMAKRGIKTMLFGPLKPVGLENPKTGEQPYAVVQLRQDNAVASLYNIVGFQTHLKWGEQKRVFRMIPGLENAEFVRYGVMHRNTFMKSPDVLLPTYQSKQRSDLFFAGQMTGVEGYVESAASGLVAGINAARLALDTEPVTFPATTAMGSMAHYITHTSAKHFQPMNANFGIFEPLKQRIRDKKERNQALADRALASIDTFKETL

>fig|1664.9.peg.1298

MALEQDWINLFLQYLIVDRHYSPETQKAYQADIKAFVDFLAANGGLTSFKQVGTLEVQTYLNEMDQKKYSGETIARRISSLRSFYRYLVRNEFLQTDPFETVQLKKQRHKLPRFFYEKEMDALFAAIVGQEPLTQRNRAILELLYATGMRVSECAQLTIGQIDFGLRVILVHGKGNKDRYVPFGNHATKALQDYLNDGRVQLMAKRQQEHDVVFVNHLGNPITSRGIEYALDQVVKKTCLTSGIHPHMIRHTFATHLLDHGADLRTVQELLGHSSLSTTQIYTHVTTAHLQKDYRQFFPRAKN

>fig|1664.9.peg.1299

MTTICAVKHNGRTAIAGDGQVTMGEKFVTKDSAKKIRRIYDNKVAIGFAGGVADAFTLQEWFEQKLEKYSGDLQHAAVALAQDWRKDPTLQKLEAMLIAINETDILLISGNGEVITPDTLAESGDQVIAVGSGGNFAQAAATALILEGKPELTAEQIVETGVNIAGGIDVYTNHNVIVESF

>fig|1664.9.peg.1300

MDLMNKTPKSIVAELDKFVIGQDKAKKAVAVALYNRYRRMQLSTKMQQEITPKNLLMIGPTGVGKTEIARRLAKVVDAPLIKVEATKFTEVGYVGRDVESMVRDLADVAVHMEESEQFKNVRGKAARQADKRLVKLLVPGIKKEQRKNNNSMNDLMSMFTALQNGETPAGLNQAETEEVTDEVRDQRLSVKEQLDKGLLDDREIEIQVDEPKKAAPMNDMMGQMGIDLNDTLGSIMPKKKMVRTVTVKEAREIFIQEESDKLVNHADIYHDALQRAENTGIIFIDEIDKIAAGGKKQSGEVSREGVQRDILPIVEGSQVNTKYGVLKTDHILFIGSGAFAESKPSDLIAELQGRFPIRVELEDLTEADFVRILTEPNNALVKQYMALIGADGVHVTFTMEAIGRIAAIAFQVNHDTENIGARRLATILEKLLEDILFEGPDMQMGDITITEQYVNDKIGKIVADKDLTRFIL

>fig|1664.9.peg.1301

MIQIANEFLTVTLSPQGAELQSVKGAVSGLEYLWQADPKVWGRHAPVLFPFVGRSKNDQYTYQGKTYPMGQHGFARDRQFEVESQTDTAVTFLLKSDEESLKIYPFEFELRVFYQVNDMHLKVGYDVKNTAQSGPMYFSVGGHPGFKVPLTDDTQFEDYFLDYRPQKSRVKIPLQGAFINLNERTLAPTDVATDINHSLFKDDALIYELKGQENKFSIRSEKTDHAISLKVSGAPFIGVWSPYPTEGELVCLEPWWGIADDVDASGELSEKFGVNQLAPGEVFNASYEITVK

>fig|1664.9.peg.1302

MTDKKEYYGADAIIDSLVNHEVEYVFGIPGAKIDRVFERLEHPVNPKTPQLILARHEQNAAFMAAGIGRITGKPGVVMATSGPGASNLATGVVTATAEGDPVLAIAGQVKRADLLRLTHQSMRNAALFAPITKFSAEVQEPENLSEVIANAYQEAESAKQGASFVSVPQDVTDAKVQSKVIKPLIAPKLGPASPVETTLLAQRIKEAKLPVLLVGMRASSPEVTAAIRNLIAETHIPVVETFQAAGVVSRELETDFYGRVGLFRNQPGDRILKRSDLVIAIGYDPIEYEPRNWNAESDAHIMVIDDMRAEIDHNFQPEKELVGDIAQTLDFLLPYMKGYQLADDVRDYLKELHAELHQRDTAPEMSADKTLNHPLNIVSELQERVTDEMTVTVDVGSHYIWMARHFRSYEPRHLLFSNGMQTLGVALPWAISAALVRPNTQIVSVSGDGGFLFSSQDLETAVRLNLNIVHIIWNDGYYDMVKFQEEMKYGESAAVQFGPVDFVKYAESFGATGLRVEKAADLGKVLDQAFATDGPVIVDIPVDYSDNKLLGQALLPDQLV

>fig|1664.9.peg.1303

MTFDYQRVYQHGTLALLVPGLFTGTLSVADLLTHGDFGIGTAHGLAGEMIILDGQAYQVTGEGQVNQLAPTDMVPFATVHFQAPELPIQTLGTVTKAELEAQILAQQPLKNLFFAVKVDGRFKKMHTRAVEAQQEPYPNLTDATRVQPEFEQDGVTGTLVGYYAPALYQGAAVAGYHVHFLNTVHDFGGHVLDYVIEDAQLTVQPFETFEQHFPSQDTDFLNSDFDLKAINADIEEAEH

>fig|1664.9.peg.1304

MLNSFPQVMTIAGSDSDGSAGAQADLNTFFSRGVYGMSVLTACVAGNSFGIHASQALTPEFINAEFEAIAADFKVRACKTGMLTDSETIMTVAKQLKQHDFGPLVLDPVIITKHGALLLADEAYQTLKTELIPMATVLTPNFYEAQKLTGISLNEPSDFQAAAENLLALGAKNVMIKGHHAAGQSIVTDYVRLADGQEFWLSEPYIETTHINGTGDSLSACICAEIAKGHSIEEAIKTAKKFVHAAIANPIDVGHKYGPINPHVQ

>fig|1664.9.peg.1305

MLQLETKGLNYSVDGQAILSDINITIEQGSYNTIVGPSGSGKSTLLRLLADLLTPTSGEILLAGQAISDYKPTQYRQRVSYCFQQPTLFGTTVGDNLVFPFEIRELPVDETLVQTQLARVGLAEMPLTKKITTLSGGEKQRIALIRNLMFKPEILLLDEITTGLDEQTKSLVNGLITHLNQEDGITVLSITHDTQEQQRKGRQLQIVAGRLVHADESSR

>fig|1664.9.peg.1306

MQMNLAVNGLSLTFAVSLVFVALIISFHEKIGLEKDMLISVGRAIVQLIIVGYLLKFIFQINHVWITLAMMLIIIFNAAANARKRASTLRHAFGISLIAILTSTGVTLGLLVLSGAIKFVPSQIIPITGMIASNSMVAIGLSYRSMLTQFKDQRQAVLERLALGASLKQASIAIVRESIKTGMSPTIDSAKTVGLVSLPGMMSGLIFAGVDPVKAIMYQILVTFMLLSATSLGSVIACYLAYHSFYNDREQLIDL

>fig|1664.9.peg.1307

MKLSLIFILAYLIGSFPSGVIIGRVFCHKDPRDAGSHNIGTTNSYRVLGPIAGTAVLFLDILKGTLAASLPMIFHTSNHSLVLVVGLAAVIGHAYSIFLRFTGGKAVATSAGILLAYNPLFFVIASTIFISVILITSMVSMASIIGPLLIAILSFYTHDWLLGTIATLVLIFLTYRHRENISRIKNGTENLVPFGLYYRYKQKKRQ

>fig|1664.9.peg.1308

MPNQKQDYNDNSIQVLEGLEAVRKRPGMYIGSTDGRGLHHLVYEIVDNAVDEALAGFGAEINVTIGADNSISVRDHGRGMPTGMHQSGIPTIEVILTVLHAGGKFTQNSYKTSGGLHGVGASVVNALSEWLTVRVVRDGVAYEERFENGGHPVGTLKKVGKANEPNGTLITFKPDASIFQVTTYNYNTLAERLRESAFLLKDVKITLTDLRADPAKEDVFHYPEGIQSFVDYLNEDKDTIGNKLYFEGTKEDIEVEFAGQYNDGYSENIMSFVNNVRTGDGGTHEAGMKAGLTKAFNDYARKVNLLKERDKNMEGSDVREGLSAVLSVRIPEELLQFEGQTKGKLGSPQARTAVDNLVYEQMSFFLLENGEFAQALVRKSLKAREAREAARKAREQSRNGKKKKKSDGLLSGKLTPAQSKNAAKNELYLVEGDSAGGSAKQGRDRKFQAILPLRGKVLNTQKAKLQEILKNEEINTMIYTIGAGVGAEFKIEDSNYDKIIIMTDADTDGAHIQILLLTFFYRYMRPLIEAGKVYIALPPLYKLQKGAGAKTKIAYAWTDDELKVVAKDMGKGYSQQRFKGLGEMNADQLWDTTMDPSSRTLIRVRIDDAALAERRVTTLMGDKVEPRRKWIEENVKFTLAEEGSILETTGQTATPVSDDNAVE

>fig|1664.9.peg.1309

MADEAQKIQELTLEEVMGDRFGRYSKYIIQERALPDIRDGLKPVQRRILYAMAIDGNTYDKGFRKSAKSVGNVMGNFHPHGDSSIYEAMVRLSQDWKLREPLIEMHGNNGSMDGDPPAAMRYTEARLSKIASEMLKDIDKKTVDFVLNFDDTAEEPTVLPARFPNLLVNGATGISAGYATEIPPHNLGEVTDGLVYLIDHPDATLEKMMTFVKGPDFPTGGILQGLDGIKKAYETGRGRVVVRAKTSIQQLRGNREQIVISEIPYEVNKAQMVKKIDELRVLKKVDGISEVRDESDRMGLSVVIELKKQADSQGILNYLFKNTDLQITYNFNMVAISDMQPKQVGLLEILHAYLKHQEEVVTRRTQYSLQKAQDRLHIVAGLVKALSILDQVISTIRASKNKKDAKTNLVAQYDFTEAQAEAIVSLQLYRLTNTDVTALEAEAAELKTAVEGYQAILGNPKELARVIKSELKSVVKAYRNDRKTEIEAEIETLEIDTSVTVIDETVMVAVSHDGYLKRSSLRSYQASEGADGGLKEGDFAILQKPMQTLDQLMLFTNSGHLIQRPVHEIADLKWKEMGEHISQSIGLAADEAIIAAFSFTPNEQNGYFVIGTTDGYIKQTAFQDLLPSRTYRSKAQDFIKLHAGANVTNVYWVSETDAQQQAVFSASYNGYGLSFALNEVSVSGAKAGGVKLMDLKDGDHVANFTLFDPTEKHAAVAIITQRGAFKRMFLSEIGLTSRARRGVLILRELKRDSHRVAAMISVAPKISLLVKTEQALLTIEPQAHATGDRYSNGSFVMDVEEVGQPVFVLAQSSEPISNHKD

>fig|1664.9.peg.1310

MVKTEKINLADYWDGFNKGDWQDEIDIRDFIQQNFTAYDGDESFLAGPTEATTTLNDQLMALKKKEREAGGVLDADNDNPATVTSHGPGYLNKDLEKIVGLQTDAPLKRAFMPFGGIRMAEDALEAYGFKTDPEQHKIFSEYRKTHNQGVFDAYTPDMRKARHYKIITGLPDAYARGRIIPDLPRIAVYGIDRLAEEKVRDFNGVGDGEMTDSVIQLREEINEQYRALQDMKKMAASYGFDISRPAKNAQEAVQWIYFGYLAAIKTQNGAAMSVGRIDTTMDIFIQRDLDRGLITEAEAQEMVDHFVMKLRMVRFIRTEDYNSLFSGDPIWATLSMCGIGMDGRHHVTKTAFRVLKTLENMGAAPEPNITLLWSDRLPEGFKRYATEVSIKSSTIQYENDDLMRNEWGSDYYGIACCVSAQPIADGVQYFGARANLAKTILYAINGGIDELGKAQVGPAYQPITSEIIDYKEFMGKYDKMLDWLADVYVNALNTIHYMHDKYYYESAQLALKNSRLDRTFATGISGLSHAVDSISAIKYGQVKIIRDEDGMAVDFEAQNPDYPRYGNNDDRADDIAKWLVKTFYNKMNTHHLYRGSKLSTSVLTITSNVVYGKNTGTTPNGRQAGEPFSPGANPAYGAEKNGALASLLSTAKIPYRYATDGISNTFGVTPNTLGHDDNARKDALVNMVDGYMVNHGMHLNINVFNKQTLMDAQQHPEEYPTLTVRVSGYCVYFADLTKEQQDDVISRTFFDEM

>fig|1664.9.peg.1311

MAIKFTTRLSTETAETVVPEETTEAPIGYVHSIESFGSVDGPGIRFVAFLQGCRMRCEFCHNPDTWNIGSGEPYTADELIAKALPYKAFWGKEGGITCSGGESLIQIDFLIDLFKKCKAQGINTCLDTCGQPFTYKEPFFSKFKELMKYTDLSMVDIKHIDPEGHKKLTGFSNKHILEMIEYMSNHGHHMWIRHVLIPERTDYDCYLERLGDYIKTLNNVDRVEVLPYHTMGIVKYEKMGIKYPLRGIEPPTHDRVVNAERLLHTADYSK

>fig|1664.9.peg.1312

MKTRQENIFSSKTLTYFLQLAETMNYTQAAQLLGITQPALTQQIKKLERTVGAPLFYSVGKKLHISDAGRTMLDATHQIYDLLNTATDEIQSATSASRGKINIGILASMELSVIQSFLIKYYQQFPNIEVGVHLLTRKEIWDRLENNKIDLAIMYLPDDSIKNWKPYRSKKIADESLVFLSNNPLLKKRKKIHFEDTLTEKWVTYPDEYYLNQFISEAYKNQMVDRPVSAAQFTSPFEILRFAEATGLSTALPKSFYDAHNVGIESNAVQFDPAIQFELAFVFRKEKEDIPRIDSFFREFNLYLEEKDYLTRIKEQNRKII

>fig|1664.9.peg.1313

MSKELVFGHQNPDTDAIVAAMAFAYLQQQLGKDVEAVALGEPNEETSYALRHFAASTPRVIKEAGSEVQSVMLVDHNEAQQSVADIKDLTVTDVVDHHRIANFETVSPLFYRAEPVGCTSTILLSMFNENKIEIPAQLAGLMMSAIISDTLLMKSPTTTEKDRIAIKTLAEIADVDFESYGLEMLKAGTNLDDKSEAELIEADAKTFDMAGKKVRIDQVNTVDIEAVFKRQAAFEAAIEADNKANGYDLFVLLVTDILNSNSEALVIGEPAEAFEKAFDVTLNNHRAQLAGVVSRKKQVVPQLTAAF

>fig|1664.9.peg.1315

MMKHTLKLVLTIVVGITALILAFLCQQPLWAQVLVSLAGGLVALSMLIEMIRTLKSGRYGVDLLAITAIIATLAVGEYWAALMVLVMLTGGDSLEDYAAHKAGRELKSLLDNSPQVAHLMRGDGLVDVAVDELQVNDQVVVKPGELVPVDGHIIEGSSLFDESSLTGESRPVERTINEPIMSGSVNGETSITMVVDQLAINSQYQAIVKLVESSAAQPAHFVRLADRYAVPFTLVAYLIAGVAWWLSKDPVRFAEVLVVASPCPLILAAPIALISGMSRASRNGIIVKTGTTIEKLALAKSAAFDKTGTITNGQLTVDQVVPMIDIEPSELCRLAASAEQASAHILARSLLQVVPAAQLLEIISLKEMTGAGVTAQLASGQTVQVGKFEFVAPDQAKQSQTQTTVYIGIDGRYAGYITFIDHLRQEAPQTMSTLHQLGLQRLMMLTGDQATTAKEIAAQVGIDEVHAGCLPADKIRLINNVTPEQRPIIMVGDGVNDAPSLASADVGIAMGAHGATAASESADVVILKDDLSRVSGAITIARDTMRVARQSVLIGIFICIGLMLIASTGVVPALIGALFQEVVDTVSILYALRARNDH

>fig|1664.9.peg.1316

MQHQRLLIQIEGAIIAAFAMALEYIPHTVGPSAIEVSFGIIPIIIYSLRRGAAAGMVSGLIWGLLDLVLRGLGNGSVLNVWQGLLEFTIAFMVVGLAGVLQKPVQASIQQNTKVKTIALLWSAAFIGTFAKYFCHFLAGAIYWGSYAPKGMNAWLYSLTVNGGSFVAGFILNGIVFGILLAMIPKLYLPKDMFLKVQSEK

>fig|1664.9.peg.1317

MDQQRYLELCEQRLADLPDYVRKFYINRQANAYSSATLYQYLQEYVRFFSWLIDEQIVTVQTTKDIPLVALDKMKLEDIELYKIFLMNKRKANNHKLSFQAVNRSINALNALWYFLTIEAEQADGEPYFYRNVMKKVKLLKSSETMATRSRHIGQKLLTGDKKHDFIAFMQVDYPSALSNRQLASYTQNCERDIAICVLALTTGIRLSELAQANVSDLNLEQLTITVIRKGGQQDTVPIAPWGLPYLQDYVQIRQQRYLAANERALFVTKYRGQAKRISNYAIEKLVAKYSEAFGVRLTPHKFRHTLATDLMSATHSETIVATQLGQTTTQATHLYTHVVDDEQRSAMDKLE

>fig|1664.9.peg.1321

MTIKIVTDSSIQLTTAEQERYDIHIVPLTIQQGAESFVDGQTITRAEFLSRLQNATTEFPTTSQPTVGSFVELYDQLGADGSTILSIHCTGLLSGTIEGAHTAAQQSTSDVHVIDSKEIDRGLAYQVVAAAQDVEAGKTLTEIIAHLDEIRSKTETYVFLDSLDALQRGGRISRMAGLLTKLIKLKVIVRLTDTELEVIAKGRGLKAFTKEINNIREHLATQDVAEIGISHVGITDELSDKIQATLEQTQPHVPYTVALTSPVIMSHVGLGAFGIFYLTK

>fig|1664.9.peg.1322

MMTNKRQQIIYEVWNAITHGVGFFGSLALVILLIVKSVQKSLSPVAIASLIVYGATLLALYLASTLFHCLAFTKAKRVFQIFDHSNIFLLIAGTYTPYCLIFIGHTAGISLLIAIWTLAIAGILTHIFSHGRYQRLETSIYVIMGWLCLLAGKTLYTNLSPSGFWLLVSGGIVFTVGALIYSFPKIPGLHLIWHFFVMLGTTLMFFSIYLNI

>fig|1664.9.peg.1324

MKIAVVTDSTAYLTEQQVKDHHLYIVPIPVILDGQVYDEGVDITSEEFYQKLKTAETFPSTSQPPLGEMLKLYEDLGNEGYDAVISIHLASTISGFVTTLKNAASTIDNIQVVPYDSGITVMLMGYLAMEAARMARDPEVTVEQITARLDDLRSTIDECFIVNDLQNLVRGGRLSNASAFIGSMLKIKPLLTFDDETNKITAFEKVRSLKKAYARAEQIFEETQERVDYPLRALIIHANDEAAALSWRDKLREKHPDLPIDISYFGPVIGAHLGEKAIALAWIKDFTKE

>fig|1664.9.peg.1325

MTFDGLMTHAVVNELNTTLADGKITKIQQPYANEVILTIRANRKTYPLLLSAHPSYARVQVTRVPYTNPATPPNFAMTLRKYLDGAILEQIEQVESDRILRLTFKSRNELGDMQKLCLIVELMGRHSNVILINLETQKILDLIRHVGVSQNRFRFLMPGADYVNPPEPTGLNAFTIDLGTLDWLSYYNEDADEQIHWLQQQFQGMSRESALELQTRLMDHDNYLAVVTDFFKQFDQAQPTLTIGEKKSWFTAFPYQSLTGTQSHFETLSELLDQYYLHKAQRDRVQQQGGALIHFIRNEIQKDEKKLKKLQKTLKAADSADDYRIKGEILTTYLRQVERGMTEVTLPNFYDNNEPIKITLSNQISPSKNAQKYFSKYQKLRNSIAYVNDQMAKTEAELQYFYTIMAQIEIAEPKDLEDIKAELQAEGYLKIKKTGAKKQKRYKISEPEVFYATDGTRIFVGKNNYQNDQLTLKKAHKTDIWLHVKNIPGSHVIIDSPTPSEQTIQEAGNLAAYFSKARLSAAVPVDYIQVKKIKKPNGARPGFVIYEGQQTLYTTPDEALVQQLRNKPVQA

>fig|1664.9.peg.1368

MAQKDNQKEMNDQLKVRREKMQFLKDEGIDPFGSRFERTHLAAALHEEFEAIEKDDLDVKNQEVTIAGRMMSKRGKGKVGFADIRDRSGKIQIYVRKDEVGEDNYKIFKKADLGDHLGITGQIMKTDMGELTVKATHLTFLSKALRPLPDKYHGLTNVEQIYRQRYLDLIANPESMDRFTKRSKIISAVREYLDTHDFTEVETPVLHGQAGGASARPFITHHNALDINLYLRIALELHLKRLIVGGMERVYEIGRVFRNEGIDTKHNPEFTMLETYAAYFDYKDVMDETEGIIRFAAHKVLGTGQISYQGQAIDLDGDFARVHMVDAIKAETGVDFWQPMTVEAARELADQHHVKYEEYWQVGHIINAFFEEFVEDTLVQPTFIYGHPVEISPLAKKNAEDDRFTDRWELFMHGNEYANAFTELNDPIDQRERFEAQAKERENGNDEAEGIDEDYVEALEYGMPPTGGLGIGIDRLVMLLTDAASIRDVLLFPTMRPDKQENEEI

>fig|1664.9.peg.1369

MTWQIGDVTIPNQVVVAPMAGITNAAFRVTAKEFGAGLVVCEMISDRGIMHNNQKTMGMMFVDPNEHPVSIQIFGGSKDTLVEAAKFVDQHTQADIIDINMGCPVNKVVKTDAGARWLLDPNKVYEMVSYVTDAVKKPVTVKMRTGWDEDHILAVQNALAAERAGASALAMHGRTRKQMYTGKADWDILKQVKAELKIPFMGNGDVRTPQDAKRMLDEVGADAVMIGRAALGNPWILRQVETYLRSGELIAEPTPREKIATAKLQLHRLVELKGENVACREFRQQAAYYLKGIPRAAKTKAAVNEVETEQAVGDILDRFVEETEARMA

>fig|1664.9.peg.1370

MKDYLVKQVSEDGQLRAYAVNATQVVTEAQEKHDTWPTSSAAFGRTIVGTLLLSAAGLKGDTKMTVKVEGDGPVGKIVVDGNAQGTVKGYVTNPHVNLPSNEKNKIDVKAGVGTTGTLSVTKDLGLKEPFTGQVPLVSGELGEDFTYYLAKSEQTPSAVGVSVFVNEDSTIGVAGGFMIQILPGADDRLIDILEARLQEMPLVSELLQQGMTPEEIITEIVGELPMKTLEELPVKYECDCSKERFAKALSSIAPQDLKQLIEEDHGAEATCRFCGKQYQFSEADLKAILAEQ

>fig|1664.9.peg.1371

MNNKKNRLFSNSLFYIIILVALVGVFSFFMNGNSGSESKEVQSSTFLSELKTDKVKSFSIQPTNGIYKISGEYRKAQKDEQPKSTFFGSQSKNKKVTHFTTTLLQSDSQVSAITNLANQNKTKIETMQEPQSGWWVSILSLILPLIIMFGLFYMMMGQAGQGGGQGGRMMNFGKSKAQKADKGANKVRFSDVAGAEEEKQELVEVVEFLKDPRKFAALGARIPAGVLLEGPPGTGKTLLAKAVAGEAGVPFFSISGSDFVEMFVGVGASRVRDLFEQAKKSAPSIIFIDEIDAVGRQRGAGMGGGHDEREQTLNQMLVEMDGFSGNEGVIVIAATNRSDVLDPALLRPGRFDRKILVGRPDVKGREAILKVHAKNKPLADDVDLKEIAQQTPGFVGADLENLLNEAALVAARRSKKDIDASDVDEAEDRVIAGPAKRDRVINPKERETVAYHEAGHAIIGLVLSDSRVVRKVTIVPRGRAGGYAIMLPKTDQFLMSKKELTEQMTGLMGGRTAEEIIFNSQSTGASNDFEQATDIARGMVTHYGMTEKLGTVALEKEGQPFVGAAYGQGPAFSEATAAAIDSEVRRLIDEAHQQATEIIQAHREQHKLIAEMLLKYETLNEKEILSLFNDGKMPEKNAEEFPSEKAATFEEAKKALEAKEAEKTDSELEAEQADSETTEVADDTADSKPTNDDSNDNA

>fig|1664.9.peg.1372

MLEQDIERILYTREDIQRVNEKLGKQITADYEGKNPLMIGILKGAIVFMTDLIREIDLHVEIDFMDVSSYGEGTISSGEVKIIKDLDTSVQGRDIIIVEDIVDTGRTLNYLMSILKTRQANSIKVCTLMDKPSRRVVPVHSDYVGMEVPNEFVVGYGLDYAERYRNMGTIGVLKPEIYSGK

>fig|1664.9.peg.1373

MLEQAFQNEIRRHQFWPTGAKVIVATSTGVDSMVLLTLLRCLPTALKPQLIVAHVNHELRTESVAEEAYLRQFCLENDLSLQVAHWPLQQHPQTGIEAAAREFRYQFFEHLMTAEKVDYLVTAHHGDDQLETLMMKFIRSGELHEMRGIQSQRPFADGTLIRPLLPFAKQDLRDFALANNITSFEDQTNYETTVLRNRVRHTLVPFLKQENGQILQNANRFSQQLTALLGQQAQLATTLLPLLDLKVTDKVISGQLKAIRTLPTAQQTAIWQLIIKQHFPQISPLKETQLQQLKQLIQSTVKPQGQLALGQRILFTKIYDRFQIGETTIGEIAAKDEKSRTLMLNKWCVLPNNEQIGIFEINNLPRKLSGQQLIWLADEQWPIVAKPCQLTDTIMIDKTHHKTLKRLFIDLKVPHEKRQNSWGIWSQETLIGHPEFRISALFNHEQTGKIRYVLCYISE

>fig|1664.9.peg.1374

MAVEVGSKVPGKVTGITNFGAFVDLGEGKTGLVHISEVSDSYIKDIHDVLTVGDEVTVKVMSDQNGKIGLSIRKAVDQPKVQEHTRPQSSRPNNNRGGYRKPEHTSAPKKEGFDDLLSGFLKDSEDRLSTIKRNTEGKRGGRGGRRS

>fig|1664.9.peg.1375

MSRKITQIENAYTKQQAAKQKVEQKSKRTRTVHKRRLLALGVIALILFSVCGVQILQNHHSLALIEKQTVVKKQSLKKAKAKEADLKVQVSQLHNDDYLAKYIRYKYYYSKKGETIYSLPQDKAPNLNEQQK

>fig|1664.9.peg.1376

MRLDKFLKVSRIIKRRSVAKEIADKGRILINDRQAKSSSNVVVGDKLEIGFGNKTMIVKITQIIETTKKSDAAEMYEILETKVAENFE

>fig|1664.9.peg.1377

MQNKQMQHLMKGAVVLSIASFVAKILSAVYRIPFQNMVGNTGFYVYQQVYPIYGIGMTFALSGFPVYISKIVAEETAPAEQTQILRRSFALLGLFGALIFLYLQYQAPAIAVAMADAELAPLIRMVSYMFLLMPFLAVTRGYFQGIFNMIPTATSQVAEQLVRVVVIILAAWLSLKLHWSVYEMGTWAMSGAFFGGLVATVALLKPAEKAFVLRGTKPKGINLGAYRSLAKRLLIEGGSICLFASLIVLLQLVDSFTVKKGLVASGLTEDFAKNLKGIFDRGQPLVQLGLVISMAFSSTLIPSLSRARQQQQDHQFQQVAESLIHISLGLSAAATTGLMILMPQVNYLLFGDADGNKALVWYMLSIVIIALINACNSVLQSLDQFHKTTIALLIGLLVKVVINQWLVQHFQIVGASVGAVISLGVVLALILRQSPELVRDALDGQHFTGKLVLICGIMAVAVRFVVTVIQPSLVSRGQAVLGAGVGIAVGVPVFISLALAWQLFSIREWLTIPGGKQLLKLAQKLKR

>fig|1664.9.peg.1378

MDLISMLGNTQQVQSVLENQKPGVRQLLTGLSGSAKTLFLATIYKQQRQPLLIIESNMFQANQVAEDLANQLNGDQIYTFPVEEVMAAEIAVSSPESRAERVRTLSFLATGKKGIVVTSVAGMRRLLPTARQWRDSQTQIEMGGEVDPKILGAQLAEMGYHRDKLVGKPGEFAMRGDIIDIFPLDTENPVRIELFDTEVDAIRSFEADTQRSIENLESVNIMPATDLLANVAQLEIAGEALQADYQQTAAKITAKDDQKALAVNFETPISRLLAGERLENLALFVDYLYPDHTSLIDYFKNSGLVIFDDYPRIQETQRVLAEEAANWQTDMLGNRRLLPAQKLLVDVHHLMKQDQHPHLYLSLFQKGMGKLKLDTLGNMPTRNVQQFFSQMPLLKTEMSRWQKQQQTVVVLVSDAKRVKKIDQTFHDFEIEATVTTKTKLVAGQIQIVQGSLQNGFELPDLKLVVLTEKELFNTAPKKKVRRQTLANAERLKSYSELKPGDYVVHVNHGIGEYVGMETLEVDGVHQDYITILYRDNGKLFIPVTQLDMVQKYVSAESKTPKINKLGGAEWQKTKSKVSAKIEDIADDLIELYAQREAEKGYAFPKDDQLQADFENQFAYPETDDQLRSTAEIKHDMEKVRPMDRLLVGDVGFGKTEVALRAAFKAVAAGKQVAFLVPTTILAQQHYENMLARFADFPVELGLLSRFKTRKEVTATLKGLEKGQVDIVIGTHRLLSKDVVFKDLGLLIVDEEQRFGVKHKERLKQLKAQVDVLTLTATPIPRTLHMSMLGVRDLSVIETPPTNRYPIQTYVMEQNAGAMREAIERELERNGQVFYLHNRVSDIEQTVDEIQALVPEATVGYAHGQMTEAQLEGVIYDFVQGKYDVLVTTTIIETGVDMPNVNTMIVEDADHYGLSQLYQLRGRIGRSSRVAYGYFMYKPDKVLTEVSEKRLQAIKDFTELGSGFKIAMRDLSIRGAGNLLGKQQHGFIDSVGFDLYSQMLSEAVAKKQGKKVAAKTNAEIDLKLEAYLPDDYINDQRQKIEIYKRIRQMDTEAAFTEIQSDLIDRFGDYPVQVAHLLTIGQLKMNADDALIETIKQVKDKIQVVLSPKGSRLVSGEQIFEALSVTRLKATVGFEQEKLSVTLIIQPKMKTGDWLRELAIFVETLVEIEKKQLVK

>fig|1664.9.peg.1379

MKMIVGLGNPGSKYAKTKHNIGFMVIDQLCEKYNVTLNKHDFEAEYGSFKYEGETVLLVKPLTFMNDSGRSVCPLMSYYQVGIDELLVIQDDMDLTMGKLRLRQKGSAGGHNGIKSIIAHTKSQTFKRLKIGIQHPQKSTVVNWVLTPFDKDGAPVINQAIDQACEAIDDWCQNDDFMKTMNKFN

>fig|1664.9.peg.1380

MANIEKDHQKVILVGDGAVGSSYAYALTLQGIAQEVGIVDIFKEKTQGDAIDLSDALAFTSPKKIYAAEYSDAKDADVVVITAGAPQKPGETRLDLVSKNLKILKTIVDPIVESGFNGIFLVAANPVDILTYATWKLSGFPKERVIGSGTSLDSARFRKDIAEMVNVDARSVHAYIMGEHGDTEFPVWSHANIGGIKISEWVKAHPEVKEEELVKIFESVRDAAYTIINLKGATFYGIGTALARITKAILDDENAVLPLSVFMDGQYGLNDIFIGSPAVINRSGITNILEIPLTDHEMESMHKSAKQLKDIVTKAFEELDIETRQ

>fig|1664.9.peg.1382

MKVSKKAYTALGVAAVILIGGYSAMSLHYNKVFLPNTVVAGTNISGKSPAEANEALLKQVNHQKFTLKEDQTTRLSFTAKDAGFSHNFQPLLKKIQSSQNGWSWLGHQLSNPKKLQSENALILNQQTFKQFANQTVAKLNTGRTASKNATIKLVNGNFEIVKEVNGNQIDQAQFGKTINKAINQGKTSIDLKQDYTVPTIKSTDSALQKTAKKMTTISDEKIHYSVNGNEFTVPKATIQGWLVNNNGAVSLDKTAVSNYVYQLKTKYDTYDKPLTFKSTKQGTVTVPAGIYGWSISPADEISALMKEVPEGKDFSRTAVIRGSGMTQKDRSVGSTYIEIDKKAQHMWLYQNGKVTIQTDVVTARPPQTTPSGVWSIWKKERNATLKGTNFDGVSEYASPVNYWMPIDETGVGIHDSPWQPKYGGTWYKEHGSHGCINTPPTIMAQLYNTVSEGIPVVVI

>fig|1664.9.peg.1383

MLIKSLVLPKERLTTIEESVTLEEALTILEDSGYRCVPVLDASGKIFRGNIYKMHIYRHKSQGGDMQLPVTSLLKNMTKYIPVDAPFFKVFFNIKDLPYIAVLDEDSNFFGILTHSTLLNMLSEAWNLELSSYVLTVLSAGEQGDLVAMSKIIAKYTSIASCMTLDAKQDEFVRRTLFTLPSGLDIEILKKITNKLEKKGFKVPEIDDLQSGKVLRNDSDEIENVI

>fig|1664.9.peg.1384

MTNQNRLRSVILNGLVAALYVVLSLFPGVLGLASSPIQFRVSEGLNHLVVFNKKYLWGVFGGVLVFNFFFSSENKILDTLFGGGQTLLALLVLTLVMPHLKKTWQKMLVNIVLFTVSMFMIAWMIVLTGSTAKTQIPFWPTYATLAISEAVIMTITAPIMYAVDQVLHFERQI

>fig|1664.9.peg.1385

MGELIQRGDIYYADLSPVVGSEQGGMRPVLIIQNDVGNHHSPTVIVAAITAKIQKPKLPTHVGITAKANGVERDSVILLEQVRTIDKQRLKDKVTHLVAEKMRQVDRALTISVGLQPVLNP

>fig|1664.9.peg.1387

MTVGYLRPTRILVDQNAIYENIQNELKHLEGTDTVIFPVLKANAYGHGLIPVAEAAQAAGAAGFCVALLDEALALRRANFTEPILVLGITQPSEIELAAANQISLTVGSLEWLQEATAIAQQVPYLHPVPIHLSIDSGMGRIGMRDEAELLAAYTFIKAHSDYFDFEGVFTHFATADDPDDTYFKEQSARFNQLMTALPERPRFVHVSNSATSLWHAACNGNIIRMGISLYGLNPSGNAIPDLPYPLKPALGIESELVFVKQVAAGTKIGYGATYEASKGEWIGTIPMGYADGWLRRMQGSTVLVDGQRCEIVGRICMDQMMIRLPKRYPVGTKVVFVGKSGDDEITLQEVADYADTIHYEIICDLSDRIPRVYTGLNEH

>fig|1664.9.peg.1388

MIVGLGIDLAEIDRFEKAQEKNSRFAEKVLTATEFELFSHYTGRRALEFLAGRFAVKEAFSKAYGTGIGQVKLQAVETLNDPQGKPYIKQDLFDGVVHVSLSHTATLVIAEVILERG

>fig|1664.9.peg.1389

MKFSELGLSEPIMKAISRAGFEEATPIQGETIPLALAGKDVIGQAQTGTGKTAAFGLPILQNLDLDNPDIQALIISPTRELAIQTQEELYRLGRDRKAKVQVVYGGADIRRQIRSLKDHPQILVGTPGRLLDHINRRTVKLDHVKTLVLDEADEMLDMGFVDDIESIIKQVPEKRQTLLFSATLPAPIMKIGKSFMTNPEMIKVKSKELTADLIDQYYVRAKDFEKFDIMTRLFDVQSPELTLIFGRTKRRVDELTRGLKARGYNAEGIHGDLSQQKRMSVLKSFKAGRLDVLVATDVAARGLDISGVTHVYNYDIPQDPDSYVHRIGRTGRAGKSGVSVTFVTPNEMEYLRIIEELTKKRMLPLQPPSEKEAFKGQLAAAVADINSMIEKGQLAKYNEEAEELLKQFSDVELASVLLKTISKEAVPVKITPERPLPSRKKGGNRGGNRGGSRGGNSGNRRGGNGGGYRGGNRRSSGDGKRRGNDSSKDGRRSSSNGDSSKRRSNDNRGDSRKKTDGRSKRNFTIRTNDK

>fig|1664.9.peg.1390

MKMQLSEIARMTGAKNETEQWATVEVTSVAFDARELRPGALFVPLAGNRDGHDFINQARENGAVATLWASARDDQPTDFPVLLVKDPLAAYQQLAKRYLLKINPKVVAITGSNGKTTTKDMTAAVLGSQYNIHKTQANFNNEIGVPQTILSMEANTEILVIEMGMDRPGQLHALSEIAMPDVAAITMIGEAHIEFFKTRDKIADAKMEITDFLKEDGLLVYDGDEPLLNERVGDLERETFGSATTNTLYPIQVRAGETKTQFKLNAWPDVAFEIPMMGAYNVNNALAAILIGQHYHIKPEVMSKALANFEVTRNRTEWLVGTAGQRMLSDVYNSNPTAAIEVLRNFSHFETDGRRIAVLGDMLELGTASKKLHESLATVLDPAQIQEVYLFGTEIKPLFDKLATIYPAEQLHYFEKTQQPELITALAETIQPSDLVVLKASHGLHLEKVVAALTTEND

>fig|1664.9.peg.1392

MLYEQIAQNKRRTVYVMIGFLMLVLAIGAALGYLFMQNTLTGVIIAAVLAAVYMVMMISNSTNVVMQMNHAHEIKDASQYPDLWHIVEDMAMVGQIPMPRVFIVDDDSPNAFATGNDPKNAAVAVTSGIMARLNREELEGVIAHEVSHIKNYDIRLSTIALALAAVISLLVNIGSNMMWWGGGRDRDDDREGGAGNVIALVFSILLMILGPLAASIAQMALSRNREYLADASGVELTRNPEGLINALLKISNSEPMQAADPSSAALYISDPFKGRGSFGAHLFDSHPPIEQRVERLRNM

>fig|1664.9.peg.1393

MLWLVVIIILVLIVGTVAFYISTYNGMVKSRNWADESWSQIDVQLKRRNDLIPNLLSTVKGYAKYEQETFTKVVEMRNQLLEMPSDDRQATMAVSNQLSESLKSILSLTESYPDLKANAEYMKLMEELTNTENKIAYARQLFNSSATRFNNKIETFPGNIIAGAHNFQKTAYLATPEVEKEVPKVSFD

>fig|1664.9.peg.1394

MFSAHPTIADSGLIDPRFIKPNAALPADYLALCQETNGGFLSRFSLPTSEPTSDGLDHVECHYLAGIATGSQAVIEVAQWPDYLIPFSQHGTQYFAFDYQQSPDNPSIRYIDTEVDQWLTVASSFSQFLAQLGTKPIVVPETDDLTLTPLQRNHYLLIAPAEQLMALLALYEADSPKDWYLDWLLFFAQSGTLEQQKCALDAYHTQRLYFKRQLPQAKSQQLAQIFAQQPALLATYQAYQKQWSPL

>fig|1664.9.peg.1395

MLTHIRQRLFRPREQWEQNLVVLWFGNFMAGVAFSLIMPFMSLYIDTLGDFSAAQLSMWSGLTYSITFLVTAIISPWWGKIADRRGRKLMLLRASLGMAIVLGAMGLVQNVYELIALRLLQGVFSGYISNSNALIATSAPQEKSGQALGTLTTGTVSGTLLGPLLGGVVAEMTGYRTTFFITGTILFVVFLLCLAFVHENLTPIEKGDQVPAKQLLQNLKYPHLIIGMFVTTMIIQASNNSISPLLSLYVRQLMDHGKNVALVSGIVAAIPGIATLIAAPRFGALGDRIGSDKILIGGLIFAVFVYIPQAFVQNVWQLGALRLLVGIADAALLPQVQTILAKYSPHNSAGRIFSYNQSFQAMGNVAGPLIGSSVSGFFGYGGVFISTTVLVVINLFWVNQNTRVLRKNRADAAKKTS

>fig|1664.9.peg.1398

MKIDRLQAFSDGVFAILLTLLVLDFKLPNYKIGHLGQAVIAQWPILAAYMLSFFYVGTLWLFHHDYFAKIKRTNWQLNILNLILLFAITLINYPTSLLSEAVAQRNLADIRFAFTSYALVALVISGLFWVLYRFMAVHQDLMIHQEQHEQFYRAIRNDPLRSVLIYVIALVGIQFNVVVGALLILLGNIFHFIAYLRLSRNIEKHHLIE

>fig|1664.9.peg.1401

MKQGIHPDYHKVVFMDSSTGFKFISGSTANSAETVEWEDGNTYPLIRVEISSDSHPFYTGKQKFTQADGRVDRFNKKYGFADKNAAK

>fig|1664.9.peg.1402

MKKMLIQGGNLLSGEVTIGGAKNSTVAIIPAAILAESKVVLDSVPHIKDVNSLLSILEDMSVTSTFTGDTVEIDPTNIVSTPLPSGKIQSLRASYYFMGALLGRFGKAIVGLPGGDDIGPRPIDQHIKGFEALGAVVTNNHGAIEINAPEGLHGAKIYLDMASVGATINLLLAAVRAEGQTVIENAAREPEIVDIATFLNNMGAKVRGAGTDIIRIEGAAHLYGHNTHTIIPDRIEAGTYLSMAAAIGDGVNVKNIISEHMDAYLAKLEEMGVKMDVKEDSIFVYPSPDLKMVQVKTMPYPGFATDLQQPLTPLLLKAQGEGLVVDTLYPKRTRHVPELIRMGADISIENDVILLHHADKLQGAEVSADEIRGGACLMIAGLMANGVTTITNASNILRGYDRVIDKLTGLGAVVKMVEE

>fig|1664.9.peg.1403

MTKYVFVTGGVVSSLGKGIVAASLGRLLKNRGLKVTIQKFDPYINVDPGTMSPYQHGEVFVTDDGTETDLDLGHYERFIDNNLNKYSNVTTGKIYSEVLRKERKGEYLGATVQVIPHITNMIKEKIMRAATTTDSDIVITEIGGTVGDIESLPFLEALRQMKADVGAENCIYIHTTLVPYLKAAGEMKTKPTQHSVKELRGIGIQPNVLVCRTEKAIPDDMRNKIAQFCDVEPEAVVESRDAESIYDIPLLLKNQGLDDFVLNHFKMTAPEADMTEWIDMLHTIKNLEGTKKIALVGKYIELQDAYISVNEALRHAGYVYNTDVKVTPIQSEDITKENVAETLAGFDGIIVPGGFGDRGLEGMILSIQYARENDVPYLGICLGMQMASIEFARNVAGITDATTGEVHPDAEHKLIDIMSDQKDLENMGGTQRLGLYPCKLKPGTKTAEAYDNQSVIQQRHRHRYEFNNEYRDLLTDKGLVFAGTSPDNRLVEVIEIPENKFFVAAQYHPEFLSRPNKPEGLFKAFIGATM

>fig|1664.9.peg.1404

MVQSTETILVLDIGGSAVKYGFWTAQKLEDREQFPTPLTRNKFYQTVADICDAHEPIAGIAVSCPGEPDEDTGIVHGMSYVPFLHLGEFQHDFATQLNRPVSLQNDAESAALAEMTLGVGQNHQNALFTIIGSGIGLAIVQDGLILKNLAEKFDNPEKRFADTLKTLNNSKVSPVQIGKTVSLKNFKLPNTIDGKTVFELAKQGDPIASQEVDQMYASLAEILIFLNAAYQPEIIGIGGGISNNPDLLPELKAHIATLLASPDSKISFYKRFYPNPDQAFTKPNLRTCYFKNDANLIGAALHYEHRFKRPY

>fig|1664.9.peg.1405

MKLDVFKGQNKEELSMIEVAHAILEGKGDTIAFADLTNEVQNYLGKSDEEVRARLSQFYTDLNEDGSFISLGENVWGLRSWYPYESVDEEVNHPEDEEDQPTKKKRRKKVNAFLADVADDDDVIDYSDDDPEDEDLDVEDEAELVVTDDDSSDDLTEYKADLQSIGDDDDDEDDALEDGIEGQLSEFSEDDEDEDDDEDDEL

>fig|1664.9.peg.1406

MASKKSTPIKIHLETEITQDGQTESHVFDEEGELIQMGTTTYLRYLEHAQGQETAVRFKLAEDGQVQLTRGTEKDATQLRLYFKMHDAQGSLYQTQYGNLPVLTTTNHLLMTLSDEPLSGEVVVHYQLEISQQVVGDYKLRLIFNA

>fig|1664.9.peg.1407

MQDLATLYPQKTWSVVNQQFSADQGQLAFAHANTLLALTKECQQPIIHFWTLEKTVILGMMDTKLPELTAATDQLTAAGYHYFVRNAGGLGIVADTGILNLGFYLPEVADHLSINAAYELMKTLFSETFNTSNITIEHFEVQHSYCPGEYDLSINGQKFAGIAQRRSKNGIAILLYASIEGDQQARGTLMQHFYQAGKAQQQTRWTFPDVHPETMANLADLLHQPLTVASVSQQLSASLQSNSGLTLQSDLQTLMQEADYQTQLTKNTQLLRRYQPGYQP

>fig|1664.9.peg.1408

MTYAPQKLTREKVFRDPVHNYIRVDYQVILDLIDTPEFQRLRRIKQLGTTSSVFQGAEHSRFTHSLGVYEITRQICDQFQINYPTKQPGDGLWNDNERLVALCAALLHDIGHGAYSHTFEHIFDTDHESITRQIIMTPETKVNQILSQVAPDFPAKVASVIDHTYPNPQVVQMISSQIDADRMDYLLRDAYNTGTKYGEFDLTRVLRVMRPYANGIAFLANGMHAVEDYIVSRFQMYQQVYFHPVSRGMEVVLQKLLQRAKFLYEQPDHHQSLAPQLLIPFFEHDFTLRDYLKLDDGVLNTYFIYWLDYPDTVLNDLADRFLSRRPLKSVKFNPSTEDCLPEMRALTEQAGYNATYYTAVNNSFDLPYDAYDPSAKSPKTQIEIIQSDGQLNELSSISNLVAAISGKFSGDERFYFPKEMSDTANLEVFAPIYQQFNSYINNGAIQAHPHNQ

>fig|1664.9.peg.1409

MSIKLVAVDMDGTLLNENNVLSPKTIEVVKAAKAQGIKVVLCTGRPLTGVTPFLHELGLTDSSDYVITFNGALVQNTATGEILVRHTLTHAQYLELETLSRTIGVHLHAEDDQFIYTANRDISPYTIGESALVNMPIKFRHVDEIAPDKAFSKVMLIDDPEVLAEGKAKIPADFFDRYQFVQSEPYFLEVLNTKAGKGNGLRDLANALNIDQSEVMAVGDQGNDLSMLAYAGLPVAMDNAIPELKKIAKVITKSNHLGQDGVAYAIEKYALNK

>fig|1664.9.peg.1410

MFFYLFGFLLFLWNAYIVWKKESHTLSNMLTLFIGLGLIALFFVPMLANLLHVPEDLRIFIGTISTVFLLYIPLFFYLYLSSLIIYLFNRPRYNQDYIIVLGSGLIDGQYVPPLLASRIERAMTFYHRQVKKGRPAPKLVFSGGQGGDEKLPESVAMQRYAIDHGIPVEQTLTEEKSVNTLQNMQFSKQIIEADTDKDKPRIIFSTNNYHTFRAGLFAKQAHLKADGIGAKTSKYFLPNATIREFIAILSMKRHQLLFVTICSLLFAILAVLLNHLN

>fig|1664.9.peg.1411

MNWIVQLYQPFFELNNWQTVIRSSEDWLLILTLVIMECLLSVDNAVVLAAQTQSLPTKIEQEKSLVYGLWGAYLFRFIIIGVGTFLIHLWEIKVAGSLYLVYLVYAYFRKQKQPKQVKKPGSKISSPHRFWRTVISIELMDIVFSIDSVLASLAISSNPVIVLIGGMIGIICMRGIAQIIARLMEKIPELNPMAYILILFIAVKLFLSIPAIDIEIPNLVFAGIVFGTILITLGIHYIRKQRA

>fig|1664.9.peg.1412

MIKEFKEFIMRGNVLDMAVGVILGAALKSIVDSLTKNLINPIISLFVGQVDLSGIAVTIPGTKAVFQIGNFLNDVINFLIIAIIVFLIVKGFNKLRDMGKKTEEEVAEEAAPTQEELYLKEIRDLLANKDHQ

>fig|1664.9.peg.1413

MAIIEHIKQQYSFLSPQEQKVALAVIQKGASIQRIGIDELAAELQVSNSTISRFVRKIGCQNFVDFKLQFSEGPQPTMTGIQGQVDHESQTSVEIYQFYQDVITKTQARIKQADLDQLVSWIKTSQQLFIYGLGSSGYTGAEFGQRLTRMGIQATVVTESHMMLMTSRIINKTDLVIGLSNSGNTEEVNQAVQNARENGAKTAAITSGTDSPLAAASDLTLFVEDSIGFASARFVNSQFALTYVIDILAMLLLEDEQYNWRMKQTVDTIMHHKLKK

>fig|1664.9.peg.1414

MKDLTKYKGIIPAFYACYDEAGEISPERVRALTEYYIKKGVKGVYVNGSSGECIYQSVEDRKIVLENVMAVAKGKLTVIAHVACNNTKDSVELAKHAESLQVDAIAAIPPIYFKLPEYSIANYWNTISAGAPNTDFIIYNIPQLAGVSLTTELYAEMRRNPNVIGVKNSSMPVQDIQMFCAAGGKDHIVFNGPDEQFLGGRLMGAAAGIGGTYGAMPALFLKLNTLISELKLEEAKDLQYAIDEIIYKMCSGHGNMYAMIKEILRINEQLDLGSVREPLAPLNEADLEIAKESATMITTAIEKYC

>fig|1664.9.peg.1415

MNLKNNFLDKVKDRLIISCQALADEPLHSSFIMARMARAAYEAGASAIRANSVVDVQAIMDTVELPVIGLDKVDYSDAPIYITPTIKEMRGIAATGAAVVACDVTGRPRPHGEQLATIVETMRTEYPDTLLMADTASLDDVKEANRLGFDIIGTTMYGYTPATEGCNIADNDFEYLKQVLAMSKAPVIAEGKIDSPEKAVTALKLGCHSVVVGSSITRPQLIAKTYIDAVNEL

>fig|1664.9.peg.1416

MQKIGFGTFNWIVLCVYLLAMLLVGVYFTKKASKSTDAFFKAEGKIPAWAAGFSIYATTLSAITFMSTPEQAFLQDWSYSVGSLAIIILIPILIKFYVPFFRKLSVTTAYEYLEHRFNPLMRIVGSILFMLYHIGRVAIVIYLPILAVTSVTDINPVLIAVIVGGLCIIYTFLGGIEGVIWSDVIQGILLLGGALLVSMLGAHYIKGGWGTVFNDAMADHKIISGLDFNASVLSRFIPLIFAGQFFNTLYQYTGSQDVIQRYQTTSTMKETVKSLWTNGLLAIITVPIFYGMGTVLYSFYSRAESLPKGFNTTALVPYFIIKTLPAGIAGLVIAAIFAAAQSTVASSLNAISSCAIADFKVRFFNDKFKQYSDVTWARVIIIISGLISLAVAIYLMLSDQSKTLDLFMTITGIFGVPLAGIFAVGIFTKRANATGALIGLIASAVLTYFAQMASISPFVVSCVSFISAFVISYLVSLVFKKSNKDITGLTVQTISETYKG

>fig|1664.9.peg.1417

MLTQASYLTFDIGGTTIKYGLIDENLSLTHLGQLETQQNQNGMIMQQLIQTTRKVMTTYKIIGIGVSTAGIVNRELGTIIYAGPTIPNYIGTEIKAILSHEFTLPVYVENDVNAALLGEKLVGAAQDANNIYCVALGTGIGGAHLLNNHLQDGGTAQANSIGYLLYDSATQTNYEQRASTLTLQAQLSAQLHLSVPEAFDAAKRGESLPLEIIKNWSRSVAQGLAQIILIVDPELLIIGGGVSKQEKFLLDLLNQSIPAYLPPNFYRTKLVTAHNFNDAALFGAVYRFFN

>fig|1664.9.peg.1418

MTKILINTIVFQKEIHSGQSQYALLNQLTAKIAGFEVRGELFKPETKHDELVQLQQLAEQRHWEFRYSIPECLFIKSEINPNLESHFKLAQQFGITALKISLGELEHITAQQVSLLNQLITTYQVQLNIENEPNQNGILNHMAMICLELTNYNSLTGYTFDSGNWYWINEAPLTAWHQLAPYTTVLHLKNIQAQETVLLDNGLTPWRQLCESLSPNIPIILEYPMSNSELESELTLVASALEA

>fig|1664.9.peg.1420

MSEQYFDPKLKIFALNSNKPLAEKIAAEVGVPLGKSSVKRFSDGEIQINIEESIRGDEIFLIQSTSAPVNDNLMEILIMIDALRRASAKTINVVLPYYGYARQDRKARSREPITAKLVANMLTMAGADRILALDLHAVQIQGFFDIPVDHLMGAPLLADYFLSNDLAENAVVVSPDHGGVTRARKLADFLKTPIAIIDKRRPRPNVSEVMNIIGNVNGKRAIIIDDMIDTAGTITLAAQALKDAGATEVFACCTHPVLSGPAIQRIEDSVIKQMIVTDSIDLPKEKLIDKMVQVSVGPLIGDAIKRIHENKPVSPLFENRFKR

>fig|1664.9.peg.1421

MVNKNAIILAAGKGTRMKSKLYKVLHEVCGRPMVDHVLTEVEKTEPATVVTIVGHGAEKVKDYLGDRSQYALQAEQLGTGHAVLQAEDLLKDQDGITIVVSGDTPLLTAGTFEKLFAYHHDKGAKATILTATAPDPTGYGRIIRNDIGIVEKIVEQKDTNDKEAAVNEINTGVYCFDNKTLFQALHEVTNENAQGEYYLTDVVEILKKKGEIVAAYKMPNFEESMGVNDRVALSQATKVMRQRINTAHMRNGVTLIDPESTYIEVGVKIGNDTIIEPNVVLKGNTTIGSDCFVGAGSTIIDSTIEDNIQITSSTIESAIMHTGSNIGPNSHLRPNAEIGVDVHVGNFCEVKNAKIGDRTKIGHLSYVGDATLGTDINVGCGVVFVNYDGVAKHHANVGSHVFIGSNSNIVAPVEIADHTFIAAGSTITDDVPEKAMAIARARQTNKENYWAKLPVAKDEEWQ

>fig|1664.9.peg.1422

MKKSPSRLWLTLDGLLILLLVVLGINWSTSHHQPAKPTASVTHSQTAHVKQTPKKQTSKKPLDWHAPSEKKPYPDVKKYAHFWIDVSIKKHRVYLMDDEKLLYTMRASTGTSDSPTPKGTFHIQEEHGEAFFNGNPGEGAKYWRSFLDHGVYLFHSVPTDKAGNFLPDEAQKLGETSNSHGCIRLSVADAKWFYQAIPVNTKVVIH

>fig|1664.9.peg.1424

MKVRRSDRLIDMTRYLLERPHTLIPLTFFSNRYESAKSSISEDLSIVKRTFFNRGTGILETVPGAAGGVRYMPMIKQVEAEEFIAEMAERLSENDRLLPGGYVYLSDLLGRPEVLRQVGRLIANQYVGKEVTAVMTVATKGIPIAQSVAMFLNVPFVIVRRDSKITEGSTVSVNYVSGSSARIEKMELSKRSLPENSKVLIVDDFMKGGGTVNGMKSLIEEFDSEMVGISVFAESVFEGNHMVEDYTSILKVDKVDIENKALSVSAGNYLTKNAEQLS

>fig|1664.9.peg.1425

MQLIEKAPAKINLSLDALYQHTDGEFEWQMIMTSIDLADYVQITLQDSPQIEVRTSKGYLPEDKRNLAYQAAQLLRHRFDIKTGAIIEIDKHIPVAAGLGGGSSDAAAVLRGLNQLWHLGLTKAELAHIGLSIDSDVPYCVYSETALVTGKGDQIQPLGDLPNFWMVVVKPEVSVSTPRILHALNCDQITDRPQTDRLLAGIQQQDAQQMTAAMANVLTPITNQRYPQIDYLMQRLTAFGAEKAQMSGSGPTVFGICRQYSRAQRIYNSMSGFCREVYLVQPLKK

>fig|1664.9.peg.1427

MPTTLANIKNGLDGHIGDKLMVVAQAGRKKVTKRKGILRETFPAVFVVDLDQDENAFERVSYSYADLLTKSIAIEFDKEPIVL

>fig|1664.9.peg.1428

MEDIANPERTRKILKRYGFKFKKSLGQNFLTNITILKQIVEAGEITKDDDVIEIGPGIGSLTEQIARKAHQVLSFEIDDRLIPVLKDTLNHYHNVTVLNQDILEADLPTLIAKHFDGQHNLKIVANLPYYITTPIMLHLLEAGLPIDRMVLMMQKEVAERIDAAPGSKAYGSLSIAVQLHSEVKLAFIVPKTAFVPQPNVDSAIVEFVGRQEPLVTVQNQQLFDQLVRGAFAQRRKTLWNNLQNQFGKQEEVKAGLVAALDQADIAPSTRAEQLSIQQFAQLSDCLNEQPVFAKKGDK

>fig|1664.9.peg.1429

MKKTIQEVIVVEGRDDTKRLREVFPDVDTIETRGSAINDEIVGKIALAQEKRGVIVFTDPDFHGEKIRKIISQNVPGVEHAFLPRSEAKPEKMGGSLGIEHAKPAAIKQALENLLTQDEDAVEQISQSDLLVAGLIAGPSAKARRECLGEVLHIGYTNSKQLYKRLKMFQVSQAEFGAALRQINEELGD

>fig|1664.9.peg.1430

MQIFDSHTHLNDTPYAGQEADFIQQAAELGVTEMAIVGSDTVLNDGALRLAQQYKALSAIIGWHPESAKDYTKVQEAQLIEQLQLPEVVALGEIGLDYHWDTSPRDVQRTVFERQIEIAKSLHMPISVHTRDALEDTYKILKETDVRDCGGIIHSFNGDSEWLKRFMDLGMQISFSGVVSFKNAREVHESAKLCPLDVMLVETDAPYLTPTPYRGKQNQPGYTRYVVEAIAKLREVAPEEIAAATYANAHRIFKLGAI

>fig|1664.9.peg.1431

MADKKPTFYVTTPIYYPSGKLTIGNSYTTIAADVIARYKRLMGFDVFFLTGTDEHGLKLEQKAAEKNLAPQAYVDGMAEEIQALWKTLEISNDKFIRTTDDYHVKAVQKIFDRLVEQGDIYLGQYEGWYSVSDEEYFTESQLTEVYKDENGKVIGGKAPSGHEVQRVAEETYKFKMSKYADRLLAYYEEHADFIEPSSRKNEMINNFIKPGLEDLSVSRISFDWGIHVTGNPKHVVYVWLDALSNYITALGYGSDDDQLFNKYWPADVHLVGKEIVRFHAIYWPIFLMALDLPLPKQIFGHGWLLMKDGKMSKSKGNVVYPEMLVERYGLDALRYYLMRAIPFGNDGVFTPEDFVSRINYDLANDLGNLLNRTVAMINKYDDGHVPSYQADVTAFDADLQATATQTIAQYSTLMDQLKFSDALDTIWQLISRANKYIDETEPWVLAKDDSRKAELDSVLAHLAESLRIVALLLQPVMTHAPREMFAQLGLDFDQEAQREMVYGNFPEAVKVIAKGTPIFPRLDLDEEVAYIKEQMMAAQKAGLVNEKVKAKQVAAEEVADFDPKATELVSEKDAIKFEDFDQSEIRVAEIKAVSKVEGADKLLKFRLDAGDQGDRQIISGIAEFYPDFEKLVGKKVLAVTNLKPRKLRGELSQGMLLSAEHGSDVELVVVPSNLVNGSQIG

>fig|1664.9.peg.1432

MITIGLTTWSEHQSLMPEKKQLTLNDYAQFLPIVEVDSFYYALQAPTVSAKWLQQVPHQFQFIVKAHKAMTKQEDYTDFTSTEKELFVRYKQSIQPLLDAGQLTAVLFQFPPFFQLNQENVQYLRRIRTWLPDVPIAVEFRNGTWYDEAYKQNMFAFLKQLKMTHVIADEAQTPTNSVPFEPVVTNPDFAMLRLHGRNMAGWQNPGAMWRKQRTLYRYNADELQQFAATVQRLTKQAKEVAIIFNNNSGGDAADNALALQQILGITFENLAPKGPEQTSLF

>fig|1664.9.peg.1437

MTEPEHRKVILYLAQSLDGFIAEKDGSTAWLSALNSSESDAAIQSFYQGVDTIIMGRKTYEQCIKLAGTYPYQEKQSYVFSKTLHDTADRTNVVTGNVPEFVRQLKAQDGGNIWLVGGAETFTMLLKAQLVDELIITIAPVLLGDGISLISTHLTDMPLELSKTRQLGQFVELTYKINY

>fig|1664.9.peg.1438

MTDIQCVIFDWAGTIIDFGSLDPVLAFQSAFNAAGIQIDTDRIRQDMGIEKHEHIAKLAKMPEVQRAWFAKYKRGITDADQLQLFNYFEQFLLNRLSTETTLTPAVLQVQTYLKAHHIHIATTTGYTKAMLAIAAKQAGLLGYHPELMVSKEDVAAGRPAPDMINHIMTAFNITDPQTVVKVGDTVIDMQEGKNAGVLTVGLIESSSLLGLSQAKLIDLPQKTRLAKFAEITKTLKAAGADYVIHNLSELPAILAKYQTPQKEHA

>fig|1664.9.peg.1439

MALDVNAIVDQLKESSILELNDLVKAIEEEFGVSAAAPVAAAGAAGADAAAEKDSFTVELSEIGQEKVKVIKAVREITGLGLKDAKGLVDNAPSALKEDVSKDEAEEMKAKLEEVGAVVNLK

>fig|1664.9.peg.1440

MSETIIAKKAQIVDTVVEKFNSAVSIVVMDYRGLTVEQVTELRKQLREAGVQMEVVKNTYLRRAADKAGYEGLDDTFTGPTAVAFSNEDVVAPAKIIANFAKSADALEIKGGMIEGKVATLDEINALATLPSRDGLLSMLLSVLQAPVRNVAYAVKAVADSKDEPAA

>fig|1664.9.peg.1441

MNNILVINAGSSSLKWQLFQQTDLSLVASGLMERMNTPEARFTFKFNGQKEQVAIANLTYEDGVIRLLKQLQDNAIIDSPSDIVAVGHRVVAGATTFEKSTLITADNLATLKALDKYAPLHNPVQVDCIETLMHILPTSVPEVAVFDSQFYLDMPDATSFYGLPYEDSQKYQIRKYGEHGISHGYILQETADFLNQPVADLKLITLHLGGGSSITASQNGKPVDTSMGFTPLEGLPMGTRAGSLDAAIVPFLMNELNKSADEIITLLNTESGILGVSGISADMRDIQSSLATNPRAELAYDMFITSAAKFIGSYYVELGGANVITFTAGIGENDAKMRADVCDRLAVLGVKIDPERNEQGHGARQISTDDSAIKVLMIPTNEELAIAKQVKALV

>fig|1664.9.peg.1442

MDKRVDSISETRTNWLEHQFKIKESGSTLKTEIIAGITTFIAMSYILFVNPTILGDAGLDKGAVFVATALTGIVGCLAMALIANYPIAVAPGLGSNAFFAYSVVLGMGIPWQTALAGVFVASLLFMLVTVLKVREVVINAIPANLKSAMAVGIGLFIAFTGLKQGGLVVASAKSFVTITNWSNPHVWLTVFGLILTVALLARKIPGALFIGLAATAIMGVVTGLIAMPTGVVSSIPSLAPTFGVSLIHLKDVFSVQMAVVVLVFFFSVFFDTTGTVIGLAQQAGFIKDNKMPKEVGKALFADSTSMIAGSVFGSTPTACYIESSTGIAVGGRTGFTAVVVAIMFALSLFFSPLLAVVTSEVTAPILIIVGSFMIKSIANIDWSEFETAFPAFMIILCVPLTYNISYGLAFGLISYPITMIAAGKGKKVHPIMYVAALLFLVLLFTMHELPGK

>fig|1664.9.peg.1443

MDKSTLKQLIDQAAGRKPADTIIKNAKIVDVYNARIIEGTLAIADSRFLGIDAAYTADNVINAHGQYVVPGLIDPHIHIESANVSPAVFGSLVTPHGTTTILADPHEIVNVAGMRGLEYMVASAKNTALDIKYTMPSCVPAANPTLETSGAVITADEIKQSYDQGLTYGLAEFMNYPGVVNADDGVLDELLVSLNARKMIDGHSPALHGQGLNAYAAAGVHNDHECTQVDEMLDRISRGMYVYLRYGTVSKNMPTLLKGVTPQNARFCCLCGDDLQSVTLRETGHLDESIRVAIQNGIDPLTAIQMATINTAQCTGLSDRGGIAPGLKADFLLVDDLEHFNVNQTFIDGQKIAANGAYLLPTDDSVAGFDDLLETVHLDNFSADQLKLNLTSDKAHVIGLQSISRTQNLVLPVAHDAEGDFHYKPSEDIVKVAVVERHHLTGNVGVGLLSGFGLQNGAIATSIGHDSHNLVVVGTNDADMVVAIDALKACQGGGVAVQNGQVIATLPFVIGGLMSTEPIDSLIAHQKAFNTICHEQLNVTAQFDPIMKLGAMPLDVIPNLRITDKGLVDVTKFEIIDINA

>fig|1664.9.peg.1444

MAKKGKNYLEAAKQVDATKAYTVEEAIDLVKKVDFAKFDASLEVAYRLNVDPKQADQQIRGAVVLPNGTGKTQRVIVFAQGEQAKQAEAAGADVVGAEDLVEKIQGGWLDFDVAVATPPMMAQVGRLGRVLGPKGLMPNPKTGTVTMDVTKAVNDIKAGQVAYRVDKAGIVHAPIGKVSFDAAKLVENFKAMQDVIIKARPASAKGQYITSLSVSSTFGPGVKVDVASF

>fig|1664.9.peg.1445

MAKKVANIVKLQIPAGKATPAPPVGPALGQAGINIMGFTKDFNARTADQAGLLIPVVITVYEDRSFDFVTKTPPAAVLLKKAAGVQKGSGEPNTKKVATVTKAQVQEIAETKMQDLNAADVEAAMRMVEGTARSMGFTVEG

>fig|1664.9.peg.1446

MNMWQIINQLLAVIVILVVAAACLIGLDYYKLAHVQHHWCPRLTHYETAPTLFIHGYRGNRYSMGHLLMRLQKTGVAKKALVIKVAANGTLTFKGPQELTASNPTIQVLFQNNHADVQDQVRWLSQIMAILKKDYDVTTINLVGHSMGAITVLRYLLKPTAVPVKAVVLLAAPVNDPSIGSDTATVFWSELTQRGPRKKTANYNYLAKRVAHFPTSISILNVAGELLGTSRHDGSVAVDSSFALRSLLRGRIDRYQEMLVRGAGGAHSMLHENRLVDQAISDFLWQD

>fig|1664.9.peg.1447

MVESLEKSWYVLHTYSGYENKVKANLESRAQSMGMENNVFRVVVPEEEEHEIKNGKDKVDMKKTFPGYVLVEMVMSDEAWFVVRNTPGVTGFVGSHGAGSKPAPLLDDEISQILRQLGMSTRHLDVEFKVGESVKIVDGAFSGLVGQITEVDDEKMKLKVNIDMFGRETATELDYDQVDELV

>fig|1664.9.peg.1448

MIKFLKSVVEEMKIVTWPNAKQTRKDTSTVVMTSVLYAIFFGVVDLAILKLLELFIF

>fig|1664.9.peg.1449

MGVKKVALACTVCGSRNYYVAENKNRTERLELNKYCKHCGQYAQHKETR

>fig|1664.9.peg.1450

MEQTKRDKMLIAAVAQNDSQALDVLFKAYLPMVYHTVAPYYIRLFDEDDWLQEARIVCYETCQCYDCQKGKSFGSFYKLRFKNHILNLLRRELAQKRRGDRQAVLIAERPEHDAISEDTMTYYQSQFELRCYLETLSPFELVSFRVLTGQLSIDEACCQLDCTIDQLKRGQSRCQTKLKRYYQENR

>fig|1664.9.peg.1451

MKKQILIADGYNIIGNWPELNKLKQNDHLTDARDSLLQTLSEYRKFREIEIILVFDAMYVPGIKQSYEQYNLEVVFTEEDETADSYIEALAGKLMSPINQVTVVSSDQAEQWTIFSRGALRVSSHDFYREIKRTHREIDHDAKRYHSKVMQRNRPWNDDQLERLAQMRDQLSDEKTD

>fig|1664.9.peg.1452

MRKNNNQSYNQKNNEFNSRRTAKNTAKPTRRPANKKPAAPVEEAAVEEQTDFVIGKHASLETLKTSDAKKINKIFLQDGLKADFVHDVVKLAKEKRLIIQNVPKNKLDLLSDRQNHQGIILAIAPFEYASVDDIFAKAKAQEEEPFILILDNLEDPHNLGSIMRTADAVGVHGIIIPKHRAVGLTSTVAKTSTGAIEHIPVARVTNLTQEIKKLKGRGLWIFGTDMAGTDYRKWDAKGPIGLIIGNEGKGLSPIVKKEVDQTLTIPMVGHVQSLNASVAAGVLMYQAYNSRQPLV

>fig|1664.9.peg.1453

MKNALQLNGIALAYMGDSAYEVYVRAHLLELGLTKPTRLQQVATHYVSAKAQAALIGLMIDDNYLTEEEMNIFKRGRNAKSYTKAKNTSVSTYRTSTGFEAVFGYLYLTEQQERVAELAHWCIQQVDAGRTHEKK

>fig|1664.9.peg.1454

MKARLRKEDSMLTVYNTLTRQKETFKPLEEGRVKMYVCGPTVYNYIHIGNARSAIAFDTIRRYLEYRGYQVDYVSNFTDVDDKMIKAANAENITVPELADRYIAAFKENTKALNIEPATLNPRATDNIEEIVAFIQDLIAKDYAYAVDGDVYYRARKFKAYGHLAGQDLDQLEQGASEHTATEETLRKEDPIDFALWKAEKGNEIAWESPWGKGRPGWHIECSVMSTKYLGDTIDIHGGGQDLEFPHHENEIAQSEAKTGKTFVNYWLHNGFVTVGDDDQKMSKSLGNFVTVHDLIQEVNPQALRFLMSSTQYRRPIRYSQSLLAEAQTNLDRLKTTLDNLAFRQATAEPGEDQIVMDKVAELETAFVTAMDDDFNVQNGLTQLYELAKLSNQYLEQTTVQADTLTSLATRLTRLLAIFGVVFKVDQLLDTEVESLIEERQAARAAKDFAKSDAIRDQLKDQGIILEDTPQGMRWRRA

>fig|1664.9.peg.1455

MMQQKPLTKRRVLGIVFVILGGALGITAFPEFWRVVHLELVTYLNNGITNAVLGAIIFYLLFLVLANPMLRAMRQLEKTLFKQSPSFLLFGSVFSLIGLLLANVVSIPLYRMPIFVLNTVVPILLMIALGYLGFKVGTTRLDEWRKFLQPKKRNADVLERKVDDNFRKYKILDTSVIIDGRIKEIAKTGFIEGTLMVPNFVLHELQLISDSADNLKRARGRRGLDILNELQKDESLSVEMYDGDFEDLTEVDGKLVKLAKLLDGIVVTNDYNLNKVCEFQNVPVFNINQLANTLKPAVLPGEAMTVTVVKAGTERQQGVAYLEDGTMIVVEDGQHYLNKPLDVIVTSALQTAAGRMIFAKPAHQQKGLNEK

>fig|1664.9.peg.1456

MAKVKTQFVCQNCGYSSPRFLGRCPNCGAWNQMVEEREQPAAAAKSNFTISGRATEPEKISTVNIQKEPRVKTELNELNRVLGGGVVPGSLILIGGDPGIGKSTLLLQVSGQLEKVGKILYVSGEESASQIKMRANRLGVNGDQLYLYPETDMGNIRHQIETLKPEYVVIDSIQTMSEPEVTSAVGSVSQVRQVTAELMRIAKTNQITIFVVGHVTKEGAIAGPKILEHMVDTVLYFEGDTHHTYRILRSVKNRFGSTNEIGIFEMREAGLQEVANPSEIFLEERLAGATGSAVVVSMEGTRPILVELQTLITPTLFGNAKRTSSGLDHNRVSLIMAVLEKRASLMLSNQDAYLKATGGVKLDEPAIDLAMAVSIASSYRDKEIPPTDCFVGEIGLTGEIRRVNRIEQRVGEAAKLGFKRIYVPKNNLQGWDPPTDIQVVGVTTIAETLKKVFN

>fig|1664.9.peg.1457

MKKRGFEIVTKYQSANLSLPVRSTAHSAGYDFACAEDFVLPSIWHYNFVRLFRMIRNGKPLVDDDFERASKTLKPFLVPTGIKAYMQDDEYLMIANRSSNPLKKGLVIPNGVGIIDSDYYGNDANEGEIFIQMLNFSPRDVILRKGERIGQGIFMPYLVADDEVAVTTKRTGGFGSSGR

>fig|1664.9.peg.1458

MDKNAMKKMAAERSVDYVEDNMILGLGTGSTVVYMVEALAKRVKAENLHLTCVCTSIRTAEQATSLGIPVKALNEVDHIDLTIDGADEVDDNFQGIKGGGAAHLFEKLVATTSTRNIWIVDEEKVVDTIGRFPLPVEVIPFGSQQVFNKFVKEGLNPEFRTTEDGQRVLTDSKNEVIDLHLEKITHPHLLATWLSEQVGVVEHGLFLDLVNEVVVGSPEGVKILKAR

>fig|1664.9.peg.1459

MTQEITHANLIKMRQALADMPQAQTLRNAVMNNGINAVAQRPDAAVTLDPTYSIDLPTGDVSFQKKSGRCWLFATLNTLRHDFEQQYNVKNFELSQNYLSFWDRIEKANLFYEKILATATKPITDREVAFALAGPDFDGGQWDNAVALIQKYGAVPKSVMPETYNSDLTTEFNSTLNLKLRTDAIKLRQLVADQAPSDKISETRTAMLSEIYRLSVYAFGEPVETFDFAYRDNDQQYHLDQGLTPLSFYQKYLNRNFDDYVTVVSSPQASKQYNQLYSLDSQDTVVEGHPMRLLNLPPDRLKALAIQSLKAGEPIWFGNDVLADLDRQKGWLDSNLYDYSSLFSIDLTMPKDQRLDYRQGVVSHAMTLTGVNLVDDQPTKWKVENTWSDKVGNKGYFSMSDAWFDDYVYEVVIKKEYLTKEEQALLDQTPIKLDPWDALQ

>fig|1664.9.peg.1461

MVQPITAKDIQQFKADFKAVPQSNVIKNTVMNNGFLASSQNTTSKALMDPVFSIDLDTGAVSNQKQSGRCWMFAALNTMRHSLQAQFKIKDFELSQNYTNFWDKFEKSNYFYENVLKTSDQPIGDRKVDFLMTTPQQDGGQWDMLCALIEKYGIVPKSVMPETYNSEKSSELNSVLNLKLRKDAVTLRQLVADGVSEADIQAKKDTMLTEVYRMLVFALGEPPVEFDFEYRDDDHNYHIEKGLTPQTFYQKYVGWDLENYVSLINSPTADKPYNHLYSVEMLGNVVGGREVRHLNLDIDDFKQLAIKQLQSGESVWFGSDVGQSSDRKLGIMDTEIYQKAALLNMDLSISKGERLDYGESLMTHAMVITGVDIVDGQPTKWKVENSWGDKVGTKGYFVMSDSWFDEFVYQIVINKRFLTPEMQRIEKEEYDHPTVLAPWDPMGALASR

>fig|1664.9.peg.1463

MVEETIDIDLLRRLSEADGIGGREREVSRLVHDYAQPYVDEISYDNLGSIILKQNGTANEGPKVMLSAHMDEVGFVVRQITPEGYLKLLPVGGWWGHVMPAQEMTVTTATGQKYIGIVGSRAPHGLPDEVKNKVMTPMTMFLDMGVADRQAIEALGIRIGDMITPYIPFRQMNDPNFLAGKAWDDRFSLGAELEVMKRTAQKPHEATLFFTGSTQEEVGIRGARTAVHQIQPDLAIALDVTTAKDTPLDNESSNRLGGGVVLAVLDSLTMANKGLLYRMERLVADLKLDVRYDFMTVGGTDACNIHKAMDGVVTMTISMPTRYMHSPRLMVHQDDYKQTIQLLTSFCQSLSKADVALFKEATRQALEKTY

>fig|1664.9.peg.1464

MLNQETIDLMQALTDINGVASNERLVAARLKQDYAQLADEVIYDNLGSIYAVKKSQTPNAPHVMVSGHMDEVGLIVTKVLDNGLLQALLLGDMSTNSLLGASVQGDFAGKNYQGTILAQTENNQVVDKTNKVQVDLGFLSKAAVEKAGVQLGDTLSFATSFVQTESGALMSRNWNGRYAPILGVELLQALQNVELPFDLYVGCTVQEQVGLRGIQTATNKVAPDLAIMLDTDAAFDYQTDSDDRIGVLGDGVLLNYYDKTVLPNRLLLQTFRETCEQDQIPYQYYYSLADSEAGWVNKLRTGCPTLFVNIPVRNMNTPRSVIAAADYQAAKAGLIQFIKQIDNDSLQAFKAENR

>fig|1664.9.peg.1465

MEILILLDQIQAGLGGTERGDLPLGGKKMAMGSADMFEKYLEKDEKIAVTLYCGDQNFMDHKDEVSLKLAAMIKKLHPDVVICGPAFHYEQYAEMCAQTGALVTEKTDIPVVAAMSKECQSVIDQYRQRVDIVKMPKKGGTGLSDALKDILALCRIKANHGDISEFPSDKIY

>fig|1664.9.peg.1466

MFDKLEKFLMPLAIKLGSNKVLIAIRDGFLITTPLIIVASIFLLIANFPIPHWSEFWGQFLGTGWESWFTNVSRAVFNTVGFFTCFSTAYAYGKQKNTDAIQAAAVALVAFLILTPTKMVVENLKEPVDGALDMNYLGTNGIFLGLVVALVAVPIYCYVTEKGWTIKMPDGVPPAVSQSFEALIPSAVVMLVFFAIRIIFGLTPYETLYNFIFEILQVPLKGAGNTLTAQIIYGFACTIFWFFGINGPAVANSVFAPISKVLTMENLDAFQAHKALPNIFTDPFSNFFTNFGGGGSTLSLLIVMLLFCRSQRIKQLGKLAILPGIFGINEPIIFGLPIVLNPILIIPFNLVPLINLLLSTMVTKWGLIPYTNGVALPWTTPIGFSGYLSTGSIVASLYQFALLALGCLIYYPFIKTLDKQYIKEEQDAEMAKAEDISFDDLTEADLNL

>fig|1664.9.peg.1468

MTYAMIGTWRMAYEGLSKAMTTLEAGGKSTDAIEIAIKEVEDYPFFKSVGYGGLPNAQGLVEMDAAFMDGNTFAIGAVAGISDVKNPISVARQLSNDHFNSFLVGAGASQYAMFNGFEARNMLTERAHKLWAKRVQEINEKNLSPYDGHDTVGMVSLDQDAGMAAGTSTSGLFMKKPGRTGDSALSGSGFYVDSDIGGATATGLGEDLMKGCLSYEIVRLMGSGLSPQAACDQAVYPFVEKLKKRYGKVGEFSLIALNNQGEWGVATNVEFTFAAGNDEQTPQIYMANPGPDQTTIIEPISEAWLIAYEKRIKAPID

>fig|1664.9.peg.1469

MIKKNKLIAFFTLIGLVTLLSACSSQTKPYDAKQKLGPQINYTITGIDAGAGIMASTQKALSTYHLEGQNWQLQTSSTAAMTSTLDKAIADKRPIVITGWQPHWMFTKYPIKFLKDPKNVFGQAEHIDTIVRKGLKKDMPEAYTILDRFHWTPAEMSKVMLQVNDGVDPEKAAKDWIKANPKQVAKWTKGVKKVHGTKIKLTYVAWDSEIASTNVVAEVLRQQGYEPTIQAMEIQPMWASVATKAADAQVAAWLPKTSGLYYKDYQGKFEDLGPNLEGAKVGLAVPKYMTNVNSIEDLKTK

>fig|1664.9.peg.1470

MLNLMTIPKLPLASWMDSFVDWLTQFTGFFNGLTNFIGGIINGFQWVFDLIPIWLFIVIIAGVTYFANRDTKKWGLILFEVAGLLLIWNLDFWRDMTQTLTLVLTSSLISLVIGVPLGIWMAKSDIAQAIFKPILDFMQTMPAFVYLIPAVAFFGIGMVPGVFASVIFAMPPTVRMTNLGIRQVPTELIEAADSYGSTEWQKLIKVQLPLAKSTLMAGVNQSMMLALSMVVIASMIGAMGLGTRVYFAVGRNDAGGGFAAGLAIVILAIIIDRLTQALNRQRQH

>fig|1664.9.peg.1471

MTVKLQVKDLTKIFGKRVPRAKELLKQGKSKAEILKETGATIGVDRANFEVESGEIFVIMGLSGSGKSTLVRMINRLIEPTEGSVLIDNEDLMQIDKKELLEVRRKKMSMVFQNFGLFPNRTILDNTSYGLEIQGIDKQTRDQKANEALDLVGLNGYGEQYPTQLSGGMQQRVGLARALANDPEILLMDEAFSALDPLNRSDMQDQLLDLQETMHKTIIFISHDLNEALKIGDHIMIMKDGEIVQTGTPEDILTRPADEYVEKFIENVDRSKVYTAGNVMIRPTTVNIEKDGPRLALKRMRENEVSTVYVVNNKRELVGIIDARDVLELVRKESRDLQSVVNANVPTTTEETPIADILDDISQTGIPFAVLNEKKQLRGIIIRGAVLGALAGNEVNHFA

>fig|1664.9.peg.1472

MEKSKKINVNGLILMIFGSIFGFANTTIAFYLMGYASIIWYILAAIFFFLPTSLMFAEYGSSLKEARGGLYSWLEASIGKKAAFIGTFIWLASWIVWMVSTAAKVWIPFSTFLQGSDQTTTWHFIGLNGTQVVGVLGVLWILALTFLSSRGIDKIQKFASFSGMTVAFLFAAFLVISLAIWFKTGFKLAEPIHGVTTFVKSPMASYQSPIGMLSFVVFSIFAYAGMETMGGVTDSMDHPEKTFPKGLMIATAFITLIYAIAIFMWGISTNWHQVLGGDKANMGNITYVLMQNLGYQFGHAFGMSTTAAVGIGTLFARITGLIMFISYLGAFAVLIYSPLKSFVLGTPKALWPKGFAEVNKHGMPEKAMWAQAVLVCLLIAVISFGGKAASEFYNVLTLMSNVSTSLPYLFLVTAFPYFKARKDLERPYEFFKTKASVQAVTIVVDLVLVFGIAFTIVQPMLEGSYMDAFWMVIGPIVFAIMAIALYHRYQKKVA

>fig|1664.9.peg.1473

MFEVTTVHIMIRLTLATVIAGIIGSDREYKNRPAGIRTHILVCLGACVIAMIQQEIAAQNVQFALHHLAIATTIRSDQARLIAQVVSGIGFLGAGTIVVTRHKIQGLTTAASLWATAGIGLAIGMGYYQIAVISAVFVIFVIVFLKKIIHINTLKKIEIKYKHKLETKEFIQTYFEEHHIQIRDVNFSVTQNERDTIYTNVYRIEIPREMQYSEIVEDISMNKNIMTIRMVSI

>fig|1664.9.peg.1474

MDKKQREDSQKHEKFIHYADGSSLEEINNTVAIPKNAGFWKTLMAFMGPGALVAVGYMDPGNWITSIAGGAQFAYTLISVILVSNLIAMLLQAMAARLGIVTGMDLAQMTRAKTGKKMGIFLWIVTELAIMATDIAEIIGSAIALELIFNIPLLWGVLITAFDVLLLLLLMKLGFRKIEAIVATLVAVILFVFLYEVILAQPNMGDVVRGFVPSPRIMTDKKMLFLALGIVGATVMPHNLYLHSSIAQARQYDRDDVAEKRKAIKFTVIDSNIQLTIAFVVNCLLLILGAAMFYGTNSDLGRFVDLFNALQNKEIVGSIASPMLSLLFAVALLASGQNSTITGTLSGQIVMEGFVRMKIPLWARRVITRGLSILPVIIFTVYYHGNEAQVENLLIYSQVFLSIALPVSMIPLTLFTSDEKIMGPFVNRPWVKYTAWFVTIVLTLLNIYLILQTVGLAA

>fig|1664.9.peg.1476

MTKQFKELEDFLGTHFIYTYDNGWKYEWYAKNDHTVDYRIHGGMVAGRWVRDQEANIVKLTDGVFKITWTEPTGTDVALDFMPNENKLHGTIFFPKWVEEHPEITVTYQNDHIALMEESREKYETYPKLLVPEFATITYMGDAGQNNEDVISQAPYEGMIEDIQEGRYFDENYKKINR

>fig|1664.9.peg.1477

MEAAVANKNHTVDIIDKKLRPIKANEALVKMEYCGVCHTDLHVKNGDFGEVPGRVLGHEGVGIVSEIGSAVTSLKVGDRVSVAWFFQGCGHCDYCVNGQETFCRQAQNAGYTVDGGMAEECIVTADYAVKVPEDLDPIAASSITCAGVTTYKAIKVSDIKPGQWLVIFGMGGLGNLALQYAKNVFNAKVIAVDINDDKLAIAKELGADVIINGKQEDVVARVKEVTGDGAHATVITAVSKVASNQAVDAVRATGKVVAVGLPVETMDLSIPRLVLDGIEVIGSLVGTRADLAEAFEFAAEGAVTPIVQTRPLADINDIMTEMAEGKISGRMVIDFTK

>fig|1664.9.peg.1479

MTTQINPYNQSIEDLQKDYPGNDFEQGLSQVEAQKRLATNGPNKLESKKTPKWQLFIRQFNNLIIYILIAAALLTTLMGHYSDAIIIGLVVIINAIIGYYQEANASDALERIKDLLATEATVYRDGQRIDIPTEDLVVGDVVFLEAGDSIPADLRIVDTDNLRLEESALTGEADSVDKQAAALTPTTVPLADQINMAFASTSVTNGSGLGVVVATAEQTEIGQISKAVTHTQSRPTPLTREINKLGAGVSYVIIAAAILLFILGFISGLYSLPVLAIAIVTMVVGSIPEGLPATTSVILAMGVSDMAKNKHAIVKTLPAVETLGSVDVIATDKTGTLTKNEMTVKDIITADQIYHVSGNGYAPEGQIQQADQPAVIDDTLALFLEAGFEANDTTLFKSDEGWAINGEPTDGAFLTVYHKGFSIDQVPQNTEIDMLPFDSDYRYIAKLVQTTTGQRRLYIKGSPDKLFPMAQQKDAAFPLDTWNQHVQRLSEQGKRVVAVGYLDVPASTTTITHEGLNKGITLLGLAGIIDPPRDEVIQALAEMNAAGVDVKMITGDHPLTAKAIGEKLGLAPVIKTITGPEWDQLSPEDKEIAAVEHQVFARTTPSNKLEIVEALQKQHKVTAMTGDGVNDAPALKRADIGVAMGIKGTDVAKDSADMILTNDNFATMSVAIQEGRRIYDNIKKSILFLLPTSFAEGLIIAFTILMQQSMPLQAAQLLWINMVSAITIQFAFIFEPAEDGIMARAPRKTGQTLMNKHDIFQMAYVSVLMALIALWAYDWLLGRGVDQVTASTMMVNIIVISKIFYLFNIRTTKLAFSKAFFSNPKAFGIIAGMIVLQLILTYVPFMQKLFYTEPLTLLEWAVSIGAGFLILIITECDKIIRLKLKR

>fig|1664.9.peg.1480

MDIYLKQAILQVVDRELGAPIYSQQPLDLTNGNTRDYLTNKIKKLSSAQSKTGVIRQGSEVANQLLNIASDFTGVSQQLVDKWYQAYQKSDDAPSADVFIALYEQDTQAYVAFLKVNYHEAYTHYVNAEEGPLTNQLIIHQSILSNKTQKADEGFAINLQTLGFELIEKQYTFSGDKRPYLSTEVIESEPQPSLEENVRVVKKAAEKIGAKFEVAKHDIVANVKEAIYETIEAKGEINTQDVAAKVFKDNVSAQTEFKAAMVEKAPADAPISPAVREIAEKKYGKQKLKLSNGIELIVPIDVYQNPDLFEFINHPDGTMSVEIKNVDEVISRL

>fig|1664.9.peg.1484

MANTNKRLAVIFGDVTLGLKGPDFHYLFSYQTGGPESLRIQGKEWLYRSPKPTFWRSTTDNDRGNQFPLKSGMWLAADQFIACQSITVAIDGQTIPLPIAPENNRYSGQETAQEVTATYTYQTITTPQTTVEVSYTIQASGKIRVAVTYHGQAGLPSLPVFGLRFVMPTPATRFIYQGLSGETYPDRMAGGMAGEYEVTGLPVTPYLVPQDCGVHMATDWVTIYRQAVLDNRLREPVETGLKFKAVDQPFAFSCLPYTAEELENATHHSELPAPHRTVLSLLGAVRGVGGIDSWGSDVEAAYQIDATQDHHLEFEISF

>fig|1664.9.peg.1485

MQPNIQWLDTPAVFRVGQLPAHSDHRYYATLAEMAQQQSSFEQSLNGTWQFHYSVNAASRPKSFYELAFDAQDFEPITVPQHIELAGYEQLHYINTMYPWEGHYYRRPAFSTSDDKQHLGMFSEADYNPVGSYLHHFDLTPALRNQRVIIRFEGVEQAMYVWLNGQFIGYAEDSFTPSEFDLTPYLKETDNCLAVEVHKRSSAAFIEDQDFFRFFGIFRDVKLLAKPRTHLEDLWVIPEYDVVQQTGQVKLRLQFSGDENRVHLRIRDQHQIILTADLTSAAQVNGLYKMPELVQAWSNQTPNLYTFELEVVDQAGETIEISQQPSGFRKIEIKDKVMLLNGKRLVINGVNRHEWHPETGRTITAEDEAWDIACMQRNHINAVRTSHYPDRLSFYNGCDQAGIYMMAETNLESHGSWQKMGAVEPSWNVPGSYDEWEAATLDRARTNFETFKNHVSILFWSLGNESYAGSVLEKMNAYYKQQDPTRLVHYEGVFRAPEYKATISDVESRMYATPAEIKAYLDNAPQKPFILCEYMHDMGNSLGGMQSYIDLLSQYDMYQGGFIWDFIDQALLVTDPVTGQRELRYGGDFDDRPSDYEFSGDGLVFATRDEKPAMQEVRYYYGEHK

>fig|1664.9.peg.1488

MVKVLIVHTNITRYQGTTDPTGLWLGESAEFVAEMQKAGIDYDFISPKGGFVPLDPRGMKYTDEAILEIYEQPDYVQRGLVATLKPSDVNPADYAAIYYTGGHGVMWDFPDNAEIQAIARAIYQQGGYLSSVCHGIAGLLQLKDTKGQFVIAGKKVTGFTTAEEVLAGKKNVVPFLNEEVAAQNGAEFTKKRAYKSFAVQDGQLVTGQNPFSVQAVTDLLIPAIQK

>fig|1664.9.peg.1495

MAQVTYNGTGRRKNSVARVRLVPGTGKITINNKDVVDYVPFANLILDMKQPLTITETTDSYDILVNVNGGGFSGQAGAIRHGISRALLTVDPDFRPALKSAGMLTRDPRMKERKKPGLKKARKASQFSKR

>fig|1664.9.peg.1496

MRTTYMAKPGEVERKWYVIDATDIALGRLSTVVASILRGKNKPTFTPNVDTGDNIIVINAEKIKLTGRKATDKLYHHHSNHPGGLKTRTAGEIRENNPERLIEMSVKGMLPKNSLGHNQFLKLHVYAGGEHKHQAQNPEVLDITDII

>fig|1664.9.peg.1497

MTQRYRVTVAYDGTDFAGFQVQPKQRTVQGTLEKALTKMSKGAYIQVYGSGRTDSGVHAMGQVVHFDYPSELPAKSMLRALNSLLPLDMEVVDSQLADDDFHARFSTVGKRYMYRVDLGHYTNPFKRRYTGHYPYPIDVERIKAALPDVMGTHDYTSFAAAGGVIKDKVRTIYEATVVYNEAENELIFEFHGNGFLYNMVRILVATLLEIGNGRRDVHDFLRLYEVKDRQQARSTAPASGLYLKEVYYK

>fig|1664.9.peg.1506

MKAAVVTDFKQDPKFDNQFPTPILNPDEVLINVIASSLSNRARSGAAGSHYTSTDQLPMIPGVDGIGTLPTGEQVYFASEGTFAEQVAVKKGHWVTVPDGLDAIKLAGMMNPALSSWMALNYRANFSAGQKVMILGATGNAGMMAVQIAKRLGASEIIAVARNTEKLKTLTDLGATQLVDLSAEPAQRNAQLAKAGSDVDIVLDYLWGDVAANAMTAIIPHRQNAEQLLQWVEIGSSAGQTAPIPGAGFRAVALSLIGSGQGSVAPIDMMKSLNAILTAEKEQPFTFTTRTLPLSDVEAGWHLPGNERLVFTI

>fig|1664.9.peg.1509

MNKKNTLLVTLALLLSNMMAGLDGTIINTALPAIISDLHGIQYMGWIVAVFLLGMAVATPLWSKLGEHIGNRRAYQLATLLFAVSSIFQAVSGNIVFFLIARTVMGIGAGGMNTLPFIIYADLYQDLRKRAQVIGYATASYSAASIVGPLIGGWIVDTFSWHWVFYINVPIALVSILCVRYFFKEQPKAHLSGSVDYLGAGIMITSLVTLLTGIQMIGTGSLTLIIGLIVAGLALLVVLYRVEDRAADPIVPNRLFKNPQLVTDFILFVVLWGAFIAFNIYIPMWAQGLLGLSALLGGMTQIPGAVTNLIGSLAGPAVQPRLGKHRVVTLGTVAFLIAFSGMLMMGDQAPFWFLLLMGAFEGFGLGMSFNVLQISVQEDAETRDVPIATSFAYLLRILSQTFMSSIYGVILNHALTQGIAKSHVKITMAMMNKLSNLQRVHELPAHLLPAMRAIMYQGLHNIMLIATVLLVIVLAFNLWLQGKAKKENVI

>fig|1664.9.peg.1510

MKTHFDTIVIGGGPGGLAAAYRLAEQQSVLVVENDLWGGTCPNRGCDPKKMLYSAVEAIDHQHALQASGLVGTSYINWPQLMAFKRQYTTQIPDGTLNGLQSAGIRTVTGTAHFIADHHLRVGETDYTADHFIIATGQTPTLPAIEGRDLLQTSNQFLDLDHLPAKIAFIGAGYIAIELANIAATAGAEVHIIQHNKRILRDFPEAMTTELITSLQNKGVQFHFETTVTKVYETTKSLVLNNAGGFTLTVDAAFAALGRHANIDELNLPAADIATGHHGIQVDEHLVSSNPRIYAIGDVVDRPQPKLTPVAGFEGRYVADQLLQTNSDPITYPLIPHTVYASPQISQVGVSVQTAQENPDQYRLNQQTTTNWYTFNRIKEPNAIVTTIFDRQTNQLVGAAAYTTIAEELINYLTPLIKNHTTAAELTDTIYNYPSPASDLKYYY

>fig|1664.9.peg.1511

MIEFEGVTKQYGQSRALDGLTLTIPDNGLFVLVGPSGSGKTTSVKMINRLIEPTSGTIKIDGQPIDQMDLQTLRLNIGYVLQNIALFPHLTIEENITIQLEVLKWSKADRQKRARKLLTLVNLDPDKYANRYPSELSGGQQQRIGIIRALATKPKVILMDEPFSALDPVTRTQLQDLVLDLQKQLHNTVVFVTHDMREALRLGDEIAVMRAGSIQQIGTAEAIFNQPKNDFVREFFDIERQPTTLTVQDLVAADLGTVVSGQPETIATTASIKALAQALIAQPEGVVVALDDQTQRQLTTTDLTTLIAKRGL

>fig|1664.9.peg.1512

MMIQQIQQILTTQSGELLQALGQHLEISLLALLIAAVIGIPLAIILTAHQKLAEAMLQVTSVLQTIPSLALLGLLIPLVGIGTVPAVITLVLYALMPIFQNTYAGLSGIDPALKEAEIAFGLPRAKRLLRIELPLAMPLIISGLRIALVMIIGTATLAALIGAGGLGTFILVGIQSNNNALLVVGATLSAVLALVVSALIRWLGTLSFKKITISLAIFVGLFGLVEGVTRFQAAPTKITIAGKLGSEPEILMNMYRDLILADHPQYDVTVKPNFGGTTFLFNALKADQIDIYPEFTGTVLQTLVKSPTKTNQNPTKTYQLAKKSLQEQFKMAYLPPMRYQNGYDLAVSAAFAKKYNVKTISDLAALNQSFTAGFDPDFYHQKDGYQGLRKTYQLKLDAKIMEPSIRYKAIADGKVDVVDGYTTDAEVARYNLVVLKDDRHFFPPYQGAPIMKESFKKAHPEVVKALNKLAGKITEDQMQQMNYQVQVKHRKARDVAHDFLVEQKIINK

>fig|1664.9.peg.1514

MTVTNDAAFTAMTNGEWYFDSEATVQQRNETRLALQKAGQILDNATRMATIQKLLGHTGSDFFIETGFEFSFGQNISVGDHFYGNRNVLICDEGRVTIGDSCKFGPGVSLLTPYHPLDPTERATKKEISGAITIGNHVWLGANVTVLPGVSLGNNVVVGAGSVVTKSYPDNVILVGNPARVLREIPAK

>fig|1664.9.peg.1517

MLFQNIAGIDWLVWIGVVAALMLLNEAARANKWVALLLFVGLPIILTIFVWPTTAGPDSSTGTWFHWVKVYSALAGCLGFMALRFSPKLQHNKWALIFPPAILAFNIMEAVIRDFQVAGLHGLVDGVVMNGGAWNIMNGVAGIINIITISGWFGIIISHDKQKDMIWPDQIWPWIIAYDVWNFAYVYNCVGDHSFYAGAALLISCTLAAFFVKKGSWLQARAQTLAFWMMFTMSYPAFVTDSQFSVQSTHSSAALMTVSALALAINVAVLVLHVYRIIKHRRNVLTDDIYAGTVAHNQVLADNTPADQQPAGLNLKK

>fig|1664.9.peg.1518

MQNKFKKVKEALKEAIISGQYSIGAKLPTETELMAQYDVSRYTIRRAVGDLEEEHFINRIQGGGMFVQDWHREWTNDADSKVIGIITTHIADYIFPSIISGIDRVLSEKGYSILISNTHNDHEKERKSLINMLDNKVAGLIIEPTQSALKNPNMDLYHEIQDDQIPTLFINASYPELDFPVITNDDLEAEKEMVNYLFELGHEAILGIFQVGDLQGVHRMNGFIQAYQQQPAIAYKSNLIMYQSGDSFEKVLKRVADYLQGSEVPTAIVCYNDQLAIRVIDFLKSKGLKVPADISVVGFDDYQMSRYISPSLTTLNHNKTKMGEDAGQMVLDMIQDKPVESILYQPPLIKRDSAGKVK

>fig|1664.9.peg.1519

MTQAKKIPSSFIYFFGSFGGILFGYDIGVMTGALPFLQHDWHLENNAGVVGWITSAVMFGAIFGGALAGQLSDKWGRRKMILLSSLIFAIGSLLSAFSPNDGQVYLIAVRVFLGLAVGAASALVPAYMSEMAPARLRGRLTGINQTMIVSGMLLSYIVDYLLKGLPEQLAWRLMLGLAAVPAIILYFGVLRLPESPRFLVKHNRLDEARQVLGYIRSKDEVETELTNIQTTAGMESNVQQSTSMKTLLSGKYRYLVIAGIGVAGFQQFQGANAIFYYIPLIVEKATGKAASSALMWPIIQGVILVLGSLLFLVIADKFKRRTLLTLGGTVMGLSFILPAIMNALIPNFNPMMIVVFLCLYVAFYAFTWAPLTWVLVGEIFPLAIRGKASGLASSFNWIGSFLVGLLFPMMIASMPQEEVFAIFGVICLLGVLFIRTCVPETMGHTLEEIEAQGTNKAHKA

>fig|1664.9.peg.1520

MMMNLIEAAQAIKSGQVSLGIELGSTRIKAVLVAQDFQTIASGSYVWENQLEDGVWTYPIDAVWTGIQASYMQMAAEVQSKYHEPITTIKSIGVSAMMHGYLAFSKDDQLLVPFRTWRNNITEQAADELTEQFNFNIPQRWSIAHLYQAILNDEAHVSQVAFMTTLAGYVHWQLSGEKVLGIGDASGVFPIDPKTGSYDARLVTQFDQIKRVQQQPWQLADILPTVLPAGHTAGQLTPAGAQLLDPSGNLMAGSLMAPPEGDAGTGMVGTNSVRKRTGNISVGTSAFSMVVLDQPLKQVHRDIDMVMTPDGSPVAMVHTNNCSSDINAWANLFNEFAARLGVSLAPDRLYETLFLEATHADQDAGGLINYSYLSGENVTKMPAGRPLFVRQPHSHFNLPNFIQTQLYAAFAPLKIGMDILVKEEQIKTDVMIAQGGLFKTPVVGQQVLANALNTPITVMSNAGEGGPWGMAVLAMFAADNNGQTLDDFLDHNVFTNPESMTLSPEAAGVAGYQTFIEAYQAGLPIESAAIAAIKDEK

>fig|1664.9.peg.1521

MLEKLKQEVYDANMRLPKLDLVTFTWGNVSGIDREKGLFVIKPSGVDYEDLKPSDMIVVNLKGEVVEGNMNPSSDTPTHTVLYNEFPEIGGIVHTHSPWAVSFAEAGIPVPALGTTHADTFYGDVPVSEALTQAEIENDYEENTGKVIVKTFKEQGVDPEAVPAVLVRQHGPFTWGATPDKAVYNAKVLEVVAEMDYHALQLTRNNSAVPQFLLDKHYYRKHGKNAYYGQNNAQSQGHAKKEVR

>fig|1664.9.peg.1522

MLNTENYEFWFVTGSQSLYGEETLRSVEKDAKEIVEKLNASHQLPYPIVFKLVATTADNITKVMKEANYNDHVAGVITWMHTFSPAKNWIRGTKLLQKPLLHLATQFLNKIPYDTIDFDYMNLNQSAHGDREYAFINARLRKNNKIISGYWGDEDVQKAMAKWMDVAVAYNESFKIKVVTFADKMRNVAVTDGDKVEAQIKFGWTVDYWGVGDLVAEVNAVSEADIDAKYADLQKEYDFVEGQNTPEKFEHNVKYQIRDYFGLKKFMDDRGYTAFTTNFEDLVGLEQLPGLAAQLLMAEGYGFAGEGDWKTAALDRLLKIMAHNEKTVFMEDYTLDLRQGHEAILGSHMLEVDPSIASDKPRVEVHPLDIGDKDDPARLVFTGMQGDAVDVTMADYGDEFKLMSYDVRGNKPEADTPHLPVAKQLWTPKQGLREGAVGWLTVGGGHHTVLSFAVDSEQLQDLSHLFDLTYVNIK

>fig|1664.9.peg.1524

MMMLVIVVLIVLLGAFTRTVFGFGEALVTMPLLALVAFDLKSATALIGALGLIVAIPAVIQHRRQIDIAIVRRLVIGSLLGVPVGIALMTYVPTVIVLRTLAGFLVIYGSYNLYQLHQHRIQKQHLKAPYWDYLAGVISGVMGSAYNSHGVPVVIYGTLKKWPVDELRGILQAHFVCVGTVVVISHLLAGFWTLQVVQLLGISLPLLLIVIPLGNWVSARIDSQTMVRYIYDLLIIFGGLLLYKTF

>fig|1664.9.peg.1525

MLIFELPIHPENQAAMARYMQDQFTFNGVKATERHAYERDFWPAVKQLPSAELMVIIKALYAREAREYQYVAIDIAARAVRKWQRADLLQFAELVTEKAWWDSVDPWKRVFSEYIKRHPAEFEWVGQLFAQKENFWLRRVGLILQLGFRDATCIDYLQAAIETDQTTPEFFIQKAIGWALRDYAKTNPEWVIDFVDTHTLSALAQREALKRL

>fig|1664.9.peg.1527

MKQALLVIDVQNDYFPGGKMALCQTEAALVAVKRLEADFQAKHLPIIYIQHIKCQKDADFFEVGTVGAQLHPDLYVTDESIIIEKHFPNSFQETRLATALQELAIDQLVICGMMTQMCIDSTTRASFERGYQPILIQDATATKALTINGESVSASHVQTAFIAALTNFASVQNSIEFIH

>fig|1664.9.peg.1530

MQLTSTGIHNGYFDDIYGGYSDALNEIGMPTYSIPFIIEQAPADTVTYAAIFYDLDAYEVTKGFPWIHWTLANLKATTVLANSSQQSPAFVQGVNTWHSPMTDNQAAEASACYGGMTPPNRDHTYTLTVFALDCSLDLEDGFYLNQLTTAMKGHILDKAVLEAKYRQFKH

>fig|1664.9.peg.1533

MQAQAVLKEKFGYDYFREGQADVIESLLAGTNVLAIMPTGGGKSLCYQIPALMLPGLTLVVSPLISLMKDQVDALNENGIPATFINSTLTQGEVQERFNQAARGEVKLLYVSPERLDSDYFLADLAELTIDLIAVDEAHCISQWGHDFRPSYLRLTDTIKSMRQQPTIVALTATATSQVADDIMLRLGIQHEVKTGFSRENLAFQVVKNQNSDRYLIDYLKVNKQKSGIIYASTRKEVERLTKLIEKAKLAVTMYHGGLNEMVRRQNQEDFLYDRKPIMVATNAFGMGIDKSNVRFVVHAQIPGSLEAYYQEAGRAGRDGLPSEAILLFKVNDVQIQHFFIDQSEMDDENKQREYAKLQEMTQYANTQQCLQQYIVNYFDDDCEKCGRCSNCLDTRESQDITIDAQKVLSCIKRMDERFGKVMVAQVLTGSKNQKIMQFRFDELPTYGLMRGDSQKEVSGLIDYLVASGYLRASGGQYPVLQITLAGVKVLKGQEKVTRKMAAKVQKTLPEDNELFERLRELRRDLAEEQGVPPFVIFSDKTLYSMCEIMPTSLTEMLDVKGVGENKLEKYGELFLDILVAE

>fig|1664.9.peg.1534

MRRYAAEFIGTFMLVFLGTGAVVIAKADTLTIGLAFGLTVTVMAYAFGGVSGGHFNPAVSIAMMINKRLEAKDGVFYIVAQFLGAIVASGLLSVLINALDLSRTGFGQTDFPKIGAGVAFLVEVIVTFSFILVILMTTSDRFGNSQMAPLAIGITLSLLIIVALNLTGGSLNPARSFGPAIFAGGSALAHYWVYLAAPIVGAILAAFTGRLLGSEER

>fig|1664.9.peg.1535

MTTIYYDYLTHDNQNLVIAATDHGVCFVGSPNQSLSELTTFMPNHDLLQDPSKLAPAIAQITAYLTHERTAWHLPLDFLVGTPFQQQVWTALQAIPYGQTVTYHQLATQIGRPTAIRAVASAVGRNPLLMIVPCHRVIRKDGSLGGYRGGLPFKKQLLALEQ

>fig|1664.9.peg.1537

MNFIKRAWQNISFKKGRSLLLVIVMTVILVFIMAGLLIRNAAVTTVNNTKQQVGASVTLSANRDQAFKKMRSSTPPTSTSKTKKPSLSMPSVSLANVKKIAALSGVANYNVSVATSANASSIDAISTSSASNNGPMGMSQSTSSGDLQITGVLNMKTLSDFKDNTNKIAKGRALTSADVNTNNVVIESELAKQNDLSVGDTIKIKATTTGKKAYTLKVVGIYKASQSSSSSMGPQQSDPSNTLYTAYTLANQIKGQTNKVDSAVFTLSNPTQKTAFLKAAKKIINTKKFSLTADDSTYQALKQSMQKMESFANKIVWLVAIAGTVILALIIILMVRERRYEMGVLLSLGEKRTKIIGQLLVEMFMLLIVSLALAGVGGQFAGQALSKQVMSSVTTSTTTDSATTQPGGNDGPGGGQAPSGQPGGNRPGGQMTQGTQNKAKSIKASDLNLKINPLTLLQLGAFGFTIIAFAVLLASANILRLEPRKILIG

>fig|1664.9.peg.1538

MKRKQIYTGLWASLLLLAPAVTSATSAHATDIQTGQQSVATSQLSASQSTSSQVASNQLVETSRQSQGVASRSQVTEVPAKTAQTTNEQPAKSAAKAVLNRDYQGALKRGAQQLVASQDTEVWSALALISSGAQLTTVQKATFHQVFAEELTGMAGHYSATDLERLTIGIAAIGEDPTQFNGVNLIAEIIKKAATAGITGQVYGIIALSTQDYGSQANQTINQLIVLVLKQQNTAGGWAFFGSGSDLDITGMTMSALGMHRDQPKVQSALDRAVALLKTTGFVKTTGGFLIPGGFSTEENSNSTAMAILGLAAAGVNPATTFVGDKGATPITNLIAYQKADGQFRWMMGSDDGALSMSTEQAVYALGQYDYLLKGKGSIYNFKKVVPEPDPKPKPDPKPAPAPKPSIPGKKPAGESSASSSSEVDLDKQLPKQPINEADEVPLTGTTATQDALPQTGHQSVWGSVLAGLFILLGVASYQTRRLKQR

>fig|1664.9.peg.1539

MVKKRLLLSVASLVLLGGIGYGTYASVQQQPTTVKHASEQSTQSKKVAHKAVIKHAESTSKKTPTKTKAKETKHSVASKQAQSKEANQVSQFEQASVAKKEQQASASAKQTPTSQSATTATPITRQSASSAVVSTAQQSTQHAESHEAPVKQPEPAAQKVTLSVYGPISEGNAALINHASVTIDDGETVLAVLKRLTGQQGMPLSYRGIGASAYVKGINGLFEFDKGPQSGWLYRVNGVFSNQSCGVYHVKSGDVIDWLYTEDLGHDRHAPQG

>fig|1664.9.peg.1540

MQLIIDQLHPLVLLVYFILMISFSMCFNHPYFLCGQLIVIIAVNWVAKNQQKTVSTLQGALLMMVLIVIMNPIINNHGTHVMMTVGGTLITAEAVVYGFLMATALAVLLLIFVTYNQHMTNHKFLALFGGIAPQLTLLTMMTMRFFPLFIRRLRDITAVQRTRGIQMVTGRLKNRAQAGMHLLAILLTNTLTEALQTADSMTARGFGVQKRSTYRRYQMTHRDYGLLMALISGGLFAFWCASQGNGQLIVYPTLGTLRLTSRDWVCLSVILLIDGLPLLLEGWEYLWWQLHR

>fig|1664.9.peg.1541

MVAIAQIEQLQFQYALANQPSLAEINLTVNAGDWVVVAGPSGSGKTTLLRQFKRELWPVGQRLGQVSFNGRLLADLDPAVSAQKIGFVFQNPENQLVMDTVIQELAFSLENSGEASANIQKRIAELVSFLGIQDILYASVHELSGGQKQLVNLAAILILQPSLLVLDEPTAQLDPIATKEFMSLLQRVHDELGITIIMSEHQLDDVLPLANQLWLMTNGCISYQGPVDQGLQAIWQQPTLRDFMPEIPRLFWQQQLTTETESVPLTVVAGQRQLPVGSIHDQLRPVVAGMQETLLTADHLDFQYIKNEPYVLNQLKLTVHTGDWLAIVGKNGIGKSTLLKVLIGLLEPRRGTVRLLERPLQKWPTEQLFKVIGYLSQQPADQFSAESVRVEFERRAVQLGREHPEEAATAMLAKLGLTALADSDPQDTSGGEQQLIALGIILLANPKLLILDEPTKGLDPLRKQALGHLLQQLQVEGLTIIMASHDMVFSARFANQCALLFDGAIVAQEAPHQFFQSNFFYTTPINRLLRPQDSAALTWEEVDR

>fig|1664.9.peg.1542

MKRIDWQSSLIFGIAFLIIAVLLGLIIQLQGQYFLALSFICLVVSMLPFYWRFEKRQIRAREIVMIAVLATIAAVSRVPFAPLPSVQPTSFVVIVAALVLGPEAGFMIGSTAALVSNFFLGQGPWTPWQMFCWGMMGVTAGLISRFDWAHNKYWLCLFGFIWGFIFGWVMNLWYFIAYVNPLSLKTFIAAYIASFYFDLAHALSNVFFIYCFYQSWYRIIQRFKIKYGLLD

>fig|1664.9.peg.1543

MASLISNTQITLDSHQNWQTVTTQIPTPSDHELLIKVQAAAVNPIDLKRRAVTTHILGYDGYGEVLAIGKTVTQFKVGDIVYYAGSTQLDGSFQAYQCMTEALCALAPDQLSASEAAGLPLVSLTAYELLFEKFHFEAAEQAHLGKNLLIINGAGGVGSIMSQLAKWAGFTVYATSSPQNFNWLKANGVDYPVDYHQTSDQLNLDQLPADHFDAIAVLYDVAPYLSAATRLIKPLGHVGSLVNMQAPLDLTPLKAKSVTFDWEYMFTKTDFDYEIASQGAILKKIARLADTNQIHSITTQVYQGLSVENLDTITTELTKGHAIGKYVLTY

>fig|1664.9.peg.1547

MSDTMQAVAINQYGDEQTLEMITLPMPTIGEHQVLIKSKATAINPLDWKLRAGYLTSMFDWAFPIVLGWDLAGVVTEVGSAVTDWQVGDEVFARPATSNRGTYAEYVAVDDELVARKPKNVTFEEAAAVPLAGETAWQALFNHGHLKAGETVLVQAGSGGVGSYAIQFAKAMGAHVITTTSAKNFDLVRDLGADEIIDYRNEKVADKAHNVDLVIDTIGGQSQVDAWSVLNPETGRQISIVGEAPETADIIKDTKMTFKAIWLIPNGEQLREIADLMADGKVKSIIDTVLPFSAEGLIKSHQLSATHHSTGKIVIKF

>fig|1664.9.peg.1548

MAIIAKDAQDVFKDAGENIIFSTLNLNRQDFSSDRAAVSDLAERIPAIINSMNIRYPDAHLRVAFGIGSDAWDYLFPNAPKPQELEPFQMIPSPKHDAVATGGDLFFHIRAKEMAVCYEVMAQFMQFIGQNATTIDETHGFRYFEGRSIIGFIDGTENPAIDETAEYALVGDEDPEFINGSYAFAQKYIHSMDTWNHTSTEEQEKTIGRKKFSDLELDDAEKPTNAHNVVSQDNEGGVEHKIVRMNVPYANPGEKMTGTYFIGYARQWTIVKRMLTNMFVGKPAGNYDHLLDFSEPTTGALFFIPSKTLLAKIAEEEI

>fig|1664.9.peg.1549

MVTQKINAADAMLKVMADWGIDHIFGLPGGSFDSTMNALHNQKAIMRYIQVRHEEVGALAASGEAKATGKIAATFGSAGPGAVHLLNGLYDAKYDHVPVLALVGQVATGVMNTDYFQEMNENPMFADVAVYNRTVMTAEQLPLVVDQAIQQAYKNSGVAVVTIPTDLGWQPIEDHFEATANLFQMGNYPEPRSTDIQQTLQLIKEAKQPIIYFGQGAKAAGDELVALSDKLSIPMMSSALSKGIVADENPAYMVSAGRVATKPGVDLAEAADLILFVGSNYEFGQYFFKPDAKFIQIDIDPTKLGHRHHVDVGILADAKTALAALLEASEPVSEKTAFYRAAVANKANWDQWTASFEDDPQTPLRVEPVFKEINQMATNDAIFNLDVGNVTIDGVRFLKMKPGQKTTISAWYATMGAALPAAIGTQAAFPNRQVWSISGDGGFTMVMQDLITQVKYQMPIINVVLSNDSFGFIEAEQDDKGQPHSGVAIQGADYGAAATALGAQGFTVRTLDELKAAFAAAQKRQGPVVIDVKIANERPLPVEQLVIDDQTQDPKAVAQFVEKYRAQGLKPFRTFLG

>fig|1664.9.peg.1550

MALIGSIALAIYWSRLGLFNNTTALQAYLGHSVLIGPIIFITIQIIQVVIPVIPGGISTAAGVLVFGPLWGFIYNYVGIAIGSIFNFLLARQFGKPFILHVINEKTYDKYIGTITEKKNFARFFALAIFLPVAPDDVLCLMAGLTEMSFKKFTWIIILCKPASIAAYSFALVYGGQFLMNLF

>fig|1664.9.peg.1551

MSKKSISLFEATLVLVILLAIMGTGVIIFQLSPQTPVLTGIALLMLWARWRHFSWDTIHEGIEEGLKSGLIPIIIFILVGALISTWIAAGVIPSLMVWGFQMVNVHWFIPSIFVVCALVGSAIGSAFTVISTVGIAFFGIGATLGLDTTIVAAAIISGAIFGDKSSPLSQSTNLTAAVTETDLFAHIKNLMWTTVPAFVGSLVLYAIIGHTSRDIPLTKISTTVAILKAHTDVSLRALLPIILIFVCARFKMPAIPTLFLNIAVSIGLIFSHHPHTSFKTIANLIENGFVAHTGNQSIDALLTRGGINSMLSTVALIIMTLTLGGLLMKLGLIQRVMAPLATRLKTPGQLISAVIASGIGVNLFIGEEFLSVILPGKAFKSTFEKSDLSNLALGRALEDGGTVINYLVPWGVAGVFAANTLGVPTLSYLPFCFFSILSPIFSILSSFTGIGLKKKSVTN

>fig|1664.9.peg.1554

MASENDQAFARKLAQIEADSKLTILSSMNQTSDDGVKQYVVYTDYREGGQRESTNSIFVYTPYANVDKFGHSIVDFQAQLLFETNKWKNEMHITVLETLGAASRLAVYDYQNLGLAQLAVKAFCNLANKLEIETIDGNISTFDRESFNKVEHILTKYGFEVKAAPGEASGTFVKRR

>fig|1664.9.peg.1555

MQLTRAFEQATCIIVLLATQDPEHTISSEVIHHRLGGSQTYLRKIIRKLVVAGLVKSVSGNSGGFTLARPASEVSIYDVVLATEGQIKSYPDTGKLDTVFNDFQPVAKQGTLIINAAFHKADMLWANELKKQTVSGLIIETMGHPDIPVIDWNQSAEQRELLIQKIIKNLK

>fig|1664.9.peg.1561

MAENEMKRELKTRHLSMIALGGSIGTGLFVTSGSAIAKAGPGGALLAYCGIGIMVFFLMTSLGEMATYLPVSGSFSTYATRFVDPALGFALGWNYWFNWAITLAVDVSTAALVMQFWLPDMPGWIFSLVALLLIFLINITSVKSFGETEYWFALIKVITVIIFLGVGVLTIFGIMGGHRTGLSNFTIKKAPFVGGLPMMLSVFVVAGFSFQGTELIGITAGESKTPEKSIPKAIKQVFWRILLFYILSIFVIACLIPYTSPSLLGASETNIAVSPFTLVFKRAGLASAASIMNAVILTSVLSAANSGMYASSRMLWSMAEDGYAAKIFAKTNKRGIPVMALIGTTIIGALTFMTSVFGTRIYLFLVAASGLTGFIAWLGIAISHYRFRRAFKVQGHQLSELKYHARWFPFGPIFAFLLCVIVIIGQDIQAFAHFDWTAIGISYMSIPLFIILYSYYKIGYKTKVIPLEKVDLSKHR

>fig|1664.9.peg.1565

MDTTSTDVGVAVVDIDGAANDGLFQKAEGISINLLNYTAGKQKIPYIIFNFHNFKTFNFSASSTYSMRAYTPAQVKANAPVDNKYYPQTTAQYNARLDLTPADNTKVRYNSAAHVMNNFNTINGGDDAAITLNSNRGSEENYSFIGTILAPHTNIIIQGGSQYSFAGNVITNRSIYLDGNVAVDKAFGANFNADGKFPGTSDLESTTKVPRILSVTLPNGVNFSPENVTTNYVFQFSLNNNVEIDPESETMHRLDGRAFKNTFLIDQPAGTSNVLWSRMNDGKWKKHEPDGKNPLQPNKTSEAQTVELSIADVTEDLKTKDNVDNYDDQTIYNAGKIDGNYVVNGVEKAFIGWHLLHKNKFSFIVASASDDLSTMTASEIKAKYDNTITNYTLNVHGDLLVNYPKVFDFGTYTLGTSEQEPVSIAKGQINVENPFYMDWKLVVKNSTSGADLVDNPLLQEDATGFNVQLYSLKTGTDDDWEAGQILNLGGLLKGPETPLLTLDTQKQLSGDEQLDAIQPAKSMPFYKSDVNLKFHNGKVDQCAKRPGKYKLEATWELSTADKVVDNTD

>fig|1664.9.peg.1572

MTLLVLAGTIGAGKTSLTKYLSDHLGTEAFYESVDDNEVLPLFYKDPTKYAFLLQIYFLNKRFDSIKQAYTDDNNILDRSIFEDSLLFHLNADLGRATATEVRVYDELLANMMQELPFAAHKKRPDLLIHVRVSFETMLSRIQKRGRSYEQLEADPTLYDYYKELNSRYDAWYDAYDESPKMQIDGDVLDFVENEADRKKVIDMIDQRVKELNEQQSKAI

>fig|1664.9.peg.1575

MLDIRVIRENVQWAKDKLATRGIDAAEIDEVVALDEKRRALIVRTEELKKNRNAASEAIAVAKRNKEDATEQIAAMKNVGAEIKALDTQLAEVKEKVDYILVRLPNFPDDSAPVGLDESFSREERKWATPREFSFEPLAHWDIGEKLGILDFERAAKVSGSRFVYYKGAGARLERAVYNFMLDQHAKEGYEEMITPYLVNGESMFGTGQFPKFTEDVYTMTNEGEPMTLIPTAEVPLTNFYRDEILDGAQLPIYFTALSPAFRSEAGSAGRDTRGLIRMHQFNKVEMVKFTKPEESFNELEKMTNDAENILKALNLPYHVITLATGDASFTSAKTYDIEVWLPAQNTYREISSCSDCTDFQARRAQIRYRDEDGHVNLVHTLNGSGLAVGRTVAAILENYQNEDGTVTIPEALVPYMGGMTKIERV

>fig|1664.9.peg.1578

MKMIQNEFKFIAKNKIIMISLIAIMFIPFLYSVFFLKSVWDPYGSTGELPVAVVNNDQPATYNGETLSAGKDMVKELKKNDQLGWRFVSAKKAKQGLKDKEYYTVVTLPKNFSANAATVLDKNPKKMQIQYETNDSLNFIGEVISEMGAKELNAQVRETVTKAYAQVMFKQVKTAGKGFSTAASGAKKLTKGSVTLSDGLNTYTAGVDKVNDGVMTMKTSVTPLSDGIQKLAAGSGALNTGLQTLNSKTGALASGASQLNSGANQLNTGLQTLNSKTGALASGASQLNNGAGQLNTGLQTLNSKTGALASGAKDLNDGAGQLNTGLQTLNDGTGQLAGGVTKLKSGLNALDSNSDQLVAGAGKVNGGVDQLIDGSQQLSGALSAAANMIDDQLDSKSEKLDLLKSSMGELQTGVEKLNAGINSPQADAISKQVTTDLTAVATDATSTGQSLQQINSKLYDKNVSTSAASQVANTGAQLQAITEVVSDPQIKAALSKDPALAQKLNNALQSAGTSVTATANQLASSGADLNTIKTNTEDTGVHLKSVQTQMTNLMTSLQALKKGVNELYVAAPQLKSGVDQAIDELTGGLKTVSAGLTKTSDKPSDMGAIQATDTINAGLRQIKLGLVGTPSQLGLIPGLSAYTAGVSKASDGTNELNSKVPALTSGVGQLATGSKALYAGTTQLNGQVPQLTSGVGQLATGSKALYAGTTQLNGQVPQLTSGVGQLAAGSKQLATGTGQLNSQVPQLTSGVSQLATGSETLNGGLGQLNAKIPTLTSGVGQLADGTSQLAANSAKLNDGAGQLTDGNKTLATALKGGAKQVNDIQLTNKTADMFAAPAELKHSNYSYVPNYGHALAPYVLSLALFVGAIVFNFAFPIRKVSMTGQSATAWYLSKISVGALVAVGMAIIEPGLMMIAGLNVDHPAQFFLVSIMFSLTSMAIIMFLSMTFDNPGRFLAMVLLMLQLGGSGGTFPMEITNHFYNVIHPFLPMTYSILGFRQAITSGLGNGQVLQTVFTLLLFMVVALILLWFGMNHLQKIGNGGRSVLDDNQKLQDLEK

>fig|1664.9.peg.1579

MPKYEEIADTLRQRIKQGIYPANSLLPNQVELVEEFDASRMTVKKAITILTMEGLVFARRGAGTKVLDHSFWNKNTAPADQYRGMSFDLADANQTLTSKIITFKVTFPSPEVKERLTLSAQQPVYEIIRLRIVDGVNSVLEHTYMPVSLAPGLTDEILEDSIYSYLKKDLHLTFAGGFRNIQADKADKYDQEYLDCAPTDPVLEVEQVVYLDSGQPIEYSRSRNRYDRRGYTYLDVNNC

>fig|1664.9.peg.1580

MNKPLIVITGASSGFGAEIAKIFNTAGYPELLLGRRADKIANLPLNFENVLIESVDVTNQAEFKTAIQKAEAKFGPTDLLVNNAGVMLLGNVLTQDAKEWQTMLDTNVMGVLNGTQIVLPAMIEHQHGTIINMSSLAGRKTFVNHAAYVASKFGVHGLSETIREEVSGKNVRISMVAPGAAETELLTHVTDQGALTDYQAWKDSMGGITLDPVHVAESVKFIYDMPQAVNIRELDIAATRQDS

>fig|1664.9.peg.1581

MTTYLIGEIATKMGVSVDTLRYYDKEGLLPFAKRNTAVHRIFTDDDLGYLEVIDCLKKSAIPIREIGQFIDWCMIGDETLPDRYDFMVEHEQQLEAKIKVLETNLAFLRWKKWYYHQAKEAGTEAIHFMPGTTQVDPKRHEEYELTLK

>fig|1664.9.peg.1583

MVLAIVQQKYVIHRYFGAHIRQYHESNTIRFTDRGLAVNPSTDRTVSLGELPLICPTRDTGDYRIPALTIQQASHNNHLDLEYRDYQIITGKPKLSDLPATYVVSDDDAQTLRINLEDLKAGVRVAMYYTIFKDLDIIATHQEVTNVGQRAITIQNCQSLSIDFTPQKLSWLSLYGAHINEANRNQHPIYPGIQKIESVRGASSPQHQPFFALLSPKTTEYTGVVYGFHLVYSGNFMGQVEQDQYGNIRAQLGLNTDTFDWRLAPNETFVAPEAIMNYSTAGLNGMSQNFHQLYQDHLVPRRFSHQERPILINTWEAMYFAIDAAKCEALAQEATDVGIELFVLDDGWFVGRNDDTTSLGDWSVDQTKLPHGIGQLADTIKQHGLLFGLWFEPEMISRKSNLYRQHPDWCLHVPNYEPMEGRNQLVLDLTRPAVQDYLIQMLRQHLSTGKIDYIKWDMNRHMSDVYSTAVDDSQQGEVWHRYILGLYHVLEVITNEFPTVLFEGCSSGGGRFDPGMLYYMPQTWTSDNTDAPSRVVIQDGYSLLYPPITMAAHVSAVPNHQVGRTTDIQTRFDIARFGNLGYELDLTQLSADEKQQIKQQTKIAKKERQLTQFGRFYRLVVTDDNYTAWLIINENRSEFNVLIYSELAQAAPHYPVFKLNYLDPNKVYQSDSGEQYGGDELMNVGLTLPRNKTDFHTVVYHFKSC

>fig|1664.9.peg.1584

MPTKLTDVAKLAKVSPTTVSRVINNYGYLSQKTIDKVHAAMAELNYQPNNLARSLQGKKAQMIGLIFPSVSNPFFGELIERLEKKLFDKGYKSILCDSNRKPEKEKAYLGMLAANQVDGIIAGGHNTELQEYEKMSAPVISFDRLLSTHIPIISSDNFAGGQIATNTLINLGAKRIGIITGSNETNSPTLLRLNGYLDALQKNGLDPFVFQIKSTTSNSLKKIEIARILREEHLDGLFCTDDLTAISVMNEAQNQNIKIPQDLKLIGYDGTELIQKYFPQLTTIIQPIDEIASLLAELITLRIEDPSAPLEMRYTLPVKLLQSNSI

>fig|1664.9.peg.1585

MPLITNWTRQLRYQPYSDWSIDYQQQLLNRVQNSPYRLNYHIQPDTGLLNDPNGFSYFNHQWHLFYQNYPMGPVHGLKSWHHLISDDLINWRKATPLLPDGNFDSHGAYSGSALPINDRLFLFYTGNVRNSDWERTPFQNGAWLSQAGTISKISEPLIKQPRHYTDHFRDPMIFKYQDQIMALIGAQDNNKIGKIAVFKADHNNINDWRPLGELTFTSEKLGYMVECPNLIFINHQPVLIFCPQGLTSEVISYQNIYPNMYVVGHSFELNTTTITQPSELTNLDDGFDCYATQAFNAPDGRALAVSWLGLPEIEYPTDKDGWAHCLSLVKELTLHDNQLYQYPVEETKALRQTKQILSLSKQSIIPTTNAYELELEVPANTSQTLHVFSNPSKSNGLVLTIDTTNGKITMDRSNAGVSFGQQYGQIRTTNVQPHHPIHINLFADQSVVELYINHGAKVMSSRLFPTENQNIIWAEIPQKAQLWPLSKVNNN

>fig|1664.9.peg.1586

MKVIDQAALDNDPDLKGTFNANGQYQIIVGPGDVNTVYDEFIKIVNIKEASKEELKNIAADNKDNIIMKFIKVLSDIFVPLIPALTAGGLLMAINNVLTGQGLFGAQSIVQMFPQWKGFAEIVNMMSSAPFTFLPILIAFSATKRFGGNPYLGAAAGMMLVMPNLVNGYGVAESIATGHMTYWHVFGLNIAQAGYQGQVIPVIGVAFILANLEKFFHKHLNDAVDFTFTPMLSIIITGFLTFTLVGPALRIVSNGVTDSLVWAYQTLGAVGMGIFGLGYSAIVLTGLHQSFPAIETTLLADIAKTGGSFIFPVAAMANIAQGAATFAVFFVTKNKQQKSLTTSAGISAMLGITEPALFGVNLKLKFPFFIGLIASGISSFIIGLLHVLSVSMGPAGIIGFIAIAPKSIPSFMMGAIISFVIAFVGTYLYGKKAMKTTEEEIINEAPATPEVVERLQDEKISAPVTGRIVDLASVPDPVFASEAMGKGIAIMPTSQDVLAPVTGVITIAADTGHAYGIKSDDGAEVLIHIGLDTVNLNGIGFEKIVQQGQHVSEGDLLGHFDIDKIKQAGLTPLTMTIVTNTAGYAQVDPLLTVDKAAMQGEEIIQLHAKKD

>fig|1664.9.peg.1587

MQTNWWQNAVFYQVYPRSFQDSNGDGIGDIQGIIQRLDYLADLGVNAIWLSPVYQSPNVDNGYDISNYQAINPEYGSMVDMEQLIEAAKIRKIKIVMDLVVNHTSDQHPWFLEARKSKDNPYRDFYIWRDPATDGSVPNDLQSNFKGSAWAFDAVTGQYYLHFYAKEQPDLNWQNPKVREAVYQMMTWWLQKGIGGFRMDVIDLIGKEPDRKIKENGPQLHAYLQEMNARVLSQYDVVTVGETWGATPEIGQMYSNPNRHELSMIFQFEQINLDKQSGMTRWDLKPLIPAELHAVFSKWQLALDGVGWNSLFWSNHDLPRIVSRWGDDSQYREKSAQALAIYLHMLKGTPYIYQGEEIGMTNYPITSYHEIEDIESRRVYQQRQQQGYAISDILNGINAKGRDNARHPMQWNGKNQSGFTDGTPWLPVNPNYLEINVELAKQDERSIYATYQQLIKLRKRNAIIRQGRFENVPTGNQNVISYKRMLGEQSWLVIINLSKNRESYTLPIQYFGPNQKIISNNLLLNKIATSGVLQPYDALVVAIGNEEK

>fig|1664.9.peg.1588

MSELLVGSVEAGGTKFVCAVGDEHYQVKDKVTFATTTPQETLARVVTFFKKFPNVTVITVASFGPIELRKNHAKYGYITNTPKIGWHDVDFVGYLKKELARPIIFTTDVNGSAYGEYVAARIAQQPVDSLVYYTVGTGVGAGAIIDGQLIGTLGHPEMGHVLVKRHPDDLKFEGICPFHGDCLEGLIAGPTFEKRVGKKGQDVDQSDPVWDIMAYYLAQAVVQQTLIIRPNKIVLGGGVVNKIFLDKVRLQFKEQLNNYVEVPKLEDYITMPLVENNGSATLGDFALALKQYNQK

>fig|1664.9.peg.1589

MATISDIAEQANVSIATVSRILNYDASLSVTEATKRKVFETAEALNYTKYKTKKHKKRKAKSTKATEKNIALFQWRQGDEEFDDLYYMSIRIGAEKAAQQLGYNLIKGDTASDAMQSVVGSICIGKFDEQTIKRIVKLSENAVFIETNFPLQNFDTVNSDFESALFNNIHMVSIV

>fig|1664.9.peg.1591

MMTRLKHFYRQHVVAVTLLFFLIVSIVMVAIVYLQPNKILSGADYHFHMNRVENLALSIKHGDFFPKISYFFIGGMGYAASLFYPDAFLYLPAILRVMGLSIKESFVIFAIFINLSTFLVTYGSGKLAGFTRKKAVIFALLYGLSIYRFADLVNRQAIGEVIALTFFPLVIVMMTRLKAGKHQYWYLLAVGMVAIGYSHMISVEMTGIFIVIYTLLNLKMLYKNGSFKYLIAAGGLSLGLLAQYFLAVGEQMATTTFQVSSQPLAFLSDRTLAFKEVVRNSLTNAVFHANTVNVGITILLGLGLACYLFWRSKNDRDLIAIALGLFIMVTPLMPWQLFDHTIINTIQFPWRFFAIITAIVSYLIAKDDLQLLNNKYYFYAIILMLGVGNVIYATNSIQNQSWRFRTNAQYNQPNPYYIGAGHEYLPAQTDYQKILKHKKQVVQYQPDKVKITNVRHTWRQYQFEFTTLKHQKAVVEVPFIYYKGYVANINHEKRLRPMQMNPQNGLTQITLKGKGKVTVFYQTTRLQKIGYLISVISFGGLMLIIYKEQYRHS

>fig|1664.9.peg.1592

MFLAPMVWYGPNIAVRNTLIPQLFGQIDPANEVWAFGIISAAATFTGAITNLLFGAFSDVTRSRWGSRKPYITIGTLVMAAMMVIIANSASVMTIIFLWIICAAGENAVAASIYAQISDRVAPKWRGTASTFYGVGFTISQQAFTILAAQFLGNIKFGIYAMALISVILGIVHLLLAKEPSNLDEPKISINKETFAKYFFFPTKGARDFYLALFAKFFMVVGSTIITTYTLFIFTDYMGVTSQQAGKSISIFSTLMLVFGVIFALISGPLADRAKRVKMPVLFPLFIAHPWAMYAYAVFAAIGNGIFNSVDGALNLNVLPSSDTAGKDLGLINLANTLSQMIGALAASAIVSTMDYQKIFLVAIILELIGAALIAMIRSVK

>fig|1664.9.peg.1594

MRFQRWLQVAKNQIALAVGLLIAVAYVWYWLGGLVVVMQGLLLVASVLGVAPILMQAYQALKVKVVSIDLLVTIAVIGAFVVGEYNESAIVTFLFLFGSYLEQRTLKKTRESIRSLSEMAPQTAQVVADDGTVTAVDVDDVAIGDHVLVKAGGQVPVDGTVITGSAFVNEASITGEAQLVKKEVGASVYSGAIVDNGTLTVVANQVGDDTTFAQIVELVEEAQDSKSPAEKFIDRFATYYTPAVLVLALVVGLITRDFKLAITILVLGCPGALVIGAPVSIVAGIGNGAKNGVLIKGGEVVNTMAGIDTMVFDKTGTLTKGETAVAQVVNYAADEATVLKLAAAVEKQSDHPLALAVVSYAQQQGLTALPIVAHIETLKGLGVKAQVGEQLVRIGRAQLLTDAGIIINKQQRAVIEASQRAGQSTVLVAINQTLALIIGIADTVKPEVKAALARLRKAGVKNLVMLTGDNEQTAAAIAREVGITEFHANLLPAEKVDYLKRYQAKGQKVAFIGDGINDSPSLALADIGIAMGSGTDVAIETSDIVLMQSTLSEVVYADYLTKATARNTVQNIVIAVGTVALLLLGLILGYVQMASGMLVHEISILVVILNAMRLIRFKVR

>fig|1664.9.peg.1595

MAKLVMQLDELSCPSCMQKIQAGVSQQAGVESVKVLFNASKVKTEYDETTTNPEQLTQVVTDLGYNVKSVKVK

>fig|1664.9.peg.1596

MLTTAAIEERYQAELKQADVDHNTPTAGAMIGHIMANLTIQRRKLRQIKWSTKGIDNGTFKAQVATLLTENDMLLDKTTERLLDEGEVIPTTQAEYTEYGMLTEDARIKYWDVPAMMAELVNDFNTANLFVSRAIKLAQKEERFALATDMIDMLGYNQHQIRVLQSQLGKDAREGLDEEDDD

>fig|1664.9.peg.1597

MATVEQHICAELVPIFSHLDHDSLLKVSALTHHEKVAKGQQVISPTAQKRLVILASGSIKVYQLSAAGKEQLLRIMEPGDFEGEKLLFTNQEEQVYGEALQDSVICTLPRAPFQELLLTYPEISLKLLEVTANKMIKLEQQANLMNIESVESRIVTYLLALVKVSETLTVKIPMKLKELATYIGTTPETLSRQLKQLEQRQLIKRQGRQVEILDYERLEDFY

>fig|1664.9.peg.1599

MDVFDYEDVQLIPNKCIVKSRSEIDTTVRFGSETFKIPVVPANMQTIIDEPLAIWLAENHYFYVMHRFQPEKRPAFIKMMHERNLFASISVGVKDDEFDFINQLAQDNLIPEYITIDIAHGHSQVVIDMIQHIKKVLPKSFVIAGNVGTPEAVRDLERAGADATKVGIGPGKVCITKIKTGFGTGGWQLGALRWCAKAATKPIIADGGIRTNGDIAKSIRFGANMVMIGSLFAGHTESPGELVEEDGQQFKEYFGSASEFQKGTHQNVEGKRILVPYKGSISDTLTEMRQDLQSSISYAGGKRLSALRKVDYVLVRHSIFNGDML

>fig|1664.9.peg.1600

MLDTYRFDEAKKKLTKHAKIQPNSWINITDPTQEEIDLLTKKLHVPSDFIYYSLDADESARAEHDPDYNATLIIFNMPILEKDDQQADKFRYKTSPLGIIVTDSVILTINKTPVDFLQSFIDNQIKNFNPQEQRRSVLQILYRISTIYLQYLRDINRSRERIENRLQKSLRNEELYDLMGIQRGLVYFMMSLKTDKMVLASLMRSNMLMLNESEIDLLDDIQIENQQAIEMAEISNSIINETADTYSSIINNNMNNVMKFLASYSIMLTIPGLVFSFYGMNVALPLANFKISWIVTIAISLTIAGLLGFRFWRKRYF

>fig|1664.9.peg.1601

MKKIDLAQQFLQANLSALQCPICQGAFATISDHQLVCEAGHSFDLAKNGTLYFLPKQMKSEYTKEMLSHRRAFLQAGFFKPFLDEIAKQLTANSRILDVGCGEGTPVYQLAQQVPAQYIGFDISKPAIQLASDYNQEGWFCVADLARMPFADQSLDTVLNIFSPSQYQEFKRVLKPDGQLIKVIPNAGYLHELRELLYQGQEKESYDHGPVLDLFEQHFPGYQEIQVQQKMTLTPETLADLVEMTPLTWTATEAQLQAITLAALPEISLDVTVLVGKQ

>fig|1664.9.peg.1602

MTNHIVLYEPLIPANTGNIARTCAGTDTVLDLIEPLGFETDDKHLKRAGLDYWDQVKINYHKNLDAFLETVPDRKHLYLITKFAKQAYSDQDVTDTSVDHYFMFGKETTGLPETFMRENEDQCLRIPMSDHIRALNLSNCAALVIFEALRQQDFPGLEKSHLYDHDKLK

>fig|1664.9.peg.1603

MQTKKDPISVDAFHFDRVSPDAEPQQNIQVSLVKIDADDEYLQEADLKAGNIYQIVTPFQVMPQGSGFAVSGQISRVVQLLDFFGTPDEIEQKEMMKLSRPLIEYIETLTYQVTAVALNEGIQLQFTAHETPEEA

>fig|1664.9.peg.1604

MATQTKKRKKKTTTKKKKTQQPDYTINFVGFLFILISLFSGLKLGFIGALFANVFRLFVGDTFVLGAILFAMLGIYLLIYGKQPPLWQQKTAGFSLAYIGILIWLSAFILHQQAINNHFVAATWRLLFEDFQKMAVTTEVGGGLVGAVIYSGSHFLFAQMGTYIIAVLVTVMGLFIFMDIPFSVVVKSSQRFVHGLFQVGRYTKSGLVKATEKGRDQVSALKEQTQNALSAVDEKTAPTKTAKKPTSEPTGEPEKTGVVVNEPATFKINGPKDLPKPIATHQATNEAEATAETPTEQVPDVAELTHAVEIDADYEMPTPELLTQIPPSDQSAEYEAIEKNQQVLKQTFDSFGVDVTVKSASLGPAVTKYEIQPAVGVKVSKIVNLADDLALALAAKDIRIEAPIPGKPYVGIEVPNTTVSTVSFREMIEQEPPHPGKPLAVPLGKDISGKVIMSDLAKMPHLLIAGSTGSGKSVAINGIITSILMSAQPHEVKLMLIDPKMVELNVYNGIPHLLIPVVTNAKKAANALNKVVKEMERRYQLFADTGQRNMTEYNQYIQGHNEDGEIKGAALPYIVVIVDELSDLMMVASNEVESAIIRLAQMARAAGIHMIVATQRPSVDVITGLIKANIPSRIAFAVSSGIDSRTILDGSGAEKLLGRGDMLYLPIGMSKPLRVQGAFISSQDVESIVSFIKAQKTAEYDEEMIPTAADEHQETADPEDEYYQEAVELVVKQQTASVSMVQRRFRVGYNRAARLIDEMESRGIIGPSEGSKPRKVLLQEVPSEQPPTE

>fig|1664.9.peg.1605

MNQKVMDGVYLNILQTQQFKTIRFSVQLVAPATKETLTKRSLLASVLSASSQAYRDQVAIANQLQAMYGAGFNMNAYRQGQLAIFSISMRIVSPQYLRDTIDLQQQAIAFIGELLLKPDVIDQAFNAVTFDREKENLQRYIDSVADDKQTYAALKLRELYFTDQTQQTPNYGDADVLKTITATDLYTYYQQMIANDRIQMTVVGNVDEEKLALDLAQLSLAPRGEMLPGIFYQQAASNAVRELTEEQAVQQGKLNMAYQLPAYFYQKDYYAALVMNALFGGTPLSLLFTNVREKASLAYYASSNIDAFRGAVMVQTGIESHQKERVLAIIEAQRQSIIAGDFTQAMFEQTKLALINQFKSSLDSPGYLGQQQFNQIFVPETELDAGRFTTAILAVSKQAVQEQAAKMQLQAIYFLKGVDTNEA

>fig|1664.9.peg.1606

MKLRQNPRLGETLYEETLENGLTVKLYPKSGYHKTYAILTTDYGAIDTTFVPAGQTDYVTVPDGIAHFLEHKLFEKADYDAFEKFGQFGASSNAFTSFTRTSYLFSTTSHLKENLDILLDFVQEPYFTTATVDKEKGIIGQEIQMYNDEPDWRLFYTVIGNLYPQHPVRTDIAGTIESIAQITPEELYQAHQTFYQPSNMNLFIVGQIDDPEEVLSWVSANQDAKDFVAPSDINRSLPEEEADGSDIIPYRSLEMPVTRSKSMVGIKGLTDFGTGQEALEMKIKMNLLLELLFGDSSTQVQKLYEQGILDDSFGYEYAIQRSFNFITLGGDTDDAQGLSDALINILEHYQESPDFSEANLALVKRAAIGEYLQAFNSLEAIANQYSDAFFDEVSPFDVLGLIEQVTLADLAQVAAEFFKIEIMTVCHILPEVAQ

>fig|1664.9.peg.1607

MSRSALIIGASGDIGTTIAERLAADGWSLYLHYNQNQERIYKLQQQLSQQYARQDFFALQLDLTTGTTAVDQLKKQLFQVDAVIFAAGMTTYGLLPEVSAEQIDALWQVHVKTPMLILQALQSKLAQSDLGRIVFIGSIYGGRGSAMEVPYSTVKGAQSAFANAYAQEVGSLGITVNVIAPGAVATKMNQQFTADEQAAIAAEIPTGRFATPTQIAYWVQTLLAPEANYLTGQTIYVDGGWLK

>fig|1664.9.peg.1608

MNEIGQKLREARIEKGYTLDDLQQITKIQKRYLSSIEEGDFDALPGTFYVRAFIKQYAQTVGVDSEALLAEYQDKLPNAKPEEYVEKSVENQTRLTRETTDSKWSKWRGLAPQIAVVAVVIVIVGIVYGITLSNRGKNRETLTETSSSVTVSGDQSTTKKASSDDNQQKAQESKAKEASKASEQSKKEADKKAKETKLSASMTSMSGSQQAFTIKNLPNKANTLTFSAENTTAWVGLIVNGSTIWQGSVTPGTDQTTQLPAGVTQFSVKTGNGPATKMKINDTAVNLNPNNETVQVRTVNFTATLAK

>fig|1664.9.peg.1609

MNLPNKLTMFRIILIPIFTILLAFNVPAGEVLVAGTAIPVSELIATVIFIVASLTDLLDGKIARSQHLVTNFGKFADPLADKLLVMTAFIFLVELGAAPAWVVAIIIWRELAVTGLRLLLSQGGEVMAAAMPGKIKTTTQMLAIILLFLHNVFFANIGLPMADIMLYVCLFFTIYSGTEYFIKNRGIFKDSFSK

>fig|1664.9.peg.1610

MAKDERQAALDAALKKIEKNFGKGSIMRMGEKVDTQVSTVSSGSLALDEALGVGGYPRGRIVEIYGPESSGKTTVALHAVAEVQKQGGTAAYIDAENAMDPKYATALGVNIDDLLLSQPDTGEQGLEIADALVSSGAVDILVVDSVAALVPRAEIEGEMGDAHVGLQARLMSQALRKLSGTINKTKTIALFINQIREKVGVMFGNPEVTPGGRALKFYSTVRLEVRRAETIKNGTDMIGNRARIKVVKNKVAPPFKVAEVDIMYGQGISRTGELVDMAVEKDIINKSGSWYSYGSERIGQGRENAKNYLADHEDVEDEVRLKVRAAYGISDVPEEDLPTTEDEQINILPDDSTEE

>fig|1664.9.peg.1611

MNLDLLLILTAVIMLIVGFAVGAILQKKAHEREIDGANKTAKGIIELAEKEAATRKKEILLEAKDENHQYRSEIENELKDRRGEVQKQENRLIQREETMDRKDATLDKKERALEEHENLLAEQAQQLTKKQAEVEALVEQQRTKLQEIAELSHDEAQKIILDETKQNLDHERAVLIKESEESAKEHADRTAKTLVAEAIQRSAADMVAETTVTVVTLPNDDMKGRIIGREGRNIRTLETLTGIDLIIDDTPEAVVLSGFDPIRREIARMTLEKLIQDGRIHPARIEEMVDKSRKEMDEQIRQIGEQAIFDVGIHTMHPDLIKVLGRLHFRTSYGQNVLNHSIEVAKLTGILAAELGEDVTLAKRAGLLHDIGKALDHEVDGSHVEIGVELATRYKEPATVINAIGSHHGDIEATSIISVLVAASDAISAARPGARSESLENYIHRLEKLESITNSFKGVDHSFAIQAGREVRVIVKPEQVTDDQATVLARDVKNQIEDQLEYPGHIKVTVIRETRTVEYAK

>fig|1664.9.peg.1612

MMPMFRIIVGLFATMLVSAAITPLVRRLAFVLGAVDKPNARRVNKKAMPSMGGLAIFIAFNVGTFILLRGQFPTHELFSIFLAECIIIITGMIDDIKELSPKEKMLGILIAGLVIYFLAGVRMNILTIPLVGTFKLGWLSFPITIFWILAITNAVNLIDGLDGLATGVSIIALFTMGVIAYFFLAITNVSVSIMIFCLVAALIGFLPHNFHPAKIFLGDTGALFIGFMIAVLSLKGLKNVTFITLLIPVIILGVPITDTVYAMLRRILNRKPISQADKHHLHHRLMQLGLSHRQTVLVIYGLALVFSLISLLYPLSTLWGSLALTIAVLFGLELFVESIGLVGENRQPLLHLLKRLLKPRDEEKD

>fig|1664.9.peg.1613

MESYLTVQADGQFELEIKKSRFICQIARVSDEAAVQAFIDQVRKTHTKANHNCFAYQLGQPANIQKQSDDGEPSGTAGVPILEVLRQMQLTNLCVVVTRYFGGTKLGTGGLIRAYSHATSAALEHLGIVQGIEQTACHLTIDYAQFDSLQNRLAQLNLAAQDIQYTTDIQLTVWLPTEEVPAFETQMTELLNGQLTLTLGQHQFNEVPISTQAIHDLRYQK

>fig|1664.9.peg.1614

MGQPVIACGRQFTAAQLADTQNNNYSLPQIERRPAFLRVKQRLICQRCQQVVPSQTCLPDGRHYCAQCLLFGRLVEGDWLYTIPEDHLFKTATPKLTWAGQLTVYQEQAAQAVVAVIQAHKWHVLTAVTGAGKTEMLFQGILVALQKGQRVCLAAPRVAVCLELYPRLQAAFATTSIMLMHGEQTEPYRYTQLVICTTHQLLKFYHAFDTVIVDEVDAFPFVDNPVLATAVEQACKPQCALLYLTATPTPAIKRAIAAKQMTVSELPLRFHGGILPEPQRHVAFNWRPRLKKGRLPKRLEHDCQICLKNQQILLFVPQVRLLKPVANRLSILLPAVRIETVHANDPEQIEKIAAFRAQQIQLMVTTTILERGVTFKNVAVLVLGAEHMVFNEAVLVQIAGRAGRHKDYTQNPVHFYYQDYTRAIKGACRQIKAQNQRGWRLQSTV

>fig|1664.9.peg.1616

MLSFNVRGENIEVTEAIRSYVEKKISKLEKYFDESATATAHVNLKVYPDKTAKVEVTIPLPYLVLRAEETSPDMYGSVDLVTDKLERQIRKYKTKINRKSRERGIKSVELVVSSEETLPEEPMQVVRTKRVSLKPMDSEEAILQMNMLGHEFFIFEDSETNGTSIVYKRQDGRYGLIETDE

>fig|1664.9.peg.1617

MANFLKSWVESDKREVGRMGKIADKVQSYEDEYSNLSDEALQAKTPEFKTRLANGETLDDILPEAFAVAREGAKRVLGLFPFRVQIIGGITLHEGNIAEMKTGEGKTLTATMPVYLNALAGQGVHVVTVNEYLSSRDATEMGELYNWLGLSVGLNLNAKTPEEKRDAYNSDITYSTNSELGFDYLRDNMVVYKEEMVQRPLNFAIVDEVDSILIDEARTPLIISGQAEKSTALYIRADRFVKTLKEDADYKIDWPTKTISLTEAGIGKAEANFGLDNLYDIENTALTHHLDESLRANFIMLKDIDYVVQDGEVLIVDQFTGRVMDGRRYSDGLHQAIEAKEGVEIQDETKTMANITYQNYFRMYNKLSGMTGTAKTEEEEFREIYNMEVISIPTNRPIARNDKSDVLYPTLESKFHAVVKDIKSRYEKGQPTLVGTVAVESSELLSRLLDENNVPHAVLNAKNHFKEAEIIMNAGQRGAVTIATNMAGRGTDIKLGPGVTDLGGLAVIGTERHESRRIDNQLRGRAGRQGDPGETQFYMSLEDDLMKRFGSERIKAFLDRMKISDDDAVIQSKMITRQVEAAQKRVEGNNYDTRKQTLQYDDVMREQREVIYKQRMQVIMAEDNLKEVIMPMISRTVKRIVQLHTQGDTADWNLEAIHDFATTSMVSEEQLTLEKLQGKSAEEIEALLMTFAEKNYATKQKQLSDENQMLEFEKVVILRVVDERWTDHIDAMDQLRNSIGLRGYGQMNPLVEYQEEGYRMFEEMISDIDYDTTRLFMKAEIRQNIRR

>fig|1664.9.peg.1618

MGDPDFWNDQQSAQQLIDQNNVLKEKYDSFNQLQAQFEELSVTLELLQEEPDDELQASFEADLEQIQSQMRQYELGQLLSGEYDSNNAIVELHPGAGGTESQDWGAMLLRMYLRWADAHGFTVETEDYQAGEEAGIKSVTLMIKGHNAYGLLRSEKGVHRLVRISPFDSAGRRHTSFCSVDVMPELDGSIDIEVRTEDLRVDVFRSSGAGGQHINKTSSAVRLTHLPTGIVVSSQAQRSQLQNRETAMNMLKAKLYQKEQEEQAKKAAAIKGEQLEIGWGSQIRSYVFHPYTMVKDHRTNYETGNGQAVMDGDLDPFMYAYLQWQLSQKNPN

>fig|1664.9.peg.1619

MIRMTNVSKEYPNGVTAIKDLSVTIDAGEFAYIVGPSGAGKSTFIKMLYHQLKATSGEIEVAGFDLVNMKDREVPYLRRKVGVVFQDFKLLPRLTVFENVAYAMEVIEAEPEVIQKRVLEVLELVGLKAKNDRFPNELSGGEQQRVAVARAIVNYPEVLIADEPTGNLDPETSEGIMDILEAINNKETIVLMATHNRQMVNERTHRVLAIEDGRLVRDEKEGEYGYED

>fig|1664.9.peg.1620

MKIRTLKRHLNDSFKSLKRNGWMSVAAVSAVTVTLLLVGVVFAMIFNFNKISNDIENDVHVRVMVERGTTNDQKNQLEKKLKKMASVKKVTYSSRKKELNKVVGSYGQSFKMFTGDDNPLYDVYMVSTTNPNKTIGVAKKAKKLAHVYDATYGGNNAKKLFSVMKNIRKWGLGFAALLLFVAVFLISNTIRITILSRRDEIGIMRLVGATNAYIRWPFLFEGAWTGLLGVIVPVIVIDFGYVWVYNHMALSMASAGYSLLRPGMFLFQLDLTMAILGIVIGALGSVVSMRRFLKI

>fig|1664.9.peg.1621

MEMFVNMLLTFFTTPLLWVAIVFAFWLALHRIKHERQTFRIAVNPKWPELKLFWWHGLLAGLALSCCTLLLGVTINLNWWLVYQIVAVVALFLMPIGFAGNVLILLSALLYLISAMIWPQYQILAGNSLLAELLIVAGLITILTGFLQKWDATKVVTPHVITSSRGRMTAMFTSRQIYLAPVFFLVPGTNQLPDWGFWPVLHIGQQSFNIVILPLILGFSVKAIKELMSALVTRDLRTQITFGGVIVVAGIVSFIFPSIILSTLSVALIICFILQWRLRRQSRANKQHFTKPYAGIRILGVLEQTPAAKMGLEAGDVIVECNGQAISNNENFYQAIQSHSTYCHLKVQDLQGEYRITESAVFADAPHELGVILFPEN

>fig|1664.9.peg.1622

MKTILVVDDEPAILTLLRYNLEQEHFKVMTATDGAQALTMALQQKFDFIILDLMLPKLDGIAVTKKIREAKLKTPIMILTAKDNETDKIVGLEVGADDYVTKPFSPREIIARIRAIERRTAQPIEQPVEQASETIVVGDLSVDETEVIAKKGDHKLHLTPKEFELLVYFMHRFNKVQSREKLLNAVWGFDYPAETRMVDIQVSHLREKIEDDPRHPVYLKTVRGFGYQLEDPAHDQA

>fig|1664.9.peg.1623

MTKHDQVRFYKLATILVAIYIAFILLINLVIYRQQSATLKERRDDYALIVKDADFKRQKWLQQNKLQVVTSQSPQHTKLERSVADVLNRDHTTRNFSVRQKIDGRWYLVAVHQSSQPTFVLLMPQQRFWHTWPEIALTVTILYLLFSLIVLWQFKRQRSHFYEHLKILVANIHHIRYEETPEPIIFQKDASLYVLAHEVNQLNKDMRHMRQKIAIQQGSFDRLIDHLPVGVMVINENRQVVLHNEAMGQLLETAIEPQKHPYIDDIKTYELTRMIEHTFRYRRSHHQETQLIQNRERFVDANVVQLNTVKSKFQALVILYDLTDIRRVEQMQLDFVSNVSHELKTPVTAITGFAETLLAGAKDDPETLQQFLKIIYEESTRLTALIQDILALSHLDRQDQTQAQDIGLKAIVEANLELMQQKIQQKEIHVSVNIPETLTFKTQKMKLNQVLKNLIANGITYNKQAGSLVIAAQVDHQQLIIRVVDSGLGIPSDLQERIFERFYRVDQARATHKSGTGLGLAIAKEITESIGGQLTVESQLGVGSQFILTFPLT

>fig|1664.9.peg.1624

MRKRSVIGLLVTFVVLLLTGCQSKQNGQSVTAVGSSALQPLIEALAEQYSAQHSGQFINVQGGGSGTGLSQIQEGAVQMGNSDLFAEEKAGIKAGKLVDHKVAVVGITPVINKKIGVKNLSLTQLAKIFSGEITNWQQVGGPDQEVVLVNRAQGSGTRSTFEQWVMGNRKTKPAQEQDSTGMVRSIVANTPGAISYLAFSYVDNTVATLKLDGVAPDDQNVMNNTWPIWSYEHVYTKGQPTGLTKEFLAYVLSDKIQNKQVIKMGYIPVAQMQVERSLDGTITKVK

>fig|1664.9.peg.1625

MDPIKASLLKKSRAAKTESRGKLISLLCISLIVFIVLAIFYFVASKGLATFFKDKVSLWRFLTQSDWNPSLKDSHGRPEVGALPMIMGSFGVTFLAALLATPFAIGAGIFMTEISPKRGEKILQPVMELLVGIPSVVYGFIGLSVVVPTMRHIFGGSGFGILAGTCVLFIMILPTVTSMTVDALKSVPRHYREASLALGATRWQTTWKVVLKAATPGILTGVVFGMARAFGEALAVQMVIGNAALMPHNLISPASTLTSVLTMGIGNTVMGSLANNALWSLALLLLLMSLLFNLLVRLIGRKGAMGR

>fig|1664.9.peg.1626

MDAKKWDKVATGVLTLIAGLIVLLLASLLGYILIRGLPHVTWQFLTNPASTFTKGGGIGVQLFNSFYLLVIAMVISVPLSIGAGIYLAEYAPKNWVTNVVRTAIEVLSSLPSVVVGLFGFLVFVIQFKLGFSILAGALALTVFNLPLLTRNVEDALKSIHFTQREAGLALGLSRYETVKHVIIPEALPNIITGMILGAGRIFGEAAALIYTAGQSAPSLDFTNWNPTYIASPLNPMRPAETLAVHIWKINSEGIMPDATAVSAGASAVLIIAVLLFNWLARVIGKRVYRKMTAA

>fig|1664.9.peg.1627

MADYDLTKTHIKKLAEPKHEIALSTEDLNVYYGDKRAMHDASLQFERYRITALIGASGSGKSTYLRSLNRMNDNIANTRVTGKIMYRDVDINSDEVDVYKMREHIGMVFQRPNPFAKSIYDNITFALKQHGQKDKKYLDEIVETTLKQAALWDQVKDSLNKSALALSGGQQQRLCIARAIAMKPDILLMDEPASALDPISTNTVEETLIRLKEQYTIVIVTHNMQQAARISDYTAFFHSGHALEFDETRKMFTRPKIKAAEDYVSGHFG

>fig|1664.9.peg.1628

MANKIIESSDVHLFYGKNEALKGINMDFAENEITALIGPSGCGKSTYLRCLNRMNDLIDSATVTGKFLLNGQDIYAPEMDTVKLRKEVGMVFQQPNPFPFSIYDNVVYGLRLSGVKDHRVLDEAVETSLKQAAVWDDVKDKLHESALSLSGGQQQRVCIARVLAVKPKVILMDEPTSALDPISSTKIENMLLELRQQYTIIMVTHNMHQASRISDKTAFFLQGELIEYAPTKKIFLNPTEKETEDYISGRFG

>fig|1664.9.peg.1629

MRRLFEDELNELHVRFSEMGMMVNEAIYKSVKAFINHDKQLAREVIADDHKINERETDLERKSFELIALQQPVSTDLRIIVTIMKASSDLERMGDHAVSIAKSTIRVKGNTRVPDIEKDIADMADKVKQMVEEVLDAYVKEDRRRAIAIAEEDTAINAYSENIYDDCVKEMQRDSETVVGSMDYMLVASYLERIGDYVTNICEWIVYLRTGKVVELNSNDKEDEF

>fig|1664.9.peg.1630

MQKQIYLAGGWFTPKQADMVARAHKALAANPTVGYIHSPQEHQYKDVTEDNDPHGLFGGYEWANQTYQNDITAMDLADLAVVLWDMADEDTGTAFEVGYLNASHTPIVFVCEDDLQENPINLMLAKGISKCVTDINELETLDFQFVAGEPYPGKIV

>fig|1664.9.peg.1631

MGAISAVMTKNDAILSDELNHASIIDGCRLSGAKIIRIKHQDMADLEEKAKAATESGLYEKVMYVTDGVFSMDGDVANLPEAVKIAEKYQLITYVDDAHGSGVMGHGKGTVKHFGLQDKIDFQMGTLSKAIGVVGGYVAGTQALIDWLKVRGRPFLFSTSLTPGSAAACITALDLIMAHPEYVDQLWENADYFKAKLKAVGFKVAKSETPITPVILGDEQLTQKFSQALVANGVYAKPIVFPTVPLGTGRIRNMPSATHTKAMLDEAVTVYEKVGRELGII

>fig|1664.9.peg.1632

MHKVMVTGCLGQIGSELVAQLRAQNGVDSVIATDIRRPDHNETVESGPFEVLDVTDYDRMLKIATDYQVDTLIHLAALLSAVAEERPQFAWQLNMTGLVNALEVARELDLKFFTPSSIGAFGPSTPKDNTPQDTIQRPTTMYGVTKVSGELLCDYYHTKYGVDTRGVRFPGLISYKTLPGGGTTGYAVDIYYEALRKGHYESFIKEGTYMDMMYMPDAIGAIIKLMNADPERLIHRNAFNITAMSFEPEQIKAAIQKELPDFEMTYAVDPARQAIADSWPNQIDASCAKAEWDFEPQYDLEAMTKDMLAQLETRI

>fig|1664.9.peg.1633

MNERQRILDLVNQGVLTSSEALILLENLAKNDQNTTKMSAEPEVPDIDDNEQETTPETDQRAIVEERARELTIALSGVNRQLQDLKQKIQADQEQVTVLDTMEDLDSLTSEKAAERQALKTAVLQNQAQIDDLEEQRQTLTEELNQIEKQRRQMAKNQWSEKLGLSDDWKENASETFDEISGRLGEASLHLGKLMKETAKNVMDNVDWKEVNFKVPGLATEKFTHEFIYSDVTPEMINVKVANGNVDFKIWDQPEIKVVAQVKLYGKMGEPTAREAFEARSNINVTDADLVFQVPNKRIQADLTFYLPANNYLQTNVKLLNGDIALDGFTGRDLYLKTSNGQLTLNSTNVAMLEAENVNGGITVTDGHYEDILGTTVNGNIVMKAQVLNSSVSTVNGDIKASFNDNQLQHLKAKSVNGTVKLALPQTIGFTLETRTRFGMIKNRLANAQTVDQHQSTGSQMLQLARNEDMQAAQLTLGTTTGNILLKDNEN

>fig|1664.9.peg.1635

MPFLLILVPILAIILAITVMGVLIGSVMILFKFAIPLLVIWLIYRLIVGHNKPRHHYHEQQHQAYYGQPSDSRGRKEARDVKVDDDNWSDF

>fig|1664.9.peg.1636

MRYWTRVLVNTILFIAISGLLPGLFHVDGILVALVASIVLSILNVLVKPILHILSFPITFLTLGLFSLVINGIMLELTSIFVSSGSTFPNVTFGFSGLGAAIVVAFILSIANTIIGNQFSRERY

>fig|1664.9.peg.1637

MADSVSMERLVTDLKLQVYSGEAHLKEKTVTLSDISRPGLELTGYFNYYPYERIQLFGMTEVSFTQNMTAEERLMIMRRMASENTPCFLISRSLEPPKEMLQAAEESGIPVLGSNLSTTRLSSLVTDYLDGQLAERRSMHGVLVDIYGLGVLITGDSGVGKSETALELVKRGHRLIADDRVDVYQQDERTVVGEAPMILRHLLEIRGIGIIDVMNLFGAGAVRADTSISLIIHLQNWSQDKKYDRLGSDTEEQMIFDVPVPKITVPVKVGRNLAIIIEVAAMNFRAKSMGYDATKTFEANLGKLIESNSDDEN

>fig|1664.9.peg.1638

MNLILGVLNPIALQLGPLAIHWYGVIIASGVVLAVVLALREAKRRNVSEDDVLNLILWALPFALIGARLYYVIFEWSYYQDNPSEIIKIWHGGIAIYGGLIAATIVFIIYCRKHFLSPWLVLDIAAPTVMIAQAIGRWGNFMNQEAFGAKTTLHFLETLHLPNWVTDQMLIDGAYRQPTFLYESTWNVLGFIIIMSVRHRQGWFKQGEILLSYIAWYAFGRFFVEGMRTDSLFIGPLRVSQILSLVLFFGAIGLWIWRRKQTLPDYLEGNTLRSAVK

>fig|1664.9.peg.1639

MTVNVAVLGAGSWGTILANLLVENGHHVELWGNDPEKVAEINEQHTNKHYLPEFKIDPRLHATLDLNEAFEAVDVVLFVIPTQVIRLVAEQIAPVLEAKGVKPVIVTASKGLEQGSHKRISEVLTETIPADVRNGIVVLSGPSHAEDVAMKDITTLTAASTDLVQAQWIQEIFMNDYFRLYTNTDVIGVEMGAALKNVIALGAGALHGLGYGDNTKAALMTRGLAEISRLGVAMGANPLTFIGLSGVGDLIVTGTSVHSRNWRTGNALGEGQKLDDVLENMGMVVEGVATCKAAYELAQQRSVDMPITNAIYNVLYLGCDIRTEIGNLMQRSGKPEIDFK

>fig|1664.9.peg.1641

MAKSYDVIVIGAGPGGMTAALYASRSNLSVLMLDRGIYGGQMNNTAEVENYPGFKSILGPELGEKMYASATQFGAEYGYGTVNSIEDKGEFKLVHTDEGDFQARAVIIATGSEHKKVGIPGEDEYGGRGVSYCAVCDGAFFKNKEVVVIGGGDSAIEEGLYLTQLASKVTVIHRRDQLRAQKILQDRAFVNDKMAFVWNAQVKTIEGDDKKVAAVTYVDKESGETKGVPASGVFIYVGIDPMTSAFKDLGILTEAGWVKTDDHMATSVPGIFAIGDVREKDLRQITTAVGDGGIAGQEVFKYIQSI

>fig|1664.9.peg.1642

MSWQDNYAIWNNQTKMDLPLREALDNMANDEEALEKAFSVPMEFGTAGMRGLLGPGINCMNIYTVRQATEGLAQFMETLPEATKKRGVAISFDSRYQSETFAHEAARVLGQHQIPTFVFDALRPTPELSFTVRHLHTYAGIMITASHNPKQYNGYKIYGEDGAQMPPKESDMITSYIRQVSDLFAIEVADEMALREAGIMALIGENVDQAYLAEVKKVTINQELVDTVGKTMSLIYTPLHGTGKVLGERALRQAGFEQFTMVAEQAVADPEFATTPFPNPEFPQTFDMAIELGKAQKADLLIATDPDADRLGAAVRQPDGSYQLMTGNQIASVLLRYILQASKDAGTLPANAAAVKSIVSTELATKIAASYNVKMIDVLTGFKFIAEQIQHFEDTNENTFMFGFEESYGYLVKPFVRDKDAIQTLVLLAEVAAYYKSNGKTLYDGIQELYAEYGYYAEQTTAIDFEGVNGPQQMADLMVRCREESPVDFAGIKVASAEDFQTQTQTFADGHTEPINLPTSNVLKYHLEDGTWIAIRPSGTEPKVKFYVGTSADDEATADLKLANFEKAIQALVK

>fig|1664.9.peg.1643

MGMHQYLQGLNNLETLNRAPGLFKYQEHSVAAHSFKVCEIAQLLGDIEEENGQTINWQVLYERALNHDYTERFIGDIKTPVKYATHELRAMLANVESSLTDNFINNEIPGFLQERYKRRLSEGKDETLEGQLLAVADKIDLLYESFGEIEKGNPENAFLEIFAESLKTIMQYQNLASAQYFIRKMLPDLLSEDFADRGKLTQLVRQTLSN

>fig|1664.9.peg.1644

MIDRQNDNLFDLVAPYEPAGDQPAAIETLTKNFEAGAKAQVLMGATGTGKTFTMSNVIKNLNKPTLIISHNKTLAGQLYAEFKQFFPNNAVEYFVSYYDYYQPEAYVPSSDTYIEKDSSINDEIDKLRHSATSSLLERNDVIVVASVSCIFGLGDPREYQNHVLSLRPGMEVERNDLLRQLVDIQFERNDIDFQRGRFRVRGDVVEIFPASRDDHALRVEFFGDEIDRIVEVDALTGEVIGERSHVAIFPATHFMTNDEKMEKAIESIKAELAERLAVLKGEGKLLEAQRLEQRTNYDLEMMQEMGYCSGIENYSRHMEDRQAGEPPYTLLDFFPKDSIMMIDESHVTMPQIRGMYNGDRARKQMLIDYGFRLPSALDNRPLTLPEFEEHVNEIMYVSATPGPYEAEQTDIQVDQIIRPTGLLDPNIEVRPIMGQIDDLVGEINDRIEKNERVFITTLTKKMSEDLTDYLKELGIKVRYLHSDVKTLERTEIIRDLRLGKFDVLIGINLLREGIDVPEVSLVAILDADKEGFLRSERSLIQTIGRASRNENGQVLLYADKITDSMRHAIDETKRRRTIQEEYNAAHNITPKTIIKPIRDAISMVQSVEHPEEIKMTNEIDLENMSKAEKLEMVERLSEQMRLAAKKLDFEQAATLRDTILKLKSEID

>fig|1664.9.peg.1645

MANDKIVIHGARAHNLKDIDVTIPRDKLVVITGLSGSGKSSLAFDTLYAEGQRRYVESLSAYARQFLGQMDKPDVDSIDGLSPAISIDQKTTSKNPRSTVGTVTEINDYLRLLWARVGQPICPNDGTEISSQSVEQMVDRVLALPERTKLQIMSPIVRGKKGQHKKIFEKIQREGYVRVQVDGEVMDVSTDLELDKNKKHDINIVVDRIVVKDGVRSRLFDSFEAALRLSDGYATADVIGGEQLLFSEHYACPTCGFTVGALEPRLFSFNAPFGACPECDGLGLKLEVDTDLVVPDTSKTLREGAIAPWNPISSQYYPQMLEQACTAFKIDMDRPFSKLTPRQKDIILNGSQGKEFHFHYENDFGGVRDVEVPFEGVLSNIDRRYRETNSDFTRTQMRTYMTELTCQTCHGKRLNRQALAVKVGGQDIAEVSDNAIKDGLPFFDNLELSEKDQVIAKPILKEVHDRLTFLINVGLDYLTLSRSAGTLSGGEAQRIRLATQIGSNLSGVLYILDEPSIGLHQRDNDRLIGSLKKMRDLGNTLIVVEHDEDTMRAADYLIDIGPGAGDLGGEVMAAGTPTEVEQNPNSLTGRYLAGQDYIPVPLKRRQGNGKKVRVTGAAENNLKDLTVDFPLGEFVAVTGVSGSGKSTLVNTILKKALAQKMNRNSAKPGQYKTITGYQNLEKLINIDQSPIGRTPRSNPATYTGVFDNVRDLFAQTNEAKLRGYKKGRFSFNVKGGRCEACKGDGIIKIEMNFLPDVYVPCEICHGSRYNSETLEVVYKGKNIAEILDMTVSEATEFFENIPKIARKLQTIVDVGLGYVTLGQSATTLSGGEAQRMKLASELQKQSTGKNLYILDEPTTGLHTDDIKRLLGVLERLVDEGNTVLVIEHNLDVIKTADYIIDLGPEGGDKGGTIVATGTPETLVNVAESYTGRYLKPILERDTARTLAAQE

>fig|1664.9.peg.1647

MTDTLNLVVITGMSGAGKTVAMQSFEDLGYFCVDNMPPSLLPKFWELVKESGKVTKIALVIDLRSRAFYDGIIEMLSGLDNTQFVTTKILFLDASDEELVSRYKETRRSHPLARNGRLMDGIHKERELLTEIRNQSQMVVNTSMLSPRELREQIFRVFKTSDNPSFHIEVMSFGFKYGLPIDADIVMDVRFLPNPYYVAEFKALNGLDKPVRDYVMEQPATEQFYQQLTALLKSIMPGYLKEGKTSVTIAIGCTGGQHRSVALAQRLADDLAVDYPVDVTHRDMKKRKESVNRS

>fig|1664.9.peg.1648

MTATNRIIRVIKGRRPKVVVIGGGTGLPVILKSLREQNADATAIVTVADDGGSSGVIRDYINVVPPGDIRNVLVALSDLPQLTLDIFQYRFKSHDAFFSGHAIGNLIIAALSEMESGIFDAVQRLSEMMAVDGHVYPAANVALTLNAAFQDGTELAGESEITAARKQIKRVWVTKTDPEDQEEPTAVQEVVAAIMAADVVVLGPGSLFTSILPNLMIKNLGEAVKQTKAEVIYICNIMTQKGETECFTDADHVRVLNEHLGQHVVDTVLVNTGQVPANYLDHQKYDEILWPVEHDFDGLRQLGCRVISDNFLKLDNHGVFHNGQKVASEIMNLAFQVNTTKRGE

>fig|1664.9.peg.1649

MASYASEVKKELTQLEVHPEHARAELAALIRMNGSLSLMNHQFVLNVQTENPAIARRIYSLLKQNYQVESELLVRRKMKLKKNNQYIVRLKYDTNMVLNDLNIVAEDGFTIHTRVSEDIIDEDQRVRSYLRGAFLAGGSVNNPETSRYHLEIYSLYEEHNQDIVRMMNRFGLNAKTTVRRSGYITYLKEAEKIADFLQVIGATNAMLKFEDIRIVRDMRNSVNRLVNCETANLNKTIDAAAKQIENIEYLRDSVGLDNLPAKLREIALLRLEFPDITLKELGEKMPSGAISKSGINHRLRKLNQLAEGYQQKVI

>fig|1664.9.peg.1650

MIKPVLLDEQLCFSIYRAQKAYNHFYGKVLKPYGLTYPQFIAMLALWEHGTMSVKELGHHLELDSGTLTPLLKRLEADGWVDRKRASDDERRVDVSLTEQAESQKLEIYERVGNCTNYLAFTSDKYHDLRQSMNEVEKHLNAIQADTDRFA

>fig|1664.9.peg.1652

MLVPTVIEQTSRGERAYDIYSRLLKDRIIMLSGEVNDQMANTIIAQLLFLDAQDSDKDISIYINSPGGSVTAGLAIMDTMNFIKSDVQTIAMGMAASMASVLLSAGTKGKRFALPNSTVLIHQPLGGAQGQQTEIEIAAREILKTRKRLNQILADNSGQTFEKLQADTDRDNYMTAQEAKDYGLIDDIMVNQPK

>fig|1664.9.peg.1654

MTHKMILDLDTGIDDALAIAYALGSPEVELIGITSEYGNVLTERSVVNSQQILHLLGHPEIPVYLGAGHSTMTNDFSVLPISAEIHGQDGVGEIHLTQPHPDAASQSAVDFILAACQQYGADLSIVATGPMTNLALAIQKDLPTLQKVGQIVIMGGALTVCGNVSPYAEANISQDPEAADLLFKSGLPVTMVGLDVTLRTLFTKKDTQEWRALATDAAKAYADMVDYYIKAYEVTSPHLHGCALHDPLAVAVAIDPSLVTTFPLNLKTEVEGPSRGRTIGDNARLDDPKTRTAVCVQVDTPRFLNAFKTRIGQLLKAVK

>fig|1664.9.peg.1658

MKISTLQYFVEVATEKSFTRAANNLYISQPTLSRHIQELEAELGVTLLIRHKYSVQLSDAGEKFFTETIDVLEKLDHLTHLFDDQKDVSQSSVVIRIGVLPNFNLSPLEERLATFKATHPNVKILLTDDTPMNLADGLNNGRYDLVFCLASYFAGNQNIEKHFFMANHLQIALPQKHHLAQQPSLKFSELESETFILLERKQSPIIVDYVVNQGLINGFNLRADYYVNNLDEGLTAVSAGKGLAFLYSGMNNGNLAQQYHIKIIDLEDVNQDQNIVTVTRKTNRNALLAQLTASLKLDNSGES

>fig|1664.9.peg.1659

MTVKITEEMIFSPSFNYLDNGIEHGVLSPFETKEVILKKGQQIAEGFKPLSCDIKMLKDTPVTLRDGVTIYADIYLPVTEEKVPTLIAWSPYGKSAGTAPRYKNLFNMLGMGNTWNSGLTKFEAPDPAYWVQHGYAVCNPDMRGIAHSEDNTTMLGPQEAQDGYDLIEWLAAQSWSNGKTALTGTSYLAFSQWYIAAEMPPHLTCINPTEGLSDGYRDLAFIGGIPDTNFIERLAVNHVSAKHAQREDLAKEMQSAPLADAPIWQDKVADPSQITIPAFVIASYSNTLHTMGTFRSWRTLGSKEKWLRIHDRQEWPYYYDQENTEELRRFFDFYLLGKDNDWLTTPKVRYSIIDFKGTNQTDIPATAFPPTESVTTKYYLNGQSRTLQLTPEQNDLPVSYLAGGLPGRTSFQMTFDQDTQFVGYPKAKLFMSVTDYNDLDVFVWIQKLDRRGNVLSEFVVPNHGAALQDFTQEGASALRYKGAWGRLRASMRNLDTKLTTPEIPAYTFDQVNKLTENEIVALDIVLSPLGLAYKAGETLRVVISSKDELGSVMPGTPGCTPDNHGTHTVHTGGQYASYIQLPLLNK

>fig|1664.9.peg.1666

MKIKAAIVEQQGAPFVIKDNIELAPLHPDDVQVHMVASGICHSDEALRKGDAIIGYPIILGHEGSGIVEKVGSNVQSLKVGDHVVLSFYACGICENCLKGMPTQCLNYAENNLSGVRPDGSSHFTENEHHVADMFDQSSFTTTTVVRERNAVKVPDDLDLRELGPLGCGYVTGSGTVLNTLKPKLGDTIAVFGTGAVGLAAMMAGKISGCTKVIAVDIIDERLALAKELGATDTINSRQTDDVVAAIQALTNGRGVNFCVDTTGITPVMEDSIKALCQGGVSATIAVTPNHIDLDTWNDLCVNDKSVVGVNMGDSIPQIDIPRLIEFYRQGMFPFDKTEKFYDFEDINLANEASIKGETIKPVLIIDPDYQI

>fig|1664.9.peg.1667

MTTIQPAPMAFFTNRPTTEIARDLLGTHLLYTSHQGTLGGLIVETEAYMGAQDTAAHAYNGRRTPFSEPLYHEPGTIYIYQLRSFFLFDIVTQAVDQPQGVLIRAIEPTHGLAQMQRNRPNKPSVNLTNGPGKLMGALGIHDKQLTFKNVATAPLTIDLANRRQPRHITTAPRIGVNAKAASGQLPYRYFITGNPYVSGTLKKDWDREQHGWL

>fig|1664.9.peg.1670

MSFFKRYIAMPQQYGLFPYVWLLFLLFPIAYSFPFKTLRQQVIIGLVLIFVIAYRNSYVATTYRPFWLLLQMVVSAILAVIVQALYLSIYTAWVFGSIPMRRRSFWGYYGAYMLSILVPTLFLYYYYGGQMGRDDWVGLAVYGLFCILSPFAAGSIQRYNRKNRQLMQTNQRLTEIIK

>fig|1664.9.peg.1671

MITLKAGYARKLLAKKPDAVPEQLLAIEQASRQNLKMVRDIVAGLRQTTIAEELINQERNLSVAGIILLTKDENQIEGLPQSNQQVLAQCLHEAVTNIIRYSHATECYVTFAQSATTFQMTIQDNGRGLKKADQFKSHGMAGMRERLEVIQGELTVDGRHGTTLTLTMPIVTEAKND

>fig|1664.9.peg.1674

MLKKRRTFIIGLGAALLCSLFLILNVANVQAKQVLQLGAQAPLDTIDISTSTGYGQTGNIFESLYRLGQKGKIEAGLAKSSQVSDDGLTWTFKIRKAQFSNGDPIRAQDFVYSWQRTIKPTTKSPYTNLFSNIKNAPAIADGKLDPKQLGVKALDKHTLQVTLTKPVAYMKTLMAYPLFAPQDQKVIEKYGKKYATKSKYMVYSGPFKLKGWSGTSEEWQFKKNPRYWDYKKVKLSAVKFTVLENTSTALYLYQDGRLDLTQLDNQQVENYSSNRDFKRYPYAQTYFLKYNFNSDNQQVKHILNNQDARLALSLAINRKTMNNRLYGFKTNPVTGFVASGLANSPVKKVDFAKSQAVPHTVDYEPKLAKEYWQKALKATGMKKVTLSLTVDSDDPNTSYVSQYLKGQLEEILPGFQLNLRTVPSQVASSRDHEGDYDILLSAWGADFKDPISFLEIMLPGAANNTGGFKNADYQKNVDLATNQDANDPSQRWADMVEAAQVLNRTQSLTPLYQNETGYLQNPKVKGIIHNTAGTQWSYKTAYIKGQ

>fig|1664.9.peg.1675

MAKQYETTMINEGGRNGFVYDPEKSTKYVIKAPGTDKTGKSTNPEQLFAAGYSSCFNSALELVLSQDQIKAETTVKATVSLYNDGPADFYIGVELVGHIEGLSPEDTKRYLEKAHEVCPYSKATRGNIDVKIDVM

>fig|1664.9.peg.1682

MTNQEIAKENIHTLYNSLMAHPDKSNALLDITDVLSQAYLTLETAKNPEALVNRLSNYIYSVGFGKIHLNKSEEQLLIDLGAYGQRAGWNGVYRGDCTSKAEFFNYSDARKYARV

>fig|1664.9.peg.1684

MYPVYLLEDDLQQQAIYQQIIANTIMINEFGMTLKCAASDTETLLAAIKDQQRGLFFLDMEIEDNRQAGLEVATKIRQMMPFAQIVFITTHEELTLLTLERKIAPLDYILKDQSMAEIKRQLIDDLLLAEKQNEAAAYHRENLFSYKIGPRFFSLPLKEVVYLYTEKENPGHINLLAVTRKVTFPGNLNALEAQYPMLFRCDKSYLVNLSNIANYDSKTRSLSFIDGSEAKVSFRKSRELVAKLKQMM

>fig|1664.9.peg.1685

MTDRIELSACTSMMVGKNASIDGSTMISRNEDRFYAIHPKRFFVQPAVHNRHETYVSKYNGLTMPLPEEGYRYTSTPNGDLSDGLNEEDGINEKNVALSATESVYANERVLAYDPLIKNGLAEDSMCSLVLPYINSAREGVKLMGDIVTKYGSAEGNGVQYSDENEVWYQEIVTGHHWVAVRIPDDCYAVAANQVAIENIDFNDPDNYMWSDGIQEFVSENHLNPSDTEWNFRKIFGTDTEKDRHYNTPRVWYAQRYLNPEIKQEPESSDLPFIRKANRKLSVEDVQYVLKSHYNETEYDPLGNGTEEQKTTYRAISLSRTQNSHICQIRNNVPDAVKGVQWLGFGVPTFCPHVPFFTNANDTDETYRELPEKMTLKNAYWLHEALAMVVESHYGVFKQADIDYQKALSEWARTKIAATDKAVENQTDAAAFLTQQNHEIATHYNEATTDLLAQLVTEGTQLSKLTFVMDKNL

>fig|1664.9.peg.1689

MLQEDTQQQEQARVDLTVDQVNQRLTVINQQLDNAYNETKDIEQRYGDTTKVNITEVDDRMETNAAVQQQKQLVAAAVENEKLLTSQQKRLTDLVDSPYFGRIDINEDGEEESLYIGTATFINEQDQFLVYDWRAPIASIYYNGTLGTVSYDTPNGPVKVDLTLKRQFKIKNGQIEHLFDTNETVGDELLQAVLGQQSDEYMQNIVATIQKEQNDIIRDTTADLLIVQGVAGSGKTSAVLQRIAFLLYHSRSHLEADQMVLFSPNRLFSHYISEVLPSLGEKNMRQVTMAEFLSHRFSGLQVETLFDRFEKDQASFPEMTQAIRRFKESAPFMQQITDYVKQVSTQPLAFNDLYFEDRPFFTKAQINKIYLDLPHNMRPGDKFIATKNTLIKRLKRQINLEVHLDWVAEKIDTLSDDDYRAIVGDREFDSGDLEQAFIAREIVQNYFADLYDAIYNDYFLDVYTEYQNFLRTACPAIIPVAVWEAMIETVAQNNERHLVGLSDAAPILYLRDLLTDSGQNHSIQYLFVDEMQDYSIAQFVYLQHAFPNAKFTVLGDYAQDVFTATYQAGNFIERLCAAFPKLKTNQISLTKSYRATAPITNFAKALLSKETALQAFSRDGRLPRVITATKEASINALTQEIFHLQKRHATIAILTKDQQTARQLFASLTPDDQVTLISETTRSLPKGVLVLPLYLAKGLEFDAVIGYDISATTFGQSSDVDLLYTLMTRAMHDLTLLGIGTLSPLITSLDPQYYQNITTTKETIA

>fig|1664.9.peg.1690

MAKKIILTGDRPTGKLHIGHYIGSLRNRVAMQDSGEYETFIMIADQQALTDNARDPEKIRNSLFQVALDYLSVGIDPAKTTVFVQSQIPALSELTMHYLNLVTVSRLQRNPTVKAEIQQKNFEQSIPAGFLIYPVSQAADITAFKATTVPVGDDQEPMLEQTREIVRTFNNVYGQEILVEPEGVFPPKGEGRLPGLDGNAKMSKSLGNAIYLSDDAETLEKKVMSMYTDPLHIKVTDPGHIEGNTVFTYLDAFDDDHEKVAELKAQYQAGGLGDVKIKRYLNEVLQAKFAPIRERRAEFEKNPAAVYKMLEEGSKKANAVAEQTLKEVRDAMGINYFN

>fig|1664.9.peg.1691

MHDFTKGNPLKVIILFTGPLLIGNIFQQLYSVIDTLIVGRTIGVNALAAVGSTGGLNMLIIGFCTGTTAGLSILTAQRYGAQDYRGVKRSFATSILISLVITVILTILSMVFSRQILVLMKTPTAIIADAQQFIMIIFAGIFASMAFNLLSNIIRALGDSRTPLFFLIVGVVVNIVLDFIFILGFKMGVEGAGFATVSAQVVSAVLCLIYIYRRIPALVLRRKDFRITPKEVREHLKVGLPMGFQSSIIAIGAIVIQIMLNTLGASAVAAYTAAGKIDQLANQVGNSFGVALATYAAQNYGARQYDRIRVGVRQTLLVSITFSIIMGALIITFGRPLVNIFIGTQQPHVTALAQIYFRYNSSCYFILAILFAIRYTLQGLGQSVMPTLAGLAELLMRIFAGIILVRMLGFTGASMANPLAWIGSCLVLLGSYFKTMRQLKRRAAKQTKKKESPNV

>fig|1664.9.peg.1692

MFKKRFIGVVALLALMLAGCQVQPATPHKPAQTTTTHKQAKTTTALAAKTFKSGDEPYLVVNHNKSTLDVRQWQTERIKYGALDDLNRTTTNTAYLSNETLGHSHTRARQNWRPTGWHNQPVMIDGHRVIPQNRGYLIAYAISFNFDEDGVFKKGQPGSNDNPKNLATQTEFSNQRTM

>fig|1664.9.peg.1695

MKNLGRRIYQGIQRHLTLLKGLFILSVLVFVIFEIGRIFRDLNGEQLRASLTTQSPVTLLAMLVIGFLAILPMLNYDFVIAELLPEEYSFWYKVKSGWIVNTFTNIAGFGGFLGASLRANFYGKKASQKEILIAISKIALFLLAGLSIWCLISIVLIFGFGIGQIYANYWLWLVGGACYFPVLLGSTRLKNAAFFSDLSVKRTLRLTAGSFFEWGFAGGFFLLIGYFLEIKGDLLQVLPLFMIANIIGVISMVPGGLGTFDLFMIFGLSAIGVGNADAVVWLSFYRVFYYIMPFLVGVGLFTHDAGSRLNHYLQGLPRQIMQKIAHHFIVFFLYISGIILLLVATVPNFAITNTVIGQLYPYTFFFLDRVTNIIVAFVMLGLARGVANRVKRVYWPLMILLVIAIANTLWRDFSIKLAVFLILVLLASLLLKRELYREKIEFSWNDRIVDGAIFVGVFLLYSFVGIANSPHLHHRKPVPTALLFPSERIWLVGFVGLLVAAATLFIVFRYLSRGGHKIATPFDETRISKVIAEYGGNEVSHLAYLKDKSVYFYQEDGEDQVFLLFRKKADRLIVMGEPVGNPDKFIDAIEALMREADLIDCHLAFYEISAELTMQLHEIGFDFIKFGEEGYVKLADFTLVGKKRRAERALMNKFEREGYQFELLTPPFSDDLMAELKAVSDDWLDGRVEKGFSLGFFDPNYLQQAAIAVIYDPEHRIVAFANDMPTGTKEVSSIDLMRHRKDAPSGIMDEVFISLFEHNKAEGYEYFNLGMAPLANVGTSEFSFIEEKIAHLIYEYGYHFYGFQGLRTYKKKYTTKWIPKYIAYQKRTSIIFTMLQILLVVNQKVTETPQRKGLAGHFMRFVQIGNLTNNQDK

>fig|1664.9.peg.1696

MTDYYYSKNPEVEHAEKNWTFELRGFNFKFTTDNGVFSKNTVDYGSRALLDAVDLSETPAGPILDMGCGYGPIGMTLAKLAPERQIDMVDVNERALGLAQKNCDLNQIQNVAIFESAEYQNVTAQYAAILTNPPIRAGKTVVQNILKGAYDHLLPDGELDVVIQKKQGAPSAKQLMADTFGNVQIIHKDKGYYILQSIKLK

>fig|1664.9.peg.1698

MIGKGVSELGYTQTEIDQWMQVAIDEANQARIIGEVPIGAVIVKDGQIIGRGHNIREHAQDATLHAEIIAIQEACMVEKSWRLEDTAIFVTLEPCPMCAGAIINSRIPNVYFGASDPKAGVTGTLMNLLTDKRFNHQATVVAGVREAECAALLQTFFKKIRENRRKKKKKTGNFKPNRVE

>fig|1664.9.peg.1699

MSYQALYRVWRPQRFDDVVGQETMTQTLKNAIITKQTSHAYLFTGPRGTGKTSAAKIFAKAINCHYQKDGEPCNECETCRAITAGALNDVIEIDAASNNGVEEIRDIRDKAKYAPTQADYKIYIIDEVHMLSTGAFNALLKTLEEPPANVVFILATTEVHKVPATIISRTQRFNFKRITAADLFKRMAYILEQKEMTYDPAALKVIAKAAEGGMRDALSILDQVLSFSDNHVTLENALDVTGSLTEALLADYVQTIHDHAPKAALQLLQQILAEGKDAQRFVEDLIEYVRDLLMYQQAPELVMANEMDLLDEHFKQLSDALSAEQLYTVIDILNETQQQLRFTNHPEIYLEVATVRLTQQPVAPAAVSANVSAPADNGQVTQLSQQVAQLQAALQKLETAGPSAPVQQAKPKPAKKISKKVNRKLIYPVLADATRTNLENLKEVWPNLLNMLDVTKRAIMRVSEPVAASQSGVVVAFNYEILFERANNDQDLLTVLENGLNRLTGNPFKVVLVPQDNWPEIRKEYLQNHAVSGHNNEDQPTTTTEKAPAPEVNEVVQQAQALFGDAVVVQKD

>fig|1664.9.peg.1700

MRGMGNMQGMMKQMQKMQKEMGQTQDELNSTEFVGKAANDAVVVTMTGDKKMKDIAIKPEAVDPDDIDMLQDLIIMAVNEAMVDIDKQTQAKMGKFTKGLPF

>fig|1664.9.peg.1701

MQYPEPIAKLIESYMKLPGIGNKTATRLAFYTIDMNEDDVTNFAKNLISARRDLHYCSVCGNITDEDPCEICRDTARSQEMILVVEQPKDVMSMERMNDYHGLYHVLHGVLSPIEGKGPDDINIANLIKRLQKTPAKEVIIATNATPEGEATAMYISRLIKPAGIKVTRLAHGLAVGSDIEYADEMTLLKAVEGRQEI

>fig|1664.9.peg.1703

MVGKLITFEGPDGAGKTSALEAVVARLQKEVSQEIVVTREPGGNPISEQIRQIILDVKNTAMDDRTEALLYAAARRQHIVEKIQPALAADKLVICDRFVDSSIAYQGAGRGIGEEAVAQMNLFATDGLTPDLTLYLDVPSEVGLARIKQHRQNQYDRLDQEKLAFHQKVRQSYLKLAQAHPDRIKTIDASQPLEAVVTQCLQVIAQANPHLFEV

>fig|1664.9.peg.1704

MKLLLAIVQDKDSNLLSSELIDANIRATKLSTTGGFLKSGNTTFIVGIEDERVPEALKIIEETCQAREQFMTPPINMDATMDSSMSYPIEVQVGGATVFVMPIEQFHRF

>fig|1664.9.peg.1705

MATLTIEQMQPKLVQQFAQIIARGQLAHGYLLAGAQGVGKMQLAQWLALRLFCQQVNEDSQPCYQCPECQRILTGNHPDVVEIKPDGKSIKIADIRYLKQEVAKSGMESSQRLFIIEAAETLTASAANSLLKFLEEPAPNVYAILCTSNKNLMLPTILSRLQVIDLANLPKEMVRQQFEEAQILPAQAQLLSHLTSDLTQAQQLLAEDWLMQVVAKITSWAQKVAQKDLESFVTIQTQLLPLLTDRSQQKIALNLIGLWYQDLLNCRYDLAGDLCFSEYRTQLNTISQQMTAAEIVAQTEFVLTAQRTFEQNVSCQNVLESLTLNLIAVA

>fig|1664.9.peg.1706

MSKTDLYEQLLTIEQQAKLTFDGITEMKAVLSKVLEENAELEIENKHLREHLQELQQTTEETDTKDLSQGLSKSKQNLQNLYEEGFHICPYFYGSRRENDEPCAFCNDVIYGERTAD

>fig|1664.9.peg.1707

MQTQSSFKTTTQGTLYLVPTPIGNLGDMTYRAIETLKDVQLIAAEDTRNTQKLLNHFEIETKQISFHEHNTQQRIETLIEKLEAGDDIAQVSDAGMPSISDPGHELVKACIEANIAVVPLPGANAGITALIASGITPQPFTFFGFLPRKGKELTETVAQLALKPETTIFYEAPHRLKKTLQALINGFGGERQVTLGRELTKKFEEFIRGDLQEALTWATDNEMRGEFVIIVAGNPTPQNLSVVATDPTLSLSEQVEAQIQQGASPNVAIKTVAKANDLKKQVVYNAYHDLTE

>fig|1664.9.peg.1708

MAGKQYTEEYRIPYFETDIKGELTLASLVNVLILASEHQLNDLNVGEETMHSLNLGWVVTQYQMTINRMPKVDEKVRIVTEAESYNRYFCYRNFWLYDEAGNECVFVQSIFVMMSYETRSMVPVVPEIMAPFESMAIKGSKRFPRIKKIDSEKASQKEYRVRYFDIDGNQHVNNVHYFEWMLDALDYDFLMDHRVTSVNIRYGHEIQYGQMTQSQVEQVMVDDIMTTRHKVAVDDLSAVEAEIAWTKR

>fig|1664.9.peg.1709

MTEKKELILAFESSCDETSVAVIENGQRILSNVIATQIKSHQRFGGVVPEVASRHHVEQITLCTQEALEQAGVTYDDLTAVAVTYGPGLVGALLIGVTAAKAIAYAHHLPLVPVNHMAGHIYAARFVKPLEYPLLALLVSGGHTELVYMPAAGQFEIIGDTRDDAAGEAYDKIGRVLGVPYPAGKEIDRLAHLGQDTFNFPRAMLKEDNLDFSFSGLKSAFINTVHHADQIGETLDQADLAASFQTSVVEVLVTKTLRAAQSLKVKQLVVAGGVAANQGLREGLAAGIESAGLDLDLIMPPLRLCGDNGAMIGAAAHIALAQNTLADLDLNAVPSLDFPYQNEL

>fig|1664.9.peg.1710

MIDDQPFYLARASQSDIPALIDIERSVYGGKTPWDRWAFASELSKRRTSIYLVLCQGSEVVAFVGAWFSTNEAHITNIAVRPSFQKRGIGHFLVHEVVALARDYPSQKITLEVRTGNTGAQSVYRRMGFKVVKTRRNYYVQEKEDAFSMCRILSQ

>fig|1664.9.peg.1711

MKLLAMDTSNQAVSVALLEDQQILGEMTVNIRQTHSQTLLPMIDQLLKQTKTSIEAIDRFVVAQGPGSYTGLRIAVTTAKSFAWTMAKELVGISSLALLAGNVQETTALIVPLFDARRDNVFAGVYQWQDQHLQNVVADQYLALTDLLAQVALLNEPVYFVGGDVQQFNAQIKATLGSQAHFVDANHNLPHAAVLGQLGANAEPVADIHGFGPHYLRKTEAEVNWGKTHDDQGSAAYVEQV

>fig|1664.9.peg.1712

MVNQDRLFGVKLDTRRVVLLAVLIALQLVIARFAISLTIYRISFGFIVTALMGWWFGPVWAGLAAVLGDLINSLMIGVPGGYFPGFTLSAFLGAFIYGCFFYRQKITWLRVILAVLTVVVVVNLGLNSWWVSILSQTPISVHFATRAVGEAATMVIHPIVIYLVMHFEPIVRLKDRIQ

>fig|1664.9.peg.1713

MKKILVLHTGGTIAMSADQDGAVAPTGQNPMAGFEKLFDNQLTIISEEFANLPSPHITPTVMLALKNRIQAAEATGIDGVVITHGTDTLEETAYFLDLTLPNNVPIVITGAMRSSNEIGSDGLHNLISAVWTAASDDAHDKGVLVVMNDEIHTARYVTKTHTTNVATFRTPTFGPIGLVSKHDANFFQELIRSEICDIQELVEPVFLLKAYAGMDGTLFEAINQPTTKGLVIEALGAGNLPPATLPAIQALLDRNIPVVLVSRCFNGIAEDVYDYDGGGVKLKKMGITFCQGLNGPKARLKLQVGLSAGKTGADLVNFVSNAVS

>fig|1664.9.peg.1715

MAEKTIMLCCAAGMSTSLLVSKMQKAAEAQGVDAEIFATGASDADNQLENKKIDCVLLGPQVRFMEGQFKEKLAPKGIGVEVIDMKAYGMMDGETVLKQALALIG

>fig|1664.9.peg.1717

MILLQVQQVARHFGGDYLFKNAQLEVQDHARVALVGPNGAGKSTLLKMIAGITTPDDGQIILGKNVQVGYLAQDSGLSSERNIYDEMLTIFEGLRQMEAQMHTLEQAIADPDANRSDEDYQALLKQYDQIQHDFSEANGYGYEAEIRGVLHGFQFPEETFTKPISTLSGGERSRLALAKLLLEKKDVLILDEPTNHLDIQTLTWLENYLQTYPGALLTVSHDRYFLDKITKEVYDMSRHTLTHYTGNYSHFLTQKSANLSLEWKAYEKQQAEIEKLQTFVDKNLVRASTTKQAQSRRRQLEKMDKLERPTNDASKIHFHFDIDQPSGNEVLQVVDAAVGYSADKVMVEPINFEVNKQERLAIIGPNGIGKSTLLKSILHQIPFLKGHERVGSNVSIGYYDQEQRNLHANKTVLNELWDEHRTTPEKEIRTLLGSFLFTGDDVLKVVSQLSGGEKARLLLTKLAMNHDNFLIMDEPTNHLDIDSKEVLEQALREFDGTVLFISHDRYFINQVATGIIELSTTTGSKRYLGDYDYYLAKKAEEEAFAQAAEVPVEVSDQPVTAQDNYKASKETQRAQRKLEREVAALEEQMATLEEQATAIQEKMTQADIITKPLVLQELQQDLEKVQADLAQTEADWEEQAMALEELTN

>fig|1664.9.peg.1718

MAENKIPRATAKRLPLYYRYLNFLNNSGKTKISSTELSEAVKVDSATIRRDFSYFGALGKRGYGYDVESLLSFFKKTLNQDRLTNVALIGIGNLGHALLNYNFRQTNNIRISAAFDVKPELIGTIQTGVPVYSMDEMVEQLKLQQIEIVILTVPIEVAQKVTDQLVDAGIKGIMNFTPIRISVPNTIRIQNVDLAKELQTLIYFLDNYNTEEASK

>fig|1664.9.peg.1719

MTQSQQNFRLIISYLIILLIPSALVRFFGLSDQFYLFTTITDSLGALLLLYFNYRGQKNSLEKHPLPLKKAIIWGISGILLALIVQIIAGSIDHLLFKNSTQSLNTLTLMTALKAQPWLIIILTIAAPTMEELVFRKAIFGGLSGRLNSVLAALISSLLFAVIHQDSHLIIYAAIGLFLCWLYRKTGSIYTTIISHAGMNLVVTLFYLSH

>fig|1664.9.peg.1720

MLKPLEDRVVIAVKDEAEQTVGGIVIASNAKQKPQTGKVVAVGAGAMTSDGQRIPLDVKENDEVIYDKYAGSEVEYEGQQYLVLHAKDIIAIIE

>fig|1664.9.peg.1721

MAKELKFSEDARSKMLAGVDQLANTVKTTLGPKGRNVVLEKAYGSPEITNDGVTIAKAIELEDHFENMGAKLVSEVASKTNDIAGDGTTTATVLAQAIIREGMKNVTAGANPVGIRRGIELATKEAVKKLHEISHKVESKDAIAQVAAVSSANEETGRLIADAMEKVGNDGVITVEESKGIDTELSVVEGMQFDRGYLSQYMVTDNDKMEADLDNPYILITDKKISNIQDVLPLLQSIVQEGKALLIIADDVDGEALPTLVLNKIRGTFNVVAVKAPGFGDRRKEQLEDIATLTGATVITSDLGLELKETTIDQLGTAGKVTVTKDDTTIVEGAGSKSAIAERVENIKKQIAETTSDFDREKLQERLAKLAGGVAVIKVGAATETELKEQKYRIEDALNATRAAVEEGYVAGGGTALIDVIESVAALEEEGDVQTGINIVLRALEEPVRQIATNAGLEGSVIVEKVKSQPVEVGYNAANGNWENMIEAGILDPTKVTRSALQNAASVAALMLTTEAVVADKPEDNPAPAAGMDPSAMGGMM

>fig|1664.9.peg.1722

MQQLKRIFIGKPLKSSDEGNQKLGRFKALAMLSSDALSSVAYGTEEIVVVLTTLSAAAIWYSIPIAAFVLVLLISLTLSYRQVIHAYPGGGGAYVVSSENLGKNAGLVAGGSLLVDYMLTVAVSISAGAEAITSAIPAMYGHQVGISFVIMIILMAMNLRGMRESASFLMVPVYLFVLMMTLMIIVGCYKIFTGAIPFHATATVGAVVPGISMALIFRAFSSGSSSLTGVEAISNAVPFFKKPRATNAAETLAIMAAILGFFFAGITFLNYWYGIVPMEKVTVLSQVAQHTFGKGFFFYIVQFATAFILAVAANTGFSAFPVLAYNLAKDKYMPHMYMDRGDRLGYSNGIITLAIGSMALIFIFNGDTSRLIPLYAVGVFIPFTLSQSGMLRKWWVERPKNWFGKSIANFVGALISFAILIILFVYRLPDIWPFFIIMPIMIWIFYKIHDHYKKVARQLRVSANEAVLHDYDGSTVIVLVSNVTQVTRGALDYAQSIGDYVIAMHVSTDENPEKEREIQAEFKADFPNVRFVDVHSSYRSIQKPIVRFVDIVSKNAQKRNFTTTVLVPQFVPRKPWQNILHNQSSLRLRTAFASRENIIISTYSYHLKH

>fig|1664.9.peg.1723

MRILFIGDTVGTMGQKMVNEYLPLLKQQYKPQVTIINGENIADGKGMTQKDYKNLLQAGADAITMGNHTWNKRDILNFIDDAKKLVRPANFPKGTPGQGMTMINVNQAKLAVINLQGRALMGELLEDPFAMLDDMVAQVSKETNCIFVDFHAETTSEKLALAWYLDGRISALVGTHTHVQTNDARVLPDGTAVLTDAGMTGPYNGILGVKREKIISRFMTQRPERFELVTEGPGQLNGCLIEIDDQTGHAKSIKPIHISPDHPFGSRF

>fig|1664.9.peg.1724

MPQKTKETPMMAQYQKVKDQYPDAFLFYRLGDFYELFNDDAVKASQLLELTLTARNKNAADPIPMCGVPHHAAQNYIDILVDQGYKVAICEQMEDPKTAKGMVKREVVQLVTPGTVMDEKAGHAKQNNYLTAVVAQGDQFGFAYTDLSTGEMKVSQISSLDVLLNEMLSLQTKEVVVNHDVPSEVVQAFVQQQILVSYQGEGADTAEVSFVSQNISQPLALAVIKQLVVYLATTQKRHLGHLQRAQAYEPSQYLKLDHSAKMNLELTASLRTGHKSGTLLWLLDETKTAMGGRLLKQWLERPLLDLNQIKNRQNQVASFLQHYFERTNLQEALTKVYDLERLAGRVAFGSVNGRDLIQLKTSLEQIPKIKDVLTGINETQAFDQALTRLDPVDDVRTLIETAINPDSPISVTDGGIIQDGYDEQLDQYRNAMSNGKQWLAQLEAQEREATGIHNLKIGFNRVFGYYIEVTRANLSSLPEGRYERKQTLTNAERFITPELKEKEQLILEAEERSTALEYELFTQVREQVKLQIERLQTLAKGVAALDVLQSFAVVSESYHYVQPTLRTDSCEIDLVDGRHPVVEKVLGRQKYIPNAVQMGKETDMLLITGPNMSGKSTYMRQLALTVIMAQMGCFVPAKSANLPVFDQIFTRIGAADDLISGQSTFMVEMMEANRAIMSATANSLILFDEIGRGTATYDGMALAQAIIEYIHDHVHAKTLFSTHYHELTALADTLTALRNVHVGAVEENGELVFLHKMLAGPADKSYGIHVAKLAGMPETLLQRADVILGQLEDKPKAPVQPATVAPAQPAAAAVEEQMALFEPEVAAPVDKKTDKVVAAIKNFDLMSATPLEALNQLYEWQKQLNKH

>fig|1664.9.peg.1725

MMGKIHELSEILSNQIAAGEVIERPASVVKELVENAIDANSTQVDIVVEQAGLQMIQVIDNGDGIEPEDVPVAFKRHATSKIATRQDLFKIQSLGFRGEALASIASVSDLTIETATADSLGTFAHFKGGLLEEQKTNPIRPGTAITVRDLFFNTPARLKYVKTFQTELANIVDIVNRLAMSHPQIAFTLTNDDHLLLKTAGNNDLKQTIAGIYGVTMAKKLLAVSASDLDFKLTGYVSLPELTRATRNYLSILINGRFIKNYQLNKAIIKGYGSKLMVGRYPIAVLAIEMDPLLIDVNVHPTKQEVRLSKETALMTLIEGAIKERLATENLIPDAMQNLKRTKTVDVDQLQMNLNQITQERRHPSGTVRPVSPMPIPVKTVTPEIAETETPLAAPTAKAPAITPERPLFEQPQALAEWDQKYATEAVGKPFGDQVVDETDESEENVVEPTVRFSELRYIGQLHSTFLLAEGEDGFYILDQHAAQERIKYEYYREKIGEVSTSVQNLLVPLVLEYPNSDALKIQEKTELLASVGIQLENFGQNSFVVHSHPTWIGQGQEEMTIRGMIDDVLKDGRISIAQFREKTAIMMSCKQSIKAHHHLDDAQAKALLADLAKTENPFNCPHGRPVLIHYTQKDLEKMFKRIQEPHHSWEGE

>fig|1664.9.peg.1726

MFILASQSPRRQALLKRVVNDFEVQPAQIDEHETPLTAPGDYVQTLAQRKGEAVAVQYPTATILAADTAISFQGTLYGKPKDRQDAYEMLRQLSGQTHQVYTGLWLMKDGLVQQKVVQTDVTFWHLSTAEIEQYLDQNEYADKAGAYGIQGAGALLINKVNGDFYNVVGLPVSTVARMLQN

>fig|1664.9.peg.1727

MVNVAVFCGASSGRRPVYTQAASDLGHWLVKRQDQLIYGGSRVGLMGTIADTVLENGGRVVGVMPEFLNQREPAHAGLSDLVMVDSMAERKSKMIELADVFIALPGGPGTLEEMSEVISWARIGQQDSPCIFYNVAGYYDLLQQFLAHMVEEGFLTQGDFEKYLFTDSLTEIATFIATYQPPKIREY

>fig|1664.9.peg.1728

MYEYLKGLVTAVNPYYVVLEVQGIGYQLQVANPYRYTESMSEVVQIFVHQAVRDTDITLFGFYDLDEKQLFQKLISVSGIGPKSALAILANSDHSGLIQAIMNDDIGYLTKFPGVGKKTAQQIALDLKGKLGDLEQSATLVGQTAIDLGSQGDSPELSDALAALSALGYSAREVKAITPKLTDFAAQTTDQYLREGLRLLMKK

>fig|1664.9.peg.1729

MADERIVSAENDDFAEASIEKTLRPQVLAQYIGQDRVKNELAVYIEAAKKREESLDHVLLYGPPGLGKTTLAMVIANELQVQIRTTSGPAIERPGDLVALLNELQPGDVLFIDEIHRLPKMVEELLYSAMEDFYIDIVVGQGPTAHPVHFPLPPFTLIGATTRAGLLSAPLRDRFGIVEHMAYYTEADLMDIVQRSAGVFNMSIVPDGALEIARRSRGTPRIANRLLKRTRDYAQVADQNTIDQAIADHALSQLQVDIRGLDGVDRKILQMMIDYYQGGPVGLKTIAANIGEENETIEEVYEPYLLQIGFLKRTQRGRMVTPAGYAHLGMPYPEK

>fig|1664.9.peg.1730

MVSTEDFDYNLPEELIAQTPMIERAASRLLVMDHETGALEDKVFYDIIDELNPGDAVVMNNTRVLPARLYGVKPDTGGHEEVLLLNNTHDDEWEVLMKPAKRAKVGTEVVFGDGQLRAIVTKELEHGGRMIEFKYDGIFMQILEALGEMPLPPYIKEKLDDPEMYQTVYAKEPGSAAAPTAGFHWTEELLQKVQDKGIKLVYLTLHVGLGTFRPVSEDNVEDHKMHSEFYRLTEEAAATLNEVKQSGGRIVATGTTSIRTLETIATKFDGEIKADSGWTEIFIKPSYQWKAVDAFITNFHLPKSTLVMLVASFTGRENILNAYQHAVDERYRFFSFGDAMFVK

>fig|1664.9.peg.1732

MTSPIHVNSEIGKLKTVLLKRPGKEVENITPDIMYRLLFDDIPYLPTIQKEHDQFAQTLRDNGVEVLYLEYLAAEAIDAGDVKEAFLDKMLNESHIKSPQVQAALKDYLISMATLDMVEKIMAGVRTNEIDIKSKALIDVSADGDYPFYMDPMPNLYFTRDPAASMGDGLTINKMTFEARQRESMFMEVIMQHHPRFANQGAQVWRDRDHIDRMEGGDELILSDKVLAIGISQRTSAQSIEELAKVLFANYSGFEKILAIKIPHNHAMMHLDTVFTMIDYDKFTIHPGIQGAGGMVDTYILEPGNNDEIKITHQTDLEKVLRDALEVPELTLIPCGGGDAVVAPREQWNDGSNTLAIAPGVVVTYDRNYVSNENLRQYGIKVIEVPSSELSRGRGGPRCMSMPLVREDLKK

>fig|1664.9.peg.1733

MTNSVFQGRSLLAEKDFTKSELEYLIDFSLHLKDLKKKGIPHHYLEGKNIALLFEKNSTRTRAAFTTAAIDLGAHPEFLGKNDIQLGKKESVEDTAKVLGSMFDGIEFRGFSQKVVEDLAKYSGVPVWNGLTDEWHPTQMIADFMTVKENFGKLKGVTLTYVGDGRNNMANSLLVTGSMLGVNIHIVAPDSLQPTQEVRDLAEGYAKETGSKNMITSDVDAGVKGSDVLYTDVWVSMGEEDKFEERVNLLKPYQINMDMVKKTGNENMIIMHCLPAFHDIETEYGKKIDEQFGIQEMEITDEAFRSKYARQFEEAENRMHSIKAIMAATLGNLFIPQA

>fig|1664.9.peg.1734

MTKRKIVVALGGNAILSTDASASAQIKAVKETVKQLVAFVKQGDQLIISHGNGPQVGNLLIQQAASDSEKTPAMPLDTVGAMSQGEIGYWMQNAFNEVLAEEGLALDVATIVTQTIVDAKDEAFQNPTKPIGPFYSEAEAKKQQSINPEAHFVEDAGRGWRRVVPSPRPIGIQEAPVIQKLVEGNVITISAGGGGVPVAKEGNKLRGVEAVIDKDFASEKLAELVGADMLIILTAVDNVYVNFNKPDQKKLTNVSVAELEDYIKDDQFAKGSMLPKIQAAIEYVNNRPDSKAIITSLDNVKNLLAHDAGTIITK

>fig|1664.9.peg.1735

MEIANFGVEEWLNVYETQATLDIAQSTIASMTMSELMALSPDNGTQFYQDLAQQKMNYGAIEGSEAFKQAVSELYQTVNSNQVLQTNGATGANLLALYALVKPGDHVISMFPTYQQLYEIPISIGATVSYWQLDEANNWVPDIAELKKLIRPETKLICLNNANNPTGTVISTELMQAIVEVARTVGAYVLVDEVYLPLGEGQQTTSIADLYELGIATNSLSKTYSVPGIRVGWLVANETLTDLFRKYRDYTMICAGVFSDQLAVYVLQHRKQVLARNRALVQRNLKIFKAWVAQEPLVDVVYPESVSTSFIHFKEIEDDEAFCKYLLKEYGVLLVPGKRFEIPGHARLGYCAPEATLKKGLAELSKALRTY

>fig|1664.9.peg.1736

MTEEKPAKKIGLLALIALVISSSIGSGVFGLTSDLASASAPGPVLIAWVIVGFGILMLALSLNNLLMKEPELEGIFSYAEKGFGPFAGFISGWGYWLSAWLGNVAFATILMSALGYFFPIFKSGQNLPSILVASVLSWSLTYFVNRGVEGAAAINTLVTICKLIPLFVFIIFGIVLFKGHLFTQAFWNNMSSSFVAGDVMSQIKNCMMVMMWVFVGIEGASMLSARAEKKSDAGKATILGLVSLLAIYILASVLPYGYLTQDQLASIKQPAMLYIFEQMVGTWGGYFIGVGLIISILGAWLSWTMLPAETMLLMAKQNLLPAYFGRVNKKKAPTFALVVTAGLIQVFLFTLLFTTKAYNFAYSLCTASIIVCYMLVAAYQIKYSWAHLQEKGNRQQLLIGVLALLFEIAGILMAGVSYLLLCFIAYIPGIYFYGRARKNNGHQHFLSKGEWLITTIIVIGAIIGIWLVVSGKIVI

>fig|1664.9.peg.1737

MIAADEYPRYIQYLRTRPEFIDFSDEEMNILMNNMKVKDFHKGQELFDQTDERSRFYFVVSGLVRAERTDESDEFTFYTYIKQNLAFPYRGMFTDQYYPYTARAMTDIEIIYFPMATFEHLLQKNTDSIVKVVQEMGSIISESEDQIQRMVTSSAKQRVIQALKIFEINLGEPLRCGRSYIPYPITVKELATMSGTTRETAGQIVKQLVAQKLLSYEHKEFRFEKAFFKDDLA

>fig|1664.9.peg.1738

MGFYVPIIVNALKECIKKGDGMMDDAEIIKTKKRFKLKMPGAFVILFILTVVAVMATWVVPSGSYAKLSYDQKSTQLVVTKPSGQVEKVPATQASLDRLGVKIKISQFTSGSINAAVSIPNTYQRLKQRPASLAAVPNSMVRGTVEAVDIMVFILVLGGLIGTVKASGAFESGLLALTKKTKGHEFLLIFFVAILMVLGGTLCGIEEEAVAFYPILVPVFIAMGYDSIVCVGAIFLASSIGTTFSTINPFSVVIASNAAGISFTEGLLWRVGGCIVGAIFVIYYLYRYSKKVKADPTQSYSYEDHDAFDKMWAITSEGAQSKAVFTWRKKLILVLFVVTFPIMVWGVMSQGWWFPTMAASFLTFAIIIMFLTATGPEGIGEKGVIDAFVNGASSLVGVSLIIGLARGINLIMNEGMISDTLLQYSSSLVAHVSGPIFILIMLVIFFVLGFIVPSSSGLAVLSMPILAPLADTVNIPRFVVVTAYQFGQYAMLFLAPTGLVMATLQMLDVKYAHWLRFVWPVVAFVLTFGGAMLVLQVLIYS

>fig|1664.9.peg.1739

MEPAIKYRLIKKEKHTGARLGEIVTPHGTFKTPMFMPVGTQASVKTMAPEDLKEMGAGIILSNTYHLWLRPGEDIVEKAGGLHKFMNWDRGVLTDSGGFQVFSLAKLRDISEEGVAFKSHLNGEKLFLSPEKAIHVENALGADIMMSFDECPPFFESYDYVKKSVERTSRWAERGLIAHQNPATQGLFGIVQGAGFEDLRRQSARDLVGMDFPGYSIGGLSVGESKGEMNRVLDFTTPMLPEDKPRYLMGVGSPDALIDGVIRGVDMFDCVLPTRIARNGTTMTSQGRLVVKNAKYSEDFRPLDPKCDCYVCKNYTRAYIRHLIKADETFGIHLTSYHNLYFLINLMHQVQDAIEQDNLLEFREAFFEEYGYNENNGRNF

>fig|1664.9.peg.1740

MLNLVLGANPFGGNSSFLIILVLMMVMMYFMVMRPQKKQQQKHQEMVNQMKKGDQVVTIGGLHGVIDSVNNDTKIVVLDCDGVYLKFNLSAIRTVEPTAKATETVVEETVDETKEETK

>fig|1664.9.peg.1741

MVKKEGVKAVVDAVSEVDKMVTDLVTRAHEALKIMETFDQAKVDHIVHQMAIAGLDHHMELAKMAVEETGRGIYEDKAIKNIFATEEIWHAIKDNKTVGVIEEDPEHGITKIAEPVGVIAGVTPVTNPTSTTIFKAEIAIKTRNPIIFAFHPNAQKCSARALEVIKEEAVKAGLPADALLFIEEPSLAATQALMNHTGIATVLATGGPGMVKAAYSTGKPALGVGAGNAPAYIEESANIKQAVNDLILSKSFDNGMICASEQAVIVDARIYNEVKKEFQAQGVYFAKASELPALNEAIIDPAKNAVRPAIPGQSAANIAKLAGIDIPEDTPVLIAEIKGVGHQYPLSHEKLSPVLAMIKAKDREDGLALCEAMLDLGGLGHTASLHTTDDALPLEFARRMKACRVLVNTPSAQGGIGDLYNEMIPSLTLGCGSYGHNSISHNVSTIDLLNIKTLAKRRNNMQWVKLPSKIYFEKNSVNYLEKMADLNKVFIVADQGMVNLGYVRIVEEVLAKRANDVQMQIFSDVEPDPSTNTIYKGAAAMRSFEPDAIVAIGGGSVMDAAKGMWLFYDSEEADFFGAKQKFLDIRKRTYKFPKLNKTKLICIPTTSGTGSEVTPFAVITDSETHIKYPLADYALTPDVAIVDSQFVESVPPRVVAHTGLDVLCHATESYVSTMASNYTKGLSLQAIKLVFDNLKASYDGDITAKGNMHDASTMAGMAFANALLGINHSLAHKLGGAFNLPHGLMIAITMPHVIRYNATTPTKRALWAKYSYFRADEDYAEIARYIGLKGNTTAELVEAYANAVTELAESVGIQMSLKANGVTKADFKQHVDELAELAYEDNCTVTNPKEPLIKELKGILEAEF

>fig|1664.9.peg.1742

MAMTPNKEDYLKIIFELGGDAKKVTNKEILAGLNVSAASVTEMVNKLVKENYVKHTPYQGIQLTSEGAREAALLVRNHRLWEVFLVDKLHYQFNTVHPEAEQLEHVTNHDLAERLADFLGHPKRCPHGGIIPNAKGEFEQQSHHALVDLEVGEKAVIERVLDDNDLLKYTLEIGLTVGDTVTLTKVGLFESPITVMDETQQTEIQVGIKAAQHIFVTPIAAD

>fig|1664.9.peg.1743

MRSDLAALFIIFGGSGDLAQRKLYPALFNLYRKGYLKESFAVIGTARRPWTDEHYHEIVMASLEGFEEDDETKQTFTSHFYYQSHDVTDAEHYETLKALAAKLDDKYQLNQNRIYYMAMSPRYFGTIASHLGSEALLTADGFNRLIIEKPFGRDYESAKALNQELTQTFDENQIYRIDHYLGKEMIQNIVALRFGNGLLSGVWNNQFIDNVQITLAESVGVEERASYYETSGALRDMFQNHIMQVIGMLAMEVPVNFSAEDVQREKTRLFRSIQPYSPAEIKQNFVRAQYAETTVDGENKPGYRQETNVAPDSQTETFVAGKVMIDNERWCGVPFYVRTGKRLARKETQINIVFKKTGVNIFADDKSDRSAMAPDVLTIYVEPEQGFSLTINGKEVGQGFSLKPDQLNYQYDKQMLADSPEAYERLILEALNGDGTNFTHWHELAASWQFVDAIRQVWDADQTPLPTYSIGSMGPKEATDLLASTDAKWIFDPTQTKK

>fig|1664.9.peg.1744

MAELFKIPLENDTSRKIIHIDMDAFYASIEEREQPDLRQKPLIIARDPSDTGGKGVVTTANYVARQYGVHSAMSAQKAAELIPKHQAVFKTPNFPLYQSVSAQIHDIFHTVTDKIEPIAFDEAYLDVTENKLGLTHTIEVVAYLQKAILEETQLVSSAGISYNKFLAKMASDYRKPAGRTLVLPEQAIAFLSRLPIEKFRGVGQKTVPKMQDLGIMTGADLLAQSEMFLMQHFGKLGYGLYRHVRGIDNRPVEYQRERKSIGNEHTYGQPLISEEQVQVQLKSLAVELAQRLRKNQKHGLTVVLKVRNRQYETITKRKTLGEYVGNAAELEAIANQIWQSIGQAEVGIRLLGITVTNLAPQTFENITLPLYDWEND

>fig|1664.9.peg.1745

MATKHDQILMYIENLPIGDKISVRSIAKTLAVSEGTAYRAIKAAENNGLVSTIERVGTIRIEQKLKGKIENLTFAEIVQVIDGEVLGGKDGLNKLLDKFVIGAMQLAAITRYITPKSLLIVGDRIDVQRLALENGAAVLITGGFHTSDEVIALANELALPILQTTYDTYTVATLINRALTDQLIKKEITTVASIYRPFSETYSLLATDTVGDYKKLSAAVKHSRFPVINEHRRLVGIVTVKDILDKPASMTLDKVMTKEPASVKKYMSIASVGHAMTWNSLEIMPVVADNLELEGIITRQDVMSNMQTAQRQPQVAETFYDQIIELLHPSESFPEFYSAAYQFEVSPQMTNNLGTISFGVLSEAVSAACQRMLQATHQRHSMIEQINLHYLRLIQIESQIQILPRILEIGRRSAKIDVDVYANHVIVAKAIVTCQILERG

>fig|1664.9.peg.1746

MTIPEQIIAKIKAYQTIIIHRHVNPDPDAIGSQVGLAEVIRNSYPDKKVYQVGSDTGNLAWLANAQPIDPALYQGALVITTDAANTPRISGEDYNQGAYLIKIDHHPNDEPYGDLNWVLPDASSTSEIIVDMINDSQGQLQLNQAAARVLYAGIVGDTGRFMFNSTTPHTMTCAAQLMTFDIQHDKINQRMNEITLEQAKLQGYLFDQLQVNDDQVAYLVVDQETQKRLNVSAQQVHSIVGTPGRLKGVLTWVIFVQQDDGTYRVHFRSKGPIINELAKQHDGGGHPLASGAKAADQAEIDTIITALSALAADYTAQ

>fig|1664.9.peg.1747

MDNQFTQFNLKPEIITALDEMHFTKPTAVQRRVIPAINAGKSVIGQSQTGSGKTHAFLLPLLNKLVPEKDEVQIVITAPSRELAYQIYTAAELITKHMNGDIRIGRYVGGTDKQRQIEKLQQHQPQIVIGTPGRILDLITSKALKIYTAKTLVIDEADMTLDMGFLKPVDNIASRLPEDLQMMVFSATIPDKLQPFLQKYMHNPVIEEVKNETIISPTIENWLVPTKGKALNQTIYSLITMGHPFMVLIFANTKSKVDDIHHYLQSQGLKVAKIHGDIPPRQRKRIMKEVHNLDYQYVVATDLAARGIDIEGISMVINAEVPREDLEFFVHRVGRTGRNGLKGTAITLYGPDDEQAVAQIEAMGIKFTPKAFKDGELVTSYDRNRRKKRVATTHELDVNLKGFVKKAKIKRKPGYKKKIKEAIQKDARQKRRIEQRVEMRERKRNNKKQNQ

>fig|1664.9.peg.1748

MVHENDLQLPNWLQLSEQARRANFGQVLRYFVSPLVAIEGIQPEVVQAGRLTFKTYSAWFSGEQFVFIPAQSAVSLGWSLGADKLAPSEWFAQTNRQLAANEGQLWADKADVDAYVEQLTTPSRIVNMPALLLARESVPRDWLAQGRYSPITRHFTGNQLFFSRFQAEIQNGLYRTQPETPLLETAHLVLRLLADQQVYQVFEKMTFTDQQLRQTLRQQGYDFVSVDQYEWLRGAGAPTLWPSGNHLPQPMAESAASFGLDFTADQQAYEITTDPLVYKGGPCVTGGRYQLERQLPSSPFYESGVSTALMGNTDLATRYRRTITVALE

>fig|1664.9.peg.1749

MKQLTSAQVRQMFLDFFKEKGHDVEPSASLIPDEDPTLLWINSGVATLKKYFDGRVVPNNPRITNAQKSIRTNDIENVGKTARHHTFFEMLGNFSVGDYFKEDAIPWAWEFLTSDKWIGLDPEKLYVTVYPKDKDAHRIWHEKVGLAEDRIIEVEDNFWDIGEGPCGPDSEIFYDRGQSFNNVSEDDPENYPGGENERYLEVWNIVFSEFNHLPNGEYVEQPHKNIDTGMGLERLVSVIQEAPTNFETDLFMPLIEATAAMSDHKQYGQNAADDISFKIIADHARAVTFAIGDGAMPANEGRGYVLRRLIRRAILNGKKLGINDAFMYKLVPIVGEIMHSYYPDVLEQKDFIEKVIRSEEDRFRETLNDGLRLLNQTIEAVKAENETEINGADAFKLFDTFGFPIELTTEYAEDEGLTVDQAGFEKEMAAQKARARNARSDEQSMGSQNEVLLNIKTKSEFTGYSELETESRLADIIQDNAEQESVTTGVAQLVFEATPFYAEMGGQVADQGIIKNLAGQVVAEVTDVQYAPNGQPLHTVKVLKEMISGVHYTLSVDPTFRGGVVKNHTATHLLDQALRDVLGEHTHQAGSLVEPDYLRFDFTNLGQVTPDQLAEVEQIVNDKIWAALPVNTVETDIETAKQMGAIALFTEKYGKTVRVVQIGDYSMELCGGTHANNTSDLGLFKITSESGIGAGTRRIEAVTSKAAFKYLNDKQVLLNELAGELKVAQTKDLPKKIQQLQADLKAEQKENEQLKAKFAQEQAGNVFENVVEAGQWRLVAADAKVAGMNELRQLADQWQQKQISDVLVLATVAGDKVSLIVAVAPDAIKAGIKAGDLIKQIAPLVGGGGGGRPDMAQAGGKNPAGIPDALAAAKEFLA

>fig|1664.9.peg.1750

MNLDKTVHFDFGKNSPKNVHETLVTVYQTLEDKGYNPINQIVGYLLSGDPAYIPRYNDARNLIRKHERDEIIEELVKNYLDKGNTK

>fig|1664.9.peg.1751

MRLMGFDVGSRTVGIAVSDLFGWTAQGVEIIRINEDESEFGLDRVKELVAQHQVTGFVLGLPKNMNNSIGPRAVKAQEYGAMLTELFPEIPVDYIDERLTTVQAERMLVEQADTSRKKRKQVIDKLAAVMILQNYLDAKGPLTKQGY

>fig|1664.9.peg.1752

MTETNKNIHQEAEQEITLIDDQGNEELYQVLFTFDSEDYGKSYVLLYPASSAEDEEVDIQAFAFTSDVAGDASQGDLFPIEDDEEWEMVEEVLNTFLADDNMQ

>fig|1664.9.peg.1753

MQAVIKMTPAQLKTIQQTYPSNRPLPPGAVFAHRGNGLNVTGYRSGKVLFQGATAEAAAAKWGPLAPAKKSKATKGSAKGDTLPPDFANWSVIGSDEVGNGSYFGPLTIAAVYVSQENLSFVRNLGVADSKTLTDAQIDQMAIKMMTKLPYHIVNIMPQKYNQVHEQMNIEKMKAVSHNFALLKVLKKIQPDQPQGILIDQFEPRATYYRYLKDEPVVLRDNVYFTTKAEQFHLAVAAASIIARHQANLSMIELSKLAGRDIPVGAHKSIDKIAADLLDQGGLEYLGQFAKLHFINTKKAQALRS

>fig|1664.9.peg.1754

MLSLIIILMLLHAVYTGMRRGIVLQVVYAIGYFIAFWIAVPLSQTLGPKLNLLVPYPSATEESYFAFFQQKVGLSLDKAFYIGVAFVLVMFISGLIIRFIGLLLNSLTFIYVFSRFNKVLGGVLNFIVVYIGIFLVLYVLALVPVDGLQNMLAHSWLAKMMVLKTPLLTSLVTQWWIVG

>fig|1664.9.peg.1755

MNHKILKTLEYDKIKQMLQGYAITAFGQEQIATLEPINEADLIQIRLNQTKDGVDIERLKGGIPLPQLENIRPHLKRIEIGAMLNGSELAQIGRVLRATSAVVRFFDDLEKDELELKALPELVAQFVTLPQLTELIRSSVADDGAILDTASTKLRGLRTGLKQLEGQIRSRMASYTHGAKAKYLSDPIVTIRNDRYVIPVKQEYRGQFGGVVHDQSASGQTLFMEPQAIMELNNRLRQLQIEEQQEIERILAELSEAIMPERHNILANAELLGQLDFVNAKAQLAKALKATEPLINAENHVDLKQARHPLIDATKVVANDIAIGADYQAIVVTGPNTGGKTITLKTLGLVQVMAQSGLFITAREESQVGVFSDIFADIGDEQSIEQNLSTFSAHMENIIQILKQIDDRSLVLLDELGAGTDPQEGAALAIAILDQIGIVGANVVASTHYPELKIYGYNRPQTINASMEFDVATLQPTYRLLIGVPGRSNAFDISTRLGLPNSIVDQAKQLMNDESQDLNNMITDLENQRKAAETEYQALRHELTEATDLHQQLSTAYQQFFEDRETEMTKAKEKANAIVEKAEVKADKVITKLRDMQMNQGAQIKENQLIDAKAELGQLHQETTLKKNKVLQRAKRRQTLKVGDDVLVTSYGQRGTLIRQVDSKNWEVQMGIIKMKIANDDLEKQKVVEDNRPQRHVTTVNSGGARHVKAQLDLRGKRYEEAMAEVDQYIDAALLANYQQVTIVHGKGTGAIRQGVQEYLQANRQVKKYEYAPANAGGNGATIVTFK

>fig|1664.9.peg.1756

MVDVLTDKDFEAETSQGVVLTDFWATWCGPCRMQSPVIEELAQTNDKVKFTKMDVDANPDTPAKFGIMAIPTLLVKKDGQVVETITGFHNKEQLTETLAKYTD

>fig|1664.9.peg.1758

MTNSIIQKIDTIAIEQPQTVAYQYGQTQYSYADLKVASDRIAAFIQDQVLPKGAPVIVFGGQQFEMVATFLGAVKAGHAYIPVEQHSDAERIQQIESVAKPAAVLSWASASQIEVDMPVFQADILADVVASGATDYDAQQSVQGDDNFYIIFTSGTTGLPKGVQISENNLLSFVDWANPAFGVADNSRVLIQAPYSFDLSVMNLYPGLCSGATLVILEKEITDNFKTLFEVLPTLKVNEWVSTPSFVEICLLAPTFDSEHYPELREFVFCGEELTHQTAEKLLERFPSAKVFNTYGPTEATVAMTSIEITADILAQYDRLPIGYTKADTKTVVVDEQNNEVAPGQPGELLISGPSVSKGYLNNPEKTNAAFFEKDGQRFYHSGDLVVADDNQLIFYKGRTDFQVKMHGYRIELEEIDHHLGQLAQVKQACTVPRYNKAHQVTQLIAYVVPAEGQVGDATLTKTLKAALAENTMAYMIPQRFVYPESLPLSVNGKVDRKALIKEVNGNA

>fig|1664.9.peg.1759

MPNLQPYANPNYFIILGIALVPLIIGFLNGKRWHIYETLVSLLFLVLIFDGDKINQGLALIGYVIFEGALIWGYTVYRQKRNNTALFDLAVVLAIMPLVIVKFVPFLNPGRNSIIGFLGISYLTFKAVGMIMETRDGSIKKFEPWLFLQFMLFFPTISSGPIDRYRRFKKDYLKVPERDHYIDMLGKAMHNFMLGFVYKFILGYAFGTLLLPRMAHLAMASRGGFLDISWGLVGYMYVYSMYLFFDFAGYSLFAVGTSYVMGIETPINFNKPFSSPNIKEFWNRWHMTLSFWFRDYIYMRLMFFLMKHKITKSKVLIANLGYLTLFLLMGFWHGLTWYYIAYGLFHAIWIILTDVWLRFKKKHKDHIISNRWTNYLAIFATFNTVCLSFLIFSGFLDRLWFH

>fig|1664.9.peg.1760

MDVENTVVEILADLTGNDDIKDDMDMDLFETGTLDSMATVQLLLELQGQLDIDVPVSEFDRNEWATPNKIIAKAKELQ

>fig|1664.9.peg.1761

MFKKLWLIFGPVLIAGACLGALLLSPFRFSHPSAKTLSTAATSMSSNVIKGEAIKDAAFNADYIPVIGSSELSRMDPLHPSVMAEKYDWKNKPFLIGAPGTQSLTHFLSLQATGQHLTGKKAVVIISPQWFVPRGVKSEMFDFFYSPLQTSYFLVHAKDSAATRYAAKRILDLTSDTTSGVMRDALKNKALGLPLTTVQKVYINNIKRPGLQHQDQLFSQLFIHGREKELAKGLKVLPDEQDLQTLDQVATTLGRKATAGNPFGIQKKFYEQRILPNQAKLKGEQSNFNYESSPEFSDFQLMLDFFAQHKMAVQFIIPPVNARWASYTGLSQDMLNNFSTKISYQLKSQGFNNIVDMTKDGNEDYFMQDTIHLGWRGWLKVDQHVQPFMATKKAGKVSYKISDDYLDRSWQDVAGNQVQDFEE

>fig|1664.9.peg.1762

MNMASDNWMNATAELLTYYEQEWGQPEHDDQRLFELLCLETYQAGLSWQTVLAKRAAFRTAFYQFEIQKVAQMTTTDIDRLLQNPAIIRNRRKLAATVNNAQVVLAIQQEQESFAHYLWDFVDGQPIVNRPKTWADVPAQSALSVAVSRDMKRRGFQFVGPVTVYSYLQGAGLIDDHPQHLKG

>fig|1664.9.peg.1764

MTKIVIAGSGAMGSRFGYMLQAAGNDVVLLDNWVEHVQAINTKGLTVAIAGQDVQHVQIPAALPTAIKDVQDVIIIFTKSMQLGAMLQSIKPLIGEKTKVVCLLNGLGHPETIERYLPKHNIFVGITLWTAGLTGPGEISLSGSGSVELQNVAPEAKGAAEELVAMLSAAGLHATYSADVLFSIWRKACVNGTLNSLCALLDCNIAQLGRTSQVNSLLASIVAEFSAVAATEAVDLDVPTTIAHIDQLFDPTQAGEHYPSMHQDLIQHHRLTEIDYLNGYVARKGQERGIPTPVNQLITQLVHTKEALVIEK

>fig|1664.9.peg.1768

MLEKTFYKKLLQHSFDFPVAVHFWDGSTDVYGTGEPEIAITFKKLIPIKSLTSNASLALGEAYMAGDIEVTGKGDAPLQKLLTAAYNSSESFMRSHSYIHFLPKQSHSEKASKKDIQSHYDLGNDFYQLWLDKTMTYSCAYFEQPTDDLETAQINKVHHILQKLNPQPNRTLLDIGCGWGTLMLTAAKEYHLKVVGITLSQEQYNFVQQRIDEEGLSNVASVELVDYRELKHAPFDYITSVGMFEHVGQENLAEYFKDVADYLTDDGVALIHGITRQQGGAKNAWINKYIFPGGYVPGLVENTQHIVDAGLQIADMEPLRRHYQKTLEIWDANFNAHRDEIQQQTSLEFVRMWDLYLQACASSFESGNIDVIQYLVSKGASARDLPMTRAYMYNV

>fig|1664.9.peg.1769

MTLYQRFVANEQLRRLVVLMGMIGVILAVRSMMNIILLTFIFTFLVTQLVRHVQRYVRIPPAAVVVPLYILVILLVYLGVTIYVPQIAKQTIKLGESVYNFYQSPSFDANKFMQLISQYMKQFNLTDQLKHGVSTLVNYITGIGTMGVTIVLSFILSFFYTIELDKLPQFGGLFLKSTYGWLFQDIAYFAKKFVNTFGVVIEAQIFIAVVNTVLTTITLTVMKMPNIPSLAIMIFLLSMVPVAGVILSCIPLCIIAFSVGGVQYVIYILVMITVIHALEAYVLNPRFMSSKTQLPIFFTFVVLFVAERFLGTWGLIVGLPIFTFFLDVLGVKTIKTK

>fig|1664.9.peg.1772

MKIVVLAGGRSTERNVSLTSGHKITHALQTKGHDVAFVDLFLGNELKDGATIDSLYSSDPVEKDYDIDDEVLTDDDINKLREDGSTQLFGPNVLEICKTADIVFLALHGGDGENGKVQAVLDVFEIPYTGSDTLAAGITMSKKVSKEILLFNNIPTARFVAAYRHQPMPEMPFEYPVVVKPSNGGSSVGTHIVHNEAELKPAVEDALRFDNEALIEEFIKGREFSLGVINEQPLPAIEIEVNDGWYDFEHKFVTGNTTKFVTPPNNLPDNVHEAMKQMALDAMHALGLTNYARIDFFWSPETGLYVIEGNTLPGMTPLSLIPQESEVLGISYPDLCEMIVNGKLALLNEK

>fig|1664.9.peg.1775

MTKFKIIFKQVFFKNIKSPAYIIMIIMPIILLGVVLGIGKLMDQSTEPAKIAVLSQQPAEQAALQQMRGQDYVIDTQIKTQPQAAQALKQEKIDGYLIIQQGNSQYFERKDSRDFDPSGIQNQLNEMNIVTLARQANVPADQIQKLMTPMTVKSVTVQFEAGQQKIDQSNQKQVGEGISLAITVLMFVFIVNYAGIIAQEIATEKGSRIMEIILSSVSATTQFFAKIAAVLALVLTQIVFYILIGVAGYHYLKQQLPLSAILKQVGPNLWTPPVWYAISFLIVGVLLYTVIAAMLGSVVSRMDQVQQAISPLLILSTISYMCGFILTTRSDIGFLKILSYVPLFSQIMLPVRFASGDLTATAATLGLGLAIITLIGLTYLALIIYRMNILVYSDKGVMRSFMRSFAMLKAERQK

>fig|1664.9.peg.1776

MTNVRMQDDFYTAVNQEWLKTAEIPADKPATGGFVALVDEIDQQLTTDFKQLKQTDNALLQHFLDYYALAADFKTRDELGINPILPFIARVRSINNLTEYNQGLADWVLSGLPTPFGLSVDADMKDTAHHALYAEAGSLILPDKTYYDEGNQSAEQLLAAFTDMMVQLLQKVGFAPVVATKTVKEALAFDRSLAPFVKSAEEAADYAKLYNPKPLSDFIAQQQSIDFKQLFSRILPVQPDKIIVTEPAYFEQLNELVNDDHFDQFKAWLLVMTIRDWSGYLNEEMRQISSIYGRALSGSKEAKPQQKSAYYLALGMFNQVVGDYYGRRYFGATAKADVEAMVKKMISVYQNRLEQNDWLTAETREKAIVKLNHLGIHVGYPEKIAPIFEKLVTVPQADDGTLFENTADFIRTIETENFAKLTQPVDRAKWEMGAATVNAYYSPSKNVIVFPAAILQAPFYGLQQSSSANYGGIGAVIAHEISHAFDNNGAQFDEFGNLHNWWQATDLDHFKTLSQAMIAEFDGLAFADQKVNGKLTVSENIADAGGLSCALEAAKGEDDVDLEAFFVNWATIWRMKATQQYQQLLLNIDVHAPNALRANVQVQNLADFYTTFDVQSADKMYLVPDKRVTIW

>fig|1664.9.peg.1777

MTEKERMVNGQIYRANDAELRADMKRARVLTRLFNQTTEEQQAYRRELTHQLFAQSGADLYIEPPFRTDYGCQTTIGQRVYMNYDCIIIDVAPVTIGDNVFFGPRVGLYTAGHPISAQGRRDELEYGKPITIGNDVWLGGNTVVNPGVTIGNNVVIGSGSVVTKDIPDNVIAVGNPCRVLRPIDQAEEDYWAREKEQYFAEIAND

>fig|1664.9.peg.1778

MKVNHNQFLLWFGAAISIAEIITGTLIAPLGLVKGSLAIIIGHLIGAFLLLLPASFLGGTRHQNAIELTDPIYGKIGFILFAILNVIQLIGWTGIMIANAAQAMQQLKLTHLSYLMNAIIVAGLIIFWLAIKTQFFFKLNNSIVLLLVVACLGLFWQLSQFAGHTTHLNANPLSFGAAIELSIAMALSWLPLIADYTKDSKKPLALSLWSTSGYFLGSLMMFFLGFLTIITTGYTDFNQFLATSRFGFLILFTIIFSTITTTFLDAYSAGVNLKNLFPKIRYSENGLGIVVTLIGLGLIANQQLFKYELFLTLISAAFTPLYTIVFFNYFYRRLPIAVNFSGWLLGAIAYYGLQHFDFPLGTTVLLMICLWLILCCIAQVVGHRKTNR

>fig|1664.9.peg.1779

MTNIAQEILQTYLVAGTQDTGRENFLPILDQALQAGITCFQFRDKGPNSLPTDAMRSDYAKKAQALCRTYHVPFIIDDRLELALDLQADGLHVGQSDQPWPKIEVAKQHGLITGLSCHTAQEILSSHQQPALDYIGVGPIFPTNSKEDAKTPLGLAQLKAFTQLSQLPVVAIGGISLKNCQQVAETGVAGAAVISAITQAKNIPQAIQLLNQPWQSSEGNNHES

>fig|1664.9.peg.1780

MNNFPQALTIAGSDSGGGAGMQADIKTIQERHVFATNVVVAITAQNTIGVQDSFPLPLELVTKQFESLAADFKIRACKTGMLADAEHVAVVAQNLRHFDFGPLTLDPVMIAKGGASLLADSAIDTLRSELLPLATVLTPNLPEAEVLTGQKIRTTADFKIAATQLQKMGAKNIIIKGGHLDNSDQACDYVLYADGSDDWLNTPRIATVRTHGTGDTFSACLTAELAKQVPLKKAIQISKAFIQAAIANPIAVGHGHGPTNHWAYQEVEHD

>fig|1664.9.peg.1781

MSKTLLDQIKQANPIITNVANSVTVDQVANVQNIIGASPIMSSDPEEAPEMVAIAQALSINIGTLSAEPIHQMKTLMAEAYRQAKPVVIDPVAVGSIHYRQKIIDELLALGTPKIIRGNAGEIAYLAGLDWQANGIDAGSGEIDLVKVAQTAAKKQQTTILLSGPTDIITDGHHTTKVANGTPLFQVHVGSGDMLTGLCAAFVAVSPDNPYQAAIDAATTFAVAGQLVAEAMPTPLPGSFYPQLLDCLFNITAADVQTHAQVTEVLTHE

>fig|1664.9.peg.1782

MKNYLQRMGRSLMLPVAVLPAAAILIGIANWVGNGDSNANVFTIFLAAGGGAILNNLPLLFAVGLALGMSEDQDGAAALAGLVAFLVPKTILAPASIQAMQGLSDIAEVNPAFGKIEGNVLIGIIAGLIAAAMYNRFHNVKLPMALSFFSGKRLVPIMAATAMMVVSAILYFVWPAVFTVLVSFGEAISKLGWVGAGLYGFFNRLLIPTGLHHALNSVFWFDVANINDIGNFLGGAKSLAAGTAVIGKTGMYQAGFFPVMMFGLPAGAYAIYKNARPERRKATASLMLAAGFASFFTGVTEPLEFSFMFVAWPLYVLHAVFTGISLAVAAFFHWTAGFAFSAGLVDYVLSLKNPVANQPLMLLVEGLVMAVIYYFGFNFAIKKFNLMTPGREADDAVDEDTAGVETDATDDKFMIQAKRIYAAIGGKDNIKVIDNCTTRLRLQLEDTANVNQPAIKAAGAAGINVLDKTNIQIIIGTEVQFVADALKELYNHNTPIATSAPEPAAAPVEAPVDSDINSGETDVFYAVANGELIDIENVDDPTFAQKMLGDGYAVVPTDGKIVAPVDGTIMTIFPTKHAIGIKTTNGLEALVHMGIDTVELNGAPFEVAVEEGQTIKHGDLLATADLDQIVAAGKQTAMMVIITNMPAVAYMKFNNMGQSVQADTVALKATTK

>fig|1664.9.peg.1783

MFNKLKYILPIVMTAILGLSAHSVFADSNTWDKPVATLGTSLLSTQKSETLSTLQNAANITDSDELTVTGSTLVKYLDQNQQTFNANSNVYSSALIQKNSSNTGINVKIVDFNGKNNITTITANQYKNAALTAGVTNATIYVTSAIPIDGSGALAGVYAAFAENGDTLNQSQVTAAQDEMSTLSDITQANKGTSGYTDSQLNNAVTGAKKDMANKGDNLTVNQITNIVNNQLEQNNLTTIINDNQKTQIINLLVKIQDSGALKSDDFKNQAGKLMDNIQSNAKNVFDKLNTESNRNFIQKLFDSIGNFFKNLFG

>fig|1664.9.peg.1786

MTSAVVVGTQWGDEGKGKITDFLGQNADAIARYQGGDNAGHTIKLGEDTYHLQLIPSGIFNSDKVSVIGNGVVVNPKSLVGELRGLADKNVDTSQLKLSDRAHVILPYHIKLDSLQEAAKGDQKIGTTNRGIGPAYMDKAARSGIRVADLLDKDLFATKLKQNLAEKNALFTKIYEVEALDFDSIFEEYYQYGQELKQYVCDTSVVLNDVLDQKGNVLFEGAQGILLDIDQGTYPFVTSSNPAGGVSTGSGVGASRIDEVVGVAKAYTSRVGDGPFPTELFDETGDFIRQAGHEYGTVTGRPRRIGWFDTVVLRHSKRVAGLTQLCLNCVDVLTGLKTLKICVAYELDGERIDRYPASLSELARCTPIYEELPGWDEDITGVKTLEELPVNARRYVERLQELVDLPLATFAVGPDRDQTNILKAIW

>fig|1664.9.peg.1787

MGKVSRFFGIEEQHSTVQREILAGFTTFISMVYILFVNPSVLGASGMDKGAVFAATALASAIGCFLMGLYAKYPIATAPALGINAFFSYSVVIGMGVSWQTALAGVFVASLIFMVITIFKLREIIIDAIPRDLKLAISAGIGMFVAFIGLHGGGLIVANKSTVVGLGDFSQGTTWLTVFGLIITIILMVKKVPGAIFIGMVLTSILGAVTGLIKLPTHFVAAAPSLKPTFLAGLNHVGDINTVQMWVVVLTFLLVTFFDTAGTLVGLAEQAGFMKDNKMPRVGKALMSDSSAMLAGSLLGTSPVGAFVESSAGIAVGGRTGLTAVVTGILFVFSLFFSPLLTVVTSQVTAPALVIVGVLMAQSLKKIDWEKLEIAIPAFLIVLGMPLTYSISDGIALGFIVYPITMITAGRRKEIHPIMYVLFFVFVIFMYILNVG

>fig|1664.9.peg.1789

MQKKTKNRLINSLLALLLLIGLILVFIGPIRNQMVKSMANRRMNTVTAAQIKKNEHKKANFDFNSVKTLDTKQIVSAAVKGDVIVLGKVAVPSVGIALPVMKGVSEATMAQGGGTMKPDQKMGADNNYALAGHSFTNIFLPLENVKVGDMVYLTDLQNVYSYRVNVKKSIAPEEVKVIDDVPGHKMVTLITCDQTVRRWCVQGELVKTEKANKTNLKVFG

>fig|1664.9.peg.1791

MAKKILVVDDEKPISDIVKFNLTKEGYDVYTAYDGEEALQQVEEVVPDLILLDLMLPKVDGLEVARQVRKSHDMPIIMVTAKDSEIDKVIGLELGADDYVTKPFSNRELVARVKANLRRQGSTSQSDKEANEENHEINIGDLTIHPEAYIVSKRGTKIELTHREFELLHYLAKHIGQVMTREHLLQTVWGYDYFGDVRTVDVTVRRLREKIEDNPSHPEWLVTRRGVGYYLRNPEQE

>fig|1664.9.peg.1792

MNKKIRLFQSIHFKIAIVFVLLLLVTLEVIGASFVKQLEYQNVKDFKQSLQVTPYVQNQLSNELMSNSGNANKNIKDIIGDMGNSAEIQVVDNKGTIRGVTNLNTQSTVGQKTRNPRVKQAIYSGHTEQEVAYSESQGSYYTAIQPLTDPNGDSNTVVGAVYIRASMEEVYKGISSITVIFLTSSLVAGLLGMALSIVISRAITRPIDEMKKQAIQMARGDYSGQVRIYGQDELGQLAIAVNNLSVRVEEAQEASESERRRLDSVMSHMTDGVIATDRRGNITIINETGQDFLNVTSDGVLGQSLLKVLKISEEYTLRDLLETQQVMVLDFSDQQDHDLILHVDFSLIQRETGYITGLVCVLHDVTEQQKNEREQREFVSNVSHELRTPLTSVRSYIEALNDGAWQDPEVAPNFLKVTQEETDRMIRMINDLLSLSRMDQGTAKMNLEFVNLNEFFGYVLDRFDMMIKRDQEHDNNASPRGTGKLPTDDPNKKYSITRDFTKRDLWVEIDTDKFMQVVDNIMNNAIKYSPDGGVITCRLLETHRHVILSISDQGLGIPRKDLGKIFDRFYRVDKARSRKQGGTGLGLAISKEVIEAHNGRVWVDSQEGKGSTFYISLPYEPIDSDGGDWDEI

>fig|1664.9.peg.1793

MKFNSFVMRVILIASIVLSIILSWKIWTNNTHYERRTDSQTTTKAVSRKSDRKISDIFLPTQILWRTNQQSQLVYNSKESLVPKLKEAMKLTKSARFKASKLSEANYYQAITTDDTLTLLYPDNISYAVFKKVFDDSNNQLSRNTTFNRIKITMGTKPQLTLMNDKSHMIYQTTMRQFSVAQVKRILDHSDVHLNVKESLLNHKPFIYVAANQSLLPYSYLVNQQPENYYITTLLDQKNTDNIDAKEQGDNVIYTDGDNNDLYKRLTINHKTGEIAYVKYEDTKSPTGLTKSFETSFEALLKTGNSLSGMRFYSYDEDTNSTTYRTYVEGFPVFYQTNFGSVKVQLLPTGQRVDFSNYSLQVPVPAGDKPVTLPSTQAVLNQMTKNGLLLENIDNIQVGYEWTQETSNDQVIDLNPTYFIQYKGVWRSLSALLAQKEELTEGS

>fig|1664.9.peg.1795

MVEKDEMRVSVLASSSSGNTTFIETPTQKILVDAGMSGKRVQDLMHSIGRDLKEVDSLLVTHEHRDHSSGVGVLARRYGIDVYANQPTWDAMASIIGTVPDEHKHVFEMGKTKTLGDIDVESYGVSHDAAAAQFYQFHHNNRAFAILTDTGYVSDQIRGTIENADAYLVECNHDLEMLRMGKYPWSLKQRILSDTGHLSNEDGASALMAFMGQQTKRVFLGHLSQENNMKELAHLTVASILEQHDYGVGTDFEILDTDPAIADPLFVV

>fig|1664.9.peg.1796

MNQNSGLMKTAIVVVISALIGGGAAYAGFSRLGNSANDVTTATQTPTKAGATKISNIKVNSSSQTTTAFQKVNTSVVSVVNLQKQQQSTGDLSSLFGSGSTDSNSSSSKSSSSDLEESSEGSGLIYEKKDGKAYIVTNNHVVAGSDKIEVILSDGTKLEASLVGTDAVSDLAVLKINGTKVKKTATFGDSDSIKAGESVLAIGSPLGSEYATSVTQGIISAKKRTIDVTDETTGQTTGQATVIQTDTAINPGNSGGPLVNMAGQVIGINSMKLASNGETSVEGMGFAIPSNEVVKIINQLVANGKVVRPALGISVIDLTNISSTQQSSVLKLPSSVNSGVVVASVTSNSVAKKAGISKYDVIVGLGGKTVSSVADLHTILYSYAMNSTTEIQYYHNGSLKKANVKLTQAASSDSTTTTKQTN

>fig|1664.9.peg.1797

MNIKIITVGKLKEKYLKAGIAEYAKRLSKFCKFEIIEVSDEKAPESLSEAEMTTVKDKEGERILAKVKDKEHVIVLAIHGKQRASEEFAKEIQDLATYGTSDITFIIGGSLGTSDAVNKRANDALSFGKLTLPHQLMRLVLTEQIYRAFMINQGSPYHK

>fig|1664.9.peg.1800

MNVLQTEDDFYQLALTYLKKAHQENIRYAELFFDPQTHMTRGVAFEAVVNGFYRACVDARAFNVDAHLIMCFLRDLSAESARQLLEIAKPYQAKILGIGLDSDEHHNPPLKFLREYGDAVAQGYRITMHADIDQVDSIQHIQQALEIIRVERLDHGTNIVENPDLVDWVEQLKIGLTFCPLSNRLVADDMKDQEVLELIDHNVKVSINSDDPAYFGGYINDNYEANTTKNDLTIDQVVQLAKNSFETAWISDFQKQTYLAEIDAYVADYL

>fig|1664.9.peg.1801

MTQLATRDFIDGLPKAELHLHLEGTLEPELKLALAQKMALISAKVRLKKFKQATTSIHSHHFSRFITPQ

>fig|1664.9.peg.1802

MAPMKQSGIQASADRMFKLQESGTTMKREIFAGLTTFVSMAYILFVNPQILGEAGMDKGAVFTATALSAIVGSLLMAFLANYPIAIAPGLGDNAFFTYSVVIAMGISWQTAMAGVVVASLIFMVITLFKVREYIIDAIPHDLKLAMAAGIGLFIAFVGLQGGGLVVASKSTLVQMGSFTVPTTWLTIFGLVVTGVLMAKKVNGAIFIGMIATTILGLVTKLIPLPAQLMSVAPSMKPTFGVGIQHLGDINQPQLWAVVLIFLLVAFFDTAGTLIGLAEQAGFMKNGKMPRIGQALMADSVSMMAGSVMGTTPTAAYVESSAGIAMGGKTGLTSLVVSILFGFSMLFSPLLTVVTSQVTAPVLIIVGVLMASSLSKIDWSRFEVALPSFLTIIAMPLTYNIAYGIAFGFLVYPVTMLAAGRRKEIHPAMYALFVVFVILLYVINILPK

>fig|1664.9.peg.1803

MSDRLTAFKELIQAGGAKRPADLIIKNGQLVNVMTAEIYPAEVAIYQGKIVAVDPDVSDYQGTETRVIDAKQQYIVPGLIDGHIHVECSKLSMTSFAEAVVPHGTTSIISGLDEYISVIGVDGLSEIFKEVDQLPMRVFWGAPFKTPYTIPASTIADNIDSTVQAQLQKRSDVYGVWETVREAVETLDEDTLKTLLSAQDNHVPVWGCAPMATGTKLNEYLMSGVRVDHESYDHQELLEKVRKGINVVIRESSVTHFLAENIRAITETNGQIARHVSFCTDDVNAMDIVNKGHLDHLVRLAIAAGVAPMTAIQMATINSAEAYRIDDQVGLIAPGRNADILLVSDLEAFEITRVLAKGQPVATDGHIDHSIERPVRPASLANTVIREAVQASDFEYHVAADASQVTVQTIASEGPFVRHAKSKTLAVEAGIVQIDPAKDVALISVLERFGKNGNQSLGFTSGWTLKKGAMASTAAPDDNNIIVMGVNPDDMALAVNTLIERDGGQVVVADGQILSFLPLPIAGIVSDVTPAELAVQEEGILKASQAIGSEVVDPMFYMTFLPITAIPDLAITDLGNVDCNELRLFDPILTIQ

>fig|1664.9.peg.1804

MAKAMTNQLKPQNIAKGMFWATMASALWGVSGTILEFISQGQKVPATWFLSVRTLGAGLILLLIGAIKFKQDIFSIFKSKRLTAWLIAYALFGIMGNQYTFFMSIQTGNAAAATILQYLSPLFIVIGTLAFVRKWPNRIDLISFGIALVGVFLSITKGNIHTLTIPFNSLIWGILSGVTAAFYVVLPRPIVNEKSPILVLGWGMFISGILFNINRPFWQAPPHLNGSGIIAILAVILIGTVFAYLCLLHSLNFAPSTVISIVDAVQPVITFILSIIFFHSKFSWVEMSGAILVILAIYILQRYQEADGEPLA

>fig|1664.9.peg.1808

MKTITAHSQHRLTSYQSKVVASTTAGFGLENMDVMFLSFALSSIIAELHLSGTQAGLIATITNIGMLLGGVFFGVLADRVGRIKTFTYTIFIFAFATAAMYFANSLTLIYLCRFLAGIGAGGEYGIGMTVLAESFPKKQLGRVSSYVGVAGQLGAIAASLLAAWVLPQYGWHALFLFGLFPVIITFFIRHHLVESRNFDTDQARPKGQIKALFKTRQLTHQTLALMVMAVVQIAGYFGLMNWLPTMMQQQLHLSVAGSSTWMLATIVGMSAGMLTFGWILDRIGPRVAFSSFLLASAAGVYVLTLPKNMTGLLIVGALVGYFSNGMFAGYGAIVSRLYPAEVRATANNVIMNVGRCVGGFSSVIIGWLLDHYSILTVMLFISTLYLISFVVMLTVQNLKAERYFNHL

>fig|1664.9.peg.1809

MATKKRHHFTFPSAYTVILLVLLLVMILTYFMPSGKYASLQYNTQSNNFTITSPTGRESTAPGTQATLKKYHVNTSLAKFKDGSVYKPVAIPNTYQHLKKANTGLFGAVKQFLNAPIQGLAETIDIITFVLILGGIVGVVNKTGAFAAGMAALSKKLKGREAWLIVIVTSLIALGGTTFGLAEETIAFYPILVPIFMAAGYDALVAIAAIYLGSSIGTMASTVNPFSVVIASNTAGISFTEGMGLRLVMLVIGTAMCIFYTIRYAEKVKKDPEKSLIYSQKAELEAHFLGDQENNVVPKFDFRKKLMLIIFAAGFVIMVFGVKEWGWLFNEISALFLAITFILAFISGLSEKDFVGEFVAGASDLLGVALVIALARGVTIIMEQAQISDTLLFWLSNGVSHMSGVIFSTVMFFVFILLGFFIPSSSGLAVLSMPVMAPLADVVGVHRDVIISAYNWGQGIMSFITPTGLVLASLAMVHVTFDKWLKFVLPLMLAIGLLAMILLGISVYL

>fig|1664.9.peg.1810

MSNKAKEFWKSTLQIVLLAAVMVGISQLLLTFVISNEQVFGPSMQPNFTQNDRVIALRHAKLKRGDVVILKAPDAKGEFYIKRIVGMPGDTVRFDRDQLYINGKKVSEPYLKAYKADFSTYLAGSDYFTLNPKTNGPTFDLKDVLGQKKVPAHHYFVMGDNRTVSKDSRYKEVGFISDDASAYNGIQGVVKLRYWPITKFKIY

>fig|1664.9.peg.1812

MPIIEANFTPVLLGSDFNVYGMARSFYELTGKPVQAYAGAELAPTRYTKIVDVDITPGFSEDPVFIEKMRDLILKYQTHTEPVILIGCGDGYAELIAKHKTELEAAFVCPYIDYDLLKRLNNKENFYQVCEEFDLPYPKTKTITQQMVLDGQVDQPFGYPVALKPANSVEWLDVHFEGRKKAFRIQSQAEFDMIIKKIYDNGYTSDLILQDFIPGDDSRMRVLNAYVDQNHQVKMMCLGHPLLEDPAPSAIGNYVAIMPEYNETIYKQIQTFLEAIKFTGYANFDMKYDERDQTYKLFEINLRQGRSSFFVTLNGYNLAQWVVKDYVTGELKDQATVYANKDNDQAALWLGVSKGVFEKYARDNEDKTKALALIKAGRYGTTFQYAKDNNPKRWVLTQWMLHNYRKNFARYFKENKG

>fig|1664.9.peg.1813

MKQFFTVLGGMGTAATESYIRLLNQRTPTTKDQDFLNYIVVNHATIPDRSTYLMDHSQPSPEPDLLEDIQQQSLLKPAFFVIACNTAHYFYDDLQAAASAPIVHMPRETVKSIQTTYPDAKRIGILGTKGTVTDGIYDEALLAEGYEVVKPSLDLQERTMDLIFNDIKGQSQMKPEKYHAILAEMQTQCDAIILGCTELSLAQEWAPDHDFPVVDSQSVLVDRSIELGLKLREKRV

>fig|1664.9.peg.1814

MANLVIVRHGESVANQLKTYTGWSDVALTSKGRQQAHQVGRQIKDAGFQFSHVHTSLLKRAILTSYIVLEELDQLALPMTKSWRLNERHYGALRGLNKDTTRTIFGVHQVARWRRSYTAVPPLLIRSSTARRYRLVPRKSRPRGESLAQATQRLLPYWQDQVVPGLLAGQDQLIVAHGSTLRALIKVIEQISDQAIDGVEVANGEALYYQFGTDLTVLKKGRLALGSETDEEKEN

>fig|1664.9.peg.1815

MKRKKINIALTICVLVLGVSLTFYFRRQFVRQGQALANTQMQLKLTKKQLDIQRAKVNKNDIDDRVVNELTTPEQAYLKQELLDNNFSGTILIVRHNHVIYQGGRGYANSEQFTTPKTTYQIGSVQKSLTAALVMKAVENGQLKLTDHLSRFYPQIPKANQITLQMMLDMRSGLFETSAPKVVQTDAQRLQFAIKHLQVKKIGQHAYSPVNYVLLAGLLEQATHKSYNDLMKQQIITPLGVQAFNFMPNFSDPQHATAYAYQKNGAYGAEIEDPAYIYTRELGTGNLYATASSLYQVEEAIVQGKVFKSKYLQQLRHTNNGQYSGGVYNYRDYFTSHGIEAGFESHVQMSQNGESGIILLSNQYKSTPSLVTMSKQIYQQLLV

>fig|1664.9.peg.1816

MRKILEYSLAGVLSFLFSRAFIHRIVLPTLWGYPRLTRVMARFPKTTVLLILFLTLALWLGWIQWDLKRLSPIYGYLIYSVYLLLLFIVLFTKASVYHAVSLNPFDFISHDRRTILEALLNVIYFIPLGGLYSLKADFTETNIIALLTILGIEILQYVFYLGTFAISDIILNWVGCLIGFTLFTIGARHFKQTIKTNA

>fig|1664.9.peg.1818

MQITRHDQPLETIGTPPKVGEQLPAFELVDADHQTRTTQQLLGKRTLISIVPDINTRVCSIQTKHFNQAIDQFTDINFYTVSTNTVEQQQAWCAAEGVQLMQLLSDEALSFGKALNLYVPSNNTLQRSIMIVEADGTISYEELIVEQTDEPNYAAALEFLKSAH

>fig|1664.9.peg.1821

MVEKQLLVITGATGSGKTTVSHWLTEMYQIPQVVTHTTRPKRHNEVDGVDYYFETESSLAQKHLIESVTYSGYHYGSSHEALELAWAKSPLISLVVDTKGAQTYLETFGKQVTVLFLTVSEATALISRLEKRNDSPEAIQKRLASAEFKRDLMVSGPLHDQAQIIVNDDWQETQKTLTTIIEQLRQ

>fig|1664.9.peg.1823

MSEMTTALAVLKANGFKITKQRREMLTYLAQYENHYIPVTQIDDHMRAIFPGMSHDTIYRNIKDFEQLGLVEQQTEGEQAHVKYQCDFEHRHHHHFICRNCGKVIELKMCPLDFFSDQLPNCQVEGHQFELYGLCEDCQENE

>fig|1664.9.peg.1834

MQETKMAQKRVLITGVSNRQTNFDYTMRELGELARADNLEVVGEVRQNIDQASHRTYFGKGKVQEIKAEAAQADADIIVVNDELSPSQIRNLEKELEMSILDRTQLILEIFASRAKTKEAQLQVAIAEAKYQLPRLHPSENKLDQQGGGGSTNRGAGETQLELDRRLIEKRITKLKGELAQIDQQQEVQRRQRTKNAVPVVALVGYTNAGKSTTMNRILQRLNPDTPEKQVFEKDMLFATLDTSVREIVLPNKQKFLLSDTVGFVTKLPHHLIQAFKSTLAEAAGADLLIHVVDYSDPNYLDMMKTTQETLDSLGVKDIPVIEAYNKADLKPDTRYPEVDGTQIVYSARDEVAIDALIALINKQLFEQYPTLDFLIPFTEGQAVAYLSERANILEQSYEEEGTKIRAQISPDLLGAVQKYVQE

>fig|1664.9.peg.1835

MKTNFDFLMPSVNFFGPGVIEKIGDRTKMLNITKPLIVTDTFLEGVPDGPVAQTLASLDQAGVQYSIFNGVEPNPKDRNIQAGKAQYLADGCDGLITVGGGSAHDCGKGIGILLTNGDDITKLAGIETLENALPPLLAVNTTAGTGSELTRHCVITNEETHLKFVVVSWRNIPLVSFNDPMLMLDVPAKLTAATGMDAFVQAIEPYISTNRNAITDGQCLQAIKLIEESLREAVANGHNIEARTKMCEAEMLAGMAFNNADLGYVHAMAHQLGGQYDAPHGVCCALLLPIVEEFNIVACPDRFAELARAMGENTDGLSTRDAAELAIKGMRQMAADVGIPTSIKAIGAKPADFEMMAENALKDGNAFSNPRKATKAEIVALFQKAYDAE

>fig|1664.9.peg.1836

MANLALIDVGGTTIKFALWDGTAHKLTKQGMVATPKSLNAYYAALTKIVRTYQLSDQIVGVGMSTPGAVNKATGIIEGASALPYIHNFEIQSALEMRFGLPVIMENEINCAALAELGSGSAEGLQNVLYLAIDSDVGGAVIIDGHIQHGSHLFSGEFGAMLMSDGHPLSEVGTITYLTQQYNQQAHAELTGTEIFALARQGNSLAHQVTTAFYQYLAQAIYNLQYCFDPEAIILGGGLEKIDFLVPHIEIERRKLLTQVQLAPFETPLLASQYQDDANLMGALVDFQQCYPQKML

>fig|1664.9.peg.1837

MIDIKTQTPPQELNRTVGFTAALSTVMGTVIGAGVFFKASSVTEVTGSTGLTMLAWLLGGIITICAGLTAAELAAAIPETGGMMRYIEKTYGSVAAFLLGWAQTTVYFPANIAALSIIFATQCLNLFGWAPSWQIPVAVIVATSLTIMNFFGSRVGGFVQSFTTVFKLIPLAIIIIFGLINPGTNASHVSLFTVAGTGTSNNVLSALGNGVLATLFAYDGWIHVGNIAGEMRHPEKDLPKSILLGLFGTMVVYLLVNAVFLLVLPISQIAGNENAAAEVAGHLFGGFGGKLVTIGILVSVYGAINGYTMTGMRVPYAMATEQHLPFSKQLSRLSKAGIPILCGILQLVIAIGMIFVGGFNTLTDMLIFVIWIFYVMTFAAVIILRKREPEMKRPYKAVLYPVVPLIAILGGTFIVINTLFTQTILALIGLGITLIGLPIYYYLEKKYHQA

>fig|1664.9.peg.1838

MDEIEPKKKYITWQVLALMDFVTVIGFDDIIYNFQNQGMAVVFSWIVLLFAYVLPYELMVGHLGSAFSKDGGGLSSWMRHTSGDVWGYLTAWCYWVASLPYLVDVANSMVVSFFWIIMGNGNIEDKLSRATFALLTAAITILFIFFQHKFTASLQILSIVGGGAMFIMTILFVLMTFAGLFNGAPIATQPMNWHTFIPKFDLHYFSTIGLLIFAMNGAELVAPYVSEMKDGKKDFPKAMMALALMTAFLTIFGSFAIGVFFDANHLPNDLKMNGSYYAFLALGQQFHMGKVLLYIFAVTQAIYMGAQLAMLLDGGTRIFLSDTAKKFLPKALTKTNKQGLPINGYWLTTGICTFVLASSVFLPAINDIFNWLLNINGIVSPFVTCFIFVAFLRVRFNSNKYPSEYTYIKNKPWAIIVGFWCLLITALGTIFSIFPQDATPGTSKYTHELILNVVVSVSLMGLGVILPLIAKYQRKKTA

>fig|1664.9.peg.1839

MDTPTVVSVKDVQKTYGKANEKQYTALKGVTFDVQRGEFVGIMGASGSGKTTLLNILSTLDKPTAGEVKINDQEISRLKGNALADFRAQEIGFMFQDFNLLENLTAYENMALPLTLQNVTGKKVREKITTIAKTLSIDAILNKYPTELSGGQKQRVAAARALVHNPAILFGDEPTGALDSKSARELLDTMAQLNEEQRVSILLVTHDPFSASYCQRILFIKDGQIGQELVRGDKTREAFYQEVLDTLGTFNH

>fig|1664.9.peg.1840

MLWKLSLTGIKGRFKDYLVLFSGLIMSSAIFYMFEALATNKDFVTSNSPIGMAAIVFQIGSVLLGIITLVYIIYANSFLMSMRRRDYGMFMMLGAKGRKIAQLIAAETLVIGTAATLIGTAIGIGLTQVVSQFLLSRLDMHVKHFSPLYLPAVITTLILFIVLFIIAAVVNQYQLLKTPVLQLLHDDQQPNRIKENKVTRLIQIVLGLVFLAVGYYMMIAIAKFKIMGLVIALITIVLGTYFLFNAFFGWLLMLLKRNEKLRNKGLNNFTLAQLSFRIKDYTKMLALVAMLFALALGAITVGIGYQNEIPKIADSASAYDVVLNQPTQKALTLTKNLKGIKQTATYQVKEDGKIVYYRIEQFKQTPLLTIEYGKTGSVSSETKVRKVPLSELQKGDNYLFRSIQVPSQTAKAVKLISNEAFAQIVAPTKVVKTYDVQDFSANLPVIKKPYQEQLKANPELKANPESDSSKYASYKMANGIFSGLEFMGFFLGIAFLAMLASCLMFKILSGAASDVKRYTMLAKIGVRQGLLKQSIAKEIGALFLLPGIVGVVHVLFGLQMFKELMIDPYNQIWIPFSIFIVLYAVYYVVTTWLYRGIVLKETKFGE

>fig|1664.9.peg.1842

MKKADKTTVTIRTIAQLANVSHMTVSRALNDSELVKPATKEKILAIAKEIGYVPNINAKSLVTNRSYMIGIFFTNLETGTSNSFMTDVVAQAQAVLPKSYSLSINSVAKAMAGQYISINNYDGIIVISQSKSDYDFIEYVHNLGLPLVVLNRVIERNDINNYAIGDRLGGELATNYAIQMGHRKLALIRGVDSFESSVQRTNGFMQAVEANGIAVDPTLIKVGDYRPESGHELMREILASGNIPSCVICENDDMAVGAISACVELGYQIPRDISFIGFDDMAYAKFITPSLTTIKKPTAQIIEMGVAKLMAIVEGEQSDVEQKIVDPEMVIRQSVVNLYQH

>fig|1664.9.peg.1844

MKQVPETKIKLTDVKELINKGYNLLGELREEADAPVAKAEFLTEEITTVYGEEAARKFYNPENFKREGSMPKVVLKTLFGEDGVQTIDGKKHHQRKNYFMDLMTPERMEDYRAILDQNLATELDQQHGTFELFDLSKRVLFNSICEWAGINLAQYDPKEIDKLASNQISMISGAITSPTNHLKGVKDRNESEGWAQSLIKEARKNPVPGKENLALYTFAQAEDLEGELLPVEVAAVELLNIIRPTVALTVWMALMGHALFSKTNLYDQLKEDFDTLQDSFIQEMRRYYPFFPMLPAIALRDVEIDGYEIPKDSWVVLDIYGTNHDARTIDHPEKFDIKRYIGKTKEISYEEEYEMIAQGGGEFRNMHRCAGEWITLHSMRVFSDQLVNKYNFSIPEQDWTVPMNQFPTYPNSKALLFKE

>fig|1664.9.peg.1845

MSKLKRFLPLILIFGSLLTLLGIKKIKEAHQPDLPTVGILQPLSHPALDEIHRGIVAGLASAGYHNHQNIKIDFQNAQNDQSNLRLMSNRFVTEKAKVTIGIATPAAQSLANATKQLPIILGAITDPASANLVKNNQHPGANITGVSDQAPIQKQLDLMQAIMPQLKTVGILYSSSDNSATSQMHLFKQLAKQKGLQVIDASLSSVNDLEQVSQNLAPKVDAIYVPTDNTIASAMGTLVHVTDKTKTPVFPSAETMVKDGGLATVGLSQYDLGVETGKMAAAVLSGRSKPASTPIRYLRKGHLVLNLKKAKDLHITIPQNLVKTAKEKGAIIK

>fig|1664.9.peg.1846

MSLIISTIDQGLLWSLLSLGVFITFRILKFPDMTVEGSFPLGAALSAVMLQQHNIALSLLVAFVGGCLAGLVTAILYNYFHVQSLLAGILTLTGLYSVNLRILGQANLAIGDQHNLYQLPFLQQFTVDQRLLILNLIIIVVAMLALALLLYTEFGQALIAAGDNPTMALSQGIAVKRTKTIGIVLSNGLIALAGGLVAQYNGFADVNMGIGVMVIGLSAIIIGERLLVGHHFISKLISIILGSIVYRLILLLVLQLGFNANDFKLFSAIILAIFIIAPQQKKGMTTK

>fig|1664.9.peg.1847

MTLNQISYQISDDNDQKQLLDQLSLDINPGEFVVLLGANGAGKSTFFNTVAGDLTPTSGQIIFNGQEITKQSAQQRTRLISRVYQDPKLGTAADMTVAENILLAMRRGQRRRLLPRRLKQQLPQIKALAKTLPNHLEQQLNRPVSTLSGGQRQTLNFLMATIQKPELLLLDEHTSALDAVSSQALMTFTDQTIQDNQLTCLMITHDLEDALRYGNRLLVLKAGQLIADFNAVEKQQLTTEKLLNYLNA

>fig|1664.9.peg.1848

MTGTNTETQKTIPASVAMLRVLESWGVDHVFGYPGGSFNSTMRALTVEQDRLKYIQVRHEQVGALAAAADAKLTGKIGVAFGSAGPGATNLLTGLYDAKEDHAPVLALVGQVPSTNMNYNYFQEFNENPMFDDVSVYNRLVMTPESLPYVVDKAIREAYKHKGVAVVIIPNNFGYVEIPDTPYSSSSPTDRKQVAGPVATDEEVDQFLAMVKDAKRPVIHVGRGIKDGGDKLVELSKKLQIPIVMTGLAKGLVPDSYEGNLGMANRAASKAADEIMAVSDLVIAIGADFPFANLIYRTHDFKFVQIDNDEAQFGRHHFLDFGIWSDATTFVEKVVARSQAAAPSPFFKAAVADMKNWQAYLDALTNRPSDPLEFEPIYKEINRIADPDAVFSIDVGDNIINSFRHLHLTPKNKWVISALFASMGSGVPGALAGQLSYPDRQVFNIAGDGAFSMVMQDLITEKKYQLPIINIITSNASLNFIKSEQDDLPMDYSGIFIEDQDFAMIAQAMGVESITVRSSKELPAAFDKALEVTKAGRPFLIDAKITDKRSIPVEELELTIKDGKLTESISASYDASRPTGKEYSVADFFAEYDGQSLKTLPELFAENGVKL

>fig|1664.9.peg.1850

MAKKQTAGRDLLGDFAPKFAELNDDVLFGQVWSRESELPAHQRSLITISALISGGNFEQLPAHLKIGKENGITEAEIVEVITHLAFYVGWPKAWSAMNAAKTIYRA

>fig|1664.9.peg.1852

MAEKHYKIGEFATLVGLSTYTLRYYEEQGLIKPQRDAAGLRYYTLEDVKWVGFILHLKGKGMRITELQNYVELRAQGDPTIEARKALLQQVKARAEAEVAELQANLVIMGRKIDWYDGKLDQSIGQLESFETYLQRFES

>fig|1664.9.peg.1853

MSKILVLGAAGQVAQLVIQDLLAAKQHELVLYLRNAERLAALKTNPHVTIIEGDVLDQARLTAALKGIDIVYANLGGEFEPLMKTVVKAMTATGVKRLIYISGLGLYHEVPGEFGAWNERAVGHDVMEDTRRAAAVVEQSPLDYTILRCAYMTDEPIVDYELTEKGTPFKGTIISRQSIAKVVVDLIDQPTLGIRASWGINQPNTDGDRPVY

>fig|1664.9.peg.1858

MMFKFMKKAPAGQLTAPVTGHYLPIEQVTDEVFASKAVGDGFAIVPDEMTNTINAPVDGVVSSIFPTKHAILIKTKQGVEVLIHMGIDTVELQGAPFEMQVEADFKVKAGEPLVTIDWAQLKAADKANTIMVVSSNATFTPAEPLPAAVTTDSVIGQFDLSAE

>fig|1664.9.peg.1859

MKWWQKATVYQIYPRSFQDSNGDGIGDINGIIQRLDYLQDLGIELIWLTPMYVSPGRDNGYDIADYYQIDPIFGDLADFERLLKEAHQRGIKIMMDMVVNHTSDQHRWFQESLKGKDNPYHDYYLWRDPVDGHEPNNWLSKFSGSAWEYVPALNQYYLHLYEKRMPDLNWRNPQLREEIYQMMQFWADLGIDGLRLDVINNISKDKDFPNDTFETPSDDGRNFYTNGPHVHEYIHEMYERVFGPNNFVTVGELSSTPVSEAIRYTNPEREELSMAFTFHHVKVDYTGGKKWTLGQYRLSDLKRILSQWQTEMAAGGGWNALFWSNHDQPRAISRFLDDGQYRDQSAKLLALVEFGLQGTPYIYQGEEIAMKNANFTSIDQYQDAESINAYHQMLADGISEDLAIKILQQKSRENCRIPMQWDMTANHGFTTGMPWLQPVVADDYSVATKAQAPDSVFAFYQQLVALRKTEPVLIEGQYQLLAPEDEAVYTYQRQLGDKKVRVIANFTDQTQQRANAAVKAVMMSNYPDQQLTTDQITLRPYEAVMLEIE

>fig|1664.9.peg.1860

MAQKDYSQLAKAIVAGVGGKENIDSLIHCITRLRFYLKDESKAQTETIKALDGVINVQKASGQYQVVIGNQVTAVYDAVIAEIGADFADEDETAQVVAKTKRKQNLTPWGHVKEAFSQLIGVITGAMSPIVGILAAGGILKGVLAMLTMPQLGALVSQKSEFYIILSAMGDSVFYFLPMLVGFTAAKKLKGDPVLTAVIGGVLVHPSLVALNGKSLLEIGSLNFLMVNYTYSIFPMILAAWLVARLTKWVKNWLPGYLQIIFTPLIVIAVVAAITLYITGPAIIWLSNGLAFGIQFLLLKSGWLSGLLIGGFYQVLVIFGLHWGILPIIANDVATSGHSYLNAILSTTMVAQGAAVLAVAIKTRKTALKELSFAGAISAFCGVTEPAIYGINLKYKRVFISGLIGSAAGGFVSGLLHGNMFGFAGSWIGFASFLDPKHPADLSNLWIFIAASAVATIVPFIVTYVWGYNDQMQEGQGMAKPQKPGTTKKVTA

>fig|1664.9.peg.1861

MSKKYEIIYRELVHKITNHVYPANSFLPSENELATAYGTSRETVRKALASLLENGFIHKIKGKGSLVLDFDKYAFPISGLTSYRELDSLFEMHTQTTVLQLQKATVPQAPFHLDPTEILPATYIQRLRTVDGEPIVIDNDYVLTSVVPEISRAVAQDSLYHYFEQELGLTIGYGNKEFTVIPATEEQCAILKLAPNSAVVSVKGITHLQDNTLIQFTESIHRADKFKFIEHARRQSI

>fig|1664.9.peg.1865

MAILIALIPALAWGSIGLVSGKLGGNAYQQTFGMTIGALVFGIGTFLVIQPKIDLFIMVIGVISGLFWSIGQGQQFQSMQAMGVSKTVPLSTGMQLIVNTLAGALLFHEWKTTHDYIFGITALVILIAGATLTSMKDPKSSLAAKEDTQFARGFRALILSTIGYGGYTIIVNWSGISATTIVLPQAIGMFIGAAIFAGVGIGKAVFDKKTGLNIITGLLWGLGNLFMLISMSDIGLAISYSLSQCGIIISTIGSIYLLGERKTKKEMVYVTLGCLLVIVGGVTLGMMK

>fig|1664.9.peg.1867

MKVLLYFESQKLLAKSGIGRALDHQKRALTAMGIDYTLDPNDTDYDILHINTYGLNSRRMIKKARKAGKKVIYHAHSTEEDFRNSFVGSNQLAPFVKHRLVSLYSKADHLITPTLYSKALLQGYGIQVPIAPISNGIDLAKYQATPDKEQAFRAYFNLKPDQKVIICVGLFFQRKGLLDFVEITKQLPEYTFIWFGDVPMYSIPRNIRLIVKKDHPANVRFPGYIKGDIIEGAYANADLFFFPSYEETEGIVVLEALASHQKVLVRDIPVYHGWLKDQQNCYMGTDNTEFKRLIQDIVEQRVPDLTEAGYQTAESKHITAIGHELAAVYQHVLTPDESNPTYTQIMQHTLKL

>fig|1664.9.peg.1868

MKLTKIKQFMASKLGFLSLLVFLFWVKTLFGYFTDFNLAASDPLQMAILIFNPLATTLLVLGILLYIKQPIISYWTGLVIYTANTALLYFNVIYYRQFTDYMTINTILGYSKVSAGLSKSSLALMNWHDALYWLDIIILAVLILLKVIKIDHRQFPKRWAFSITSLAVLLFGVNLGLSEISRPQILSRTFDRNYIVKYLGIDTFTVYDAVKTTQNNQVRAQAESYDLDSVLDFTKQHYAAPNADYFGAAKGKNVIVIHLESFQQFLIDFKFEGQEVTPFLNSLYHNQNTLAFSNFFNQVGQGKTSDAENMLETGVYGLPQGSVFTTLGSDNTFQAAPSILKQNGDYTSAVSHGNVGTFWNRNNVYKNFGYNYFFDSSYFNTSPDNVLQYGLKDKLLFGQSIKYLEQLQQPFYSKFITVTNHFPFPLPKTDGDFPRATTENSAVNNYFATAHYLDQSLAEFFNYLKSSGLYDKSMIVLYGDHYGLSNSDNKALSGVLGRDYDTWTDNDNAQLQRVPYMIHMPGLKGGIQTQYGGEIDALPTMLHLLGINSQNYVQFGTDLLSKQHDQVVAFRNHNFVTPEYTVLDGTIYQNQDGTEVTQPTAKLQKQVAKWQKQVNHELSLSDSVNEKNLLRFYTPTGFKPVDPADYNYQTGYQQLVDIENKLGTKSTSVLSQNKGKSTVPLFKTDAPELADDQSSLTEVPKSKALKDSAATSSSSSSESGQSTSE

>fig|1664.9.peg.1871

MAKQHTIHSVAKSEGTMGDWDLFATWIGANANNGTWYIGGIIAATGLIQGATLLIAIGCLSYVLLALASYMGYQTGVGAMGLTRASFGVKGSVLPSLINVVQFIGWAAVNTYIAATSVSFILKEIMGWSGNDAATNRLSLVVGIIIMSILHLISISLGEKSVRWIERIGIVLVIILVTWESIIVFKMVPFKDLVAWRPAAKFRLEAGRAIDILAAFNLAWVTAAADFSRFAKRKKAATVYSFLGANIGLFWFAFIGLTATIATAITLNHFDPNNADPSTIAAKLGLGVLALLVIVITSTTANAVNLMAAGSALTNIFPKLKLTPALWCVTLLATVVTFIPVYVASFLTTFETFLDSIGMFLGPEIAIFLVDYFWIRRRHYQVDALNTIAGPYWYTKGYHLTALFSWVIGVISYLILNQFAIVHAYTGATFIAMLITALFYGVAVQLQTKKVI

>fig|1664.9.peg.1872

MLIQNVHIENQTALKSIRIENGLITAIESHLEPQQNEKIIDGQQQLALPPFVDPHVHLDSTLTAGQPEWNESGTLFDGIRIWSERKKTLTHQDVKDRALKALKMQASHGVQFVRSHVDVTDPELVALKALIEAREEVKDWMTLQLVAFPQEGILSFPNGKALMTKAAQLGVDAIGAIPHFEFNREYAVESLKFAFELAQEYDLLFDAHTDEIDDPNSRSLETVATLALETGMKDKVTASHTTAMGCYNDAYMYKLMRLLKMADLNFIANPLVNLFLGGRFDTYPKRRGLTRVKELTANGLNVAFGEDDIQDPWYPMGDGNMLDVLHAGLHATQMMGYTEIMNAYRFITHNGARAMHVTDQYGLEVGKPAHLILMASDNFYNAINQRAAVTLSIHAGRIISETQPATTQLFI

>fig|1664.9.peg.1873

MKLIYQWMLAFFGVIMTTIVIVGIAFTQYSTKTAYNNTWDQLEGYASVIEREAFKQGSQATLSKQFIDDSQAILSQQRVSFNFFNAQNEMVYPAPNSNVSVKKKYWKYLKKGQVQRLSMSKDSRQFNNDQDQLVVFKPVFYNDKLIYVIGVGSSIKNVKSGLVTMRKNLLIAFLLSTIGGILLSYVLARYQIYRISRLRNATHLVAEGDFDVHIDNPGKDELDDLATDFNEMVHSLNESEAEIKRQEDRRRQFMADAAHEMRTPLTTVKGLLEGLAYDAIPEEMREKSISLMQNETNRLIRLVNENLDYEKIRTNQIKLNQQTFNAVEALQNIQEQLQKKAADAGDTIVLEAQEEVAIYADYDRFVQVMFNIMQNAIQFTNNGQITVTAEAGFHETILTVSDTGIGMTPEQVKNIWERYYKADPSRKNTKYGESGLGLAIVHQLVQLHHGTIEVQSEPDQGTTFTLTFPDETVTTDAN

>fig|1664.9.peg.1874

MKILMVEDNKSVSEMMGMFFQKEAWDAHFAYDGNEAVEQFSVDPDSWDIITLDLNLPGMDGMQVAQKIREQSKVVPIIMLTARDSESDQVLGLELGADDYVTKPFSPITLIARIKALHRRAELVDEQVGAEDAQVEEVDNSDYDVQTDHFKMSSKTREAYLMDEPILDLTPKEFDLLKTLAKNPRQVFSREQLLELVWDYQYFGDERTVDAHIKKLRQKIEKVGPQVIQTVWGVGYKFDDSAVNKH

>fig|1664.9.peg.1876

MTYKGLVFFDLDGTLYNEHSRVDPAVAIAVKQLRANHYLPIISTGRSPLEIQEALAITGIDSFIALNGSYIQFEGQPVYQGTIPTKTIEQVVTIAQEAGEAVAFYDDQAIRITSVTQAAKDAYAEVNAPVPSVDPEYYLNHPIYMLLILTNDNDAAYATDDLTFYRNTPYSIDTVEKNGSKQTGIKRLLASQGLEDMPTYAFGDGRNDIPMLSYVDYPTAMGNGIPEAKAQAKYITSANVEGGIIEGLRHWKLI

>fig|1664.9.peg.1877

MSKHSLFAKKKIDASQFKNGLLERNLTAVGLTAMGVGAVVGAGIFITPGIIAAKYAGPGAMLSYLLAAVVCALAALCYSEFASTIPLAGSAYTYVYTVFGELVAWILGWALVSEYLFAVASVAVSWSAYFQNLMAGFELHLPTALSAAWEPGKPAGVNLIAGIVVLIVGFLLSNGMRESTRINTIMVGVKIAVIVIFLGVAIFYVKPANFQPFLPYGASGVLSGAALAFYAYIGFDAVSTASEEVINPQRDMPIGIIASLLIASLLYAAVATVLVGVVHYTKLNVGDPVAFALQLMHQNWVAGIISLGAVAGMTTVLLVMTYGGTRLIFAMSRDGLLPAVFAKINPKTHVPVANTWIFAVVTSIVAALVPLDKIAELVNIGTLFAFATVSLGVIFLRRIPNYNELPQGFKVPLYPIVPILSCILCVLLMTQLQMTTWIVFVIWLVIGLVIYFSYSYHHSQQRM

>fig|1664.9.peg.1878

MNSDLKAIAILAYQSLIAEVQLAPKPGLVDPESNGAHDDMDYPLFLKSIAALKAYLLAYIEAGYQSTSDQQLFLTLRQIGQDAEKQMLLATSQINTHKGANFSYAFILGAIGRAGQTLTLSKLVEQNFKPVFEIIRNMTKGLVSRDFSQITDKTQLSYGEQLYVEYGFTGIRGEAEAGYPSLEYIALPTLQKYRMLPEDDRHLLLMLTLMTEVQDMNLVHRGGIDGWQMVQKVAAEILQETDDMVLVRRKLRQFDAQLIEAHLSPGGTADLLSLGIFLERLKYSMATQKA

>fig|1664.9.peg.1879

MDKIVNAVAKNLDLSQNRPIKELIYEALRKTVILGDIPAGVRINEKYFSEMMNISRTPIRYALKRLADEDLVIRKTGIGVIVKGISVKDAYEIYAIRKELDALATRQAMRLMTTAEFEQLNQLLLETRRLNEAGDVTAVLQKFSEFNQFIYDKSQMYRLKSIVVELQEYLVYFRDICIRSTDRCDLALAEHWLIYRGMLNQDVNQINLITHEHLNHSLDFIVKEMRHRHLE

>fig|1664.9.peg.1880

MVNRKVEITETVLRDGQQSLIATRMPIADMLPILKTMDQVGYHALEVWGGATFDACIRYLNEDPWERLRQIRKQVPHTKLQMLLRGQNILGYKNYADDVVESFIERSLANGIDIIRLFDALNDTRNLETALKATKKYGGHAQMTIAYTTSAFHTVPYYVELAVKMANMGADSICIKDMAGILTPQTAFDLVAKIKAAIDLPLEVHTHATSGIAEMTYLKAVEAGADIIDTAISPFAGGTSQPATESMLISLQNLGYEVDLDQTKLSEIADYFAPIRDRFREEGILNPKVKDIQPKALIYQVPGGMLSNLLAQLQAQNMADAYQEVLEEIPKVRADLGYPPLVTPLSQMVGTQALMNIVSGQRYQVVPNEIKDYVKGLYGHPPVAIKPEMVTKIIGDQQPITQRPADLLAPQLPEFEKEIQQYAHNIEDVLMYALFPEQARDFLGRREDRFYDVPVQKIDIQFETEF

>fig|1664.9.peg.1881

MSEFRQLTGPAIDLGQMLTAREQRVVNQTRLLKKYPEATLLCGTMRIPGPIKTGPKLKGLVQQFCETIQKQYKDKLVDQLIKLEAITGPEFYFVLNIAPKQIKQEMVCFEQGNPLGAIWDLDVLYWESEQLVQVSRVALGAPRRTCLVCGQDAKNCSRSRQHGLLEVQLKIEQIIEEGWDARW

>fig|1664.9.peg.1882

MTINQVGRDIPDQYASQYGVYAGELTNIKDYQRATKKVHPVKPRDSKLLASIHDAIIQTGLKDGMTISFHHHFREGDYIMNMVLAEIAKMGIKDLAIAPSSIANVHEPLIDYIKQGVVTHVTSSGLRDKVGAAISEGILADPVIIRSHGGRARAIERGDIHIDVAFLGAPSADEYGNANGVKGKTTCGSLGYAMVDAQYADQVVIITDSLMPYPNTPISIPQTLVDYVVEVDAIGDPEGIAKGATRFTKNPKELLIAEYAAKMITESTYFKDGFSFQTGTGGAALAVTRFLKQAMIDQNIKASFVLGGITNAMAELLEDGLVEKVIDVQDFDHPSAISLGKNSEHYEIDAAMYASPLSKGSVINQLDIAILSALEIDTQFNVNVITGSDGVLRGASGGHSDTSTACKMSMVIAPIVRGRIPTIVDQVTTAVTPGSSIDVVVTELGIAINPLRTDLIAAFKDSKIPQLTIEALKEKAYAITGEPAPIKYGDRLVALIEYRDGTLIDVVKNV

>fig|1664.9.peg.1883

MDRLRRTMMFVPGANPAMLRDAILYGADSIMFDLEDAVSMKEKDSARLLVYSALKTFDYQSVETVVRINELSSGGRQDIEAMILGGINVIRLPKTETADDIRAVDAAIESVESHYQIPVGTTKMMAAIESAEGVLNAREIAIASNRMIGIALGAEDYVTSQKTRRYPDGQELFFARNYILHAARAAGIAAIDTVYSDVDNEDGLRQEASLIKQLGFDGKSVINPRQIPIINAIYAPTQAEIQNAKEVLAAIEEAESKGSGVIAINGKMVDKPIVERAYRVLAMAKAANMEVK

>fig|1664.9.peg.1884

MELKQIATAGTMESSDIMITLSPNKTGLEIKLQSNVEKQFGQQIRTMIESVLKQFEITNVTVDAVDKGALDCTIKARTIVAVYRGLGKEDYDWEEINKWIA

>fig|1664.9.peg.1885

MVTSKRIWLTINQEEAQKWQVLLNESDLTTDWQVDYTVGLFEGEELIATGSVYDNIIKCVAIKNNQQSQNLLALVVQDLLAYLEELQKYHSFVYTKPVTAVYFESLGFKVIVKTDTVVVLERGFPNIDTYRQKLLTAKREGRRIGAIVMNANPLTLGHQYLIETAAAASDVLYLFVVSENRSTFSEATRLEIVRQVAQDNPKIVVLPTDNYIVSNMTFPTYFLKEKANQDIAKVQAKVDATLFLEIIAPILSIQTRWVGEEPLSPVTQIYNEQMMAVFGNKLKLKIIPRLTQDGTVISATRVRAAIEQDDWETVQKLVPQQTYTILKGS

>fig|1664.9.peg.1887

MAALVQGITSITIGQIVMMLIGCLLMYLGIKKEYEPTLLVPMGLGTLLVNFPGTGVLTQVVGGSKTEGVLDVLFNAGINTELFPLLIFIGIGAMIDFGPLLQNPFMLLFGAAAQFGIFFTIVVAVLFGFDIKEAASIGIIGAADGPTAIFVSNQLAPKLLGPITVAAYSYMALVPIIQPMAIKAVTTKKERQIRMTYKAENISKMTKILFPIIITILAGFIAPISLPLVGFLMFGNLLRECGVLDRLSNTAQNELVNIVSILLGLTISVKMQAGLFLNVQTLMIIAFGLVAFIMDSVGGVLFAKLLNIFRKEKINPMIGAAGISAFPMSSRVIQKMASEEDPKNFVLMYAVGANVSGQIASVIAGGLLLSFFS

>fig|1664.9.peg.1888

MLRKFKISIDGQEYLVEMEEIGGTPTPVAPVTQAVEQPAAPAPEAKSESQPVVNQAAADADAMAAPMPGTVIKILVAPGDQVVENQPLLVLEAMKMENEIVADRAGQVSQIFVERNQSVNAGDALITIK

>fig|1664.9.peg.1889

MFGWFKKQKQAVPEPLKQPEEPVIEEWLTVPKYVSVDPKAYPQVVLIATSIASGENPQSQFIVRRLLVPNPEAKRVSMIASCLMATQEQQQYVVRSIKKRVSEEA

>fig|1664.9.peg.1890

MLLTIIAYAMIIVFMYIIMKKKMSPFTALVIIPLIFAIIAMLTGVAKKGSLGDFVLEGIKTTANTGIMLLFAILYFSIMLDAGLFDPITKKMIRFAKGDPMKVLIATAVVAATVSLNGDGTTTTLICCSAFIPIYKKLDMKLMNLGVLVILQNTIMNLLPWGGPTARAMAVLNVDASILAYLAPGMILALAYVIFYVAPHMGRQERKRLGVKQLTDAEIEEMTAVTDPEVQKMRRPQNFVFNGILTIVLIGWLVAGSFISAIEMPPLLLFLVGTCIALMVNYPSLKDQSKRIGENGGDAVQVVILVFAAGVFMGLFQGTGMAEALAQSFTQIIPHQLAGFWGLVIAVISAPGAFFISNDGFYFGVLPVLAQAGRAYGFSNMDMALASLMGQAFHLLSPLVAFIYLLLRLTGLDMGQWQKEAAKYALGVFIIFVITIILSGHMPLYIPQN

>fig|1664.9.peg.1891

MVADQLEAALINRGLAKPKRLEIHNLVTSTNQLAKQYLLNASVMPDVIIANQQSAGYGRLKRKFYSPQQTGIYMSLLLPDIPLNEEATLLTPLSAVAVVRAIRAVCQIETQIKWVNDIYYEGQKVVGILAESIPHHGIVIGIGIDFKDDGQLSQLVEKAGVLLPNTSPITRNQLISQILLEFDRLLPTYRQKEFMAEYREVSLLITKKIVIRLNEEKTLTGYVQDITNNGELVIQTVGQRLQIIRAGEVERVLSW

>fig|1664.9.peg.1893

MAEKRDYYDVLGVGRDASDDEIKKAYRKLSKKYHPDINKAPDAEAKFKEVTEAYEALSDPQKRAAYDQYGHAGMNGGFGGGAGAGQGFGGFGGGAEGFGGFDDIFSSFFGGGARQQPNGPRQGSDLQYRMDLKFEEAVFGKETKISYSREAECHTCHGSGAKPGTSAETCHKCHGAGQIQVERQTPLGRMMSRETCDVCGGTGKEIKSKCDTCHGTGREEERHTVKVKVPAGVEDGQQMRLQGQGEAGSNGGPYGDLFIVFRVAPSDEFERDGAQIFVEVPISFVQAALGDEIEVNTVHGPVKLKIPAGTQTNTVFRLRGKGAPKLHGTGNGDQKVTVNVVTPKSLNSKQRDALKAFAVASGDSVNPQDNNLFDKILNKKHKK

>fig|1664.9.peg.1894

MSKVIGIDLGTTNSAVAVLEGGQPKIITNPEGARTTPSVVSFKNGEIQVGEVAKRQAITNPDTIASIKRHIGEAGYKVTVGDKSYTPQEVSAMILQYIKKFAEDYLGEEVTEAVITVPAYFNDSQRQATKDAGKIAGLDVKRIINEPTASALAYGLDKTETDEKVLVYDLGGGTFDVSVLELGDGVFQVLSTNGDTRLGGDDFDEAIMNWLVENFKSDNGIDLSKDKMAMQRLKDAAEKAKKDLSGVSSTQISLPFISAGENGPLHLEMTLSRTEFDRLTSDLVDRTKAPVMNALKDAGLDANEIDKVILNGGSTRIPAVQEAVKNWTGKEPDHSINPDEAVALGAAVQGGVISGDVKDVVLLDVTPLSLGIETMGGVFTKLIDRNTTIPTSKAQTFSTAADNQPAVDIHVLQGERPMAADNKTLGRFQLTDIPAAPRGVPQIEVKFDIDKNGIVNVSAKDLGTNKEQKITIKSNSGLSDEEIDRMMKEAQENEEADTKRKEEVDLKNDVDQLIFQTDKTLKELEGKVSDEELQKAKDAKEELVKAQQENNLEDMKTKRDALSEIVQELTVKLYQQAQEAQQAAGGAEGNATDAKTDDGTVDGDFEEVKDDKE

>fig|1664.9.peg.1895

MTKQEKAENQEKPTEETVEETPKKETPFEPVMEADEVEETTEAQAPVEEADDKLAELQKKYDAMEDSFLRSQAEIKNIQMRNQKEQANLLKYDGQSLAKDVLPVLDNLERALAAEATDESLKKGVQMTYDHMKHALEDHGVKEIEAQGQAFDPTIHQAVQTVAVDGDQKADTVVQVFQKGYYLKDRVLRPAMVVVAQ

>fig|1664.9.peg.1896

MLTERQLMILKEIIRLFTESGQPVGSKKLMSELPMHVSSATIRNDMADLENVGLIEKTHSSSGRVPSMKGYRYYLDHLIQPAVLNPMDVATVQQSFGRHYHKIDEIVSQSANILSNLTSYTAITLGPEMAAIRLTGFRLVPLGNHQVMAIIVTSAGTVDNQVFTIPNAISGDELEKAIRVVNDHLIGLPLTVVSQKLKTEVPALLMQYMGSPGGFLNIFDDVLKQASQERLYVGGQSNLLNFSELTDVSQLKSIYNIINQSDDLAKLLELSPGEANSQVQVRLGNEMTNDLLKNYSLMTVNYDVGEHGQGLIALLGPTSMPYSRMIGLLDLFREELAKKLIDYYADFDDSQS

>fig|1664.9.peg.1897

MKSAIILTFRGYYDHQNFEDGCVRYHWGDDASRALFNPQAEFGWQLDRLIIGRYVCIASGVVILMGGNHNHHSDWISAYPFPEQIAASYEPKGDTIIEDGAWLGMRALIMPGVHIGQGAIIAAGAVVVKDVPAYAVVGGNPARVLKSRFTPADIETLQAIDWYHWPIEKVQAAQSILASHSVADLQQFHENY

>fig|1664.9.peg.1898

MAGAYIHIPFCEHICYYCDFNKVFIEGQPVDEYVDMLIREFQLVMAEYPDEKIETIYVGGGTPTTLSPAQLQRLLDGIHQYLPYEGGEFTFEANPNDLQDTAKLQVLKDNGDNRLSIGVQSFNDDILKKIGRVHRSADVYRAIANARQVGFENISIDLIFRLPQQSEADFMDSLKQAIDLDLPHYSTYSLILEKKTIFYNLMRQGKLRLPSQDVEAHMYQNAIDSFQQAGLEQYEISNFAKPGYQSAHNLTYWRNEKYFGFGAGAYGYLGKDRYHNFGPIQQYLEPLQAHKVPVIERHVLPLTEQMEEELFLGLRTMQGVSIKHFEEKFRYLLQNIYGETVANQIEKGYLKQEGDWLRLTESGKFLGNEVFQEFLLDSY

>fig|1664.9.peg.1899

MRVIELIHPYSKEQVPAEEIVLALGFFDGVHRAHQAVIKTAKELAVQQNRPLAVMTFDIHPAIVYRNVNQDDFRYLSTVERKQELMADLGVDILYVVHFNDGFAKLAPQDFVDQYLVALHAKTVVAGFDYTYGKKDIANMQTLSTYAKDRFEIVTVAEHDYQGHKIGSTLIRENLDQGQVAAANELLGYVYQTTGEVVHGEARGRELGFPTANIESQKPERLPGIGIYAVRLLVRGQWYWGMASIGRNVTFHANNPVTVEINLLDFSADIYGEQVKVEWYQYLRGEVKFDSAEALIDQLHQDEADTRTYFEKLEGQH

>fig|1664.9.peg.1900

MDGIIPLYKERGMTSNDCVFKVRRILHMKKVGHSGTLDPNVDGVLPICIGQATKVVDQLVHSGKVYTGEITLGLSTTTEDLDGEVVEEQRLAEPISTEKIKETLASFLGDSIQIPPMFSAVKVNGRRLYDYARAGDPVERPQRKITITQFDLQGEPEFDAKTGRQTFRFIAGCSKGTYIRTLAVDFGRKLGLPAVMSDLTRLKSGGIQIGSCVTLAQLAEAADNGQLADILIPLDHVFEENVKVALDDDQWAKILNGVFLTFPEQTEEILALTYEGHIKALYQVANTKQHLYRPYKMYLQNQGTH

>fig|1664.9.peg.1906

MKHRVGRVEQEIQREVNDILLKRVRDPRVEGVTITDINLSGDLQHVKVYYSILSNLASDAEKAQKGLDKAKGLIRSELGQRIKLYKIPEITFEQDGSVRYGEHIDELLRKLHQDEAQR

>fig|1664.9.peg.1907

MGKKRIYELAKEINVASKDILETANKKGYDLKNHMATIDDNQEKTLRAAFQTKATPAASKPATPAASKPATPAAPKASEKSESGKIKISKTAIRRRPEADKKPTQHSNNRPQANANRNGQASSGQNRTNNARPNNNSARPNNSRPNTNSRPNNNSQNRSTSANHPMSLQEQISQANARRQRTQERIQQQREQREADEKKRREQASRPRPTRNNASNNRPSNGKPTNGARPTTNSPRPTVTKDGRPLGSSRPNNNNSARPNTTNNRPTNSRPATTPSRPVSAQEMQQKMQANTVSASKPASSNTASKPKNFGPDKKRGGGYNSYGNSQQRFNKKRKKTRKQQLAEQNAAKKEMPQRKERPLPEVLVFSEGMNVADIAKKIHREPAEIIKKLFMLGIMVNMNQSLDKDTIELLATDYGIEAEQKVEVDIADIDSVFETEAKSDANLVSRPPVVTIMGHVDHGKTTLLDNLRNSHVTDGEAGGITQHIGAYQTKLNDRLITFLDTPGHAAFTNMRARGADITDIIILVVAADDGVMPQTIEAINHAKAAKAPIIVAVNKIDKPGANPDHVMEQLMGYGLVPEDWGGDTIFVKISAKTGENIDDLLEMVLLEADVLELKANRDQKAVGTVIEARLDKGKGPVASLLVQQGTLRIGDPIVVGNTFGRVRVMTNDRGRRVKEVFPSEPVEITGLNDVPQAADRFVVFEDEKTARAAGEERAKRALLKERSRSNPVTLDNLFETMKQGELKEVGVIIKADVQGSAEALAGSLRKIEVEGVRVNIIHEAVGAINESDVTLAAASNAIIIGFNVRPTPQAKIQADAEQVDIRLHRIIYKAIEEIEAAMKGMLEPVYEEKVTGQLTVRETYNVSKIGTIAGCIVDTGVIRRDSGVRLIRDSIVIYEGKLSSLKRFKDDVKEVKTGFECGVTIEDYNDIKIDDQIEAYVMEEVPVK

>fig|1664.9.peg.1908

MSEQPDRLLQLLGICMRARQLVSGEEQVLKAIQQSKAKLVFIGQDISPLTQKKLTDKSHFYKIPETKAYTAAQLTQAIGLHRATIAVVDAGFAKKMITLLG

>fig|1664.9.peg.1909

MMKQRKVPMRKDVISQEMKPKKEMVRIVKTAEGEISIDPTGKKSGRGAYVSLEPDAIKTAQAKKILEKTFGIDVSADFYTELFEYVDHQKARQELFGERK

>fig|1664.9.peg.1910

MSKEMLGALDALEQEKGVKKEIVIEALEAALVSAYKRNYGQAQNVEVEFDQKKGNIHVYAVKEVTDEVFDSRLEVSYQDALEINKAYEVGDTIRFEVTPKDFGRIAAQTAKQVIMQRVREAERSIIYNEYSQYENEIMQGIVERRDNKFIYVNLGKIEAVLSKQDQIPNEVYNAHDRIKVYVSKVENTTKGPQVFVSRTHPDLVKRLFEQEVPEIYDGTVEIVSIAREAGDRTKMAVRSTDPNVDPVGTCVGPKGQRVQLVVNELHGENMDIVKWEEDPSDYIANALNPAEVIAVQFNDGNEHTCTVIVPDYQLSLAIGKKGQNARLAAKLTGYKIDIKPESEVEFVDGDDSEDESAVVEGFEEETITPETTEETVAVAPETAEVAEDIEQPAETVVDSEEQPQE

>fig|1664.9.peg.1911

MSTVVETVHDLLVPILDQHHFELVDVEFVKEGSSWYLRVFIDKPNGIDLEDCALVNDALSEKLDSIDPDPIPQAYFLDVSSPGAERPLKKETDFQNALGEYIHISLYQAVDGEKIYQGILKALDQETLTLAIKIKTRQKEVTFNRKEIAKARLAIEF

>fig|1664.9.peg.1912

MLDRIRKGTPYFFEKLVVELLSAMGYKGTNGQAFVTQKSNDSGIDGIINQDALGTSTVYIQAKRYQVGNNVGRNEMQAFFGALSGFGSDRGVFITTSNFSNGAIEYAKNQHIVLINGVRLSDLMLQYQVGVNVKKQYTTFDIDEDFFIEDD

>fig|1664.9.peg.353

MRIVVIGSVAAGTSVAAKARRNNEDHEIVVYEKGTDISYSVCGMPFYLGGEIEALDDLIPRDAAWFKTRFNVDIITATEVVSIDEVHQTLLVKHLPTNEIVEDHYDKLVLATGAAPRVIAPFDQGYDNVFTLHHMNDTRAIDDYLKTKSISNVLIFGTGYVGLEMAEQFHRRGLSVTLVQRSRQIMTNYDGEIAYRAEQVILENGVNVIKGVTTESLEVEEQRITAVKLSDGTQLPADLVLLATGVIPQTQLVDYMGIAKGTSGAIQVTDRMLTSVPNIYAVGDVAESFSLIDGSPLYRPMGSTANKMGRIAGDAMTGGALAHRGILGTGIVRLFDTTVAQTGFTEKVARAKGYEIDVLYNIKPGHADYLNGTENVIKAITDHQTGRLLGAQIIGQDGVDKRIDVFVTAISFGAKVADLFHLDLAYQPLYATTKDPVLYTGMALENARNGRPLLSPIELYQQLTMGDSLQVIDVRSQKQYEQQHIETAISMPLGEIRKRIAELDPTMPTVVYCNKGVTGNAAQNLLLNMGFETVYNLSGGHKNYQLVKTYLQQNG

>fig|1664.9.peg.369

MGTVTWYIDDTTATLHLSGGVLPDKVANDTTNTPWYFPNSPYIARIAHISIDGEITAKDVSYMFWGLTNLTTVQGLANLKGATNFTMLFASDSALQSVDATNLDFSKVTAMNSMFSDCANLVSVGDTANWQLGQVTTMVRCFSGDKKLSQLNSTNWDTSNIQNMNNTFFNCTALTNLDVSKWQTAKMTNLGSTFSQSGITVLDVSNWDTSHVTNLSSTFLNTSIAELDVSNWDTSQVTTMAYTFSGCSRLETLDVSKWQLGKNTSLSYTFSGDQRLTQLDVSKWQTANVTNMASTFSGTVGVKTLAVKDWQTTKVITMAGMFAKSGVDQLEIADWDTSNVQSMRLMFDATKLTTLDYPDWNTASVTDMSYMLRGMTKLTDAYFTNWDTSQVTNMGGMFLNDQQLAHLRLGSKFKFLTSTSTGPSLAEPSTETPYFGKWQRHDANDNQVGNTYTSAALMAQYDGTTVPTGDYYWAVATPPTITKLVRNVTADGNNAPFKTATTAQKGDTVEYQVNITQPSGQQLDRGAVFEDVLDSHLKFNNNKELSISYSDENGDFHGEQLIKFNDQWQIALGENLAIGQKAQVLIKAIVNDDSAPEIDNLFKLVSGSYGAGTTSNTAIVHVKKPLTLTEAIKNETTDTDWDTKQDVSPGDRVGFKLDYQNTTGATSNQITFNDPLVTNELTYQTGSLKVTYQDGTTETVSDAAQTQFATTGKLTLTKSLANYEAVKLSFSATVNPLVTSGTTLHNKATLTADNVSQPVTSNTVDMNVVQSEHQLTIRYVDLDEDLSQPASAGTQIAAPITVSGKTGTALSTLLPGQQVAPKVIEGYTIYSVSEDPDLKPENWQKAYRDDPQIGDQDRIITYGYKKAMLSIDAPSSWEFGDYNNKPMDRTYYLNHNQGTPQAVTVTDNYGVQNWQLQVSQAKPFVDSEQHELTDAKWAFSNGNVKTLSNTDVGTVTNNSEHFTLASGQSATLMTMTKSGHFQSDSPDTSDPENPYTQVGQGQWAYRFGDEKSADYSIGLNVPATTKRYTGHYRTKLTWSLSVGP

>fig|1664.9.peg.371

MCTSLTYSTVDGHHFLARTMDFSFELNGNPLFLPRQHQWQPVLEKQAIQNQYALMGAGAKLGDQYLVADGFNEHGLGCAELYFAHAAVYEPQPVPNKLNLVAEEFIVWVLGNHQTLEEVAADLDNVRIIESDQGVMGANQPLHWILSDRSGKTMVIEPRGNGLQLIDDPVGVMTNTPDLDWHIKNLSNYLNLQPQPFMERPFGNYQAGLFSQGTGTQALPGSYTPPDRFVRAAYSRQYMPEANNVAEGVNHILHILDNVTIPKGVNIGSGGSSDYTQYQGISGLNNLAYYMVNYDNRHVYQTNLTTDLIENQKEPLIYHLPQGQQNTILN

>fig|1664.9.peg.374

MANMYGNDPFFNNDMDDIFNQVFRRMGNSESARYVINGRELSPDEFAQYRATGKLPNREKEIEIKQNGEHALKKGGILEKLGRNLTQEARDGLLDPVIGRYNEIQETAEILSRRTKNNPILVGDAGVGKTAVVEGLAQGIVAGKVPETIKDKEIYSIDLSSLEAGTQYRGSFEENIKNLIKEVKAAGNVILFFDEIHQILGIGSTGGEDGGKGLADIIKPALSRGELTVIGATTQDEYRNTILKNAALARRFNDVLVNEPSAADTVKILQGIKGLYEKHHHIELPDDVLKAAVDYSVQYIPQRTLPDKAIDLLDMTAAHLAAKRPETDKAKLAAELKQLEADKAEAVEAENYQAAEDLKVRIEKIKTQLENNDQHAAVMIATVDDIAQSVERLTGIPVAKMGSNDIQRLKNLGQRLKDKVIGQDEAVEMVARAIRRNRAGFSEGNQPIGSFLFVGPTGVGKTELAKQLALDMFGNKEAIIRLDMSEYADRTAVSKLIGTTAGYVGYDDNSNTLTERVRRNPYSIVLFDEIEKADPQVLTLLLQVMDDGRLTDGQGNVIDFKNTVIIATSNAGFGNEKLTMNDQEDAKLLDKLAPYFRPEFLNRFNGIVEFTHLTKADLSQIVDLMLTNVEQTLAKKELTLVVSKEAKDWLMEEGYDEAMGARPLRRVIEQQIRDKVTDFYLDHADVKDLKADLVDDQIVIQAVDSEN

>fig|1664.9.peg.378

MQTAILTGITAYISTSIDYLIILMVIFGSVNRKERWLVYWGDLLGTSVLVAASLVMAFILGFVPQEWLLGLLGIIPVLMGLRLLISGESDDDEVVESQMKKRRNIIMNVAIITIATCGADNIGIYVPIFAQSTVQTLAVILITFFFMLSLFCYVGYLLIKIPKVAEILERYGMYITAIVYIGLGLYIMFESGTIQHIVKMLG

>fig|1664.9.peg.382

MKPLIVYYSRTGNTKAVAKLIHAQVGGDLVALETQEKRPTNYRAEVDLNAREQLQNQLPPLKTNIPDFEQYD

>fig|1664.9.peg.385

MDMVQLNNGVQMPQLGFGVFQMTDLAECEQAVVDAIASGYRLIDTAAAYHNEAAVGRAIKRSGVAREDLFITSKLWVSDASYERAKKGIDQSLQNIGVDYLDLYLLHQPFGDVIGAWRALEEAYKAGKIRAIGVSNFYADQLKNLILSTNVKPVINQIEVNPWYQEHSEVAFAQAEEIQVEAWAPFAEGKHSIFTDERIAAIGSRYGKSNGQVILRWLLQRGIVVIPKSVHQNRMAENINVFDFELTTAEMATMNDLDKGESQFFDHRDPAAIEQIFGTSLAALKENK

>fig|1664.9.peg.389

MTNQKVVIITGASNGMGAAATKVFADHGWIVYGGARRVGKIPTGQDIHALSLDVTDDESIRSFAKTVLEEQKRIDVLINNAGYGENGPVEDTPTEHIHRQFETNFFGAVTLTQLILPRMRQQRFGRIVNISSIGGDIYSPLGAYYHATKAALQQWSDTLDIEVEQFGIQSVVVQPGGTQSSWGSVAVENIYKNLKKDTAYKPLVAYMADVLRDDAPGMGASVEDLANLFYRAATDTKPKRRYFNNFHDRLTVMIARSFPGRFRKQVLKGLNQNKK

>fig|1664.9.peg.391

MAKLVFIRHGQSEWNLSNQFTGWVDVNLSEEGVRQAQNAGALLKKEGILFDQAYTSVLTRAIKTLHYALEGSDQLWIPETKSWRLNERHYGALQGQNKAEAAEKWGDEQVHVWRRSYDTLPPLLDANDEGSAANDRRYAHLDPKAIPGGENLKVTLERVIPFWEDEIAPKLIDGQNIIVAAHGNSLRALTKYIENISDEDIMDVEMATGEPVVYDLDENLNVVSKKKLN

>fig|1664.9.peg.393

MKKTTNRWIALVAMSLGVFMGLLDVTVVNVALPSMAVDFKTTFSNLQWVLNAYTLMYAVSLLIVSKLGDMFGRKKIFIMSMILFTVASVVNGLAPNLLVLDIGRAVQAIGGSGMMSLSMALVASNFEGRERGTALGILGSVIGFSTAIGPLVGGFLVENFGWPSIFFVNLPVGIISVYLVIRNVKETPAYGAGESIDFMGMILSAIGLFSGIYGLIQKEQHMHWAWTDMRIMGWLVASVLILALFVFVELKAKAPMMNLTLLTNRHFVGVVILAFSLGAGIFALAAYQTSLMQNYMGYSAFSTGVHQLPMSLWSLVLGPFTGVLGYKFGKKWMIAFGFTVSALGLLIFRQVTTTDFTYMQLLPLLALTGLGNAIINPMINTAAMDNINPKEIGMASGLLNVFRQIGITVGVVILGLSQTNAYESSLSAGFSGSSMPKAAATGIHQALVEAGAFSGHAVAWSQQLVKTPYAAKVQQMVVTAFDKGMIAVVTTSLVLMVIAILAALFLLKETKSVDQK

>fig|1664.9.peg.394

MYELLMLSALMSRNMSGYKLRIILENAMLPRRKISNGVLYPLLDKLESKGCIGFDEADQDSRGTKIAHITEEGRRYFAELMAKPVAHDAKWDDAYRFKMRGMHYIDSEEQISILQDYRDELAEDLNVYMGVKNHLTDLRDEEGTDTNYYQWQVRSMDFVITTLKAKVDWLEAQIREIEKMEIVK

>fig|1664.9.peg.396

MKKVIVLGSTNVDTILNVQRFPQPGETLAMDGRAVAGGGKGANQAIAAVRSGAQTAFISKVGKEGAADFMLDTFKKDGMNVDHVRCSETAGTGQAYIMVDAHGQNSILIYGGANQDVTVEDIHEAEAAIKEADRIVAQFEVPVPAIIEAFKIARSNGVQTILNPAPAIKNIPAELLAVTDMIVPNETEAEIITGIKVTDEASMKANAEAMFKLGIKVVIITVGSKGSFYATPTATGFVPAFKVKAVDTTAAGDTFIGALSTKLELDYSNIEAAMTYANKASSIAVQTKGAQNSIPYEKDIQL

>fig|1664.9.peg.397

MRKTKVINTQISSVISDMGHFDTLSIGDAGMPVPAGTKKIDVAIENGVPSFIQVLTNILSELEVQKVYLANEIKTANPEQLEAIKALIGETPIEFINHSQMKQDLNKAKAFVRTGEMTPYSNIILESGVVF

>fig|1664.9.peg.398

MNAVNILIGLMPMIGWGIFPVIVGKIGGKPASQILGTTIGTLILAIVVAIFRGTPIPETKTFIFCLISGACWALAQIITFHVFETMGVSRTMPITTGFQLVGASLWGVFVLGNWSSSQSKLIGFTAIALIIIGVYLTAWSEDKSSASKSGAVKGVLLLLVGELGYLGYSAFPQAVSADGFQGFLPQAIGMTIVGIIFGVTQTKKDYKPFKEATSYKNIFSGFFFAFAALTYLISAQPSVNGLATGFVLSQTSVIFATIGGIYILKEKKSKKEMIAVMVGLLLVLVAGSVTAFIK

>fig|1664.9.peg.400

MEKILAINAGSSTLKWQLFEMPSETVIAKGMIDRLGLSDSVFTAKYGDNQKFKEVQDVTTHEMAATLLLTRLKELGIVSHLDEVTGVGHRVVGGGEAFSDSMVINPVALDEINRLAEYAPLHNPTQAYYIKIFTTLLPGVPQVAVFDTSFYSTLAPENYLYSIPQEYYQKYGARKYGAHGTSHRYVAHRAAEILETPLESQKMITLHLGSGVSITAVQDGHAVDTSMGFTPLAGITMGTRSGDIDVSLVAFLAKKLEITMPEMIDILNHKSGLLGISELSPDMRDLEETADTRPQSALALSIFVNRVVKYVGSYVALMNGIDTLVFTAGSGENGSELRADICKQLTCFGVKLDEEKNNVRSQERIISADDSKVKVLIVPTNEELMIARDVMRLK

>fig|1664.9.peg.402

MESACTTILVGKKASLDGSTMIARNDDTFMPLTPQRFYMNPAVKGRKGEKIKSWLNGFEATLPEDGYRWPAVPNVDYKNKGYYDESSINEVNVAMSCTESTYGNEKSLAYDPLVKDGLDEDCMQAMVAPYINSAREGVSYLGNLIKQYGSPAGNSVLFSDHDEVWYMEIVTGHLWLAQRIPDDGYAVAANQVAIQQVDFSDKDNFMWAEGIQEYVEKHHLNPDKTGWNFRHIFGTMNEKDRHYNTPRVWYAHHLFDSEFDMSPESADLPFIRKIEHKISVEDIEMLLGSHYNETEFDPMGHGADADKYRYRPIGLNRTQNAHILQLRNDVPKEQAAIMWITLGMPTYTPFIPFFTNANDTDPSFSETPMKWDINSAYWMYRTISVLVEGHYSQHIQGNVDYLTSCKQELRTMLDSIEEEAKNYQGEALTKYLTEQNYLIVNTMKDKAMGMIGELVMSGINLSKLTFNMDRNL

>fig|1664.9.peg.407

MKQGTTILTLDNGYHLWTNTQGTGDIHLLCLHGGPGGNHEYWENFGKELADLGVQVHMYDQLGSFYSDQPDYSKPGNDQLLTYDYFLDEVEEVRQKLGIDNFYLIGQSWGGALVQMYAAKYGQHLKGAIISSMVDEIDEYVTNINKIREDIMTPEQLKFMQDCEAKNDYDNDEYQALVDKLNAGYVDRKQPLAISHLIPTMATDVYGVFQGDNEFVVTGKLKDWHFRDQLHKITVPTLITFGEHETMPIATAKIMAEKIPHSRLVTTPNGGHHHMIDNAPVYFDHLKTFIKDVESGNFKD

>fig|1664.9.peg.408

MTNLALVIVLLAALMTPLLMAKFKMSYLPTAVAEIIVGIILGTSGFHLIVSTPTLTELSTLGVTVLIFLSGMEIDFDLFKKQPTPTDPQLKVQPSPVRLAITSFAFILLISFGLSGLLAWSGFFKDIGLATILFSTIALGVVIAALKEKELLSQPLGQTILLIAVLGEIIPLVALTVYSSLNNPGSKSLWLLSLIFVAAIYLLVRFKGVYHFFARIDKSTTQLDIRLAFFLIITLVSIAEEVGAESILGAFLAGMVMKLLRPREETTDKLTSIGYGFFIPIFFIMTGAKLDLPALLADRKSLSLIPFFFIGFMLSKAATYWVLKRRFKQTNAIAGTFLSATTITLVLPILTVGLNLKTITEQQSGAFTLAAILSCILGPILFNRFYQPTPEDLPRKRVKFIGANIMTIPIAQQLSNGLYTTQLFTDNQGNYEIYHSAADLTLLPDLSEDSLVTSGAFDADIIILGYLNHEQNYRLAQLALDAKVPRIIARFEAKDILDEKYDQLKEQGVEVFNTYEANISLLRSLIETPSTLQIIDNTDAGLFEVTVQNRRFTGLQISNLPFVDQVTISRIYRNQELIMPHGDTQIHLNDHLILTGNKKAVPAIRQQLETRN

>fig|1664.9.peg.411

MTDVQKSLEQSRLTEIIGLIKKAETHFEGAVKRAKSEEKEINENFFNDVRLNFNNDSAMTETAVSIEQQRQMLQERNHSWQQSSRQLETLQKMEKTPCFARVDFQEKGEPKSESIYIGLGSFTDTDDHFLIYDWRAPISSIYYEGKTGAVTYQTPDGPQDVNISLKRQFLIEDGQIKALFDTQETIGDQMLLEVLGEKSDTQMKSIVTTIQREQNQIIRNTDADLLFVQGAAGSGKTSAILQRIAFLLYRYRGNLDSSQVIMFSPNQLFNDYIADVLPELGEQNMIQMTYFQYVARRLPNMAVQSLFDQFETPVNETDGRISRLKESLVFYKAVKKYAAHLEKADMRFRDMKVKGRVLITKEHISEVYYRFNENYHLGNRLDATKESLLRTLSHKVDHETKADWVTEQAENMSDEEIRELMSGKDFKTGDAESKFIAKQIVLEAFKPVRRSIQRNRFLSIQAQYIHFLRVVPQLVKLADFDITEEQWQYHVTHFVAQLKAKQMTLQDVTPYLHLYDLMTGKKGERDMRFVFIDEIQDYTPYELAFLKLHFPKARFTLLGDLNQAIFTKGNSTNLLQQVQQLFDAQNTKVVQLTRSYRSTQQVTDFTKGLLKGGQAIEAFNRVGDKPNLIVRQSEAELVADVNRQLAANEADELSTAIITKTLVQAQELTKMLKETGTKVTLIRSENQRLAAGTLVVPSFLAKGLEFDAIIGWQISAANYNHEDQRQLLYTICSRAMHRLTLLATDALSPLIDNVDPEKYTLTK

>fig|1664.9.peg.412

MKNEMNYYEISREEWRYFHGRGEKYTAITDDELQQVSGLNDRISLTDVSDIYVPLRHLLQMKYEQFQENQIKQSEFLEIKPHKTPFIIGIAGSVAVGKSTTARLLQLLLSRVYPDKTVQMITTDGFLYSTSELKQKGILDKKGFPESYDMPQLISFLNAVKNNVAPVKAPKYSHQIYDIIPDEFDIIDDPDILIVEGINVLQLPTTEQIYVSDFFDFSVYVDANPSLIEKWFLERFDLLLDLAKDDPTNYYYPYAISDRASAFKMARRVWRDIDLRNLNDYILPTRNRADLILHKTKHHLIDKVFLRKY

>fig|1664.9.peg.413

MAQKDMQDFDKIIVLDYGSQYNQLITRRIREFGIFSELLPNTTTAAEIKKIAPKGIIFSGGPMSVYDDGAFSVDPEIFELGIPILGICYGMQLISFKNGGNVEASTEREYGKAEITVTDKDSDLFKGLPEKQTVWMSHGDKVTAIPEGYVTVAESDNTPFTAIENRDKHIYGIQFHTEVQNTEFGNDILKNFAFGVCGAQDNWTMNDFIDMQIEKIREQVGDKKVLLGLSGGVDSSVVGVLLHRAIGDQLVSIFVDHGLLRKGEVEQVMESLGGKFGLNIIQVDAKERFMSKLAGVSDPEKKRKIIGNEFIQVFDEEATKLNGIDFLAQGTLYTDIIESGTSTATTIKSHHNVGGLPEDMQFSLIEPLNTLFKDEVRDLGEKLGMPYELVWRQPFPGPGLGIRVLGEVTEDKLKIVRDSDLILREEFALAGLDKTVWQYFTVLPGIRSVGVMGDGRTYDYTVGIRAVNSIDGMTADFSRIPWDILQKVSVRIVNEVDHVNRIVYDITSKPPSTVEWE

>fig|1664.9.peg.417

MKDIYVVGAAIIKDNQILCTKRATDRILGDLWEFPGGKIEADEAPEAALVRELKEELGADILVGPAVATDVHQYDFGNVHLTVYYAQFTKESFGLVAHSQMKWCRQSELDQLTWAPADVAAMQAIQKQDLSQLTFK

>fig|1664.9.peg.418

MTDLQTELQNSALAGLVDKERYISQDEYLPKLLFNTAEDHVKTHLDEELMTCQHFTFAVAFITEAVLAMLKPKLADLALKGVTGRILTSNYLGFNNPKVFKELLKIPNLEVRVLKQSDSFHAKGYIFDKGEYQSMIIGSSNLTETALIRNYEWNLRITSYENAALTDQVINEVEQQWQRAAALTPEWITDYEADYQTIVFNRQKNEPIETVAEESADYIAITPNHMQKDALKSLNFLRHEGHQRALIVSATGTGKTYLGAFDVKRVNPKKFLFIVHREQILKKAKASFHQVLGGDWSEYGILSGNENQREARYLFATIQTLAKESTLSQFDPEEFDYIMIDEAHKSGASSYHRVIDYFKPQFLLGMTATPERTDDFNIYELFDYNLAYEIRLQDALEEDMLAPFHYIGVTDYELNDEIGEEKTPLQNLVSSERMDYVEQQLNYYGYSGDQVHGLIFCSRKDEAKEVARIMTTKGYPSVALTGEDNVDYREQVIQDFETGRYQYIVTVDIFNEGIDIPCINQVVMLRNTQSSIIFIQQLGRGLRKFPRKDFVTVIDFIGNYKNNYLIPIALTGDHSRNKNSLRNKLSTDQIIGMSTINFTEVAKQRVYDSINNSNLTELKQLRDDYQDLKKRLGRMPMLFDFTTHGSIDGSVLIAKYDNYYQFLLKMKEDVHLNSYEDTVLRMLSKELLNGMRIHELLLLKLLFEKDGVVSVDDFKKALEQVNARVDQKTLDSMLSVLNLKFYQKANQKKYGMEPYVILSNNEYYLNEEILRAYQSNLNFDNLVDDVLATGLFKADDYDQTQPLTIGKKYTRKDMCRLLGWEKDLSAVVNGYKLAYNTCPLFITYTKSQDIDDGIKYEDEFLNSQIMRMFTRSPRKLDSPEVQAMVQGNASGVLKMPLFVKKSDDEGSDYYYLGLVNIDQKSLRQESMRNKKGKTVPVVTMNLILEKPVQYNLYLNLTKN

>fig|1664.9.peg.419

MMKSILLVGQSNMAGRGFIQDVPVLRHERVKMLRNGRWQMMAEPIHFDREVAGVGPATSFAAAWVQAHPDEELGLIPCAEGGSSIDEWASDEVLMRHAIAEAKFAQEFSELIGVLWHQGESDSLKGGYQTYAAKLTAVFSHLHQALGQANLPIIVGQLPDFLGQEGFGASATEFNDINREMANVVAQDPHSYLVNAAELTANPDGIHIDAASQRRFGLRYYAAFANQEDVLAVLPDEAIQLEALYQRPETPQEKMYRLSRAFALGQMSYPEFIAQLTGEGTAK

>fig|1664.9.peg.424

MIQNRIKVLRAERNWTQADLAERTGISRQAVISIEKYKYTPSLELAFKIAQVFGVEITTVFSPEEDSK

>fig|1664.9.peg.427

METTTQSTPHLSRSQKHADKAGMSLTEWQAATKFDGTDWGWVFMSIGMAIGAGIVFLPVKVGLVGLWVFLVSAVIAYPAMYLFQKLFINTLAESEKCEDYPSVISGYLGKNWGFLLGVLYFLMLLIWVFVYSTAITNDSASFLHSFHVTKTVWSNNPFYGLIVICLLVAIASRAEKLLFKISSFMVCTKLIIVFVLGVVMIQHWDLRNVAAFPDLGYLIKQTIILLPFTLTSILFLQSLSPMVISFRSHNKNIEVARYKSIRAMNIAFFTLFFVVFFYAISFNLALGHDQAVEAYEKNISTLAIAAQNMNGNSIKILSFILNIFAVMTAFFGVFLGFHEACQGIAVNILHRIMPKHKINKKNLSFGILIFAILVSWSAILLNAPVLSFTSLCSPIFGMIGCLIPVYLVYRVPALNKLKGPSLYLIVFTGILLMISPFLAFI

>fig|1664.9.peg.429

MQTILGSSGQIGQELAKELYKNYTHDIRLVSRNPRKVNPEDQLSPANLLNFEETNQAVAGSEIVYFTAGLPMNSDMWTVQFPIMIDNVIRACQINHSKLVFFDNTYMYAKNATPQTENSPFQPDGQKATARAKMAQTVIDAMQAGQIETVICRAPEFYGPAKTNSITNTLLFNKIKAGQKARIPVSDQFLRTLIWTPDASRAMALIGNTPDAYGQTWHLPCADSITYKGLVELIQEITYQPISYSIIKMWQFKLASFFNQNSRELQELLPRYQADNIFVSDKFKTRFPEFKITSFEEGIREILTV

>fig|1664.9.peg.430

MPNYKALQQDLTDVSQFLIALGDPKRQAIIIRLLEESDCDGLQVSELTEATGLSRPAVSHHLKVLKDAKLIDYRAEGTKNFYYLNHDTTEIVKLKQFLDKVTQIMTTHTK

>fig|1664.9.peg.431

MFAADNNYADQLLIAIKSILAHIPAETTVHFLILDNELTPQTKCLVRQITKQSHQVDFIKINQQLLKNCPESNHINKTAYYRILAPQILLRKGISRVLYLDVDILVQTDITPLYESHLGTNIVGAIIDPGQALALPRLGVSPEKSGNIYFNSGVMLIDTFRWEENQISELTLRFINQHPERIIFHDQDALNAILAGKVQLLHPAWNVQNSLIFRKHQPINATYKKLFDEAIAQPKIVHFTTHNKPWNTLKEHPFLAQYQAYSQQLGALKKDSINIVSAVNAAFIEPLAILYASILNHNDTQRRYSFYVLEDHLTEADKIPLKQVVAAFNADLTFLKVDEALLANIVESDRILKSAYYRILIPQVLNGIDRALYLDCDALCNVNLERLWNIDLGEFPLAAVEDAGFHQRLEKMAIKCHSTRYFNSGMMLMDLKKWRQQAITEKTLDFINHHPEKLRFHDQDALNAVLHDQWLHLHPKWNAQTNIIMDKTTPPQHLQQQFIEAKKAPAIVHFCGHEKPWHAVSTHPFTPQYRYYRHRFLKPKRQASIIPFSKNTQISLEN

>fig|1664.9.peg.433

MVSRILPIENGHNFRELGGYQTLDGKTLKTKRLLRTANLADLSPKDLALLNSYNVSVDIDLRSKDERQQAPDKVPVSAKYKSVPIFKEDQTESTTTEAELYQRYTNNPLGGQMQMQKVYQELVQDEHSIKMYRQFFEILLQQSADNQATLFHCTGGKDRTGMAAYYLLNALNVPEPVIRQDYLLTNTASQQHIAQRMSRLAAKHASSGFIKSTHALMSVQSAYLDTATTTIKQNWGSTQNYLQTALKLSKSDINQLRATYLN

>fig|1664.9.peg.434

MVKADLKDPRELYKVSGFEEQEQPAPALQGKMRPKPDCGEADYQGREQLMNRKVLITGGDSGIGRAVAIAFSREGADIALQYLPGEESDANEVAGLIESAGRKAVLIPADFKMQEAPQQVVAQAVAQLGGLDTVVLNAAQQVAHPTLEDLPIQQVRDSFEINVVAMYGIAKAAIAHIPAGGSILTTTSIQGFNPSSQLLDYAATKAAIRNFTINLAQQLADKGIRVNGVAPGPIWTPLQLAQGQLEGALPKFGQNTLLKRAGQPVEVAPVYVFLASNAASYITGQIYGVTGGESIN

>fig|1664.9.peg.435

MLHWLWVLIIGALIGIIAGAITSRDLPAGWIGNIVGGLVGAWLGQSLFGSWGPQLAGMALVPSILGAVIIVFLVSMLFSRKKS

>fig|1664.9.peg.439

MDKPNKPMDKMMDKPNKETPDHNAQIEKELNFSDGVIEKIAGQTVHDIDGVLELSGGMMSQLADRFRDEADPTKGVDADIDDQEVTIELDAILEYGKSAPDIFEQTTDQIAKSVHKMTGLNVTKIKMNVSDLLTKKEWAAKQEAGKKDNKDNE

>fig|1664.9.peg.440

MTNQLTTNEGQPWADNQHSQTAGQRGPVLIQDYQLLEKLAHFNRERIPERVVHAKGAGAKGYFKVTKDMSAYTKAAVFSGVGKKTPLITRFSQVAGEAGYPDTYRDVRGFAVKFYTEEGNYDIVGNNTPVFFVNDPLKFPDFIHSQKRDPRTHARSQDMQWDFWSLSPESVHQVTILMSDRGIPASYRMMHGFGSHTFKWVNAQGEQFWVKYHFKTNQGVHNLSNELADELAGKDTDYLQNDLFDAIETGDYPSWTVAVQLVPYEDGLNYPQDIFDVTKVISQKDYPLIEIGQMVLDENPTNNFEDIEELAFSPANLVPGIEASPDKLLQGRLFGYKDAERYRLGANYEQLPINRPKVPVHNYERDGAMAQNQETGVNYEPNSQDGPTEVPAAKIHSDQLSGTTGNFSTDPDYYSAAGKLYRLLSADEQTRLIENIRMNLGQVTKPEIQIREVKQFYQADPEYGRRVATALNLDLAQFE

>fig|1664.9.peg.443

MKFVRRLMALSLAFVGLMLCQMTQVQAAAQTQKADFEVQPILPDEQEDLSLNYFNMSLAQGQTKKIEMRIQNFTDHAITVHSDLRNSMTQVGGGVSFQANTKGLDPSLKVPFTKIAKLDKKSETIKLAAQETKILKMTVKMPEDRTNGMIYGDWHFIEYLHKKGGQSSVGSNYAYSVGVALKGQHYKVYPELKYDKTEAMIYRRHAAMGIKIRNTQPMVINKVSAKAVVSKEGLFSSKHVYTTSNQSVAPNSVLTLPISWDYEQLKPGKYTIDTVVHGQNLWNKLPLTWRFKKSFTIKADDVKTVNAQALKKPKNKWAYVATASGVLMLVSITGLIKVLRRS

>fig|1664.9.peg.446

MKKPIKGSCTTVLVGKKASIDGSTMISRNDDGHEALDPQRFVVVNPEDQPRDYQAVISGVQIKLPDNPMRYTSIPNSLLTNGIWPAAGINSENVTMSATETITTNPRIQGLDPLVPGGIGEEDIVTLVLPYIHSAKEGVERLGALLEEFGTYEPNGIAFSDQDSVWWLETIGGHHWAAVRIPDDAYVVAPNRMNIDHFAFGSDNTLCSADLKALIDDNNLNPDFEGYNLRHIFGSATIKDTVYNNPRTWYGQQYFNPEIKQDPMDHDLPFICHANRKISIEDVKFVLSAHFENTEYDPYGSAPEDVKTRFRPIGINRNHNVHILQVRNNVPAEIAGIHWLAYGANTFNTVVPFYANVNDTPESYKNATGTFDLNNMYWLSCTTALLGDTDYDFYVDMRNTFELEAMSAYHEIQNATDKAFDGQKDAVAFLEEANNKLATESLQRQTKLLGEMVISGSEHMKLRYNLND

>fig|1664.9.peg.447

MLTVKNLTVAYDDTPVFTDVAVHFDAGKITGIIGPNGAGKSTLIKAILGLVKAQQGSVLYQGKSMRAVQKQVAYVEQRKDLDLNFPISVFDVVLTGTYGKLGLFRDPGKQAIAASRAALEQVALADFERRQIGQLSGGQLQRVFVARAIVQEAEIIILDEPFVGIDLQSETAIMAIMKQWRDAGKTIIVIHHDLNKVSQYFDDLVVMNHGIVDYGPTDQVYNAQNIERAFSADLSAVLFEKQEVDQ

>fig|1664.9.peg.448

MTSISAFMGALGRYEFLQSALLTAIMVGIMSGVIGSFIILRGMSLMGDAISHAVLPGVAVAYMLGINVLVGASVFGILAVVLIGFVATHSKIKTDTSIGVVFSAFYALGFILISMAESATNLHHILFGNILAVSDQDIMTTAIVLGIVILFVSFFYKELLITSFDETYARTYGLKTQVLHYGLMLVLTLVTVSALQTVGIILVVAMLITPAATAFLWTNKLVTMLFLSASIGAVAAISGLYFSYTFNWASGPAIVLMAAVLFAVSFICAPKQGFLKWRSAKGGQQR

>fig|1664.9.peg.449

MKKILITLLTVAGIIGGVYGFIHQRAQAKIQANSQHTKLRVVTTNSILEDMVANVGQDRIELYSIVKRGTDPHEYEPQPTDISKATDADVLFHNGLNLETGGNGWFKKLVSIAHKKFGEDVFATTKGIKVQHLTTNKDEPDPHAWLDLANGMQYVENITVALQAKDPKNADYYRQNADQYIARLEKLHAKAQTQFADIPEKRRVLVTSEGAFKYFGAAYDVTPTYIWEINTESQGTPTQMKSVLAKIAATDVQSLFVETSVSPKSMAKVAQETGLPIYAKIFTDSLAQKGKPGDTYYTMMKWNIDKIHAGMSHQ

>fig|1664.9.peg.450

MVDLADCLLVIDLQNGVCKGKQPVANFKQLIEQVNARIEIYKANKRPIIFVQHNDTTLLAPQYAWQLVPELSVPADAQFVQKTHANSFWQTNMQVLLEQKQIHSLEICGAQTEYCVDTTVKVAHSLGYQLQMVSGLSTTVANSLMTATQTIAFYEDIWADRFLTLIEG

>fig|1664.9.peg.452

MKKLKDIKQYSSYAFGAFGHDAFYATLSTYFMIFVTSQLFDTKNAAFNAKMIGYVTSMMVVIRIVEIAFDPLIGGVVDNTRTRWGKFKPWLLIGSLISSVGLVMIFTNFGGLATNSPYLYLVLFAIVFVILDIFYSFKDIAFWSMLPALTVDSEKRTKFGAIARFGSTLGAQGVIIVIVPIVVFFSQMFSGTHGSEQTSAGWLGFAVVIGVVSFLGALATIIGTKEEKSAIRENTEKIRFRDVFKVIGKNDQLMWLSLSYFLFAFSYVITNSLLIYYFKYVMGRAAAFSTVGVITAILGVISVALFPTLVSLIHRRAIYLGGIAMMMLGYILFLFAGSNLVVVLIAVGLFFFPYPLVFLAALMTITDSVEYGQLKSGTRNESVTLAVRPLLDKLAGAFANGVVGIAAIHAGMTGNAKPSDITNGGLLQFKTYMFFVPMALLIVSALIFMAKIKLTEAKHAEIVSELEKKLD

>fig|1664.9.peg.453

MKKLKIVLGVIGVILVIGGGFYKIKENQAIRAIKTQNKQVSNRINQDKPISFLLLGADTGSDGRVDRGLSDSMMVVTLNPKTKKTLVYSIPRDSLAEMVGSKTKNVQKINAAYELGQAKMAKKTVSEFIGVPIDYSVTIDMGALKELVDFVDGVDVKTNIDVSFDGQTIKKGRHHLNGKQALVYTRMRYQDPRGDYGRQLRQQEVLRGVADKIQKPQYLVHLSGLMTKLGKHISTDLTADQTKTLIENYHQCGTDITSGQIIGKEAWINGSSYQVIATDKLQATSNKLRESLGLQTKTLSNTETELNQLNTAFFKDNTNKTFNTDGLNTTYYSDNTY

>fig|1664.9.peg.455

MGLGTRLFRKESLERYLQQDQRLAKTLTAKDLIALGVGAVIGTGIFILPGTIAALHSGPAITLSFMIAAVVCAVAAMCYAEFSSALPVAGSAYSYGNIIFGELIGWLLGWALFLEYMLSVAAVSTGWSAYFVSFIEGFGVHIPKAITGSFDPAHGTYINLFAVLIVTLISVLLMSGTRSSTRINNLMVMIKIGVVLLFLVVGIFYVKSSNWQPFMPFGVSGVFKGASLVFFAYLGFDCVSASAAEVKNPQKNLPIGIIGTLVICTLLYILVAFVLTGMVSYRELNVANPVAFALQVVHQNWFAGLLSLGALAGMFTMMLTMTYSSSRLVYSIGRDGLLPKMLGKIETRHQTPINSVRVVTVIIATLGGLVSLDQLTNLVNIGTLIAFFFMSIGVIPLRKRKDIPNKDGFKVPLYPWLPLLSGLLCLFMLFELPAVTWMAAGIWFILGLIIYFSYGLKHSRLND

>fig|1664.9.peg.461

MSRYTGPKWKLSRRLGISLSGTGKELARRPYAPGQHGNDRRGKISEYGMQLSEKQKLRLMYGLTERQFRNLFARAGKIREGKHGVNLMILLEQRLDNIVYRLGLASTRAQARQLVNHGHVTVDGKRVDIPSYEVKPGQEISIREKSKNLVIIKDAIEGTVGRPSFVEFDADNLKGSLVRLPERSELEPEIDEALIVEFYNR

>fig|1664.9.peg.462

MLYILIGIVVVVLIGYLGVAFYQRYFTKQIKSLEERKGALMALPIPEKLTKLRSLRLTGESQQNFDRWEKQYNDITNHNFEAIEAYLFDAEKANGKYQFLLVARILKQLRAYLQETDQDLVDVKDALDQLLANEADTREQIETLRVKYQGLRKRLLTKSFSFGPGLEGLEAILGQLEEDFNQANELTGAGDYLGAKMMLQKLIEQTDDLEDKMARIPKRYSELANEFPEQIDEISETYQAMLREHYNFGDNQLEVQLTQVTERVDKSLDLMEALDIESVEANNVEIAQNIDQLYASLEVELNARQTVEEHIGKTETIVSHAQTQNRELLLELDHLNQSYALTHDELVTAKKLTKQINEQKAMLEGHQGKIANHEAVYSVIADDLMAIDERLTEIETQQRDIHEQVSGLHQGEAVANENLQQFELELRNLKRTVEKLHLPGLADSYLDFFFVVTDEIKRLDHDLSQIKINLDEIAKQMVLIQDDLDKLKVKTNNLIDSALITEQLLQYANRYRIDFPEIQTACEEAQAVFNREFDYPKSVDILATALERIDPGAYTQVEKNYYSQKQTDAL

>fig|1664.9.peg.463

MIYFDNSATTKIAPGALQTYQAVSEQFYGNPSSLHVVGEKAFHLLEQSRQQIADLLNVQSDEIYFTSGGTEGDNWALKGTAIEKSAFGKHLITTSVEHPAIIKSMQQLEKLGFEVTYLPVDKFGRINPADLKAAIRSDTILVSIMAVNNEIGTCQPLMAAAEILKDYPNIHFHVDAVQGIGKGIQKEIFNDRVDFVTLSGHKFHGPRGTGILYKKRDRHLAPLLTGGGQEHDLRSGTENVPAIAAMAKALRLLLTDEAQKVQKQAAIRERIYQHVSQAEKTVMFSQLTPDFVPHILCFAIKGVRGETTVHAFEEHEIYISTTSACSSKKGMESSTLKAMHVNEKIATSAIRISLDEYNTLAEADEFIKAFDQINHRFKKINS

>fig|1664.9.peg.464

MQYTEIMVRYGELSTKGKNRNDFIGRLNGNVTKALHEYKQLRIHPKRDRMHIILNGDDAEGVIERLRHVFGIQNFSPSIEVNRDLDSVKETALAMMKEIGKPGMTFKVNTRRSDHNFFLDTNDMNRELGGYLSDELPELEVQMKKPDITLRVEIRQDAIYLTNQVIQGAGGLPVGSAGKGMLMLSGGIDSPVAGYLTLKRGVDIEMVHFFSPPYTSDNALNKAKELTAKLVPYVGGIKFIEVPFTEIQEEVKHSVPEGYLMTIQRRMMLRLTDQIRAKRQGLAIFNGESVGQVASQTLESMMAINDVTTTPIVRPVATMDKNEIIEIAKDIDTYDLSIMPFEDCCTIFAPPAPKTRPNLDKTRFYEQRIDVDALIERSLAGVKVTEIKAGDQFLNQDEEIIAELL

>fig|1664.9.peg.465

MRTPKHDLPEAVAKRIPIYYRYFKLLETDGIERIKSEQLAKLVAIPSATIRRDFSYIGDLGRSGYGYEVSHLIQIFSAVLKADILTKMAVIGVGNLGRALIENNFRRNDNLQITCAFDTNPALVGQTLNGAPIYAIDQLATVIPAAGITTAISTVPSEASQRSAEQLIDAGITSILNFAPTRLQVPRHINVRYLDLTAELQTLLLFEE

>fig|1664.9.peg.466

MTDHKEMSTKYDPNQVEDGRYQDWLKEDLFKPNANPDAKPYSIVIPPPNVTGKLHLGHAWDTTLQDMLIRQKRMQGYDVLWLPGMDHAGIATQAKVEAKLAEQGISRYDLGREKFIDQVWEWKDEYAATIHDQWAKMGLSLDYSRERFTLDDGLSDAVRKVFVNLYNKGLIYRGEYIINWDPKARTALSDIEVLHQDDEGAFYHVSYPLTDGSGSIEIATTRPETLPGDTAIAVHPDDERYADLVGKTVTLPLMNREIPIIADHYVDKDFGTGALKITPAHDPNDFEVGNRHDLPRINVMNEDASMNESAGKYNGMDRFEARKAIVADLKEQGFLIKVDPMTHSVGHSERTGVQVEARLSTQWFVKMKPLAEMALKNQETDQKVNFVPERFENTFTQWMENVHDWVISRQLWWGHQIPAWYHKQTGEMYVGEEAPEDIENWTQDSDVLDTWFSSALWPFSTMGWPNTEAPDFKRYFPTNTLVTGYDIIFFWVSRMIFQSLEFTEQRPFEHVLIHGLIRDEQGRKMSKSLGNGIDPMEVIEKYGADALRWFLTSGSTPGQDVRFSYTKMDAAWNFINKIWNASRFVIMNLEDTPAPTKVPEAANLDLTDKWILSQLNQTVADVTRLYEGFEFGEAGRTLYNFIWNDFCDWYIEMAKEVLYGDDQEAIANKRYNLAYVLDQTLRLLHPVMPFVTEEIWQSMPHTGESIMTASYPEVHAELDDQEATTQMNALIDLIRSVRNIRSEANAPLSKPIDILINIQDTPLMAIFKQNQDFIERFVHPKSLEIAEGLTAPALAKTAIISGAEVYVPLAELLDLDEEITRLEGELKRLNGEIKRAQGKLANKGFTDRAPEKVVQEERDKQADYEQQYQSVEKRLAELKAAR

>fig|1664.9.peg.467

MLVTDYETTLAYIHSRPRLHKEANLNRMTQLLAALGNPHQGQQFIHVTGTNGKGSVVNMVSSLLVELGLKVGRYTSPFITRFNERIAIDQQPIADDDLIAVTQHVQQIIETVQQADSDFEVTEFELITAIMFTYFKQQAVDIAVIEVGIGGLYDSTNVLTPLISVITSVGLDHQALLGDTLAAIATQKAGIIKAQHPVVIGKLPAEAQTVIVETAKRLASPLAISGVDFSTTAVKTLPTWGQQLNFQNKTVTLKQLQIPLMGDYQIENAAVALETFCQYLALTQQPLDIKTLKRGLAHVSWPGRFEKVNDEPLIILDGAHNPAGVEQLVKTAKHQFKHQDIYILFGALGDKSLAGMLPQLNQLATHLVLTTVPDNPRAATQAQYAAVDNTIPFEADWPVALTTMVSELSADDVLLVTGSLYLISTIRSYFKGVS

>fig|1664.9.peg.468

MQTRGIIFDMDGLLVDSEKVYYQANQLAAQKMGFEVTAEDHQAILGTTDTYLRQYFLQKLGSPELVKQFIDLSYRTVDEIIQDQGVAIKPGLVELLDYCDNHGINRVIASSNFRTMVEDFMQSTGLKPRFNQIVSGDEVTHGKPHPEIFLKALDKLAIPAPSALVLEDSPNGILAASKADIPVIMVPDLIAPTAITNQQTLATVDNLAAVIPFLEK

>fig|1664.9.peg.469

MLTSQSKLQPREQVALYGAASLSDEQLATVILRSGTQHLPVEVVAQRLLQRYPDFQDLEQADLVQLRQIEGIGPVKAIELQAICELSRRIQNQRTLRFGVVASSQMVGQRMIESLAGETQEQLLAVYLDVKNQIIQIKQLYLGALNSSVAHPREVFKWAVQYSAAKFILVHNHPSGQLAPSTQDINFTKRIVNCGQLMGITCLDHLIIGSSQYLSLREEGYLVD

>fig|1664.9.peg.470

MFGLGTRNIGIDLGTANTLVYMDGKDIVLREPSVVAKNTQTGEVIAVGSEARDMIGRTPGNIVAIRPMKDGVIADYDTTAAMLKYFIEKTIGHSSSKPFVMVCVPSGVTEVEKRAVIDATKVAGARDAYVIEEPFAAAIGAGLPVMDPTGSMVVDIGGGTTDVATISLGGIVSSRSIRMAGDKFDDAIISYIRQNSNLLIGERTAEQIKLAIASASPERAKEVESMSVRGRDLVTGLPKTVDIKASDIAEAIKEIVTAIIDAIKETLEETSPEIAADVIDHGIVLTGGGALLKHLPEVISDATKVPVFIAQDPLDCVAVGTGESLKNIEVMKKN

>fig|1664.9.peg.471

MQKFFSNKKLIILLVTLIVCMGFIAMSVGLRNSKKSPSFIRQFGNDVTGITNQVVSVPANGIGKGVDALTDLINTYQENKALKAQLDDLAQTKVQAQTLKEENKNLKKELNLAGTLTDYTTLNASVLSRNPDNWQNSLVINKGALAGLKKNMPVMSESGIIGRISEVNKTNAKVELVSSTGRNSNGFAAVVNTKDGKQVNGIMTGYNSDKKQLKLGQIKTDTAIAKGDKVVTSGLGGLTPKGLYLGKVASVKKDDYGLALTVYVTPAADLNNLTVVTVIKRSIEGE

>fig|1664.9.peg.472

MGTFRLKHRYLIPIYLLVAFYLDGAISSLGEQWLYTPDHTLISRLPMLMFIMVAFVLPEETNLSWYAVGIGLLYDLYYTGMIGMYLFVLPLIIYIVRYLKQYIPDSPFFIGLVDVICLTLLEASIYIMNRLVNLTSMTPVDFISNVLAPTIALNLVFFIFLYLPLKKLLLKLKSER

>fig|1664.9.peg.473

MDAVTLRGRKEGFELNIDAGADFDQALDSIVALLSKIRSEQPNLGNDKIELELTTGMRLLSEQQKKALYQTLEPFPEFDIMSIQSSVMTIEQAQIERQENSIHVEPNVIRSGQEVNYVGDVLFVGNLHQGAVIKATGSIFILGSVAGIVHAGFPDNADAIVAGNLTEAVQLRIADAVEIIDPKKTPFNSQSISYINDLHVIDYGEITDLKQINPKLYRKMEEQ

>fig|1664.9.peg.474

MGTALVITSGKGGVGKTTSSANIGTALALLDKKVCLLDLDIGLRNLDVVLGLSNRIIYDIVDVAKGRAKLHQALIKDKRFDEKLYLLPAAQNTDKEALVPEDVTAIVTALKEEFDFVIIDCPAGIESGFKNAIAGADGAIVVATPEISSVSDADRVVGLLEETEMQIAPRLVINRIRRHMMNDGETMDVDEITKHLSVSLLGIVFDDDAVIKTSNAGEPIVLDPKNSASQGYRNIARRLLGETVPLMTLKEKKIGFWGRLFGKK

>fig|1664.9.peg.475

MQYSLSILPALLSGAKMTLAIFSLTVIGAIPLGLLLSLGLLSKFKPLHYLLNLYVWLMRGTPLLLQLIFVFYGLPVIGIVFNRFDAALIAFILNYAAYFAEIFRGGFQAVDDGQIEAASVLGLTYPQTILKIIVPQTVKIVLPSVGNEVINLIKDSSLVYVIGIGDLLRAGNVASARDVNLVPLLLVGVIYLALTAICTWLLGRLEQYYRYYR

>fig|1664.9.peg.476

MFKLENINQSFKGRQILNDLNLEVETGSILAIVGPSGAGKSTLLRLISGLDQPDSGRFVIDGETYVPSAAEKGGRIGVVFQDFRLFPHLSVLENVTLAPKMVKQLAKDKANALGQQLLRQLGLSAQEAAYPFQLSGGQRQRVAIARALAMQPDILCYDEPTSALDPSLVASVAELILSLREDGMTQIVVTHDIQFANKIADQVFELQPNEANQ

>fig|1664.9.peg.477

MMRLKRLATAGLLLGLLVSLAGCHSRQKTDSWATIERRQKVVIGIDDSFVPMTYREKNGQLTGFDVELAKAIFKGSGIQVDFQSIDWDMKETELNNQTIDLIWSGYSKTEERAKRVAFSQPYLTNHQMIVSMKQNPVNRLADLKGQNLGVQTGSSGMAQLDRRPTVLKQKIANQTPILYDTYNNAFIDLQAKRIKGILIDQVYANFEIKARANGQDFQAIESPFAAEKFAVGMRPEDQKLRRFINRRYQVLKQNGQLAKIQAKWFKE

>fig|1664.9.peg.478

MTTIIIIILSLILVINTIGAIITVFRQSRDIAATWAWLLVLILLPVIGFGFYLFFGKKLSAEHLYDLRTQAKLGIDERVTQQKAELRQRRKQNHHSTTPEIQSLINLFLVSNTAVLTQENKVSVLTDTTTAQERLLADMSRARDHIHLEYYAFFDDPFGHILIKTLAYKAYQGVKVRVIYDGFGSQKIPRSFFKPLEEAGGQVTPFFSSRFRLINFRLNFRNHRQLVVIDGNTAYLGNFNQHSVDTHLPVRDTRLRISGDGVLSFQSRFFMDWNAATQRQKVYYSQHYFPTSVEHGQTSMQLVSGGPDRDLPEIKLGFLKLIAAAKKRLWIQTPFFIPDDSVLAALILAINSGVTVKIMIPEKAKHSLVQHANLYYARQIVKAGGQIYLYKASAFRARTMMVDGQLSAVGTANLDIRSFKLNFETTSFLYDPALTNTLELTFQRDLKASVPLTKQQLNAQTTNQRLSQDLARLLAPIL

>fig|1664.9.peg.479

MQKPLNWIKRYPAVLILFILVGVGLFATHVLATYQQITIKANVVNVRQGPGLSYDTMGQASKGEVMNVISQKNNWYQVRLSGDKIGWVASWLVNNTEVSATSNRVATVTNDFANVRQSSNASSPLLGKVNKGDKLTVLYQQNGWSQVKYNSAVGWVQSDLISISNEAPTAVQTDTKTDDSSSQSTSDIKSVTTQLDNTKLRSGPGVNYTYSQVYSANTKLTYLDKSDTWYKVKDADGNTGYVASWVVTPSAKNEVVKTSATSLSEATIVLDAGHGGNDVGALSNSNKYEKTYTLKTVDAIAKKLKAAGANVVLTRSSDKFVDLAPRPALSNKLRADAFISIHFDSSAQSNQASGTTTYYYSNSKDTSLANALNNQIKSLPLTNRGVEYGNYQVLRDNERPSVLLEMGYINSDQDFNYISSSSYQEKVADAVYAGLQNYFK

>fig|1664.9.peg.480

MGYQKPKGTADILPGESDQWQLVEKTARDVFARYQFKEIRTPLFESYDVFSRSSGDTSDIVSKEMYDFMDKGDRHIALRPEGTAGVVRAFVENKLYGPEHQKPYKVYYMGPMFRYERPQSGRQRQFHQIGVESFGSESPAVDVEVISMAMRLLREFKITDLKLAINTLGDAASRAAYHEALVNYLEPHFDELSDDSKVRLHKNPLRVLDSKDAKDQEIVKDAPVILDYLTDEAKVHFNTVKTLLESLNIPYEVDTEMVRGLDYYNHTIFEIMTNNKVLGRGYTTVLAGGRYNGLVEQLGGPDMPGVGFGLGVERLLLLMNAQNSDLVAAPVLDAYVVGIGAETSATTLHLVESLREAGLTADRDYLDRKPKAQFKTANKLNAQFVVTIGESELADKTAHVKDMTSGVEITVPLTMLEADFAAVKNELKAQEENA

>fig|1664.9.peg.481

MKTRTTYSGLVDETFVGQTVTLHGWVQKRRSLGNLIFVDLRDREGLVQLVFNQDNADILTLANTLRNEYVIEVTGTVQARDEAAINPDMKTGKVEVTVSELTILNEAKNLPFEIKDGITTSEETKLKYRYLDLRRPEMQKAIIRRSQITQAAHRYLDNNGFLNIETPDLTRSTPEGARDYLVPSRVYPGSFYALPQSPQVFKQLLMDAGFDRYYQMARCFRDEDLRGDRQPEFTQIDVETSFMSAEEIQEQAEGIIKQVMQDVMHIDVPTPFKRMKWQEAMDRYGSDKPDIRFDLEIQDLTEMMKTSSFKVFSDTANSGNLVRAIVVPEGAAKYSRKMLDEQTEYIKRYGAKGMAWVKVVDGELTGPAAKFLKEQEAELLSALSANDGDLVLFVADKFQVVCDSLGYLRKHFAHDMGLVDESQFAYLWVVDWPLFEYDEGFGRWIAAHHPFTRPNDSDVDLLETDPHKAHAQSYDIILNGYELGGGSLRIYQRDIQERMFKALGISPETYEEQFGHLLSAMDYGFPPHGGFAIGLDRFAMLLAGRDNIRDVIAFPKNSHASEPMTNAPSRVAPAQLDELGLEVEPEVEKD

>fig|1664.9.peg.482

MSDDLRQQLTPEQYAVTQENATEMPFSGEYDEFDQLGIYVDVVSHEPLFSSLDKYDAGCGWPSFTRPITKLNEKRDQTHGMERVEVRSQNADSHLGHVFTDGPQEQGGLRYCINSAALKFISKDELKAAGYGEYLALFEKEGQ

>fig|1664.9.peg.483

MATETAIFAGGCFWCMVHPFDQQPGIIKVVSGYTGGHVANPTYEQVCSHTTGHTEAVEITFDPAVISYAELVEIYWRQTDPTDAMGQFQDRGDSYRPVIFVNSAQQRVIAEASKEKLIASGRFNEPIVTQIEAAKPFYPAEEYHQDFYKKEPWRAELGEVKRKASIKEKWSD

>fig|1664.9.peg.484

MAKVSAAILYGICVSIALNFFWEEGGVYASGITGFAQFIHTVTAKFMPFLPFPLSTSLMLFLLNVPLFVLAWFKIGHHFTIYTFIAVIFSSLMVQIMPLTQLTDNPIICAIFGAVINGFGTGMALKNDISTGGLDIIGIVLRKKTGRSVGTINIIFNVALVFAAGFIFGWEHALYSILSIFVNGKVIDMVYTRQQKMQVMIITDNPKVVIQMIQDEMRRGITIVHDVEGAYNREEKTMLFTVISRFEMHDLEMVMKEADDHAFVSITEAVKILGHFYEPKIK

>fig|1664.9.peg.485

MLLGSHVSMKGKEMLLGSAQQAAEFGANTFMIYTGAPQNTRRKPIEELNIQAAQELIAAKNLGPIVVHAPYIVNLGNTVKPENFKFAIEFLQQEVIRAEALGASQIVLHPGAHVGAGADAGIQQIIKGLNEVLRPDQTAQIALETMAGKGTEVGRRFEEIAQMIDGVTLNDKLSVTFDTCHTSDAGYNIREDFDGVLNEFDHIIGLDRLKVIHLNDSKNPQGAHKDRHANIGFGEIGFDALHGVVTHPQLVDVPKIMETPYVGEDKKHNFAPYAYEIAMLKKGEFDPELLTKIEQNEGRL

>fig|1664.9.peg.488

MSQSTPLTVFVLSDSVGQTALQLAQAALAQYPNIKPDIIRFPFVHSVDKLTDVLSKAVPGETIVVHTLATTGLSEIAQNYCDAKKIASFDMMSPLSKLITAQTGLAPSGEAGALHHLNERYFDRISAMEFAVMYDDGKDPHGFLEADIVLLGVSRTSKTPLSLFLANRNIKVANLPIVPQAHIPDEIWHVDPKKIIGLMNTPEVLNNIRRERMIAYGLNPDTTYSDMDEIKAELDFAQDLYDKIGCQVINVANRSIEETAAIILEQTGLDFASSSTDHVN

>fig|1664.9.peg.489

MGKTVVRSNESLDDALRRFKRSVSKAGTIQEYRKREFYEKPSVKRKLKSEAARKRKKF

>fig|1664.9.peg.490

MSLLEQLSSDLKTAMKAKDKLRLTVIRSLKTALTNAKISAGQDLSSDEELSVLSSQVKQRKDSLAEFEKGDRADLAEQTKAEIEIVQAYLPEQLDEAAVIVIVDEAMQATGATGKADFGKVMQYVMPKVKGRADGAMVNQVVKSKLS

>fig|1664.9.peg.491

MVEVEERLFQLATPNLAAALMGAHDHFLKIIEESLTVELIAFDDHITIRGTQPGLRQANAVLESLVALLQHGVILKETDVVSATKMALKGTLNQFEALYTEPLINDRKGQPIRVKTFGQRQYIDAIRHNDVTFGIGPAGTGKTFLAVVMAIATLKKGTVERIILTRPAVEAGESLGFLPGDLKEKVDPYLRPIYDALYAVLGKEHTERLMDRGVIEIAPLAYMRGRTLEEAFVILDEAQNTTPAQMKMFLTRLGFNSKMIVNGDISQIDLPNKTQSGLVQAEQILRDIPKISFVQFSAEDVVRHPVVARIINAYAAMDKTRK

>fig|1664.9.peg.492

MDIQIIDETKIVSEAQIKLVEDVLEFAGQKLELAEDTEMSVTFVTNERIRQINQEYRNTDRATDVISFAIEEDPEEEGLPANFEELFDIPKNIGDLFVSLEKAAEQAETYGHSFERELGYTMVHGFLHLNGYDHIHTKDEVKMIPLQETILDEFGLKR

>fig|1664.9.peg.493

MNSDSNAKKTQTDKNRNFNQSTHHALDGIITAFKEEANLRRDFVIAMVVLVAGYFARLHYLDWLFLILAIFMVLLAEFWNTVMEHFVDLLVDRQFHPLAKKIKDISAGSVLIGACLAVIIGCVVFGHAVLYYF

>fig|1664.9.peg.494

MSDTYRSGFVAIVGRPNVGKSTFMNRMIGEKIAIMSSKAQTTRNKIQGIYTDDNAQIVFVDTPGIHKPHNELDEYMDQAALSTFNEVDAILFMISGVDKKGPGDQYIMDQLKNVKKPVYLVVNKIDAIHPDDLLPMIEQYRHELDFKAVYPISALEGNNVPEMLKELELTLPEGPQYYPGDQLTDHPEYFVVGELIREKILELTHEEVPHSVAVVVERMKDRVNGKLQIEANIIVERDGQKRIIIGQKGSIIKEIGIRSRREIEALLGEKVNLKLWVKIQKNWRDNNQYLREFGYNKKRL

>fig|1664.9.peg.495

MAEHRFEDFNGLVMYRKNYKEKDMLVKILTDRFGKKMFYLRGANKPKFRLSAAILPFTQAEYGGDIRDDGLSFLNNVKSATQFQTISQDLFLNAYATYILNLIDVGFPDSEPLGIWYDKVEQALNLIDEGFDAAMITHIIEIQLLQVFGVQPQLQGCAVCGRTDLAFDYSESYGGLLCQRHWHLDPNRFHSSQRAIYYLRLFSVIDLFKIQSVNVKEATQVELKMIIDRLYQDTVGLSLKSKQFIDKMYSFDSQLPQLKKVPSEIDSTPKDD

>fig|1664.9.peg.496

MVKKLNVQEMILTLQRFWSEKGCMLMQSYDTEKGAGTMSPYTFLRAIGPEPWNTAYVEPSRRPADGRYGENPNRLFQHHQFQVIMKPAPLNIQEFYLDSLRALGIDPLEHDIRFVEDNWENPSMGCAGVGWEVWLDGMEVTQFTYFQQVGGLQCDPVTSEITYGVERLASYIQDVDNVFDLEWGNGVLYGDIFKEPEYEHSKYAFEDSNQEMLLKFFDEYETEAKRLIDLGLVHPAYDYVLKCSHTFNLLDAHGAVSVTERAGYLSRIRRMAHSIAQAFVAEREKRGFPLLKHAQEAKTEVVDHD

>fig|1664.9.peg.497

MTKPFLLEIGLEEMPAHVVMPSIQQLEKRVRDFLEEQHLEFGQLKTYATPRRLAILIEDLADKQADIHEEAKGPAKKIALDAEGNWSKAAQGFVRGQGLTTDDITFKELKGVEYVYVEKSIIGKPVEAILPELKAVIMAMTFPTRMHWADYDFEYIRPIHWIIALLDDEVIDFSILNVTTGRTTQGHRFLGQEVTLAKATDYEAALETQFVIADEAKRQNLIQTQIEKMAQENDWQVDLDANLLEEVTNLVEWPTAFYGQFDAKYLEIPEEVLITSMKDNQRYFYARSQAGDLLPVFIGVRNGNDAFMENVIAGNEKVLTARLEDADFFYHEDQKVTIAENVAKLQNVSFHDKIGSLAEKMTRVGLIAQFLGQKFGLSEAELVDLQRASDIYKFDLVTGMVGEFAELQGVMGEKYALLQGETPAVATAVREHYMPISAEGALPATKVGAVLAIADKFDSMADFFAVGMIPSGSNDPYALRRQAYGIVRILENQDWHFPMADFQAAMSHIVKQQADFDLDWSANAAAISDFLTDRVRQRFNGMRPKVRRDIVDAVLKNQGQDPVVMFEAAKVLSAHQDDADFKATIEAITRVLRLAEKVDFETTDLTVDPALFENDAEKALYTAVEDLRGATATNTLAENYAALQTLRPLIEAYFDATMVMAEDEAVRRNRLTQLMIIAQIALSLGDLNELIVK

>fig|1664.9.peg.498

MLQMKRVYEAAADSDGYRILVDRIWPRGISKERAEIETWAKEITPTTDLRKWFGHEPAKFPVFKEKYLAEIATNPDWPEFCHLVQHQLALGDVTLVYAAKDPQYNHVVILMALLKKTLDK

>fig|1664.9.peg.499

MAGKIPNEVIDEIRSQTNIVDVVGQYVQLKKAGKNLFGVCPFHDEKTPSFSVSEEKQIFHCFSCGRGGNVFKFLMELEQISFPEALTKVADFAGVTLADSYKPTAVHRESSEVTQFKQLYQQANELFKHILTSTVAGQPALDYLHERQMTDALIETFSIGYLPDQSDLLLTFFQNKDIPYQVLRQSGLFIETQTGKLHDRFSGRVMFPIRNAQGEVIAFSGRVLTKQPDQPKYLNSPETVIFNKRKVLFNYDLAKQTIHQSKKVYLFEGFMDVIAAYSADVVNGVASMGTSLTTEQLNLLAQQAQELVVCYDGDQPGIEAMKRAINLLQQHTTLELSVVVLPGGVDPDEYVRQYGGPQFKETLQNGTETPIAFELRYLKQGLNLDNEKDQLDYVQQALEVVARVESPLAIEVYLKQVEADSGITLDTLKRQLQTVRVKHATQVPINQVGQPEAPPYFDDFQAPPLPPEEMAGAFGDPRTIAPKRQPKRLTRIEHAEQEILHMLIHNEDVRLQLENNADFSFVHTPYQLLYELWRSFIAEGQPADIAGFTSYIPDDLQELVVQIDLLDLPEEANQEALNDCLAVIGQNSIQERLKEAKIALKEATKLGNHSEELRLTNEVIQLVSQMK

>fig|1664.9.peg.500

MADNKKADTKKLDTAVKALIKEYKPKKEITNDELVAKIVKPFALEADEINELMQQVEDHGIGIVDEDGNPSAATLKKEKVSAKELKDMSAPTGIKINDPVRMYLKEIGRVSLLNAQQEVDLALRIEQGDQEAKQRLAEANLRLVVSIAKRYVGRGMQFLDLIQEGNMGLMKAVEKFDHRKGFKFSTYATWWIRQAITRAIADQARTIRIPVHMVETINKLIRIQRQLLQDLGREPTPEEIGAEMDMNTDKVREILKIAQEPVSLETPIGEEDDSHLGDFIEDQDATSPEEHASYELLKEQLESVLDTLTDREENVLRLRFGLDDGRTRTLEEVGKVFGVTRERIRQIEAKALRKLRHPSRSKQLKDFLE

>fig|1664.9.peg.502

MSPINLSKRLAAVAQFVPQDSIVADIGSDHAYLPIWLTEQKRLKGAIAGEVVAGPFESTQTHVTQWHLEDQIQVRLGDGVEVLNPEEDQVTCIVIAGMGGLLITEILECGLAHLNGHERLVLQPNIMEPTVREWLMKHHYAIVEEAIVAEDGHIYEVIVGEPRTEAVELDEADLVFGPVLRRRQPIDFQLKWSTLLNKKRHLLVQLQGAQVTPTEKIATVQHEIQLIEEVQTWDN

>fig|1664.9.peg.503

MGQLIASDLIAAIEEYAPLVLKEGNDPTGFQIGRRDKVVKKVLVTLDVRPEVVQEAIDNEIDFIFAHHPVMFRPARNLDLSDPQNKMYADILTHDMTVYAAHTNLDKAQGGMNDWLAAALALKNVQPFNVTDYEALMKLAVFVPETHADVVRQALGQAGAGELGNYQNCSFSAEGTGRFEPQTAADPFIGQAGQAEAVTEVKIEVILPAAKKNRVLKAMLAVHPYEEPAYDLIPLANQQQPIGIGRIGQVDQPMTVRDYAQFVCETFDLTGLRLISNEPGKLVETVAVVGGDGGKFFPAALQAKADLYITGDVYYHTGHDMLAAGLSVIDPGHHIESIVKTKMTALFEEWRQINDWQVTFIKSQQKTDPFTFIFNAK

>fig|1664.9.peg.504

MAKYEKLIPRFLEYITTETRSDENATTIPSTQTQVVFLHKLMDDLKEIGLSDVKYNEKNGYVTALLPSNIDKKVPTMGFLSHVDTADFNAKDVNPQTIKNYDGESIIKLDEAGQFVLDPKEFPNMKNYKGQTLITTDGSTLLGSDDKSGVAEIITAMDYFIQHPEIKHGDIKIGLGPDEEIGTGADHFDAEDFATDFAYTMDGGPIGQLEYETFNAAAMKVDIQGKNVHPSEAKDIMINALQVAVDFQDAFPRDEVPEKTDGRQGFYHLLSLDGTVDEAHMAYIIRDFDRDGLETRKAFAAKVAEDMNAKYGEGRVKATIKDQYYNMAEVLKDHMDVVDLAKDAMEAIDIKPLIEPVRGGTDGSKISFMGIPTPNIFAGAENMHGRYEFVSVQTMEKAVDTMIKMNELNVERN

>fig|1664.9.peg.505

MHLEFTTGQTQTAVSLIPRHLDDCLRELILINQKKDAIDILEWRLDYWQAPAQLLTAAEKIAALDLPLILTVRTTNDGGLASAQDYLTYYAPLIKAHIGQAIDLEWSLAAEQRHQLAELAHQQHYQVLLSHHDTVQTPDNDTLRTQLLAMQADPDADLLKLATTAQSPADTTRLLAATQSFTHQFDKPLTTMAMSEFGVASRIFGGQFGSAISFGYLETPSAPGQLPIEQLKGLLTTNQA

>fig|1664.9.peg.507

MQIMNHTLTTNHQDFQVTAYWLDSNQDFNNTGYQPVVIICPGGGFRYHSQRETEPIALKALAEGCHAVVLPYQLIEPDQMVYPTALQQLAKTIEWITDQSDTQKVDPKRIVLVGFSAGGHLVATFNGIATNPELNTQYQLDSYRGHHAAVILSYPVIDLDAGFPKEESEKERITTDQRLWHAQDTLTKWAKPCFVWQTATDELVPAINSLLYVTELNRLNITTEYHLFSDGVHGLALANKVTQRPGKPQDVNEPVAQWLPLALTWLAEIDIAHR

>fig|1664.9.peg.508

MISGYTALYGLIAHPAQHSLSPFIHNTGFHQIQMDARYAVFDSQATPAAIASAIKTLGIRGVNLSMPYKQSLVPLVDDLTPTAKLVGAINTIKNEAGHLIATNTDGDGFWRALQKAHPQRRYRSVTILGAGGAALAVIEAAVRYGVQQVTVFKRANATYDSVIQRLAQISLASGLQIIVEPYDDQLALATALQNADCLINATNIGMAATPGNPLPINLLQYLPEDSLVADLIYAPRETAFLKTAQKAHYQIQNGLGMLIEQAALSFEFWTNESMATTPIYQHLNGGNDAS

>fig|1664.9.peg.509

MIIQFKATTSKSLIQYFCERQRQAGVVVYQSAQHVALIGLKEVTLTAAEQTGVDSMTTAEPSYVLSSRLFQPENTIIQLPHSTIGGEQFTMMAGPCSVESAEHILAMAKVAKEGGATLLRGGAYKPRTSPYSFQGMGEAGLKALRQAADATGMDVITEVMDETHVALIAQYTDVFQIGARNMQNFSLLKAVGQTNIPVALKRGLAATVDDLLNAAEYIAANGNRQIMLLERGIRTFDNQYTRNTFDLNAVPVLQKLSHYPVVVDPSHATGEWDLVTPMARAGVATGASGMMVEIHDHPEVAFSDGPQALKPATYLEMCRQIWAIDQLMRSW

>fig|1664.9.peg.510

MPTISVNLTTQEYQIKIENGLATSIGREVQQVWSARKIALVTDTNVGPLYQAQVAAQLTQAGFQVTVLTVPAGESAKSLEQAMSLYETLVTANFNRSDGLIALGGGVVGDLTGFVASTYMRGLPFIQIPTSLLAQVDSSVGGKTAVDLPAGKNLVGTFYQPELVLIDPQMLETLPQRQLVTGCAEVVKVAALVGADFWNLVQQIESPTAILDKAPELIARSIAYKTQIVMADVQESGQRRLLNFGHTIGHAVESLADGELTHGEAVSIGLIAISRLFEQPTQIAAQLQTVLERVGLPVTHPLLQSPALFEKITHDKKNQGALINIVYLKAIGQPTILQLPLTQFSAQLKMKQRSF

>fig|1664.9.peg.512

MTFKLPALPYEYVALEPYIDSETMHLHHDKHHKTYVTKLNAALAKHPDLASKSLHDLLTHIDDLPESLQTPIRNNAGGHANHSFFWRILTENAPLMPTGELLEKIEARFGTFKDFQAEFNNAALGVFGSGWAWLVVDHDDELQIMTTPNQDSPIMSGNRPLIGLDVWEHAYYLKYQNNRIDYIENFWKIINWPLVVELI

>fig|1664.9.peg.517

MNAQLKKGLLEFCVLATLKKADSYGYQIIKDTSSVIEISDSTLYPILKRLEKQEQIESYQVAHNGRLRKYFHLTPIGAAAIEQFLNEWTQVTAIYEYIKGEA

>fig|1664.9.peg.519

MITSETVYHFVGIKGSGMSALALVLNDLGYQVQGSDIDQYTFTQRDLEKADIKILPFSADNLHEGLTVIAGNAFQDDQIEIKTALEMGLPVVRYHQFLGELLKNYTSIGVAGAHGKTSTSGLLAHVLSGVAKTSFLVGDGTGKGIKDAQFFAFEADEYRRHFLAYSPDYLIMTNIDFDHPDYYTGIEDVFDAFQSEAYKVQKGIFAWGDDPELRKLKSNAPIYYYGTKSNDDFQARNIQRTVEGSEFDAYYHDENIGHYHVPLYGEHNVLNSLAVIAVSYLEKIDGREVARELADFKGVKRRFTEKKVADVTIIDDYAHHPSEIKATLDAARQKYPDRQIVAVFQPHTFTRTIALMDDFAASLNLADEVFLTDIFSSAREKSGKVSSADLGAKITKGGRVLKLDNMSPLLDFEQPVVVFMGAGDVQKYEYAYEELLSNLSPKDN

>fig|1664.9.peg.520

MCGIVGFAGGLFDLPKKKEVLNQQMDVIKHRGPSGAGDFIDDKVALGFRRLSIIDLTKGDQPIYNEDHTKLIFFNGEVYNYREIRKELEPLGHEFKTDSDTETILHAYEQWGKDVLKKIRGMFVFVIYDLQTKTLFGARDFFGIKPLYYTTLDDGTFMFSSEIKTFMRHPEFKRTLNKKALKSFMMNQYNDLQETFFEGVFRFPAGHYFTYKDGQLDIEKYWDMQFDPSNLSFEEEVKKIDASVEESIKMHNVADVPVGAFLSEGVDSSYVTSILQPQEVFSVGFDDQTYNEVTAAHALADELGLNFNETKVKADDAFAEFDKIQYYLDEPDGNPSCVPLWFLSRFAREKVTVALSGEGADELFAGYVNYGMHSHNTTIKAFTSSLKKLPKGARAKLAKTVKKMPAFPGRVHLYTNLANPKEFYAGQSVIYDIENPSIFGSDEANQYLKPAYQNDTTIQENYQADFDRVKDADPVSQMQYIDMHHFMLNDILQKADKLSMAHSLELRVPFLDPEVAKTAGQVPTEYLINSKDTKYAYRQASAKHLPAAWANRPKLGFPVPIKQWLETDKYYEQVKALFSEDFVSEFFDQGQLLALLDANKEQRINGRRKIWTIYTFLTWYKVYFINIEDYLVLG

>fig|1664.9.peg.521

MLIASMNQKSFPNILVVMTQPDVANQVTTEKQSIVRISDQADQTIGYNFLAIDQLLPELQFNGVQALSVADVATLNQALEAAGFTPDLVADETPHIVVGYVQECVVHPDSDHMHVTQTQIAPDQTVQIVCGAANIAQGQKVIVATVGAVMPEGKIIWPGQLRGVQSDGMICAAREIGVEGAPAKGIMVLPDDWEVGQAVTADAVAALLK

>fig|1664.9.peg.522

MKEFAKMAETNLTHLITKGKHVLFFTATWCSDCRFIKPAMPEIEAEFPEYEFIEIDRDQYIDICEEWEIFGIPSFVVIDNGREVGRFVNKDRKTKREISEFIHSLS

>fig|1664.9.peg.523

MKKNSQTLKYLLTGFGLAVAAAAGTRQYKTQQKMVKLDKILEDVKASLRHEGQIVGSWIEESPHLFSQNKRTFPVYQGGIICERDEGTNQFHFKVDAKTGVVIEFSQL

>fig|1664.9.peg.524

MRLRNKPWAADLIAENPDMILVSPENIASNWQSRFAKEQPIYIEVGSGKGQFITQMAQKCPDRNFIAVEIQESAIAVILQKQVELKLPNLQLLLGNGAALTTFFAENEVAGVYLNFSDPWPKTRHEKRRLTYKSFLAEYQQIMQPTGYLRFKTDNQGLFEYSLASLNAYGMVFDDISLDLHNSDLAEDNIQTEYEEKFSKRGQVIYRLEAHYK

>fig|1664.9.peg.525

MNFDLDTGWQVRSAGGDTGAAYLGVRASEKIFLKRNTSPFLAALSAEGITPRLIWTKRISSGDVLTAQEWLNGRTLKRDEMNSPQVAKLLARVHQSHLLKRMLQKVGGQVWQAPEMLQTYFYDLPQDLRQHPFLAQTSQYLKDTMSALQTPSLEVCHGDLSHKNWLLSDINRLYLVDWDSAMLADPAVDFGMILFQYVPRQEWQEWLENYGLEVTPELLMRVNWYGSLNLLQSIKHHYQRGRFTEMNHDILRLEEVLSEIIKKN

>fig|1664.9.peg.526

MRQLWQKRVRQHIQEQVKYLKLVFNDHFVLALIFLLGALGYTYANAVKGLDPQAWWLKLLLSLVLLAAVSFGRFATLLKEADSVFLLPKESDLPVYLKAARGYSAWLPVIFTGLVTGLIAPTLLITKSVPSWQLLLVWATLIAIKDRRFSNQLLNWYQIDAKLPKIVWLLVDWLLIGSQLFSGLAIAGVLIAIGLAYYQRRQLTTIQQTTLFDWLAAVSAEDSRMGRIYRLYNLFTDVPGLNSQVKRRRYLDWLLPVAKRSGNPYLYLYTRGFIRGTEFSGLYVRLVVLGGIILCFSHLWWLSLALGLLLIYLVGFQLLPFYTQYEQIIFTHLYPIAKQARVKAFQQLLLGLLLCQAILFSVILLVTMGLDLQAGLSVLALFALVGVFVQFYLPQRLVK

>fig|1664.9.peg.527

MSLEINQLTGGYAHVPVLQEVSFTVPNQQVVGLIGLNGAGKSTTIKHIIGLLMPQKGTITLNGVTLQSNPDQYRQSMAYVPETPVLYPELTLREHLELAMMAYDLDFEKTWAKAMVLLKKFRLDNKLDWFPVHFSKGMRQKVMIVNAFMTDADLFIIDEPFTGLDPLAIHDLLDIIADKKAAGAAILMSTHILATAQQYADSFVLLNQGKVRTTGTLEELKAEFNMADANLDDIYLQMTKEG

>fig|1664.9.peg.528

MDDCIFCKIVRQEIPSTVVYEDDVVKAFLDITQTTPGHTLVIPKQHVANIFEYDADLAAAVFSRVPKIANAIKASNPAIKGMNICNNNGEVAYQSVFHSHIHLLPRYTAADGFSMQFADNSADYTPEKLQTIADAIKQQLEA

>fig|1664.9.peg.530

MMKKWLLAAASLLMVVTLAGCGSNTIATLKGGKVTQDEFYKEIKETSAGKQQVQQMILQKALQEQYGSKSLTKKIDKTYNTYKKQYGSSFTSVLAQSGLTTSSFKNQITTQMLANAALKANKKVTNADLKKQWKTYEPKVEVQHILVEKKDTAETVISELKKDNSTKNFTALAKKYSTDTGTKKDGGKLPVFDSTDTSLDPTFKTAAFKLKTNEYTTTPVKTSYGYHVIRMIKNPGKGKMADHKKTLTDQVYAKWANDQTVMAKVYTKVLKKADVTIKDKDLSDILSSYGVNAKKSSAKSSSK

>fig|1664.9.peg.531

MTTKKLFDYQNDENLALFVLIKAADVRVAKNGKQFIAFTFQDQSGQISGKYWDASDEDVALFTPGKIIHLQGKRELYQSNPQIKIYKLRLATEEEPQVIEQFIQKAPVQTADMEEEISQVLFEITNPNWQRLVRHLIQKHQADFFSFPAAKTNHHAFAGGLAYHTLSMLRLAKTIAAQYPQIDAALLYSGVLLHDLGKTLELSGPVSTEYTVRGNLIGHIVLVDEEIVKACETLKIDINSEDMLVLRHVVLAHHGLMEYGSPVRPQILEAEVLHQIDEMDASITMMTQSLQHAEAGQYTERLFAMDGRHFYRPVNDQQLTQD

>fig|1664.9.peg.533

MKFIHAADAHIDSPFVGLKKASTNLWTTIHESTFTAFEKLVQTAIDEAVDFVLLVGDSFDQEAQSLQVQKFLKQQFERLNAVEIPVYLSYGNHDYWDPNQIHFDFPKNVHVFTTAVEAKTLTTKAQETVTIVGFSYGQRWLETDQTTQFPPRAATTYTIGTLHGSQSTTSDADHYAPFTLSELNQLQYDYWALGHIHKRQILQERPYVIYPGNLQGRHKNEPGEKGFYLVTAQAGQFELEFKPTTVIEWQRQRFEIPENPSLNQLQEALSQQLAQSETASLVALELTHAERLSSDLIQRVNNGELLAYLQAALESSAVMHWPYALKLQFAAQQQFSQIDQAYWQSAADKIFTPDYILEKAQPLLKEKFIREALEEPDFQDDLKTAAETILQEKKLED

>fig|1664.9.peg.534

MTVNIYDDANQMATNLQTLPQFLGLQNAFAALKKDDIAFTMFKKFQTQQMTLQQKQMQGEELTDDEIQEIQELAQKIGDIDAIKNLMEQERQLSQLMDELNQIISKPIADIYQG

>fig|1664.9.peg.535

MNKQDSPIWLAIKRFFKRAWRVVKYVWYRLQLTRWLILGILLLVFFMSSYLTFKAKTANVGEIKANLQTSTTLYDKKDARAGTLYTQKGTYVELDKISPEIQNAVISTEDRSFYTNPGFSVKGLARAAVNLVIHHGQITGGGSTLTQQLAKNAKLSQKQTFSRKFEELFLAVQITKTYSKKDILAMYLNNAYFGNGVWGVQDASRRYFGKNASELDASEAAVIAAMLRSPSYYNPADHLDNATSRRNLILGLMVDNGKLTESQAKAAKSETLVVKNTLEQNDSYKYPYYFDEVVNEAVKRFGLKEEDLVKNGYKIYTALDQSQQSKMENTFEQNWLFPDNAADGTLVQGSSIAIDPKTGGVSAVVGGRGDYTIRGYSRITQMKRQPGSTIKPLAVYTPALEDGYHYDSSLQDKKKSYGTNKYTPTNPDNVYTGSMPMYQAVANSTNAPAVWLLNQIGLKRGIKSVEDFGISVPKSDQNLAMALGGWQGGVSPYQMASAYTAFANNGELSETHFIRKIVDASGAVIVDNTTPSKSRIMSKKTSKEMTSMMLGVYNSGTGRYAKPDGYQVAGKSGSTEVPDSYGYGTKDQWLIGYTPDIVVATWMGYDKTDQEHFMKNTSIDGLAPIFKYEMQQLLPTTAQTNFDTEDAQSMAKKEDDQDSDKVVNSLQSGIKDGVNKAKETVNQWYNNLKGFFN

>fig|1664.9.peg.536

MIIYQKKVTLETDFSPMSVRGLLNHWLVPQKMQHFLRINREILVNGQYRAFNRNVEPGDEITLNFSEVSVKVQNYELDATQSFSVLFESDDLLIVNKPAGIKTHPNRPGENGTLMNQVAFYLAQQGKKAYMVHRIDEATSGVLLIGKTPIVIGILNRQLSQKKMARQYIAIVNDPEHELAEHATINSPIGIDPENDRQRCVDYINGLSAITHYRVLSRKGATAVLAVDLETGRTHQIRVHLASIGHPILGDPLYNPTDTTPRLLLHGQTLRYTEPYTDQPRTVTAPVPDAFEPYLS

>fig|1664.9.peg.537

MTHLNTWQQLLYYFQHNGGYVLSQFNRHFLISIYGVLLAAIIGIPIGILIAHRKKMSDVVIGIANVIQTVPSLAMLSIIMLGLGLGTNTVIMTVFLYSLLPIIKNTYTGMINVSPNILDSGLGMGMTKWQLLTMVELPLSLAVIMAGIRNALVVAIGITAIGSFVGAGGLGDIIIRGTNATDGGALILAGALPTALMAIIADLVLGFIQRRLEAPQAHH

>fig|1664.9.peg.538

MLKRLKKITVLTLLGGCLLLLGSCGLPGLGGSAKDTIRISSQSTTESQILANIVSQLIAHETDNKVELVNNLGSSTVTHESLIRGDADIATSRYAGTEITGTLKMKPTKDPKKAQTIVKREFKDRYQQTWFPTYGFADTYAFMVTQQMAKKYHLETVSDLGRVAKQLTAGVDSVWVNHEGDGYQEFKQTYGYDFKKVAPMQIGLVYSALAAGKMDVVLGYSTDGRIKSYNLEVLKDDRHFFPPYDASLVATDQILKQHPELKPLLNRLSGQINLDTMQTLNYKVDNDLLEPAVVAKQFLTQHDYFKKGANK

>fig|1664.9.peg.539

MLNFLNQNGADLLIKTWEQLYISAIALLLGALVAVPLGIALTRVPKTAKVVIAIASMLQTVPSLALLALMIPLLGIGKVPAIVALFIYSLLPILRNTYIGMNDVDPVLKDSAKGMGMTPLQSIMQVEVPMAMPVIMSGIRLSAVYVIAWATLASYIGAGGLGDFIFNGLNLFKPDLIIGGTIPVTILALLVDFLLGRLEKRMTPVAIR

>fig|1664.9.peg.540

MAHYLEFDHVSKIYRGGNKAVDDISFTVDQGEFICLIGTSGSGKTTTMRMINRMINPTHGTITLAGKSLADTDPVKLRRKIGYVIQNIGLMPHMTIRDNITIVPKLLKWSDEDREKKAQELLKLVELPADFLDRYPSQLSGGQQQRIGVIRALAAGQDIILMDEPFGALDPITREALQDLVKQLQVEMVKTVIFVTHDMDEALKLSSRIAIMDGGKIIQYGTPNEILRQPANDFVKDLIGHDRLLEAKPNVETVGQIMLKTPVTITPDQSLSSAIKLMRQRRVDTLLVVDDQQLLQGLVDLENIDHHYKDAGTVQDIMSTKVNFVNQTDLVRDTIERILKRGWKYVPVVDDDHRLVGIVTRTALVDVVYDAVWGDDEPDNATD

>fig|1664.9.peg.541

MMKKVDRQTRIEQIINQQVITTQEELLATLRDDGIDATQATISRDIREMQIVKARDVNGELRYTVFHDDERTQEQRLNDKLREVALTITQVQVMNVIKTVPSNGNLLAALIDDLGYEDVVGTLAGHDTIVVISPDEAHAADLNQRFSAHLAGTNRN

>fig|1664.9.peg.548

MTKFQAQGLPLLNGPRVTGDGYYEAVVQDPEQNLIELTV

>fig|1664.9.peg.550

MSIITDILGREVLDSRGNPTVEVEVYTEDGGFGRAIVPSGASTGEHEAVELRDGDKSRFGGKGVLKAVENVNGPLAKEIVGFDTTDQRGIDAAMIKLDGTENKGKLGANAILGVSLAAARAAADELGLPLYEYLGGPNAHVLPTPMMNVINGGAHSDNKVDFQEFMIMPVGAKSVREAIRMGSETFQALKSLLSADGKVTSVGDEGGFAPDFANNEEPLQYLIKAIEKAGYKAGEDISIAIDVASSELWNNEDKTYKLRWSTGEEFTTPEFVKYLEGLVAKYPIISIEDPIDENNWEDWASITKELGEKVQLVGDDFFVTNTDYLRKGIKMGAANSILVKVNQIGTLTESLEAIEMAKEAGYTAVVSHRSGETEDTTIADLVVATNAGQIKTGSMSRTDRLAKYNQLMRIEEQLGDTASYKGINSFYNIKK

>fig|1664.9.peg.551

MRKPIIAGNWKMNMNPTQTTEFVNAIKANLPKFDQTESVIGAPAVDLPALLEAAKGTDLKVAAENCYFEEAGAFTGETSPKTLNEMGVDYVIIGHSERRDYFHETDEDINKKAHAIFKNNMTPIFCCGESLETREAGKAEEWVSNQVTEGLKGLSADQVSSMVIAYEPIWAIGTGKTASADQAQEICAVVRQTVAKLYDQTVADKVRIQYGGSVKPANVKEIMGKEDIDGGLVGGASMEPASFLDLVHFND

>fig|1664.9.peg.552

MAKQTVADLKDIKGKKVLVRVDFNVPIKGGVIGDDNRIVAALPTIQYIIDNGGKAILLSHLGRIKSDEDKKELTLKPVAARLGELLNKDVAFVASNEGQELEDAINAMTDGQVLVMENTRFQDIDNDFGKRESKNDPKLGEYWASLGDMFVNDAFGTAHRAHASNVGIATAMKANNKPAVAGYLLEKEIKFLGEAVDAPERPFVAILGGAKVSDKIGVIEHLLAKADKVIVGGGMTYTFYAAKGLSIGNSLVEEDKIELAKELIEKAGDKLVLPVDNVVADAFSNDAKTETVEGNIPDGYMALDIGPKAIADFENVLKDAKTVVWNGPMGVFEMDNFAKGTLAIGEFLGNLSGATTIVGGGDSTAAVKKLGVGDKLTHISTGGGASLEYLEGKTLPGIAAISDK

>fig|1664.9.peg.553

MTTKVGINGFGRIGRLALRRIYATSSDVLEVVAINDLTSPDMLAHLLKYDTAHGQFDHEVSATENSIVIDGKNIPVYAESDARNIPWVKNDGVDLVLESTGFYTSEEKAQAHLDAGAKKVLISAPAGAVKTVVYGVNDDTITADDNIISAASCTTNCLAPLAKAVNDAFGIKAGTMTTIHAYTATQKLQDGPDRGGNVRAARAAAANIIPHSTGAAKALGLVVPALKGKLDGHAQRVPVITGSLTELVATLDKEVTADEINAAVKKVTEGNESFGYNADQIVSSDIIGTEFGSVFDPTQTQIVGGGAGQVVKVVAWYDNESGFTAQMIRTLEKFASLI

>fig|1664.9.peg.554

MRDELSAIEAVAPDFVGVVKKRYQVLQHIDWLAPVGRRTLAEQLKLSERVIRTETDFLKAQGLLNSSKSGMVLTVKGQEVLDQLKQVMDQFLGLRQTERELSRYLGIEHCLIVSGNSDEQPKVLTEMGKLVNSTLQLLLPQGQSVVAVMGGTTMAKAAHELSTDLSKKRNLIFVPARGGIGESVDIQANAVCAEMASQTGGKHRALYVPEQVSEKTYAPLLEEPSVQEVLKLIERSRGVLHSIGEAITMAERRDMPIETIKMLKEKHAVGEAFGYFFNAQGEVVYKIPRIGLQIRDLLNIPCVLAVAGGTSKAQAIEAYMHLAPQQTWLITDEGAANSILQGATL

>fig|1664.9.peg.556

MNKRISSCTTILVGKKATLDGSTLIARNEDGYNKPNPQKFRVIQPADQPIDYTSAINGLKIKLPTNPLRYTSTPDADDQYGIWAGAGINSATVAMTACETITNNPHILAIDPLTDDGIGEADFVTLVLPYSQSARQAVLRLGQLLETYGTYETNGIAFSDHDEVWYLETIGGHHWAAIRIPDNAFVVAPNRLNIAEFDFASPNTLFSEDLPSLIAQYHLNPDDDQVNLRHIFGTATIKDAHYNNPRAYAILKAFGGLDEGDYEDQELPFMMVPTKKLTIEAVKWALSSHFENTPFDPYGTGSKDDRQRYRTIGLNRNEETHILQIRNDVPEALAGIHWLASGPNTFNGLVPFYANTNTTPAVYRETQTSFDPNNIYWLNRLTALLGDTDFDRYQGLRDAFEAQALIDCRKIQLKTDAAILANPENSTAQLTAANQTLADAYFRNLNQLLGKMVTTGANHMKLRCSLND

>fig|1664.9.peg.557

MQYFKQLFSNTFAFLKQSQAYFRSVLLMHGFLLLICLPLLSKASYFILVHYQIQFLALSNLPVLIHQHPLVLLALVGIALLILLLAFFEFTFLLLSVYFIQIKRPLALQALLKMTVAQITHLRIGTIFFFLYYYILIIPLGGLGYHSDLLTKFKLPAFILDYIVTNRRLIAVLGLLIYLSLLYIGLRLIFTLPNIILQQMSLKKALHTSWRFTHHQLVTLVGRLLLIVGGLVGFTTVSFFLLIQLQAFIETHTSVGFQSVVILMTFLQVVVLINLVLSTVTIFFVLVDALLSDSQFNQPAINYSSLKLSVPLNGGLLLLVAMVTLMGTGFYNWNYLRAVSIQQPISISHRGVSQKNGVQNSLTALNQTHHLKPNYVEMDVQLTKDHQFVVFHDFQLAPLTGQSGTPQSKTLAQLTEMTVREHKRTAPIASFDDYLALANQLHQKLLIEIKTPTTDNPGLVQKFLAKYQTSIEAHDHMVQSLNWQVVEDVKKAAPQIQTGYILPFNFIGPPISRADFYAVEMTTVNRHFIQAAHQEKKAVFVWTPNDQQAVQRMMYFGADGVITDNLKAVEQAQNQHIKPHYADKLAFYVMGIG

>fig|1664.9.peg.560

MKIIVVGSSHGGFETVQESLLDYPDATIQWYEQGDFLSFLSCGMQLYLEGTVKNVDDVSYATPEGMRAKGVDVRLQQAITAINADQHTVQVKDLKNNTTREESYDKLVLSVGAVPFELPVAGRDLANVYAMRGRDWAIKLKAKTVDPTVKNVVVIGSGYIGIEAAEVFAKAGKNVTVVDVLPRLLSLYLDQEFTDELTTEMQAHNIYPAVGQSVKEYRGADGKVTKVVTDQAEYDADLVIEAAGIRPNTAWLKDVLDLDRSGRIKINEYLQTSQPDIFAVGDATVVKYAPTGEEIPIALATNARRQGRYAAKNLVTADQPVPAVSGSSALSVFDYHFASTGIKEGTADKSGVKTQSVVVTETYRPHFVPADENPEVKFKLTFDPETGRILGAQIMSKADVTANINAISLAIQGKTTVDDLAYADFFFQPGFDRPWNIMNVAAQKAQRLMH

>fig|1664.9.peg.563

MATNNLVLKTADICDIRDIEEARATIKPYIRETPLIQSMFLSRNVAKGNVYLKLENMQLTGSFKFRGANNKINHLTEEQRQRGIVTASAGNHAQGVALTAKLLGIDATVVMPEEAPIAKQEATAGYGANVVLHGATFNDARLYMEQLAEEKGMTIVHPYDDREVMAGQGTIGLEILDEIWNVDTVIIPVGGGGLISGVATALKSFNPSIHIIGVQSENVHGMAASIDAGKITSHHDDFTLADGTDVAIPGDLTYPVVQNLVDEFILVTEDEIAMAMTDLMQRTKIVTEGAGALPTAALLSGKIDPKWLEDKNIVAMVSGGNVDLTRVSGIIEHLFKPADTSKGVVG

>fig|1664.9.peg.568

MPTNKKEGIIFTTLMCFSMVLGMSIYNLLLHGSFSFVALITGLIPGFIVAFILDVFVIGVLAKKIAFSLPLNKDNKLVMILTISCLMILGMVTCMSLFGVLIEGGIPNDLGGAYRHAWAMNIIVALPLQLIVVGPFSRAILGKIQANAATAETAE

>fig|1664.9.peg.569

MLLFGIVSAFLMLGGDTLQHSISQLLDTMTSHSQWLYIGIYIINFIFLVWLGCSHYGRIRLGQPNEKPEYNDFQWGSMVFATGIDASILMLSTTDPLQYLQNPPFGAKPFSAAAYRYANVCGQFNWGPMAWMIFATATIAIGYALYVKNLRVQRLSAAIDLLEGPQTYKKVLRQLIDFIVVFGIMGGIGSSIGMEIPVISKVLSTMTGFSDTIWLKLALFVVLFILFTLTVYAGLNKGIKKLSAWHIYTAIGFLLLVLLIGPTRHLIGAEGHNLFHLFGKFGQLSLNATGSDSHFAQHEMLFYWGWWLSYMPVMGLFIARISRGRTIRQVLLGMATYGALSCMSFYAILGGYALFLQSSGQVDLVTLLNTQGQAAVIAAVITTLPWKIVMLAIFCLSCFIFLATTISSSAFIVSSFTSLELKIGEQPNRWNRMAWVVIFILFSLGIVLVGSFQTVQAVCVIAGFPLIFVCALLLVSIFHAVHNGEIPIEIENEETSHFLKKKLTPKFFM

>fig|1664.9.peg.570

MAQKNILQYLLLGLLNQAPKTGYDLKRVFENEIGEFWQAKHSQIYPELKRLETEDLITHIELISGTKLMKKQYTITPSGVSYFNEWRYSPTSEILASKDEFILKLYFIESKNDQHLPAMFAEQTTLHTYKLTHLLARKALVFPNQATITANYGHYLILEHAINREQGYLKWLATADC

>fig|1664.9.peg.571

MAKKSKIAKLAKQRALVAKYADLRATLKAEGDYAALAELPKDSSPVRLRNRDLIDGRPRSFMRKFGMSRINFRELAHKGQIPGVKKASW

>fig|1664.9.peg.572

MKNVSKKSPSILLMIVLVGFPQISESIFTPVLPMISQAFHVSAQTAQLTMSTYFMAFAFGVLFWGWLSDQIGRRLTMLLGIGVYLLGNGGLLLAGHFSWLILARLVQAFGASVGSVVTQTIMRESFSGVRGAQVFAKVGAAMALAPALGPLIGGLVQTYFGYRQVFSVLIMMAVGAILYAGACLPETRPAGVPAQVNIGAVTKRLLTDKKVWVYGAVISGILFSYYAEAPFIFINHFHLGTVQYGWLGLVLAAASILGAMTTNYGAPKFGAVMMAKIGLVLALIGSLLLLVASYHDQLLLMVILILIVFWGLNTTLPVVLNLVLVGYEAVIGTASGLFSFGYYLAISALTYGMSLLHTGAISRLPLYIVTIVAVMALWYGLVIQNNEV

>fig|1664.9.peg.579

MSAWESKFTKKGFTFDDVLLVPVESHVLPNEVDLGVQLAKNIKLNTPIMSASMDTVTEAPMAIAMARQGGLGVIHKNMSIERQADEVLKVKRSENGVIIDPFYLTADKPVSAAEDLMRTYRISGVPIVSNLDELKLVGIITNRDLRFISDFSVEIGTVMTHEALVTAPVGTSLEEAEQILQQNRIEKLPLVGDDGRLAGLITIKDIEKVQEFPKAAKDQYGRLLVAAAVGVTSDTFDRAEALLKAGADAIIIDTAHGHSAGVLRKIAEIRARFPEATLIAGNVATAEGTKALYDAGVDVVKVGIGPGSICTTRIVAGVGVPQLTAIYDAASVAREYGKTIIADGGIKYSGDIVKALAAGGNAVMLGSMLAGTDEAPGEFEIYQGRRFKTYRGMGSLAAMSHGSSDRYFQSGVNEANKLVPEGIEGRVAAKGALGDVIYQLLGGLRSGMGYVGAANLRDLQDNAQFVQISNAGLTESHPHDVQITKEAPNYSAR

>fig|1664.9.peg.580

MKILIVDDDKEIVELLSIYVKNEGYEPIQAFTGKEALTKIATNPGIDLMILDIMMPNMSGIEVIKAVRKDSQVPILVVSAKTTDMDKIQGLITGADDYVSKPFNPLEVMARVKSLLRRSQQQVTNEVPDILEVGPLVIKRDSHEVTTIAGKQIQLTALEFGILYLLASHPNRVFSADDIFERVWKQESIVSAKTVMVHVSHLRDKIEEATDGEKVIETVWGVGYKVEV

>fig|1664.9.peg.581

MNEYQVGQDFKKVQLTAKEKSELFAEGIITVILLLLLNLSIMVLLQQAIVNNPQLRGGVWMIKNAIAIGPNGWHIWSWQNWFVILMGIADVLVVYWRLIRRYHQMQMWHVIGELHYIADGHLEHRINLRVNRDLQRVIDSVNTLVDSTVRSMEEERKIEKSKDELITNVSHDLRTPLTSIIGYLGLIEDNQYQTKDELTKYTHTAYVKAQQMKVLVEDLFEYTKVRQTSTPIAKTTFDMEQMLEQLAASFELEAQKKNMQITVECQPAPLMMEADTEKLGRVFNNLIANALKYGKGGKQIILSAEKVGQEAIIKVSNDGQQIPSDSLNQLFDRFYRVEASRSQETGGTGLGLAITQSIVALHGGYIYADSTPELTSFVIHLPLKLGRQIEPQNGAAKNS

>fig|1664.9.peg.582

MFKRILQVLMTGLLLVTPVAVATSATQQGVQAATATSAPQVDASAAIAIDPQSGQVLYEKNADQVLPIASMTKMITAYIVLDQIKQGKLKWEQTTKPDAAIYKLSQDKELTNVPLQADKAYSVRELYQAMLIASANDAAMMLANLVAGSQKNFVDLMRQKVSQWGIKDAKLYSVSGLNNQYLTGETYPGSAADAENEMSASEVAIVAQHLLKTFPEVLETTKQTAFTFGPKTIDETKMATWNLMLPGQNNAPKDYVVDGLKTGTTDKAGDCFTGTATKDGQRILTVVMHANGSDKGRRFIETNKVMQYVFDNWQQTQLVKKGATIKPFATADVKYGRTKTVALETPQAIAMWVPKGTQVANVDWDYQVVQGGQKRQLTAPVKKAAVIGKVTPKIAGVTNRYLGTTPQYTVQTKTADPKANVFVLIGTGVREFFTNLF

>fig|1664.9.peg.583

MALTLDACTLILKEHYLLKSVALNGDSNLNMTGIAYDSRKVTADTLFFCKGNFRPVFLTNAKEAGAVTYVAEQPIVEGNGMNALIVTNVQKAMAVLSAAFYDYPQDDLFIVAYTGTKGKTTASYFTQSILQEATNQKTALFSTIDRVLGPKPEDRFKSDLTTPESLDLFHDMRQAVENGMTHLVMEVSSQAYKKNRVYGLTFDVGFFLNISPDHVGPNEHPNFEDYLHCKLQLLVNSRHCVINAQTQNFGDVYAAATTTTEPENIYLFADTSYQPAQPVDIDFRFENQAMTLAESRFKVTAVSKKAIQLGVSGDYQLRLIGDFNESNATAAIIGAALGGADLIAAQQGIRRLQIPGRMETLAVEGHGQVYVDYAHNYASMKALLSFLQKEYNQPKLLVVVGSPGDKGVSRRAGFAQVLNEYADRAILTTDDPGYEDPAAIAAEILAGIDQDKVATTVEIDRATAIKQAIEESQPGDIVVLAAKGADAYQKVRGVDTPYPTDMVIAKQVAQYLS

>fig|1664.9.peg.585

MNKKRLGIVALLLMVAMVIAGCGQTKQTTKGIQVVSSLDFYGEAAKAVLGKHGQVTSIINSPSLDPHSYEPTTQDAKKVAQADVVIENGLGYDQWLSKVAASSDSDQQTMITVNKLMKRTDGDNEHLWYDLETMPTLTKQLITRFSKIDPAHKADFQNNGQAYLKKLAGLKTQLAGLKAQAKGQKVAVSEPVFDYALKEMGYQVANQHFAKAIEDGTDPSPQDIDAMRQLIKKHQIAFLVVNKQEESRTIKEMHQLATKEKVPVVQVTETLPAKQTYLTWMQQQFDAVGKAQNVK

>fig|1664.9.peg.586

MTELVSVKNLGMTFSNRQVLKDLNFKIERGSMTSLIGPNGVGKTTLVRILIGSLQPTAGELTFSPDKQSLNIGYVPQFRNLEAEYPLSIRSFVSLNQLTKHLPWHTKSERQALDKILAQTHLTALQKTTVGNASGGEKQKAYLAQALISEPDLLILDESTASLDIETKEEVMALVAELNRLHGLTVIFVTHDLDLARKYTDQYLFLKRGQYEMGPMSQLDPNLELD

>fig|1664.9.peg.587

MLTFDFMRHAFVASTFIAITCGLIGVFVVARNMSFLAHTLAEIGFAGASFGVFLGIAPLNGMLLFTMISSITIGQMSAKESRREASISAISSLFVGLGILFLSLSNRNASYATNILFGSIIGISLTDVYQLITLSAVIVVGILLFYRYLVFDSYDSIGAQAQGLKTNWLSIYFLILLAISVSIAAQIVGSLLVFILVTLPAFIAKYLAKSVPAMLAVSVGAALLGVWLGLWLGYITNWPVTFFIATLEFIFYFIALLYHQHVSH

>fig|1664.9.peg.588

MTIHKLPTRSEVPENLTWDLSTIFKSDSDFEVAVATLKQALPALEQYNQPFKTADQLVTTIEQVLTLFRQLETVYVYASMKNDQDTANTTYQGYQAQVDALAADVSAAAAFLEPAILEIPVDQLTDWQTNEPALKSYQHFIQTITDSREHVLSGQEEALIAAAGDIFGAASQTFSVLDNSDIQFPDVVDETGQTHQLSNGLYSQLLQSTDRQVRQDVFEALYSTYDQFKNTFATTLATEIKGHNYLAQVHHYDSARQAALAPKAIPESVYTTLVDQVNQHLPLLHRYVKLRQKQLALPQLHMYDLYTPLLGKPALSYTYPQAQTTAREALAVLGPEYTPIVDQIFDQRQIDVVENKGKRSGAYSGGAYDTNPFILLNWQDDLNNLYTLVHETGHSGHSYLTRHNQPYVYGDYPIFVAEIASTTNENLLTEYLLKTQKDPKVQAYLLNYYLDGFKGTVFRQTQFAEFEHYIHDAAANGTPLTADFMSTYYADLNAHYYGDAVAKDPQIALEWARIPHFYMNYYVYQYATGFAAATTLADQISTNQPGALDNYLAYLKNGSASYPIETMQKAGVDMTNADYLEHAFDVFEQRLDQLEALLADM

>fig|1664.9.peg.589

MQNSNFFGSREDKYKKGKRHRKLKIFLLVIVVLVFSGGAYAMRVSSQLKSAADKINKSEATSKAIKNKKAFSILLLGVDTGAEGRIDKGNSDTMIVATVNPKKEQVKLVSIPRDTLAELQGTKTFDMQKINAAYNVGGSKMAKKTVEKMFDVPIDYYVTINMGALSKIVDAVGGVDVNVPFSFKYNTTFTKGKMHLTGKEALDYSRMRYDDPEGDYGRQKRQRQVITSLIKSAVSFKSLTNYESILKTLEDSVATDLSFDDMVAIQSNYRTAAKSISSDYLKGRNATIEGSSYQVPTTSEIQRVSDVLRTSLGLEKTNIDNAETKQNALNTNFATTEYQDEFTIYPESVLKDGTTTSEAASTTTTQDNTTQYSQSAPQSNWQQPQQEQSQWQSSY

>fig|1664.9.peg.590

MLVNVIGLLVFIGVGFLFSKKKKDIDWRSVLIMLVINLVLAWFLTSFSVGRDIVLGAANGFNWLVQVAYTGIAFALPSWVNVKQMDFVASVLLPILMIIPLFDILTYIGVLPWIIKWLGRGLAKITGQPKFESFFAVEMMFLGNTEALAVSSLQLKQMKAERTLTLAMMSMSCVTASIIGAYTQMMPGQFILTAIPVNIINAIIVTNILNPVKVTPEEDTIAKMGGSGSAIGEEEAADGKIEREPFFSFLGDSILNAGKLVLIITANVIAFVALAALIDKVLQLFNPWITLEHLLGIVMFPFAWLMGLDVSHAFEFAQYMGTKLVTNEFVVMGKVQNSITTFAPHYQAVLTVFVTSFANFSTVGMIIGAFKGLVDREKNDMIAKNVGYMLLSGILVSLLSAATVGLFVW

>fig|1664.9.peg.591

MADLVYQTIIADLKKAINAGAYPSMKLPDERTLTEHYQVSRSSVKRALNVMANQGIIFKKRGSGTFINPLYLKSGSFFNYSGSNLGVTDSFKAGDRKPGIKLLDFQVIKPSAELQRDLFLEPDDFVYEIKRLRLFDDEPFMIETGFIPIKIMPGLTEKIISGSIFNYLETEKNQEVTRAFLSIFAEPSTQDDQDKLQLKPTEPVGIMEGIFFLDDGTPFEFSTMRLHYQYMKYNTFVSVSGRE

>fig|1664.9.peg.592

MTDYSSKEYLAKVDAFWRAANYISVGQLYLKDNPLLQRPLEAKDVKVKPIGHWGTISGQNFLYAHLNRAINKYDLNMFYVEGPGHGGQVMVSNSYLDGSYTEIYPEISQDVEGMKKLFKQFSFPGGVASHAAPETPGSIHEGGELGYSLSHGVGAILDNPDVIAAVVVGDGEAETGPLAASWLSSTFINPKNDGAVLPILNLNGFKISNPTILSRKSDEELTKYFEGNGWAPIFVEGDDPEKMHPATAAAMDEAIEKIQAIQKAARENGDTSRPVWPMIVFRAPKGWTGPKTWDGVPIENSFRAHQIPVPIDSTDMQHADALVDWMKSYRPEELFTAEGQLKPEIAAMAPKGDKRMAANPITNGGIDPKPLRLPDYRDYAVDNTEHGKVVAQDMIVLGEYVRDIIKDNDQNKNFRIFGPDETMSNRLNHIFDVTNRQWMEPIKEPNDQFMATEGRVLDSQLSEHQAEGWLEGYVLTGRHGFFASYESFLRVVDSMLTQHFKWLRKADEQAWRNKYPSLNVIATSTVFQQDHNGYTHQDPGVLTHLAEKKPEFIREYLPADANTLLATMNTVFQSQEKINLVIASKHPRQQWFSIDEATVLVKNGLKIIDWASTDQDAEPDVVIAAAGTEPTLESLAAISILHKQYPDMKIRFINVVDLLKLRSPKVDPRGLTDEEFDMYFTKDKPVVFAFHGFEGLVRDIFFDRHNHNLHVHGYRENGDITTPFDMRVLNQMDRYNLSKEVAIDVLGDQAGQFAQSMDDMVAKHNQYIRDEGTDLPEVEEWQWTPLR

>fig|1664.9.peg.593

MAKNKIRVRYAPSPTGHLHIGNARTALFNYLFARHNKGTFVIRIEDTDTKRNIADGENSQLDNLKWLGMDWDEGPDKPGNYGPYRQSERREIYTPLIQELVEKGLAYESYKTEDELTAEREAQKAAGEAPRYVYEYEGMSEAEIKASQEAAKAKGLQPVVRLRLPKDHVYKFDDIVKGEISFESDRLGGDFVIMKRDGMPTYNFAVVVDDHLMEITHVLRGDDHIANTPKQLAVYEAFGWEPPIFGHMTLIINTETGKKLSKRDESVLQFIEQYRELGYLPEAMFNFIALLGWSPVGESELFNRQEFIKQFDPKRLSKSPASFDQKKLEWVNNQYVKQSDPNKIMDLSLDSLIKAGKIAENPDSKTIEWARHLIALYQDQMSYTAQIVEMSDVFFEEPEQLDAEALEELNNETAPVVLKEFSERLKDLTLFTAPRIMQIIKGIQKDTKIKGRLLYMPIRIATTREMHGPSLPDSIELLGKDRVLAHLEKTLAEIK

>fig|1664.9.peg.594

MKVLTFGEMLMRLKTPGNQRLLQADSFEASYGGAEANVAVSLALLGDQVAYLTKLPENLLGQKGMQTLRGYGVDTTKIQYGDGRLGLYFFEKGASVRSTNVVYDRAHSSFATMTASEFDWATIFEDIDYFYFSGITPAISAELAQAAEAACQYCQAHDIPVVCDLNYRGKLWSPEQAQATIKRLMPYVTICIAHDEDFEATLGIQAFDGDESHGIAQKESFKAAMQQVRQIFPNCQMVASILRNIRSVEDSEWLALLLKDDQFFETGTYQMHVMEGVAAGDAFGAGLMHGLLNNFTPDKMLQFGLAASVAKLTIAGDFNLMSEAEIEAIAQSGGGARLSR

>fig|1664.9.peg.595

MTKKVAFITGAAQGIGKAIAQRLSNDGFAVAIADFNLEGAQAVAAEINETGRAIAVKVDVSDRDQVFAAVETTVAELGGLDVVVNNAGVGPTTPIETITPEQFEKVYAINVGSVYWGIQAAVKAFRDLGHGGKIINASSQAGQVGNPELALYGGTKFAIRGITQTAARDLADQGITVNAYCPGIVKTPMMMDIAHKVGQNADKDDEWGMQQFAKDITLGRLSEPEDVAACVAYLAGPDSNYMTGQALIIDGGMVFN

>fig|1664.9.peg.599

MAQAIDSLLRSYYDNFSEKYKQIADYIFQEAQFNQRLTIQEFAANCGVSTATISRFAKILGFDNFQAFKMALLAKEANTSPLFHEINANDTFTTMAEKIFTSNSNALKATWSLLTEEQLQKAVALINRAHLLSLFGLGASGIVAQDGYHKFLRTSIPTVFNQDYHLQLMQATKLTNQDCAIIISHSGQNKDALELARILKERQVPLIVITSFGNSSLAKLGNITLLSISEETNYRAEALHALIAQISLIDSLFMMVAVNNGQETEQSFKEIRAEIDRTRQ

>fig|1664.9.peg.600

MKLAMVGLGKMGINLVANLRRNGHEVVAFDLNQAAVEEAVNLGATAATDLDDVLAQLTSPRIVWVMLPAGKPTDATIQTLSEKMNAGDMVIDGGNSFFEDSERHNALLKEQGIHFFDAGTSGGMSGANANGNFMIGGDSPEAFKTIEPIFKSIAQEDGYLYTGKAGSGHYLKMVHNGIEYGMMQSIAEGFDVLEHGRFEYDNEAVAKVWSNGSVIRGWLMELAQSAFSKDPELESLKGIMHSSGEGQWTLDEALKLQVPTPVIGLSLMMRYRSLEDDTFTGKVVAALRNEFGGHAVDKK

>fig|1664.9.peg.601

MMKYMIGADIGTTSTKAVLYDLKGNVKAYANVGYPLYQNVPDMAEEDPDEIFEAVVEVLGKVMRKGHVQAGELQGVSFSAAMHSLILMDRNHQPLTRAITWADNRAAKYSNELKANGLGHEIYQHTGTPIHPMAPLSKIIWLRHEQPDLFKQAAQFIDIKTYVFQKLFHTYKMEYSIASATGLFNIFNLDWDQQALEVAGITRDQLPELVEPTAQITGLDTTYSQETGISSDVPFIFGASDGVLSNLGVNAIDPGVLAVTIGTSGAVRVVVDKPVVDPNGRLFCYALTKDKWVVGGPVNNGGIVFRWVRDQLFAPEKLTAEQMQVSTYDLLTQIAEKIPAGSDGLLFHPYLGGERAPIWDSDARGSFFGLTRQHTRAHMVRAALEGIVYNLYMVMLMIEGVAGKPKSIQATGGFARSELWRQMLADIFEQDVTIPESFESSCLGAAVLGMYSLGLVDDLSAVKDMIGVTNVHEPKAENFDVYRELIPIWIRLTRELSPEYSAIADFQRRHMQVKEATKD

>fig|1664.9.peg.602

MELLVLLLGIMLLLLLIIKFKLNTFVSLVLTAMVVGVGLGMPLTKIATSVQNGIGGQLGELSIVFGFGAMMGRLVADAGGAFRIAHTLIDKFGKKRLQIAIMLASFIIGIALFFEVGMVLLVPIVFAIAVEASVPILYLGIPMAAALSVTHGFLPPHPAPTAITSALGANAGHVLLYGVIIAIPSVIIAGPLFTKVARKMIPDAFARQGNLTALGPQKTFKLEETPSFGISVVTSLFPVILMAITTVYQMLFNGGSLPKNPTLLDSIIGFIGTPAIAMTISLLLAMYTMGWGRRIKTPAIMKTIEEAVKSIAMLLLVIGGGGAFKQILIDGGVGDSVTTLFAHASLSPLVLGWLIAVVLRIALGSATVASLTAAGLVLPLMQSAGVNPALMVLSIGAGSLAASHVNDAGFWMFKEYFDLSVKETLATWTVLETLISIVGLVGVLLLSLVV

>fig|1664.9.peg.604

MKNIALNIRDFFLDDKLLLAATIAAIVTSFFNRPHLSYVNFHVIVSVFCIMTLVQVYQRLHVLDYFARELIIKSHSKRALMQMLLALTFVGAMFLTNDMTVLTFVPLFILIARQLDFSPILPVTLITISANLGSSLTPFGSPHNIFLVSFYHMTIRHFFEYSIPVLIVSIIMLTATTFLFPKEPIKLHGLHPVEVEKGPLWYFVPLTVVVFLAVFSLIPLWVTPILVIIAILVFDPKLFKTVDYGLLLTFFCFFIAVGNLSHIPTIATFMRSLVGTAKQTYLTGLISCQLIANVPTEILLSSFTNHSHAMFLAINIGGVGTMFASLANLIAYKRFKKGWGKDIGKFLVVFTGMNFLFLIILGTFGYFLI

>fig|1664.9.peg.606

MSILKVEGLSQQFLDKQLYDDAGFQVNKGEHMGITGQNGVGKSTLINILTGAILPDEGKVTWQKNIKIGYLDQYAKLAPGLSIFEFLKTAFADLYAQEERMSQYYADYATSLDDQLLEKAGAIQELLEAKNFYEIDTRVMQVATGLGIDALGMDHDVSQLSGGQRSKIILAKLLLEEPDMLLLDEPTNYLDTNHIDWLSDYLNNFEGAFITISHDYDFLERVTTCIADIEFGKITKYTGTLQQAFRQKADNQASYMKAYENQQKKIEKTKAYIRKYKAGSRSTIAKSREKQLAHMDILTPPSNRVKANFRFPYHATASQILLTTNDLEIGYENALLPALNFSIGRDQKVVLKGFNGIGKSTLIKTIIKEIPAISGDFEIASTVKFGYFKQDLKWPNNMATPLQYLQGEYPEQKQKELRQVLSRTGLTAEEAMKPLKQLSGGEQSKVKIAELLLTSSNFLLLDEPTNHLDDDTKNALRKAIQEFEGGVLLVTHEEDFYQGDWIDKVLDVESLIKS

>fig|1664.9.peg.608

MITVSEMSLTLSGRKLYEDVNLKFTPGNCYGVIGANGAGKSTFLKLLEGKLQPSSGHISMGPNERMSSLNQDHYAFEEFEVLETVIQGHKKLYEVMQAKDALYAKAEFTEEDGMKAADLEAEFAEMNGWDAESEAAQLLQALGIDESLHHVKMSELMEGQKIKVLLGQALFGKPDILLLDEPTNGLDSASVEWLENFLADFPNIVIVVSHDRYFLNQVCTMMCDVDFGRIKTFVGNYDFWLESSKLAAKLQSNVNAKKEEQIKELQDFIARFSANASKSKQATSRKKQLEKITLEDIQPSSRQYPFIKFDFERELGNDLLRVENVSKTIDGVKVLDNISFSVKPDEKAAIVSRNDVATAVLMQILAGEVEPDSGTVTWGVTSQQSYLPRDTNSAFNDDQLIILDWLRQFAAKGEDDNTFLRGFLGKMLFSGDEVTKEINVLSGGEKVRCILSKIMLQKSNTLLLDDPTNHLDLESITSLNDALIDFKGALIFTSHDHQFIQTIADHIIEVSANGLVDRAETNYDEFLAHPELQKQVAKLYA

>fig|1664.9.peg.609

MREYDVAHYDVIVVGAGHAGSEAALAAARMGRETLLLTINLDMVAFMPCNPSLGGPAKGIVVREIDALGGQMGRNIDKTYIQMRMLNTGKGPAVRALRAQADKHAYHRTMKRTIETTEHLTLRQGIAESLIVEDGVCKGIVTNTGARYSADSVILTAGTASRGKIIIGELTYSSGPNNSIPSIKLSESLEDNGFVLTRFKTGTPPRVDGTTIDFSKTEEQPGDKEPNHFSFETPDSAYLKDQLSCWMTYTNDTTHQVIRDNLDRAPMFTGVIEGVGPRYCPSIEDKIVRFADKPRHQIFLEPEGRETEEYYVGDFSTSMPEEIQHKMIHSIEGLENAQMMRPGYAIEYDVVEPWQLKPTLETKVVENLYTAGQMNGTSGYEEAAGQGLMAGINAALKQTGRDPFILDRSEAYIGVLIDDLVTKGTKEPYRLLTSRAEYRLMLRHDNADLRLTEKGHELGLINDDRFDSYEVKKAAVEAELARLEKIRLKPTPEIQAFLAAKGEAPLKDGVLASDFLKRPEVKYADLIQFIPAVEGIDNRVVEQVEIQVKYAGYIDKEKAKIAKLKRMEAKKIPANIDYDAIEGLATEGRQKLQKIQPETLAQASRIGGVNPADIGILSVYIQQGKIAKVK

>fig|1664.9.peg.610

MPTTVTEFDTITAISTPPGEGAISIVRLSGDDSLAIIKRVYRGKDLDKVASHTINYGHIIDPKTDAVVDEVMVSVMRAPKTFTREDVIEINCHGGIVATNRILQLLMSYGARMAEPGEFTKRAFLNGRIDLTQAESVMDLIRAKTDRAMQVAVDQLDGSLTHLIKNLRQEILEVLAQVEVNIDYPEYDTDEMTTRILLEKAELVKGRIGELLQTAQQGKVLREGLATAIVGRPNVGKSSLLNHLLHEDKAIVTDVAGTTRDVLEEYVNVRGVPLKLVDTAGIHDTEDKVEKIGVERSRAAITKADLILLVLNQSEPLTIEDRELITATTDKKRIIILNKTDLPNQLDLDELQTLVRADEVIQTSILTSEGVTDLEAQIAKLFFGGIENSQSTVMITNARQIGLLNQAQQSLDEVISGIAAGMPVDLVQIDMTNCWDKLGEITGDSAPDELITELFSQFCLGK

>fig|1664.9.peg.611

MPTFEGKDIQAAIDAGLAALKLTRDKVTVDVLEEGKKGFLGFGKQPAIVTLNPITVAEPTPVEPEQPSVEDVATEPADQPEKAPMRQSREATIEALKQYITDITAQIGTPATMTVTKEHKQVIFHLKTTKEGLLIGKHGKTINALQYLAQTYYDHHTKGKQLMMLDVGDYRQRRATIVKHLADKAAREVVATGKAVTLEPMPAFERKIVHGYLTNNRHVQTHSEGRGDRRVIVVESARSF

>fig|1664.9.peg.612

MTYSEQWDLETIFKGGLASPALENRLQALGQQIPVVLKMTSDWQFVFDEPAFEQMQAITMQLQQIESGLKQMGSFVNMIQSVDERNQQVAPMMDRINQLQNTARQIDVLLTKKLVALTVEQFETLVSQPAFAPIQFNLREKRQQGADLLDEQTEKLLDQLALDGLQGWSDHYDTIVAETQIPYLDQTLSAGQAQNKFESDPDSKVREVVFKNWQQTWDRFSPMLADTLNHLAGFRLTTYQAHGTTDFMKKPLEYNRMQPATLDAMWSVVSQNKAPLVAFLNRKAALMGKKQLAWQDTWAPVVVDDFQMTKKSYDEAAEFIIAHFRDFSPKMAAVAQTAFEKRWIEAEDRPGKQPGGYMTDLPETNDFRIFMTYDGTPGGVSTLAHELGHGFHTMMINQFPGWRQDYAMNVAETASTLAELIVVDATVNEAQDRATKINLLDQKITNAIDMFMNIHARYLFEHRFYEMRQQGLLTPDDLKGIMLSAQKEAYADALTDYDPLFWADKQHFFFDDVPFYNFPYTFGYLFSMGIYAKAQSAPHFEADYIALLQDTANMSTEELAQKHLGVDLTQPAFWQAAADSIAQDVALFLELTATDLD

>fig|1664.9.peg.614

MNSKNRKRLLLVASLFLFMLIAAGCSTAPVSSASTGFWDHYIIYNFSRFLLWLADIFGGSYGWAIVAFTIIIRLIILPLTWWQSKTMMKQQEVAPEIQALQKKYASKDVETQQKLREETQKLYAEAGVNPVIGCLPLLVQMPVLIALYQAIYRTSALKSGSFLWMQLGKADPYFIMAILAAVFTFATSYLTMMSQPTKNATTTAMLWVMPIMIFVMAMNIASAVSIYWVVTNAFSVGQTMVIQNPFKIRREREAKEQVKRDREKALAKARKKATKKR

>fig|1664.9.peg.615

MRKSYRVKKEQDFQTVFDASQSVANRNFVVYRLDKPGQKHFRVGLSVGKKVGNAVMRNQVKRYIRQSITELKPDLLQPVDFLVIARRGANQLTMAETKQNLIHVFKLAKLLKEEADSASE

>fig|1664.9.peg.616

MTTKRTFQPKKRHKERVHGFMKRMNTKNGRKVLARRRAKGRKVLSA

>fig|1664.9.peg.617

MPDLQELWNYLQEKFQTDLTTVGFNAWIKTAKPLAFRANELLIEVPSVLHKEYWENNLATKVVEGAYEFAEIELTPIFVLPGESDNLTPLEPEEEHVLTKAETPTFLRETHLNSKYTFDTFVTGKGNQMAHAAALVVSEEPGVLYNPLFLYGGVGLGKTHLMQAIGHQLLESKPETNVKYVTSEAFANDFINSIQTKNQEKFRQEYRNVDLLLVDDIQFFADKEGTQEEFFHTFNDLYNDKKQIVLTSDRLPNEIPKLQERLVSRFKWGLPVDITPPDLETRIAILRNKADAEHLEIPEDTLSYIAGQIDSNVRELEGALVRVQAYATMQNAEITTSLAADALKGLKLNGKSSQLSIAKIQSVVAKYYSLTVADLKGRKRVKEIVLPRQIAMYLAREMTESSLPKIGQEFGGKDHTTVMHAHERISQSLTTDQNLKDAILDLKNTMKS

>fig|1664.9.peg.619

MTQEIKLEAEFVTLGQLLKEAGIIETGGKAKWFLRENTVLVNGEPDDRRGRKLYPEDVIEVPDNGQFIVKQQERL

>fig|1664.9.peg.620

MYLSELQLNHYRNYESVDVHFSPDTNVLIGENAQGKTNLLEAIYVLALARSHRTNTDRELIQWHEDFAKITGLVQRSAGKTPLELVLSQKGKKAKVNHLEQAKLSQYIGRLNVVLFAPEDLNIVKGSPAVRRHFIDMEFGQMSSKYLYNISQYKSILKQRNQYLKQLQRRQAKDLVYLGVLSDQLAAYGAEVTVARRQFLQQMEKWAQKLHQEITKDREVLTFKYQSQIPEEQLDQSVEELYQQFQTLYEKQQTREVEQGTTLIGPHRDDVQFLVNDKDVQAFGSQGQQRTTALSVKLAEIDLMKAQTGEYPILLLDDVLSELDDLRQTHLLKTFQNKVQTFLTTTSLENVKKEIIATPRVFTVTNGVVTEEQVE

>fig|1664.9.peg.621

MADDQLNKETEAQIEAKEERAKSYDASQIQVLEGLEAVRKRPGMYIGTTSSQGLHHLVWEIIDNGIDEALAGFATEINVVVEPDNSITVTDDGRGIPVGIQAKTGKSALETVFTILHAGGKFGGGGYKVSGGLHGVGASVVNALSTELDAVVTQGGKRYYIDFKRGKVQTPMKMIGDAPEHEHGTKVHFVPDPDIFTETTIYDDKILTTRIRELAFLNKGLKLTFTDKRADTHEKLEFHYEGGIKSYVEFLDEKKETLLDEPIYVEGEQNGITVEVALQYTNDYRTNLLTFANNIHTYEGGTHESGFKTALTRVINDYATKQNIFKASDANLSGEDVREGLTAVVSVKHPDPQFEGQTKTKLGNADARSVTDRMFSDHFTKFLMENPSIARQIVDKGSLAARARLAAKRAREVTRKKNGLEISNLPGKLADNSSKDPEISELFIVEGDSAGGSAKSGRSRLTQAILPIRGKILNVEKASMEKILANEEIRTLFTAMGTGFGQEFDVEKARYHKLIIMTDADVDGAHIRTLLLTLFYRYMRPIVEAGYVYIAQPPLYRVKQGKMMRYIDSDEELQDVLGTLQPSPKPEVQRYKGLGEMDASQLWETTMDPENRRMLRVNVEDAEEADQIFEMLMGDRVEPRRLFIEENAVYMDPKSIDA

>fig|1664.9.peg.622

MANENGQDGRIENVNLSKTMRKSFLEYAMSVIVARALPDVRDGLKPVQRRILYGMSELGVTPEKPYKKSARIVGDVMGKYHPHGDSAIYEAMVRMAQEFSYRYMLVDGHGNFGSVDGDGAAAMRYTEARMSKMAVEMLRDINKNTIDYVPNYDESEREPAVLPARFPNLLVNGATGIAVGMTTNIPPHNLAEVISAIHILMDNPDATTADLMDALPGPDFPTGGLVMGKSGIRHAYESGKGTITLRAKVDVEVKKNGRERIIVTEIPYMVNKAKLVERIAELARDKRLEGITDLNDESDRDGMRIVIDVRRDMSASVVLNNLYKLTPLQTSFSFNMVAIVNGAPKVLSLKSILEHYLVHQEEVIRRRTEFDLKKAEARAHILAGLRIALDHIDAIIQIIRSSKTGDTAKVLLMERYDLSDKQAQAILDMRLVRLTGLERDKVENEYNNLMALITDLKDILAKPERIKKIIYDELLEIQSKFGDKRRTELLVGEVLSIEDEDLIEEEDVVIALTHNGYIKRLPASEFKVQNRGGRGIQGMGVHDDDFIEHLISTSTHEVLLFFTNTGKVYRAKGYEIPEYGRTAKGIPIINLLGINSDERVQTVVNIGKEVKPEQALFFITRLGTVKRTAVGEFANIRSNGLKALTLHEDDELSNVLMTDGQQNIFIGTHNGYAVSFSETAVRSMGRTAAGVRGIRLREGDYVVGSDLLIPGQEILVISEKGYGKRTAVSDYPIKGRGGKGIKTANITEKNGPLAGIAAVTGEEDILLITDTGVLIRFKVANVSQTGRATLGVRLIRVDDEAKVATMAKVEAEPDEPETQTATTEPTVPTENVTDTGLAQVDDLLARAEENQNEPNND

>fig|1664.9.peg.623

MAQSKYEVTYIISTTLDEESKTALVNRFDGILKDNGAEVVDSKDWSKRRLAYEINNQHEGIYHIVNVLADNDEALNEFDRLAKIDGAILRHMIVKRED

>fig|1664.9.peg.624

MINRVVLVGRLTRDVDLRYTSSGAAVGTFSMAVNRQFTNANGDREADFINCVIWRKSAENFANFTKKGSLVGVDGRLQTRNYENQQGQRVYVTEVVVDNFSLLESRTTTEQRQGDGASQNFNSNQSNGSQQSGFTSPQQTGNAPAANNTQADPFANNGQAIDISDDDLPF

>fig|1664.9.peg.626

MKNKIKQLKLPQFLDDARLRMVALVLLGLTAVSLIFAAMLNWLIALILFVLLIGTLITVLYAIETVTENTAKYVSDLSYRIKRGEQEALIKMPIGILLYNEKGEIQWTNPYLQQYLGNKEVLGKTIKEVDPELAELVVQNEDSNETKLVHWGDNQFQIIIQESIGVVYLLDITRYAAIEDRYEDEQVAIGQVFLDNYDEITQTMDDQAISNLNNYVTNELSRWANQFQMFLKRVDDDHFFVLAYAKSLHAAEKEKFKILDEIREETSKQNFPLTLSVGFAYGESDLAVLATTSQSNLDLALGRGGDQVVVKANDGQARFYGGKTNPMEKRTRVRARMISQALQEVFKQVDSIFVMGHIRPDMDALGASFGIRRIAEMNGKKCYVVVDQNNIHTDIQRLLEEVKGYPEIAVEVLSPEEAVEKATANSLLVMVDHSKPSITMAPKLYEKLAQRTIVLDHHRRGEEFPENPMLVYIEPYASSTCELVTEMFEYQPQNVANINKFEATTMLAGITVDTQSFSMRTGTRTFDAASYLRSVGADASMVQHLLKENVDNYIQKNHLIETIEMVEDNMALCMGEDNRSYDPVIAAQAADTLLSLSGIEAAFVIVKRPDGMIAISARSLGDVNVQVIMEKMGGGGHLSNAATQLKDATVSEAKAKLLAVLAELFDPEEAEENATND

>fig|1664.9.peg.627

MKVIFLEDVRGKGKKGQVKDVPDGYAQNFLIKNGKAKPATTAAVSALKGQQHAEAKNAAAELAEAKVLKTKIEDDKTIVEVKSKAGEDSRLFGSIPSKQIAQALEQQYKIKVDKRKIDLPEPIKALGYRNVDVRIHPDVTATIRVHIVAE

>fig|1664.9.peg.628

MVNNELIEHTPPQNNEAEQAVLGAVFINSDALVEAMEYVTADDFYRKAHRLIFETMVELNERGEGIDAVTIKSELDARNQLEDIGGIGYLAELADAVPTAANVVYYAKIVSEKAMLRRLIQTAQNIVAKGYAQDEDVTDILDTAEQEIMAVSEQQNKAGFKSISDVLTTSIEHIDKLYQEEESITGLSTGYPDLDKITAGLHEDELIILAARPGVGKTAFVLNIAQNIGKMTNENVAIFSLEMGAEQLVNRMLCAEGSIDANHLRTGQLEESEWQNLIIAMGSLSKANIYIDDTPGVKMAEIRAKCRRLAKEKDGIGLIVVDYLQLIEGSGQENRQQEVSAISRQLKKLAKELRVPVIALSQLSRGVEQRQDKRPVLSDIRESGSIEQDADIVAFLYRDDYYRDEPGEDGEDGGSQPAPDPENADVGEVEVIIEKNRAGARGTVKLLFVKTYNKFSSISYSQGEY

>fig|1664.9.peg.629

MADFQSDSARTIEPAPKLSHIKAAILGLQHLLAMYSGDVLIPLLVGAALHFNAAQMTYLVSVDIFMCGIATLLQLKRTPLTGIALPVVLGCAVEYVGPLTAIGTNSQLGIDVMYGSIIGAGVFILLISGVVARLRWLFPPIVTGSLITLIGFTLIPVAFQNLGGGDITAKSFGSVPNLIAGFSTIVLILIFSIWGHGFIQQIAILIGIIGGTLVGAAVGLVSLDPVGQASWFKLPELFYFGTPRFEWTSIASMSLAALTTMIESIGVFFALGEIVGRNITSNDLKRGFRAEGLAAILGGLFNTFPYSTFSQNVGIVQLSGIKTKKPVYYSAFFLIFLGLLPKIGAVATIIPTSVLGGAMVVMFGIVGIQGIKMLHKVDLEDNRNLLVATLSIGMGLGIAIHPTLLQALPASVQTILGNGLVVGSLTAVILNLLVNYRSLIEAKHQQ

>fig|1664.9.peg.630

MTPKTFVATLKEHQIDVSQKQLDQFEIYFERLVATNQNVNLTAITEKEAVYLKHFFDSVAPTLYLESLRTMPLNICDIGAGAGFPSLPMKILFPQLKVTIVDSLNKRIHFLEELVAELGLTDVTLVHDRAELFSQVKRPYREQFDMVTARAVAPMNVLAEFCLPAVKVGGQFVALKASQSDTELAEAAFAIETLGGQLKEDIAFNLPETNDPRHLVIIDKVQSTPDKYPRRAGVPVKKPLMAKEEK

>fig|1664.9.peg.631

MAISFFGKKKEETSSSVNQVVMVPVAAIVPNRFQPRKVFNTDHIGELASTIEQHGLLQPIVLREYEDQKFEIIAGERRFRAIQTLKWAELPAIVQKMDDHETASMALIENLQREELTAVEEADAYQNLMKLNGFTQASLAEKMGKSQSFVANKLRLLKLSQPVQEAIMNHEISERHGRSLLKLDEFNQQMVLHQILAEQLTVKDTEALVASIINPEPAVEEEVADVEEEPATIEESTETPKKAGKKKAPKKEMTIADTKVAVNTIKKSIELIEQSGIPVSAVEDDQKDVYRITIEIPKK

>fig|1664.9.peg.632

MAHVISIANQKGGVGKTTTTINLAVCLADAGNRVLIIDSDAQGNATSGIGIQKSQVEKDIYDVLVDEIPIKEAILKTNHQHVDIVPATIQLAGAEIELTAQMAREMRLKLGLEAVLNDYDYVLIDCPPSLGQLSINAFTASNSILIPVQSEYYALEGLSQLLNTVRLVQKHFNPNLAIEGVLLTMYDARTNLGAQVIEEVRKYFGDRVYDTIIPRNTRLAEAPSHGVSIIDYDPKSRGAEVYQELAKEVLAANGQ

>fig|1664.9.peg.633

MVNKNGKGLGRGLEAVFSAFEGPNKNRADELVEELALEDIRPNPYQPRKTFDEAALAELAASIKKSGVFQPIIVRKSINGYELIAGERRFRASKLAGKTTIPAITRQFDEEAMMEIAVLENLQREDLTPLEEAQAYDTLLKKLNLTQAEVSERLGKSRPYIANYLRLLGLPADVKQLLQEGQLSMGQARTLLSLKKKSQISALAKRTIAESLTVRQLEQLVNQMNQTKAAKVAKTKIKSPYIQASENQLVDKFDTKVNIAMNSKGRGKIEIDYGTVEDLNRILALLDVNID

>fig|1664.9.peg.634

MYALGDLVQMKKQHACGTNRFEVVRLGMDIRIKCMGCGHAVLLPRREFEKKQKKVLVAAADVDTTTEPHYQLLSKGPNQTNF

>fig|1664.9.peg.635

MSLTAGIVGLPNVGKSTLFNAITKAGAEMANYPFATIDPNVGMVEVPDKRLDRIQEIIPAKKIVPTTFEFTDIAGIVKGASKGEGLGNKFLENIRQVDAIVHVVRAFDDDNITSVTGTVDPLDDIDTINLELSLADLESVTKRYARVEKIARTKDKEALAEFEVLKKIKPVLEEGGSVRSIEFNEDEAKMVKGFFLLTSKPVLYVANIAEDAMADPDSVDYVKQIQDFAAKEGAEVIAISARTEEEIASLDDADKAEFLEAEGVTESGLDKLIRASYHLLGLATFFTAGGKETRAWTFRQGMKAPQTAGVIHSDFERGFIRAETVSFADLDQYGSMQAVKEAGRLRLEGKEYMVEDGDIIEFRFNV

>fig|1664.9.peg.638

MTKGTVLFDLDGTIADSQKGIINALEYMIEELALPTQTTAQLISFIGPPLNETIMKVFSLDEAATQQAIKTFQAYYAPKGLYENELYPGMHETLAALQAQDLQLAIATSKPEPFAKKIIDHLELTPYFSGVYGASVDATTRVKKADVITYALAELGLSTKTEPLLMVGDRQNDILGAKQNEMAAVGVSYGFGSIAELKKAGAVQIVTQPTDLITTLPANLKL

>fig|1664.9.peg.639

MMTNLSILYISLAGNTRSFVTDLQDYAQQQHTTNPALPTIKLTEISDATPLKDETAPFYAFVPTYLDGGNGIDNGVKELMTNSLGEYIAYGHNADLCYGVIGSGNRNFNEQYCLTAKRYAEQFNAPFIADYELRGNSRDVGRIYMLLVANAQKHA

>fig|1664.9.peg.646

MLVTFFLAAFFVAAEFALVQTRPSALEDAIHSGNGSPKKLKLALKMTQNLNEYLSTTQVGVSVAGIILGWIGESTVETVLVDLLGLTHLVSESGLHIIGAVAGVLVLTYLEVVFTEIVPKNISIDIPMQMLMAVAKPLHYCHIIFYPFVWLLNVSANGVVKLIGFAPADESSDVLSQAEIISLSKNAVTGGELDRNDLVYMQRAFDFNDRIAKDVMVDRTSLYVVDITDTVKDVVKDYLQQGFSRFPVVAENDKDKILGYVYAYDLVRQAQVDDSIRVSKLLRSIISVPETTPIHSLLTQMINKQTPIVVVVDEYGGTSGIVTDKDIYEELFGTVKDEIDDVSDEYIIKEDDNRYRVSGKTTLYDFERFFKADIEAFEASDSVTVAGYVLENFANLHQDSVVSIDRFDLTVAEFNRGYIDWFEVSVNPQRADTTL

>fig|1664.9.peg.647

MTTLTTTYTLANGVQIPVLGFGTWQTPDGDTAIKSVKAALEAGYRHIDTAQAYQNEASVGTAIQESGVARQDLFLTTKIWNANHSYDLTMQSFEESLQKLQTDYVDLLLIHWPNPIDFRDNWETANAETWRAMEELYNAGKAKAIGVSNFRAHHLEALKKTAKVQPMVNQIFLAPGELEAETVQYSRDNGLLLEAYSPLGTGKIFDVPEMKTLAAKYQRSIPQIALRWSLQHGFLPLPKSVHANYIQENTQLFDFELTPEDMQTIDQLDGVVGKAKDPDTATF

>fig|1664.9.peg.648

MPNAQRQNSALYKLTRPANQLTAEQHQLKTAIINFCEQNRNADHATFLIKGAAGTGKSVILNSVFSTLQELARKTPTSPLYQTNNKLLVNHPEMLKLYQNNVQPTNALLKKDFQRPTTFINQASKQETRADIVLVDEAHLLLTHSDPYNHFRQDNQLDEIRKHSRITILIFDEQQVLKVKSYWQQQQLLAKLQTGPYQLAQLHHQFRIQANPSVESWLQAFHKMQIEALPQDPHFEFKIFADAAQLYAAIRQRNQDSGLARLLATYDFPATLNDPHDHYVTAPHFKLRWDRYQPTAKTYWADREDSIDEVGSVYTIQGFDLNYAGVILGPSIGYDPQQDCLLIKPEYYEDQAAFAGANRFDNPTQIKTQIILNSLYVLMSRARNGLYLYALDPALQKRLLALQTATFPGQ

>fig|1664.9.peg.651

MPTLKEQVIAASREIGIDKIGFTSAAPFDYLEASLIEQKKNGHSSGFEHKVLEERLYPELIFDQPKSIIAIALAYPTKIHNKPARTGAKRGSFARASWGIDYHDILRDKMAQLIDAIKELADEDDEVTFKPMVDTGELSDVAVAQRAGLGFIGLNGLLITPEFGSFVYLGEIITNIPFEPDTPMANQCGSCTRCIDYCPPKALLGDGRLNATRCLSYQTQTKGFMPEEYRPKIRSVIYGCDICQQVCPFNKGEDFHFHPDMEPDPEVVMPELQPLLTISNKEFKVKFGHLSGSWRGKKPIQRNAIIALANVKDRTAIPQLLQLIDNDPRPVIRGTAAWALGQLVRDVTPEMLDFLQQAADKEEEPEAQVEFHKALLILNERFEER

>fig|1664.9.peg.652

MTQRILPLEKGHNFRELGGYQTTDGRTLKWHKLLRSANLAHLTDADLNYLDQYGLRYDIDLRSEDEKAKAPDRLPANATYEFLPVFAVDETANSASQDELYQLFSKDPLSGHTRMQKVYDNLINQPHSKQAYRRFFELLLANDQDHQTTLFHCTAGKDRTGMATVFLLSALNVPAETIQADYLLTNLASADFVQKSLQRLQQKTADQGIYQSVQSLLTVHLDYLQTAQMAIRQESGTIQNYLKTELQLTNHDLTDLQKIYLM

>fig|1664.9.peg.653

MITYFELQTTGEIHENRSEKAANWIHFEDPSKHEMQHFARKYHLPRMLFEVAQDINEVARYETFTTETGETLSHICLLVPVKTHDSEKHAEYITRPFSFIISGQTLLTVGHQQSELVAEMQRITGTDQQVESIVLQSISLIYQRYLTALDVVAQQIHQLEKNVHISTRNQILYALKALKKSVVYLDLGLKSNQQSLPQIKETALFEEVADQKDRLYRIDLQFAKSDKAITTYRALLEQLSDLLSDIISNRLNNIMKTLTSISIVLTVPTIIGGFWGMNVPVPLTHSGKGFILLLGLSGIVSALVGWWLKKKDYF

>fig|1664.9.peg.654

MNSMVQEYKRILVPVDGSEEAELALNKAVQVAKMNHAQVDILNVLDTKQYAGSYSGMLSGDAIFQISEDAQNYLNSLKYDIQTNDNLMDVEIHVRFGNPKNVIARDFPSEYKTDLIMMGSTGLNAVERMLMGSVTEYVNRTAICDVLIVKTDVNNNPYKK

>fig|1664.9.peg.655

MYNAKETRYDDIIYRRVGHSGLKLPAISLGLWHNFGETDKVATQKEIIFGAFDMGITHFDLANNYGPPAGSAEENFGRILHSDLKQYRDEMIISSKAGYYMWPGPYGEWGSKKNLIASCDQSLQRMQLDYVDIFYSHRPDPETPIEETARALDLLVHQGKALYIGISNYSAEQTKAITKIFRELGTPFIIHQPRYNMLDRWIEDGLTDVLAEEGLGAITFSPLAQGMLTNRYLNGIPADSRAARPDSPFLSPEKVDQTISTVQELNKIAQQRGQSLAEMALAWNLQQPTVASVLVGASRLSQLQDSVHALDNLTFAPEELAAIQKVLK

>fig|1664.9.peg.656

MSILMGIVGLVFIFAIGWLVSSNRKKIRYKQIGILLVTQFVISFLCLHTSGGVKVLAGISNFFSWLMGQAAGGVDFVFGGVVIKSGASVFFLNVLMPIVFISALVGILNYIKVLPFIIKWLGKGINKLSGMGELESYFAVSTAVLGQPEVFLTIKERIPNLSPQRLYTICASAMSAVSAAMLASYMKMVPGKFVVVAVFLNIFSALIISCIVNPYEPEVEDQKTIEAHLSTEKEPFFQVLGNYIVDGFQLALTVAAMLIGFVSLVTFLNNTFGFFFNVTFTDIIGWVFAPVAFVIGVPADDIVKVGSLMATKLITNEFVAMGNLSQIAHSLSPKATAIISTYLVSFANFGTIGIISGSIKAIDQKQGAVVAKFTMKLLLGATMASMLTGTIVGMYF

>fig|1664.9.peg.657

MDYKLVQKTKVLVTDDLPKTILSILKETQYKKPLIVMDSFLKEVAIVKRLQVKLTENNIQFSIYNKVVSDPPTTVVDEGAEFFKAQDCDSLIAIGGGSAIDVARGINIVRINGGKISEYVTASNEIKDCPGLIAVPTTSGTGSELSNALIVTDTENQTKLAVLANNAVSEFAVLNPELVMTLPKGMTIATGLDAFSHAAEGYTSTLASPVTDAICEKIMYLLVKYLPKVIEDGQDREARERVMVAATLGGWMLNNAGTHAGHSVAHVIGSKYHMPHGMACAYALPGVLQFIAPVRSKKVKEIGLILGAEYPADPTDTEIGRITAIQYREFRDKVLGMKPFTDFNISEDELLTNIESVVNERFAGNTPKQIDQEGAMMLLKNFG

>fig|1664.9.peg.659

MIKKQIYSLITYDESVKTMEAGADHIGLVPMQDGGVPAHRVPLETVDKIFAEAKRRGVVTVAILLNKDPKEMLDIAKRVRPDILHIAGMEYTADQAFRDQLRAVCPETELMQAVLVDGPEAVERAKEYAKFSDYLLTDSGLAADTGIGASGLTHDWSIDAAIVANVDIPVIIAGGLGPDNVEECIKEIKPYGVDSLTKTSIKYDGGRMEKDIDKVREFCERADKAAAEVGL

>fig|1664.9.peg.660

MEDKYVVVVGGLNMDIAGMPGKDFIERDSNPGEVNLSVGGVGQNIAHNLANLGVPTYLMTVYGDDQYGAILHQECETNKINLDYAAEIKGANSSTYLYVTDGSGDMLAAINDMSIVNNITPDFLKERLAVINQATLCIVDANIPQESIEWLADHLKVPMYVDPVSVAKARRFENALNKIDTFKPNEMEAELLTGIKISDESSAKMAAECLVNQGIRHVFISLGAHGILCADESQTVLVPIIKVPIISCNGAGDCSMATIAWAYYHFGKKASLKEIGQYAQAASSITVGSASAVSSHLTAQNVIDRCRQAVKN

>fig|1664.9.peg.661

MTKREEEILKIIKENPLISQQELADKFGLTRSGVAAHIFNLTKKGYIAGKGYIINEPNFVTVIGGVNIDILGLTDTTLIQQNSNPGHITFSLGGAGYNIAHNLTKLAVPNYFITVYGDDLNGQKFEEDAQKNHLAIQHSKKIPDKSTSSYLYVNDPDGTLNVGVDDMGIYDDITPDFLSERLATINTSAYCIIDTNLPEKTIDWLFDNCKVPIFVKTVSLNKSYKLQNVLSKIDTLMTTDKELAMLTNSSVTDAKTAEKSAKKLLKKGIEHIYVLMPHTGLFYIDNRNTKLIAGIPIKRINNNGASAALTAAVVDSRLNNLGWEKTTQIAYTAALICMESAKSVNPLLSKDYLLKTVTRYLES

>fig|1664.9.peg.662

MTKKIILDCDPGHDDAVAMMLAHGNPEIDLLAVTTVAANQTLTKVTRNALAVATMIGMHDVPIAAGCSRPLIEEIKVAADIHGESGLDGVVLPKPTVELEKIHAVDLIIDLIMSHPAKTITLVPTGALTNIAMAARKEPQIIDRVKEVVLMGGGYHEANASAVAEFNIKFDPEAAKIVFNAGWQVTMVGLDLTHQALATPDIVSEIKAIHTETSAFVVDLLAFFRDMYKKGQNFDAPPVHDPCAVAYVIDPTVMTTKKVPVDIELNGTLTRGMTVADFSYPIPEDCQTSVAVKLDHAKFWGLVEDALRTIG

>fig|1664.9.peg.663

MIEAVNLRAGMTFEQDGKLIKVLEANHHKPGKGNTVMRVKLRDLRTGSIVETTMRPEVKVEQAMIDTKDVQYLYTQDNVAFFMDLETYEQYEIQTSAIEAELKYLLENMNCKIQFFGSEVIGVTLPTTVNLRVVETQPSIKGATVTGSGKPATMETGLVVSVPDFISADEVLEINTQEGTYVKRAAK

>fig|1664.9.peg.667

MSILNMRRVLVGVDDSADALLAFEYAIKRAVKDDLELVIVSVLENDELNVYQALNKDYIHGQYNDLEKHVLDYQEQARAEGVKNVRAVIAEGEPGEVIIKEVIPKVQPDLLIIGSKAKEGIAKYFGSQAAYMAKYAPVPVLVVR

>fig|1664.9.peg.668

MNKVKGPKKHKLIEYANGPSLEEINGTVEVPEGKTFWKTLLAYSGPGALVAVGYMDPGNWSTSITGGQSFQYLLMSVILVSSLIAMLLQYMAAKLGIVTQMDLAQAIRARTSKSLGIVLWILTELAIMATDIAEVIGAAIALYLLFNIPLVIAVFITILDVMLLLLLTKVGFRKIEAIVVALIVVIFVVFAYEVALSNPDWAGVIVGLVPTAKTFATTPNVGGMSPLTGALGIIGATVMPHNLYLHSAISQTRKIDRNNEEQVAQTVRFSTWDSNIQLTMAFFVNALLLIMGVAVFKTGAVKDPSFFGLFEALSDTSTMSNGILASVARTGILSTLFAVALLASGQNSTITGTLTGQVIMEGFVHLRMPLWLRRLVTRLLSVIPVLICVMMTSNKPPLEEHQALNTLMNNSQVFLAFALPFSMLPLLMFTDSRVEMGDRFKNSLVIRVLGWLSVIGLTYLNMLGLPGQIEAFFGDHATAAQLALADHIAYVLIAAVLALLVWMIVELYKGNQRFEQQLAAQAAE

>fig|1664.9.peg.673

MKDILRPIGIIARSLDSIANIEFKKFDLTRGQYLYLVRIAENPGIIQERLIELIKVDRATVTRAIQKLSRAGLITRQADETNLKIKHLYLTPAGQAIYPEIKRENDYSNQVALQGLTTAETQVLHELLTKVSTNVAADWEQVKKGQQRPY

>fig|1664.9.peg.675

MALSIGEVAQQYDLSIPTLRYYEEQGLLPFIKRSPAGRREFRQADLDCLCDIECLKKTGMQLKDIKQYIDWRTAGDETLDQRLALIQQRRQMLASEIESLQAELTKLDHKEWYYTKASEAGTEAIFTAGCDTEYERAHQNK

>fig|1664.9.peg.676

MQKLSALTAPMALSNGVTIPGLGYGTYQTPNEETKKAVLEALSVGYRHIDTAAVYGNEQGVGDALKESDIAREDIFVTSKLWNTERGYDATKAAFAKTIATLGVDYLDLYLIHWPANTKQFGAKAAELNAETWRAMEDLYNEGKIRAIGVSNFMPHHLDELMKTAVIKPMVDQIEVHPGWPQAEAVRYNQAHDILVEAWAPLGEASALSNETIAAIATKHGKTAAQVCLRWGIQQGVLPLPKSTHQERMAQNTDIFDFELTDAEMTQISALENLGGQCMVPDEVDF

>fig|1664.9.peg.679

MIKKKAIMIGAGLSNMAAAVYLIQEGGWSGDAITFYSLDDHGSNDGAPVSETTDEYWNKNHPLENTKGYVARGGRMLNYRTYVDLMDLLDRIPSATEPNMTAEEDTRSFDAQHRTFDKARLLEGSKGILDAGHLGFNNKDRLLMTKLIMMPDSEEEKLDSVSIAEYFKDDPHMFQTNFWYMWETTFAFRTQSSAQELRRYMHQMIYEFTQIEHLVGVNRTRYNQYESIMLPLINYLKDQGCRIILNRRITDLDFKETQMTDEITVTGMTMTNTETDEVEHITVDDDTAVIFTNGSITDSATLGDYNTPAPENMDYGAAAGLWKQIASKFYNLGNADKFFADRNASEWVSFTLTTKNHILLNEIERITTQIPGNALNSFLSTKPITPLGQKDVNMSIVVHHQPHFTTQKPNEAVIWGYFLYPRRSGEFVDKPYIEMTGKEMLQELIGQLAKVDPGPINIREKEAEIMDSIINNIPVYMPYASALFNNRAKTDRPEVIPEHSTNLAFTGEFVEQPYQMIFTEQSAVRSGEIAAYHFARIPMAKLVDTPRYDRDIKTLMRATKKMFE

>fig|1664.9.peg.683

MNIKEASQQTGVTATTIRYYEKEGLIPAIDRNDVGVRAIDDRIIRRINFVKTMRAAGMSIEALKEYIRLFDAQEDNTAQQFKLLNEQIAIMEEKRDDLQTAIDHLRWKVDHYEDHMAVTEEELRAVEKNRADQ

>fig|1664.9.peg.685

MEVIKMKKTNKKVILAIILISYFMILLDNSIIFTGTVKIAAELNLSQTTLSWVTNAYSLTFGGLLLLGGSIGDIIGRKRVFMIGLTVFAVGSLLVGLANSAVMIILARAFQGIGSAILAPTTLALLMDSFSGEARTRAIAVYGATAGIGASIGLVIGGIFASLLSWRDGFFINVPIAIIMIGLTVLFIPTTQATQAKRLDLIGALTSIIGMTALVYRLVGQQGRLLALLVAVVAIAAFIWQEARTKQPMMPLRLFADCERLGGYIARFFYLGAMLSMWFLTPQIMQTHLGFTPLQAGIGFFPLTVVNFIVALQVSKLTTRFGNTKLLTVGIATTAIGMCALGFFSEQVGYALGIAVPMILMGIGQGLTLSPLTVAGVANTLPADAGAASGVVNMVHQIGGSIGMSFIVAVSATFKNGLTSYHVAMGLATVLLIGALLAVIGLILPTLKKESE

>fig|1664.9.peg.686

MAELMRFYQSFQPEHYQVFLDINRAQKRFSGRTTITGTAKDAVVLIHQNGLMIETVSAAGKSVPFTVDNDANALKVEVGQPGEVTLVISYTAVLTDSMMGIYPSYYQVDGVQKELIGTQFETNFARQAFPCVDEPEAKATFDLAIKFDEHEGETILSNMPEKEVIDGVHHFETTVRMSTYLVAFAFGELQGAQTKTKSGVQVGVFATKAHQANELDFALDIAKRSIEFFEDFYQTPYPLPHSWQLALPDFSAGAMENWGLVTYREAYLLLDPDNTALTTKHRVATVIAHELAHQWFGDLVTMQWWDDLWLNESFANMMEYVAIDALEPDWHIWESFQTSEASSALQRDATDGVQSVHVQVNNPAEIDALFDGAIVYAKGARMLVMVRTLIGDDALRAGLKAYFAAHQFGNATGADLWAALGKAANLDVGAIMDSWLEQPGYPVVTAKVVDGQLTLSQQQFFIGEGQEVGRQWQIPLKSNYAATPSLMTDRKLVLGDYARLRADNGQPFRLNVGNESHFIVNYDQTLFDDILKDSAEMAPIAQLQLLQDLRLLAEGRQINYADVVPVLAPFAKSNSNLVADALYTVAGNLKKFVTAGETSEQHLRTFFDKLSKAQVARLGWTVQPTDTNDDQLMRPTVLSAALYAKNQAAIDAAHALFQANQDQLATLPAAIRVLVLMNEVQNFGNAALYSQLLEAYRQTSDASYKNDLSAALTWTTDPELIITLISKFEDAGTIKPQDLRAWFRGLLANDASQQATWDWIRNDWQWLEDTVGGDMEFTTYITSIAAVFKTPERLAEFKAFFEPKLDTPGLTREITMDIKVIESRVALIESEKEKVNAAIAQQI

>fig|1664.9.peg.687

MSIQNKVVIITGASSGIGAATAKLLASKGAKVVLAARRKDRLQALVAEIGENAIYQATDVTNRDQMASLAQLALDRFGRVDVLYNNAGVMPQGNLSERDYDQWQKMLDINIMGVLNGIGAVLPTMQAQQDGLIISTDSVAGHVVYPASAVYNGTKYAVRAIMEGLRQEEKDNGIRSTIVSPGAVKTELVNTIGNTAVFEGVKQLMDAPEESALALNPEDIANAVLYAIDQPKHVVVSEVLIRPAKQQV

>fig|1664.9.peg.688

MDYSLRTRIANQPIYRDSELFKEIHLVKGNNERLVMEMNTGYHDAAEVRAYLKCITDQEIDEPVTVSLPFYTDYAKHITFGKEIFINNNVLFVDLGGITIDDHVLIGPRASLITVNHLENPADRRGLFVKPVHIKQNAWIGAGATILPGVTVGENAIVAANATVTKDVPANMIVAGTPAKVIRQIKAEKEF

>fig|1664.9.peg.690

MEKTEKIAILEKLVSIDTTDQAEGVIADYLADLFTQHGIQTEKVASKPGRENLVAYLGEATDKVLAVTGHMDVVSIGDRSKWTSDPFTLTARDGKLFGRGATDMKSGLAALVIAMIELHDQNVPLNGQIKLLATVDEEKNETGAQTLTAQGYADNLTALLVAEPSGVDKQALANSTDKFPTEMVQKLLAANQTNEQHFLIFAHNGSLDFKVTATGKTAHSSMPELGINAIDHLLAYYNRQKQYFDQKHPIDDVLGDIVPVTTLINGGEQINSVPGHAELTCRVRTTPALTGDQVIADLNAIIAELNQQTDMNLALTVINNQPPVKSNPQAPFIQSVQKIGAQKLSQAYPLMHVAGGTDAAHFAKNNPNLPVAVVGPGNDTSHMIDEYVDEEMYLKHIDFFKAVMTDYLK

>fig|1664.9.peg.691

MRERVQEKSVEVTVLLLGIIFVATNLRAPLTAVGPLTGLTKDNIGISGTIAGFLTTIPLLAFAILSPVAPKIAEKLSLEYTIGYSTIVLVIGLFIRSIPQTSTLLFGTILVGSAIAMCNVLIPSLIKREFNHRLGLITGIYSISMNLCRAIASGVSFPLANNLNLGWNNSLRIWLILALIACIAWIPQLKRHDKPDKIEELPIENSIWHSKTAWQVTIFMGLQSLVFYVLVAWLPEMLIQKGFTAEQAGYLLSMMQLFLLPFTFIIPIIAGRVKRQSKIAINTSILMCLGISGLFINNTIIIIIGIAFIGISGGCAFGLSMMFFNLKTRNAREGAELSGMAQSIGYLLAAIGPTLFGFMHDMTHNWNVSLIILIVTAILLGGFGFFAGEDNYIN

>fig|1664.9.peg.693

MMTAQILNEANFNDEIKEGVTLVDFWATWCPPCKMQGPIVEQLADDYAGKAKIAKVDVDQNQNLAAQFGIQAIPTLLIMKDGQLQEQLVGLQRKEALATKLDATLA

>fig|1664.9.peg.694

MEEQILQYRDAVYEQVVKVGKSLGNVTRLRILDLLVQGPKTVENLANTIGLSVATTSRNLQILKKARLVEIERHKNFVTYRLTSDQIKQLISLLVTVAEQSQPELAAIQTTFKQQTGSPQALTISALKAKLTAESTYLVDLRPNDEYETQHLPGAHNIPYDELPQHLAELPQDKEIVVYCRGRLCAYANVASQALHDAGFKVATFNQSVWEWNQLMSS

>fig|1664.9.peg.702

MNKLILGRYIPGDSILHRMDPRAKLIASFYYIGIIFLARSWPAYLLLFVFTMALILISKIKLSFFLKGVRPLLWLILFTVLLQIFFTRGGTVYWQWGFLSLTKFGLLNGAYIFMRFVLIIFMSTLLTLTTPPLSLADAIESILKPLKVIHFPVYEVALMLSIALRFVPTLMDETTKIMNAQRARGVDFGEGSLLQQMKSIIPILIPLFVSSFNRADDLATAMEARGYQGGEGRSKYRVLKWQTRDTLAFVAMAVLTVGLVILR

>fig|1664.9.peg.703

MDITFKDVSYTYQPGTPFQGIGLKHINLTIESGSYTALIGHTGSGKSTLLQHLNALLKPTEGIVQIGDRQITPETNNKNLKIIRQKVGMVFQFPESQLFEATVQKDIAFGPQNFGVPEAEALERAKAMVELVGLPEAVLEQSPFDLSGGQMRRVAIAGVLAMQPEVLILDEPTAGLDPVGRREMMGLFEKLHREQGMTIVMVTHQMDDVANYADHVVVLENGGIAKSGTPREIFADPEWLTSKQLGLPTTTQLAQALIKKGFVFPKVPLTEHELAAMLRDQLPQGAGGANE

>fig|1664.9.peg.704

MDENKIIEVAHLKYEYPQASRLALNDLSVSINAGEWVAIIGHNGSGKSTFAKSLNGLLDLQSGDITIDGLPLSIETVWDIRRKIGMVFQNPDNQFVGATVEDDVAFGLENQGIERTEMQRRVQDAVDRVGMTQFMTREPSRLSGGQKQRVALAGIIAQQPEILILDEATSMLDPKGRQEVLETIHTLKQETNMTVLSITHDIDEAASADRIVMLDKGQVIDQGTPAEIFAYGQRLLDLGLDVPYPEKLKAALTKLGVPMPADYLTTERMVDHLWTLHSKM

>fig|1664.9.peg.705

MSYRKLGRTSSQRKAMLRDLTTDLIINERIVTTEARAKEIRKTTEKMITLGKRGDLHARRQAAAFVRNEVADIREEDEAVIVQSALQKLFSDIAPRYAERNGGYTRILKTAPRRGDGALMVIIELV

>fig|1664.9.peg.706

MIEFEKPNITKVDESTNYGKFVVEPLERGYGTTLGNSLRRILLASLPGTAVTDIQIDGVLHEFSTIDGVLEDVTQIILNIKKLALKLHVEEDKTIEIDVKGPATVTAADIISDDDVEVLNTDQYICTVAEGGNFHVRMTVKKGRGYVAAVQNKSDDMPIGVLPIDSIYTPISRVNYQVESTRVGRRNDFDKLTLDVWTNGSISPREAISLAAKIMTEHLAIFVDLTDEAKNAEIMVEKEETHKEKMLEMTIEELDLSVRSYNCLKRAGINTVQELTDKSEADMMKVRNLGRKSLEEVKNKLFDLGLGLRTEE

>fig|1664.9.peg.707

MANKKNAPRKRRVKKNIEAGVAHIHSTFNNTLVMITDPNGNAVAWSSAGSLGFKGSRKSTPFAAQMAAEAAGKEAMEHGMKSIEVAVKGPGSGREAAIRSLQATGLEVTAIRDVTPVPHNGSRPPKRRRV

>fig|1664.9.peg.708

MARIAGVDLPRDKQIVIALTYIFGIGNTTAVKVLADAGVPTDVRTRDLTPDQEDKIRAALDTVKVEGDLRREVSLNIKRLQEIGSYRGMRHRRGLPVRGQNTKNNARTRKGKKVSIAGKKK

>fig|1664.9.peg.709

MAKDDVIEIEGKVTDTLPNAMFKVELENGAVILAHVSGKIRKNYIKILPGDRVTVELSPYDLTKGRITYRFK

>fig|1664.9.peg.710

MNLMLMGLPGAGKGTQAEKIVDAYHIPHISTGDMFRAAMADQTDLGVKAKAFIDKGELVPDDVTNGIVEERLSQADTNVGYLLDGFPRTLDQADALAVITDKLNKPLDGVINIDVDPEILADRLSGRFICKTCGATYHKLYHPTQVEGTCDRCGGHVFFQREDDKPETVKNRLKVNIEMNTPLLDFYEKRNLLYTVDGNQEIDDVFAAVKKVLDTIKD

>fig|1664.9.peg.711

MLKTLRNAFKVKEIRSKILFTLGVLIVYRLGAQITVPGVNAGALTKLGSTGLIPLLDTVSGGGLANYSIFSMGVSPFITAQIVVQLLQMDIVPKFVEWSKQGEIGRRKLNQVTRYLTIILAFIQSIGITAGFNSLSQMGLVKDPGIKTFVSIGIILTGGTMLITWLGEQITDKGLGNGVSMIIFAGIIARLPSGLHQIIKEHVLNASKGEMWLGILFVVGLIVAVLVIVTFVTWVQQANRKIPIQYTRREAGAGDNSYLPLKVNVAGVIPVIFASSFIMTPQTILMAFQASHGEDAWFKVMSDIFSMQTVTGSIVYTVLIILFTFFYAFVQVNPEKVAENLTKQGSYIPGVWPGKGTEKYLSSVLMRLSTVGALFLGVIALLPQLAANLGGLPQSIGLGGTSLLIVVGVALESTRQLEGLLMKRKYVGFIR

>fig|1664.9.peg.712

MKLHELKPNEGARDVRKRVGRGTSSGTGKTAGRGQKGQKARSKVRLGFEGGQMPLFRRMPKRGFKNINRKEYAVVNLNDLNRFEDGTEITATVLIEAGVVKNELSGVKVLANGELNKKLNIKVSKYSEAAKAAVEAAGGSIEVI

>fig|1664.9.peg.713

MAKLKITLRKSAAHRLPEQRKMVKEFGLNRVNSSVIKPDDAATRGVIFKLAHLVTVEEIKD

>fig|1664.9.peg.714

MTYIDPTHLDLEDRVVSINRVTKVVKGGRRLRFAAIVIVGDKNGHVGFGTGKAQEVPEAIRKAVEDAKKNLINVPKVGTTLPHEVIGRFGAGRVLLKPAVEGSGIAAGGAVRAVMELAGIDDVTGKTLGSKTAINVIRATIDGLTRMKTAEQIAELRNISVESLQN

>fig|1664.9.peg.715

MISKPDKNKTRQKRHTRVRGKISGTADCPRLNVFRSNKNIYAQLIDDVAGVTLASASTLDKEVKVEGTKVEQAQQVGALVAQRAVKAGHKVVVFDRGGYLYHGRIAALATAARENGLEF

>fig|1664.9.peg.716

MSRIGLKVIEVPAGVTVTKDGENNITVKGPKGELTRHFNPIIEMHEEGNLINFTRSSDSDRAMHGTMRANLNNMILGVTEGFKKTLDLIGVGYRAQLKGKTLVLNVGYSHPVEMEAPEGVNVEVPSNTNIIISGISKQKVGQFAAEIRDVRPPEPYKGKGIRYTDEHVRRKEGKTGK

>fig|1664.9.peg.717

MVMTDPIADYLTRIRNANMVRHESLEVPASRIKKDISEILKREGFIRDYEVIEDDKQGIIRVFLKYGKNNERVISGLKRISKPGLRNYVKANEVPKVLNGLGIAIISTSNGVVTDKEAREKAAGGEVLAYVW

>fig|1664.9.peg.718

MTNRLKEKYVKEMTPALIEKFNYTSSMQVPKIEKIVLNMGVGDAVSNAKNLDKAVEELGLIAGQKPLITKAKKSIAGFRLREGMPIGAKVTLRGERMYDFLDKLVNVSLPRVRDFHGVSAKSFDGRGNYTLGVREQLIFPEIDYDKVDRVRGLDVVIVTTSNTDEEARELLTQFGMPFAK

>fig|1664.9.peg.719

MFVKTGDKVKVISGKDKGKEGTIIKAMPKEGRVVVEGINTIKKHVKPNAQNPNGGIVDTEASIDASNVMLIDPSNNEATRVGYKVVDGKKVRVSKKTGESIDK

>fig|1664.9.peg.720

MIQSESRLKVADNSGAREILTIKVLGGSGRKTANIGDVIVATIKQATPGGVVKKGEVVKAVIVRTKSGARRVDGSYIKFDENAAVIINDDKTPKGTRIFGPVARELRDSDFMKIVSLAPEVL

>fig|1664.9.peg.721

MSEESRNHRKVYQGRVVSDKMDKTITVMVETYKTHPEYGKRVKYSKKYYAQDDNNEAKVGDVVRVMETRPLSRTKRFRLLDVVEKAVII

>fig|1664.9.peg.722

MKAKDIIELTTAEMLEKEHQYKEELFNLRFQQATGQLENTARLKQVRQNIARIKTVLRQQELNK

>fig|1664.9.peg.723

MLVPKRVKHRREFRGKMRGAAKGGKEVTFGEFGLQALESSWITNRQIEAARVAMTRYMKRGGKVWIKIFPHKSYTAKGVGVRMGSGKGAPAGWVAVVKREKIMFEIGGVSEEVAREALRLASHKLPVKTKIVKREEVGGESNEG

>fig|1664.9.peg.724

MGQKINPTGFRVGVIRDWDAKWYAEKDFATFLHEDLKIRKYINTKLADASVSTIEIERAANRVNVSIHTAKPGMVIGKGGSEVENLRKALNNLTGKKVHINIVEIKKPDLDAHLVGEGIARQLEARVAFRRAQRQAMQRTMRAGAKGIKTQVAGRLNGADMSRIETHAQGTVPLHTLRADIDYSWDEAMTTYGKLGVKTWIYRGEVLPAKANNNTKGGK

>fig|1664.9.peg.725

MADQITSATASAMSVRMPARKVRMVIDLIRGKSVAEAIAILEFTPRAASPVVIKVLKSAIANAEHNHDLDAENLVVTKAYANEGPTLKRFRPRAKGSASPINKRTSHITVVVSEKEA

>fig|1664.9.peg.726

MSRSLKKGPFADAHLLNKIEAQADSEKKQVIKTWSRRSTIFPSFIGYTIAVYDGRKHVPVFISDDMVGHKLGEFVPTRTFHGHGNDDKKTKAR

>fig|1664.9.peg.727

MGIIKYKPTTNGRRNMTSSDFAEITKTTPEKTLLESQSHTAGRNAHGHITVRHRGGGHKQYYRVIDFKRIKDDIKATVKSIEYDPNRTSNIALIQYPDGIKAYIIAPKGLEVGMIVESGVNADIKVGNALPLANIPDGTLIHNIELKPGKGGQLVRSAGTSAQLLGKEGKYAIVRLTSGETRMILLTCRATVGTVGNGQHELIKIGKAGRKRWMGIRPTVRGSVMNPNDHPHGGGEGKAPIGRPSPMSPWGKKTLGKKTRSSKARSEKLIIRHRKSR

>fig|1664.9.peg.728

MEARDVILRPVVTESSMAAMDDKKYTFDVDVRANKTQVRYAIEEIFGVNVKNVNIMNVRGKLKRQGRYAGYTKKRRKAIVTLTADSKEIKIFED

>fig|1664.9.peg.729

MANVTLFKQDGSQNGNVELNDSIWAIEPNENVVFDAIIMQRASLRQGTHAVKNRSAVRGGGRKPWRQKGTGRARQGSIRSPQWRGGGVVFGPTPRSYSYKLPRKVRRLAIKSVLSQKVIDNDLIVVDSFSFDAPKTKEFAEVLNKLDVNTKVLVVLEDGNDFTALSARNLPNVTVVPADGINVLDVVGNQKLILTQAALSKIEEVLA

>fig|1664.9.peg.730

MTTKGILGRKVGMTQVFTENGELIPVTVIAATPNVVLQVKTNETDGYEAIQVGFEDKREVLSNKPAKGHVAKANTTPKRFIREFRDVALGDYEVGTEIKVDTFAAGDVVDVTGVTKGHGFQGNIKKDGQSRGPMGHGSRYHRRPGSMGAVINRVFKGKLLPGRMGNNQRTVQNLVVVSTDVEKNVILVKGNVPGAKNSMVTIKTAVKAHK

>fig|1664.9.peg.731

MAKQKIRIRLKAFEHRILDQSADKIVETAKRTGASISGPIPLPTERTLYTVLRSPHKHKDSREQFEMRTHKRLIDIVNPTPKTVDSLMKLDLPSGVDIEIKL

>fig|1664.9.peg.732

MDKAALLIIDYTNDFVAPDGALTAGKPAQDIASTIVTLADQFLAKKQFIILPTDLHVANDPFHPEAKLFSPHNLVDSRGRAFYGPLANWYAEHQDSPYVYAFAKNRYSAFANTNLDNFLRERQITDLHLTGVCTDICVLHTAVSAYNLNYPLTIHKKAVATFTPNGQEWALAHFKNSLGATII

>fig|1664.9.peg.733

MANKREFPLDKTRNIGIMAHIDAGKTTTTERILYYTGKIHKIGETHEGASQMDWMEQEQERGITITSAATTAEWKGNRVNIIDTPGHVDFTIEVERSLRVLDGAITVLDAQSGVEPQTENVWRQATTYGVPRIVFVNKMDKLGANFDYSMTTLEDRLQANAHAVQMPIGAEDEFQGVIDLIEMQADIYDEDELGAKWDTVDVPADYLEQATKRRAELVEAVADVNDDIMDKYLEGEEISKEELKAAIRQATIDLKFFPVFAGSAFKNKGVQMLMDGVVDYLPSPLDVRPYNAKNPEDDSEVELMAGDDKPFAGLAFKIATDPFVGRLTFFRVYTGTLQSGSYILNATKDKRERVGRLLQMHSNHRNEIPEVFSGDIAAAIGLKNTTTGDSLTDVDHPLILESMEFPDPVIQVSVEPESKEDRDKLDLALQKLAEEDPTFKAETNNETGETLISGMGELHLDIMVDRMRREFKVVAKIGEPQVAYRETFTKQASAQGKFVRQSGGKGQYGDVWVEFTPNEEGKGFEFEDAIVGGVVPREYIPSVEQGLKESMANGVLAGYPLIDVKAKLYDGSYHDVDSNESAFKIAASMALKNAAKQAGAEILEPIMKVEVIAPEEYLGDIMGQVTARRGAVEGMEARGNAQIVNAMVPLSEMFGYATTLRSATQGRGTFTMVFDHYSAVPKSIQEEIIKKNGGQ

>fig|1664.9.peg.734

MPRKGYVAKRDVLPDPMYNSKLVSRLINRLMIDGKRGTASTILYDAFDIIKEETGNEPLEVFEEAMNNIMPVLEVKARRIGGSNYQVPIEVRPERRTTLGLRWLVSYARLRGEHTMDQRLAREIMDAANNTGAAVKKREDTHKMADANRAFAHYRW

>fig|1664.9.peg.735

MPTINQLVRKGRKSRTSKSDAPALNFGYNSMKKKATDNPAPQKRGVATRVGTMTPKKPNSALRKYARVRLSNLIEVTAYIPGIGHNLQEHSVVLIRGGRVKDLPGVRYHVIRGALDTAGVDGRMQSRSKYGTKRPKK

>fig|1664.9.peg.737

MTTIDYAKTALVVIDLQPGIADRPTLAPHAGETVVQNAQQLIEDFEAHQGFVVLVNVGVTDGQDQLTPNLGERTPDYRQPSPLLNTRLLAEIANYKNAHQITKRQWGAFYGTDLDLQLRRRGIETIVLCGISTNIGVESTARDAFEHNYHQIFIEDAMTASTAIEHQHSLQYIFPRMGQVMSTASFLEKK

>fig|1664.9.peg.738

MIDVNKFESMQIGLASSDKIRSWSYGEVKKPETINYRTLKPERDGLFDERIFGPTKDWECACGKYKRIRYKGIVCDRCGVEVTRSKVRRERMGHIELAAPVTHIWYFKGIPSRMGLVLDMSPRALEEVIYFASYVVIDPGDTALEKKQLMTEREYREKLDEYGNKFNAKMGAEAIKELLQDVDLEGEVAELKENLKSAQGQKRTRAIRRLDILDAFRKSGNRPDWMVMDAIPVIPPDLRPMVQLEGGRFATSDLNDLYRRVINRNNRLKRLLDLMAPNIIVQNEKRMLQEAVDALIDNGRRGRPVTGPGNRPLKSLSHMLKGKQGRFRQNLLGKRVDYSGRSVIDVGPTLKFYQCGLPREMALELFKPFVMHELVKRDMASNIKNAKRKIDRQDDDVWDVLEDVIKERPVLLNRAPTLHRLGIQAFEPILVDGKSIRLHPLACEAYNADFDGDQMAIHVPLSDEAQAEARMLMLAAHHILAPKDGKPIVTPSQDVVLGNYYLTLEQKNRLGEGMIFKDTNEVLMAYQTGHVHLHSRIGLSVADMANRPFTDDQRHKIMVTSVGKVMFNEIMPADFPYLNEPTTDNLMNGVPDKYFIDNGEDIHDYLAGAPVILPFKKGFLSDIIAQVFKVYKVQRTSDLLDDMKTLGYVQATHAGLTVGVADVPQLPEKQEIVDDAHKKVTTISKQFRRGLITDEERHARVIEIWNDAKDDIQQRLTDSFDPTNPISMMSDSGARGNISNFTQLAGMRGLMAAPNGGMMEVPVTSNFREGLSVMEMFMSTHGARKGMTDTALKTADSGYLTRRLVDVAQDVIIREEDCGTDRGLLIHSLREGNEMIEPLYDRLVGRCPMKTIVNPTTGEVMVAKNQLMDEQLAQAVVDAGVEEVTIRSVFTCNTKHGVCKKCYGRNMANGEEVEVGEAVGTVAAQSIGEPGTQLTMRNFHTGGVAGGEDITQGLPRIQEIFEARNPKAIATITEVTGEVIAVEENPAEHTREITVQGETDTRSYSVPYTSNIIVSEGDMIHRGEKITGGSVDPKQLIKVRDVLSTENYLLHEVQKVYRMQGIEIGDKHVEVMVRQMLRKVRVMDPGDTEILPGTLMDISEFTDRNHDTLISGGVPATCRPVLLGITKASLETNSFLSAASFQETTRVLTDASIRGKSDPLLGLKENVIIGKIVPAGTGMAKYRHMEPKTVGAVSENVYSISDIEAQMKQEETKE

>fig|1664.9.peg.739

MAGHLVNYGKHRTRRSYARIKEVLELPNLIEIQSNSYQWFLDEGLREMFDDIMPIDDFAGNLSLEFVDYQLLEPKYTVEEARQHDANYSAPLHVTLKLTNHETGEIKSQDVFFGDFPLMTDQGTFIINGAERVIVSQLVRSPGVYFNSAIDKNSRTTYGTTVIPNRGAWLEFETDAKDIAYVRIDRTRKIPMSVLVRALGYGSDQEIIDILGDNDSLMLTLEKDIHKNTDDSRTEEALKDVYERLRPGEPKTADSSRSLLFARFFDAKRYDLASVGRYKINKKLSLKTRLLGQTLAETLADPDTGEVIAAKDTLVDRQVMDALAPYLDQEDFKAVTYQPSDEGVLPEPMTLQVIKVYSQKTPDKEINLIGNGHIDAKVKHVIPADIIASMNYFFNLQEGLGSTDDIDHLGNRRIRSVGELLQNQFRIGLSRMERVVRERMSIQDTSTVTPQQLINIRPVVASIKEFFGSSQLSQFMDQTNPLGELTHKRRLSALGPGGLTRDRAGYEVRDVHYTHYGRMCPIETPEGPNIGLINSLASYAVVNRYGFIETPYRRVSWDTHDVTDKIDYLTADEEDNYVIAQANSPLNDDGSFVDNTVLARYKDDNIETSIDKLDYMDVSPKQVVAVATACIPFLENDDSNRALMGANMQRQAVPLVNPHAPLVGTGMEYKAAHDSGIALLAQHAGTVEYVDAKAIRVRREDSSLDTYELMKFRRSNAGKNYNQRPIVAKGDHVDVDEIIADGPAMEKGELALGQNPLIAFMTWNMYNYEDAIVLSERLVKEDLYTSIHIEEYESEARDTKLGPEEITREIPNVGEDSLKDLDEFGIVRVGAEVKDGDILVGKVTPKGVTELSAEERLLHAIFGEKAREVRDTSLKVPHGGGGIIQDVKIFTREAGDELSPGVNMMVRVYITQKRKIQVGDKMAGRHGNKGTVSIVVPEEDMPYMPDGTPVDILLSPMGVPSRMNIGQVLELHLGMAARNLGIHVATPVFDGAQDKDLWDAVREANMPSDGKSILYDGRTGEPFDTRVSVGVMYYMKLAHMVDDKLHARSIGPYSLVTQQPLGGKAQFGGQRFGEMEVWALEAYGAAYTLQEILTYKSDDVVGRVKTYEAIVKGEPIPKPGVPESFRVLVKELQSLGLDMKVLDIDNQEIELRDMDDDDDDVVNVDALSKYAKEQEEKKAQQEAEKAQAASAEDPSAE

>fig|1664.9.peg.740

MTYSEQAQTGKKEYAALKEASPEVMGAFGDLHKANVKDGALTVKEKELIALGIAIATRCEGCILSHMNRLIKEGVSRDEVIETINTAVMMSGGPGTVYGGKALAYFDERQ

>fig|1664.9.peg.741

MKRISTVLLSIGTIALLLAGCQGKQASQGDSKAQSAKITAVGSTALQPLVEQAGATYQTKHAGIDITVQGGGSGTGLAQVSDGSVTIGNSDIFAEEKAGINAKKLVDHKVAVVAIAPVVNEKLGIKSLTQAQLKDIFTGKIKNWHAVGGPDQSITVVNRAQGSGTRQTFENLGLKTNKVMTSQEQDSSGTVQKIVRQTPGAISYLAFPYIKDGLKAVALDNVQPTTKNVLTNQWPIWSYEHAYTKGAPNYQTAKFIHYLQSKDVQKTLVPKLGYIPMTQMKVERHADGSIQDVK

>fig|1664.9.peg.742

MINNKMKTLSDKYPTKMAVTSEIINLKAILNLPKPTEAFMSDLHGEYDAFQHLIRTGAGNLRQKINELFSGEMTPETMQAFAFLVYYPTERLALKHQALSENELNQWYLTTFKRMIDLLKFVSTKYTRSKVRKAMAPDFVYITEELMYGDVANVDKKRYFQEITATIIELGQADALIIATSHTIQRLVVDQWHIIGDIYDRGPHPDLIVDQLTQLPAVDVQWGNHDILWFGAASGSELCLLNLLRICARYNNLAIIEETYGIDLSNLVRFAAQHYQANPAFMPVEDPNQGPLTRAEKLKISQVQQALAIMQFKLELTVIKRHPEFNMDHRLLLSQVDFKRRILHLNGQEYPLENTCFQLVDPENPEALTAEESQIIADLLAAFTRCQKLRKHLTFLIDHGSMYRIYNQNLLFHGCLPVDAEGHFLTLTLANQHYAGKQLLDFFDQQIRSSFNHPLHQANLSTDLLWYLWTGPLSPLFGKNAMTTFERYFCPDPETHVETKNAYYSLRHDADFIQQLLSEFNLSPETGHILNGHTPVKKGHDPIMANRQMIVIDGGFSKAYHHTTGIGGFTLLYNSYGMQLVTHQPFTTKADAIANMKDIISTRRVIDQVSQRQRVSQTNIGAAIKTEIEQLQTLLTIQPDH

>fig|1664.9.peg.743

MNNLFTPSAKNVLMLAQEQAKKFNHHALGTEHLLLALVLESEGIAGNALRELGVTPTDVLEEIERLTGYGDSIVAMGVGGYLPYSPKAKQVLDLARVESQQANSVKIGTAHLLLALLRDDDIIAARILLNLGLSLAKTRQLLLQKMGIDAATAKKRAKASAKKVDQEGTPTLNQLARDLTQMARENQIDPVVGRDSEVKRLVQILARRSKNNPVLIGEPGVGKTAIAEGFAQRIINGDVPSDMQQKRLMMLDMGSLVAGTKYRGEFEDRLKKIIEEIHEDGQVILFIDELHTLIGAGGAEGAIDASNILKPALARGELQLIGATTLDEYQKYIEKDAALERRFATVTVDEPTPEDAEQILHGLRPRYEEHHGITISDEALHEAVILANRYITNRFLPDKAIDLMDEAAAKVRLDAVNQKSPIDRLETELQSLAKEKEVAVGAQDFEKAAAIHEKEIIAKAKMAKRQAAEQENGVRTDIQVQPEDIAQVVAQWTGVPVTQLQRKESERLLQLEKVLHERVVGQEEAISAVARAIRRARSGLKDPKRPIGSFMFLGPTGVGKTELAKALAEAMFGSEDNLIRVDMSEYMERYSTSRLVGAAPGYVGYDEGGQLTEKVRNKPYSVVLFDEVEKAHPDVFNILLQVLDDGYLTDSKGRKVDFRNTIMIMTSNLGATALRDDKSVGFGVKDVTADYKAMQGRILEELKKSFRPEFLNRIDETVVFHSLTQPELREIVKIMSKGILNRLAEQGVKIKMTAAAMDVVAKAGFDPEYGARPIRRALQTQVEDRLSEALLAGEITTDTPVTIGATKGEITINSKKEKTTVK

>fig|1664.9.peg.744

MQSQNISDIIEAYLKKILADSEQIEIRRSEIAKLFNCVPSQINYVINTRFTEQRGYVVNSKRGGGGYIRIVKVQFLDDRDFLEALIGSVEDRISQADTLAIIQKLYDEQILSQKEGNLVLAMLNAQTLAVNDKQLEEQLRARMLIAVLERLRYEIK

>fig|1664.9.peg.745

MQKRYLSVLAGLLIVGLLLTGCRTKKADPNTIVIGASTIPHAAILRHIKPELKKEGITLDVKAFSDYVLPNKALASGELTANYYQHIPFMKKANHDNGYHLVSAGGIHLEPLGLYSKRVKKLSAVKTGATVLVSNSQTDWGRVLSILKTGGLITLKPGVKVENATFNDIAKNPKKLKFKYTFEPKLMPELLKNDEGTLVAINSNYAVQAGFTPEKDAVAIESLSSPYVNVVAVNKGDENKPAIKKLIKALKSKSTQAWIKKQWHGAVLPVK

>fig|1664.9.peg.746

MANINGFQSYFQFKQIDWPTMWEATVETVWLTAVSVLLVAILGFLLGLVLYQTNGKMDFMSRLLNGIVALFVNVFRSVPFIILIVLLIPMTKELMGTMIGPKAAIPALIVSAAPFYARLVELAFREVDSGVIEATRALGASKWQIIWQVLLPESMPALVSGLTVTAISLLGYTAMAGVIGAGGLGNLAYTEGFQANQNTITLVATIIILIIVFICQKAGDIIIAKIDHR

>fig|1664.9.peg.747

MLELKQVGKVYQREQQTFTVLSDISLKIEAGEIYGIIGYSGAGKSTLIRLLNGLEKPTSGDVLVNGQSIVGLSEQAMRPIRQKIGMIFQHFNLLWSKTILENIALPLKLAGVGKKERLAKAKEMLALVELTDLGAAYPSELSGGQKQRVAIARALISEPAILLCDEATSALDPKTTNSILSLLAKINQETGITIVLVTHEMDAVRRICQRIAVMAAGRVIEEGTTQAIFETPQAEVTKAFVSESLVITPSETAASIEQLLTRVPEGTIVKLRFNEEQSSQPVIGQLMRRYPSVEVSVISGSLQQTINGALGYLYIQIQASPDDLKQALSDLSQQTIEVEVLRHG

>fig|1664.9.peg.748

MTMSNNDYFWTEEIAASIRRIGLTKGAQNEFGLIKFVDVPVEGQKMALDMPLVAIEAEKMVQELDSPLAGELVNVNTDLADNPELLNADNEKDAWIADIRISDN

>fig|1664.9.peg.749

MPKELEKVDDTSRVDYGIVFSVFMLALVGLMSIYVAVTHDTSSASSTRAVLMQAAWYVIGGIAIVAIMQFDSEQLWRVAPIAYGIGIFLLVAVLFFYNRVVAIQTGAKSWFSLGPISFQPSEVMKPAYILMMSRVVTKHNTEYPEHTINADWLLLGRLLIWTLPVMVLLKLQNDFGTLLVFVAILGGIILVSGITWKILAPAMAIMAAIGGTALTLAVTTPGRAILTHLGFKTYQFNRIDAWLHPFDNTASTSLQLSQSLKAIGSGQLFGKGFNQIQVNVPVRESDMIFSVIGENFGFIGSCLVILLYFLLIYQMIRVTFDTKNEFYAYVSTGVIMMILFHVFENIGMSVGLLPLTGIPLPFISQGGSSLLGNMMGIGFIMSMRYHYKSYMFSESEDTF

>fig|1664.9.peg.750

MSKKEQRVEVTIDDINRNGQAMTSVKVGKNEVGVIVEDSAKKRFEATVEGTGQSIITKTHDDAVNELLREYHLHQG

>fig|1664.9.peg.751

MIQAIQKHGAIKGLIMGIGRILRCHPFVKGGYDPVPDHFTVFRNKSVND

>fig|1664.9.peg.753

MAKDIGIDLGTANVLINVMDKGIVLNEPSVVAIDTKTKKVLAVGSEAYSMVGRTPGNILAVRPLKDGVIADFDMTEAMLAYFINKLNVKGFLSKPNILICCPTNITSIEQKAIVEAAEKSGGGKVFLEFEPKVAAVGAGMDIFQPRGNMVIDIGGGTSDIAVLSMGEVVTSRSLRLAGDRMNAEIAAYVKRKHNLLIGEHTAETIKLKIGSVWHADASEQLEVRGRDIVGGLPVSITIGADEVELALHETMMAIVAAAKEVLEVTPPELAGDIIDRGIMLTGGGALLRNIANLFSEHLKVPVVLAEKPLDAVALGTGVLLENIAQKRAH

>fig|1664.9.peg.754

MEKLIIQGGQPLVGDVHIEGAKNAVLPIMAAALLASKGPVELTNVPILSDVFMMQDVLKSLDARVKFDEQRNYLMIDANQPLNFEAAFEYVSKMRASIVVMGPLLARLGHARVAMPGGCAIGSRPVDLHLKGFEALGATITQSHGYIEAKADQLVGANIYLDFPSVGATQNIMMAATLAKGTTVIENVAREPEIVDLANVLNKMGAKVFGAGTEEIRIEGVTELKGTEHSIVQDRIEAGTFMIAAAATKGNVLVEEAISEHNKPLLSKLAEMGAQVIEEENGIRIIGPDELKPSNIKTLPYPGFPTDMQAQMTALQLMAHGTSVMTETVFENRFMHLEELRRMNADYQIEGQSVILYGPTELTGAEVAASDLRAAAALVIAGLAANGETLVTNLQYMDRGYYHFHQKLRALGAHISRADFDEAGHRKTTQKLA

>fig|1664.9.peg.755

MQSIGSQAVVTIISHIGFIVLSFYGLQSLRLEQLFKPNHIRQIQIVLMFTAIVMGYLVSQFFLEIIAQARNIIFLVQ

>fig|1664.9.peg.756

MMAEEQKVLTVNIVTPDGVVYDHHASMLVVPAMAGQLGIMANHEPIITPLEIGEIRVKRTDNPGHEDAIAITGGFMEVSHNIASIVADGAERARDINLSRAQRAKQRAEDAIKTASEKHDSDELRRAQIALQRAMNRIDVKNHLQ

>fig|1664.9.peg.757

MSMGKVVQVIGPVVDVEFSLDTDLPDINNALTVDKGNDETVVLEVALELGDGVMRTISMESTDGLRRGMPVEDAGRAINVPVGKETLGRVFNVLGETIDGGEEFPADFRRDSIHRSAPKFEELNTSSEILETGIKVIDLLAPYVRGGKIGLFGGAGVGKTVLIQELIHNIAEEHGGISVFTGVGERTREGNDLYFEMKESGVLEKTAMVFGQMNESPGARMRVALTGLTIAEYFRDVEGQDVLLFIDNIFRFTQAGSEVSALLGRMPSAVGYQPTLATEMGQLQERITSTKKGSVTSIQAIYVPADDYTDPAPATTFAHLDATTNLDRKLTQQGIYPAVNPLESSSSALDPEIVGQEHYEVASEVQHVLQRYRELQDIISILGMDELSDDEKIIVARARRIQFFLSQNFHVAEAFTGQAGSYVPVKDTVSGFKAILAGDYDDVPEEAFRLVGNIDAALAKAKEMGYTQSEKAVDQD

>fig|1664.9.peg.758

MAESLMDIKRRIASTKKTGQITSAMQMVSGAKLSQIEKNSVAYQVYTDKIREIVTHLAASQLIDIARQKSSLQAEPADSTIKKAIKHEVTLSNLLVERPIKKTGYLVITSDRGLVGAYNSSILKAMVQMISETHQSPDEYAILAVGGTGADFFKARGMNLTYEYRGVSDVPSFEEVKQIIKTAVAMYDNGVYDELYVCYNHHVNSLTSGFRAEKMLPITDLDVSEVADQNLEYITEPSVDDALDAILPQYAESLIYGAMLDSKTAEHAASMAAMKSATDNANNLISELSIKYNRARQAQITTEITEIVGGAAALE

>fig|1664.9.peg.759

MSIKTEEISALIKQQLANYNEELAVEEVGTVTYVGDGIARAHGLENALSSELLEFSNGSYGVAQNLETNDVGIIILGEYENIREGDQVKRTGRIMEVPVGDALIGRVVNPLGQPVDGRGEIKTDKTRPIEKKAPGVMARQSVSEPLQTGLKAIDALVPIGRGQRELIIGDRKTGKTSVAIDTIINQKGQDMICIYVAIGQKESTVRTQVETLRRYGAMDYTIVVEAGPSQPAPLLYIAPYAGAAMGEEFMYNGKHVLIVYDDLSKQAAAYREISLLLRRPPGREAYPGDIFYLHSRLLERAAKLSDELGGGSMTALPIIETQAGDISAYIPTNVISITDGQIFLESDLFYSGTRPAIDAGSSVSRVGGAAQTKAMKKVSGTLRLDLSSYRELEAFTQFGSDLDAATQAKLNRGKRTVEVLKQPLHKPLPFEQQTVILYALTHGFIDDVPVDDIMRYESGLNDYLESNAKDLMDEIRQTTKLPDTDKLDAAIKAFTEGFVPEQAVDDKDSDK

>fig|1664.9.peg.760

MKLDKYAVGQRYAKALFSLAEQEQQFESIHDEIQALETIFNDNPKLGTVLTDTTLSGLKQRNLLESLSNDFSTLMQHFLSLVFDYQRMAEMPYIIAAYEDLYDQHKGIAHAKVTSAVALDDDQLAKISQSFAKREGLNEVLIESVVDPDIIGGIVLESNHKVIDGSVKHGLDQIKSLLLK

>fig|1664.9.peg.761

MFSNLIVGASASYLGDSLFVLVVFIILVALVGKFAFGPVSKMMQERSNKITNDLDSAAQSREDAAKLAAQRATELKSSKSEAVEIVNTAKQNGEKQREGMITLAQEEVQTLKQNAKKDIEQSRLDALNSARDDVAQLSIEIASKLIKKELSVADQKSLINSYIEGLDKQNETR

>fig|1664.9.peg.762

MNFLAAAIAAGLAAFAASYGNGKVISKTIESMARQPELSAQLRSTMFIGVGLIEAVPILSIVVSFLILFS

>fig|1664.9.peg.763

MGDKYPIISILGIRFNLANCLSVLLSAAIVFALVFFLSRKIALKPTKRQNVLEWMIDFTNGIVKSAMPGEEGKQFYLFAFVMFLFVFISNQFGLIFQMKVDEVVWLKSPTADPIITMSLAMIVLVLSHYFSIERLGFGNYLKSYIKPVGFFLPINIIEQFTNFLTLSLRLYGNIFAGEVLLNLLVKMAFSHGPATMLVTAPIQMVWQGFSVFIGSIQAFVFVTLSMVYISEKVEMED

>fig|1664.9.peg.764

MAKFTVLNHPLIQHKLTIIRNKNTGTKVFREVANEIAELMVYEITRDLAMEDVEVETPMGPAIEKQLSGKKLAVVPILRAGLGMVDGVLELIPAAKVGHIGMYRDEKTLQPHEYFVKLPTDIDQRQLFIVDPMLATGGSAIMAIDALKKRGATSMRLVVLVAAPEGVKAVQEAHPDVDIYAAGLDDGLNEEGYIYPGLGDAGDRLFGTK

>fig|1664.9.peg.765

MLAKTDPVINDLIKQEENRQRHNIELIASENIVSGAVQEAQGSVLTNKYAEGYPNKRFYGGCEYIDEIETLAIERAKELFGADHVNVQPHSGSQANMAVYQALLEPGDKILGMNLTDGGHLTHGSPFNFSGQLYDFYSYGVADTNEQLDYASLAAKAQEVHPKMIVAGASAYSRTIDFPRLREIADQVGAYLMIDMAHIAGLVATGVHPSPVPYADVVTTTTHKTLRGPRGGMILCKTEYAKAIDSAIFPGIQGGPLEHVIAAKAVAFGEALQPEFTAYTKQIVANAQAMAAVFDQSDLVRVVSGGTDNHLMLLDLTNSGLNGKELQNLLDSVHITVNKNTIPFEKLSPFKTSGIRIGTPAITSRGFKETDCEQIANLILEVIEKYDQLEAMTAISEAVLKLTDQFPITQAKFLD

>fig|1664.9.peg.766

METKRYTVNQIEQAAAALNAGELVSFPTETVYGLGADATNPAAVKKVYAAKGRPSDNPLIVHVASVETVEHYAVTSNPAFGQLVKAFWPGSLTIILPLKHGAFDAVVTGGLKTAAFRMPDNQVTLSLIKAAGVPIVGPSANTSGKPSPTLADHVLHDLTGKIAGVIDDGPTGVGLESTVIDLSVATPVILRPGVVTQEAIERVIGPIMTNKHKVGAAETPKAPGMKYKHYAPNAQVHIVANPDDFDAAIDWASQQAVPFGVMAVDTILEKQAKVRFKDQFSLGESVVTASQHLFEGLRYFDLNPEVKLILVQGFAKEGVGLAYMNRLEKSAGQKYFSKQQAD

>fig|1664.9.peg.767

MAQLTYLKALNWAFLFLEERNKEHEAARFLLLGRHHWTTTQLVLHYRDEMPANEYEQYQADLAAFAEDQPAQYILGYANFYGRDFKVTPATLIPRLETEELVEWVLSVAPVKDRPLKVLDVGTGSGAIAITLACERPDWQVAAVDISPAAIAVAQQNAQTLGAKVDFIEGDFLTPVMGQQFDVIVSNPPYIAEDERSVMDASVLKHEPDLALFAPNNGLAFYERFAQEVPAFLEPEGALFLEFGYQQKEAIVRIFEQRDPLFSLEVQKDMANWSRMMRVSYDFNRNLLL

>fig|1664.9.peg.768

MDKMFEQLDGLLDRYAELQELMSDPEVINETSRYMELSKEEAGLREIVGKYTRLKEVLSEVTENEELLRETTDSEMTELVKADLEELQTEKAQLEQEIKILMLPTDPNDEKNIIMEIRGAAGGDEASLFAGDLLNMYQRYAESQNWQTEIIDETATEVGGFKEVAMMITGKNVYSKLKYENGAHRVQRIPKTESQGRVHTSTATVAVMPEYDGVDVELEAKDIRVDVYRASGAGGQHINKTSSAVRMTHIPTGIVVAMQDQRSQQQNRVKAMKILQARVYDYYESQNQSEYDSSRKSAVGSGDRSERIRTYNYPQNRVTDHRIGLTLNKLDRIMNGELGDVIDALILFDQTEKLEQLQDGTAHLS

>fig|1664.9.peg.769

MTQLFFRYGAMNSGKSIEILKVAHNYEEQNKSVIILTSGIDNRDGVGIVSSRIGLKREATPIFSDTNIYELVEATNPDAACVLIDESQFLERHHVIEAAKVVDDLNIPVMAFGLKNDFKNELFEGSKYLLLFANKIEEMKTICWFCRKKAIMNLRMNNGLPVYTGEQIQIGGNEAYYPVCRKHYFNPPIQEEIEKEG

>fig|1664.9.peg.770

MTIKSSFATTTGRFSYWFLHTFMKGGSSLPGQIATNLDPAVLKNLTKNYDVIIITGTNGKTLTTALTVQTLKQKYDNIMTNSTGSNMMQGITTAFLAHHPKKGQKQLAILEVDEANVEPLCRYVEPKAIVMTNIFRDQMDRFGEIYTTYNKILAGVKLVPNTTLIMNGDVPLFSSVELPNPKIYYGFNHQPDGEMLAPSNTDGVLCPVCQHIIHYKFISYSSQGKFYCPNCGFKRPELTDQVTAINEMRPTSSQFEINQAPFEISIGGMYNIYNALAAYSVGRFLDVPVSDIQKAFTQNKKVFGRQEVIQLKDKAVTLVLVKNPVGLNQVLQMISSDPEDFSLAMLLNANYADGIDTSWIWDGEFEDLLKHHIPTVITGGERRDDITFRLKVAGVTDEQLHETADLDATLAAIEAAPTKHVYVLATYTAVLQLRKKMAEANYIKKGLSV

>fig|1664.9.peg.771

MTQTLKICHLYGNLMNTYGDLGNILVLDYYAKKIGLKTETDVISIGQDFDASRYDIVLFGGGQDFEQKIISEDLTSKKAALTAYIENGGVMLAICGGYQLLGHYYIGANGDKIPGIGALDHYTLSQDNSRFIGDIVIENREFNETYLGFENHNGRTFLGKDEKPLGYVKRGHGNNGEDGTEGCVYKNTFGSYFHGPILTHNGVLAKRLLTTAISRRFPEFDLQPLADLPIEESRYQVDAAGVKKVQN

>fig|1664.9.peg.772

MNYIYAFAEDQTLTNQELGGKGANLAEMTRLGLPVPAGFTITTAACRQYLTSAKQETGFLEDELMKAVHILEEKTQRQLGNAEQPLLVSVRSGAPISMPGMMDTILNLGLNDQTVIGLAEMTNDPWFAYDCYRRLIQMFGDVVYGIDKPIFEAALTALKNKRQVQKDTDLTLVDLKQLIETYKGLYVAAGQYQFPQSVDQQLRLAIEAVFKSWQNPRAQTYRRLNQIDGAMGTAVNVQQMVFGNYQGQSGTGVAFTRNPATGEPGLFGEYLLNAQGEDVVAGIRTPEPIATLKAQLPVIYEQFKTIANQLEAHYRDMQDMEFTIEAGQLYVLQTRVGKRTAPAAFRIAVDLVDEGVIDRPTAISRLKPTMIDGLLHPLFETSAVQQATQLLTGLPASPGAATGAIYFDAQKAQQAHEAGEKVVLVRQETSPEDIDGMVISEAIVTSRGGMTSHAAVVARGMGCCCVVGCQQLQVDYEQREAHFAGRVLKEGAIISVDGHTGRLYLGALPQQTGVHETTLGTVLSWCDEIAPFAVWANAETPQEVATAFEFGAQGLGLVRTEHMFFGPERIFKMRQMILATELADRQVALAALKALQINDFKQIFSLAEERPCTIRLLDPPLHEFLPQNEVEQAELATALGISLAEIKRRIADKAEVNPMLGHRGSRLAVTYPEIYQMQVLAIVESALAVQTTYHHAVTPKIMLPLIGTATEMAYLKQLLQQTINDYLVENQQQLTYQIGTMIEIPRACLVADQIAATADFFSFGTNDLTQMTYGFSRDDIGHFMPDYQQQGLMPAEPFQTLDQDGVGALMQLAVQKGRQTKPDLSIGVCGEVGGDPTAMPFYRDLGIDYVSCSPYRVPSARLAAAQSQLTLAD

>fig|1664.9.peg.773

MELSNRQQAIIKIVKGNQPISGTKIAEQLHLSRATLRNDFAILTMTGILDARPKVGYFYVGQEVMPLLTDELYQETVGQLMVPPLLIPQTTTIDEAVTQLFMHDVGSLYVTDEQSGLLGVLSRKDLLRATINTTNTQATPVAMIMTRMPNITTTTEDTPVLHAGQRLIAHQVDSLPVVAIDGITVIGKITKSIIMRYFIEAGLHNQS

>fig|1664.9.peg.774

MTAIPIFIISDSIGETARTVIAAVNAQFPASVTLKIQRFPFITDQKTLTPILQDAHQEQAIIVTTLVNHILQETVTQFCQVKHLTLIDLLSPLTTAISERSQTASLETPGSLRKLDEHYFHRISAMEFAVRYDDGQDPRGLLEADIVLLGVSRTSKTPLSMYLANQNYRVANLPLIPNVPLPKELFKVPAHKIIGLTMPLSTLLKIRQERLATLGLPQTTNYSNMTTVGDELAYANQIFEQLNATTINVADRSIEETASLIQTLI

>fig|1664.9.peg.776

MQKQIEAIEQFVRTALAQEHSGHGFDHIQRVVNNARLIQRQVGDGDLTTITLAALLHDVIDEKIVSDVAQARQSVAAVLTQNGIDKMQSTVIFDIIDHMSFSKNINQHQALSLEGQIVQDADRLDAIGAIGIGRTFMYGGAHDGVAYDPEIQPRAELTKANYREPSTVVNHFYEKLFKLAATMNTQVAREIATARTQVMQDFVAEYIAEWQGEK

>fig|1664.9.peg.777

MTEPLFLKPVFQEKIWGGRKLAEAFDYDLPDGDIGECWAISAHPHGPSTIENGPLKGLTLDQAWAQHRDYFGDAKGEVFPLLTKILDAEASLSVQVHPDDAYAAAHEGPTELGKTECWYILDAEPGAYLIYGHHAKNKAELTEMIEAGKWDDLLRKVPVKTGDFFYVPSGTIHALNKGIMALETQQSSDTTYRLYDYDRVDAKTGEKRELHLKQSIDTTIVPHQDPVLNIQTEHVGDSTITTYVQPPMSPFFSVYQWLVKGDLHFDRTTAPYTLVSVVDGEGELVADGQTYLIHKNMHFVLPFGIKSWDLKGNMQIIASEPGNK

>fig|1664.9.peg.778

MRFEQTIHQIEALVADEVVPGVSYAVIEGQQVTTKVLGYSELTPKLIPLEPGQLYDLASLTKVIGTTTLILRLLERGRLSLTTKVHSILPAFKDRRVTVLHLLTHTSGLEGYIPNRNQLAAPALKEALLTQLTVGPNFGKKVVYTDIGMLYLGWMLEVIYGKPIQDLIQSLVLTPLGMTESTFAPEASRAVPTELTTARGLIQGVVHDPKSYVLQNHSGAAGLFAPLDDVVRFAQFQLGQLKVAEAPVSQASVKGLYRDWTPAHLGRSLGWDLRFTADQTPLIYHTGFTGTFMLLDRKRQTGLVVLSNRIHPTADNQAFLDRRTMIVDQFLAEDKV

>fig|1664.9.peg.779

MAEKAATNRPQGGHGPGRGMGGVVEKPKNFWGTTNRLLKYMSSRLIALVFVIVLAIVSVIFQIRTPKVLGEATTEIFKGVMKGNAMQKAGIAMDRLPIDFDKIQHIIMTVILMYLASAVFSFLQQFIMTRISQQTVYELRKELKDKMQRVPINYYDTHSNGDIMSRAINDMDNIASTLQQSLTQMVTSTVTFIGVLWMMLTISWQLTLIALVTIPLSLIVIGIVAPKSQKFFAAQQKSLGLLNNQIEENFSGQVVLKTFNREQVTIDTFEEQNEKLYKAAWKAQFISGVIMPLMTFVNNLGYVFVAIIGGIRVSHGQVTLGDMQAFMQYTQQFSQPISQLANLANTIQSTIASAERVFAVLDEEDMSEEPSTVPVVQDDANQLEMEHVEFGYQGKDLLLTDYNLQVKPGQMIAIVGPTGAGKTTIINLLERFYDVSGGSIKLKGVDTRDMSREELRRHFAMVLQDTWLFTGTIWDNLKYGNESASDEAILAAAKAAHVDGFVRQLPEGYNTVLNEEASNISQGQRQLLTIARAFVADPDILILDEATSSVDTRTEIHIQHAMERLLQNRTSFVVAHRLSTIRDADNIIVMNHGSIMETGTHDELMVQDGFYADLYNSQFSGNVEI

>fig|1664.9.peg.780
[truncated: 209,768 more chars]
